# Total synthesis, isolation, surfactant properties, and biological evaluation of ananatosides and related macrodilactone-containing rhamnolipids†

**DOI:** 10.1039/d1sc01146d

**Published:** 2021-05-04

**Authors:** Maude Cloutier, Marie-Joëlle Prévost, Serge Lavoie, Thomas Feroldi, Marianne Piochon, Marie-Christine Groleau, Jean Legault, Sandra Villaume, Jérôme Crouzet, Stéphan Dorey, Mayri Alejandra Dìaz De Rienzo, Eric Déziel, Charles Gauthier

**Affiliations:** Centre Armand-Frappier Santé Biotechnologie, Institut National de la Recherche Scientifique (INRS) 531, Boulevard des Prairies Laval (Québec) H7V 1B7 Canada charles.gauthier@inrs.ca; Laboratoire d'Analyse et de Séparation des Essences Végétales (LASEVE), Département des Sciences Fondamentales, Université du Québec à Chicoutimi 555, Boulevard de l'Université Chicoutimi (Québec) G7H 2B1 Canada; Université de Reims Champagne-Ardenne, INRAE, USC RIBP 1488, SFR Condorcet-FR CNRS 3417 51100 Reims France; School of Pharmacy and Biomolecular Sciences, Liverpool John Moores University L3 3AF Liverpool UK

## Abstract

Rhamnolipids are a specific class of microbial surfactants, which hold great biotechnological and therapeutic potential. However, their exploitation at the industrial level is hampered because they are mainly produced by the opportunistic pathogen *Pseudomonas aeruginosa*. The non-human pathogenic bacterium *Pantoea ananatis* is an alternative producer of rhamnolipid-like metabolites containing glucose instead of rhamnose residues. Herein, we present the isolation, structural characterization, and total synthesis of ananatoside A, a 15-membered macrodilactone-containing glucolipid, and ananatoside B, its open-chain congener, from organic extracts of *P. ananatis*. Ananatoside A was synthesized through three alternative pathways involving either an intramolecular glycosylation, a chemical macrolactonization or a direct enzymatic transformation from ananatoside B. A series of diasteroisomerically pure (1→2), (1→3), and (1→4)-macrolactonized rhamnolipids were also synthesized through intramolecular glycosylation and their anomeric configurations as well as ring conformations were solved using molecular modeling in tandem with NMR studies. We show that ananatoside B is a more potent surfactant than its macrolide counterpart. We present evidence that macrolactonization of rhamnolipids enhances their cytotoxic and hemolytic potential, pointing towards a mechanism involving the formation of pores into the lipidic cell membrane. Lastly, we demonstrate that ananatoside A and ananatoside B as well as synthetic macrolactonized rhamnolipids can be perceived by the plant immune system, and that this sensing is more pronounced for a macrolide featuring a rhamnose moiety in its native ^1^*C*_4_ conformation. Altogether our results suggest that macrolactonization of glycolipids can dramatically interfere with their surfactant properties and biological activity.

## Abstract 2

We show that macrolactonization of gluco- and rhamnolipids dramatically interfere with their surfactant properties and biological activity.

## Graphical abstract

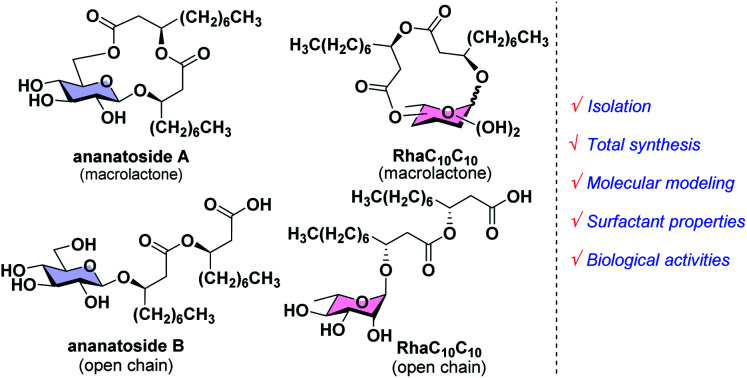

## Introduction

Bacteria represent a rich reservoir of structurally diverse glycosylated metabolites.^2^ Among these compounds, microbial glycolipids show considerable potential for biomedical and biotechnological applications.^3^ Microbial glycolipids are surfactants, *i.e.*, amphiphilic surface-active compounds, which are made by the combination of a lipidic chain covalently linked to a carbohydrate moiety. Because of their ability to form pores and destabilize biological membranes, microbial glycolipids have attracted increased attention as therapeutic agents.^4^ Glycolipids exhibit a wide range of pharmaceutical activities, including antibacterial,^5^ antifungal,^6^ antiviral,^7^ hemolytic,^8^ anticancer,^9^ and adjuvant^10^ activities. In addition, surfactants of microbial origin are increasingly considered for use in diverse biotechnological applications such as in the food and cosmetic industries as well as in bioremediation technologies.^3^

**Fig. 1:**
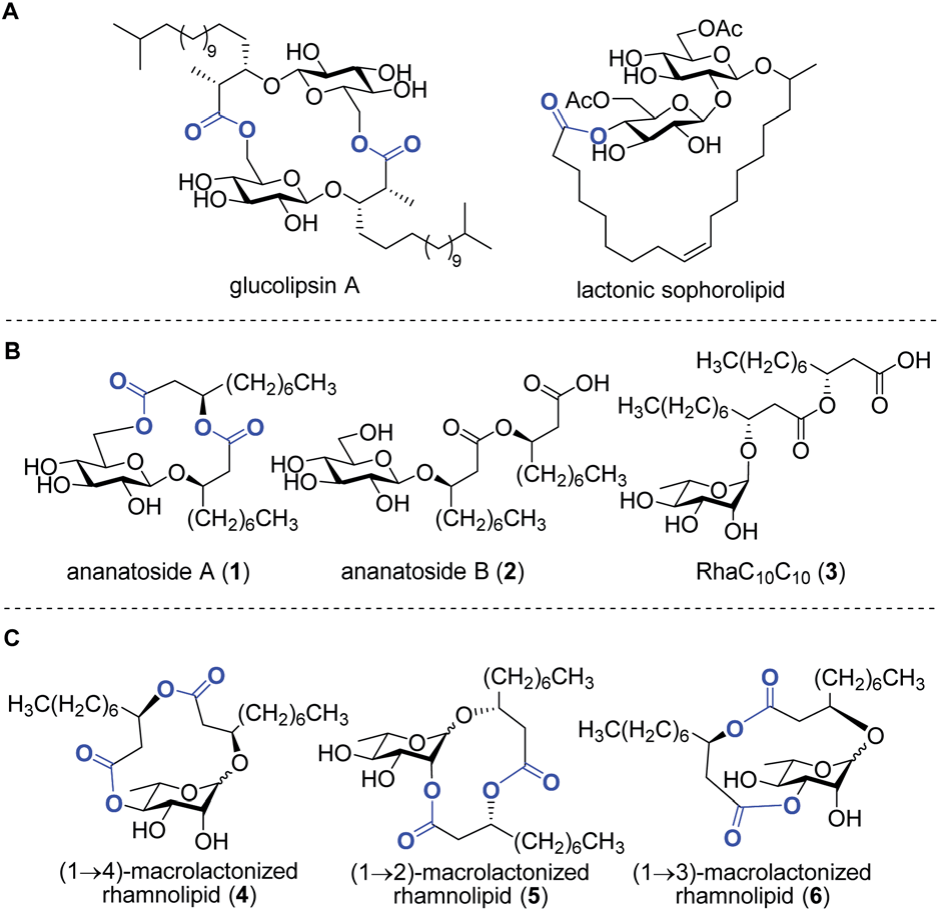
Examples of macrolactone-containing *gluco*lipids produced by microbes (A); structures of target ananatoside A (**1**) and ananatoside B (**2**) produced by *Pantoea ananatis* along with the related RhaC_10_C_10_ (**3**) (B); structures of target macrodilactone-containing rhamnolipids **4–6** (C). Lactone functionalities are highlighted in blue.

Rhamnolipids are a specific class of microbial biosurfactants that have been intensively investigated in recent years.^11^ Structurally, rhamnolipids are α-configured mono- or di-l-rhamnose residue(s) *O*-linked to an (*R*)-β-hydroxyalkanoic acid dilipidic chain of C_6_ to C_14_ carbon length (see RhaC_10_C_10_**3** in Fig. 1). As compared to the commercially available petroleum-derived synthetic surfactants, the biodegradability, low critical micelle concentrations (CMCs), and high tension surface activity make rhamnolipids exquisite environmental alternatives for industrial applications.^12^ Rhamnolipids are also of interest because they exhibit a plethora of intriguing pharmacological activities. They inhibit the formation of microbial biofilms,^13–15^ which play crucial role in infections caused by many pathogenic bacteria. They exhibit antimicrobial activities against both Gram-positive and Gram-negative bacteria as well as against plant pathogenic fungi.^12^ Because of their ability to interact with biological membranes, rhamnolipids can trigger the death of cancer cells^16^ and induce hemolysis of red blood cells.^17,18^ Furthermore, as invasion pattern molecules (also known as elicitors),^19^ rhamnolipids can stimulate the plant immune system resulting in the strengthening of plant cell walls along with the production of antimicrobial compounds.^20–23^

Rhamnolipids are mainly produced by pathogenic Gram-negative bacteria belonging to the *Pseudomonas* and *Burkholderia* genera,^11,24^ which hampers their exploitation at the industrial level since several are human pathogens. As such, there is an increased interest to identify non-pathogenic bacteria that can produce high concentrations of rhamnolipids and structurally-related biosurfactants. Our group has recently identified the bacterium *Pantoea ananatis* BRT175 as an alternative non-pathogenic producer of biosurfactants.^25,26^ The biosynthesis of rhamnolipids involves the successive function of three enzymes: RhlA, which directs the biosynthesis of the lipidic precursor, along with RhlB and RhlC, which are two rhamnosyltransferases.^27,28^ Although the production of biosurfactants in *P. ananatis* is also catalyzed by RhlA and RhlB homologues, we unexpectedly identified a *gluco*-rather than a *rhamno*lipid from *P. ananatis* ethyl acetate extracts.^25^ The isolated compound, that we called ananatoside A (**1**, Fig. 1), features an unprecedented 15-membered macrodilactone ring comprising a β-d-glucose residue linked to a C_10_C_10_ dilipid chain through both C1 and C6 positions.^26^ Biologically active glucolipids containing mono-, di-, and trilactones of different macrocyle sizes have been identified from other microorganisms^29–31^ including, to name a few examples, lactonic sophorolipids (Fig. 1), glucolipsins (Fig. 1), cycloviracins, fattiviracins, macroviracins, and anthrobacilins. By restricting the rotation of the substituents and stabilizing the active conformation of the glycolipid, the presence of the macrocycle is a prerequisite to the bioactivity of these constrained saccharides.^30,32^

The identification of ananatoside A (**1**) as a novel microbial macrolide prompted us to investigate *P. ananatis* extracts with the aim of identifying structurally similar biosurfactants. As part of our research program on the synthesis of microbial glycans,^33–36^ we are interested in developing synthetic routes that would allow an alternative and straightforward access to these macrodilactone-containing glycolipids, and enable the assessment of their tensioactive properties and biological activities. Within this framework, we herein present the total synthesis of ananatoside A (**1**), its newly identified open-chain congener ananatoside B (**2**), the related RhaC_10_C_10_ (**3**) as well as five unprecedented, anomerically pure (1→2)-, (1→3)-, and (1→4)-macrodilactone-containing rhamnolipids (**4–6**) (Fig. 1). We show that ananatoside A (**1**) can be efficiently obtained *via* three different pathways implying either a chemo- or enzymatic macrolactonization, or an intramolecular glycosylation as the key steps of these synthetic sequences. DFT calculations were used to decipher the ring conformations and anomeric configurations of synthetic macrolactonized rhamnolipids **4–6**, which were readily obtained through intramolecular glycosylation. Their surfactant properties and biological activity, *i.e.*, antimicrobial activity, cytotoxicity, hemolytic activity, as well as their interaction with the plant immune system, were also investigated, providing meaningful fundamental insights into the impact of the presence of the macrodilactonic ring on the physical and biological properties of this relevant class of microbial glycolipids.

## Results and discussion

### Isolation of ananatoside A (**1**) and ananatoside B (**2**)

The non-pathogenic bacterium *P. ananatis* BRT175 was found to be a producer of surface-active glycolipids based on its genetic homologies with rhamnolipid biosynthetic genes. The microbial biosurfactants were extracted with EtOAc from the supernatant of a liquid culture of BRT175, which was acidified to pH ∼3 prior the extraction. Purification of the crude extract was performed using a semi-preparative reversed-phase HPLC system equipped with a charged aerosol detector (CAD).^37^ The use of the CAD detector was particularly appealing over traditional UV/visible detectors as it allows the detection of molecules lacking chromophore groups such as glycolipids (see Fig. S1†). Through this optimized procedure, ananatoside A (**1**) was isolated as a yellow oil and its physical and analytical data (*R*_f_, specific rotation, HRMS, and 1D and 2D NMR) were in perfect agreement with those we recently published.^26^ A more polar and major congener (see Fig. S1†), that we have named ananatoside B (**2**), was also isolated from the EtOAc extract as a white amorphous powder. This compound was analyzed by HR-ESI-TOF-MS in the positive mode, yielding pseudomolecular ion peaks at *m*/*z* 538.3587 [M + NH_4_]^+^ and *m*/*z* 543.3142 [M + Na]^+^, pointing towards the presence of a glycolipid featuring a hexose residue linked to two hydroxydecanoic acid chains. Comparison of the 1D and 2D NMR data of ananatoside B (**2**, see Fig. S2† for main COSY and HMBC correlations) with our previously reported data for ananatoside A (**1**) suggested the presence of an open-chain β-linked glucolipid congener without the macrolide ring (no HMBC cross-peak between H6 and C1′′). The absolute configuration of both the glucose and β-hydroxydecanoic acid chains were determined following acid hydrolysis and measurement of specific rotation in comparison with an authentic d-glucose sample and literature data for the lipid chain, as we previously described.^26^ Based on these chemical and spectroscopic evidences, we established the complete structure of ananatoside B (**2**) as shown in Fig. 1. The structural determination of ananatoside A (**1**) and ananatoside B (**2**) were further confirmed through total synthesis as described in the next section.

### Total synthesis of ananatoside A (**1**) and ananatoside B (**2**)

#### Retrosynthetic analysis

The total synthesis of macrolactone-containing natural products has usually been accomplished *via* late-stage ring-closing metathesis or macrolactonization as key steps of the synthetic routes.^29,30,38^ Notwithstanding the success of these pioneering approaches, they present some drawbacks such as the use of highly diluted reaction mixtures and the possible di- or oligomerization of the acyclic precursors.^29^ Owing to their thermal stabilities together with their optimal regio- and enantioselectivities, commercially available lipases stand as a tantalizing synthetic tool for the formation of macrolactone rings from unprotected substrates.^39–41^ Alternatively, capitalizing on glycosylation chemistry,^42,43^ carbohydrate-embedded macrolactones can be built upon stereoselective intramolecular glycosylation using the ester-linked β-hydroxylipid as a stereodirecting tether.^44^ Importantly, the latter approach has only been implemented in rare occasions for the total synthesis of macrolactone-containing natural products.^29^

**Fig. 2:**
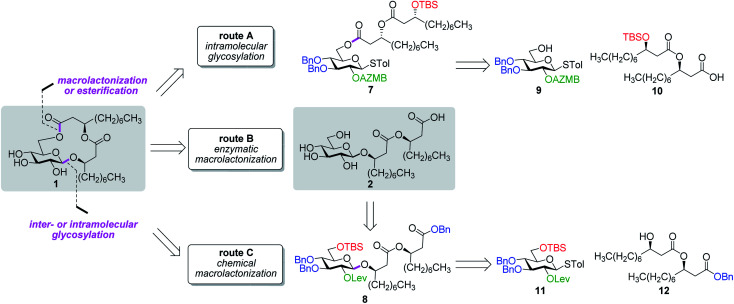
Retrosynthetic disconnection strategy for the total synthesis of ananatoside A (**1**) and ananatoside B (**2**) according to three different pathways: chemical macrolactonization, enzymatic macrolactonization, and inter- or intramolecular glycosylation. AZMB: 2-azidomethylbenzoyl; Bn: benzyl; Lev: levulinoyl; STol: thiotolyl; TBS: *tert*-butyldimethylsilyl. Blue: permanent protecting groups (Bn); red: temporary protecting groups (TBS); green: esters used as temporary protecting groups enabling neighboring group participation (AZMB and Lev).

Along these lines and as depicted in our retrosynthetic analysis strategy (Fig. 2), we envisioned to build the macrolide skeleton of ananatoside A (**1**) *via* three parallel synthetic pathways (routes A–C) as to maximize the chances of efficiently reaching our target. We first hypothesized that ananatoside A (**1**) would be formed through the intramolecular glycosylation of derivative **7** following chemoselective cleavage of the C6-*O*-TBS group (route A). Notably, precursor **7**, activated in the form of an STol glycoside,^45^ would be equipped with a (2-azidomethyl)benzoyl (AZMB)^46^ group at C2 that would act as a neighboring participating group enabling the formation of the 1,2-*trans*-glucosidic linkage. The β-selectivity would also be favored by the steric constraint exerted by the resulting macrolide.^44^ The propensity of the AZMB group to be orthogonally cleaved by means of, for instance, Staudinger reduction^36,47^ without affecting the macrolide functionality represents a further advantage of using this protecting group at this specific position. Precursor **7** would be readily synthesized by Steglich esterification^48^ between thioglucoside **9** and dilipid **10**.

Inspired by the success story of the total synthesis of several macrolide-containing natural products^29,38,49–52^ and by way of comparison with the intramolecular glycosylation strategy, we also wanted to construct the 15-membered ring of ananatoside A (**1**) through both enzymatic and chemical macrolactonizations (Fig. 2). Regarding the chemical macrolactonization (route C), glucolipid **8** would act as an exquisite acyclic precursor for this intramolecular transformation following unmasking of the seco acid functionality. Moreover, global deprotection of this compound (**8**) would complete the total synthesis of ananatoside B (**2**). Glucolipid **8** would be prepared through the stereocontrolled glycosylation of thioglucoside **11** with benzylated dilipid **12**. Here again, the use of a neighboring participating group, *i.e.*, levulinoyl (Lev),^36,47,53^ that could be selectively cleaved in the presence of an ester functionality would insure a successful outcome for the final steps of the synthetic route. Finally, we hypothesized that ananatoside A (**1**) could be enzymatically synthesized from unprotected ananatoside B (**2**) using commercially available solid-supported lipase such as Novozyme 435 (route B).^41^ If successful, this enzymatic process would allow the direct and straightforward conversion of ananatoside B (**2**) into ananatoside A (**1**) from the isolated natural product or the synthetic compound.

#### Synthesis of building blocks

**Scheme 1:**
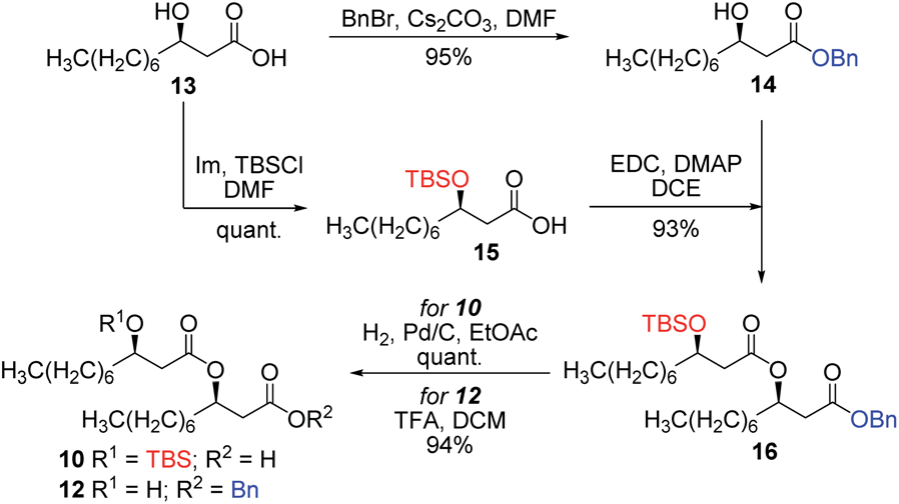
Synthesis of β-hydroxydecanoic acid derivatives.

Our synthetic journey commenced with the assembly of the dilipidic side chain derivatives. To do so, (*R*)-β-hydroxydecanoic acid **13** had to be synthesized first. According to the literature, monolipid **13** had previously been prepared *via* either a cross-metathesis/Mitsunobu sequence,^54^ the Reformatsky reaction^55^ or from Meldrum's acid.^56–59^ We decided to follow the latter approach as it allowed the straightforward formation of target monolipid **13** in high overall yield and enantiomeric purity. Therefore, as depicted in Scheme S1,† (*R*)-β-hydroxydecanoic acid **13** (ref. 57) was prepared in a four-step sequence. The configuration and enantiomeric purity (*R*, 99% ee) of methyl ester **S4** were confirmed through the preparation of Mosher's ester **S5** (ref. 56) and comparison with literature data (see Fig. S3†). Thereafter, β-hydroxydecanoic acid **13** was subjected to regioselective benzylation at the carboxylic acid moiety yielding benzyl ester **14** in a nearly quantitative yield (Scheme 1). In parallel, derivative **13** was protected with a TBS group at the C3 position affording silylated derivative **15**.^57^ These two compounds were condensed together under the activation of EDC/DMAP. Resulting protected dilipid derivative **16** was then either subjected to Pd-catalyzed hydrogenolysis of the benzyl ester or to TFA-mediated cleavage of the TBS group leading to the formation of acid **10** or alcohol **12**, respectively, ready for coupling with the carbohydrate derivatives. Optimization of this synthetic sequence allowed us to prepare gram amounts of these chiral lipidic intermediates.

**Scheme 2:**
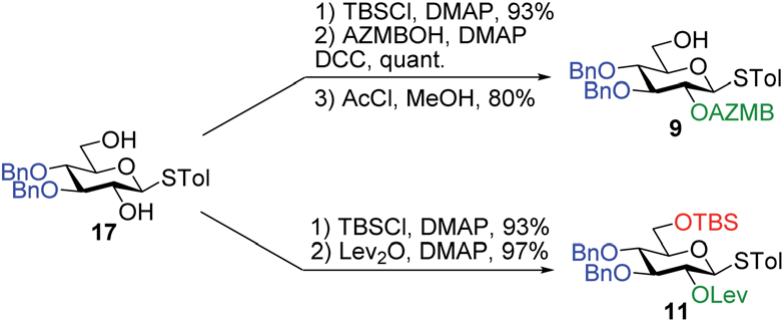
Synthesis of thioglucoside derivatives **9** and **11**.

Having completed the synthesis of the dilipid derivatives, we then focused our attention on the synthesis of thioglucosides **9** and **11**, which were either used for subsequent Steglich esterification or glycosylation reactions, respectively. These two compounds were prepared from diol **17**,^60^ which was obtained in a three-step, one-pot sequence from the corresponding pertrimethylsilylated thioglucoside. As revealed in Scheme 2, regioselective silylation at the C6 position of diol **17** followed by esterification with AZMBOH and subsequent desilylation under the action of *in situ* generated HCl led to derivative **9** bearing a free OH at C6 in 74% yield over three steps. Alternatively, a levulinoyl group was installed at C2 by treatment of the C6-*O*-TBS-protected derivative with levulinic anhydride to provide fully protected thioglucoside **11** in 90% yield over two steps.

#### Synthesis of ananatoside B

**Scheme 3:**
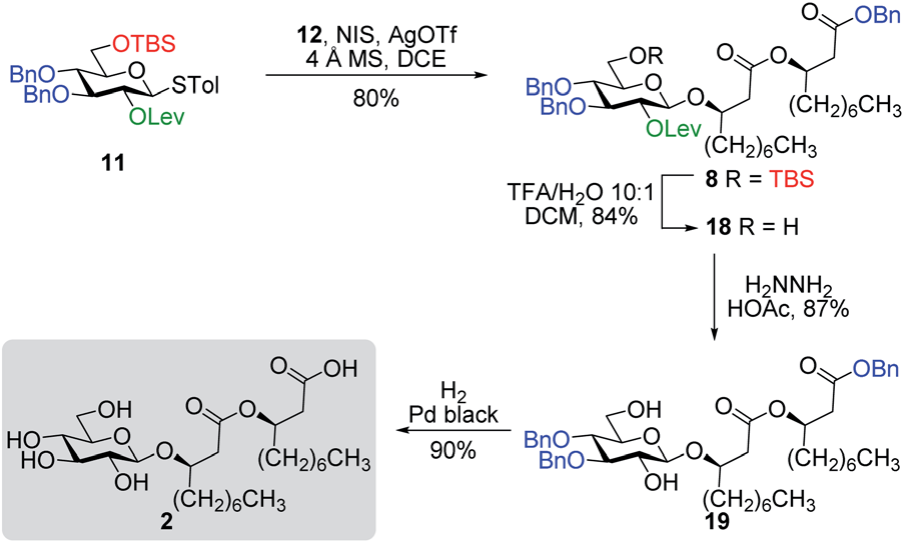
Total synthesis of ananatoside B.

Our next challenge was to proceed with the total synthesis of ananatoside B (**2**). Fully protected thioglucoside **11** was glycosylated with alcohol acceptor **12** under the promotion of NIS/AgOTf activating system,^61^ providing anomerically pure β-glucolipid **8** in 80% yield (Scheme 3). The stereoselectivity of the reaction was confirmed by ^1^H NMR (H1, d, ^3^*J*_H1,H2_ = 8.0 Hz for Glc*p* in the ^4^*C*_1_ conformation). Then, TFA-mediated cleavage of the TBS group, delevulinoylation under the action of hydrazine acetate, and Pd-catalyzed hydrogenolysis cleanly led to ananatoside B (**2**) in 66% yield over three steps. Physical and analytical data (*R*_f_, specific rotation, HRMS, and NMR) of synthetic ananatoside B (**2**) were in perfect agreement with the isolated natural product (see ESI† for details).

#### Synthesis of ananatoside A by intramolecular glycosylation

**Scheme 4:**
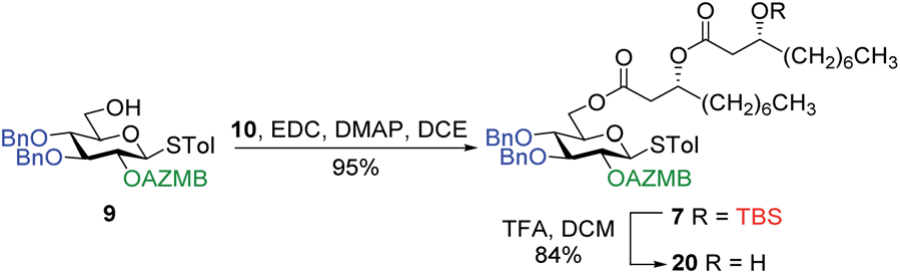
Synthesis of alcohol **20** ready for intramolecular glycosylation.

The total synthesis of ananatoside A (**1**) was our next task. We commenced by studying the intramolecular glycosylation pathway. To do this, we needed to prepare the acyclic precursor **20**. Dilipid **10** was condensed at the C6 position of thioglucoside **9** through activation with EDC/DMAP (Scheme 4). Resulting derivative **7** was treated with TFA in DCM to cleave the TBS group giving ω-hydroxy thioglucoside **20** in 84% yield ready for intramolecular glycosylation.

As shown in Table 1, different conditions were screened for the intramolecular glycosylation of acyclic precursor **20**, *i.e.*, promoter, molar volume, and additive. Initially, the concentration was set to 0.01 M (corresponding molar volume of 100 mL mmol^−1^) to minimize intermolecular interactions and thus preventing the formation of di- and oligomeric byproducts. At this concentration, AgOTf and TMSOTf were first evaluated as catalysts in combination with NIS (entries 1 and 2).^61^ In both conditions, target macrolide **21** was isolated in good yields with full β-stereoselectivity while no traces of diolide or higher oligomers were detected. Encouraged by these results, the impact of the concentration of precursor **20** on the reaction outcome was investigated by conducting the intramolecular glycosylation at 0.001 and 0.1 M (entries 3 and 4). We were pleased to find that macrolide **21** was formed quantitatively when the concentration of the acyclic precursor was set to 0.001 M. Moreover, even at a concentration of 0.1 M, which could have favored intermolecular interactions, only monomer **21** was isolated although the yield was significantly lower (47%). The use of excess amounts of TMSOTf (entry 5) or other triflates such as Yb(OTf)_3_ and Zn(OTf)_2_ (entries 6 and 7, respectively) also resulted in the exclusive formation of target macrolide **21**. Inspired by Fürstner and co-workers^50^ who completed the total synthesis of cycloviracin B_1_ through metal-templated macrolactonization, we then sought to evaluate the impact of an excess of metallic ions on the outcome of the glycosylation and to observe if oligomeric macrolides could be generated. As it is known that potassium and sodium are effective ions for such templated reactions,^50^ KOTf and NaOTf were employed as additives in the presence of catalytic amounts of TMSOTf (entries 8–10). Once again, the intramolecular reaction was highly favored as no other products than macrolide **21** were isolated from the reaction mixture.

**Table 1:** NIS/triflate-promoted intramolecular glycosylation of precursor **20**

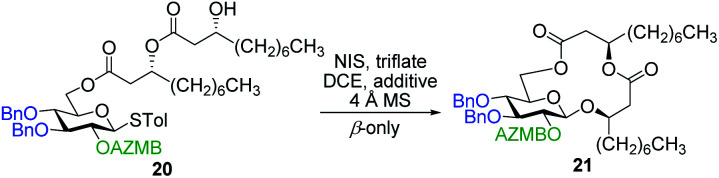				
Entry	Triflate (equiv.)	Molar volume (mL mmol^−1^)	Additive	Yield^a^ (%)
1	AgOTf (0.4)	100	—	70
2	TMSOTf (0.2)	100	—	86
3	TMSOTf (0.2)	1000	—	Quant.
4	TMSOTf (0.2)	10	—	47
5	TMSOTf (2.1)	100	—	85
6	Yb(OTf)_3_ (1.6)	100	—	21
7	Zn(OTf)_2_ (1.6)	100	—	75
8	TMSOTf (0.2)	100	NaOTf	78
9	TMSOTf (0.2)	100	KOTf	63
10	TMSOTf (0.2)	50	KOTf	61

a Isolated yield.

#### Synthesis of ananatoside A by chemical macrolactonization

As previously mentioned, the second pathway that we chose to investigate for the preparation of ananatoside A (**1**) was based on a chemical macrolactonization^49^ as the key step. As depicted in Table 2, glucolipid derivative **18** was used as an advanced intermediate for this approach. The benzyl ester moiety of the latter compound was thus selectively cleaved by catalytic hydrogenation transfer, using cyclohexadiene as the hydrogen transfer source.^62,63^ Intramolecular cyclization of the resulting seco acid **22** was then studied through Keck (DCC, DMAP, PPTs)^64^ and Fujisawa [2-chloro-1,3-dimethylimidazolinium chloride (DMC), DMAP]^65^ macrolactonizations. These intramolecular esterifications were performed at different concentrations and temperatures, with and without the slow addition of seco acid **22**, and by using two different coupling systems (Table 2, entries 1–4). Pleasingly, target macrolide **23** was formed in 71% yield when using DMC-mediated macrolactonization under concentrated conditions (entry 4) and, as for the intramolecular glycosylation, only monomer **23** was detected. Cs_2_CO_3_ or KOTf were then added in the reaction mixture as to favor oligomerization but only traces of dimer were detected by HRMS (entries 5–7). Interestingly, it was possible to improve the yield up to 80% for the formation of target macrolide **23** when KOTf was used as an additive (entry 6).

**Table 2:** Chemical macrolactonization of precursor **22**

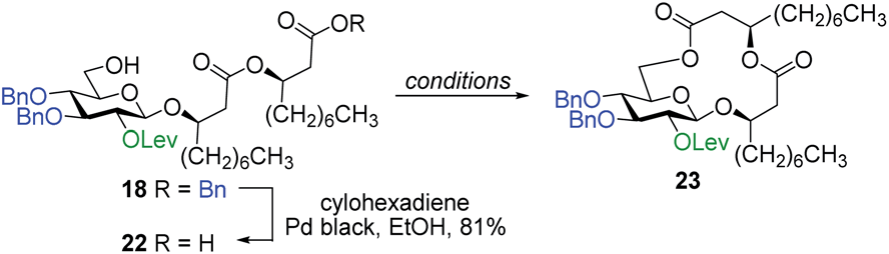			
Entry	Reagents	Molar volume (mL mmol^−1^)	Yield^a^ (%)
1	DCC, DMAP, PPTs	860	64
2	DCC, DMAP, PPTs	60	40
3	DMC, DMAP	60	67
4	DMC, DMAP	15	71
5	DMC, DMAP, Cs_2_CO_3_	60	47^b^
6	DMC, DMAP, KOTf	60	80^b^
7	DMC, DMAP, KOTf	15	73

a Isolated yield.

b Traces of dimer were detected.

**Scheme 5:**
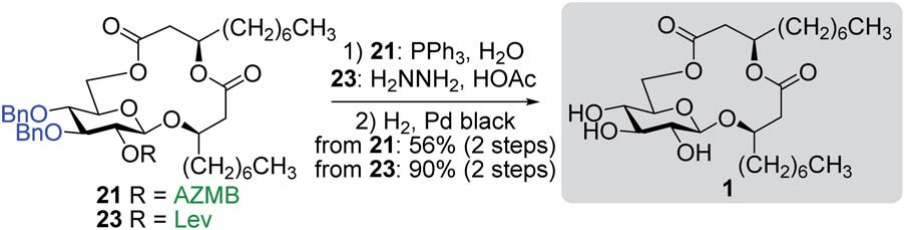
Deprotection of macrolides **21** and **23** into ananatoside A (**1**).

With protected macrolides **21** and **23** in hand, we were able to complete the total synthesis of ananatoside A (**1**) (Scheme 5). Therefore, the AZMB group of macrolide **21** and the Lev group of macrolide **23** were orthogonally removed through Staudinger reduction or treatment with hydrazine acetate, respectively. Pd-catalyzed hydrogenolysis of the resulting alcohols cleanly led to the formation of ananatoside A (**1**) in 56 or 90% yield over two steps from macrolide **21** or **23**, respectively. Physical and analytical data (*R*_f_, specific rotation, HRMS, and NMR) of synthetic ananatoside A (**1**) were in perfect agreement with the isolated natural product^26^ (see ESI† for details). The overall yields and number of steps for both synthetic sequences were very similar, *i.e.*, 33 and 35% over eight steps from known diol **17** for the intramolecular glycosylation and macrolactonization pathways, respectively.

#### Synthesis of ananatoside A by enzymatic macrolactonization

We were also interested in the direct conversion of ananatoside B (**2**) into ananatoside A (**1**) by taking advantage of an enzyme-catalyzed macrolactonization. Although efficient in terms of overall yields, both previously described synthetic approaches required protecting groups manipulation. Contrarywise, enzymatic reactions offer the advantages of being renewable and specific, and of minimizing side product formation and purification steps.^41^ Immobilized *Candida antarctica* lipase B (CALB), commercially available under the brand name of “Novozyme 435”, was therefore selected for this synthetic study as it has showed high substrate versatility and efficiency for macrolactonization reactions.^39^ As revealed in Table 3, the reaction was conducted in the presence of molecular sieves (4 Å) as to insure strictly anhydrous conditions throughout the enzymatic process. As hydrophobic solvents favor such type of enzymatic reactions,^39^ toluene was first employed at 75 °C (entry 1). However, after a seven-day period, only 30% of conversion was observed (as estimated by ^1^H NMR). When switching to hexanes, an even lower conversion was obtained (entry 2), and only traces of ananatoside A (**1**) were detected when the more polar THF was employed as the reaction medium (entry 3). We were pleased to find that switching back to toluene and conducting this enzyme-catalyzed reaction under microwave irradiations for three hours at 75 °C allowed the isolation of ananatoside A (**1**) in a satisfying 64% yield. To the best of our knowledge, this is the first report of using CALB for the enzymatic conversion of a monoglycolipid into a macrolide.

**Table 3:** Enzymatic macrolactonization of ananatoside B (**2**) into ananatoside A (**1**)

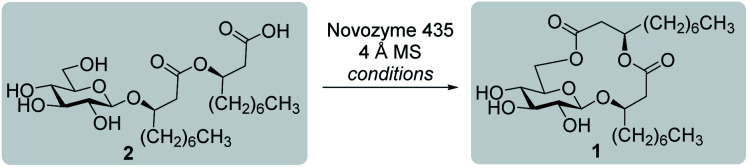				
Entry	Solvent	Temp. (°C)	Time (h)	Yield (%)
1	Toluene	75	168	30^a^
2	Hexanes^b^	55	114	10^a^
3	THF^b^	55	112	Traces
4	Toluene^b^	75^c^	3	64^d^

a Conversion (estimated by NMR).

b Reaction in sealed tube.

c Microwave heating.

d Isolated yield.

### Synthesis of RhaC_10_C_10_ (**3**) and macrolactonized rhamnolipids **4–6**

#### Retrosynthetic analysis

**Fig. 3:**
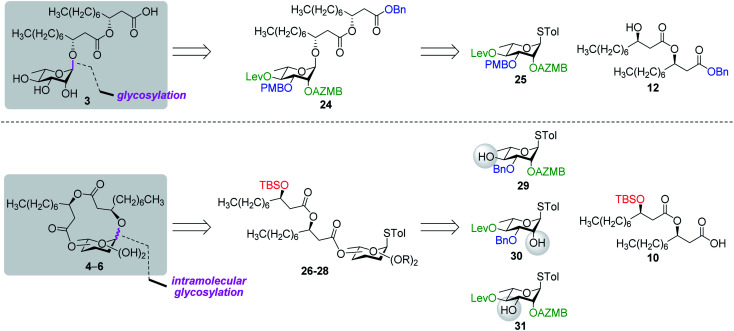
Retrosynthetic analysis of rhamnolipid 3 and macrodilactone-containing rhamnolipids **4–6**. PMB: *para*-methoxybenzyl. Blue: permanent protecting groups (Bn and PMB); red: temporary protecting groups (TBS); green: esters used as temporary protecting groups enabling neighboring group participation when branched at C2 (AZMB and Lev).

The preparation of microbial rhamnolipids has only been reported in few occasions in the literature.^55,56,58,59,66,67^ We thought it would be worth reinvestigating the synthesis of RhaC_10_C_10_ (**3**) as to obtain a pure synthetic sample that could be evaluated for its physical properties and biological activity in direct comparison with ananatosides. Furthermore, using an orthogonally protected donor such as thiorhamnoside **25** in which each hydroxyl groups (C2, C3, and C4) could be selectively unmasked (Fig. 3), we hypothesized that a series of unprecedented macrolactonized rhamnolipids (**4–6**) could be produced *via* an intramolecular glycosylation strategy. As a parallel to ananatoside A (**1**) and B (**2**) acid/lactone pair, these macrolides would serve as model compounds to study the impact of macrolactonization on the biological activity of rhamnolipids. Therefore, as depicted in Fig. 3, rhamnolipid **3** would be obtained from the intermolecular glycosylation of rhamnoside **25** with dilipid **12** followed by global deprotection. As for macrolactones **4–6**, they would become accessible *via* the intramolecular glycosylation of ester-linked rhamnolipid acyclic precursors **26–38**, which would be formed by Steglich condensation of dilipid **10** with their corresponding unmasked alcohols (**29**, **30**, and **31**). We anticipated that the presence of a macrolactone functionality in target rhamnolipids **4–6** would induce substantial conformational changes of the rhamnopyranose ring, which is typically found in the ^1^*C*_4_ conformation, hence making non-trivial the determination of anomeric configurations in resulting macrolides. As such, DFT calculations would be used as a theorical tool in conjunction with NMR spectroscopy to determine the exact anomeric configurations and ring conformations of rhamnolactones **4–6**.

#### Synthesis of building blocks

**Scheme 6:**
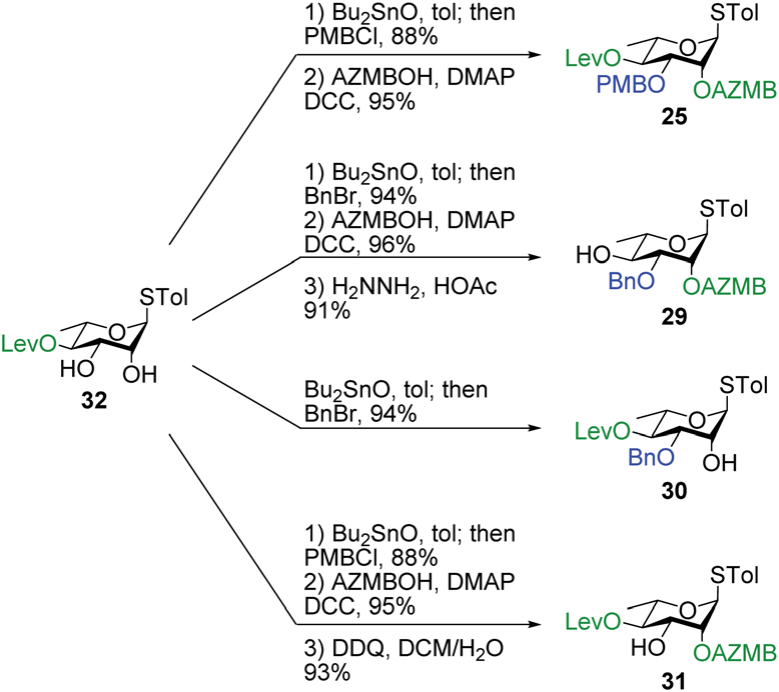
Synthesis of thiorhamnoside building blocks **25**, **29**, **30**, and **31**.

The synthesis of the thiorhamnoside building blocks was straightforward (Scheme 6). Our approach mainly relied on the stannylene acetal-mediated^68^ regioselective introduction of either PMB or Bn groups at C3 from known diol **32**.^36^ Then, installation of the AZMB group at C2 followed by orthogonal cleavage at either C3 or C4 positions provided functionalized thiorhamnosides **25**, **29**, **30**, and **31**. These optimized procedures allowed us to prepare gram amounts of each building blocks in pure anomeric forms.

#### Synthesis of rhamnolipid C_10_C_10_

**Scheme 7:**
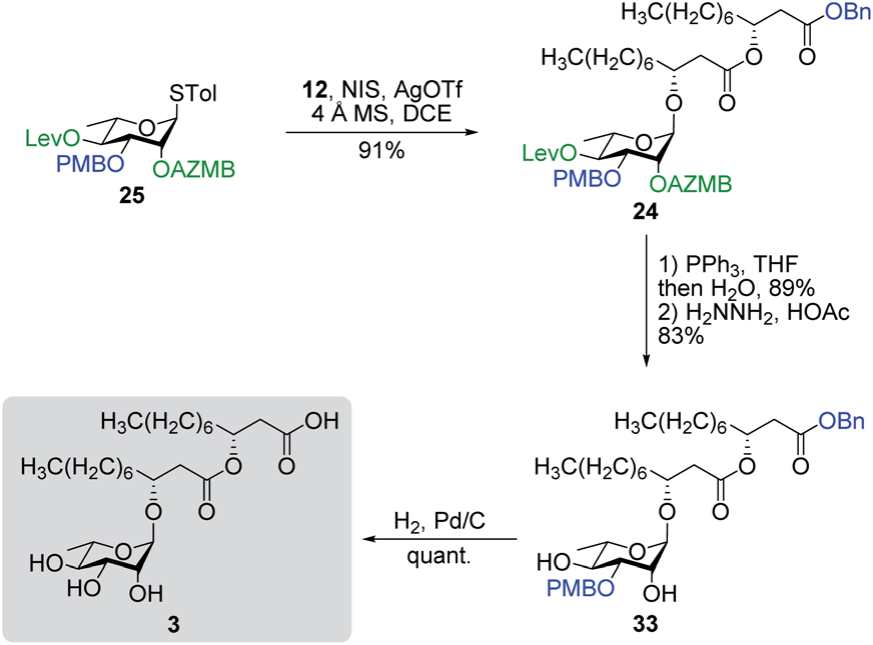
Total synthesis of RhaC_10_C_10_ (**3**).

With our newly developed fully functionalized thiorhamnoside **25** in hand, we were ready to conduct the synthesis of RhaC_10_C_10_ (**3**).^59^ As depicted in Scheme 7, coupling of rhamnosyl donor **25** with dilipid acceptor **12** under the promotion of NIS/AgOTf cleanly provided protected rhamnolipid **24** in 91% yield with full control of 1,2-*trans* stereoselectivity owing to the neighboring group participation of the AZMB group at C2. Then, global deprotection of derivative **24** was accomplished uneventfully following a three-step sequence including Staudinger reduction, chemoselective cleavage of the Lev ester, and Pd-catalyzed hydrogenolysis of both benzyl ester and PMB ether functionalities. The overall yield for the synthesis of RhaC_10_C_10_ (**3**) was 56% over six steps starting from diol **32**. We have also completed the total synthesis of rhamnolipid **3** from thiorhamnoside **S6** (ref. 69) bearing the commonly used 3,4-*O*-butanediacetal^56,67^ as a diol protecting group (see Scheme S2†). The latter route was less efficient in terms of overall yield as compared to the former one.

#### Macrolactonization of rhamnolipids

**Scheme 8:**
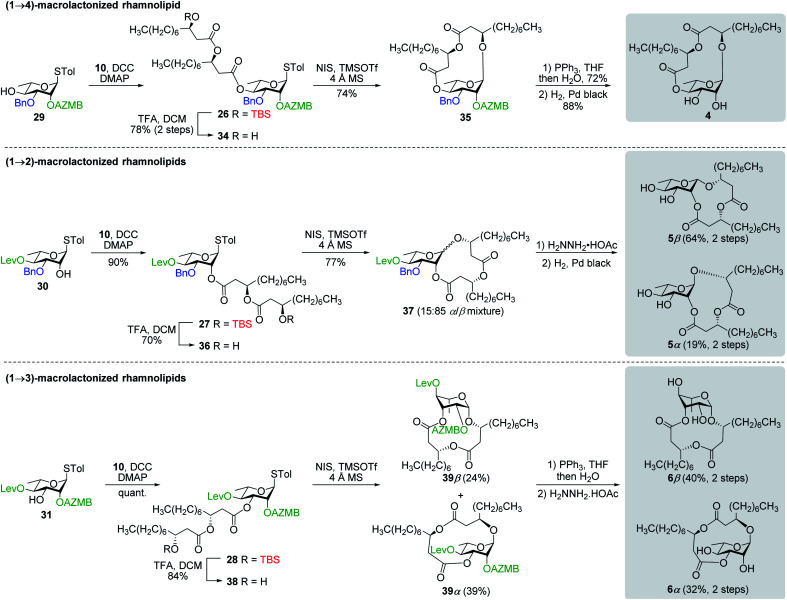
Synthesis of (1→4), (1→2), and (1→3)-macrolactonized rhamnolipids **4–6**.

Our last synthetic challenge consisted in the preparation of (1→2)-, (1→3)- and (1→4)-macrolactonized rhamnolipids (**4–6**). These three types of macrolactones were obtained following a similar synthetic route (Scheme 8). Our approach entailed esterification of thiorhamnosides **29**, **30**, or **31** with dilipid **10** by means of DCC/DMAP coupling reactions to afford derivatives **26**, **27**, and **28**, respectively, in excellent yields. Subsequent chemoselective cleavage of the C3′′ TBS group in the presence of TFA led to acyclic precursors **34**, **36**, and **38**, respectively, which were ready to be macrolactonized. Intramolecular glycosylation of precursor **34** under the activation of NIS/TMSOTf furnished protected (1→4)-macrolide **35** in 74% yield with full control of *α*-selectivity. Then, Staudinger reduction followed by Pd black-mediated hydrogenolysis provided target (1→4)-macrolide **4**. Because of the presence of the dilipid chain at C2 imposing steric constraints on the pyranose conformation, intramolecular glycosylation of precursor **36** under previously mentioned conditions led to the formation of an inseparable α/β anomeric mixture of protected (1→2)-macrolide **37** with the β-anomer found as the major compound (α/β = 15 : 85). Following cleavage of both the Lev and Bn groups, α- and β-(1→2)-macrolides, *i.e.*, compounds **5α** and **5β**, respectively, were isolated in pure forms in a combined yield of 83%. Intramolecular glycosylation of precursor **38** also generated a mixture of anomers although this time they were easily separable by silica gel chromatography under their protected forms (**39β**, 24%, β-anomer; and **39α**, 39%, α-anomer). Orthogonal cleavage of both the AZMB and Lev groups completed the total synthesis of target (1→3)-macrolide **6** in pure α- and β-anomeric forms. The determination of the anomeric configurations in macrolides **4**, **5β**, **5α**, **6β**, and **6α** was accomplished by NMR with the help of DFT calculations as described in the next section.

### Molecular modeling of macrolactonized rhamnolipids

**Fig. 4:**
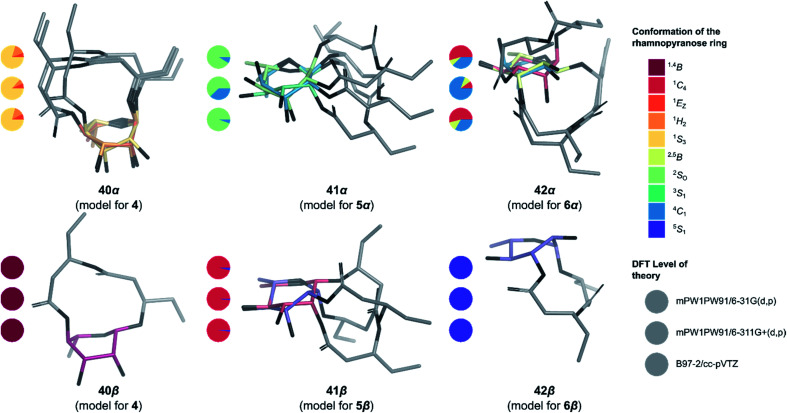
3D Structure of the macrolactonized rhamnolipids models **40–42**. Only the most stable structure of each conformer is depicted. Pie chart gives Boltzmann population for standard six-membered ring^1^ for the three levels of theory used in this study (from top to bottom: mPW1PW91/6-31G(d,p), mPW1PW91/6-311+G(d,p) and B97-2/pVTZ).

To determine the anomeric configuration of a monosaccharide, the ^3^*J*_H1,H2_ coupling constant is commonly measured by ^1^H NMR.^70^ In the case of rhamnopyranosides, the *J* values for the two anomers are too low to be a suitable selection criterion. Therefore, the δ_C_ at C5 (70.0 ppm for α-Rha*p* or 73.2 ppm for β-Rha*p*)^71^ or the ^1^*J*_C1,H1_ coupling constant (167.2–172.3 Hz for α-Rha*p* or 152.3–159.8 Hz for β-Rha*p*) are preferred for rhamnopyranosides.^72^ These methods are valid only because rhamnopyranosides usually adopt a typical ^1^*C*_4_ conformation. However, in the case of macrolactonized rhamnolipids **4–6**, other conformations are likely to appear due to the steric constraints imposed by the bicyclic nature of these compounds, rendering useless these empirical rules for identifying the proper anomer. To overcome this problem, *in silico* models of synthetic rhamnolipids were prepared with truncated alkyl chains as to minimize the number of conformers to be considered (Fig. S4†). The conformational space of these models was explored in depth (Fig. S5†) using the improved RDKit algorithm, which is based on the geometry of distances and empirical preferences for certain angles of torsion (ETKDGv2).^73^ The geometry of the unique conformers was optimized by molecular mechanics (MMFF94) followed by quantum molecular modeling using density functional theory (mPW1PW91) and the basis set 6-31G(d, p). The thermochemical parameters were calculated to deduce the abundance of conformers in solution using the Boltzmann equation, thus eliminating marginal conformers (<1%). The retained conformers were inspected so that no imaginary vibrational frequency could be found. Examination of the sugar conformation of the conserved structures confirmed the irregular nature of these glycosides. Indeed, three different skew boat (^1^*S*_3_ for **4**, ^2^*S*_0_ for **5α**, and ^5^*S*_1_ for **6β**) along with two chair conformations (^4^*C*_1_ for **6α** and ^1^*C*_4_ for **5β**) were found for the main conformers of the modelized compounds (Fig. 4). The shielding tensors were calculated with the same level of theory and then converted to chemical shifts using the multiple reference method.^74,75^ First, the means of the absolute values of the errors (MAE) were calculated between each synthetic molecules **4–6** and the corresponding *in silico* anomer pairs **40–42** (Table S1†). In most cases, it was possible to assign the α- and β-anomers to a single model, but the case of compound **5β** was found to be ambiguous at the B97-2/pVTZ level of theory. Indeed, when only the ^1^H NMR data were considered, we found that **5α** and **5β** would both be of α-configuration. To ensure the validity of these assignments, the pooled comparison method proposed by Lauro *et al.*^76^ was performed for the **5α**/**5β** and **6α**/**6β** anomeric pairs (Table S1†). In this way, the anomeric configuration of each synthetic macrolactone was unambiguously assigned as depicted in Scheme 8.

### Surfactant properties of natural glycolipids

Biosurfactants are molecules that can associate with each other leading to the formation of micelles. They can also interact with surfaces between phases of different polarity such as the air/water or oil/water interface.^77^ To gain quantitative insights into the surface-active properties of microbial glycolipids, the surface tensions at CMC (*γ*_CMC_) of ananatoside A (**1**) and ananatoside B (**2**) were measured using a Fisher tensiometer by means of the du Noüy ring method. The CMC, referring to the concentration of surfactant at which micelles start to form, was determined from a plot of surface tension again each concentration. These data were compared to those obtained for synthetic RhaC_10_C_10_ (**3**). Due to the presence of a free carboxylic acid moiety in biosurfactants **2** and **3**, the surface tensions were measured at neutral pH, which corresponds to the presence of their anionic forms in solution based on an estimated p*K*_a_ of 5.5 for RhaC_10_C_10_ (**3**).^78^ As shown in Table 4, the CMC value for ananatoside B (**2**) was similar to the one measured for rhamnolipid **3**, denoting that ananatoside B (**2**) can be considered a potent biosurfactant. In the case of ananatoside A (**1**), the measured CMC value was even lower than for RhaC_10_C_10_ (**3**). However, the latter value must be taken with cautious as the presence of the macrolactone functionality decreased considerably the water solubility of ananatoside A (**1**) as compared to its open-chain congener. Consequently, the surface tension measurements were performed quickly to avoid the precipitation of ananatoside A (**1**) into the aqueous medium. It is interesting to note that the surface tension values measured for biosurfactants **2** and **3** were on the same order of magnitude than those measured by Pemberton and co-workers^59^ for structurally similar synthetic rhamnolipids bearing C_10_C_10_ lipid chains.

**Table 4:** Critical micelle concentration (CMC), surface tension (*γ*_CMC_), and hydrodynamic diameter (*Z*-average) of naturally occurring biosurfactants **1–3**

Glycolipid^a^	CMC^b^ (μM)	*γ*_CMC_ b (nM m^−1^)	*Z*-Average^b^^*,*^^c^ (nm)
**1**	43 ± 2	37 ± 1	>800^d^
**2**	63 ± 2	28 ± 1	99.0
**3**	58 ± 1	30 ± 1	92.1

a Synthetic samples.

b Data taken at pH 7.0.

c Hydrodynamic diameter of micelles measured with a Zetasizer instrument at concentrations of 80 μM for ananatoside B (**2**) and RhaC_10_C_10_ (**3**), and 100 μM for ananatoside A (**1**).

d Size distribution showed the formation of aggregated particles.

In parallel to these experiments, we took advantage of dynamic light scattering (DLS) to evaluate the hydrodynamic diameter of biosurfactant micelles formed at concentrations above the CMC (Table 4).^79^ DLS considers the intensity of light scattered by spherical particles, which prevents its application for highly polydisperse and non-spherical colloidal systems. Using a Zetasizer instrument, the results showed that the *Z*-average, *i.e.*, the mean diameter of the micelles, was in the same range for ananatoside B (**2**) and for rhamnolipid **3** (see Fig. S6†). Once again, due to its hydrophobicity, ananatoside A (**1**) was too polydisperse for conducting a proper distribution analysis, showing a Z-average value above 800 nm, which meant that the particles were aggregated in water at the tested concentration. Similar results were obtained for ananatoside A (**1**) when the DLS experiments were conducted in DMSO as the solvent. These results showed that ananatoside B (**2**), similarly to rhamnolipid **3**, formed monodisperse colloidal particles at concentrations above its CMC.

The propensity of surfactants to stabilize emulsions shows promising for biotechnological applications such as bioremediation of contaminated soils and water.^77^ Therefore, the emulsifying properties of microbial biosurfactants **1–3** were evaluated against light as well as heavy hydrocarbon contaminants such as cyclohexane and kerosene, respectively (see Fig. S7†). The emulsifying activity results showed that ananatoside B (**2**) and RhaC_10_C_10_ (**3**) were equally able to generate emulsions with cyclohexane and kerosene while ananatoside A (**1**) was unable to form any emulsion, mainly because of its lack of affinity for water. Altogether, these results once again highlighted that ananatoside B (**2**), but not its macrolide counterpart **1**, exhibits potent surfactant properties that could be advantageously used for biotechnological applications.

### Antimicrobial activity, cytotoxicity, and hemolytic activity of natural and synthetic glycolipids

As previously mentioned in the introduction section, numerous studies have reported the antimicrobial potential of rhamnolipids against several pathogenic bacteria and fungi.^12^ However, as rhamnolipids were usually tested as mixtures of different congeners and not as pure synthetic compounds, there seems to be no clear consensus regarding the antimicrobial activity of one specific congener. Hence, as an initial investigation toward their biological functions in bacteria, the *in vitro* antimicrobial activity of ananatoside A (**1**), ananatoside B (**2**), RhaC_10_C_10_ (**3**), and macrolides **4–6** was evaluated against Gram-negative, *i.e.*, *Pseudomonas aeruginosa* PA14, *Pseudomonas aeruginosa* LESB58, and *Escherichia coli* DH5α, and Gram-positive bacteria, *i.e.*, *Staphylococcus aureus* MRSA, *Staphylococcus aureus* Newman, and *Bacillus subtilis* PY79, as well as against fungi, *i.e.*, *Candida albicans* ATCC 10231 and *Candida albicans* LSPQ 0199. At the maximum tested concentration (37.5 μg mL^−1^), glycolipids **1–6** were unable to inhibit the growth of these microbes. These results suggest that the biosynthetic production of ananatoside A (**1**) and ananatoside B (**2**) by *P. ananatis* is not aimed for antimicrobial purposes.

As the anticancer potential of microbial rhamnolipids has previously been reported,^16^ we next sought to evaluate the *in vitro* cytotoxicity of our synthetic and natural glycolipids **1–6** against cancerous cell lines, *i.e.*, human lung carcinoma (A549) and colorectal adenocarcinoma (DLD-1). The compounds were also tested against human normal skin fibroblasts (WS1) to estimate their selectivity towards cancer cells. As revealed in Table 5, very interesting conclusions can be derived from these cytotoxicity results. First, it appears that only macrolactones, as compared to their open forms, were able to inhibit the growth of human cell lines. Second, the cytotoxic activity of the active macrolides was not specific to cancer cell lines as comparable IC_50_ values were measured against healthy cells (WS1), pointing toward a general mechanism involving the formation of pores in cell membrane.^16^ Specifically, ananatoside A (**1**) was cytotoxic against human cell lines (IC_50_ = 50−58 μM) while its open form counterpart ananatoside B (**2**) was inactive at the maximum tested concentration (IC_50_ > 200 μM). Furthermore, macrolactonization of the inactive RhaC_10_C_10_ (**3**, IC_50_ > 200 μM) generated cytotoxic macrolides **4**, **5β**, and **6β** (IC_50_ = 50–112 μM). While (1→4)-macrolactonized rhamnolipid **4** was the most active congener, only the β-anomers of (1→2)- and (1→3)-macrolactonized rhamnolipid **5β** and **6β**, respectively, were found to inhibit the growth of human cell lines. These results showed that both the position of the macrolactone ring and the anomeric configuration of the glycosidic bonds can impact the cytotoxicity of the rhamnolipids.

**Table 5:** Cytotoxicity and hemolytic activity of natural and synthetic glycolipids (**1–6**)

Glycolipid^a^	Cytotoxicity			Hemolysis
	(IC_50_ in μM)^b^			(HC_50_ in μM)^c^
	A549	DLD-1	WS1	Erythrocytes
**1**	50 ± 7	58 ± 2	52 ± 4	12.3 ± 0.3
**2**	>200^d^	>200^d^	>200^d^	>200^d^
**3**	>200^d^	>200^d^	>200^d^	65 ± 6
**4**	56 ± 2	70 ± 10	57 ± 1	12.0 ± 0.7
**5β**	103 ± 7	110 ± 2	105 ± 5	14 ± 2
**5α**	>200^d^	>200^d^	>200^d^	>200^d^
**6β**	112 ± 2	112 ± 3	110 ± 1	12.3 ± 0.5
**6α**	>200^d^	>200^d^	>200^d^	>200^d^

a Synthetic samples.

b Half maximal inhibitory concentration measured *via* the resazurin assay. Etoposide was used as a positive control^80^ showing IC_50_ values of 1.2, 27, and 34 μM against A549, DLD-1, and WS1 cell lines, respectively.

c Half maximal inhibitory concentration measured on sheep blood erythrocytes. Triton X-100 was used as a positive control showing an HC_50_ value of 52 ± 2 μM.

d No inhibition or activity at the maximum tested concentration (IC_50_ or HC_50_ > 200 μM).

Owing to their surfactant properties, glycolipids can break the membrane of red blood cells causing hemolytic activity.^18^ To understand if the cytotoxicity of macrolactones **1**, **4**, **5β**, and **6β** was due to their surface tension activity, we evaluated the hemolytic potential of synthetic glycolipids **1–6** against sheep erythrocytes (Table 5). As anticipated, all the cytotoxic glycolipids were shown to exhibit hemolytic activities on red blood cells with HC_50_ ranging from 12 to 14 μM. The non-cytotoxic rhamnolipid **3** was also found to exert a weak hemolytic activity (HC_50_ = 65 ± 6 μM). Altogether, these data suggest a non-specific mechanism of cytotoxicity involving the intercalation of macrolactones **1**, **4**, **5β**, and **6β** into the lipid constituents of the cell membranes.

### Interaction of natural and synthetic glycolipids with the plant immune system

**Fig. 5:**
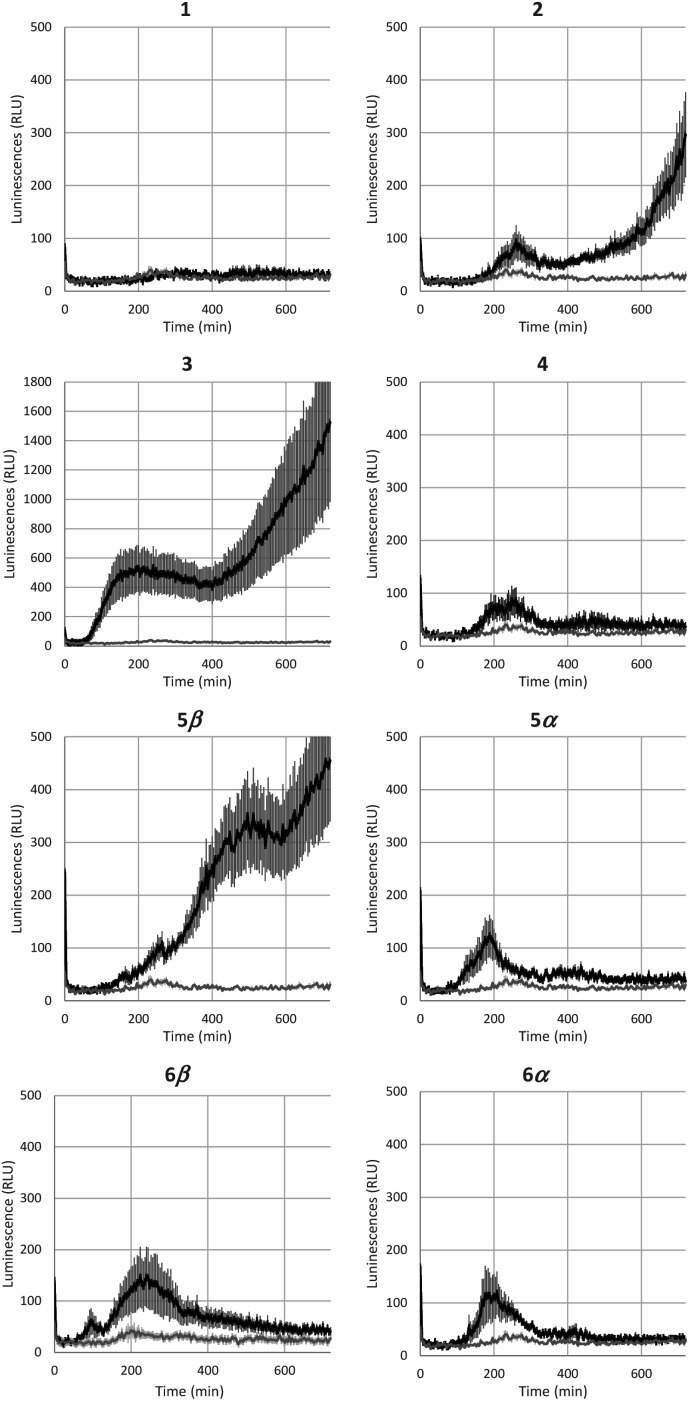
Extracellular reactive oxygen species (ROS) production following treatment of tomato leaf disks with ananatoside A (**1**), ananatoside B (**2**), RhaC_10_C_10_ (**3**), and related macrodilactone-containing rhamnolipids (**4–6**). Production of ROS was measured in tomato leaf disks following treatment at 100 μM with synthetic glycolipids (**1–6**). MeOH (0.5%) was used as a control. ROS production was measured using the chemiluminescence of luminol and photon counts were expressed as relative luminescence units (RLUs). Data are mean ± SEM (*n* = 6). Experiments were independently realized three times with similar results.

Bacterial rhamnolipids, such as compound **3**, trigger the plant immune system, which can ultimately lead to plant protection against diseases caused by fungal and bacterial pathogens.^23^ As ananatoside A (**1**) and ananatoside B (**2**) share structural similarities with rhamnolipid **3** and because they have been isolated from *P. ananatis* cultures, an emerging plant pathogen causing important agricultural damage worldwide,^81^ we wanted to investigate whether these bacterial glycolipids, along with our unprecedented series of synthetic rhamnolactones, could be perceived by the plant immune system. We relied on measuring the extracellular content of reactive oxygen species (ROS) produced by tomato plants (*Solanum lycopersicum*) following treatment with the synthetic compounds, as an early and important marker of plant immunity.^20,22,82^ Tomato leaves were challenged with synthetic glycolipids **1–6** at concentrations of 100 μM and the production of ROS was monitored over a 720 min time frame (Fig. 5). As expected, rhamnolipid **3**, used as a positive control in this study, triggered a long-lasting and strong production of ROS in tomato leaves.

Although less strong in amplitude, a similar pattern of ROS production was measured for ananatoside B (**2**), but not for ananatoside A (**1**), its macrolactonic counterpart. These responses seem to be however plant-specific because, when tested in *Arabidopsis thaliana*, both natural glycolipids **1** and **2** were perceived by plants (see Fig. S8†). As for rhamnolactones **4–6**, all of them were sensed by tomatoes: the luminescence curves showed a small burst at 200 min followed by a return to basal levels over the next 200 min. The only exception to this general trend was compound **5β**. Indeed (1→2)-macrolactonized rhamnolipid **5β** induced a strong and long-lasting production of ROS following the initial burst at 200 min. This response was stereospecific as corresponding α-anomer **5α** did not show a similar sensing pattern. Interestingly, our molecular modeling showed that macrolide **5β** was the only one to be found in the native ^1^*C*_4_ conformation for the rhamnose ring (see Fig. 4), which could be part of the explanation for these intriguing results. Taken together, these results highlight that (1), glycolipids sharing the same 3-hydroxyalkanoate dilipidic chain than rhamnolipids but bearing a glucose instead of a rhamnose moiety can be perceived by plants; and (2) macrolactonization of these glycolipids alter the sensing patterns according to the plant species as well as the conformation of the rhamnopyranose ring.

## Conclusions

In summary, we have isolated and structurally characterized rhamnolipid-like ananatoside A (**1**) and ananatoside B (**2**) from an organic extract of *P. ananatis*, a non-human pathogenic producer of biosurfactants. We have accomplished, for the first time, the total synthesis of ananatoside A (**1**) and ananatoside B (**2**), confirming the structure of these bacterial glucolipids. The macrodilactone-containing ananatoside A (**1**) was efficiently synthesized according to three alternative pathways: (1) *via* the stereoselective intramolecular glycosylation of a thioglucoside donor; (2) *via* the chemical macrolactonization of a seco acid precursor; and (3) *via* the direct enzymatic macrolactonization of its open form congener ananatoside B (**2**) using a solid-supported lipase. Capitalizing on the expeditious intramolecular glycosylation of orthogonally protected thiorhamnoside donors, we have accomplished the synthesis of diastereoisomerically pure (1→2), (1→3), and (1→4)-macrolactonized rhamnolipids. We have determined the anomeric configuration of these unprecedented macrolides through molecular modeling and found that the rhamnose ring adopts unusual conformations including skew boat and flipped chair conformations. Determination of the surfactant properties of bacterial glucolipids **1** and **2** in comparison with rhamnolipid **3** has revealed that ananatoside B (**2**), but not its macrolide counterpart **1**, represents a potent biosurfactant, as shown by its efficient emulsifying activity. Furthermore, we have shown that synthetic glycolipids **1–6** do not exhibit any significant antimicrobial activity against Gram-negative and Gram-positive bacteria as well as against fungi. We have also highlighted that the presence of the macrodilactone functionality can convert non-cytotoxic glycolipids into cytotoxic ones according to the position and anomeric configuration of the macrolides. We have revealed a direct correlation between the cytotoxicity and the hemolytic activity of these macrolides pointing towards a mechanism involving the formation of pores into the lipidic cell membrane. Finally, we have demonstrated that natural glucolipids **1** and **2** as well as unnatural macrolides **4–6** can be perceived by the plant immune system, and that this sensing was long-lasting for macrolide **5β** featuring a rhamnose moiety in its native ^1^*C*_4_ conformation. Altogether our results suggest that macrolactonization of glycolipids can dramatically interfere with their surfactant properties and biological activity. Our interdisciplinary study could serve as a foundation for the rational design of rhamnolipid-like biosurfactants with improved properties for biotechnological and/or therapeutic applications.

## Author contributions

M. C. and M.-J. P. synthesized the compounds. M. P., S. L., and C. G. performed the isolation and structural elucidation of ananatosides. S. L. and T. F. conducted the DFT calculations. M.-C. G. performed the antimicrobial and hemolytic assays. J. L. performed the cytotoxicity assays. S. V., J. C., and S. D. assessed the compounds against the plant immune system. M. A. D. D. R. conducted the surfactant assays. All authors analysed and discussed the results. M. C., S. L., M. P., E. D., and C. G. wrote the manuscript. E. D and C. G. secured funding for this work.

## Conflicts of interest

There are no conflicts to declare.

## Supplementary Material

SC-012-D1SC01146D-s001
