# Supplementary material for: Total synthesis, isolation, surfactant properties, and biological evaluation of ananatosides and related macrodilactone-containing rhamnolipids
Source: Chem Sci. 2021 May 4;12(21):7533–46. doi: 10.1039/d1sc01146d (PMC8171317; doi:10.1039/d1sc01146d)
Supplement: SC-012-D1SC01146D-s001 [file SC-012-D1SC01146D-s001.pdf]

Electronic Supplementary Material (ESI) for Chemical Science.
This journal is © The Royal Society of Chemistry 2021

SUPPLEMENTARY INFORMATION

Total Synthesis, Isolation, Surfactant Properties, and Biological Evaluation of Ananatosides and Related Macrodilactone-Containing Rhamnolipids

Maude Cloutier,[1] Marie-Joëlle Prévost,[1] Serge Lavoie,[2] Thomas Feroldi,[2] Marianne Piochon,[1] Marie-Christine Groleau,[1] Jean Legault,[2] Sandra Villaume,[3] Jérôme Crouzet,[3] Stéphan Dorey,[3] Mayri Alejandra Dìaz De Rienzo,[1,4] Eric Déziel,[1,*] and Charles Gauthier[1,*]

[1]Centre Armand-Frappier Santé Biotechnologie, Institut national de la recherche scientifique (INRS), 531, boulevard des Prairies, Laval (Québec), Canada, H7V 1B7;

[2]Laboratoire d'analyse et de séparation des essences végétales (LASEVE), Département des Sciences Fondamentales, Université du Québec à Chicoutimi, 555, boulevard de l'Université, Chicoutimi (Québec), Canada, G7H 2B1;

[3]Université de Reims Champagne-Ardenne, INRAE, USC RIBP 1488, SFR Condorcet-FR CNRS 3417, 51100 Reims, France;

[4]School of Pharmacy and Biomolecular Sciences, Liverpool John Moores University, L3 3AF, Liverpool, United Kingdom.

* Corresponding author. Tel.: +1 450-687-5010; E-mail address: charles.gauthier@inrs.ca; eric.deziel@inrs.ca

TABLE OF CONTENTS

1. Supplementary Schemes, Figures, and Tables………………………………………… p. S3

2. General Methods………………………………………………………………………. p. S13

3. Experimental Procedures for Isolation and Synthesis…………………………………. p. S14

4. Experimental Procedures for Biological Evaluation and Surfactant Properties..………p. S97

5. Molecular Modeling……………………………………………………………………… p. S102

6. NMR Spectra for New Compounds……………………………………..……………p. S104

7. HPLC Chromatograms of Synthetic and Natural Surfactants…….…………………… p. S306

8. References…………………….………………………………………………………….. p. S308

1. Supplementary Schemes, Figures, and Tables

Scheme S1. Synthesis of Monolipid 13 from Meldrum's Acid (S1).

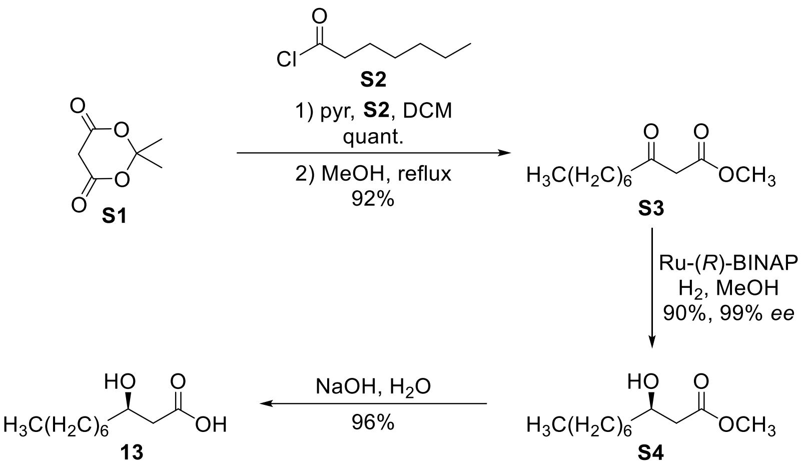

Scheme S2. Alternative Synthesis of Rhamnolipid 3.

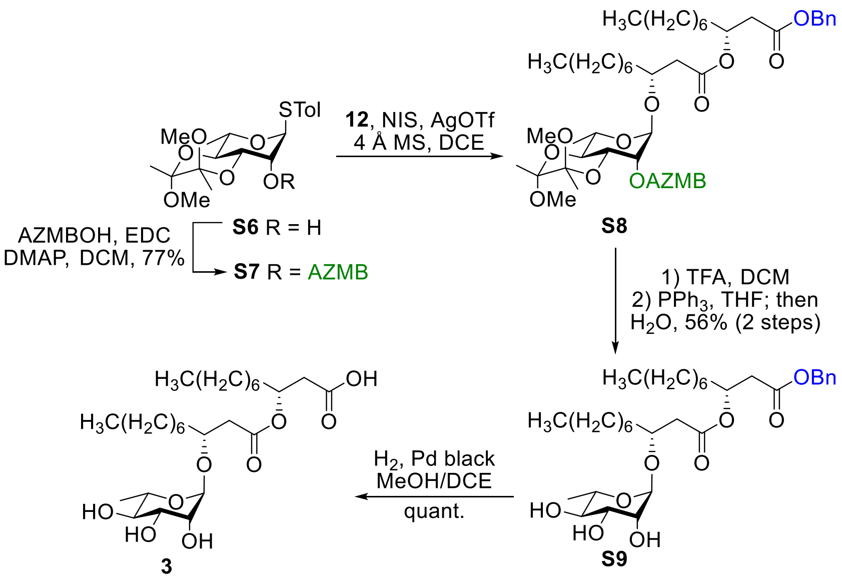

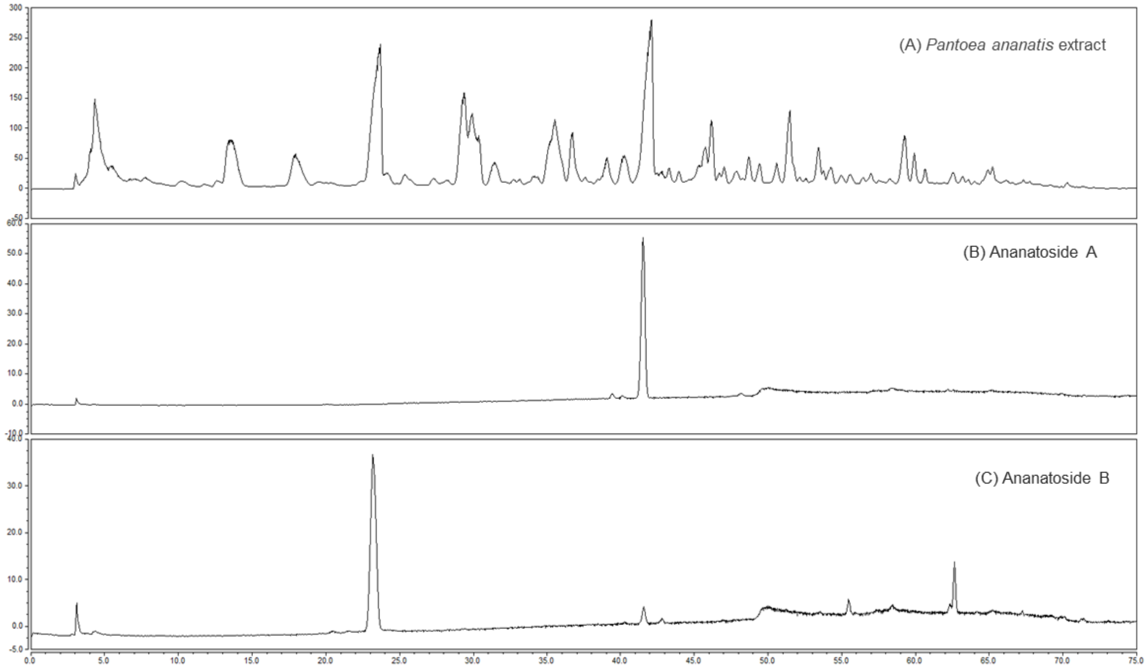

Figure S1. HPLC-CAD chromatograms comparison of (A) crude ethyl acetate *Pantoea Ananatis* extract, (B) synthetic ananatoside A (**1**) and (C) synthetic ananatoside B (**2**).

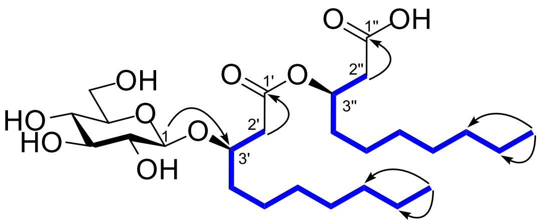

Figure S2. Proposed structure of ananatoside B (**2**) along with key 2D NMR COSY (▬) and HMBC (→) correlations.

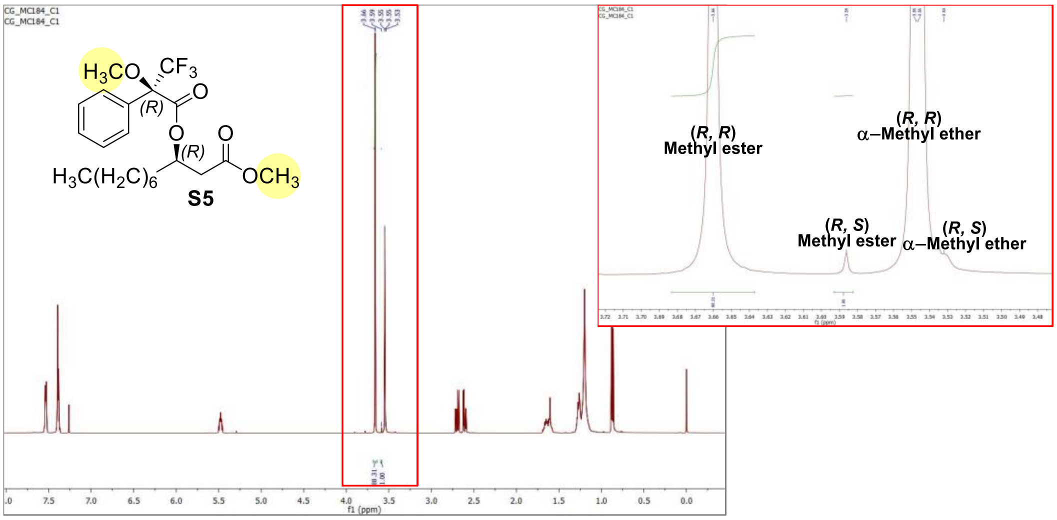

Figure S3. Enlargement of the ^{1}H NMR spectrum of Mosher's ester **S5**.

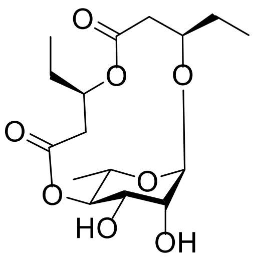

40α

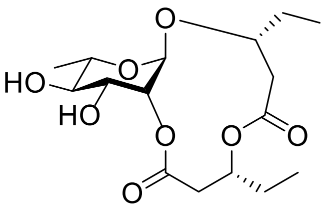

41α

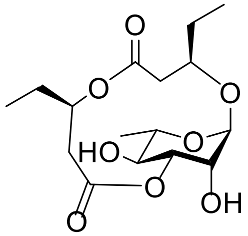

42α

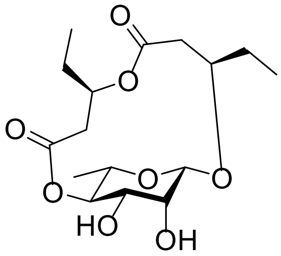

40β

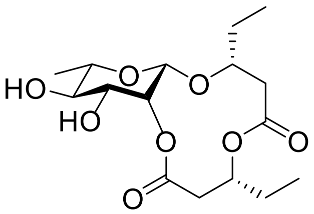

41β

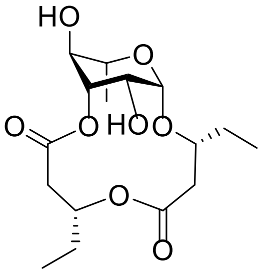

42β

Figure S4. Structures of the *in silico* modelized compounds.

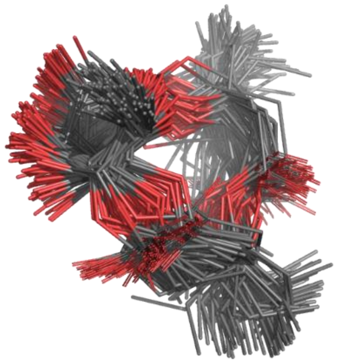

40*α*

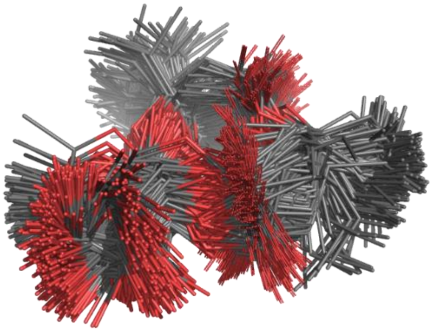

41*α*

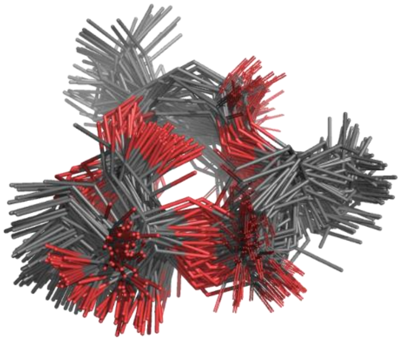

42*α*

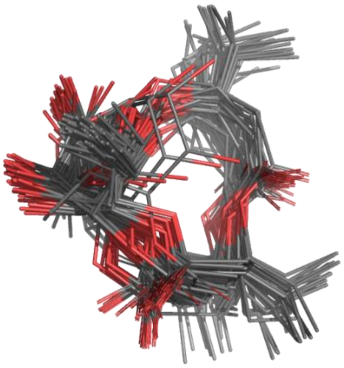

40*β*

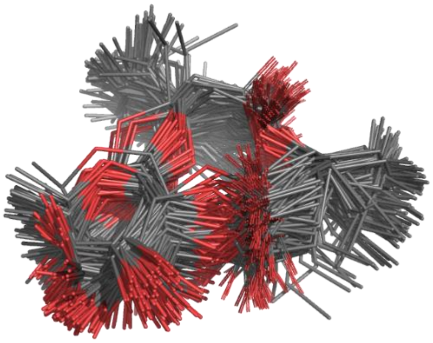

41*β*

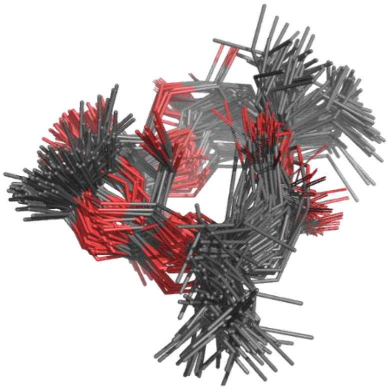

42*β*

Figure S5. MMFF94 conformer populations for the macrolactonized rhamnolipid models **40–42**. Only carbon (grey) and oxygen (red) are displayed for simplicity.

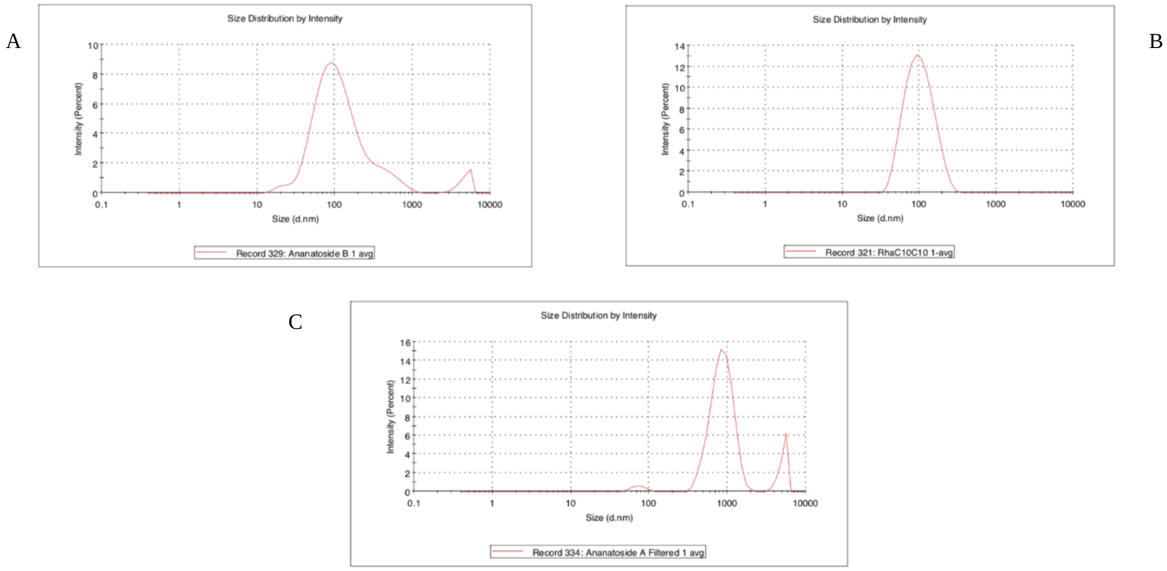

Figure S6. Size distribution of (A) ananatoside B (**2**), (B) Rha$C_{10}C_{10}$ (**3**), and (C) ananatoside A (**1**) measured by DLS.

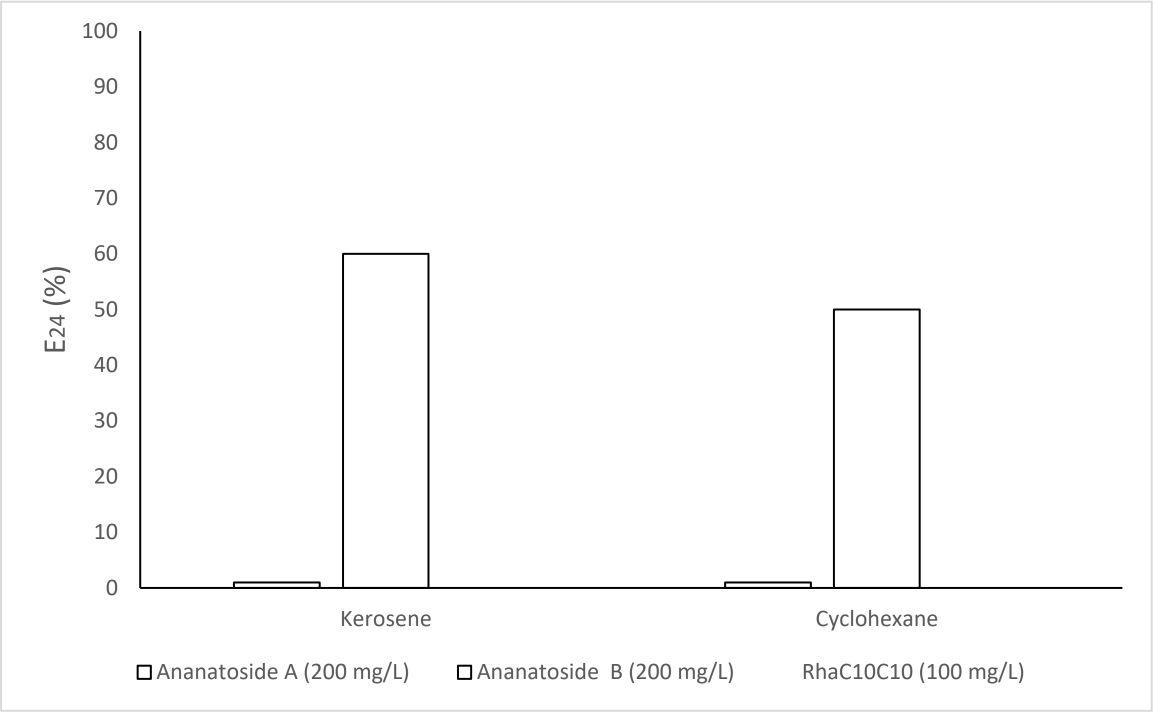

Figure S7. Emulsification activity (E_{24}) of kerosene and cyclohexane by ananatoside A (**1**), ananatoside B (**2**), and $RhaC_{10}C_{10}$ (**3**).

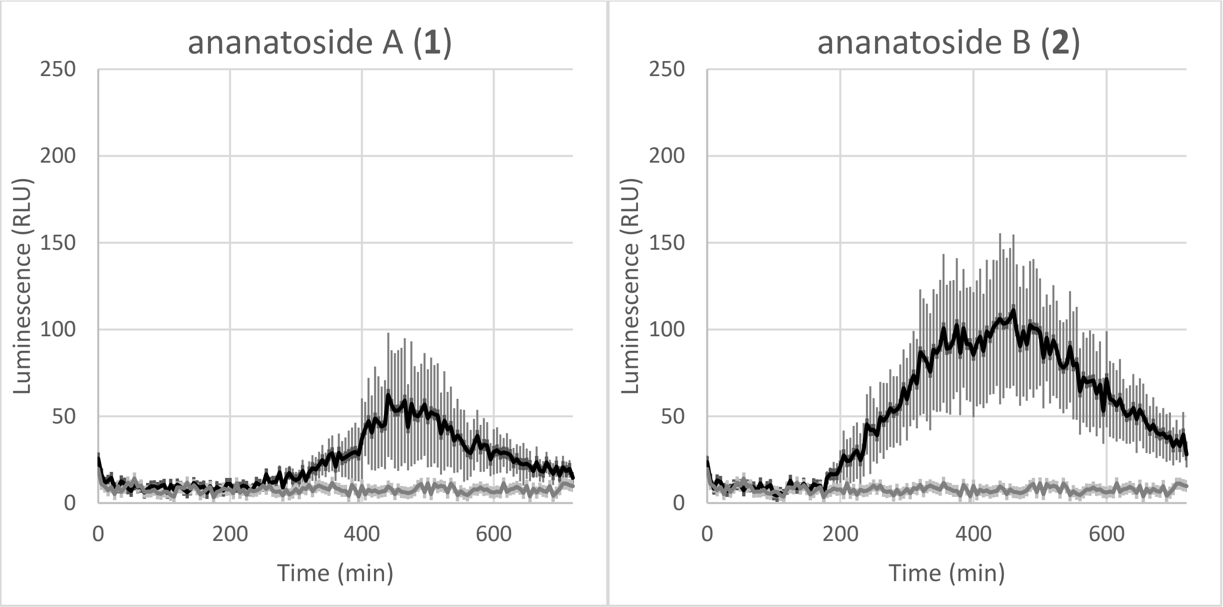

Figure S8. Extracellular ROS production following treatment of *Arabidopsis* petiole with ananatoside A (**1**) or ananatoside B (**2**). Production of reactive oxygen species (ROS) was measured in *Arabidopsis* petiole following treatment at 100 μM with ananatoside A (**1**) and ananatoside B (**2**). Methanol (0.5%) was used as a control. ROS production was measured using the chemiluminescence of luminol and photon counts were expressed as relative luminescence units (RLUs). Data are mean ± SEM (*n* = 6). Experiments were realized three times with similar results.

Table S1. ^{13}C and ^{1}H NMR MAE Values Related to Comparison of Experimental and Predicted Chemical Shifts.

	Comparison pair	mPW1PW91/6-31g(d,p)		mPW1PW91/6-311+g(d,p)		B97-2/cc-pVTZ	
		^{13}C MAE	^{1}H MAE	^{13}C MAE	^{1}H MAE	^{13}C MAE	^{1}H MAE
Classic comparison	**4** → **40*α***	0.90	0.17	3.13	0.08	3.01	0.09
	**4** → **40*β***	1.62	0.24	3.60	0.15	3.05	0.18
	**5*α*** → **41*α***	0.89	0.08	2.77	0.08	2.75	0.09
	**5*α*** → **41*β***	3.06	0.28	3.87	0.30	4.54	0.26
	**5*β*** → **41*α***	1.74	0.23	3.45	0.14	3.04	0.19
	**5*β*** → **41*β***	1.39	0.12	2.86	0.12	3.43	0.10
	**6*α*** → **42*α***	2.58	0.24	4.05	0.17	3.73	0.19
	**6*α*** → **42*β***	3.49	0.25	4.30	0.25	4.69	0.23
	**6*β*** → **42*α***	3.02	0.31	3.74	0.23	3.08	0.23
	**6*β*** → **42*β***	1.08	0.12	2.91	0.10	2.96	0.09
Comparison alignment	**5*α*** → **41*α***; **5*β*** → **41*β***	1.43	0.12	1.25	0.09	1.36	0.08
	**5*α*** → **41*β***; **5*β*** → **41*α***	4.67	0.47	5.01	0.42	5.10	0.41
	**6*α*** → **42*α***; **6*β*** → **42*β***	2.32	0.24	2.12	0.21	2.14	0.19
	**6*α*** → **42*β***; **6*β*** → **42*α***	6.38	0.50	6.02	0.45	5.71	0.44

Table S2. Number of Conformers Retained after Each Step of Modeling.

Filter	Number of Conformers					
	40α	***40β***	***41α***	***41β***	***42α***	***42β***
ETKDGv2	100 000	100 000	100 000	100 000	100 000	100 000
1st RMSD (0.5 Å)	420	255	831	710	363	497
MMFF94s energy window (80 kJ•mol^{-1})	262	133	523	306	238	216
2nd RMSD (0.25 Å)	137	73	367	229	135	114
mPW1PW91/6-31G(d,p) energy window (10 kJ•mol^{-1})	10	10	15	9	12	5

2. General Methods

All starting materials and reagents were purchased from commercial sources and used as received without further purification. Air and water sensitive reactions were performed in oven-dried glassware under an Ar atmosphere. Moisture sensitive reagents were introduced *via* dried syringe. Anhydrous solvents were either prepared from commercial solvents and dried over heat-gun activated 4 Å molecular sieves (MS) or supplied over MS and used as received. Powdered 4 Å MS were activated before use by heating with a heat gun for approx. 15 min under high vacuum. Reactions were monitored by thin-layer chromatography (TLC) with silica gel 60 F_{254} 0.25 mm pre-coated aluminum foil plates. Compounds were visualized by using UV_{254} and/or orcinol (1 mg•mL^{-1}) in 10% aqueous H_2SO_4 solution with heating and/or CAM and/or $KMnO_4$ with heating. Normal-phase flash column chromatography was performed on silica gel 60 Å (15-40 μm). NMR spectra were recorded at 297 K in the indicated solvent ($CDCl_3$, py-d_5) with 400 or 600 MHz instruments, employing standard softwares given by the manufacturer. ^{1}H and ^{13}C NMR spectra were referenced to tetramethylsilane (TMS, $\delta_H = \delta_C = 0.00$ ppm) as internal reference. Assignments were based on ^{1}H, ^{13}C, COSY, HSQC, undecoupled HSQC, and HMBC experiments. High-resolution mass spectra (HRMS) were recorded on an ESI-Q-TOF mass spectrometer. Optical rotations $[\alpha]_D^{20}$ were measured on an Anton Paar polarimeter. The retention factors (R_f) were calculated from silica gel F_{254} 0.25 mm pre-coated glass TLC plates. Preparative TLC purification was accomplished using PLC silica gel F_{254} 1 mm pre-coated 20 × 20 cm glass TLC plates.

3. Experimental Procedures for Isolation and Synthesis

Bacterial Culture for Isolation.

Pre-culture tubes of *Pantoea ananatis* BRT175 (3 mL) were grown at 30 °C in LB medium with shaking (240 rpm) in a TC-7 roller drum (New Brunswick, Canada). Under exponential growth phase, LB medium pre-culture flasks (100 mL, each) were seeded with an initial OD_{600} = 0.1 and cultures were incubated overnight at 30 °C with shaking (150 rpm). Six two-liter flasks, each containing 500 mL Mineral Salts Medium (MSM), were inoculated at an initial OD_{600} = 0.1 and cultures were grown at 30 °C with shaking (150 rpm) for five days. The MSM contained ($g{\bullet}L^{-1}$): 0.9 Na_2HPO_4, 0.7 KH_2PO_4, 2.0 $NaNO_3$, 0.1 $CaCl_2{\bullet}2H_2O$, 0.4 $MgSO_4{\bullet}7H_2O$, and trace element solution (2 $mL{\bullet}L^{-1}$). The composition of trace element solution was ($g{\bullet}L^{-1}$): 2.0 $FeSO_4{\bullet}7H_2O$, 1.5 $MnSO_4{\bullet}H_2O$ and 0.6 $(NH_4)_6Mo_7O_{24}{\bullet}4H_2O$. Dextrose (20 $g{\bullet}L^{-1}$) was provided as a carbon source.

Isolation of Ananatoside A (1) and Ananatoside B (2).

At the end of the cultivation period, culture supernatant was recovered by centrifugation and concentrated HCl was added to reach a final pH = 3. Pooled supernatants (3 L total) were then extracted twice with equal volumes of EtOAc. The organic fractions were then pooled, dried over anhydrous $MgSO_4$, and concentrated under reduced pressure. The crude extract (0.2 g) was suspended in CH_3CN/H_2O (1:1) and used directly for purification. Semi-preparative HPLC purification was performed on a Thermo Fisher Scientific Ultimate 3000 HPLC-CAD system equipped with a Dionex LPG-3400SD pump, a WPS-3000SL autosampler, a TCC-3000SD column oven, and a charged aerosol detector (CAD) Corona Veo. The power function value was set at 1.0, the filter at 1 s, the data collection rate at 10 Hz, and the evaporator temperature at 35 °C. Nitrogen (57.2 psi) was used for nebulization. All data were analyzed using the Thermo Fischer Chromeleon 7.2.9 software. For the purification, a reverse phase column Hypersil Gold (250 × 10 mm) was

used with a mobile phase consisting of CH_3CN/H_2O gradient containing 0.1% formic acid. Prior the injection, the column was equilibrated for 10 min with 50% of CH_3CN. The injection volume was set to 250 μL. The elution gradient started from 50 to 60% CH_3CN for 20 min, then to 100% CH_3CN within the next 40 min and hold for 15 min. HPLC flow rate was set to 5.0 mL•min^{-1} and the oven temperature was set at 28 °C. A flow splitter was used after the column to deliver only 5% of the mixture to the CAD detector and the remaining to the fraction collector. Fractions containing ananatoside A (**1**, 42.0 min) and ananatoside B (**2**, 23.6 min), respectively, were pooled and concentrated under reduced pressure to give ananatoside A (**1**, 37 mg) as a light yellow oil (physical and analytical data agreed with those published)[1] and ananatoside B (**2**, 30 mg) as a white amorphous powder. Data for ananatoside B (**2**): $[\alpha]^{20}_{D}$ +14.6 (*c* 0.24; EtOAc); ^{1}H NMR (600 MHz, pyr-d_5) δ (ppm) 5.81-5.77 (m, 1H, H-3''), 4.99 (d, J = 7.7 Hz, 1H, H-1), 4.60-4.58 (m, 1H, H-3'), 4.52 (d, J = 10.4 Hz, 1H, H-6a), 4.39 (dd, J = 11.6 Hz, J = 5.3 Hz, 1H, H-6b), 4.26-4.21 (m, 2H, H-3, H-4), 4.03 (t, J = 7.9 Hz, 1H, H-2), 3.95-3.91 (m, 1H, H-5), 3.27 (dd, $J_{2a'-2b'}$ = 15.0 Hz, $J_{2a'-3'}$ = 5.4 Hz, 1H, H-2a'), 3.04 (dd, $J_{2a''-2b''}$ = 15.5 Hz, $J_{2a''-3''}$ = 6.9 Hz, H-2a''), 2.87 (dd, $J_{2b''-2a''}$ = 15.8 Hz, $J_{2b''-3''}$ = 5.7 Hz, 1H, H-2b''), 2.82 (dd, $J_{2b'-2a'}$ = 15.1 Hz, $J_{2b'-3'}$ = 7.3 Hz, 1H, H-2b'), 1.84-1.17 (m, 24H, 12 × CH_2), 0.85-0.80 (m, 6H, 2 × CH_3); ^{13}C NMR (150 MHz, pyr-d_5) δ (ppm) 173.8, 172.0 (2C, C-1', C-1''), 105.4 (C-1), 79.0, 78.7 (2C, C-5, C-3), 77.8 (C-3'), 75.8 (C-2'), 72.2, 72.0 (2C, C-3'', C-4), 63.5 (C-6), 42.6 (C-2'), 40.4 (C-2''), 35.8-23.3 (12C, 12 × CH$_2$), 14.7 (2C, 2 × CH$_3$); $[\alpha]^{20}_{D}$ +103 (*c* 0.2, $CHCl_3$); HRMS (ESI-TOF) *m/z* $[M + NH_4]^+$ calcd for $C_{26}H_{52}NO_{10}$ 538.3586; found 538.3587; *m/z* $[M + Na]^+$ calcd for $C_{26}H_{48}NaO_{10}$ 543.3140; found 543.3142.

HPLC Analysis of Natural and Synthetic Compounds. Analytical reversed phase HPLC analyses were performed using the same equipment as described above. For the separation, a reverse phase column Hypersil Gold (250 × 4.6 mm) was employed. The method was the same as described for the semi-preparative purification. The injection volume was set to 50 μL, the flow rate was 0.8 mL•min^{-1}, oven temperature was set to 28 °C, and detection was accomplished using the CAD detector. The gradients consisted of: method A: 50 to 60% CH_3CN (20 min), 60 to 100% CH_3CN (30 min), and 100% CH_3CN (10 min); method B: 50 to 60% CH_3CN (10 min), 60 to 100% CH_3CN (25 min), and 100% CH_3CN (25 min); method C: 50 to 80% CH_3CN (50 min) and 80 to 90% CH_3CN (10 min); and method D: 50 to 60% CH_3CN (20 min) and 60 to 100% CH_3CN (40 min).

Methyl 3-Oxodecanoate (S3).

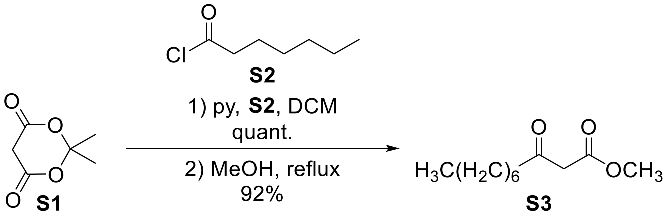

To a solution of Meldrum's acid (**S1**, 7.44 g, 51.64 mmol, 1.05 equiv) in anhydrous DCM (22 mL) at 0 °C under Ar was slowly added anhydrous pyridine (8.0 mL, 98 mmol, 2.0 equiv). A solution of octanoyl chloride (**S2**, 8.00 g, 49.18 mmol, 1.0 equiv) in anhydrous DCM (15 mL) was then added dropwise, and the mixture was stirred for 1 h at rt under Ar. The solution was washed with aqueous 2 M HCl (2 × 40 mL), the aqueous layer was extracted with DCM (2 × 15 mL), and the combined organic phases were washed with aqueous 2 M HCl (2 × 15 mL) and brine (40 mL). The organic phase was dried over $MgSO_4$, filtered, and evaporated under reduced pressure to give the corresponding enol as a brown-red oil (13.3 g, quant.). The latter compound (12.8 g, 47.4 mmol, 1.0 equiv) was solubilized in anhydrous MeOH and the solution was refluxed for 3 h under Ar. The solution was evaporated under reduced pressure and the residue was purified by silica gel flash chromatography (Hex/EtOAc 10:0 to 95:5) to give keto-ester **S3** as a colorless oil (8.77 g, 92%). Physical and analytical data of compound **S3** agreed with those published.[2]

(*R*)-Methyl 3-Hydroxydecanoate (S4).

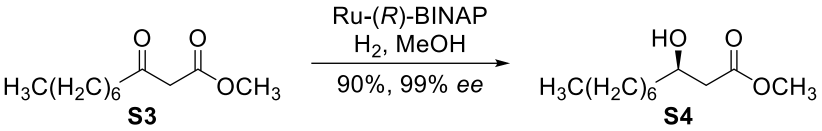

Preparation of the catalyst: In a heat gun-dried reaction flask under an Ar atmosphere were added (*R*)-BINAP (379 mg, 0.608 mmol, 0.024 equiv) and (COD)Ru(2-methylallyl)$_2$ (162 mg, 0.507 mmol, 0.020 equiv). The reactants were then solubilized in anhydrous acetone (25 mL), which was previously degassed with Ar. A solution of 48% aqueous HBr (0.13 mL) in degassed anhydrous MeOH (6.3 mL) was added to the reaction flask, and the mixture was stirred at rt for 30 min under an Ar atmosphere. The solvents were then evaporated under nitrogen flux.

Preparation of hydroxy-ester: To the previously prepared catalyst was cannulated a solution of keto-ester **S3** (5.07 g, 25.33 mmol, 1.0 equiv) in anhydrous degassed MeOH (51 mL). The solution was stirred at 55 °C for 18 h under an H_2 atmosphere. The solution was then cooled at 0 °C, filtered over Celite, and evaporated under reduced pressure. The residue was purified by silica gel flash chromatography (Hex/EtOAc 9:1 to 8:2) to give hydroxy-ester **S4** (4.61 g, 90%) as a colorless oil. Physical and analytical data of compound **S4** agreed with those published.[2] The enantiomeric purity of compound **S4** was determined through the synthesis of Mosher's ester **S5**.[3]

(*R*)-Methyl *α*-Methoxy-*α*-trifluoromethylphenylacetyl-(*R*)-3-hydroxydecanoate (S5).

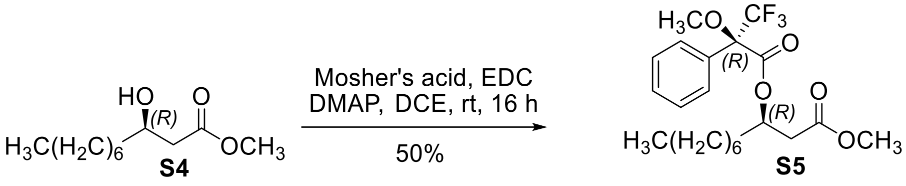

Methyl ester **S4** (15 mg, 0.074 mmol, 1.0 equiv) was solubilized in anhydrous DCE (0.4 mL). Mosher's acid (26 mg, 0.11 mmol, 1.5 equiv), EDC (28 mg, 0.15 mmol, 2.0 equiv), and DMAP (2 mg, 0.02 mmol, 0.2 equiv) were successively added to the solution, which was then stirred at rt for 16 h under an Ar atmosphere. The solution was evaporated under reduced pressure and the residue was purified by silica gel flash chromatography (DCM) to give Mosher's ester **S5** (15 mg, 50%) as a colorless oil. Physical and analytical data of compound **S5** agreed with those published.[3] Analysis of this spectrum allowed to determine the enantiomeric purity of methyl ester **S4** (99% *ee*, see Fig. S3) as previously reported.[3]

(*R*)-3-Hydroxydecanoic Acid (13).

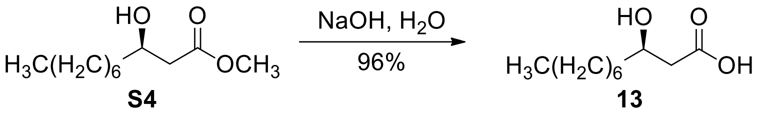

Alcohol **S4** (4.31 g, 21.3 mmol, 1.0 equiv) was solubilized in aqueous 1 M NaOH (43 mL) at 0 °C. The mixture was stirred at 0 °C for 1 h, then at rt for an additional 1.5 h. The solution was acidified with aqueous 2 M HCl until a pH of ~2−3 was reached and the aqueous phase was extracted with EtOAc (3×). The combined organic layers were washed with brine, dried over anhydrous $MgSO_4$, filtered, and evaporated under reduced pressure to give acid **13** (3.84 g, 96%) as a white amorphous solid without further purification. Physical and analytical data of compound **13** agreed with those published.[2]

(*R*)-Benzyl 3-Hydroxydecanoate (14).

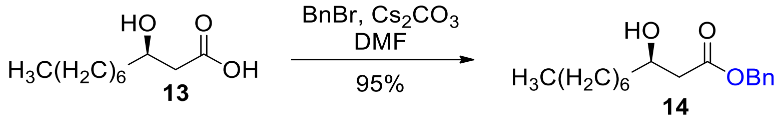

Acid **13** (143 mg, 0.761 mmol, 1.0 equiv) was solubilized in anhydrous DMF (3.8 mL) under Ar. BnBr (0.20 mL, 1.7 mmol, 2.2 equiv) and Cs_2CO_3 (347 mg, 1.07 mmol, 1.4 equiv) were successively added and the mixture was stirred for 21 h at rt under Ar. The suspension was diluted with DCM and washed with saturated aqueous NH_4Cl. The aqueous layer was extracted with DCM (2×). The combined organic layers were washed with brine, dried over anhydrous $MgSO_4$, filtered, and evaporated under reduced pressure. The residue was purified by silica gel flash chromatography (Hex/EtOAc 95:5 to 85:5) to give benzyl ester **14** (201 mg, 95%) as a colorless oil: R_f 0.24 (Hex/EtOAc 8:2); $[\alpha]^{20}_{D}$ –15 (*c* 0.6, $CHCl_3$); ^1H NMR (600 MHz, $CDCl_3$) δ (ppm) 7.38–7.32 (m, 5H, 5 × CH_{Bn}), 5.15 (s, 2H, CH_{2Bn}), 4.04–4.01 (m, 1H, H-3), 2.85 (d, J = 3.5 Hz, 1H, O*H*), 2.56 (dd, $J_{2a\text{-}2b}$ = 16.5 Hz, $J_{2a\text{-}3}$ = 3.0 Hz, 1H, H-2a), 2.46 (dd, $J_{2b\text{-}2a}$ = 16.5 Hz, $J_{2b\text{-}3}$ = 9.1 Hz, 1H, H-2b), 1.54–1.26 (m, 12H, H-4, H-5, H-6, H-7, H-8, H-9), 0.88 (t, J = 7.0 Hz, 3H, H-10); ^{13}C NMR (150 MHz, $CDCl_3$) δ (ppm) 173.0 (C-1), 135.7 (C_{Bn}), 128.8 (2C, 2 × CH_{Bn}), 128.5 (CH_{Bn}), 128.4 (2C, 2 × CH_{Bn}), 68.2 (C-3), 66.6 (CH_{2Bn}), 41.5 (C-2), 36.7–22.8 (6C, C-4, C-5, C-6, C-7, C-8, C-9), 14.2 (C-10); HRMS (ESI-TOF) *m/z* $[M + Na]^+$ calcd for $C_{17}H_{26}NaO_3$ 301.1774; found 301.1764.

(*R*)-3-(*tert*-Butyldimethylsilyloxy)decanoic acid (15).

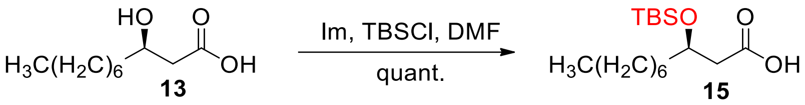

Imidazole (904 mg, 13.3 mmol, 10.0 equiv) was added to a solution of TBSCl (701 mg, 4.65 mmol, 3.5 equiv) in anhydrous DMF (2.3 mL) at 0 °C. The mixture was stirred at 0 °C for 15 min under an Ar atmosphere, after which a solution of acid **13** (252 mg, 1.33 mmol, 1.0 equiv) in anhydrous DMF (0.5 mL) was added. The solution was stirred at rt for 16 h under an Ar atmosphere, then transferred to a separatory funnel. Brine was added to the mixture and the latter was extracted with a Hex/Et_2O mixture (3:1 *v/v*). The organic layer was dried over anhydrous $MgSO_4$, filtered, and evaporated under reduced pressure. The residue was solubilized in a mixture of MeOH (36 mL) and THF (18 mL), to which a solution of K_2CO_3 (450 mg, 3.26 mmol, 2.45 equiv) in H_2O (6 mL) was added at 0 °C. The mixture was stirred at 0 °C for 1 h after which brine (18 mL) was added. The solution was acidified with aqueous 1 M HCl until a pH of ~3 was reached. The solution was extracted with a Hex/Et_2O mixture (3:1 *v/v*), dried over anhydrous $MgSO_4$, filtered, and evaporated under reduced pressure. The residue was dried under high vacuum for 16 h (evaporation of the remaining solvents and TBS alcohol) to give acid **15** (402 mg, quant.) as a colorless oil. Physical and analytical data of compound **15** agreed with those published.[2]

(*R*)-Benzyl 3-(((*R*)-3-((*tert*-Butyldimethylsilyl)oxy)decanoyl)oxy)decanoate (16).

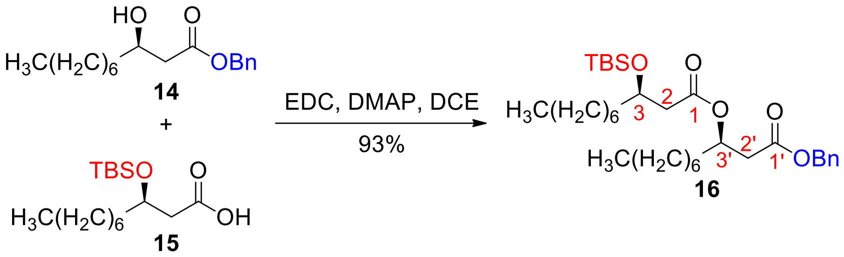

Alcool **14** (91 mg, 0.32 mmol, 1.0 equiv) and acid **15** (121 mg, 0.388 mmol, 1.2 equiv) were solubilized in anhydrous DCE (3.9 mL) under Ar. EDC (186 mg, 0.970 mmol, 3.0 equiv) and DMAP (12 mg, 0.10 mmol, 0.3 equiv) were successively added and the mixture was stirred at rt for 16 h under Ar. The solution was evaporated under reduced pressure and the residue was purified by silica gel flash chromatography (Hex/EtOAc 95:5) to give dilipid **16** (171 mg, 93%) as a colorless oil: R_f 0.57 (Hex/EtOAc 8:2); $[\alpha]^{20}_{D}$ +53 (*c* 0.7, $CHCl_3$); ^1H NMR (600 MHz, $CDCl_3$) δ (ppm) 7.37−7.31 (m, 5H, 5 × CH_{Bn}), 5.24−5.20 (m, 1H, H-3’), 5.11 (s, 2H, CH_{2Bn}), 4.07 (p, J = 6.8 Hz, 1H, H-3), 2.65 (dd, $J_{2a'-2b'}$ = 15.4 Hz, $J_{2a'-3'}$ = 7.1 Hz, 1H, H-2a’), 2.57 (dd, $J_{2b'-2a'}$ = 15.4 Hz, $J_{2b'-3'}$ = 5.8 Hz, 1H, H-2b’), 2.41 (dd, J_{2a-2b} = 14.8 Hz, J_{2a-3} = 5.9 Hz, 1H, H-2a), 2.36 (dd, J_{2b-2a} = 14.8 Hz, J_{2b-3} = 6.7 Hz, 1H, H-2b), 1.63−1.24 (m, 24H, 12 × CH_2), 0.89−0.86 (m, 15H, H-10, H-10’, C(CH_3)$_{3TBS}$), 0.06 (s, 3H, CH_{3TBS}), 0.04 (s, 3H, CH_{3TBS}); ^{13}C NMR (150 MHz, $CDCl_3$) δ (ppm) 171.1, 170.3 (2C, C-1, C-1’), 135.9 (C_{Bn}), 128.7, 128.4 (5C, 5 × CH_{Bn}), 70.7 (C-3’), 69.4 (C-3), 66.6 (CH_{2Bn}), 42.9 (C-2), 39.3 (C-2’), 37.5−29.3 (8C, 8 × CH_2), 26.0 (3C, C(CH_3)$_{3TBS}$), 25.3−22.8 (4C, 4 × CH_2), 18.2 (C(CH_3)$_{3TBS}$), 14.3, 14.2 (2C, C-10, C-10’), −4.46 (2C, 2 × CH_{3TBS}); HRMS (ESI-TOF) *m/z* $[M + Na]^+$ calcd for $C_{17}H_{26}NaO_3$ 585.3946; found 585.3928.

(*R*)-3-(((*R*)-3-((*tert*-Butyldimethylsilyl)oxy)decanoyl)oxy)decanoic acid (10).

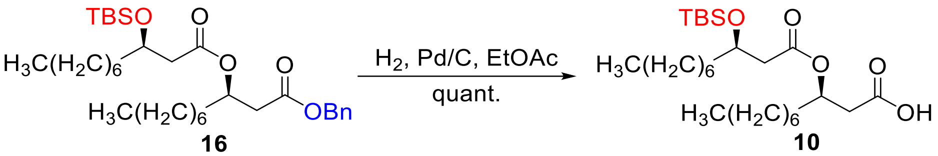

Dilipid **16** (122 mg, 0.216 mmol, 1.0 equiv) was solubilized in EtOAc under an Ar atmosphere, and 10% Pd/C (12 mg, 1 mg•mg^{-1} of dilipid) was added. The suspension was stirred at rt for 20 h under an H_2 atmosphere. The suspension was filtered over Celite and the solvents were evaporated under reduced pressure yielding acid **10** (102 mg, quant.) as a colorless oil: R_f 0.43 (DCM/MeOH 96:4); $[\alpha]^{20}_D$ +19 (*c* 0.7, $CHCl_3$); ^{1}H NMR (600 MHz, $CDCl_3$) δ (ppm) 5.20 (p, J = 6.0 Hz, 1H, H-3'), 4.09 (p, J = 6.0 Hz, 1H, H-3), 2.65 (dd, $J_{2a'-2b'}$ = 15.9 Hz, $J_{2a'-3'}$ = 6.9 Hz, 1H, H-2a'), 2.57 (dd, $J_{2b'-2a'}$ = 15.9 Hz, $J_{2b'-3'}$ = 5.8 Hz, 1H, H-2b'), 2.46 (dd, J_{2a-2b} = 14.9 Hz, J_{2a-3} = 6.0 Hz, 1H, H-2a), 2.41 (dd, J_{2b-2a} = 14.9 Hz, J_{2b-3} = 6.5 Hz, 1H, H-2b), 1.69-1.26 (m, 24H, 12 x CH_2), 0.89-0.85 (m, 15H, H-10, H-10', C(CH_3)$_{3TBS}$), 0.06 (s, 3H, CH_{3TBS}), 0.05 (s, 3H, CH_{3TBS}); ^{13}C NMR (150 MHz, $CDCl_3$) δ (ppm) 176.2, 171.2 (2C, C-1, C-1'), 70.4 (C-3'), 69.3 (C-3), 42.9 (C-2), 38.9 (C-2'), 37.5-29.3 (8C, 8 x CH$_2$), 26.0 (3C, C(CH$_3$)$_{3TBS}$), 25.3-22.8 (4C, 4 × CH$_2$), 18.2 (C(CH$_3$)$_{3TBS}$), 14.3, 14.2 (C-10, C-10'), -4.46 (CH$_{3TBS}$), -4.52 (CH$_{3TBS}$); HRMS (ESI-TOF) *m/z* $[M + H]^+$ calcd for $C_{26}H_{53}O_5Si$ 473.3657; found 473.3653; *m/z* $[M + Na]^+$ calcd for $C_{26}H_{52}NaO_5Si$ 495.3476; found 495.3465.

(*R*)-Benzyl 3-(((*R*)-3-Hydroxydecanoyl)oxy)decanoate (12).

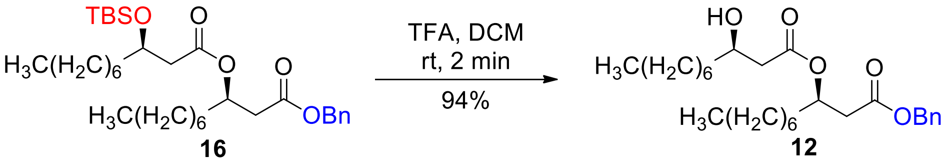

A solution of dilipid **16** (192 mg, 0.340 mmol, 1.0 equiv) in DCM (0.7 mL) was added dropwise in TFA (1.4 mL) during a one-minute period, then the mixture was stirred for one additional minute. The reaction mixture was quenched with saturated aqueous $NaHCO_3$. The organic layer was dried over anhydrous $MgSO_4$, filtered, and evaporated under reduced pressure. The residue was purified by silica gel flash chromatography (Hex/EtOAc 9:1 to 8:2) to give alcohol **12** (143 mg, 94%) as a colorless oil: R_f 0.28 (Hex/EtOAc 8:2); $[\alpha]^{20}_{D}$ –13 (*c* 0.4, $CHCl_3$); ^1H NMR (600 MHz, $CDCl_3$) δ (ppm) 7.38−7.32 (m, 5H, 5 × CH_{Bn}), 5.30-5.26 (m, 1H, H-3’), 5.11 (s, 2H, CH_{2Bn}), 3.98−3.94 (m, 1H, H-3), 2.95 (br s, 1H, OH), 2.63 (dd, $J_{2a'-2b'}$ = 14.2 Hz, $J_{2a'-3'}$ = 6.2 Hz, 1H, H-2a’), 2.60 (dd, $J_{2b'-2a'}$ = 14.2 Hz, $J_{2b'-3'}$ = 4.1 Hz, 1H, H-2b’), 2.42 (dd, J_{2a-2b} = 15.8 Hz, J_{2a-3} = 2.9 Hz, 1H, H-2a), 2.31 (dd, J_{2b-2a} = 15.8 Hz, J_{2b-3} = 9.2 Hz, 1H, H-2b), 1.64−1.25 (m, 24H, 12 × CH_2), 0.89−0.85 (m, 6H, H-10, H-10’); ^{13}C NMR (150 MHz, $CDCl_3$) δ (ppm) 172.6, 170.6 (2C, C-1, C-1’), 135.7 (C_{Bn}), 128.7 (2C, 2 × CH_{Bn}), 128.6 (2C, 2 × CH_{Bn}), 128.5 (CH_{Bn}), 71.0 (C-3’), 68.4 (C-3), 66.8 (CH_{2Bn}), 41.9 (C-2), 39.3 (C-2’), 36.7−22.8 (12C, 12 × CH_2), 14.2 (2C, C-10, C-10’); HRMS (ESI-TOF) *m/z* $[M + Na]^+$ calcd for $C_{27}H_{44}NaO_5$ 471.3081; found 471.3089; *m/z* $[M + K]^+$ calcd for $C_{27}H_{44}O_5K$ 487.2820; found 487.2824.

***para*-Methylphenyl 3,4-Di-*O*-benzyl-6-*O*-*tert*-butyldimethylsilyl-1-thio-*β*-D-glucopyranoside (S10).**

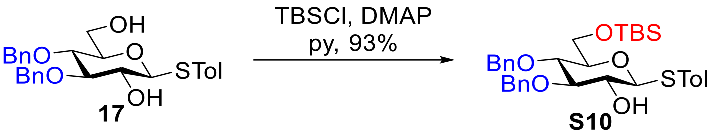

TBSCl (866 mg, 5.54 mmol, 1.5 equiv) and DMAP (45 mg, 0.37 mmol, 0.1 equiv) were successively added to a solution of diol **17**[4] (1.72 g, 3.69 mmol, 1.0 equiv) in anhydrous pyridine (5.6 mL). The mixture was stirred at rt under Ar for 3 h, then diluted in toluene (80 mL), washed with water (30 mL) and brine (30 mL). The organic layer was dried over $MgSO_4$, filtered, and evaporated under pressure. The residue was purified by silica gel flash chromatography (Hex/EtOAc 9:1) furnishing alcohol **S10** (1994.6 mg, 93%) as a colorless oil: R_f 0.38 (Hex/EtOAc 8:2); $[\alpha]^{20}_{D}$ –21 (*c* 1.4, $CHCl_3$); ^1H NMR (600 MHz, $CDCl_3$) δ (ppm) 7.47-7.45 (m, 2H, 2 × C*H*-STol), 7.36-7.27 (m, 10H, 10 × C*H*-Bn), 7.09-7.08 (m, 2H, 2 × C*H*-STol), 4.89 (d, *J* = 11.1 Hz, 1H, C*H*H_{Bn}), 4.85-4.83 (m, 2H, C*H*H_{Bn}, CHH_{Bn}), 4.67 (d, *J* = 10.8 Hz, 1H, CHH_{Bn}), 4.40 (d, *J* = 9.6 Hz, 1H, H-1), 3.90 (dd, $J_{6a\text{-}6b}$ = 11.4 Hz, $J_{6a\text{-}5}$ = 1.8 Hz, 1H, H-6a), 3.87 (dd, $J_{6b\text{-}6a}$ = 11.4 Hz, $J_{6b\text{-}5}$ = 3.6 Hz, 1H, H-6b), 3.61 (t, *J* = 9.1 Hz, 1H, H-4), 3.58 (t, *J* = 8.6 Hz, 1H, H-3), 3.40 (t, *J* = 8.9 Hz, 1H, H-2), 3.34 (ddd, $J_{5\text{-}4}$ = 9.2 Hz, $J_{5\text{-}6b}$ = 3.5 Hz, $J_{5\text{-}6a}$ = 1.8 Hz, 1H, H-5), 2.37 (s, 1H, OH), 2.33 (s, 3H, CH_{3STol}), 0.92 (s, 9H, C(CH_3)$_{3TBS}$), 0.10 (s, 3H, CH_{3TBS}), 0.09 (s, 3H, CH_{3TBS}); ^{13}C NMR (150 MHz, $CDCl_3$) δ (ppm) 138.6 (*C*-Ar), 138.50 (*C*-Ar), 138.48 (*C*-Ar), 133.8 (2C, 2 × *C*H-STol), 129.8 (2C, 2 × *C*H-STol), 128.6-127.6 (11C, 1 × *C*-Ar, 10 x *C*H-Bn), 88.1 (C-1), 86.1 (C-3), 80.5 (C-5), 77.1 (C-4), 75.5 (*C*H_{2Bn}), 75.2 (*C*H_{2Bn}), 72.5 (C-2), 62.2 (C-6), 26.1 (3C, C(*C*H_3)$_{3TBS}$), 21.3 (*C*H_{3STol}), 18.5 (*C*(CH_3)$_{3TBS}$), -5.0 (*C*H_{3TBS}), -5.2 (*C*H_{3TBS}); HRMS (ESI-TOF) *m/z* $[M + NH_4]^+$ calcd for $C_{33}H_{48}NO_5SSi$ 598.3017; found 598.3019; *m/z* $[M + Na]^+$ calcd for $C_{33}H_{44}NaO_5SSi$ 603.2571; found 603.2578.

***para*-Methylphenyl 3,4-Di-*O*-benzyl-6-*O*-*tert*-butyldimethylsilyl-2-*O*-levulinoyl-1-thio-*β*-D-glucopyranoside (11).**

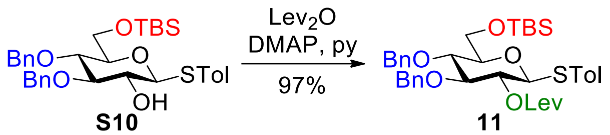

Alcohol **S10** (507 mg, 0.861 mmol, 1.0 equiv) was solubilized in anhydrous pyridine (5.6 mL) and DMAP (263 mg, 2.15 mmol, 2.5 equiv) was added to the solution. A solution of Lev_2O (1.42 g, 6.63 mmol, 7.7 equiv) in anhydrous pyridine (6.6 mL) was added dropwise to the mixture, which was then stirred at 50 °C for 1 h under an Ar atmosphere. The solvents were then evaporated under reduced pressure and co-evaporated with toluene. The residue was purified by silica gel flash chromatography (Hex/EtOAc 9:1 to 8:2) to give fully protected **11** (567 mg, quant.) as a colorless oil: R_f 0.37 (Hex/EtOAc 7:3); $[\alpha]^{20}_{D}$ +54 (*c* 0.9, $CHCl_3$); ^{1}H NMR (600 MHz, $CDCl_3$) δ (ppm) 7.40-7.39 (m, 2H, 2 × CH_{STol}), 7.34-7.25 (m, 10H, 10 × CH_{Bn}), 7.08-7.07 (m, 2H, 2 × CH_{STol}), 4.93-4.89 (m, 1H, H-2), 4.79 (d, *J* = 11.3 Hz, 1H, C*H*H_{Bn}), 4.78 (d, *J* = 10.9 Hz, 1H, C*H*H_{Bn}), 4.69 (d, *J* = 11.3 Hz, 1H, CHH_{Bn}), 4.67 (d, *J* = 10.9 Hz, 1H, CHH_{Bn}), 4.52 (d, *J* = 10.0 Hz, 1H, H-1), 3.88 (dd, $J_{6a\text{-}6b}$ = 11.4 Hz, $J_{6a\text{-}5}$ = 1.6 Hz, 1H, H-6a), 3.84 (dd, $J_{6b\text{-}6a}$ = 11.5 Hz, $J_{6b\text{-}5}$ = 3.9 Hz, 1H, H-6b), 3.69-3.65 (m, 2H, H-3, H-4), 3.34-3.31 (m, 1H, H-5), 2.73-2.71 (m, 2H, CH_{2Lev}), 2.57 (dt, $J_{Ha\text{-}Hb}$ = 17.2 Hz, $J_{Ha\text{-}CH2}$ = 6.6 Hz, 1H, C$H^{a}H_{Lev}$), 2.48 (dt, $J_{Hb\text{-}Ha}$ = 17.2 Hz, $J_{Hb\text{-}CH2}$ = 7.0 Hz, 1H, CHH^{b}_{Lev}), 2.32 (s, 3H, CH_{3STol}), 2,17 (s, 3H, CH_{3Lev}), 0.91 (s, 9H, C(CH_3)$_{3TBS}$), 0.10 (s, 3H, CH_{3TBS}), 0.07 (s, 3H, CH_{3TBS}); ^{13}C NMR (150 MHz, $CDCl_3$) δ (ppm) 206.4 (*C*O_{Lev}), 171.5 (*C*OOR_{Lev}), 138.33 (C_{Ar}), 138.28 (C_{Ar}), 138.1 (C_{Ar}), 133.3-127.8 (15C, C_{Ar}, 14 × *C*H_{Ar}), 86.3 (C-1), 84.5 (C-3), 80.4 (C-5), 77.5 (C-4), 75.4 (*C*H_{2Bn}), 75.2 (*C*H_{2Bn}), 72.4 (C-2), 62.2 (C-6), 38.0 (*C*H_{2Lev}), 30.1 (*C*H_{3Lev}), 28.3 (*C*H_{2Lev}), 26.1 (3C, C(*C*H_3)$_{3TBS}$), 21.3 (*C*H_{3STol}), 18.5 (*C*(CH_3)$_{3TBS}$),

-5.0 (CH_{3TBS}), -5.2 (CH_{3TBS}). HRMS (ESI-TOF) *m/z* $[M + K]^+$ calcd for $C_{38}H_{50}KO_7SSi$ 701.2939; found 701.2940; *m/z* $[M + Na]^+$ calcd for $C_{38}H_{50}NaO_7SSi$ 717.2678; found 717.2688.

Benzyl (*R*)-3-*O*-[(*R*)-(3'-*O*-Decyl)-3,4-di-*O*-benzyl-6-*O*-*tert*-butyldimethylsilyl-2-*O*-levulinoyl-*β*-D-glucopyranosyl]decanoate (8).

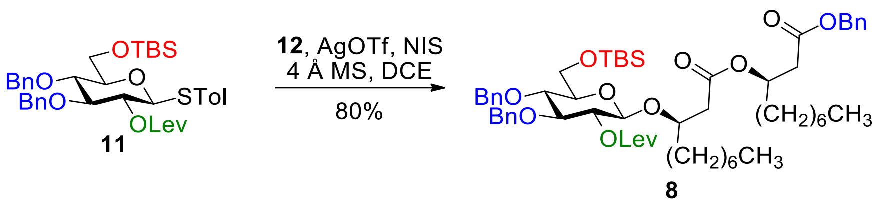

Donor **11** (221 mg, 0.326 mmol, 1.2 equiv), acceptor **12** (122 mg, 0.272 mmol, 1.0 equiv), and NIS (98 mg, 0.44 mmol, 1.6 equiv) were dried under high vacuum for 1 h. Activated 4 Å MS (488 mg, 4 mg•mg^{-1} of acceptor **12**) and anhydrous DCE (5.4 mL) were added and the suspension was stirred under an Ar atmosphere for 1 h. The mixture was cooled to –10 °C and AgOTf (14 mg, 0.054 mmol, 0.2 equiv) was added while the reaction flask was protected from light with aluminum foil. The suspension was stirred from –10 to 0 °C for 30 min, quenched with Et_3N, and filtered over Celite. The solvents were evaporated under reduced pressure and the residue was purified by silica gel flash chromatography (Hex/EtOAc 95:5 to 9:1) to give glucolipid **8** (219 mg, 80%) as a yellowish oil: R_f 0.35 (Hex/EtOAc 8:2); $[\alpha]^{20}_{D}$ –3 (*c* 0.5, $CHCl_3$); ^{1}H NMR (600 MHz, $CDCl_3$) δ (ppm) 7.37-7.28 (m, 15H, 15 x CH_{Bn}), 5.23-5.19 (m, 1H, H-3''), 5.10 (s, 2H, CH_{2COOBn}), 4.89 (dd, $J_{2\text{-}3}$ = 9.2 Hz, $J_{2\text{-}1}$ = 8.2 Hz, 1H, H-2), 4.80-4.78 (m, 2H, CHH_{Bn}, CHH_{Bn}), 4.70-4.67 (m, 2H, CHH_{Bn}, CHH_{Bn}), 4.41 (d, J = 8.0 Hz, 1H, H-1), 3.90-3.87 (m, 1H, H-3'), 3.85 (dd, $J_{6b\text{-}6a}$ = 11.5 Hz, $J_{6b\text{-}5}$ = 3.5 Hz, 1H, H-6b), 3.81 (dd, $J_{6a\text{-}6b}$ = 11.4 Hz, $J_{6a\text{-}5}$ = 1.3 Hz, 1H, H-6a), 3.70 (t, J = 9.3 Hz, 1H, H-4), 3.63 (t, J = 9.3 Hz, 1H, H-3), 3.28-3.26 (m, 1H, H-5), 2.89 (dd, $J_{2a'\text{-}2b'}$ = 15.4 Hz, $J_{2a'\text{-}3'}$ = 4.1 Hz, 1H, H-2a'), 2.71 (dt, $J_{Ha\text{-}Hb}$ = 18.1 Hz, $J_{Ha\text{-}CH2}$ = 7.1 Hz, 1H, CHH_{Lev}), 2.66-2.59 (m, 2H, H-2a'', CHH_{Lev}), 2.56-2.51 (m, 1H, H-2b''), 2.51-2.44 (m, 2H, CH_{2Lev}), 2.42-2.37 (m, 1H, H-2b'), 2.15 (s, 3H, CH_{3Lev}), 0.89-0.85 (m, 15H, $C(CH_3)_{3TBS}$, H-10', H-10''), 0.05 (s, 3H, CH_{3TBS}), 0.03 (s, 3H, CH_{3TBS}); ^{13}C NMR (150 MHz, $CDCl_3$) δ (ppm) 206.2 (CO_{Lev}), 171.5, 170.8, 170.2 (3C,

COOR$_{Lev}$, C-1’, C-1’’), 138.5 (C_{Bn}), 138.4 (C_{Bn}), 135.8 (C_{Bn}), 128.7-127.8 (15C, 15 x CH$_{Bn}$), 101.8 (C-1), 82.9 (C-3), 77.8, 77.6 (2C, C-4, C-3’), 76.0 (C-5), 75.22 (CH$_{2Bn}$), 75.16 (CH$_{2Bn}$), 74.0 (C-2), 70.7 (C-3’’), 66.6 (CH$_{2COOBn}$), 62.1 (C-6), 41.4 (C-2’), 39.3 (C-2’’), 38.0 (CH$_{2Lev}$), 34.8-22.8 (13C, CH$_{3Lev}$, 12 x CH$_2$), 28.1 (CH$_{2Lev}$), 26.1 (3C, C(CH$_3$)$_{3TBS}$), 18.5 (C(CH$_3$)$_{3TBS}$), 14.2 (2C, C-10’, C-10’’), -4.9 (CH$_{3TBS}$), -5.3 (CH$_{3TBS}$); HRMS (ESI-TOF) *m/z* $[M + NH_4]^+$ calcd for $C_{58}H_{90}NO_{12}Si$ 1020.6227; found 1020.6229; *m/z* $[M + Na]^+$ calcd for $C_{58}H_{86}NaO_{12}Si$ 1025.5781; found 1025.5783.

Benzyl (*R*)-3-*O*-[(*R*)-(3'-*O*-Decyl)-3,4-di-*O*-benzyl-2-*O*-levulinoyl-*β*-D-glucopyranosyl]decanoate (18).

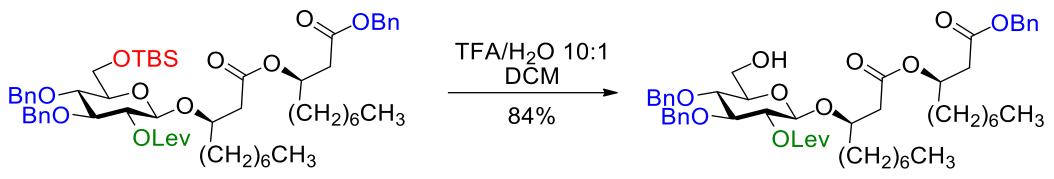

To a solution of glucolipid **8** (172 mg, 0.172 mmol, 1.0 equiv) in DCM (0.4 mL) was added a TFA/H_2O solution (10:1 *v/v*, 0.09 mL). The reaction mixture was stirred at rt for 40 min and then quenched by adding saturated aqueous $NaHCO_3$. The organic layer was dried over $MgSO_4$, filtered, and the solvents were evaporated under reduced pressure. The residue was purified by silica gel flash chromatography (Hex/EtOAc 9:1 to 8:2) to give alcohol **18** (129 mg, 84%) as a white amorphous solid: R_f 0.28 (Hex/EtOAc 7:3); $[\alpha]^{20}_D$ –12 (*c* 1.1, $CHCl_3$); ^{1}H NMR (600 MHz, $CDCl_3$) δ (ppm) 7.37-7.25 (m, 15H, 15 × CH_{Bn}), 5.24-5.18 (m, 1H, H-3''), 5.13 (s, 2H, CH_{2COOBn}), 4.91 (dd, $J_{2\text{-}3}$ = 9.5 Hz, $J_{2\text{-}1}$ = 8.1 Hz, 1H, H-2), 4.77 (d, *J* = 11.3 Hz, 1H, CHH_{Bn}), 4.75 (d, *J* = 10.8 Hz, 1H, CHH_{Bn}), 4.69 (d, *J* = 11.4 Hz, 1H, CHH_{Bn}), 4.54 (d, *J* = 10.9 Hz, 1H, CHH_{Bn}), 4.41 (d, *J* = 8.0 Hz, 1H, H-1), 3.99-3.95 (m, 1H, H-3'), 3.84 (dd, $J_{6a\text{-}6b}$ = 11.5 Hz, $J_{6a\text{-}5}$ = 3.5 Hz, 1H, H-6a), 3.67-3.62 (m, 2H, H-3, H-6b), 3.50 (t, *J* = 9.3 Hz, 1H, H-4), 3.40-3.37 (m, 1H, H-5), 2.94 (br s, 1H, O*H*), 2.72-2.56 (m, 5H, CH_{2Lev}, H-2a'', H-2b'', H-2a'), 2.49-2.46 (m, 2H, CH_{2Lev}), 2.36 (dd, $J_{2b'\text{-}2a'}$ = 15.3 Hz, $J_{2b'\text{-}3'}$ = 5.0 Hz, 1H, H-2b'), 2.15 (s, 3H, CH_{3Lev}), 1.62-1.24 (m, 24H, 12 × CH_2), 0.89-0.86 (m, 6H, 2 x CH_3); ^{13}C NMR (150 MHz, $CDCl_3$) δ (ppm) 206.1 (CO_{Lev}), 171.5, 171.4, 170.4 (3C, $COOR_{Lev}$, C-1', C-1''), 138.4 (C_{Bn}), 137.9 (C_{Bn}), 135.9 (C_{Bn}), 128.7-127.8 (15C, 15 × CH_{Bn}), 101.1 (C-1), 83.0 (C-3), 78.4 (C-4), 77.8 (C-3'), 75.7 (C-5), 75.2 (2C, 2 x CH_{2Bn}), 73.9 (C-2), 71.1 (C-3''), 66.7 (CH_{2COOBn}), 62.3 (C-6), 41.4 (C-2'), 39.1 (C-2''), 37.0 (CH_{2Lev}), 35.6-22.8 (13C, 12 × CH_2, CH_{3Lev}), 28.0 (CH_{2Lev}), 14.2 (2C, 2 x CH_3); HRMS (ESI-TOF) *m/z* $[M + NH_4]^+$

calcd for $C_{52}H_{76}NO_{12}$ 906.5362; found 906.5365; *m/z* $[M + Na]^+$ calcd for $C_{52}H_{72}NaO_{12}$ 911.4916; found 911.4929.

Benzyl (*R*)-3-*O*-[(*R*)-(3'-*O*-Decyl)-3,4-di-*O*-benzyl-*β*-D-glucopyranosyl]decanoate (19).

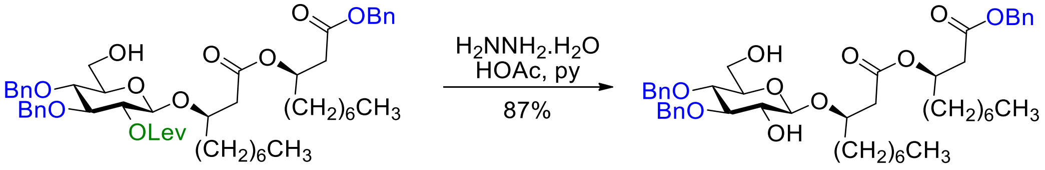

18 **19**

Hydrazine monohydrate (32 µL, 5.0 equiv) and HOAc (0.55 mL) were successively added to a solution of alcohol **18** (117 mg, 0.131 mmol, 1.0 equiv) in anhydrous pyridine (0.85 mL) at 0 °C. The mixture was stirred at rt for 16 h under an Ar atmosphere, then co-evaporated with toluene. The residue was purified by silica gel flash chromatography (Tol/EtOAc 95:5 to 9:1) to give diol **19** (91 mg, 87%) as a white amorphous solid: R_f 0.54 (Tol/EtOAc 8:2); $[\alpha]^{20}_{D}$ –9 (*c* 0.6, $CHCl_3$); ^{1}H NMR (600 MHz, $CDCl_3$) δ (ppm) 7.37-7.27 (m, 15H, 15 × CH_{Bn}), 5.25-5.20 (m, 1H, H-3''), 5.12 (d, J = 4.7 Hz, 2H, CH_{2COOBn}), 4.93 (d, J = 11.2 Hz, 1H, CHH_{Bn}), 4.83 (d, J = 11.1 Hz, 1H, CHH_{Bn}), 4.81 (d, J = 10.7 Hz, 1H, CHH_{Bn}), 4.56 (d, J = 10.9 Hz, 1H, CHH_{Bn}), 4.32 (d, J = 7.8 Hz, 1H, H-1), 4.08-4.04 (m, 1H, H-3'), 3.84 (dd, $J_{6a\text{-}6b}$ = 11.8 Hz, $J_{6a\text{-}5}$ = 2.0 Hz, 1H, H-6a), 3.63 (dd, $J_{6b\text{-}6a}$ = 11.9 Hz, 1H, H-6b), 3.59 (t, J = 8.9 Hz, 1H, H-3), 3.47-3.42 (m, 2H, H-2, H-4), 3.40-3.37 (m, 1H, H-5), 2.67 (dd, $J_{2a''\text{-}2b''}$ = 15.5 Hz, $J_{2a''\text{-}3''}$ = 7.0 Hz, 1H, H-2a''), 2.61-2.56 (m, 2H, H-2b'', H-2a'), 2.43 (dd, $J_{2b'\text{-}2a'}$ = 15.1 Hz, $J_{2b'\text{-}3'}$ = 4.9 Hz, 1H, H-2b'), 1.56-1.24 (m, 24H, 12 × CH_2), 0.89-0.86 (m, 6H, 2 × CH_3); ^{13}C NMR (150 MHz, $CDCl_3$) δ (ppm) 171.4, 170.4 (2C, C-1', C-1''), 138.8 (C_{Bn}), 138.1 (C_{Bn}), 135.8 (C_{Bn}), 128.7-127.8 (15C, 15 × CH_{Bn}), 102.3 (C-1), 84.6 (C-3), 78.0 (C-4), 75.7 (C-3'), 75.3 (C-5), 75.2, 75.1 (3C, C-2, 2 × CH_{2Bn}), 71.1 (C-3''), 66.7 (CH_{3COOBn}), 62.4 (C-6), 41.3 (C-2'), 39.2 (C-2''), 35.3-22.8 (12C, 12 × CH_2), 14.2 (2C, 2 × CH_3); HRMS (ESI-TOF) *m/z* $[M + NH_4]^+$ calcd for $C_{47}H_{70}NO_{10}$ 808.4994; found 808.4967; *m/z* $[M + Na]^+$ calcd for $C_{47}H_{66}NaO_{10}$ 813.4548; found 813.4532.

Synthetic Ananatoside B (2).

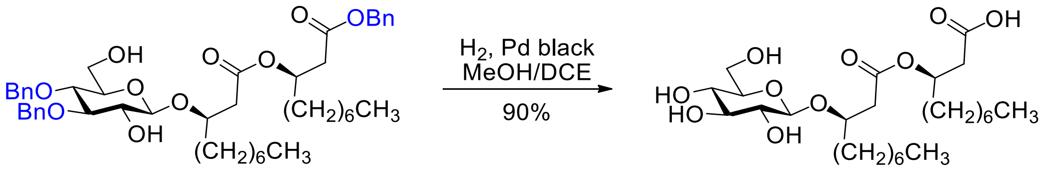

19 2

Pd black (78 mg, 1 mg•mg^{-1} of diol **19**) was added to a solution of diol **19** (78 mg, 0.099 mmol, 1.0 equiv) in DCE (1 mL) and MeOH (2 mL). The mixture was stirred under an H_2 atmosphere at 40 °C for 16 h, after which it was filtered over Celite and the solvents were evaporated under reduced pressure. The residue was purified by silica gel flash chromatography (DCM/MeOH 95:5 to 8:2) to give synthetic ananatoside B (**2**, 46 mg, 90%) as a white foam. R_f 0.50 (DCM/MeOH 8:2); $[\alpha]^{20}_{D}$ +103 (*c* 0.2, $CHCl_3$). Physical and analytical data of synthetic ananatoside B (**2**) agreed with those of the isolated compound. Analytical HPLC analysis was performed using method D (23.3 min.).

***para*-Methylphenyl 2-*O*-*ortho*-(Azidomethyl)benzoyl-3,4-di-*O*-benzyl-6-*O*-*tert*-butyldimethylsilyl-1-thio-*β*-D-glucopyranoside (S11).**

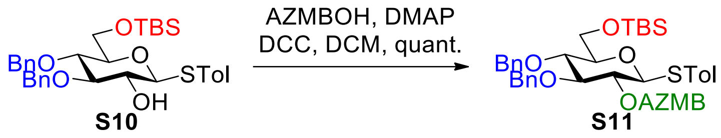

To a solution of alcohol **S10** (889 mg, 1.53 mmol, 1.0 equiv) in anhydrous DCM (15.3 mL) were added DMAP (187 mg, 1.53 mmol, 1.0 equiv), DCC (632 mg, 3.06 mmol, 2.0 equiv), and AZMBOH (407 mg, 2.30 mmol, 1.5 equiv). The mixture was refluxed for 4 h, after which the suspension was cooled at 0 °C and filtered over Celite. The solvents were evaporated under reduced pressure and the residue was purified by silica gel flash chromatography (Hex/EtOAc 95:5) to give fully protected **S11** (1.14 g, quant.) as a colorless oil: R_f 0.46 (Hex/EtOAc 8:2); $[\alpha]^{20}_{D}$ +5 (*c* 0.8, $CHCl_3$); ^{1}H NMR (600 MHz, $CDCl_3$) δ (ppm) 7.94-7.92 (m, 1H, C*H*-AZMB), 7.59-7.57 (m, 1H, C*H*-AZMB), 7.54-7.53 (m, 1H, C*H*-Ar), 7.40-7.28 (m, 8H, 8 × C*H*-Ar), 7.13-7.11 (m, 5H, 5 × C*H*-Ar), 7.07-7.05 (m, 2H, 2 × C*H*-Ar), 5.16 (dd, *J* = 9.8 Hz, *J* = 9.0 Hz, 1H, H-2), 4.84-4.77 (m, 3H, C*H*H_{AZMB}, CH_{2Bn}), 4.72-4.66 (m, 3H, H-1, CHH_{AZMB}, C*H*H_{Bn}), 4.60 (d, *J* = 11.2 Hz, 1H, CHH_{Bn}), 3.93 (dd, $J_{6a\text{-}6b}$ = 11.4 Hz, $J_{6a\text{-}5}$ = 1.7 Hz, H-6a), 3.89 (dd, $J_{6b\text{-}6a}$ = 11.4 Hz, $J_{6b\text{-}5}$ = 3.8 Hz, 1H, H-6b), 3.81-3.76 (m, 2H, H-3, H-4), 3.41 (ddd, $J_{5\text{-}4}$ = 9.3 Hz, $J_{5\text{-}6b}$ = 3.5 Hz, $J_{5\text{-}6a}$ = 1.6 Hz, 1H, H-5), 2.32 (s, 3H, CH_{3STol}), 0.83 (s, 9H, C(CH_3)$_{3TBS}$), 0.13 (s, 3H, CH_{3TBS}), 0.10 (s, 3H, CH_{3TBS}); ^{13}C NMR (150 MHz, $CDCl_3$) δ (ppm) 165.2 (*C*OOR_{AZMB}), 138.29 (*C*-Ar), 138.25 (*C*-Ar), 138.0 (*C*-Ar), 137.9 (*C*-Ar), 133.5-127.8 (20C, 2 × *C*-Ar, 18 × *C*H-Ar), 86.3 (C-1), 84.5 (C-3), 80.6 (C-5), 77.7 (C-4), 75.5 (*C*H_{2Bn}), 75.2 (*C*H_{2Bn}), 72.5 (C-2), 62.2 (C-6), 53.0 (*C*H_{2AZMB}), 26.1 (3C, C(*C*H_3)$_{3TBS}$), 21.3 (*C*H_{3STol}), 18.5 (*C*(CH$_3$)$_{3TBS}$), -4.93 (*C*H_{3TBS}), -5.21 (*C*H_{3TBS}); HRMS (ESI-TOF) *m/z* $[M + Na]^+$ calcd for $C_{41}H_{49}NaN_3O_6SSi$ 762.3004; found 762.3001; *m/z* $[M + K]^+$ calcd for $C_{41}H_{49}N_3O_6KSSi$ 778.2743; found 778.2779.

***para*-Methylphenyl 2-*O*-*ortho*-(Azidomethyl)benzoyl-3,4-di-*O*-benzyl-1-thio-*β*-D-glucopyranoside (9).**

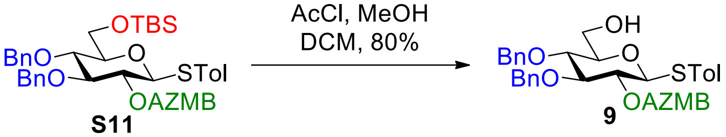

AcCl (1.5 μL, 0.021 mmol, 0.30 equiv) was added to a solution of derivative **S11** (52 mg, 0.071 mmol, 1.0 equiv) in anhydrous MeOH (1.42 mL) and anhydrous DCM (0.5 mL) at 0 °C. The mixture was slowly heated to rt over 3.5 h under Ar, then diluted in DCM (2 mL) and washed with saturated aqueous $NaHCO_3$ (5 mL) and brine (5 mL). The organic layers were dried over $MgSO_4$, filtered, and evaporated under reduced pressure. The residue was purified by silica gel flash chromatography (Hex/EtOAc 8:2 to 7:3) to give alcohol **9** (35.4 mg, 80%) as a white amorphous solid: R_f 0.26 (Hex/EtOAc 7:3); $[\alpha]^{20}_{D}$ +26 (*c* 0.5, $CHCl_3$); ^{1}H NMR (600 MHz, $CDCl_3$) δ (ppm) 7.93-7.92 (m, 1H, C*H*-AZMB), 7.59-7.58 (m, 1H, C*H*-AZMB), 7.55-7.54 (m, 1H, C*H*-Ar), 7.40-7.37 (m, 1H, C*H*-Ar), 7.35-7.28 (m, 7H, 7 × C*H*-Ar), 7.15-7.12 (m, 5H, 5 × C*H*-Ar), 7.11-7.09 (m, 2H, 2 × C*H*-Ar), 5.20 (t, J = 9.6 Hz, 1H, H-2), 4.85-4.81 (m, 2H, C*H*H_{Bn}, C*H*H_{AZMB}), 4.78 (d, J = 9.6 Hz, 1H, C*H*H_{Bn}), 4.75 (d, J = 10.0 Hz, 1H, H-1), 4.70 (d, J = 14.9 Hz, 1H, CHH_{AZMB}), 4.66 (d, J = 11.0 Hz, 1H, CHH_{Bn}), 4.63 (d, J = 11.2 Hz, 1H, CHH_{Bn}), 3.92 (ddd, $J_{6a\text{-}6b}$ = 11.9 Hz, J = 2.7 Hz, J = 2.3 Hz, 1H, H-6a), 3.83 (t, J = 9.1 Hz, 1H, H-3), 3.75-3.71 (m, 1H, H-6b), 3.68 (t, J = 9.4 Hz, 1H, H-4), 3.48 (ddd, $J_{5\text{-}4}$ = 9.7 Hz, $J_{5\text{-}6a}$ = 4.6 Hz, $J_{5\text{-}6b}$ = 2.6 Hz, 1H, H-5), 2.33 (s, 3H, CH_{3STol}), 1.91 (t, J = 6.5 Hz, 1H, OH); ^{13}C NMR (150 MHz, $CDCl_3$) δ (ppm) 165.2 (*C*OOR_{AZMB}), 138.7 (*C*-Ar), 138.0 (*C*-Ar), 137.8 (2C, 2 × *C*-Ar), 133.4-127.9 (20C, 2 × *C*-Ar, 18 × *C*H-Ar), 86.3 (C-1), 84.2 (C-3), 79.7 (C-5), 77.72 (C-4), 75.4 (*C*H_{2Bn}), 75.3 (*C*H_{2Bn}), 72.5 (C-2), 62.1 (C-6), 53.0 (*C*H_{2AZMB}), 21.3 (*C*H_{3STol}); HRMS (ESI-TOF) *m/z* $[M + NH_4]^+$ calcd for $C_{35}H_{39}N_4O_6S$ 643.2585; found 643.2592; *m/z* $[M + Na]^+$ calcd for $C_{35}H_{35}NaN_3O_6S$ 648.2139; found 648.2146.

***para*-Methylphenyl 2-*O*-*ortho*-(Azidomethyl)benzoyl-3,4-di-*O*-benzyl-6-*O*-(*R*)-3-(((*R*)-3-(((*tert*-butyldimethylsilyl)oxy)decanoyl)oxy)decanoyl-1-thio-*β*-D-glucopyranoside (7).**

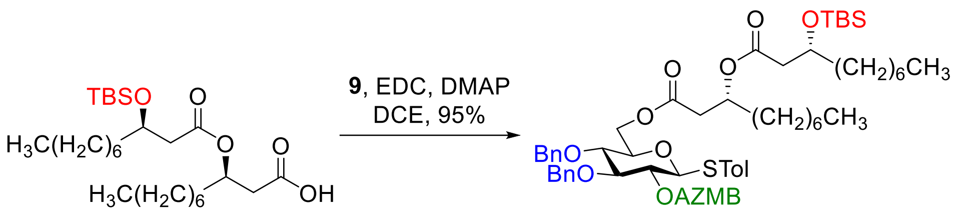

To a solution of alcohol **9** (115 mg, 0.184 mmol, 1.0 equiv) and dilipid **10** (104 mg, 0.221 mmol, 1.2 equiv) in anhydrous DCE (2.2 mL) were successively added DMAP (7 mg, 0.06 mmol, 0.3 equiv) and EDC (106 mg, 0.551 mmol, 3.0 equiv). The mixture was stirred at rt for 16 h under Ar atmosphere. The solution was then evaporated under reduced pressure and the residue was purified by silica gel flash chromatography (Hex/EtOAc 95:5 to 90:10) to give compound **7** (188 mg, 95%) as a colorless oil: R_f 0.5 (Hex/EtOAc 8:2); $[\alpha]^{20}_D$ +8 (*c* 0.4, $CHCl_3$); ^{1}H NMR (600 MHz, $CDCl_3$) δ (ppm) 7.94-7.93 (m, 1H, CH_{AZMB}), 7.60-7.58 (m, 1H, CH_{AZMB}), 7.55-7.54 (m, 1H, CH_{AZMB}), 7.41-7.38 (m, 1H, CH_{AZMB}), 7.35-7.27 (m, 7H, 5 × CH_{Bn}, 2 × CH_{STol}), 7.15-7.12 (m, 5H 5 × CH_{Bn}), 7.09-7.08 (m, 2H, 2 × CH_{STol}), 5.24-5.20 (m, 1H, H-3''), 5.19 (t, *J* = 9.4 Hz, 1H, H-2), 4.84-4.81 (m, 2H, CHH_{AZMB}, CHH_{Bn}), 4.76 (d, *J* = 11.1 Hz, 1H, CHH_{Bn}), 4.70-4.68 (m, 2H, H-1, CHH_{AZMB}), 4.62 (d, *J* = 11.4 Hz, CHH_{Bn}), 4.60 (d, *J* = 11.3 Hz, 1H, CHH_{Bn}), 4.47 (d, *J* = 11.6 Hz, 1H, H-6a), 4.23-4.21 (m, 1H, H-6b), 4.08 (p, *J* = 6.1 Hz, 1H, H-3'), 3.83-3.80 (m, 1H, H-3), 3.63-3.59 (m, 2H, H-4, H-5), 2.66 (dd, $J_{2a''\text{-}2b''}$ = 15.7 Hz, $J_{2a''\text{-}3''}$ = 7.0 Hz, 1H, H-2a''), 2.52 (dd, $J_{2b''\text{-}2a''}$ = 15.7 Hz, $J_{2b''\text{-}3''}$ = 6.0 Hz, 1H, H-2b''), 2.45 (dd, $J_{2a'\text{-}2b'}$ = 14.8 Hz, $J_{2a'\text{-}3'}$ = 5.9 Hz, 1H, H-2a'), 2.41 (dd, $J_{2b'\text{-}2a'}$ = 14.9 Hz, $J_{2b'\text{-}3'}$ = 6.7 Hz, 1H, H-2b'), 2.33 (s, 3H, CH_{3STol}), 1.64-1.22 (m, 24H, 24 × CH_2), 0.88-0.85 (m, 15H, $C(CH_3)_{3TBS}$, 2 × CH_3), 0.06 (s, 3H, CH_{3TBS}), 0.05 (s, 3H, CH_{3TBS}); ^{13}C NMR (150 MHz, $CDCl_3$) δ (ppm) 171.1, 170.1 (2C, C-1', C-1''), 165.1 ($COOR_{AZMB}$), 138.5-128.0 (24C,

6 × C_{Ar}, 18 × CH_{Ar}), 86.2 (C-1), 84.5 (C-3), 77.8, 77.2 (2C, C-4, C-5), 75.5 (CH_{2Bn}), 75.3 (CH_{2Bn}), 72.3 (C-2), 70.5 (C-3’’), 69.4 (C-3’), 63.2 (C-6), 53.0 (CH_{2AZMB}), 42.9 (C-2’), 39.0 (C-2’’), 37.5-22.8 (15C, 12 × CH_2, C(CH_3)$_{3TBS}$), 21.3 (CH_{3STol}), 18.2 (C(CH_3)$_{3TBS}$), 14.25 (CH_3), 14.23 (CH_3), -4.42 (CH_{3TBS}), -4.45 (CH_{3TBS}); HRMS (ESI-TOF) *m/z* $[M + NH_4]^+$ calcd for $C_{61}H_{89}N_4O_{10}SSi$ 1097.6063; found 1097.6038; *m/z* $[M + Na]^+$ calcd for $C_{61}H_{85}NaN_3O_{10}SSi$ 1102.5616; found 1102.5600.

***para*-Methylphenyl 2-*O*-*ortho*-(Azidomethyl)benzoyl-3,4-di-*O*-benzyl-6-*O*-(*R*)-3-(((*R*)-3-(hydroxydecanoyl)oxy)decanoyl-1-thio-*β*-D-glucopyranoside (20).**

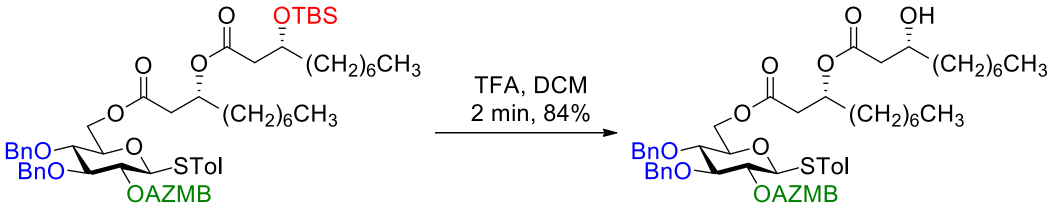

7 20

A solution of compound **7** (450 mg, 0.416 mmol, 1.0 equiv) in DCM (0.9 mL) was added dropwise to TFA (1.8 mL) over a one-minute period. The mixture was stirred for one additional minute and quenched with saturated aqueous $NaHCO_3$. The organic layer was dried over $MgSO_4$, filtered, and the solvents were evaporated under reduced pressure. The residue was purified by silica gel flash chromatography (Hex/EtOAc 9:1 to 8:2) to give alcohol **20** (338 mg, 84%) as a white amorphous solid: R_f 0.37 (Hex/EtOAc 7:3); $[\alpha]^{20}_D$ +9 (*c* 0.4, $CHCl_3$); ^{1}H NMR (600 MHz, $CDCl_3$) *δ* (ppm) 7.94-7.93 (m, 1H, 1 CH_{AZMB}), 7.60-7.58 (m, 1H, CH_{AZMB}), 7.55-7.54 (m, 1H, CH_{AZMB}), 7.40-7.38 (m, 1H, CH_{AZMB}), 7.35-7.26 (m, 7H, 5 × CH_{Bn}, 2 × CH_{STol}), 7.15-7.11 (m, 5H, 5 × CH_{Bn}), 7.09-7.08 (m, 2H, 2 × CH_{STol}), 5.31-5.27 (m, 1H, H-3’’), 5.19 (t, *J* = 9.6 Hz, 1H, H-2), 4.84-4.81 (m, 2H, C*H*H_{AZMB}, C*H*H_{Bn}), 4.76 (d, *J* = 11.1 Hz, 1H, CHH_{Bn}), 4.71 (d, *J* = 8.4 Hz, 1H, H-1), 4.69 (d, *J* = 13.1 Hz, 1H, CHH_{AZMB}), 4.62 (d, *J* = 9.2 Hz, 1H, CHH_{Bn}), 4.60 (d, *J* = 8.9 Hz, 1H, CHH_{Bn}), 4.47 (d, $J_{6a\text{-}6b}$ = 11.5 Hz, 1H, H-6a), 4.21 (dd, $J_{6b\text{-}6a}$ = 11.7 Hz, $J_{6b\text{-}5}$ = 4.1 Hz, 1H, H-6b), 3.97 (br s, 1H, H-3’), 3.84-3.81 (m, 1H, H-3), 3.64-3.61 (m, 2H, H-4, H-5), 2.93 (s, 1H, O*H*), 2.62 (dd, $J_{2a''\text{-}2b''}$ = 15.5 Hz, $J_{2a''\text{-}3''}$ = 8.1 Hz, 1H, H-2a’’), 2.55 (dd, $J_{2b''\text{-}2a''}$ = 15.6 Hz, $J_{2b''\text{-}3''}$ = 4.6 Hz, 1H, H-2b’’), 2.45 (dd, $J_{2a'\text{-}2b'}$ = 15.8 Hz, $J_{2a'\text{-}3'}$ = 3.0 Hz, 1H, H-2a’), 2.37 (dd, $J_{2b'\text{-}2a'}$ = 15.8 Hz, $J_{2b'\text{-}3'}$ = 9.2 Hz, 1H, H-2b’), 2.33 (s, 3H, CH_{3STol}), 1.67-1.22 (m, 24H, 12 x CH_2), 0.88-0.86 (m, 6H, 2 x CH_3); ^{13}C NMR (150 MHz, $CDCl_3$) *δ* (ppm) 172.6, 170.4 (2C, C-1’, C-1’’), 165.1 (*C*OOR_{AZMB}),

138.6-128.0 (24C, 6 × C_{Ar}, 18 × CH_{Ar}), 86.2 (C-1), 84.5 (C-3), 77.4 (2C, C-4, C-5), 75.6 (CH_{2Bn}), 75.3 (CH_{2Bn}), 72.3 (C-2), 70.8 (C-3’’), 68.5 (C-3’), 63.3 (C-6), 53.0 (CH_{2AZMB}), 42.0 (C-2’), 39.1 (C-2’’), 36.8-22.8 (12C, 12 × CH_2), 21.3 (CH_{3STol}), 14.2 (2C, 2 × CH_3); HRMS (ESI-TOF) *m/z* [M + NH_4]$^+$ calcd for $C_{55}H_{75}N_4O_{10}S$ 983.5198; found 983.5237; *m/z* [M + Na]$^+$ calcd for $C_{55}H_{71}NaN_3O_{10}S$ 988.4752; found 988.4781.

Macrolide 21.

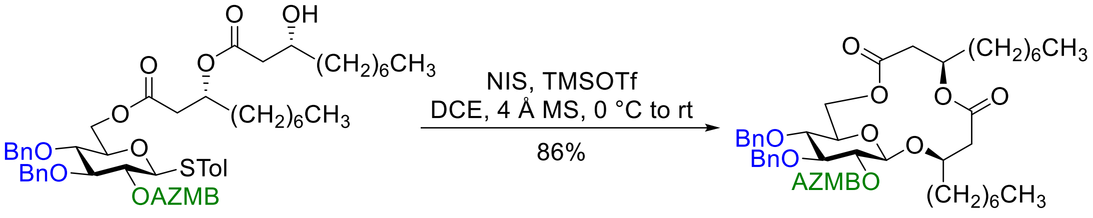

Compound **20** (71 mg, 0.073 mmol, 1.0 equiv) and NIS (28 mg, 0.12 mmol, 1.6 equiv) were dried together under high vacuum for 1 h. Activated 4 Å MS (300 mg, 4 mg•mg^{-1} of substrate) and anhydrous DCE (7.8 mL) were subsequently added to the reaction flask and the mixture was stirred at rt for 1 h under an Ar atmosphere, after which TMSOTf (3 µL, 0.003 mmol, 0.2 equiv) was added at 0 °C. The reaction was stirred for an additional 30 min at 0 °C, then quenched with Et_3N. The suspension was filtered over Celite and the solvents were evaporated under reduce pressure. The residue was purified by silica gel flash chromatography (Hex/EtOAc 95:5 to 9:1) to give macrolactone **21** (54 mg, 86%) as a white amorphous solid: R_f 0.43 (Hex/EtOAc 8:2); $[\alpha]^{20}_D$ +21 (*c* 0.2, $CHCl_3$); ^{1}H NMR (600 MHz, $CDCl_3$) δ (ppm) 7.96-7.95 (m, 1H, CH_{AZMB}), 7.59-7.54 (m, 2H, 2 × CH_{Ar}), 7.39-7.27 (m, 6H, 6 × CH_{Ar}), 7.12-7.10 (m, 5H, 5 × CH_{Ar}), 5.55-5.50 (m, 1H, H-3’’), 5.16 (dd, $J_{2\text{-}3}$ = 9.6 Hz, $J_{2\text{-}1}$ = 8.4 Hz, 1H, H-2), 4.87 (d, J = 11.2 Hz, 1H, CHH_{Bn}), 4.81 (d, J = 15.2 Hz, 1H, CHH_{AZMB}), 4.77-4.73 (m, 3H, H-1, CHH_{Bn}, CHH_{AZMB}), 4.62-4.56 (m, 2H, CHH_{Bn}, CHH_{Bn}), 4.34 (t, J = 10.8 Hz, 1H, H-6a), 4.14 (td, J = 9.4 Hz, J = 3.6 Hz, 1H, H-3’), 4.08 (dd, $J_{6b\text{-}6a}$ = 11.3 Hz, $J_{6b\text{-}5}$ = 1.7 Hz, 1H, H-6b), 3.80 (t, J = 9.2 Hz, 1H, H-3), 3.60 (td, $J_{5\text{-}4}$ = 10.0 Hz, $J_{5\text{-}6b}$ = 1.7 Hz, 1H, H-5), 3.38 (t, J = 9.6 Hz, 1H, H-4), 2.67 (dd, $J_{2a'\text{-}2b'}$ = 18.5 Hz, $J_{2a'\text{-}3'}$ = 8.7 Hz, 1H, H-2a’), 2.60-2.56 (m, 2H, H-2’’), 2.30 (d, $J_{2b'\text{-}2a'}$ = 18.4 Hz, 1H, H-2b’), 1.53-0.94 (m, 24H, 12 × CH_2), 0.87 (t, J = 7.0 Hz, 3H, CH_3), 0.77 (t, J = 7.3 Hz, 3H, CH_3); ^{13}C NMR (150 MHz, $CDCl_3$) δ (ppm) 171.6, 169.6 (C-1’, C-1’’), 164.9 ($COOR_{AZMB}$), 138.5 (C_{Ar}), 137.7 (C_{Ar}), 137.6 (C_{Ar}), 133.1-

127.9 (15C, C_{Ar}, 14 × $\mathit{C}H_{Ar}$), 101.8 (C-1), 82.8 (C-3), 79.0 (C-4), 77.9 (C-3’), 75.3 ($\mathit{C}H_{2Bn}$), 75.1 ($\mathit{C}H_{2Bn}$), 73.8 (C-2), 72.6 (C-5), 69.7 (C-3’’), 63.9 (C-6), 53.2 ($\mathit{C}H_{2AZMB}$), 41.7 (C-2’), 40.5 (C-2’’), 36.5-22.7 (12C, 12 × $\mathit{C}H_2$), 14.2, 14.1 (2C, C-10’, C-10’’); HRMS (ESI-TOF) *m/z* $[M + NH_4]^+$ calcd for $C_{48}H_{67}N_4O_{10}$ 859.4852; found 859.4877; *m/z* $[M + Na]^+$ calcd for $C_{48}H_{63}NaN_3O_{10}$ 864.4406; found 864.4434.

(*R*)-3-*O*-[(*R*)-(3'-*O*-Decyl)-3,4-di-*O*-benzyl-2-*O*-levulinoyl-*β*-D-glucopyranosyl] Decanoic acid (22).

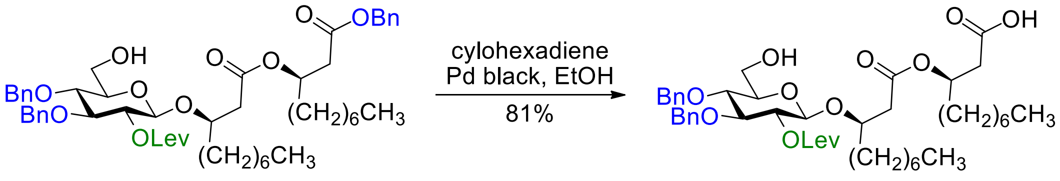

Glucolipid **18** (153 mg, 0.172 mmol, 1.0 equiv) was solubilized in absolute EtOH (3.4 mL) and one drop of anhydrous DCM was added. To this solution were added 1,4-cyclohexadiene (0.14 mL, 1.7 mmol, 10 equiv) and Pd black (153 mg, 1 mg•mg^{-1} of glucolipid **18**), and the mixture was stirred at rt for 4 h under an Ar atmosphere. The mixture was filtered over Celite and the solvents were evaporated under reduced pressure. The residue was purified by silica gel flash chromatography (DCM/MeOH 98:2 to 9:1) to give acid **22** (112 mg, 81%) as a white amorphous solid: R_f 0.36 (DCM/MeOH 95:5); $[\alpha]^{20}_{D}$ –16 (*c* 0.6, $CHCl_3$); ^{1}H NMR (600 MHz, $CDCl_3$) δ (ppm) 7.34-7.26 (m, 10H, 10 × CH_{Bn}), 5.25-5.21 (m, 1H, H-3''), 4.96 (t, *J* = 8.5 Hz, 1H, H-2), 4.79 (d, *J* = 11.0 Hz, 1H, C*H*H_{Bn}), 4.78 (d, *J* = 11.4 Hz, 1H, C*H*H_{Bn}), 4.69 (d, *J* = 11.5 Hz, 1H, CHH_{Bn}), 4.63 (d, *J* = 10.9 Hz, 1H, CHH_{Bn}), 4.42 (d, *J* = 7.9 Hz, 1H, H-1), 4.04-4.00 (m, 1H, H-3'), 3.89 (dd, $J_{6a\text{-}6b}$ = 12.1 Hz, $J_{6a\text{-}5}$ = 1.9 Hz, 1H, H-6a), 3.71 (dd, $J_{6b\text{-}6a}$ = 11.8 Hz, $J_{6b\text{-}5}$ = 5.1 Hz, 1H, H-6b), 3.68-3.63 (m, 2H, H-3, H-4), 3.40-3.36 (m, 1H, H-5), 2.72-2.61 (m, 2H, CH_{2Lev}), 2.59-2.49 (m, 3H, H-2a', H-2a'', H-2b''), 2.48-2.46 (m, 2H, CH_{2Lev}), 2.41 (dd, 1H, $J_{2b'\text{-}2a'}$ = 15.3 Hz, $J_{2b'\text{-}3'}$ = 4.3 Hz, 1H, H-2b'), 2.15 (s, 3H, CH_{3Lev}), 1.62-1.21 (m, 24H, 12 × CH_2), 0.89-0.86 (m, 6H, 2 × CH_3); ^{13}C NMR (150 MHz, $CDCl_3$) δ (ppm) 206.2 (CO_{Lev}), 172.1, 171.8 (3C, $COOR_{Lev}$, C-1', C-1''), 138.3 (C_{Bn}), 138.0 (C_{Bn}), 128.6-127.9 (10C, 10 × CH_{Bn}), 100.2 (C-1), 82.8 (C-3), 78.0 (C-4), 77.7 (C-3'), 75.6 (C-5), 75.2 (CH_{2Bn}), 75.1 (CH_{2Bn}), 73.9 (C-2), 71.7 (C-3''), 61.8 (C-6), 41.0 (2C, C-2' C-2''), 37.9 (CH_{2Lev}), 35.2-22.8 (14C, CH_{2Lev}, CH_{3Lev}, 12 × CH_2), 14.2 (2C, 2 × CH_3); HRMS (ESI-TOF)

m/z [M + NH_4]$^+$ calcd for $C_{45}H_{70}NO_{12}$ 816.4893; found 816.4907; *m/z* [M + Na]$^+$ calcd for $C_{45}H_{66}NaO_{12}$ 821.4447; found 821.4468.

Macrolide 23.

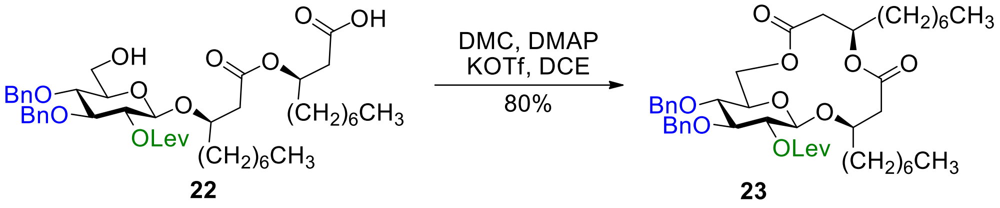

To a cooled solution of seco acid **22** (7 mg, 0.009 mmol, 1.0 equiv) in anhydrous DCE (0.5 mL) were added DMC (4 mg, 0.02 mmol, 2.5 equiv) and KOTf (3 mg, 0.02 mmol, 2.0 equiv). The mixture was stirred at 0 °C for 1 h under Ar atmosphere. DMAP (54 mg, 0.44 mmol, 50 equiv) was added and the mixture was stirred for 3h30 while allowing the solution to gradually reach rt. The solvents were evaporated under reduced pressure. The residue was purified by silica gel flash chromatography (Hex/EtOAc 95:5 to 8:2) to give macrolactone **23** (5.4 mg, 80%) as a white amorphous solid: R_f 0.59 (Hex/EtOAc 7:3); $[\alpha]^{20}_{D}$ +11 (*c* 0.5, $CHCl_3$); ^{1}H NMR (600 MHz, $CDCl_3$) δ (ppm) 7.34-7.26 (m, 8H, 8 × CH_{Bn}), 7.25-7.24 (m, 2H, 2 × CH_{Bn}), 5.52-5.47 (m, 1H, H-3''), 4.91 (dd, $J_{2\text{-}3}$ = 9.6 Hz, $J_{2\text{-}1}$ = 8.3 Hz, 1H, H-2), 4.83 (d, J = 11.1 Hz, 1H, CHH$_{Bn}$), 4.75 (d, J = 11.3 Hz, CHH_{Bn}), 4.70 (d, J = 11.3 Hz, 1H, CHH$_{Bn}$), 4.57 (d, J = 8.2 Hz, 1H, H-1), 4.51 (d, J = 11.2 Hz, 1H, CHH_{Bn}), 4.29 (dd, $J_{6a\text{-}6b}$ = 11.2 Hz, $J_{6a\text{-}5}$ = 10.2 Hz, 1H, H-6a), 4.15-4.11 (m, 1H, H-3'), 4.05 (dd, $J_{6b\text{-}6a}$ = 11.4 Hz, $J_{6b\text{-}5}$ = 1.8 Hz, 1H, H-6b), 3.67 (t, J = 9.2 Hz, 1H, H-3), 3.53 (td, $J_{5\text{-}4,\,5\text{-}6a}$ = 10.0 Hz, $J_{5\text{-}6b}$ = 1.8 Hz, 1H, H-5), 3.29 (t, J = 9.3 Hz, 1H, H-4), 2.75-2.69 (m, 1H, CHH$_{Lev}$), 2.67-2.58 (m, 2H, H-2a', CHH_{Lev}), 2.57-2.45 (m, 4H, CH_{2Lev}, H-2a'', H-2b''), 2.32 (d, $J_{2b'\text{-}2a'}$ = 18.5 Hz, 1H, H-2b'), 2.15 (s, 3H, CH_{3Lev}), 1.54-1.23 (m, 24H, 12 × CH_2), 0.89-0.86 (m, 6H, 2 × CH_3); ^{13}C NMR (150 MHz, $CDCl_3$) δ (ppm) 206.1 (CO_{Lev}), 171.5, 171.4, 169.6 (3C, $COOR_{Lev}$, C-1', C-1''), 138.1 (C_{Bn}), 137.6 (C_{Bn}), 126.7-128.0 (10C, 10 x CH_{Bn}), 101.7 (C-1), 83.0 (C-3), 78.8 (C-4), 77.7 (C-3'), 75.3 (CH_{2Bn}), 75.1 (CH_{2Bn}), 73.7 (C-2), 72.5 (C-5), 69.6 (C-3''), 64.0 (C-6), 41.6 (C-2'), 40.5 (C-2''), 37.9 (CH_{2Lev}), 36.4-22.8 (14C, CH_{2Lev}, CH_{3Lev}, 12 × CH_2), 14.2 (2C, 2 × CH_3); HRMS (ESI-

TOF) *m/z* [M + Na]$^+$ calcd for $C_{45}H_{64}NaO_{11}$ 803.4341; found 803.4337; *m/z* [M + K]$^+$ calcd for $C_{45}H_{64}KO_{11}$ 819.4080; found 819.4077. HRMS data for the corresponding dimer: HRMS (ESI-TOF) *m/z* [M + Na]$^+$ calcd for C_{90}H128NaO_{22} 1583.8789; found 1583.8749.

3,4-Di-*O*-benzylated Macrolide S12.

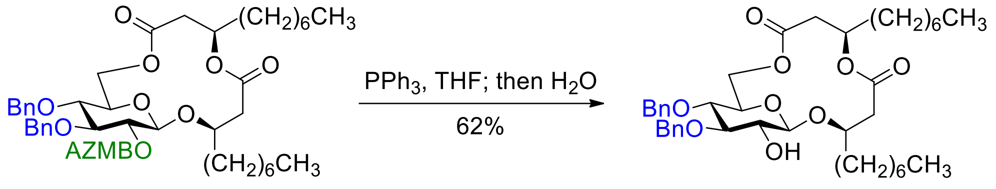

Route A: PPh_3 (24 mg, 0.090 mmol, 1.6 equiv) was added to a solution of macrolactone **21** (48 mg, 0.056 mmol, 1.0 equiv) in anhydrous THF (1.7 mL). The mixture was stirred under an Ar atmosphere at 60 °C for 2 h, after which H_2O (0.2 mL) was added. The solution was stirred at 60 °C for an additional 4 h. The solvents were then evaporated under reduced pressure and co-evaporated with toluene. The residue was purified by silica gel flash chromatography (Tol/EtOAc 98:2 to 95:5) to give alcohol **S12** (24 mg, 62%) as a white amorphous solid.

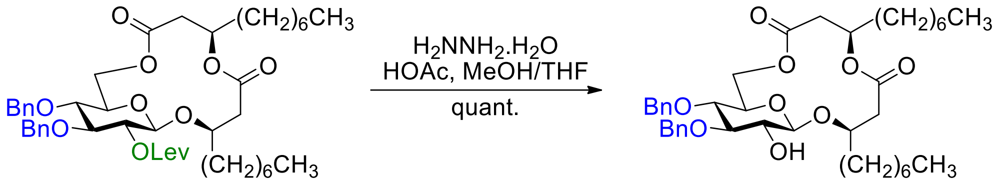

Route B: To a solution of macrolide **23** (20 mg, 0.026 mmol, 1.0 equiv) in anhydrous THF/MeOH (10:1 *v/v*, 1.8 mL) was slowly added a solution of $H_2NNH_2.H_2O$ (18 μL, 0.37 mmol, 14 equiv) and HOAc (45 μL) in anhydrous THF/MeOH (5:1 *v/v*, 0.4 mL). The solution was stirred under an Ar atmosphere for 30 min until a white solid was formed. The suspension was co-evaporated with toluene and the residue was purified by silica gel flash chromatography (Hex/EtOAc 95:5 to 9:1) to give alcohol **S12** (17 mg, quant.) as a white amorphous solid. R_f 0.67 (Tol/EtOAc 8:2); $[\alpha]^{20}_D$ +4 (*c* 0.4, $CHCl_3$); ^1H NMR (600 MHz, $CDCl_3$) δ (ppm) 7.37-7.24 (m, 10H, 10 × CH_{Bn}), 5.54-5.49 (m, 1H, H-3’’), 4.96 (d, J = 11.1 Hz, 1H, CHH$_{Bn}$), 4.89 (d, J = 11.1 Hz, 1H, CHH$_{Bn}$), 4.80 (d, J = 11.1 Hz, 1H, CHH_{Bn}), 4.52 (d, J = 11.2 Hz, 1H, CHH_{Bn}), 3.36 (d, J = 8.0 Hz, 1H, H-1), 4.37 (dd,

$J_{6a\text{-}6b}$ = 11.3 Hz, $J_{6a\text{-}5}$ = 10.1 Hz, 1H, H-6a), 4.27-4.23 (m, 1H, H-3'), 4.03 (dd, $J_{6b\text{-}6a}$ = 11.3 Hz, $J_{6b\text{-}5}$ = 1.9 Hz, 1H, H-6b), 3.61 (t, J = 8.9 Hz, 1H, H-3), 3.54 (td, $J_{5\text{-}6a,\,5\text{-}4}$ = 10.0 Hz, $J_{5\text{-}6b}$ = 1.9 Hz, 1H, H-5), 3.48-3.45 (m, 1H, H-2), 3.23 (dd, $J_{4\text{-}5}$ = 10.0 Hz, $J_{4\text{-}3}$ = 8.7 Hz, 1H, H-4), 2.65 (dd, $J_{2a'\text{-}2b'}$ = 18.5 Hz, $J_{2a'\text{-}3'}$ = 8.8 Hz, 1H, H-2a'), 2.60-2.52 (m, 2H, H-2''), 2.36 (d, $J_{2b'\text{-}2a'}$ = 18.3 Hz, 1H, H-2b'), 2.21 (d, J = 1.8 Hz, 1H, O*H*), 1.67-1.25 (m, 24H, 12 × CH_2); ^{13}C NMR (150 MHz, CDCl$_3$) δ (ppm) 171.3, 169.6 (2C, C-1', C-1''), 138.6 (C$_{Bn}$), 137.9 (C$_{Bn}$), 128.6-127.9 (10C, 10 × CH_{Bn}), 103.5 (C-1), 84.4 (C-3), 78,4 (C-4), 77.8 (C-3'), 75,3, 75,1 (3C, CH_{2Bn}, CH_{2Bn}, C-2), 72.8 (C-5), 69.7 (C-3''), 64.1 (C-6), 41.5 (C-2'), 40.5 (C-2''), 36.4-22.8 (12C, 12 × CH_2), 14.2 (2C, C-10', C-10''); HRMS (ESI-TOF) *m/z* [M + Na]$^+$ calcd for $C_{40}H_{58}NaO_9$ 705.3973; found 705.3988; *m/z* [M + NH$_4$]$^+$ calcd for $C_{40}H_{62}NO_{10}$ 700.4419; found 700.4435.

Synthetic Ananatoside A (1).

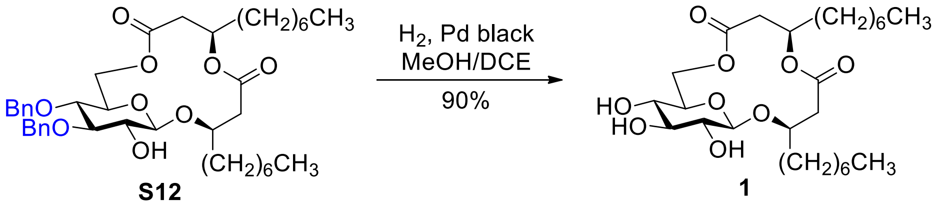

Route A: Protected macrolide **S12** (21 mg, 0.031 mmol, 1.0 equiv) was solubilized in MeOH (0.6 mL) and DCE (0.3 mL) under an Ar atmosphere. Pd black (21 mg, 1 mg•mg^{-1} of substrate) was added, and the mixture was stirred at 40 °C for 16 h under an H_2 atmosphere. The suspension was filtered over Celite and evaporated under reduced pressure. The residue was purified by silica gel flash chromatography (DCM/MeOH 99:1 to 95:5) to give ananatoside A (**1**, 14 mg, 90%) as a white amorphous solid. Analytical HPLC analysis was performed using method D (41.5 min.).

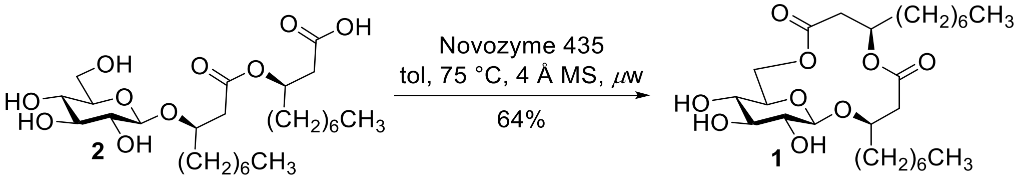

Route B: Novozyme 435 (2.4 mg) was dried over activated 4 Å MS (90 mg) in a dessicator under vacuum containing calcium sulfate at 63 °C for 3 days. The enzyme and the molecular sieves were then added to a suspension of ananatoside B (**2**, 4.7 mg, 9.0 μmol, 1.0 equiv) and 4 Å MS (90 mg) in anhydrous toluene (0.9 mL). The suspension was stirred at 75 °C under microwave radiations for 3 h then filtered over a pad of $MgSO_4$ and silica (DCM/MeOH 8:2) to give ananatoside A (**1**, 3 mg, 64%) as a white amorphous solid. R_f 0.43 (DCM/MeOH 9:1); $[\alpha]^{20}_{D}$ +19 (*c* 0.7, $CHCl_3$). Physical and analytical data of synthetic ananatoside A (**1**) agreed with our previously published data.[1]

***para*-Methylphenyl 4-*O*-Levulinoyl-3-*O*-*para*-methoxybenzyl-1-thio-*α*-L-rhamnopyranoside (S13).**

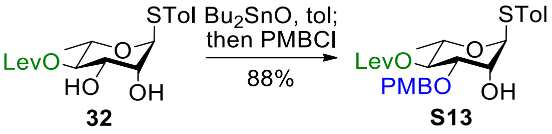

Bu_2SnO (1.54 g, 6.17 mmol, 1.1 equiv.) was added to a solution of diol **31**[5] (2.07 g, 5.61 mmol, 1.0 equiv.) in toluene (67 mL) and the mixture was refluxed using a Dean–Stark trap for 2 h. The solution was cooled to rt and CsF (895 mg, 5.89 mmol, 1.05 equiv.), TBAI (2.18 g, 5.89 mmol, 1.05 equiv.), and PMBCl (0.91 mL, 5.9 mmol, 1.2 equiv.) were successively added. The mixture was stirred under an Ar atmosphere at 40 °C for 16 h. The suspension was cooled at 0 °C, filtered over Celite, and rinsed with DCM. The solvents were evaporated under reduced pressure. The residue was purified by silica gel flash chromatography (Hex/EtOAc 9:1 to 7:3) to give compound **S13** (2.41 g, 88%) as a yellow oil: R_f 0.6 (Hex/EtOAc 4 : 6); $[\alpha]^{20}_{D}$ −140 (*c* 1.2, $CHCl_3$); ^1H NMR (600 MHz, CDCl3) *δ* (ppm) 7.33–7.32 (m, 2H, 2 × CH_{STol}), 7.27–7.26 (m, 2H, 2 × CH_{PMB}), 7.12–7.10 (m, 2H, 2 × CH_{STol}), 6.91–6.89 (m, 2H, 2 × CH_{PMB}), 5.46 (d, *J* = 1.2 Hz, 1H, H-1), 5.08 (t, *J* = 9.6 Hz, 1H, H-4), 4.59 (*d*, J = 11.7 Hz, 1H, CHH_{PMB}), 4.53 (*d*, J = 11.8 Hz, 1H, CHH_{PMB}), 4.22 (dq, $J_{5\text{-}4}$ = 9.9 Hz, $J_{5\text{-}6}$ = 6.2 Hz, 1H, H-5), 4.18 (dd, $J_{2\text{-}3}$ = 3.0 Hz, $J_{2\text{-}1}$ = 1.5 Hz, 1H, H-2), 3.81 (s, 3H, CH_{3PMB}), 3.75 (dd, $J_{3\text{-}4}$ = 9.4 Hz, $J_{3\text{-}2}$ = 3.3 Hz, 1H, H-3), 2.77–2.72 (m, 2H, CH_{2Lev}), 2.59–2.49 (m, 2H, CH_{2Lev}), 2.32 (s, 3H, CH_{3STol}), 2.19 (s, 3H, CH_{3Lev}), 1.18 (d, *J* = 6.3 Hz, 3H, H-6); ^{13}C NMR (150 MHz, CDCl3) *δ* (ppm) 206.5 (CO_{Lev}), 172.1 ($COOR_{Lev}$), 159.6 (C_{Ar}), 137.8 (C_{Ar}), 132.1 (2C, 2 × CH_{STol}), 130.0, 129.7 (5C, C_{Ar}, 2 × CH_{STol}, 2 × CH_{PMB}), 129.6 (C_{Ar}), 114.1 (2C, 2 × CH_{PMB}), 87.3 (C-1), 76.7 (C-3), 73.0 (C-4), 71.7 (CH_{2PMB}), 69.9 (C-2), 67.6 (C-5), 55.4 (CH_{3PMB}), 37.9 (CH_{2Lev}), 30.0 (CH_{3Lev}), 28.1 (CH_{2Lev}), 21.2 (CH_{3STol}), 17.4 (C-6); HRMS (ESI-TOF) *m/z* [M

+ $NH_4]^+$ calcd for $C_{26}H_{36}NO_7S$ 506.2207; found 506.2204; *m/z* [M + Na]$^+$ calcd for $C_{26}H_{32}NaO_7S$ 511.1761; found 511.1756.

***para*-Methylphenyl 2-*O*-*ortho*-(Azidomethyl)benzoyl-4-*O*-levulinoyl-3-*O*-*para*-methoxybenzyl-1-thio-*α*-L-rhamnopyranoside (25).**

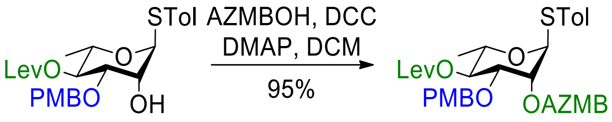

AZMBOH (229 mg, 1.29 mmol, 1.6 equiv), EDC (497 mg, 2.59 mmol, 3.3 equiv), and DMAP (201 mg, 0.863 mmol, 1.1 equiv) were successively added to a solution of alcohol **S13** (387 mg, 0.793 mmol, 1.0 equiv) in anhydrous DCM (8.6 mL). The mixture was refluxed under an Ar atmosphere for 4 h, then cooled at rt, and the solvents were evaporated under reduced pressure. The residue was purified by silica gel flash chromatography (Hex/EtOAc 95:5 to 7:3) to give fully protected **25** (490 mg, 95%) as a colorless oil: R_f 0.63 (Hex/EtOAc 1:1); $[\alpha]^{20}_{D}$ –24 (*c* 0.8, $CHCl_3$); ^{1}H NMR (600 MHz, $CDCl_3$) δ (ppm) 8.04-8.03 (m, 1H, CH_{AZMB}), 7.57-7.54 (m, 1H, CH_{AZMB}), 7.49-7.48 (m, 1H, CH_{AZMB}), 7.41-7.39 (m, 1H, CH_{AZMB}), 7.37-7.36 (m, 2H, 2 × CH_{STol}), 7.24-7.22 (m, 2H, 2 × CH_{PMB}), 7.14-7.12 (m, 2H, 2 × CH_{STol}), 6.87-6.85 (m, 2H, 2 × CH_{PMB}), 5.77 (dd, J_{2-3} = 3.2 Hz, J_{2-1} = 1.7 Hz, 1H, H-2), 5.49 (d, *J* = 1.5 Hz, 1H, H-1), 5.18 (t, *J* = 9.7 Hz, 1H, H-4), 4.78 (d, *J* = 14.6 Hz, 1H, CHH_{AZMB}), 4.74 (d, *J* = 14.6 Hz, 1H, CHH_{AZMB}), 4.63 (d, *J* = 11.8 Hz, 1H, CHH_{Bn}), 4.47 (d, *J* = 11.8 Hz, 1H, CHH_{Bn}), 4.33 (dq, J_{5-4} = 9.8 Hz, J_{5-6} = 6.2 Hz, 1H, H-5), 3.90 (dd, J_{3-4} = 9.7 Hz, J_{3-2} = 3.2 Hz, 1H, H-3), 3.80 (s, 3H, CH_{3PMB}), 2.79 (ddd, *J* = 18.3 Hz, *J* = 8.0 Hz, *J* = 5.8 Hz, 1H, CHH_{Lev}), 2.68 (dt, *J* = 18.3 Hz, *J* = 6.0 Hz, 1H, CHH_{Lev}), 2.63-2.58 (m, 1H, CHH_{Lev}), 2.50 (dt, *J* = 17.2 Hz, *J* = 6.1 Hz, 1H, CHH_{Lev}), 2.33 (s, 3H, CH_{3STol}), 2.18 (s, 3H, CH_{3Lev}), 1.25 (d, *J* = 6.2 Hz, 3H, H-6); ^{13}C NMR (150 MHz, $CDCl_3$) δ (ppm) 206.5 (CO_{Lev}), 172.1 ($COOR_{Lev}$), 165.9 ($COOR_{AZMB}$), 159.5 (C_{Ar}), 138.3 (C_{Ar}), 137.5-128.4 (14C, 4 × C_{Ar}, 10 × CH_{Ar}), 113.9 (2C, 2 × CH_{PMB}), 86.5 (C-1), 74.4 (C-3), 73.1 (C-4), 71.21, 71.17 (2C, CH_{2PMB}, C-2), 68.0 (C-5), 55.4 (CH_{3PMB}), 53.2 (CH_{2AZMB}), 38.0 (CH_{2Lev}), 30.0 (CH_{3Lev}), 28.1 (CH_{2Lev}), 21.3 (CH_{3STol}),

17.5 (C-6); HRMS (ESI-TOF) *m/z* $[M + NH_4]^+$ calcd for $C_{34}H_{41}N_4O_8S$ 665.2640; found 665.2623; *m/z* $[M + Na]^+$ calcd for $C_{34}H_{37}NaN_3O_8S$ 670.2194; found 670.2223.

Benzyl (*R*)-3-*O*-[(*R*)-(3'-*O*-Decyl)-2-*O*-*ortho*-(azidomethyl)benzoyl-4-*O*-levulinoyl-3-*O*-*para*-methoxybenzyl-*α*-L-rhamnopyranosyl]decanoate (24).

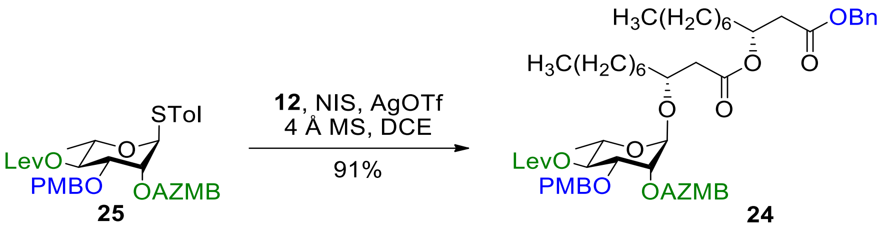

Donor **25** (210 mg, 0.324 mmol, 1.2 equiv), acceptor **12** (121 mg, 0.270 mmol, 1.0 equiv) and NIS (97 mg, 0.43 mmol, 1.6 equiv) were dried under high vacuum for 1 h. Activated 4 Å MS (484 mg, 4 mg•mg^{-1} of acceptor **12**) and anhydrous DCE (5.4 mL) were added and the suspension was stirred under an Ar atmosphere for 1 h. The mixture was cooled to –10 °C and AgOTf (14 mg, 0.054 mmol, 0.2 equiv) was added while the reaction flask was protected from light with aluminum foil. The suspension was stirred from to –10 to 0 °C for 1.5 h, quenched with Et_3N, and filtered over Celite. The solvents were evaporated under reduced pressure and the residue was purified by silica gel flash chromatography (Hex/EtOAc 95:5 to 8:2) to give rhamnolipid **24** (238 mg, 91%) as a yellow oil: R_f 0.30 (Hex/EtOAc 7:3); $[\alpha]^{20}_D$ +8 (*c* 0.6, $CHCl_3$); ^1H NMR (600 MHz, $CDCl_3$) *δ* (ppm) 8.05-8.03 (m, 1H, CH_{AZMB}), 7.57-7.54 (m, 1H, CH_{AZMB}), 7.49-7.48 (m, 1H, CH_{AZMB}), 7.42-7.39 (m, 1H, CH_{AZMB}), 7.36-7.30 (m, 5H, 5 × CH_{COOBn}), 7.18-7.16 (m, 2H, 2 × CH_{PMB}), 6.82-6.80 (m, 2H, 2 × CH_{PMB}), 5.45 (dd, $J_{2\text{-}3}$ = 3.2 Hz, $J_{2\text{-}1}$ = 1.9 Hz, 1H, H-2), 5.26-5.21 (m, 1H, H-3''), 5.12-5.10 (m, 3H, CH_{2COOBn}, H-4), 5.00 (d, *J* = 1.7 Hz, 1H, H-1), 4.79 (d, *J* = 14.7 Hz, 1H, CHH_{AZMB}), 4.73 (d, *J* = 14.7 Hz, 1H, CHH_{AZMB}), 4.59 (d, *J* = 11.7 Hz, 1H, CHH_{PMB}), 4.41 (d, *J* = 11.7 Hz, 1H, CHH_{PMB}), 4.08-4.04 (m, 1H, H-3'), 3.93-3.89 (m, 2H, H-3, H-5), 3.77 (s, 3H, CH_{3PMB}), 2.75 (ddd, *J* = 18.3 Hz, *J* = 7.8 Hz, *J* = 6.1 Hz, 1H, CHH_{Lev}), 2.68-2.63 (m, 2H, CHH_{Lev}, H-2a''), 2.61-2.52 (m, 3H, CHH_{Lev}, H-2a', H-2b''), 2.47-2.42 (m, 2H, CHH_{Lev}, H-2b'), 2.16 (s, 3H, CH_{3Lev}), 1.58-1.24 (m, 24H, 12 × CH_2), 1.21 (d, *J* = 6.3 Hz, 3H, H-6), 0.90-0.85 (m, 6H, 2 ×

CH_3); ^{13}C NMR (150 MHz, $CDCl_3$) δ (ppm) 206.5 (CO_{Lev}), 172.2, 170.6, 170.3 (3C, $COOR_{Lev}$, C-1', C-1''), 166.1 ($COOR_{AZMB}$), 159.3 (C_{Ar}), 137.5 (C_{Ar}), 135.9 (C_{Ar}), 133.0-128.4 (13C, 2 × C_{Ar}, 11 × CH_{Ar}), 113.8 (2C, 2 × CH_{PMB}), 96.8 (C-1), 75.3 (C-3'), 74.5 (C-3), 73.1 (C-4), 71.2, 71.0 (2C, CH_{2PMB}, C-3''), 70.1 (C-2), 67.1 (C-5), 66.6 (CH_{2COOBn}), 55.4 (CH_{3PMB}), 53.2 (CH_{2AZMB}), 40.4 (C-2'), 39.2 (C-2''), 38.0 (CH_{2Lev}), 34.0-22.8 (14C, 12 x CH_2, CH_{3Lev}, CH_{2Lev}), 17.6 (C-6), 14.2 (2C, 2 × CH_3); HRMS (ESI-TOF) *m/z* $[M + NH_4]^+$ calcd for $C_{54}H_{77}N_4O_{13}$ 989.5482; found 989.5479; *m/z* $[M + Na]^+$ calcd for $C_{54}H_{73}NaN_3O_{13}$ 994.5036; found 994.5053.

Benzyl (*R*)-3-*O*-[(*R*)-(3'-*O*-Decyl)-4-*O*-levulinoyl-3-*O*-*para*-methoxybenzyl-*α*-L-rhamnopyranosyl]decanoate (S14).

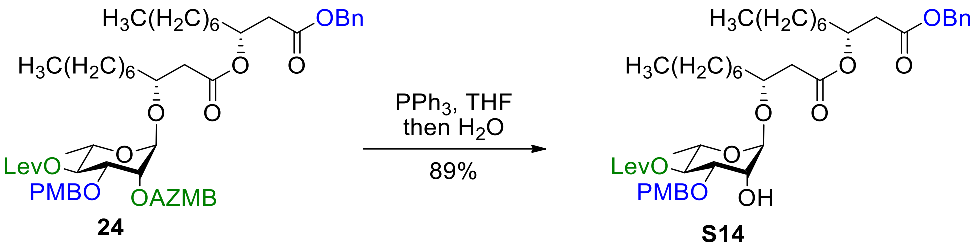

To a solution of rhamnolipid **24** (360 mg, 0.371 mmol, 1.0 equiv) in anhydrous THF (11 mL) was added PPh_3 (156 mg, 0.593 mmol, 1.6 equiv). The mixture was stirred at 60 °C under an Ar atmosphere for 2 h, after which water (1.5 mL) was added. The mixture was heated at 60 °C for 4 h, then co-evaporated with toluene. The residue was purified by silica gel flash chromatography (Hex/EtOAc 9:1 to 65:35) to give alcohol **S14** (269 mg, 89%) as a yellowish oil: R_f 0.37 (Hex/EtOAc 6:4); $[\alpha]^{20}_D$ –17 (*c* 0.6, $CHCl_3$); ^{1}H NMR (600 MHz, $CDCl_3$) δ (ppm) 7.36-7.30 (m, 5H, 5 × CH_{COOBn}), 7.23-7.21 (m, 2H, 2 × CH_{PMB}), 6.87-6.86 (m, 2H, 2 × CH_{PMB}), 5.25-5.21 (m, 1H, H-3''), 5.11 (s, 2H, CH_{2COOBn}), 5.01 (t, J = 9.7 Hz, 1H, H-4), 4.92 (d, J = 1.3 Hz, 1H, H-1), 4.55 (d, J = 11.7 Hz, 1H, CHH$_{PMB}$), 4.49 (d, J = 11.6 Hz, 1H, CHH_{PMB}), 4.05-4.01 (m, 1H, H-3'), 3.92 (br s, 1H, H-2), 3.86-3.82 (m, 1H, H-5), 3.79 (s, 3H, CH_{3PMB}), 3.71 (dd, $J_{3\text{-}4}$ = 9.5 Hz, $J_{3\text{-}2}$ = 3.3 Hz, 1H, H-3), 2.71-2.62 (m, 3H, CH_{2Lev}, H-2a''), 2.59-2.54 (m, 2H, H-2b'', H-2a'), 2.50-2.46 (m, 2H, CH_{2Lev}), 2.42 (dd, J = 15.2 Hz, J = 6.0 Hz, 1H, H-2b'), 2.16 (s, 3H, CH_{3Lev}), 1.62-1.22 (24H, 12 × CH_2), 1.16 (d, J = 6.3 Hz, 3H, H-6), 0.90-0.86 (m, 6H, 2 × CH_3); ^{13}C NMR (150 MHz, $CDCl_3$) δ (ppm) 206.4 (CO_{Lev}), 172.1, 170.6, 170.2 (3C, $COOR_{Lev}$, C-1', C-1''), 159.5 (C_{Ar}), 135.8 (C_{Ar}), 130.0-128.4 (8C, C_{Ar}, 7 × CH_{Ar}), 113.9 (2C, 2 × CH_{PMB}), 98.0 (C-1), 76.7 (C-3), 74.6 (C-3'), 72.9 (C-4), 71.7 (CH_{2PMB}), 70.9 (C-3''), 68.9 (C-2), 66.5 (2C, CH_{2COOBn}, C-5), 55.3 (CH_{3PMB}), 40.3 (C-2'), 39.1 (C-2''), 37.9 (CH_{2Lev}), 33.9-22.7 (14C, CH_{2Lev}, CH_{3Lev}, 12 × CH_2), 17.3 (C-6),

14.19 (CH_3), 14.17 (CH_3); HRMS (ESI-TOF) *m/z* $[M + NH_4]^+$ calcd for $C_{46}H_{72}NO_{12}$ 830.5049 found 830.5056; *m/z* $[M + Na]^+$ calcd for $C_{46}H_{68}NaO_{12}$ 835.4603; found 835.4616.

Benzyl (*R*)-3-*O*-[(*R*)-(3'-*O*-Decyl)-3-*O*-*para*-methoxybenzyl-*α*-L-rhamnopyranosyl]decanoate (33).

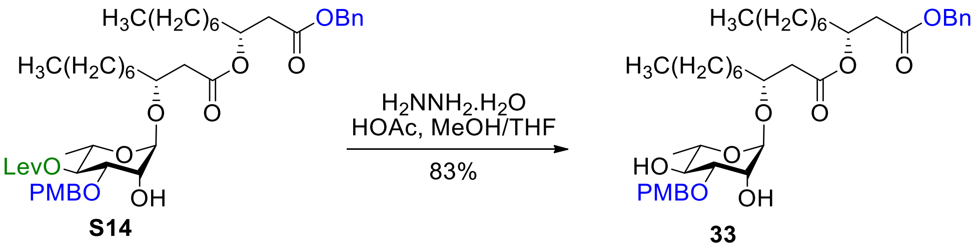

To a solution of compound **S14** (212 mg, 0.261 mmol, 1.0 equiv) in anhydrous THF/MeOH (10:1 *v/v*, 18.3 mL) was slowly added a solution of $H_2NNH_2.H_2O$ (177 μL, 3.66 mmol, 14 equiv) and HOAc (444 μL) in anhydrous THF/MeOH (5:1 *v/v*, 3 mL). The solution was stirred under an Ar atmosphere for 45 min until a white solid was formed. The suspension was co-evaporated with toluene and the residue was purified by silica gel flash chromatography (Hex/EtOAc 9:1 to 7:3) to give diol **33** (155 mg, 83%) as a colorless oil: R_f 0.52 (Hex/EtOAc 1:1); $[\alpha]^{20}_D$ –19 (*c* 0.6, $CHCl_3$); ^{1}H NMR (600 MHz, $CDCl_3$) δ (ppm) 7.36-7.31 (m, 5H, 5 × CH_{COOBn}), 7.27-7.26 (m, 2H, 2 × CH_{PMB}), 6.88-6.86 (m, 2H, 2 × CH_{PMB}), 5.25-5.21 (m, 1H, H-3''), 5.10 (d, J = 1.1 Hz, 2H, CH_{2COOBn}), 4.91 (d, J = 1.4 Hz, 1H, H-1), 4.60 (d, J = 11.2 Hz, 1H, CHH$_{PMB}$), 4.53 (d, J = 11.2 Hz, 1H, CHH_{PMB}), 4.11-4.07 (m, 1H, H-3'), 3.94 (dd, $J_{2\text{-}3}$ = 3.1 Hz, $J_{2\text{-}1}$ = 1.6 Hz, 1H, H-2), 3.79 (s, 3H, CH_{3PMB}), 3.74 (dq, $J_{5\text{-}4}$ = 9.6 Hz, $J_{5\text{-}6}$ = 6.5 Hz, 1H, H-5), 3.60 (dd, $J_{3\text{-}4}$ = 9.2 Hz, $J_{3\text{-}2}$ = 3.3 Hz, 1H, H-3), 3.50 (t, J = 9.4 Hz, 1H, H-4), 2.65 (dd, J = 15.6 Hz, J = 7.6 Hz, 1H, H-2a''), 2.60-2.53 (m, 2H, H-2b'', H-2a'), 2.41 (dd, J = 15.3 H, J = 5.6 Hz, 1H, H-2b'), 1.62-1.26 (m, 27H, H-6, 12 × CH_2), 0.90-0.86 (m, 6H, 2 × CH_3); ^{13}C NMR (150 MHz, $CDCl_3$) δ (ppm) 170.8, 170.6 (2C, C-1', C-1''), 159.6 (C_{Ar}), 135.7 (C_{Ar}), 130.1-128.4 (8C, C_{Ar}, 7 × CH$_{Ar}$), 114.2 (2C, 2 × CH$_{PMB}$), 97.9 (C-1), 79.7 (C-3), 73.9 (C-3'), 72.0 (C-4), 71.6 (CH$_{2PMB}$), 70.9 (C-3''), 68.5 (C-2), 68.2 (C-5), 66.7

(CH_{2COOBn}), 55.4 (CH_{3PMB}), 40.2 (C-2’), 39.2 (C-2’’), 34.1-22.8 (12C, 12 × CH_2), 17.7 (C-6), 14.23 (CH_3), 14.21 (CH_3); HRMS (ESI-TOF) *m/z* $[M + Na]^+$ calcd for $C_{41}H_{62}NaO_{10}$ 737.4235; found 737.4245; *m/z* $[M + Na]^+$ calcd for $C_{41}H_{62}KO_{10}$ 753.3975; found 753.3982.

Rhamnolipid 3.

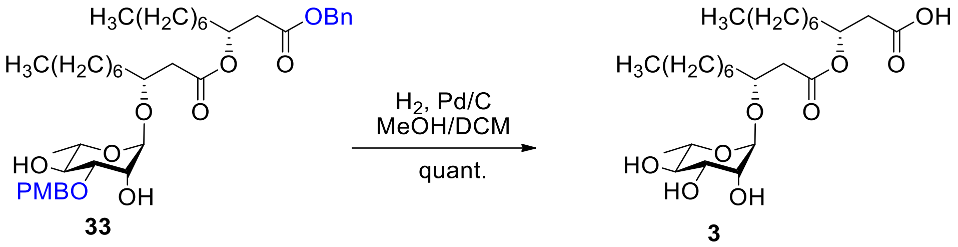

Pd black (12 mg, 1 mg•mg^{-1} of diol **33**) was added to a solution of diol **33** (12 mg, 17 μmol, 1.0 equiv) in MeOH (0.34 mL) and DCE (0.17 mL). The suspension was stirred under an H_2 atmosphere at 40 °C for 16 h. The mixture was then filtered over Celite and the solvents were evaporated under reduced pressure. The residue was purified by silica gel flash chromatography (DCM/MeOH 95:5 to 8:2) to give C_{10}-C_{10} rhamnolipid **3** (7.9 mg, quant.) as a colorless oil. R_f 0.61 (DCM/MeOH 8:2); $[\alpha]^{20}_{D}$ –45 (*c* 0.7, $CHCl_3$); HRMS (ESI-TOF) *m/z* $[M + NH_4]^+$ calcd for $C_{26}H_{52}NO_9$ 522.3637; found 522.3639; *m/z* $[M + Na]^+$ calcd for $C_{26}H_{48}NaO_9$ 527.3191; found 527.3189. Physical and analytical data of C_{10}-C_{10} rhamnolipid (**3**) agreed with those published.[6] Analytical HPLC analysis was performed using method C (36.3 min.).

***para*-Methylphenyl 2-*O*-*ortho*-(Azidomethyl)benzoyl-3,4-*O*-(2,3-dimethoxybutan-2,3-diyl)-1-thio-*α*-L-rhamnopyranoside (S7).**

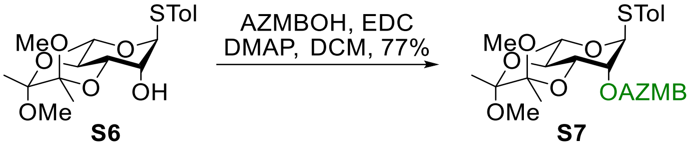

To a solution of compound **S6**[7] (129 mg, 0.33 mmol, 1.0 equiv) in anhydrous DCM (3.3 mL) were successively added DMAP (78 mg, 0.050 mmol, 1.0 equiv), EDC (129 mg, 1.00 mmol, 3.0 equiv), and AZMBOH (89 mg, 0.37 mmol, 1.5 equiv). The mixture was refluxed for 4 h under an Ar atmosphere, then cooled at rt and the solvents were evaporated under reduced pressure. The residue was purified by silica gel flash chromatography (Hex/EtOAc 98:2 to 95:5) to give compound **S7** (141 mg, 77%) as a white amorphous solid: R_f 0.67 (Hex/EtOAc 8:2); $[\alpha]^{20}_{D}$ –164 (*c* 0.5, $CHCl_3$); ^{1}H NMR (600 MHz, $CDCl_3$) δ (ppm) 8.04-8.02 (m, 1H, CH_{AZMB}), 7.58-7.53 (m, 2H, 2 × CH_{AZMB}), 7.41-7.38 (m, 3H, CH_{AZMB}, 2 × CH_{STol}), 7.12-7.11 (m, 2H, 2 × CH_{STol}), 5.53-5.52 (m, 2H, H-1, H-2), 4.87 (d, J = 15.2 Hz, 1H, CHH_{AZMB}), 4.81 (d, J = 15.2 Hz, 1H, CHH_{AZMB}), 4.37 (dq, $J_{5\text{-}4}$ = 9.6 Hz, $J_{5\text{-}6}$ = 6.1 Hz, 1H, H-5), 4.19 (dd, $J_{3\text{-}4}$ = 10.2 Hz, $J_{3\text{-}2}$ = 3.1 Hz, 1H, H-3), 3.90 (t, J = 10.0 Hz, 1H, H-4), 3.33 (s, 3H, CH_{3OMe}), 3.31 (s, 3H, CH_{OMe}), 2.32 (s, 3H, CH_{3STol}), 1.35 (s, 3H, CH_3), 1.31-1.30 (m, 6H, H-6, CH_3); ^{13}C NMR (150 MHz, $CDCl_3$) δ (ppm) 166.7 ($COOR_{AZMB}$), 138.1-128.0 (12C, 4 × C_{Ar}, 8 × CH_{Ar}), 100.4 (2C, 2 × $C(O)_2CH_3$), 86.7 (C-1), 73.9 (C-2), 69.4 (C-4), 67.9 (C-5), 66.9 (C-3), 53.2 (CH_{2AZMB}), 48.3 (CH_{3OMe}), 47.9 (CH_{3OMe}), 21.2 (CH_{3STol}), 17.9 (CH_3), 17.7 (CH_3), 16.7 (C-6); HRMS (ESI-TOF) *m/z* $[M + Na]^+$ calcd for $C_{27}H_{33}NaN_3O_7S$ 566.1931; found 566.1953; *m/z* $[M + K]^+$ calcd for $C_{27}H_{33}KN_3O_7S$ 582.1671; found 582.1687.

1-*O*-{Benzyl (*R*)-3-*O*-[(*R*)-(3'-*O*-Decyl)-2-*O*-*ortho*-(azidomethyl)benzoyl-[3,4-*O*-(2,3-dimethoxybutan-2,3-diyl)]-*α*-L-rhamnopyranosyl} Decanoate (S8).

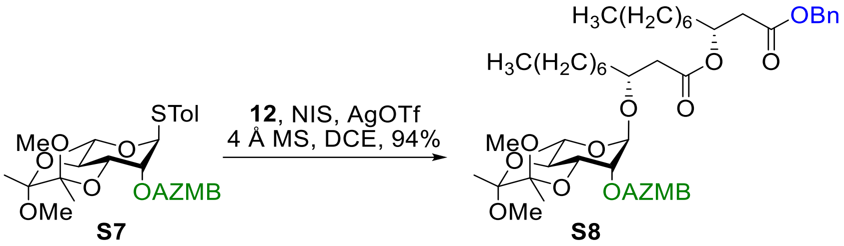

Donor **S7** (85 mg, 0.16 mmol, 1.2 equiv), acceptor **12** (59 mg, 0.13 mmol, 1.0 equiv), and NIS (47 mg, 0.21 mmol, 1.6 equiv) were dried under high vacuum for 1 h. Activated 4 Å MS (234 mg, 4 mg•mg^{-1} of acceptor **12**) and anhydrous DCE (2.6 mL) were added and the suspension was stirred under an Ar atmosphere for 1 h. The mixture was cooled to –10 °C and AgOTf (7 mg, 0.03 mmol, 0.2 equiv) was added while the reaction flask was protected from light with aluminum foil. The suspension was stirred from –10 to 0 °C for 1.5 h, quenched with Et_3N, and filtered over Celite. The solvents were evaporated under reduced pressure and the residue was purified by silica gel flash chromatography (Tol/EtOAc 99:1 to 985:15) to give compound **S8** (106 mg, 94%) as a colorless oil: R_f 0.56 (Tol/EtOAc 95:5); $[\alpha]^{20}_{D}$ –74 (*c* 0.6, $CHCl_3$); ^{1}H NMR (600 MHz, $CDCl_3$) δ (ppm) 8.05-8.04 (m, 1H, CH_{AZMB}), 7.59-7.55 (m, 2H, 2 × CH_{AZMB}), 7.42-7.39 (m, 1H, CH_{AZMB}), 7.35-7.31 (m, 5H, 5 × CH_{AZMB}), 5.25-5.21 (m, 1H, H-3''), 5.18 (dd, $J_{2\text{-}3}$ = 3.2 Hz, $J_{2\text{-}1}$ = 1.5 Hz, 1H, H-2), 5.11 (s, 2H, CH_{2COOBn}), 4.99 (d, J = 1.1 Hz, 1H, H-1), 4.89 (d, J = 15.2 Hz, 1H, CHH_{AZMB}), 4.82 (d, J = 15.3 Hz, 1H, CHH_{AZMB}), 4.14 (dd, $J_{3\text{-}4}$ = 10.2 Hz, $J_{3\text{-}2}$ = 3.3 Hz, 1H, H-3), 4.08-4.04 (m, 1H, H-3'), 3.94-3.90 (m, 1H, H-5), 3.79 (t, J = 10.0 Hz, 1H, H-4), 3.28 (s, 3H, CH_{3OMe}), 3.27 (s, 3H, CH_{3OMe}), 2.69 (dd, J = 15.5 Hz, J = 7.0 Hz, 1H, H-2a''), 2.64-2.56 (m, 2H, H-2b'', H-2a'), 2.42 (dd, J = 15.3 Hz, J = 6.9 Hz, 1H, H-2b'), 1.59-1.23 (m, 33H, 12 × CH_2, 2 × CH_3, H-6), 0.89-0.85 (m, 6H, 2 × CH_3); ^{13}C NMR (150 MHz, $CDCl_3$) δ (ppm) 170.5, 170.3 (2C,

C-1’, C-1’’), 166.9 (*C*OOR$_{AZMB}$), 137.3-128.0 (12C, 3 × C_{Ar}, 9 × CH_{Ar}), 100.3 ($C(O)_2CH_3$), 99.9 ($C(O)_2CH_3$), 97.3 (C-1), 75.7 (C-3’), 72.7 (C-2), 70.9 (C-3’’), 69.2 (C-4), 67.1 (C-5), 66.6, 66.2 (2C, CH_{2COOBn}, C-3), 53.3 (CH_{2AZMB}), 48.2 (CH_{3OMe}), 47.8 (CH_{3OMe}), 40.6 (C-2’), 39.2 (C-2’’), 34.0-22.8 (12C, 12 × CH_2), 17.9, 17.8 (2C, 2 × CH_3), 16.7 (C-6), 14.2 (2C, 2 × CH_3); HRMS (ESI-TOF) *m/z* $[M + NH_4]^+$ calcd for $C_{47}H_{77}N_4O_{12}$ 885.5220 found 885.5225; *m/z* $[M + Na]^+$ calcd for $C_{47}H_{69}NaN_3O_{12}$ 890.4774; found 890.4779.

Benzyl (*R*)-3-*O*-[(*R*)-(3'-*O*-Decyl)-*α*-L-rhamnopyranosyl]decanoate (S9).

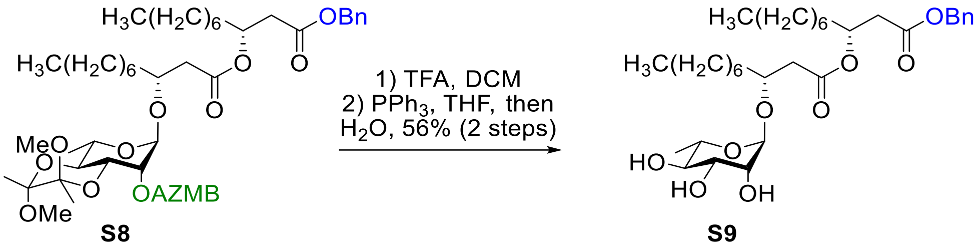

TFA (223 μL) and H_2O (12 μL) were added to a solution of compound **S8** (20 mg, 23 μmol, 1.0 equiv) in DCM (230 μL). The mixture was stirred at rt for 1 h and quenched with saturated aqueous $NaHCO_3$. The organic layer was washed with brine, dried over $MgSO_4$, filtered, and the solvents were evaporated under reduced pressure. The residue was filtered over silica gel, then solubilized in anhydrous THF (0.7 mL). PPh_3 (9 mg, 40 μmol, 1.6 equiv) was added and the mixture was stirred under an Ar atmosphere at 60 °C for 2 h. H_2O (90 μL) was then added and the solution was stirred at 60 °C for 4 h. The solution was co-evaporated with toluene and the residue was purified by silica gel flash chromatography (DCM/MeOH 99:1 to 97:3) to give triol **S9** (8 mg, 56%) as a colorless oil: R_f 0.38 (DCM/MeOH 95:5); $[\alpha]^{20}_D$ –60 (*c* 0.1, $CHCl_3$); ^1H NMR (600 MHz, $CDCl_3$) δ (ppm) 7.38-7.32 (m, 5H, 5 × CH_{COOBn}), 5.28-5.22 (m, 1H, H-3''), 5.11 (s, 2H, CH_{2COOBn}), 4.88 (d, J = 1.4 Hz, 1H, H-1), 4.21-4.15 (m, 1H, H-3'), 3.86-3.80 (m, 1H, H-2), 3.76-3.68 (m, 2H, H-3, H-5), 3.38-3.32 (m, 1H, H-4), 2.66 (dd, $J_{2a''-2b''}$ = 16.1 Hz, $J_{2a''-3''}$ = 8.4 Hz, 1H, H-2a''), 2.59 (dd, $J_{2b''-2a''}$ = 16.1 Hz, $J_{2b''-3''}$ = 4.2 Hz, 1H, H-2b''), 2.47 (dd, $J_{2a'-2b'}$ = 15.4 Hz, $J_{2a'-3'}$ = 8.5 Hz, 1H, H-2a'), 2.40 (dd, $J_{2b'-2a'}$ = 15.4 Hz, $J_{2b'-3'}$ = 4.2 Hz, 1H, H-2b'), 1.52-1.45 (m, 2H, CH_2), 1.31 (d, J = 6.2 Hz, 3H, H-6), 1.26-1.21 (m, 22H, 11 × CH_2), 0.89-0.84 (m, 6H, 2 × CH_3); ^{13}C NMR (150 MHz, $CDCl_3$) δ (ppm) 171.2 (2C, C-1', C-1''), 135.5 (C_{COOBn}), 128.8-128.3 (5C, 5 × CH_{COOBn}), 96.9 (C-1), 74.0 (C-4), 72.9 (C-3'), 72.0 (C-3'), 71.3 (C-2), 70.9 (C-3''), 68.2 (C-5), 67.0 (CH_{2COOBn}), 40.0 (C-2'), 39.2 (C-2''), 34.2-22.8 (12C, 12 × CH_2), 17.4 (C-6), 14.2 (2C, 2 × CH_3);

HRMS (ESI-TOF) *m/z* $[M + NH_4]^+$ calcd for $C_{33}H_{58}NO_9$ 612.4106; found 612.4100; *m/z* $[M + Na]^+$ calcd for $C_{33}H_{54}NaO_9$ 617.3660; found 617.3657.

Rhamnolipid 3 from compound S9.

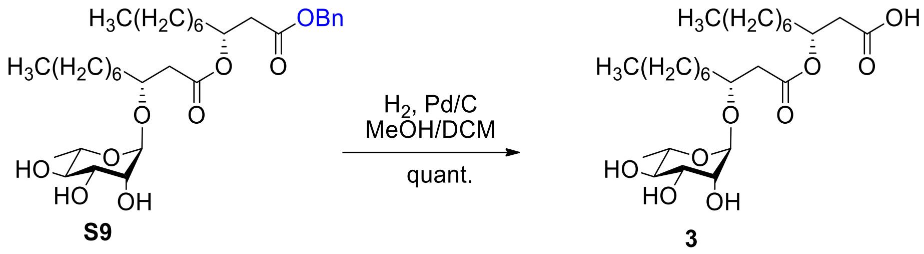

Pd black (11 mg, 1 mg•mg^{-1} of triol **S9**) was added to a solution of triol **S9** (11 mg, 19 μmol, 1.0 equiv) in MeOH (0.38 mL) and DCE (0.19 mL). The suspension was stirred under an H_2 atmosphere at 40 °C for 16 h. The mixture was then filtered over Celite and the solvents were evaporated under reduced pressure. The residue was purified by silica gel flash chromatography (DCM/MeOH 95:5 to 8:2) to give C_{10}-C_{10} rhamnolipid **3** (9.5 mg, quant.) as a colorless oil. Physical and analytical data of C_{10}-C_{10} rhamnolipid (**3**) agreed with those published.[6]

***para*-Methylphenyl 3-*O*-Benzyl-4-*O*-levulinoyl-1-thio-*α*-L-rhamnopyranoside (30).**

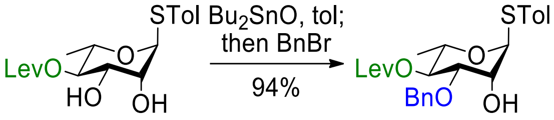

32 **30**

Bu_2SnO (128 mg, 0.514 mmol, 1.1 equiv) was added to a solution of diol **32**[5] (172 mg, 0.467 mmol, 1.0 equiv) in anhydrous toluene (5.6 mL). The suspension was refluxed with a Dean-Stark apparatus for 2 h and cooled to rt. CsF (75 mg, 0.49 mmol, 1.05 equiv), TBAI (181 mg, 0.491 mmol, 1.05 equiv), and BnBr (67 μL, 0.56 mmol, 1.2 equiv) were then added to the mixture and the latter was stirred at 40 °C for 16 h under an Ar atmosphere. The suspension was cooled at 0 °C, filtered over Celite, and the solvents were evaporated under reduced pressure. The residue was purified by silica gel flash chromatography (Hex/EtOAc 9:1 to 6:4) to give alcohol **30** (201 mg, 94%) as a white amorphous solid: R_f 0.48 (Hex/EtOAc 1:1); $[\alpha]^{20}_D$ –149 (*c* 0.9, $CHCl_3$); ^{1}H NMR (600 MHz, $CDCl_3$) δ (ppm) 7.39-7.36 (m, 2H, 2 × CH_{Bn}), 7.34-7.31 (m, 5H, 2 × CH_{STol}, 3 × CH_{Bn}), 7.12-7.11 (m, 2H, 2 × CH_{STol}), 5.47 (d, J = 1.5 Hz, 1H, H-1), 5.11 (t, J = 9.6 Hz, 1H, H-4), 4.67 (d, J = 12.0 Hz, 1H, CHH_{Bn}), 4.60 (d, J = 12.0 Hz, 1H, CHH_{Bn}), 4.26-4.21 (m, 2H, H-2, H-5), 3.77 (dd, $J_{3\text{-}4}$ = 9.4 Hz, $J_{3\text{-}2}$ = 3.3 Hz, 1H, H-3), 2.79-2.68 (m, 2H, CH_{2Lev}), 2.59-2.48 (m, 2H, CH_{2Lev}), 2.33 (s, 3H, CH_{3STol}), 2.18 (s, 3H, CH_{3Lev}), 1.19 (d, J = 6.3 Hz, 3H, H-6); ^{13}C NMR (150 MHz, $CDCl_3$) δ (ppm) 206.5 (CO_{Lev}), 172.2 ($COOR_{Lev}$), 137.9 (C_{Ar}), 137.6 (C_{Ar}), 132.1-128.1 (10C, C_{Ar}, 9 x CH_{Ar}), 87.3 (C-1), 77.0 (C-3), 73.0 (C-4), 72.1 (CH_{2Bn}), 69.9 (C-2), 67.6 (C-5), 38.0 (CH_{2Lev}), 30.0 (CH_{3Lev}), 28.1 (CH_{2Lev}), 21.2 (CH_{3STol}), 17.4 (C-6); HRMS (ESI-TOF) *m/z* $[M + NH_4]^+$ calcd for $C_{25}H_{34}NO_6S$ 476.2101; found 476.2115; *m/z* $[M + Na]^+$ calcd for $C_{25}H_{30}NaO_6S$ 481.1655; found 481.1670.

***para*-Methylphenyl 2-*O*-*ortho*-(Azidomethyl)benzoyl-3-*O*-benzyl-4-*O*-levulinoyl-1-thio-*α*-L-rhamnopyranoside (S15).**

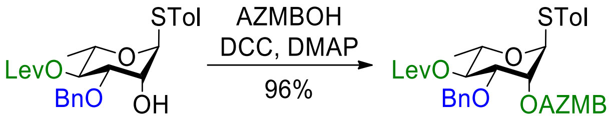

To a solution of alcohol **30** (380 mg, 0.828 mmol, 1.0 equiv) in anhydrous DCM (8.3 mL) were added DMAP (101 mg, 0.828 mmol, 1.0 equiv), DCC (342 mg, 1.66 mmol, 2.0 equiv), and AZMBOH (220 mg, 1.24 mmol, 1.5 equiv). The solution was refluxed under an Ar atmosphere for 4 h, then cooled at 0 °C, filtered over Celite, and the solvents were evaporated under reduced pressure. The residue was purified by silica gel flash chromatography (Hex/EtOAc 9:1 to 8:2) to give compound **S15** (493 mg, 96%) as a colorless oil: R_f 0.51 (Hex/EtOAc 6:4); $[\alpha]^{20}_{D}$ –22 (*c* 2.2, $CHCl_3$); ^{1}H NMR (600 MHz, $CDCl_3$) δ (ppm) 8.05-8.03 (m, 1H, CH_{AZMB}), 7.56-7.54 (m, 1H, CH_{AZMB}), 7.49-7.47 (m, 1H, CH_{AZMB}), 7.42-7.39 (m, 1H, CH_{AZMB}), 7.37-7.36 (m, 2H, 2 × CH_{STol}), 7.34-7.37 (m, 5H, 5 × CH_{Bn}), 7.14-7.12 (m, 2H, 2 × CH_{STol}), 5.79 (dd, $J_{2\text{-}3}$ = 3.2 Hz, $J_{2\text{-}1}$ = 1.7 Hz, 1H, H-2), 5.50 (d, J = 1.5 Hz, 1H, H-1), 5.21 (t, J = 9.7 Hz, 1H, H-4), 4.77 (d J = 14.6 Hz, 1H, CHH$_{AZMB}$), 4.74-4.70 (m, 2H, CHH_{AZMB}, CHH$_{Bn}$), 4.54 (d, J = 12.1 Hz, 1H, CHH_{Bn}), 4.34 (dq, $J_{5\text{-}4}$ = 9.9 Hz, $J_{5\text{-}6}$ = 6.2 Hz, 1H, H-5), 3.92 (dd, $J_{3\text{-}4}$ = 9.7 Hz, $J_{3\text{-}2}$ = 3.2 Hz, 1H, H-3), 2.80 (ddd, J = 18.1 Hz, J = 8.0 Hz, J = 5.7 Hz, 1H, CHH$_{Lev}$), 2.69-2.58 (m, 2H, CHH_{Lev}, CHH$_{Lev}$), 2.49 (dt, J = 17.2 Hz, J = 6.0 Hz, 1H, CHH_{Lev}), 2.33 (s, 3H, CH_{3STol}), 2.17 (s, 3H, CH_{3Lev}), 1.26 (d, J = 6.2 Hz, 3H, H-6); ^{13}C NMR (150 MHz, $CDCl_3$) δ (ppm) 206.5 (CO_{Lev}), 172.1 ($COOR_{Lev}$), 165.9 ($COOR_{AZMB}$), 138.3 (C_{Ar}), 137.6 (C_{Ar}), 137.5 (C_{Ar}), 133.1-128.0 (15C, 2 × C_{Ar}, 12 × CH_{Ar}), 86.4 (C-1), 75.0 (C-3), 73.1 (C-4), 71.6 (CH_{2Bn}), 71.1 (C-2), 68.0 (C-5), 53.1 (CH_{2AZMB}), 38.0 (CH_{2Lev}), 29.9 (CH_{3Lev}), 28.1 (CH_{2Lev}), 21.3 (CH_{3STol}), 17.5 (C-6); HRMS (ESI-TOF) *m/z* $[M + NH_4]^+$ calcd for $C_{33}H_{39}N_4O_7S$ 635.2534; found 635.2540.

***para*-Methylphenyl 2-*O*-*ortho*-(Azidomethyl)benzoyl-3-*O*-benzyl-1-thio-*α*-L-rhamnopyranoside (29).**

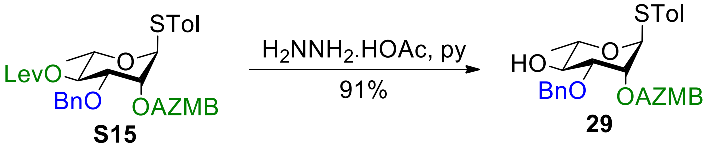

AcOH (2.4 mL) and hydrazine monohydrate (139 μL, 2.87 mmol, 5.0 equiv) were successively added to a solution of compound **S15** (409 mg, 0.662 mmol, 1.0 equiv) in anhydrous pyridine (3.7 mL) at 0 °C. The mixture was stirred at rt for 16 h under an Ar atmosphere then co-evaporated with toluene. The residue was purified by silica gel flash chromatography (Hex/EtOAc 9:1 to 8:2) to give alcohol **29** (313 mg, 91%) as a yellow oil: R_f 0.67 (Hex/EtOAc 6:4); $[\alpha]^{20}_{D}$ –23 (*c* 1.1, $CHCl_3$); ^1H NMR (600 MHz, $CDCl_3$) δ (ppm) 8.02-8.01 (m, 1H, CH_{AZMB}), 7.57-7.55 (m, 1H, CH_{AZMB}), 7.59-7.48 (m, 1H, CH_{AZMB}), 7.41-7.38 (m, 3H, CH_{AZMB}, 2 × CH_{STol}), 7.32-7.29 (m, 5H, 5 × CH_{Bn}), 7.14-7.12 (m, 2H, 2 × CH_{STol}), 5.82 (dd, $J_{2\text{-}3}$ = 2.7 Hz, $J_{2\text{-}1}$ = 1.6 Hz, 1H, H-2), 5.50 (br s, 1H, H-1), 4.79 (d, J = 11.3 Hz, 1H, CHH_{Bn}), 4.74 (s, 2H, CH_{2AZMB}), 4.54 (d, J = 11.3 Hz, 1H, CHH_{Bn}), 4.27 (dq, $J_{5\text{-}4}$ = 9.3 Hz, $J_{5\text{-}6}$ = 6.2 Hz, 1H, H-5), 3.83 (dd, $J_{3\text{-}4}$ = 9.4 Hz, $J_{3\text{-}2}$ = 3.1 Hz, 1H, H-3), 3.74 (t, J = 9.4 Hz, 1H, H-4), 2.33 (s, 3H, CH_{3STol}), 1.38 (d, J = 6.2 Hz, 3H, H-6); ^{13}C NMR (150 MHz, $CDCl_3$) δ (ppm) 165.8 ($COOR_{AZMB}$), 138.2 (C_{Ar}), 137.5 (C_{Ar}), 137.4 (C_{Ar}), 133.2-128.3 (14C, C_{Ar}, 13 × CH_{Ar}), 86.7 (C-1), 78.1 (C-3), 72.4 (C-4), 71.7 (CH_{2Bn}), 70.7 (C-2), 69.4 (C-5), 53.1 (CH_{2AZMB}), 21.3 (CH_{3STol}), 17.9 (C-6); HRMS (ESI-TOF) *m/z* $[M + Na]^+$ calcd for $C_{28}H_{29}NaN_3O_5S$ 542.1720; found 542.1731; *m/z* $[M + K]^+$ calcd for $C_{28}H_{29}KN_3O_5S$ 558.1460; found 558.1470.

***para*-Methylphenyl 2-*O*-*ortho*-(Azidomethyl)benzoyl-3-*O*-benzyl-4-*O*-(*R*)-3-(((*R*)-3-hydroxydecanoyl)oxy)decanoyl-1-thio-*α*-L-rhamnopyranoside (34).**

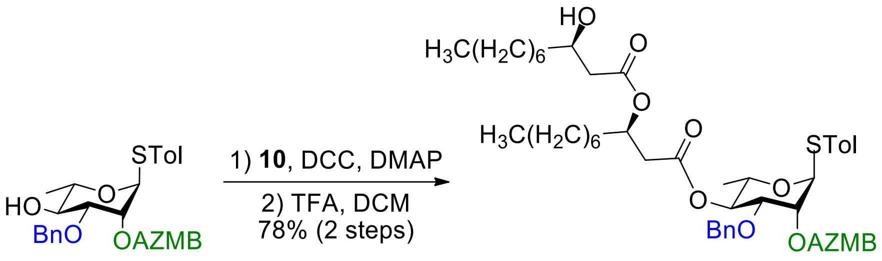

Alcohol **29** (195 mg, 0.375 mmol, 1.0 equiv) and acid **10** (248 mg, 0.525 mmol, 1.4 equiv) were solubilized in anhydrous DCE (4.5 mL). To this solution were successively added DMAP (5 mg, 0.04 mmol, 0.1 equiv) and DCC (232 mg, 1.13 mmol, 3.0 equiv). The mixture was refluxed for 1 h under an Ar atmosphere, then cooled at 0 °C, filtered over Celite, and the solvents were evaporated under reduced pressure. The residue was filtered over silica gel to remove most of the impurities, then solubilized in DCM (3.7 mL). TFA (3.6 mL) was added to the latter solution and the reaction mixture was stirred at rt for 10 min after which it was quenched with saturated aqueous $NaHCO_3$. The organic layer was dried over $MgSO_4$, filtered, and evaporated under reduced pressure. The residue was purified by silica gel flash chromatography (Hex:EtOAc 9:1 to 8:2) to give alcohol **34** (245 mg, 78% over 2 steps) as a colorless oil: R_f 0.63 (Hex/EtOAc 7:3); $[\alpha]^{20}_D$ –20 (*c* 0.7, $CHCl_3$); ^{1}H NMR (600 MHz, $CDCl_3$) δ (ppm) 8.05-8.03 (m, 1H, CH_{AZMB}), 7.57-7.55 (m, 1H, CH_{AZMB}), 7.49-7.48 (m, 1H, CH_{AZMB}), 7.44-7.41 (m, 1H, CH_{AZMB}), 7.37-7.36 (m, 2H, 2 × CH_{STol}), 7.32-7.26 (m, 5H, 5 × CH_{Bn}), 7.14-7.12 (m, 2H, 2 × CH_{STol}), 5.79 (dd, $J_{2\text{-}3}$ = 3.2 Hz, $J_{2\text{-}1}$ = 1.8 Hz, 1H, H-2), 5.51 (d, J = 1.6 Hz, 1H, H-1), 5.27-5.20 (m, 2H, H-4, H-3’’), 4.77-4.69 (m, 3H, CH_{2AZMB}, CHH$_{Bn}$), 4.52 (d, J = 12.1 Hz, 1H, CHH_{Bn}), 4.33 (dq, $J_{5\text{-}4}$ = 9.8 Hz, $J_{5\text{-}6}$ = 6.2 Hz, 1H, H-5), 4.00-3.96 (m, 1H, H-3’), 3.93 (dd, $J_{3\text{-}4}$ = 9.7 Hz, $J_{3\text{-}2}$ = 3.2 Hz, 1H, H-3), 2.61 (dd, $J_{2a''\text{-}2b''}$ = 16.0 Hz, $J_{2a''\text{-}3''}$ = 7.2 Hz, 1H, H-2a’’), 2.51 (dd, $J_{2b''\text{-}2a''}$ = 16.0 Hz, $J_{2b''\text{-}3''}$ = 5.4 Hz, 1H, H-

2b''), 2.45 (dd, $J_{2a'-2b'}$ = 16.1 Hz, $J_{2a'-3'}$ = 2.9 Hz, 1H, H-2a'), 2.36-2.32 (m, 4H, H-2b', CH_{3STol}), 1.62-1.24 (m, 27H, H-6, 12 × CH_2), 0.89-0.86 (m, 6H, 2 × CH_3); ^{13}C NMR (150 MHz, $CDCl_3$) δ (ppm) 172.5, 169.7 (2C, C-1', C-1''), 165.9 (COOR$_{AZMB}$), 138.4 (C_{Ar}), 137.6 (C_{Ar}), 137.5 (C_{Ar}), 133.2-127.8 (15C, 2 × C_{Ar}, 13 × CH$_{Ar}$), 86.4 (C-1), 75.2 (C-3), 73.2 (C-4), 71.5 (CH$_{2Bn}$), 71.0, 70.9 (2C, C-2, C-3''), 68.3 (C-3'), 67.8 (C-5), 53.1 (CH$_{2AZMB}$), 41.8 (C-2'), 38.9 (C-2''), 36.7-22.8 (12C, 12 x CH$_2$), 21.3 (CH$_{3STol}$), 17.7 (C-6), 14.2 (2C, 2 × CH$_3$); HRMS (ESI-TOF) *m/z* [M + NH$_4$]$^+$ calcd for $C_{48}H_{69}N_4O_9S$ 877.4780; found 877.4756; *m/z* [M + Na]$^+$ calcd for $C_{48}H_{65}NaN_3O_9S$ 882.4334; found 882.4313.

Macrolide 35.

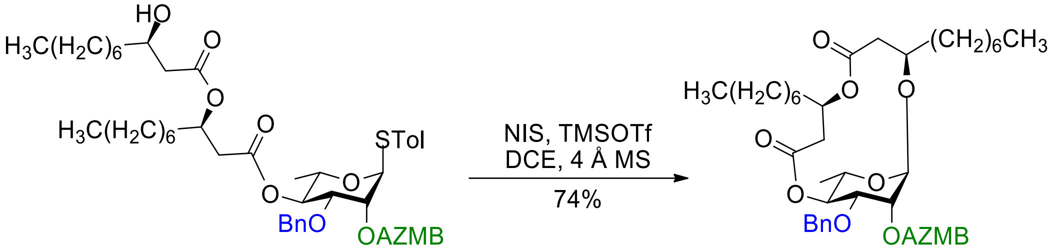

Alcohol **34** (101 mg, 0.116 mmol, 1.0 equiv) and NIS (42 mg, 0.19 mmol, 1.6 equiv) were dried under high vacuum for 1 h. Activated 4 Å MS (400 mg, 4 mg•mg^{-1} of alcohol **39**) and anhydrous DCE (11.6 mL) were added and the suspension was stirred under an Ar atmosphere for 1 h. The mixture was cooled to 0 °C and TMSOTf (8 μL, 0.05 mmol, 0.4 equiv) was added. The suspension was stirred at 0 °C for 1 h, quenched with Et_3N, and filtered over Celite. The solvents were evaporated under reduced pressure and the residue was purified by silica gel flash chromatography (Hex/EtOAc 9:1) to give macrolactone **35** (63 mg, 74%) as a colorless oil: R_f 0.70 (Hex/EtOAc 7:3); $[\alpha]^{20}_{D}$ –50 (*c* 0.6, $CHCl_3$); ^{1}H NMR (600 MHz, $CDCl_3$) δ (ppm) 8.11-8.10 (m, 1H, CH_{AZMB}), 7.60-7.57 (m, 1H, CH_{AZMB}), 7.51-7.50 (m, 1H, CH_{AZMB}), 7.40-7.37 (m, 1H, CH_{AZMB}), 7.21-7.19 (m, 3H, 3 × CH_{Bn}), 7.16-7.13 (m, 2H, 2 × CH_{Bn}), 5.43-5.39 (m, 1H, H-3’’), 5.20 (s, 1H, H-1), 5.15 (d, J = 5.7 Hz, 1H, H-2), 4.81 (d, J = 4.0 Hz, 1H, H-4), 4.75 (d, J = 14.6 Hz, 1H, CHH$_{AZMB}$), 4.70 (d, J = 14.6 Hz, 1H, CHH_{AZMB}), 4.63 (d, J = 11.8 Hz, 1H, CHH$_{Bn}$), 4.49 (d, J = 11.8 Hz, 1H, CHH_{Bn}), 4.29 (br t, J = 4.9 Hz, 1H, H-3), 4.25-4.21 (m, 1H, H-3’), 3.96 (q, J = 6.8 Hz, 1H, H-5), 2.57 (dd, $J_{2a''-2b''}$ = 12.7 Hz, $J_{2a''-3''}$ = 11.1 Hz, 1H, H-2a’’), 2.50-2.41 (m, 3H, H-2b’’, H-2a’, H-2b’), 1.69-1.53 (m, 4H, 2 × CH_2), 1.47 (d, J = 6.8 Hz, 3H, H-6), 1.31-1.21 (m, 20H, 10 × CH_2), 0.88 (t, J = 7.0 Hz, 3H, CH_3), 0.83 (t, J = 7.0 Hz, 3H, CH_3); ^{13}C NMR (150 MHz, $CDCl_3$) δ (ppm) 172.8, 170.9 (2C, C-1’, C-1’’), 165.6 (COOR$_{AZMB}$), 137.9-128.1 (12C, 3 × C_{Ar}, 9 × CH$_{Ar}$), 94.3 (C-1, $^1J_{C1-H1}$ = 173 Hz), 73.5 (CH$_{2Bn}$), 73.2 (C-2), 72.2 (C-3), 71.5, 71.4, 71.1 (3C, C-3’, C-3’’, C-

4), 68.4 (C-5), 53.1 (CH_{2AZMB}), 41.4 (C-2’’), 40.3 (C-2’), 35.5-22.7 (12C, 12 × CH_2), 20.9 (C-6), 14.22 (CH_3), 14.17 (CH_3); HRMS (ESI-TOF) *m/z* $[M + Na]^+$ calcd for $C_{41}H_{57}NaN_3O_9$ 758.3987; found 758.4010; *m/z* $[M + K]^+$ calcd for $C_{41}H_{57}KN_3O_9$ 774.3726; found 774.3742.

Macrolide S16.

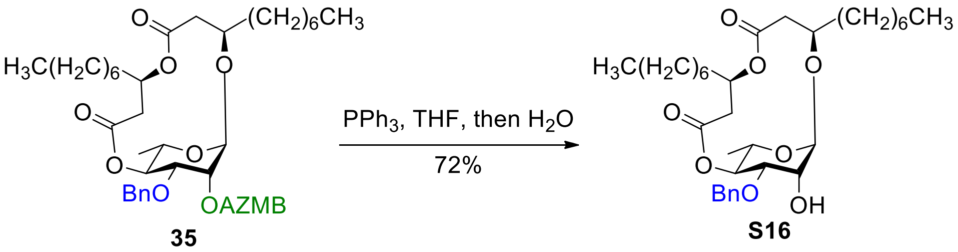

PPh_3 (30 mg, 0.12 mmol, 1.6 equiv) was added to a solution of compound **35** (53 mg, 0.072 mmol, 1.0 equiv) in anhydrous THF (2.2 mL). The mixture was stirred at 60 °C for 2 h under an Ar atmosphere, after which H_2O (0.3 mL) was added. The solution was stirred at 60 °C for 4 h and co-evaporated with toluene. The residue was purified by silica gel flash chromatography (Hex/EtOAc 95:5 to 9:1) to give compound **S16** (30 mg, 72%) as a colorless oil: R_f 0.61 (Hex/EtOAc 7:3); $[\alpha]^{20}_{D}$ –90 (c 0.2, $CHCl_3$); ^1H NMR (600 MHz, $CDCl_3$) δ (ppm) 7.38-7.31 (m, 5H, 5 × CH_{Bn}), 5.40-5.36 (m, 1H, H-3’’), 4.94 (s, 1H, H-1), 4.82 (d, J = 3.8 Hz, 1H, H-4), 4.77 (d, J = 11.4 Hz, 1H, CHH_{Bn}), 4.55 (d, J = 11.4 Hz, 1H, CHH_{Bn}), 4.19-4.14 (m, 2H, H-3’), 3.93 (dd, J = 6.0 Hz, 3.9 Hz, 1H, H-3), 3.87-3.81 (m, 2H, H-2, H-5), 3.17 (d, J = 10.9 Hz, 1H, OH), 2.52 (dd, $J_{2a''-2b''}$ = 12.8 Hz, $J_{2a''-3''}$ = 11.0 Hz, 1H, H-2a’’), 2.47 (dd, $J_{2a'-2b'}$ = 13.0 Hz, $J_{2a'-3'}$ = 3.2 Hz, 1H, H-2a’), 2.43-2.40 (m, 2H, H-2b’, H-2b’’), 1.71-1.44 (m, 4H, 2 × CH_2), 1.40 (d, J = 6.8 Hz, 3H, H-6), 1.30-1.26 (m, 20H, 10 × CH_2), 0.88 (t, J = 6.7 Hz, 6H, 2 × CH_3); ^{13}C NMR (150 MHz, $CDCl_3$) δ (ppm) 172.8, 170.9 (2C, C-1’, C-1’’), 136.8 (C_{Bn}), 128.9 (2C, 2 × CH_{Bn}), 128.5 (C_{Bn}), 128.3 (2C, 2 × CH_{Bn}), 97.8 (C-1), 73.8 (C-3), 73.6 (CH_{2Bn}), 71.4, 71.0, 70.8 (3C, C-3’, C-3’’, C-4), 68.4 (C-2), 67.5 (C-5), 41.4 (C-2’’), 40.4 (C-2’), 35.4-22.8 (12C, 12 × CH_2), 20.9 (C-6), 14.2 (2C, 2 × CH_3); HRMS (ESI-TOF) m/z $[M + Na]^+$ calcd for $C_{33}H_{52}NaO_8$ 599.3554; found 599.3564; m/z $[M + K]^+$ calcd for $C_{33}H_{52}KO_8$ 615.3294; found 615.3302.

(1→4)-Macrolactonized Rhamnolipid (4).

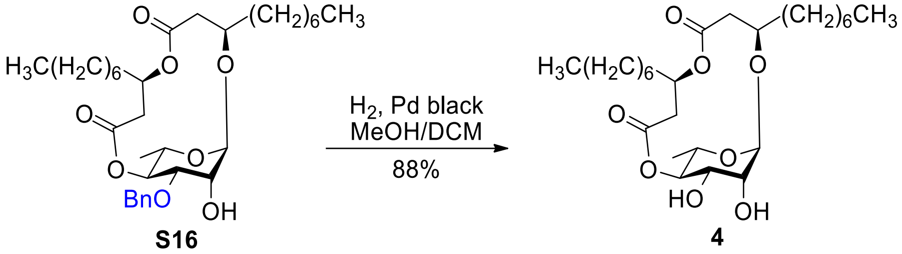

Pd black (41 mg, 1 mg•mg^{-1} of alcohol **S16**) was added to a solution of alcohol **S16** (41 mg, 0.072 mmol, 1.0 equiv) in DCE (0.7 mL) and MeOH (1.4 mL). The suspension was stirred under H_2 atmosphere at 40 °C for 16 h, filtered over Celite, and evaporated under reduced pressure. The residue was purified by silica gel flash chromatography (Hex/EtOAc 9:1 to 6:4) to give diol **4** (31 mg, 88%) as a colorless oil: R_f 0.28 (Hex/EtOAc 6:4); $[\alpha]^{20}_D$ –71 (*c* 0.5, $CHCl_3$); ^{1}H NMR (600 MHz, $CDCl_3$) δ (ppm) 5.43-5.39 (m, 1H, H-3''), 4.97 (s, 1H, H-1), 4.73 (d, *J* = 4.1 Hz, 1H, H-4), 4.17-4.13 (m, 1H, H-3'), 3.88-3.84 (m, 2H, H-2, H-5), 2.80 (d, *J* = 8.4 Hz, 1H, O*H*), 2.54-2.41 (m, 5H, O*H*, H-2a'', H-2b'', H-2a', H-2b'), 1.73-1.47 (m, 4H, 2 × CH_2), 1.43 (d, *J* = 6.9 Hz, 3H, H-6), 1.30-1.26 (m, 20H, 10 × CH_2), 0.89-0.86 (m, 6H, 2 × CH_3); ^{13}C NMR (150 MHz, $CDCl_3$) δ (ppm) 172.7, 170.8 (2C, C-1', C-1''), 97.8 (C-1), 73.9 (C-4), 71.6 (C-3''), 71.2 (C-3'), 69.0, 68.2, 67.8 (3C, C-2, C-3, C-5), 41.3, 40.4 (2C, C-2', C-2''), 35.4-22.8 (12C, 12 × *C*H_2), 21.4 (C-6), 14.2 (2C, 2 × *C*H_3); HRMS (ESI-TOF) *m/z* $[M + NH_4]^+$ calcd for $C_{26}H_{50}NO_8$ 504.3531; found 504.3525; *m/z* $[M + Na]^+$ calcd for $C_{26}H_{46}NaO_8$ 509.3085; found 509.3078. Analytical HPLC analysis was performed using method A (39.4 min.).

***para*-Methylphenyl 3-*O*-Benzyl-2-*O*-(*R*)-3-(((*R*)-3-((*tert*-butyldimethylsilyl)oxy)decanoyl)oxy)decanoyl-4-*O*-levulinoyl-1-thio-*α*-L-rhamnopyranoside (27).**

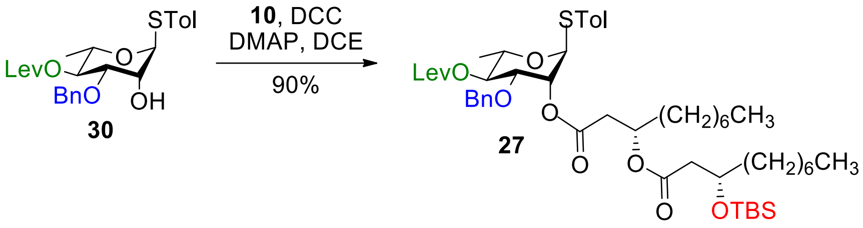

DMAP (5 mg, 0.04 mmol, 0.1 equiv) and DCC (244 mg, 1.18 mmol, 3.0 equiv) were added to a solution of alcohol **30** (181 mg, 0.394 mmol, 1.0 equiv) and acid **10** (261 mg, 0.551 mmol, 1.4 equiv) in anhydrous DCE (4.7 mL). The suspension was refluxed for 1 h under an Ar atmosphere then cooled at 0 °C, filtered over Celite, and the solvents were evaporated under reduced pressure. The residue was purified by silica gel flash chromatography (Hex/EtOAc 97:3 to 92:8) to give compound **27** (323 mg, 90%) as a colorless oil: R_f 0.46 (Hex/EtOAc 8:2); $[\alpha]^{20}_{D}$ –20 (*c* 0.6, $CHCl_3$); ^{1}H NMR (600 MHz, $CDCl_3$) δ (ppm) 7.35-7.32 (m, 4H, 2 × CH_{STol}, 2 × CH_{Bn}), 7.30-7.28 (m, 3H, 3 × CH_{Bn}), 7.12-7.11 (m, 2H, 2 × CH_{STol}), 5.60 (dd, $J_{2\text{-}3}$ = 3.2 Hz, $J_{2\text{-}1}$ = 1.7 Hz, 1H, H-2), 5.33 (d, *J* = 1.5 Hz, 1H, H-1), 5.18-5.14 (m, 1H, H-3''), 5.04 (t, *J* = 9.7 Hz, 1H, H-4), 4.64 (d, *J* = 12.0 Hz, 1H, CHH_{Bn}), 4.43 (d, *J* = 12.0 Hz, 1H, CHH_{Bn}), 4.26 (dq, $J_{5\text{-}4}$ = 9.6 Hz, $J_{5\text{-}6}$ = 6.1 Hz, 1H, H-5), 4.07-4.03 (m, 1H, H-/'), 3.79 (dd, $J_{3\text{-}4}$ = 9.7 Hz, $J_{3\text{-}2}$ = 3.3 Hz, 1H, H-3), 2.81-2.76 (m, 1H, CHH_{Lev}), 2.71-2.56 (m, 4H, H-2a'', H-2b'', CHH_{Lev}, CHH_{Lev}), 2.50-2.44 (m, 1H, CHH_{Lev}), 2.42 (dd, $J_{2a'\text{-}2b'}$ = 14.8 Hz, $J_{2a'\text{-}3'}$ = 5.9 Hz, 1H, H-2a'), 2.37 (dd, $J_{2b'\text{-}2a'}$ = 14.8 Hz, $J_{2b'\text{-}3'}$ = 6.8 Hz, 1H, H-2b'), 2.32 (s, 3H, CH_{3STol}), 2.17 (s, 3H, CH_{3Lev}), 1.59-1.21 (m, 27H, 12 × CH_2, H-6), 0.90-0.82 (m, 15H, 2 × CH_3, C(CH_3)$_{3TBS}$), 0.03 (s, 3H, CH_{3TBS}), 0.02 (s, 3H, CH_{3TBS}); ^{13}C NMR (150 MHz, $CDCl_3$) δ (ppm) 206.5 (CO_{Lev}), 172.0, 171.1, 169.8 (3C, C-1', C-1'', $COOR_{Lev}$), 138.2 (C_{Ar}), 137.7 (C_{Ar}), 132.4-128.0 (10C, C_{Ar}, 9 × CH_{Ar}), 86.4 (C-1), 75.0 (C-3), 72.9 (C-4), 71.6 (CH_{2Bn}), 70.5, 70.3 (2C,

C-3'', C-2), 69.4 (C-3'), 67.9 (C-5), 42.9 (C-2'), 38.9 (C-2''), 38.0 (CH_{2Lev}), 37.4-22.8 (17C, 12 × CH_2, CH_{2Lev}, CH_{3Lev}, $C(CH_3)_{3TBS}$), 18.2 ($C(CH_3)_{3TBS}$), 17.5 (C-6), 14.2 (2C, 2 × CH_3), -4.5 (2C, 2 × CH_{3TBS}); HRMS (ESI-TOF) *m/z* $[M + NH_4]^+$ calcd for $C_{51}H_{84}NO_{10}SSi$ 930.5580; found 930.5605; *m/z* $[M + Na]^+$ calcd for $C_{51}H_{80}NaO_{10}SSi$ 935.5134; found 935.5159.

para-Methylphenyl 3-_O_-Benzyl-2-_O_-(_R_)-3-((_R_)-3-(hydroxydecanoyl)oxy)decanoyl-4-_O_-levulinoyl-1-thio-_α_-L-rhamnopyranoside (36).

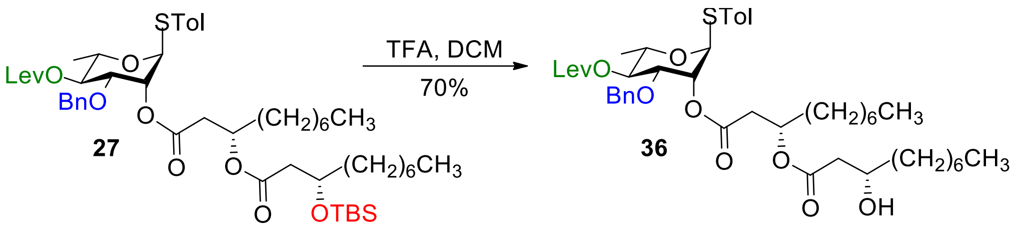

TFA (2.7 mL) was added to a solution of compound **27** (256 mg, 0.280 mmol, 1.0 equiv) in DCM (2.8 mL). The mixture was stirred at rt for 10 min and quenched with saturated aqueous $NaHCO_3$. The organic layer was dried over $MgSO_4$, filtered, and the solvents were evaporated under reduced pressure. The residue was purified by silica gel flash chromatography (Hex/EtOAc 9:1 to 8:2) to give alcohol **36** (158 mg, 70%) as a colorless oil: R_f 0.39 (Hex/EtOAc 7:3); $[\alpha]^{20}_{D}$ –26 (*c* 0.9, $CHCl_3$); ^{1}H NMR (600 MHz, $CDCl_3$) δ (ppm) 7.36-7.32 (m, 4H, 2 × CH_{Bn}, 2 x CH_{STol}), 7.30-7.28 (m, 3H, 3 × CH_{Bn}), 7.12-7.11 (m, 2H, 2 × CH_{STol}), 5.60 (dd, $J_{2\text{-}3}$ = 3.2 Hz, $J_{2\text{-}1}$ = 1.7 Hz, 1H, H-2), 5.35 (d, *J* = 1.4 Hz, 1H, H-1), 5.25-5.21 (m, 1H, H-3’’), 5.04 (t, *J* = 9.8 Hz, 1H, H-4), 4.64 (d, *J* = 12.0 Hz, 1H, C*H*H_{Bn}), 4.43 (d, *J* = 12.0 Hz, 1H, CHH_{Bn}), 4.26 (dq, $J_{5\text{-}4}$ = 9.9 Hz, $J_{5\text{-}6}$ = 6.2 Hz, 1H, H-5), 3.95-3.91 (m, 1H, H-3’), 3.79 (dd, $J_{3\text{-}4}$ = 9.7 Hz, $J_{3\text{-}2}$= 3.3 Hz, 1H, H-3), 2.79 (ddd, *J* = 18.3 Hz, *J* = 8.0 Hz, *J* = 5.8 Hz, 1H, C*H*H_{Lev}), 2.69-2.64 (m, 3H, CHH_{Lev}, H-2a’’, H-2b’’), 2.58 (ddd, *J* = 17.2 Hz, *J* = 8.0 Hz, *J* = 5.5 Hz, 1H, C*H*H_{Lev}), 2.48 (dt, *J* = 17.2 Hz, *J* = 6.2 Hz, 1H, CHH_{Lev}), 2.37 (dd, $J_{2a'\text{-}2b'}$ = 15.9 Hz, $J_{2a'\text{-}3'}$ = 3.3 Hz, 1H, H-2a’), 2.34-2.29 (m, 4H, CH_{3STol}, H-2b’), 2.18 (s, 3H, CH_{3Lev}), 1.59-1.21 (m, 27H, H-6, 12 × CH_2), 0.89-0.86 (m, 6H, 2 × CH_3); ^{13}C NMR (150 MHz, $CDCl_3$) δ (ppm) 206.5 (CO_{Lev}), 172.6, 172.0, 170.0 (3C, $COOR_{Lev}$, C-1’, C-1’’), 138.2 (C_{Ar}), 137.6 (C_{Ar}), 132.4-128.0 (10C, C_{Ar}, 9 × CH_{Ar}), 86.3 (C-1), 74.9 (C-3), 72.9 (C-4), 71.6 (CH_{2Bn}), 70.9 (C-3’’), 70.4 (C-2), 68.3 (C-3’), 67.9 (C-5), 41.8 (C-2’), 39.1 (C-2’’), 38.0 (CH_{2Lev}), 36.7-22.7 (14C,

12 x CH_2, CH_{2Lev}, CH_{3Lev}), 17.4 (C-6), 14.2 (2C, 2 × CH_3); HRMS (ESI-TOF) *m/z* $[M + Na]^+$ calcd for $C_{45}H_{66}NaO_{10}S$ 821.4269; found 821.4270.

Macrolide 37.

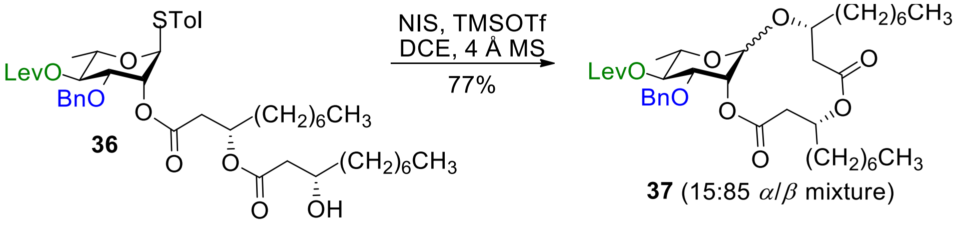

Alcohol **36** (120 mg, 0.150 mmol, 1.0 equiv) and NIS (54 mg, 0.24 mmol, 1.6 equiv) were dried under high vacuum for 1 h. Activated 4 Å MS (480 mg, 4 mg•mg^{-1} of alcohol **36**) and anhydrous DCE (15 mL) were added and the suspension was stirred under an Ar atmosphere for 1 h. The mixture was cooled to –10 °C and TMSOTf (11 μL, 0.06 mmol, 0.4 equiv) was added. The suspension was stirred at –10 °C for 35 min, quenched with Et_3N, and filtered over Celite. The solvents were evaporated under reduced pressure and the residue was purified by silica gel flash chromatography (Hex/EtOAc 9:1 to 8:2) to give macrolactone **37** (78 mg, 77%) as a 15:85 α/β mixture as a colorless oil: R_f 0.20 (Hex/EtOAc 7:3); ^{1}H NMR (600 MHz, $CDCl_3$) δ (ppm) (data for major β-anomer) 7.37-7.27 (m, 5H, 5 × CH_{Bn}), 5.55-5.49 (m, 1H, H-3’’), 5.42 (dd, J_{2-3} = 3.7 Hz, J_{2-1} = 1.9 Hz, 1H, H-2), 4.97 (t, J = 9.0 Hz, 1H, H-4), 4.78 (d, J = 12.2 Hz, 1H, CHH$_{Bn}$), 4.67 (d, J = 1.9 Hz, 1H, H-1), 4.50 (d, J = 12.2 Hz, 1H, CHH_{Bn}), 3.92-3.88 (m, 1H, H-3’), 3.58 (dd, J_{3-4} = 9.2 Hz, J_{3-2} = 3.7 Hz, 1H, H-3), 3.49 (dq, J_{5-4} = 8.7 Hz, J_{5-6} = 6.3 Hz, 1H, H-5), 2.80-2.75 (m, 1H, CHH$_{Lev}$), 2.72-2.66 (m, 2H, CHH_{Lev}, H-2a’’), 2.60-2.49 (m, 4H, CH_{2Lev}, H-2b’’, H-2a’), 2.38 (dd, $J_{2b'-2a'}$ = 12.3 Hz, $J_{2b'-3'}$ = 2.2 Hz, 1H, H-2b’), 2.17 (s, 3H, CH_{3Lev}), 1.73-1.26 (m, 27H, 12 × CH_2, H-6), 0.88 (t, J = 4.9 Hz, 6H, 2 × CH_3); ^{13}C NMR (150 MHz, $CDCl_3$) δ (ppm) (data for major β-anomer) 206.5 (CO_{Lev}), 172.1, 171.9, 171.8 (3C, C-1’, C-1’’, $COOR_{Lev}$), 137.8 (C_{Bn}), 128.5 (2C, 2 × CH_{Bn}), 128.1 (2C, 2 × CH_{Bn}), 127.9 (C_{Bn}), 96.1 (C-1, $^1J_{C1-H1}$ = 162 Hz), 76.6 (C-3’), 75.8 (C-3), 72.32, 72.30 (2C, C-3’’, C-4), 71.4 (CH_{2Bn}), 70.9 (C-5), 68.7 (C-2), 41.5, 41.3 (2C, C-2’, C-2’’), 38.0 (CH_{2Lev}), 34.7-22.7 (14C, 12 × CH_2, CH_{2Lev}, CH_{3Lev}), 18.1 (C-6), 14.2 (2 × CH_3); HRMS (ESI-

TOF) *m/z* [M + NH_4]$^+$ calcd for $C_{38}H_{62}NO_{10}$ 692.4368; found 692.4374; *m/z* [M + Na]$^+$ calcd for $C_{38}H_{58}NaO_{10}$ 697.3922; found 697.3928.

Macrolides S17*β* and S17*α*.

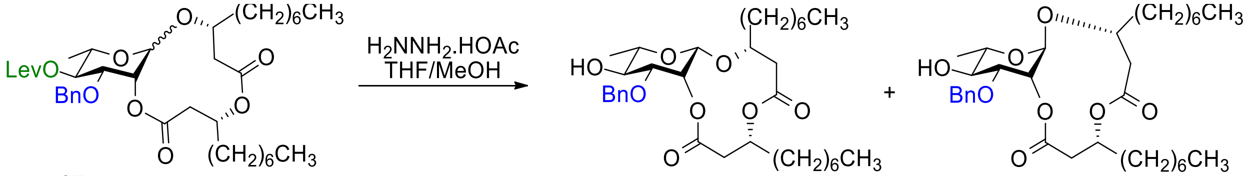

37 (15:85 α/β mixture) **S17**β (74%) **S17**α (21%)

To a solution of compound **37** (66 mg, 0.098 mmol, 1.0 equiv) in anhydrous THF/MeOH (10:1 *v/v*, 6.9 mL) was slowly added a solution of hydrazine monohydrate (67 μL, 1.4 mmol, 14 equiv) and HOAc (167 μL) in anhydrous THF/MeOH (5:1 *v/v*, 1.4 mL). The solution was stirred under an Ar atmosphere for 1 h until a white solid was formed and TLC showed complete conversion. The suspension was co-evaporated with toluene and the anomers were rapidly purified by silica gel flash chromatography (Tol/EtOAc 95:5 to 9:1) to give macrolides **S17*β*** (β-anomer, 42 mg, 74%) and **S17*α*** (α-anomer, 11.6 mg, 21%, contaminated with 15% of compound **37**) as colorless oils.

Data for compound **S17*β***: R_f 0.50 (Tol/EtOAc 8:2); $[\alpha]^{20}_D$ +10 (*c* 0.6, $CHCl_3$); ^1H NMR (600 MHz, $CDCl_3$) δ (ppm) 7.35-7.30 (m, 5H, 5 × CH_{Bn}), 5.56-5.52 (m, 1H, H-3’’), 5.43 (dd, $J_{2\text{-}3}$ = 3.1 Hz, $J_{2\text{-}1}$ = 1.4 Hz, 1H, H-2), 4.93 (d, J = 11.0 Hz, 1H, CHH$_{Bn}$), 4.65 (d, J = 1.3 Hz, 1H, H-1), 4.43 (d, J = 11.0 Hz, 1H, CHH_{Bn}), 3.89-3.85 (m, 1H, H-3’), 3.50 (t, J = 9.3 Hz, 1H, H-4), 3.42 (dd, $J_{3\text{-}4}$ = 9.5 Hz, $J_{3\text{-}2}$ = 3.5 Hz, 1H, H-3), 3.36 (dq, $J_{5\text{-}4}$ = 9.0 Hz, $J_{5\text{-}6}$ = 6.1 Hz, 1H, H-5), 2.69 (dd, $J_{2a''\text{-}2b''}$ = 13.2 Hz, $J_{2a''\text{-}3''}$ = 12.1 Hz, 1H, H-2a’’), 2.56-2.52 (m, 2H, H-2b’’, H-2a’), 2.38 (dd, $J_{2a'\text{-}2b'}$ = 12.1 Hz, $J_{2a'\text{-}3'}$ = 2.1 Hz, 1H, H-2b’), 2.32 (s, 1H, OH), 1.54-1.21 (m, 4H, 2 × CH_2), 1.37 (d, J = 6.1 Hz, 3H, H-6), 1.31-1.26 (m, 20H, 10 × CH_2), 0.89-0.87 (m, 6H, 2 × CH_3); ^{13}C NMR (150 MHz, $CDCl_3$) δ (ppm) 172.2, 172.1 (2C, C-1’, C-1’’), 137.3 (C_{Bn}), 128.8 (2C, 2 × CH$_{Bn}$), 128.6 (2C, 2 × CH$_{Bn}$), 128.3 (CH$_{Bn}$), 96.0 (C-1, $^1J_{C1\text{-}H1}$ = 156 Hz), 79.3 (C-3), 76.0 (C-3’), 72.5, 72.4 (2C, C-5, C-3’’), 71.6 (CH$_{2Bn}$), 71.0 (C-4), 68.6 (C-2), 41.4, 41.2 (2C, C-2’, C-2’’), 34.7-22.8 (12C, 12 × CH$_2$), 18.0

(C-6), 14.2 (2C, 2 × *C*H_3); HRMS (ESI-TOF) *m/z* $[M + NH_4]^+$ calcd for $C_{33}H_{56}NO_8$ 594.4000; found 594.4013; *m/z* $[M + Na]^+$ calcd for $C_{33}H_{52}NaO_8$ 599.3554; found 599.3569.

Partial data for compound **S17*α*** (contaminated with 15% of compound **43**): R_f 0.34 (Tol/EtOAc 8:2); HRMS (ESI-TOF) *m/z* $[M + NH_4]^+$ calcd for $C_{33}H_{56}NO_8$ 594.4000; found 594.3999; *m/z* $[M + Na]^+$ calcd for $C_{33}H_{52}NaO_8$ 599.3554; found 599.3556.

(1→2)-Macrolactonized Rhamnolipid 5*β*.

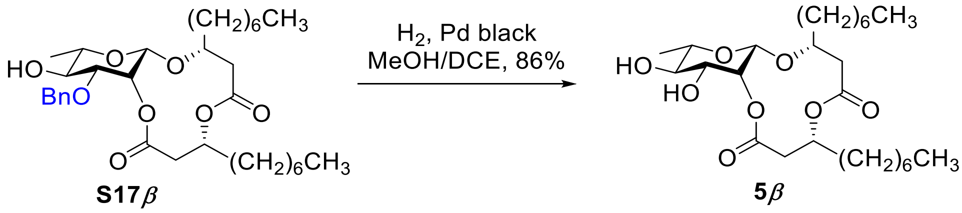

To a solution of **S17*β*** (12.3 mg, 0.0260 mmol, 1.0 equiv) in MeOH (0.5 mL) and DCE (0.3 mL) was added Pd black (12.3 mg, 1 mg•mg^{-1} of alcohol **S17*β***). The suspension was stirred at 40 °C under an atmosphere of H_2 for 16 h then filtered over Celite. The solvents were evaporated under reduced pressure and the residue was purified by silica gel flash chromatography (Hex/EtOAc 8:2 to 1:1) to give macrolactone **5*β*** (9 mg, 86%) as a colorless oil: R_f 0.38 (DCM/MeOH 95:5); $[\alpha]^{20}_{D}$ –22 (*c* 1.3, $CHCl_3$); ^{1}H NMR (600 MHz, $CDCl_3$) δ (ppm) 5.37-5.33 (m, 1H, H-3''), 4.96 (dd, J = 1.5 Hz, 3.3 Hz, 1H, H-2), 4.68 (d, J = 1.0 Hz, 1H, H-1), 3.92-3.89 (m, 1H, H-3'), 3.71-3.69 (m, 1H, H-3), 3.45-3.41 (m, 2H, H-4, H-5), 2.68 (t, J = 11.7 Hz, 1H, H-2a''), 2.52 (t, J = 12.6 Hz, 1H, H-2a'), 2.47 (dd, $J_{2b''\text{-}2a''}$ = 12.1 Hz, $J_{2b''\text{-}3''}$ = 1.7 Hz, 1H, H-2b''), 2.38 (dd, $J_{2b'\text{-}2a'}$ = 12.9 Hz, $J_{2b'\text{-}3'}$ = 1.9 Hz, 1H, H-2b'), 1.72-1.54 (m, 4H, 2 × CH_2), 1.39 (d, J = 5.1 Hz, 3H, H-6), 1.30-1.26 (m, 20H, 10 × CH_2), 0.89-0.87 (m, 6H, 2 × CH_3); ^{13}C NMR (150 MHz, $CDCl_3$) δ (ppm) 174.3, 172.9 (2C, C-1', C-1''), 97.2 (C-1), 76.5 (C-3'), 73.8 (C-2), 73.1, 73.0, 72.8 (3C, C-3, C-4, C-5), 72.3 (C-3''), 41.13, 41.12 (2C, C-2', C-2''), 35.0-22.7 (12C, 12 × *C*H_2), 18.1 (C-6), 14.2 (2C, 2 × *C*H_3); HRMS (ESI-TOF) *m/z* $[M + NH_4]^+$ calcd for $C_{26}H_{50}NO_8$ 504.3531; found 504.3534; *m/z* $[M + Na]^+$ calcd for $C_{26}H_{46}NaO_8$ 509.3085; found 509.3087. Analytical HPLC analysis was performed using method B (32.1 min.).

(1→2)-Macrolactonized Rhamnolipid 5*α*.

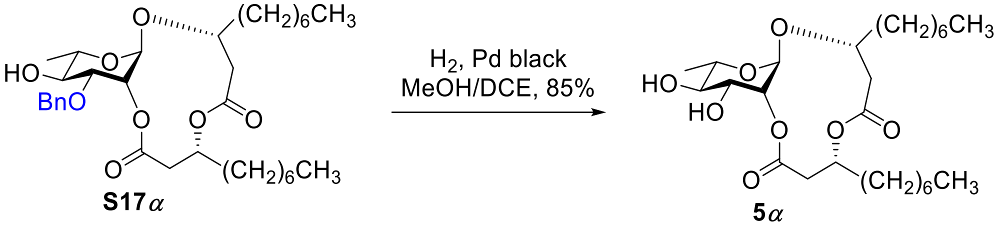

To a solution of **S17*α*** (8.8 mg, 0.015 mmol, 1.0 equiv) in MeOH (0.3 mL) and DCE (0.2 mL) was added Pd black (8.8 mg, 1 mg•mg^{-1} of alcohol **S17*α***). The suspension was stirred at 40 °C under an atmosphere of H_2 for 16 h then filtered over Celite. The solvents were evaporated under reduced pressure and the residue was purified by silica gel flash chromatography (Hex/EtOAc 8:2 to 6:4) to give macrolactone **5*α*** (6.3 mg, 85%) as a colorless oil: R_f 0.36 (DCM/MeOH 95:5);$[\alpha]^{20}_{D}$ –54 (*c* 0.5, $CHCl_3$); ^{1}H NMR (600 MHz, $CDCl_3$) δ (ppm) 5.37-5.35 (m, 1H, H-3''), 5.15 (dd, $J_{2\text{-}1}$ = 6.8 Hz, $J_{2\text{-}3}$ = 2.6 Hz, 1H, H-2), 5.04 (d, *J* = 6.8 Hz, 1H, H-1), 4.26-4.24 (m, 1H, H-3'), 3.88 (br s, 1H, H-5), 3.83 (p, *J* = 6.5 Hz, 1H, H-5), 3.56 (d, *J* = 6.8 Hz, 1H, H-4), 2.57-2.52 (m, 2H, H-2a'', H-2a'), 2.44 (dd, $J_{2b''\text{-}2a''}$ = 11.8 Hz, $J_{2b''\text{-}3''}$ = 2.7 Hz, 1H, H-2b''), 2.33 (dd, $J_{2b'\text{-}2a'}$ = 14.5 Hz, $J_{2b'\text{-}3'}$ = 3.0 Hz, H-2b'), 1.79-1.33 (m, 6H, 3 × CH_2), 1.30 (d, *J* = 6.4 Hz, 3H, H-6), 1.22-1.19 (m, 18H, 9 × CH_2), 0.81 (t, *J* = 6.9 Hz, 6H, 2 × CH_3); ^{13}C NMR (150 MHz, $CDCl_3$) δ (ppm) 170.9, 170.2 (2C, C-1', C-1''), 91.9 (C-1), 76.8 (C-4), 76.3 (C-3'), 73.8 (C-3), 72.7 (C-2), 71.4 (C-3''), 71.0 (C-5), 41.1 (C-2''), 38.8 (C-2'), 35.2-22.8 (12C, 12 × *C*H_2), 19.1 (C-6), 14.2 (2 × *C*H_3); HRMS (ESI-TOF) *m/z* $[M + NH_4]^+$ calcd for $C_{26}H_{50}NO_8$ 504.3531; found 504.3532; *m/z* $[M + Na]^+$ calcd for $C_{26}H_{46}NaO_8$ 509.3085; found 509.3086. Analytical HPLC analysis was performed using method B (47.3 min.).

***para*-Methylphenyl 2-*O*-*ortho*-(Azidomethyl)benzoyl-4-*O*-levulinoyl-1-thio-*α*-L-rhamnopyranoside (31).**

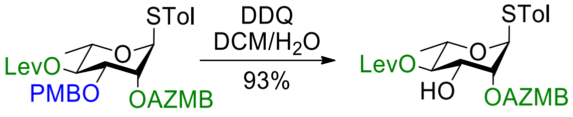

To a solution of rhamnoside **25** (248 mg, 0.383 mmol, 1.0 equiv) in DCM/H_2O (10:1, 8.4 mL) was added DDQ (174 mg, 0.766 mmol, 2.0 equiv). The reaction mixture was stirred at rt for 4 h then quenched with saturated aqueous $NaHCO_3$. The aqueous phase was extracted with DCM (3×) and the combined organic layers were washed with saturated aqueous $NaHCO_3$ and brine. The organic phase was dried over $MgSO_4$, filtered, and the solvents were evaporated under reduced pressure. The residue was purified by silica gel flash chromatography (Hex:EtOAc 9:1 to 65:35) to give alcohol **31** (187 mg, 93%) as a colorless oil: R_f 0.42 (Hex/EtOAc 1:1); $[\alpha]^{20}_D$ –99 (*c* 0.3, $CHCl_3$); ^{1}H NMR (600 MHz, $CDCl_3$) δ (ppm) 8.06-8.04 (m, 1H, CH_{AZMB}), 7.58-7.55 (m, 1H, CH_{AZMB}), 7.47-7.46 (m, 1H, CH_{AZMB}), 7.44-7.42 (m, 1H, CH_{AZMB}), 7.39-7.38 (m, 2H, 2 × CH_{STol}), 7.14-7.12 (m, 2H, 2 × CH_{STol}), 5.62 (dd, J_{2-3} = 3.4 Hz, J_{2-1} = 1.5 Hz, 1H, H-2), 5.54 (d, *J* = 1.3 Hz, 1H, H-1), 5.08 (t, *J* = 9.8 Hz, 1H, H-4), 4.83 (d, *J* = 14.4 Hz, 1H, CHH_{AZMB}), 4.70 (d, *J* = 14.4 Hz, 1H, CHH_{AZMB}), 4.40 (dq, J_{5-4} = 9.8 Hz, J_{5-6} = 6.2 Hz, 1H, H-5), 4.18 (dd, J_{3-4} = 9.8 Hz, J_{3-2} = 3.3 Hz, 1H, H-3), 3.22 (br s, 1H, O*H*), 2.89-2.78 (m, 2H, CH_{2Lev}), 2.67-2.61 (m, 2H, CH_{2Lev}), 2.33 (s, 3H, CH_{3STol}), 2.20 (s, 3H, CH_{3Lev}), 1.28 (d, *J* = 6.2 Hz, 3H, H-6); ^{13}C NMR (150 MHz, $CDCl_3$) δ (ppm) 207.3 (CO_{Lev}), 173.2 ($COOR_{Lev}$), 166.2 ($COOR_{AZMB}$), 138.3 (C_{Ar}), 137.1 (C_{Ar}), 133.3-128.5 (10C, 2 × C_{Ar}, 8 × CH_{Ar}), 86.2 (C-1), 75.4-75.2 (C-2, C-4), 69.3 (C-3), 67.4 (C-5), 53.6 (CH_{2AZMB}), 38.3 (CH_{2Lev}), 29.9 (CH_{3Lev}), 28.3 (CH_{2Lev}), 21.3 (CH_{3STol}), 17.5 (C-6); HRMS (ESI-TOF) *m/z* [M + NH_4]$^+$ calcd for $C_{26}H_{33}N_4O_7S$ 545.2065; found 545.2070; *m/z* [M + Na]$^+$ calcd for $C_{26}H_{29}NaN_3O_7S$ 550.1618; found 550.1619.

***para*-Methylphenyl 2-*O*-*ortho*-(Azidomethyl)benzoyl-3-*O*-(*R*)-3-(((*R*)-3-((*tert*-butyldimethylsilyl)oxy)decanoyl)oxy)decanoyl-4-*O*-levulinoyl-1-thio-*α*-L-rhamnopyranoside (28).**

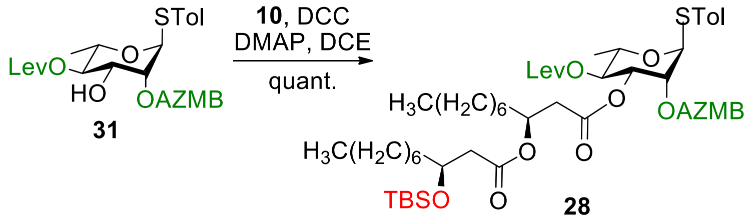

DMAP (4 mg, 0.03 mmol, 0.1 equiv) and DCC (206 mg, 0.996 mmol, 3.0 equiv) were successively added to a solution of alcohol **31** (175 mg, 0.332 mmol, 1.0 equiv) and acid **10** (220 mg, 0.465 mmol, 1.4 equiv) in anhydrous DCE (4 mL). The reaction mixture was refluxed for 2 h under an Ar atmosphere then cooled at 0 °C, filtered over Celite, and the solvents were evaporated under reduced pressure. The residue was purified by silica gel flash chromatography (Hex/EtOAc 97:3 to 87:13) to give compound **28** (326 mg, quant.) as a colorless oil: R_f 0.44 (Hex/EtOAc 8:2); $[\alpha]^{20}_D$ –3 (*c* 0.4, $CHCl_3$); ^{1}H NMR (600 MHz, $CDCl_3$) *δ* (ppm) 8.06-8.05 (m, 1H, CH_{AZMB}), 7.60-7.57 (m, 1H, CH_{AZMB}), 7.52-7.51 (m, 1H, CH_{AZMB}), 7.45-7.42 (m, 1H, CH_{AZMB}), 7.39-7.38 (m, 2H, 2 × CH_{STol}), 7.14-7.13 (m, 2H, 2 × CH_{STol}), 5.71 (dd, J_{2-3} = 3.3 Hz, J_{2-1} = 1.6 Hz, 1H, H-2), 5.48 (d, *J* = 1.3 Hz, 1H, H-1), 5.40 (dd, J_{3-4} = 10.1 Hz, J_{3-2} = 3.3 Hz, 1H, H-3), 5.28 (t, *J* = 9.9 Hz, 1H, H-4), 5.14-5.10 (m, 1H, H-3''), 4.82 (d, *J* = 14.7 Hz, 1H, CHH_{AZMB}), 4.79 (d, *J* = 14.7 Hz, 1H, CHH_{AZMB}), 4.46 (dq, J_{5-4} = 12.4 Hz, J_{5-6} = 6.2 Hz, 1H, H-5), 4.08-4.04 (m, 1H, H-3'), 2.82-2.71 (m, 2H, CH_{2Lev}), 2.57-2.55 (m, 4H, CH_{2Lev}, H-2a'', H-2b''), 2.45 (dd, $J_{2a'-2b'}$ = 14.8 Hz, $J_{2a'-3'}$ = 5.8 Hz, 1H, H-2a'), 2.37 (d, $J_{2b'-2a'}$ = 14.8 Hz, $J_{2b'-3'}$ = 6.9 Hz, 1H, H-2b'), 2.33 (s, 3H, CH_{3STol}), 2.18 (s, 3H, CH_{3Lev}), 1.56-1.19 (m, 27H, 12 × CH_2, H-6), 0.88-0.85 (m, 15H, 2 × CH_3, $C(CH_3)_{3TBS}$), 0.06 (s, 3H, CH_{3TBS}), 0.04 (s, 3H, CH_{3TBS}); ^{13}C NMR (150 MHz, $CDCl_3$) *δ* (ppm) 206.3 (CO_{Lev}), 172.0, 171.1, 169.6 (3C, C-1', C-1'', $COOR_{Lev}$), 165.5 ($COOR_{AZMB}$), 138.4 (C_{Ar}), 137.7 (C_{Ar}),

133.4-128.0 (10C, 2 x C_{Ar}, 8 x CH_{Ar}), 86.1 (C-1), 72.5 (C-2), 71.5 (C-4), 70.2 (C-3''), 69.7, 69.4 (2C, C-3, C-3'), 67.9 (C-5), 53.2 (CH_{2AZMB}), 42.9 (C-2'), 38.8 (C-2''), 37.9 (CH_{2Lev}), 37.4-22.8 (16C, 12 × CH_2, CH_{3Lev}, C(CH_3)$_{3TBS}$), 21.3 (CH_{3STol}), 18.2 (C(CH_3)$_{3TBS}$), 17.5 (C-6), 14.3 (CH_3), 14.2 (CH_3), −4.5 (2C, 2 × CH_{3TBS}); HRMS (ESI-TOF) *m/z* $[M + H]^+$ calcd for $C_{52}H_{80}N_3O_{11}SSi$ 982.5277; found 982.5286; *m/z* $[M + Na]^+$ calcd for $C_{52}H_{79}NaN_3O_{11}SSi$ 1004.5097; found 1004.5111.

***para*-Methylphenyl 2-*O*-*ortho*-(Azidomethyl)benzoyl-3-*O*-(*R*)-3-((*R*)-3-(hydroxy decanoyl)oxy)decanoyl-4-*O*-levulinoyl-1-thio-*α*-L-rhamnopyranoside (38).**

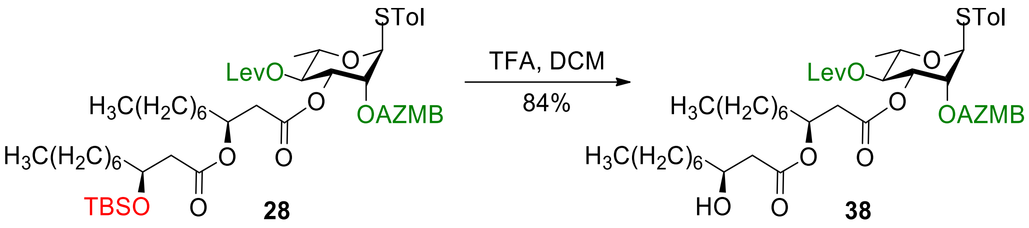

TFA (3 mL) was slowly added to a solution of compound **28** (307 mg, 0.313 mmol, 1.0 equiv) in DCM (3.1 mL). The reaction mixture was stirred at rt for 10 min then quenched with saturated aqueous $NaHCO_3$. The organic layer was dried over $MgSO_4$, filtered, and the solvents were evaporated under reduced pressure. The residue was purified by silica gel flash chromatography (Hex/EtOAc 9:1 to 7:3) to give alcohol **38** (227 mg, 84%) as a colorless oil: R_f 0.51 (Hex/EtOAc 6:4); $[\alpha]^{20}_D$ –28 (*c* 0.7, $CHCl_3$); ^{1}H NMR (600 MHz, $CDCl_3$) δ (ppm) 8.06-8.05 (m, 1H, CH_{AZMB}), 7.61-7.58 (m, 1H, CH_{AZMB}), 7.52-7.51 (m, 1H, CH_{AZMB}), 7.46-7.43 (m, 1H, CH_{AZMB}), 7.40-7.38 (m, 2H, 2 × CH_{STol}), 7.14-7.13 (m, 2H, 2 × CH_{STol}), 5.73 (dd, $J_{2\text{-}3}$ = 3.3 Hz, $J_{2\text{-}1}$ = 1.6 Hz, 1H, H-2), 5.47 (d, J = 1.4 Hz, 1H, H-1), 5.41 (dd, $J_{3\text{-}4}$ = 10.1 Hz, $J_{3\text{-}2}$ = 3.3 Hz, 1H, H-3), 5.27 (t, J = 9.9 Hz, 1H, H-4), 5.22-5.18 (m, 1H, H-3’’), 4.82 (d, J = 14.6 Hz, 1H, CHH_{AZMB}), 4.77 (d, J = 14.6 Hz, 1H, CHH_{AZMB}), 4.46 (dq, $J_{5\text{-}4}$ = 9.9 Hz, $J_{5\text{-}6}$ = 6.1 Hz, 1H, H-5), 3.99-3.95 (m, 1H, H-3’), 2.77-2.75 (m, 2H, CH_{2Lev}), 2.64-2.52 (m, 4H, CH_{2Lev}, H-2a’’, H-2b’’), 2.45 (dd, $J_{2a'\text{-}2b'}$ = 15.8 Hz, $J_{2a'\text{-}3'}$ = 3.2 Hz, 1H, H-2a’), 2.39 (dd, $J_{2b'\text{-}2a'}$ = 15.8 Hz, $J_{2b'\text{-}3'}$ = 8.9 Hz, 1H, H-2b’), 2.33 (s, 3H, CH_{3STol}), 2.18 (s, 3H, CH_{3Lev}), 1.60-1.21 (m, 27H, H-6, 12 × CH_2), 0.88-0.85 (m, 6H, 2 × CH_3); ^{13}C NMR (150 MHz, $CDCl_3$) δ (ppm) 206.3 (CO_{Lev}), 172.6, 172.1, 170.0 (3C, $COOR_{Lev}$, C-1’, C-1’’), 165.6 ($COOR_{AZMB}$), 138.4 (C_{Ar}), 137.7 (C_{Ar}), 133.4-128.0 (10C, 2 x C_{Ar}, 8 × CH_{Ar}), 86.1 (C-1), 72.4 (C-2), 71.5 (C-4), 70.6 (C-3’’), 69.8 (C-3), 68.4 (C-3’), 67.9 (C-5), 53.2 (CH_{2AZMB}), 41.9 (C-2’), 38.8 (C-2’’), 37.8 (CH_{2Lev}), 36.8-22.7 (14C, 12 × CH_2, CH_{2Lev}, CH_{3Lev}), 21.3 (CH_{3STol}), 17.5 (C-6),

14.23 (CH_3), 14.20 (CH_3); HRMS (ESI-TOF) *m/z* [M + NH_4]$^+$ calcd for $C_{46}H_{69}N_4O_{11}S$ 885.4678; found 885.4691; *m/z* [M + Na]$^+$ calcd for $C_{46}H_{65}NaN_3O_{11}S$ 890.4232; found 890.4252.

Macrolides 39*β* and 39*α*.

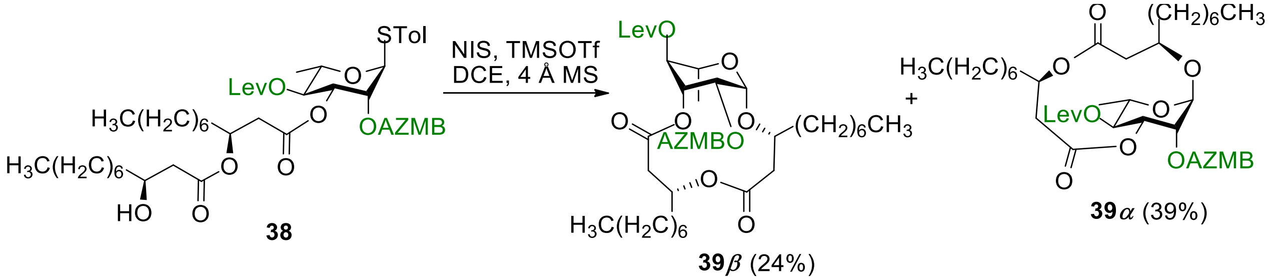

Alcohol **38** (209 mg, 0.241 mmol, 1.0 equiv) and NIS (87 mg, 0.39 mmol, 1.6 equiv) were dried under high vacuum for 1 h. Activated 4 Å MS (836 mg, 4 mg•mg^{-1} of alcohol **38**) and anhydrous DCE (24 mL) were added and the suspension was stirred under an Ar atmosphere for 1 h. The mixture was cooled to −10 °C and TMSOTf (17 μL, 96 μmol, 0.4 equiv) was added. The suspension was stirred at −10 to 0 °C for 45 min, quenched with Et_3N, and filtered over Celite. The solvents were evaporated under reduced pressure and the residue was purified by silica gel flash chromatography (Hex/EtOAc 9:1 to 7:3) to give macrolactone **39*β*** (*β*-anomer, 43 mg, 24%) and macrolactone **39*α*** (*α*-anomer, 71 mg, 39%) as yellow oils.

Data for compound **39*β***: R_f 0.60 (Hex/EtOAc 6:4); $[\alpha]^{20}_{D}$ –38 (*c* 0.7, $CHCl_3$); ^{1}H NMR (600 MHz, $CDCl_3$) δ (ppm) 8.13-8.11 (m, 1H, CH_{AZMB}), 7.59-7.56 (m, 1H, CH_{AZMB}), 7.50-7.47 (m, 2H, 2 × CH_{AZMB}), 5.44 (br t, J = 3.6 Hz, 1H, H-3), 5.31 (t, J = 3.7 Hz, 1H, H-2), 5.18-5.14 (m, 1H, H-3’’), 5.08 (d, J = 3.3 Hz, 1H, H-1), 5.05 (t, J = 3.2 Hz, 1H, H-4), 4.93 (d, J = 14.6 Hz, 1H, C*H*H_{AZMB}), 4.77 (d, J = 14.6 Hz, 1H, CHH_{AZMB}), 4.09-4.05 (m, 1H, H-5), 3.92-3.87 (m, 1H, H-3’), 2.87-2.61 (m, 5H, 2 × CH_{2Lev}, H-2a’’), 2.48 (dd, $J_{2a'-2b'}$ = 12.0 Hz, $J_{2a'-3'}$ = 3.3 Hz, 1H, H-2a’), 2.41-2.34 (m, 2H, H-2b’, H-2b’’), 2.21 (s, 3H, CH_{3Lev}), 1.90-1.86 (m, 1H, C*H*H), 1.62 (d, J = 7.3 Hz, 3H, H-6), 1.52-1.24 (m, 23H, 11 × CH_2, CH*H*), 0.88 (t, J = 6.9 Hz, 6H, 2 × CH_3); ^{13}C NMR (150 MHz, $CDCl_3$) δ (ppm) 206.4 (*C*O_{Lev}), 171.7, 171.5, 170.5 (3C, C-1’, C-1’’, *C*OOR$_{Lev}$), 166.0 (*C*OOR$_{AZMB}$), 138.0 (*C*$_{AZMB}$), 133.2-127.6 (5C, *C*$_{AZMB}$, 4 × *C*H$_{AZMB}$), 95.4 (C-1, $^1J_{C1-H1}$ = 168 Hz),

79.3 (C-3’), 73.1 (C-4), 71.4, 71.2 (2C, C-5, C-3’’), 67.9, 67.8 (2C, C-3, C-2), 53.3 (CH_{2AZMB}), 41.4 (C-2’), 40.6 (C-2’’), 38.1 (CH_{2Lev}), 36.0-22.8 (14C, CH_{2Lev}, CH_{3Lev}, 12 × CH_2), 20.9 (C-6), 14.2 (2C, 2 × CH_3); HRMS (ESI-TOF) *m/z* $[M + NH_4]^+$ calcd for $C_{39}H_{61}N_4O_{11}$ 761.4331; found 761.4339; *m/z* $[M + Na]^+$ calcd for $C_{39}H_{57}NaN_3O_{11}$ 766.3885; found 766.3891.

Data for compound **39*α***: R_f 0.51 (Hex/EtOAc 6:4); $[\alpha]^{20}_D$ –8 (*c* 0.3, $CHCl_3$); ^1H NMR (600 MHz, $CDCl_3$) δ (ppm) 8.12-8.10 (m, 1H, CH_{AZMB}), 7.61-7.58 (m, 1H, CH_{AZMB}), 7.54-7.53 (m, 1H, CH_{AZMB}), 7.47-7.44 (m, 1H, CH_{AZMB}), 5.64 (dd, J_{3-4} = 10.0 Hz, J_{3-2} = 3.6 Hz, 1H, H-3), 5.40 (dd, J_{2-3} = 3.7 Hz, J_{2-1} = 1.4 Hz, 1H, H-2), 5.22-5.17 (m, 2H, H-3’’, H-4), 5.03 (br s, 1H, H-1), 4.87 (d, *J* = 14.7 Hz, 1H, CHH_{AZMB}), 4.84 (d, *J* = 14.7 Hz, 1H, CHH_{AZMB}), 4.29 (dq, J_{5-4} = 12.4 Hz, J_{5-6} = 6.2 Hz, 1H, H-5), 4.26-4.22 (m, 1H, H-3’), 2.76-2.74 (m, 2H, CHH_{Lev}, CHH_{Lev}), 2.65-2.60 (m, 2H, H-2a’’, H-2a’), 2.58-2.56 (m, 2H, CHH_{Lev}, CHH_{Lev}), 2.45 (dd, $J_{2b''-2a''}$ = 6.6 Hz, $J_{2b''-3''}$ = 4.1 Hz, 1H, H-2b’’*), 2.43 (dd, $J_{2b'-2a'}$ = 6.5 Hz, $J_{2b'-3'}$ = 4.1 Hz, 1H, H-2b’*), 2.17 (s, 3H, CH_{3Lev}), 1.65-1.20 (m, 27H, 12 × CH_2, H-6), 0.87-0.85 (m, 6H, 2 × CH_3); ^{13}C NMR (150 MHz, $CDCl_3$) δ (ppm) 206.5 (CO_{Lev}), 172.4, 171.2, 168.8 (3C, C-1’, C-1’’, $COOR_{Lev}$, 165.8 ($COOR_{AZMB}$), 137.6-128.5 (6C, 2 × C_{AZMB}, 4 × CH_{AZMB}), 94.2 (C-1, $^1J_{C1-H1}$ = 170 Hz), 72.24, 72.15, 71.8 (3C, C-2, C-4, C-3’), 70.7 (C-3’’), 66.5 (C-5), 53.2 (CH_{2AZMB}), 38.8, 38.4, 37.9 (3C, C-2’, C-2’’, CH_{2Lev}), 34.2-22.8 (14C, CH_{2Lev}, CH_{3Lev}, 12 × CH_2), 17.3 (C-6), 14.2 (2C, 2 × CH_3); HRMS (ESI-TOF) *m/z* $[M + H]^+$ calcd for $C_{39}H_{58}N_3O_{11}$ 744.4066; found 744.4068; *m/z* $[M + NH_4]^+$ calcd for $C_{39}H_{61}N_4O_{11}$ 761.4331; found 761.4338.

Macrolide S18*β*.

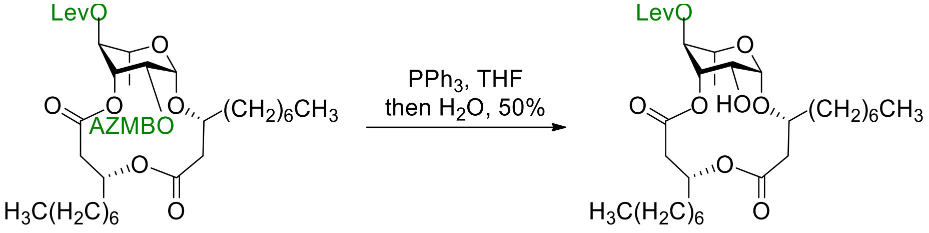

39*β* **S18*β***

PPh_3 (16 mg, 0.060 mmol, 1.2 equiv) was added to a solution of compound **39*β*** (38 mg, 50 μmol, 1.0 equiv) in anhydrous THF (1.5 mL). The mixture was stirred at 60 °C for 2 h under an Ar atmosphere, after which H_2O (0.2 mL) was added. The solution was stirred at 60 °C for 4 h and co-evaporated with toluene. The residue was purified by silica gel flash chromatography (Hex/EtOAc 9:1 to 75:25) to give the corresponding *O*-2 alcohol **S18*β*** (15 mg, 50%) as a colorless oil. R_f 0.35 (Hex/EtOAc 6:4); $[\alpha]^{20}_{D}$ +61 (*c* 0.3, $CHCl_3$); ^{1}H NMR (600 MHz, $CDCl_3$) δ (ppm) 5.54-5.48 (m, 1H, H-3’’), 5.18 (t, J = 3.9 Hz, 1H, H-3), 4.97 (t, J = 3.8 Hz, 1H, H-4), 4.89 (d, J = 2.5 Hz, 1H, H-1), 4.10-4.06 (m, 1H, H-3’), 3.99 (dt, $J_{2\text{-}OH}$ = 12.6 Hz, $J_{2\text{-}1,\,2\text{-}3}$ = 3.6 Hz, 1H, H-2), 3.93-3.87 (m, 1H, H-5), 3.40 (d, J = 12.6 Hz, 1H, O*H*), 2.82-2.46 (m, 8H, 2 × CH_{2Lev}, H-2a’’, H-2b’’, H-2a’, H-2b’), 2.18 (s, 3H, CH_{3Lev}), 1.80-1.52 (m, 4H, 2 × CH_2), 1.40 (d, J = 7.0 Hz, 3H, H-6), 1.30-1.26 (m, 20H, 10 × CH_2), 0.90-0.87 (m, 6H, 2 × CH_3); ^{13}C NMR (150 MHz, $CDCl_3$) δ (ppm) 206.3 (CO_{Lev}), 175.1, 171.6, 170.1 (3C, C-1’, C-1’’, $COOR_{Lev}$), 98.2 (C-1), 78.5 (C-3’), 73.4 (C-4), 71.2 (C-3), 70.8 (C-3’’), 70.1 (C-5), 66.9 (C-2), 41.3, 40.9 (2C, C-2’, C-2’’), 38.0 (CH_{2Lev}), 35.7-22.8 (14C, CH_{2Lev}, CH_{3Lev}, 12 × CH_2), 20.5 (C-6), 14.2 (2C, 2 × CH_3); HRMS (ESI-TOF) *m/z* $[M + NH_4]^+$ calcd for $C_{31}H_{56}NO_{10}$ 602.3899; found 602.3904; *m/z* $[M + Na]^+$ calcd for $C_{31}H_{52}NaO_{10}$ 607.3453; found 607.3454.

(1→3)-Macrolactonized Rhamnolipid 6*β*.

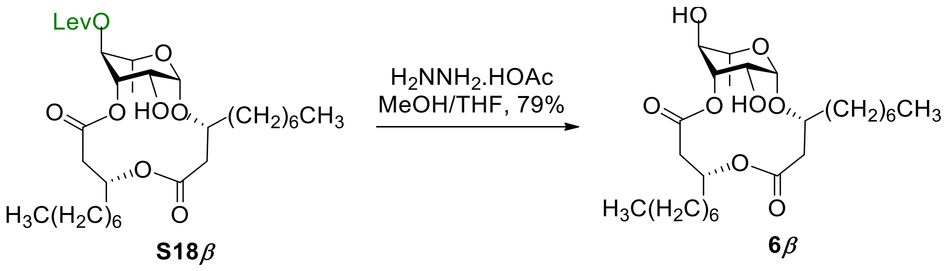

To a solution of alcohol **S18*β*** (13 mg, 22 μmol, 1.0 equiv) in anhydrous THF/MeOH (10:1 *v/v*, 1.5 mL) was added a solution of hydrazine monohydrate (15 μL, 0.30 mmol, 14 equiv) and HOAc (37 μL) in anhydrous THF/MeOH (5:1 *v/v*, 0.3 mL). The solution was stirred at rt under an Ar atmosphere for 30 min until a white precipitate was formed. The suspension was co-evaporated with toluene and the residue was purified by silica gel flash chromatography (Hex/EtOAc 8:2) to give macrolactone **6*β*** (β-anomer, 8.3 mg, 79%) as a colorless oil: R_f 0.47 (Hex/EtOAc 6 :4); $[\alpha]^{20}_D$ –47 (*c* 0.6, $CHCl_3$); ^{1}H NMR (600 MHz, $CDCl_3$) δ (ppm) 5.59-5.55 (m, 1H, H-3’’), 4.80 (d, J = 0.9 Hz, 1H, H-1), 4.68 (dd, $J_{3\text{-}2}$ = 5.6 Hz, $J_{3\text{-}4}$ = 3.9 Hz, 1H, H-3), 4.10-4.04 (m, 3H, H-2, H-3’, O*H*), 3.71-3.65 (m, 2H, H-4, H-5), 3.36 (d, J = 12.8 Hz, 1H, O*H*), 2.80 (dd, $J_{2a''\text{-}2b''}$ = 18.3 Hz, $J_{2a''\text{-}3''}$ = 11.3 Hz, 1H, H-2a’’), 2.66 (dd, $J_{2b''\text{-}2a''}$ = 18.3 Hz, $J_{2b''\text{-}3''}$ = 1.4 Hz, 1H, H-2b’’), 2.55 (dd, $J_{2a'\text{-}2b'}$ = 13.4 Hz, $J_{2a'\text{-}3'}$ = 3.8 Hz, 1H, H-2a’), 2.44 (dd, $J_{2b'\text{-}2a'}$ = 13.4 Hz, $J_{2b'\text{-}3'}$ = 11.4 Hz, 1H, H-2b’), 1.73-1.26 (m, 27H, 12 × CH_2, H-6), 0.89-0.83 (m, 6H, 2 × CH_3); 175.0, 171.8 (2C, C-1’, C-1’’), 99.6 (C-1), 79.5, 79.2 (2C, C-3, C-3’), 74.9 (C-4), 69.3, 69.1 (2C, C-3’’, C-5), 66.9 (C-2), 40.9 (C-2’), 39.6 (C-2’’), 35.0-22.8 (12C, 12 × CH_2), 19.9 (C-6), 14.2 (2C, 2 × CH_3); HRMS (ESI-TOF) *m/z* $[M + NH_4]^+$ calcd for $C_{26}H_{50}NO_8$ 504.3531; found 504.3537; *m/z* $[M + Na]^+$ calcd for $C_{26}H_{46}NaO_8$ 509.3085; found 509.3094. Analytical HPLC analysis was performed using method B (45.4 min.).

Macrolide S18*α*.

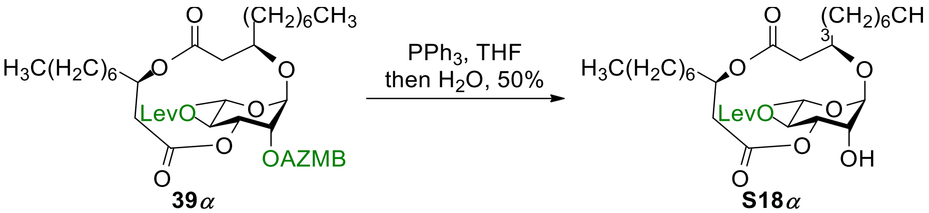

PPh_3 (30 mg, 0.12 mmol, 1.2 equiv) was added to a solution of macrolactone **39*α*** (71 mg, 95 μmol, 1.0 equiv) in anhydrous THF (2.9 mL). The mixture was stirred at 60 °C for 2 h under an Ar atmosphere, after which H_2O (0.4 mL) was added. The solution was stirred at 60 °C for 4 h and co-evaporated with toluene. The residue was purified by silica gel flash chromatography (Tol/EtOAc 95:5 to 85:15) to give *O*-2 alcohol **S18*α*** (28 mg, 50%) as a colorless oil: R_f 0.32 (Tol/EtOAc 8:2); $[\alpha]^{20}_{D}$ –60 (*c* 0.9, $CHCl_3$); ^{1}H NMR (600 MHz, $CDCl_3$) δ (ppm) 5.45 (dd, $J_{3\text{-}4}$ = 9.9 Hz, $J_{3\text{-}2}$ = 3.6 Hz, 1H, H-3), 5.31-5.28 (m, 1H, H-3''), 5.08 (t, *J* = 10.0 Hz, 1H, H-4), 4.90 (s, 1H, H-1), 4.25-4.23 (m, 1H, H-3'), 4.10-4.05 (m, 2H, H-2, H-5), 2.75-2.70 (m, 3H, $CH_{2\mathrm{Lev}}$, H-2a''), 2.60-2.54 (m, 3H, $CH_{2\mathrm{Lev}}$, H-2b''), 2.47-2.43 (m, 2H, H-2a', H-2b'), 2.29 (d, *J* = 2.6 Hz, 1H, O*H*), 2.17 (s, 3H, $CH_{3\mathrm{Lev}}$), 1.65-1.21 (m, 27H, 12 × CH_2, H-6), 0.89-0.86 (m, 6H, 2 × CH_3); ^{13}C NMR (150 MHz, $CDCl_3$) δ (ppm) 206.5 (CO_{Lev}), 172.3, 170.8, 169.0 (3C, C-1', C-1'', $COOR_{\mathrm{Lev}}$), 95.5 (C-1), 71.83, 71.75, 71.5 (3C, C-3, C-4, C-3''), 70.3 (C-3'), 69.7 (C-5), 66.3 (C-2), 39.4, 38.8 (C-2', C-2''), 37.9 ($CH_{2\mathrm{Lev}}$), 34.4-22.8 (14C, $CH_{2\mathrm{Lev}}$, $CH_{3\mathrm{Lev}}$, 12 × CH_2), 18.3 (C-6), 14.24 (CH_3), 14.23 (CH_3); HRMS (ESI-TOF) *m/z* $[M + NH_4]^+$ calcd for $C_{31}H_{56}NO_{10}$ 602.3899; found 602.3907; *m/z* $[M + Na]^+$ calcd for $C_{31}H_{52}NaO_{10}$ 607.3453; found 607.3459.

(1→3)-Macrolactonized Rhamnolipid 6*α*.

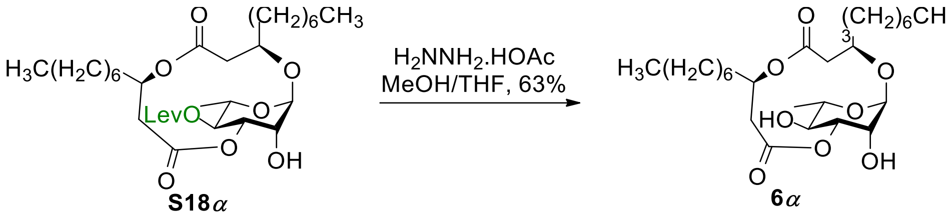

To a solution of alcohol **S18*α*** (25 mg, 42 *μ*mol, 1.0 equiv) in anhydrous THF/MeOH (10:1 *v/v*, 3 mL) was added a solution of hydrazine monohydrate (29 *μ*L, 0.59 mmol, 14 equiv) and HOAc (72 *μ*L) in anhydrous THF/MeOH (5:1 *v/v*, 0.6 mL). The solution was stirred at rt under an Ar atmosphere for 30 min until a white precipitate was formed. The suspension was co-evaporated with toluene and the residue was purified by preparative TLC (DCM/MeOH 94:6) to give macrolactone **6*α*** (*α*-anomer, 13 mg, 63%) as a colorless oil: R_f 0.45 (DCM/MeOH 95 :5); $[\alpha]^{20}_{D}$ –16 (*c* 0.8, $CHCl_3$); ^1H NMR (600 MHz, $CDCl_3$) δ (ppm) 5.26 (dd, $J_{3\text{-}4}$ = 9.6 Hz, $J_{3\text{-}2}$ = 3.4 Hz, 1H, H-3), 5.17-5.12 (m, 1H, H-3’’), 4.86 (s, 1H, H-1), 4.28-4.26 (m, 1H, H-3’), 3.92-3.87 (m, 2H, H-2, H-5), 3.44 (t, J = 9.7 Hz, 1H, H-4), 2.74 (dd, $J_{2a''\text{-}2b''}$ = 16.0 Hz, $J_{2a''\text{-}3''}$ = 2.6 Hz, 1H, H-2a’’), 2.66 (dd, $J_{2b''\text{-}2a''}$ = 16.0 Hz, $J_{2b''\text{-}3''}$ = 10.7 Hz, 1H, H-2b’’), 2.46 (dd, $J_{2a'\text{-}2b'}$ = 15.8 Hz, $J_{2a'\text{-}3'}$ = 2.3 Hz, 1H, H-2a’), 2.40 (dd, $J_{2b'\text{-}2a'}$ = 15.9 Hz, $J_{2b'\text{-}3'}$ = 10.1 Hz, 1H, H-2b’), 1.72-1.46 (m, 4H, 2 × CH_2), 1.33 (d, J = 6.2 Hz, 3H, H-6), 1.28-1.26 (m, 20H, 10 × CH_2), 0.88 (t, J = 6.8 Hz, 6H, 2 × CH_3); ^{13}C NMR (150 MHz, $CDCl_3$) δ (ppm) 171.4, 170.5 (2C, C-1’, C-1’’), 96.7 (C-1), 74.7 (C-3), 73.0 (C-3’), 72.6 (C-4), 71.3 (C-3’’), 70.7, 68.4 (2C, C-2, C-5), 39.3 (C-2’), 39.1 (C-2’’), 34.2-22.8 (12C, 12 × CH_2), 17.2 (C-6), 14.2 (2C, 2 × CH_3); HRMS (ESI-TOF) *m/z* $[M + H]^+$ calcd for $C_{26}H_{47}O_8$ 487.3265; found 487.3976. Analytical HPLC analysis was performed using method B (32.8 min.).

4. Experimental Procedures for Biological Evaluation and Surfactant Properties.

Antimicrobial Activity and Synergy Testing. Minimum inhibitory concentration (MIC) determinations and synergy testing were performed for *Pseudomonas aeruginosa* PA14 (ED14), *Pseudomonas aeruginosa* LESB58 (ED639), *Staphylococcus aureus* MRSA (ED711), *Staphylococcus aureus* Newman (ED94), *Escherichia coli* DH5α (ED78), *Bacillus subtilis* PY79 (ED66), *Candida albicans* ATCC 10231 (ED3866), and *Candida albicans* LSPQ 0199 (ED3867) by the checkerboard method in 96 well plates with Mueller-Hinton broth. Kanamycin and tetracyclin (antibiotics used as controls) were tested at concentrations ranging from 3.125 to 37.5 $\mu g \cdot mL^{-1}$, and ananatoside A (**1**), ananatoside B (**2**), and $RhaC_{10}C_{10}$ (**3**) were tested at concentrations ranging from 1.5625 to 37.5 $\mu g \cdot mL^{-1}$. The biosurfactants were serially diluted along the ordinate, while the antibiotics were diluted along the abscissa. Overnight cultures of each microorganism were diluted by 1000-fold in Mueller-Hinton broth, and added to each well with the corresponding combination of compounds. The plates were incubated aerobically overnight at 30 °C and 200 rpm. Control wells were included with each run. Fractional inhibitory concentrations (ΣFICs) were calculated as follows: ΣFIC = FIC A + FIC B, where FIC A is the MIC of compound A in the combination/MIC of compound A alone, and FIC B is the MIC of compound B in the combination/MIC of compound B alone. The combination is considered synergistic when the ΣFIC is ≤0.5, indifferent when the ΣFIC is ›0.5 to ‹2, and antagonistic when the ΣFIC is ≥2.[8]

Cell Culture. Human lung carcinoma (A549), human colorectal adenocarcinoma (DLD-1), and human normal skin fibroblasts (WS1) cell lines were obtained from the American Type Culture Collection (ATCC). All cell lines were cultured in minimum essential medium containing Earle's

salts and L-glutamine (Mediatech Cellgro, VA), to which were added 10% foetal bovine serum (Hyclone), vitamins (1×), penicillin (100 IU•mL^{-1}), streptomycin (100 μg•mL^{-1}), essential amino acids (1×), and sodium pyruvate (1×) (Mediatech Cellgro, VA). Cells were kept at 37 °C in a humidified environment containing 5% CO_2.

Cytotoxicity Assay. Exponentially growing A549, DLD-1 or WS1 cells were plated in 96-well microplates (Costar, Corning Inc.) at a density of 5×10^3 cells per well in 100 μL of culture medium and were allowed to adhere for 16 h prior treatment. Increasing concentrations of each compound in biotech DMSO (Sigma-Aldrich) were then added (100 μL per well) and the cells were incubated for 48 h. The final concentration of DMSO in the culture medium was maintained at 0.5% (*v/v*) to avoid solvent toxicity. Cytotoxicity was assessed using resazurin[9] on an automated 96-well Fluoroskan Ascent F1™ plate reader (Labsystems) using excitation and emission wavelengths of 530 and 590 nm, respectively. Fluorescence was proportional to the cellular metabolic activity in each well. Survival percentage was defined as the fluorescence in experimental wells as compared to that in control wells after subtraction of blank values. Each experiment was carried out three times in triplicate. IC_{50} results were expressed as means ± standard deviation.

Hemolytic Activity. The hemolytic activity of the synthetic surfactants was evaluated as previously described with small modifications.[10] Defibrinated sheep blood (Oxoid) was centrifuged at 1000 × g for 5 min. The pellet containing erythrocytes was washed once and suspended in 1X PBS to obtain a 1% erythrocytes suspension. All samples were suspended in DMSO/PBS (5:1 *v/v*) and serially diluted in a 96-well plate to obtain a range of concentrations from 1 mM to 7.8 μM. The erythrocytes suspension (160 μL) was added to 40 μL of samples to obtain final concentrations

ranging between 200 μM and 1.56 μM. Triton X-100 was used as a positive hemolysis control. The plate was then incubated at 37 °C with agitation at 150 rpm for 1 h and centrifuged at 1500 × g for 5 min. The supernatant was transferred in an empty plate and absorbance was measured at 540 nm using a Cytation3 microplate reader (Biotek). HC_{50} was calculated in comparison with the positive control. The experiment was performed in triplicate and repeated three times.

Plant Material and Growth Conditions. Tomato plants (*Solanum lycopersicum* L var. Ailsa craig, Scotland) were grown on soil in growth chamber with white fluorescent light [200 μmol•(m^{-2} s^{-1})], under 16 h/8 h light/dark regime, 60% relative humidity, and a temperature of 24/20 °C during four weeks prior treatment. *Arabidopsis thaliana* plants (ecotype Col-0) were grown on soil in growth chambers with white fluorescent light (150 μmol m^{-2} s^{-1}), under 12 h/12 h light/dark regime, 60% relative humidity, and a temperature of 20/20 °C during six weeks prior treatment.

Reactive Oxygen Species (ROS) Production. ROS assays were performed on four-week old tomato plants or six-week old *Arabidopsis* plants. Briefly, tomato leaf disks of 6 mm diameter or *Arabidopsis* petiole sections of 5 mm long were cut and placed in a 96-well plate (Optiplate TM-96 white, PerkinElmer) containing 150 μL of distilled H_2O and then incubated at rt for 24 h to reduce the wounding response.[11] The elicitation solution containing 0.2 μg•mL^{-1} luminol (A4685-5 g, Sigma), 20 μM horseradish peroxidase (P6782, Sigma) and the tested glycolipids (**1**, **2**, **3**, **4**, **5*α***, **5*β***, **6*α*** or **6*β***) at 100 μM was prepared. Methanol (0.5%) was used as negative control. Prior elicitation, the incubating distilled H_2O was carefully removed from each well, avoiding any tissue damage or desiccation. Then, the elicitation solution (150 μL) was quickly added to each well containing a leaf disk or a petiole. Luminescence (relative light units, RLU) was measured every 4

min during 720 min with a luminometer (Tecan SPARK 10M). Data are mean ± SEM (n = 6) and experiments were realized three times.

Surface Tension of Biosurfactants. The surface tensions of ananatoside A (**1**), ananatoside B (**2**), and Rha$C_{10}C_{10}$ (**3**) were measured in 20 mL aliquots by the du Noüy ring method using a Fisher tensiometer model 20 (Fisher Scientific, Pittsburgh, PA). The instrument was calibrated against water and measurements were performed in triplicate at rt. The critical micelle concentration (CMC) was determined from a plot of surface tension against each concentration, where the CMC value corresponds to the intersection between the regression straight line of the linearly dependent region and the straight line passing through the plateau.[12]

Emulsification Analysis. The emulsification activity of culture extracts was tested against kerosene, *n*-hexadecane, and cyclohexane. Aliquots (5 mg) of ananatoside A (**1**), ananatoside B (**2**), and Rha$C_{10}C_{10}$ (**3**) were mixed with 5 mL of each solvent and vortexed at high speed for 2 min. After 24 h, the height of the stable emulsion layer was measured. The emulsification activity (E_{24}) is calculated as the ratio of the height of the emulsion layer and the total height of liquid after 24 h.[13]

Particles Size Analysis. Size measurements were performed in triplicate for ananatoside A (**1**), ananatoside B (**2**), and Rha$C_{10}C_{10}$ (**3**) in pure water using dynamic light scattering (DLS) on a Malvern Zetasizer Nano-ZS (Malvern Instruments, Malvern, UK). Samples were irradiated with red light (HeNe laser, wavelength λ = 632.8 nm) and the intensity fluctuations of the scattered light (using an angle of 173°) analysed to obtain an autocorrelation function. The Z-Ave value was reported as the mean diameter of nanoparticles where the cumulant method was adopted for data

analysis, data was acquired in automatic mode, the software incorporated a data quality report that indicated good quality for all data obtained.[14]

5. Molecular Modeling

Model compounds of macrolactonized rhamnolipids **4** to **6** were generated with ethyl groups instead of heptyl side chains (**40** to **42**) because we supposed that they would only increase the degree of liberty without improving the precision of the prediction for the chemical shifts. Both anomers were built in Avogadro[15] and files were exported in MDL mol file.[16] The RDKit ETKDGv2 algorithm[17, 18] was invoked in python to generate 1 000 conformers which were pruned with a RMSD threshold of 0.25 Å. The conformers were minimized using the MMFF94s force field and filtered with an energy windows of 80 kJ•mol^{-1} followed by a RMSD threshold of 0.25 Å. Following this step, the conformers of each isomer were aligned and inspected visually to ensure that the conformational space was thoroughly covered (Fig. S5). The conformers were further optimized at the mPW1PW91/6-31G(d,p) level of theory[19] using Gaussian 16 (rev. C.01)[20] after which a last filtration was accomplished using a 10 kJ•mol^{-1} window. All the DFT calculations were performed with the polarizable continuum model using the integral equation formalism variant (IEFPCM) to consider the solvent effect (chloroform) and an ultrafine grid for the integrals. The number of conformers retained after each step is presented in Table S2.

The retained geometries were optimized at two additional levels of theory, mPW1PW91/6-311+G(d,p) and B97-2/cc-pVTZ,[21, 22] after which the vibrational frequencies and thermochemical parameters were computed at each corresponding level of theory. NMR shielding tensors were computed using the gauge-independent atomic orbital (GIAO) method[23] and averaged using a Boltzmann-weighting function based on the thermal free energies at 25 °C of each conformer.[24] Shielding tensors for acetone and TMS were calculated at the same levels of theory and then used to compute NMR chemical shifts based on a multi-standard approach.[25, 26] A regression analysis

between experimental and referenced values was also performed to reduce systematic errors,[24] but it led to the same conclusion.

The experimental and calculated chemical shifts were compared following two schemes, a classical one in which each experimental NMR dataset was separately compared against both *in silico* anomers, and the combination scheme as developed by Lauro,[27] in which one pair of isolated anomers (ex. **5*α*/5*β***) was compared against their *in silico* analogs (*e.g.*, **41*α*/41*β*** or **41*β*/41*α***). The comparison criterium was the maximum absolute error (MAE) in both schemes, and the ^{1}H and ^{13}C chemical shifts were treated separately.

The conformers were categorized in conventional cyclohexane conformations assessed based on the six torsional angles of the sugar ring.[28] The relative abundances of these conformations were evaluated for each model compounds at each level of theory. 3D representations of the most abundant conformer in each category were rendered with PyMol, while the pie charts were crafted using the ggplot2 module in R.

Raw DFT calculation results for model compounds **40-42** are available on the preprint server ChemRxiv following this link: https://chemrxiv.org/ndownloader/files/26536706.

6. NMR Spectra for New Compounds

Figure S9 | ^{1}H NMR spectrum ($CDCl_3$, 600 MHz) of (*R*)-benzyl 3-hydroxydecanoate (**14**).

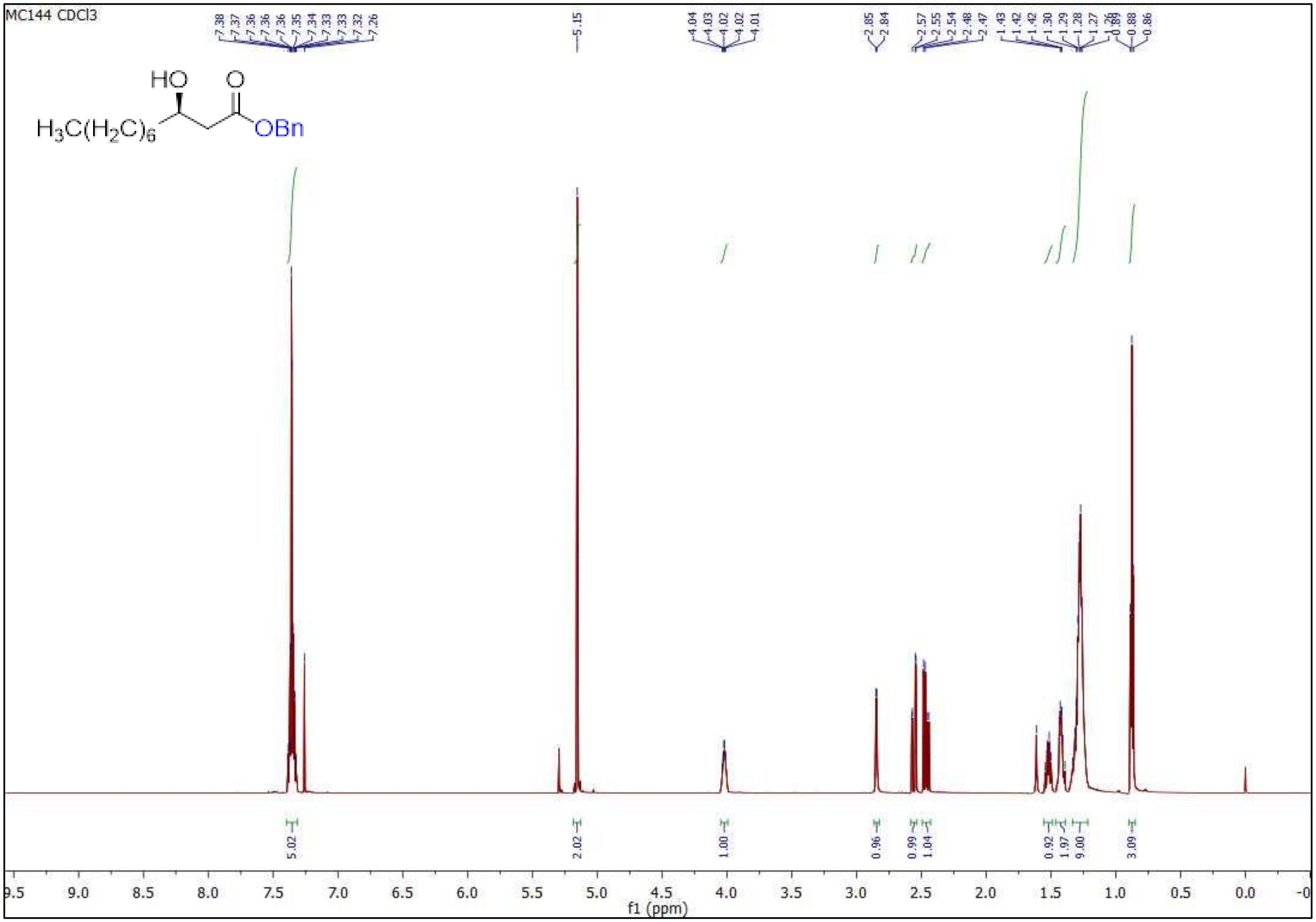

Figure S10 | COSY NMR spectrum ($CDCl_3$, 600 MHz) of (*R*)-benzyl 3-hydroxydecanoate (**14**).

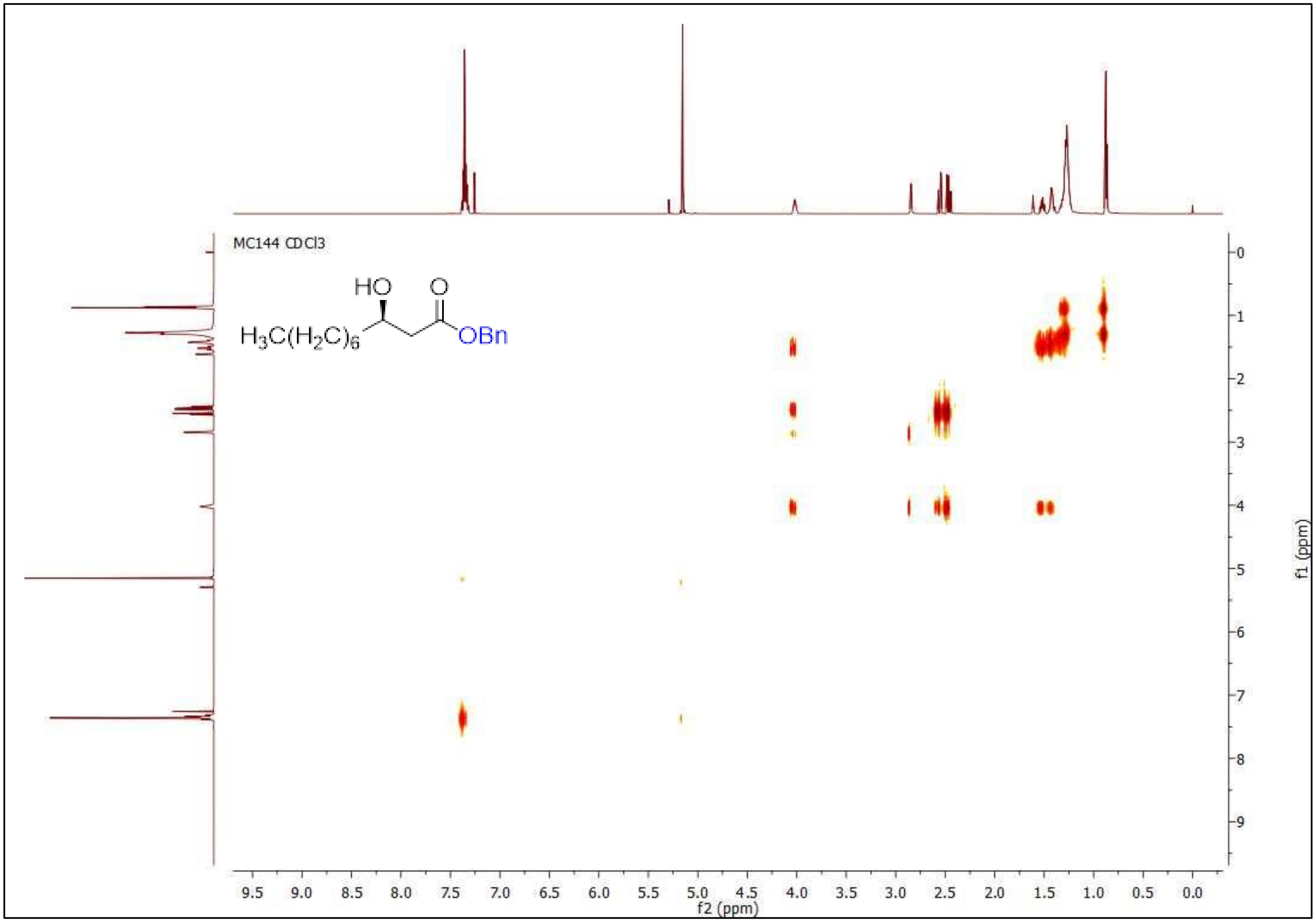

Figure S11 | ^{13}C NMR spectrum ($CDCl_3$, 150 MHz) of (*R*)-benzyl 3-hydroxydecanoate (**14**).

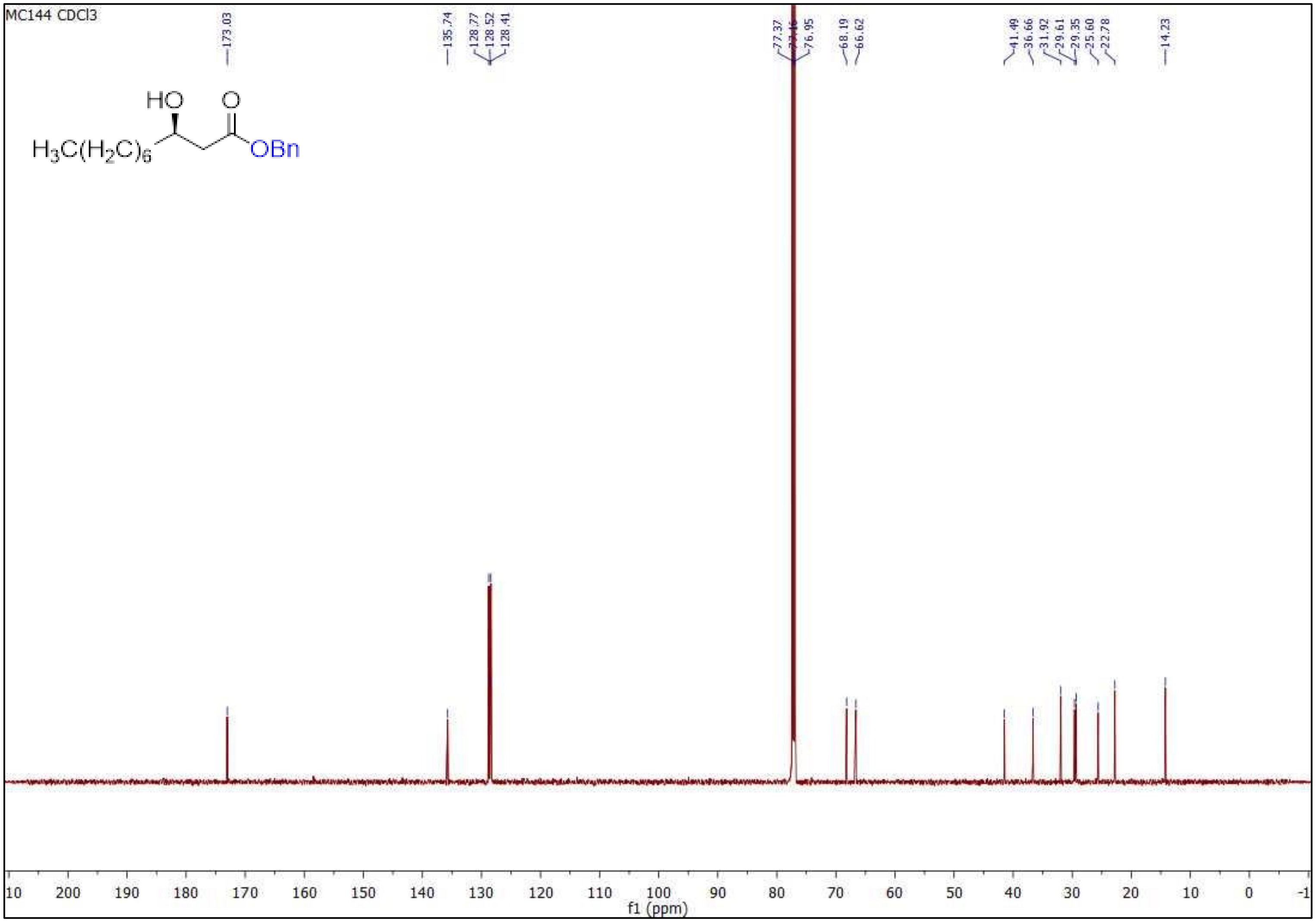

Figure S12 | HSQC NMR spectrum ($CDCl_3$, 600 MHz) of (*R*)-benzyl 3-hydroxydecanoate (**14**).

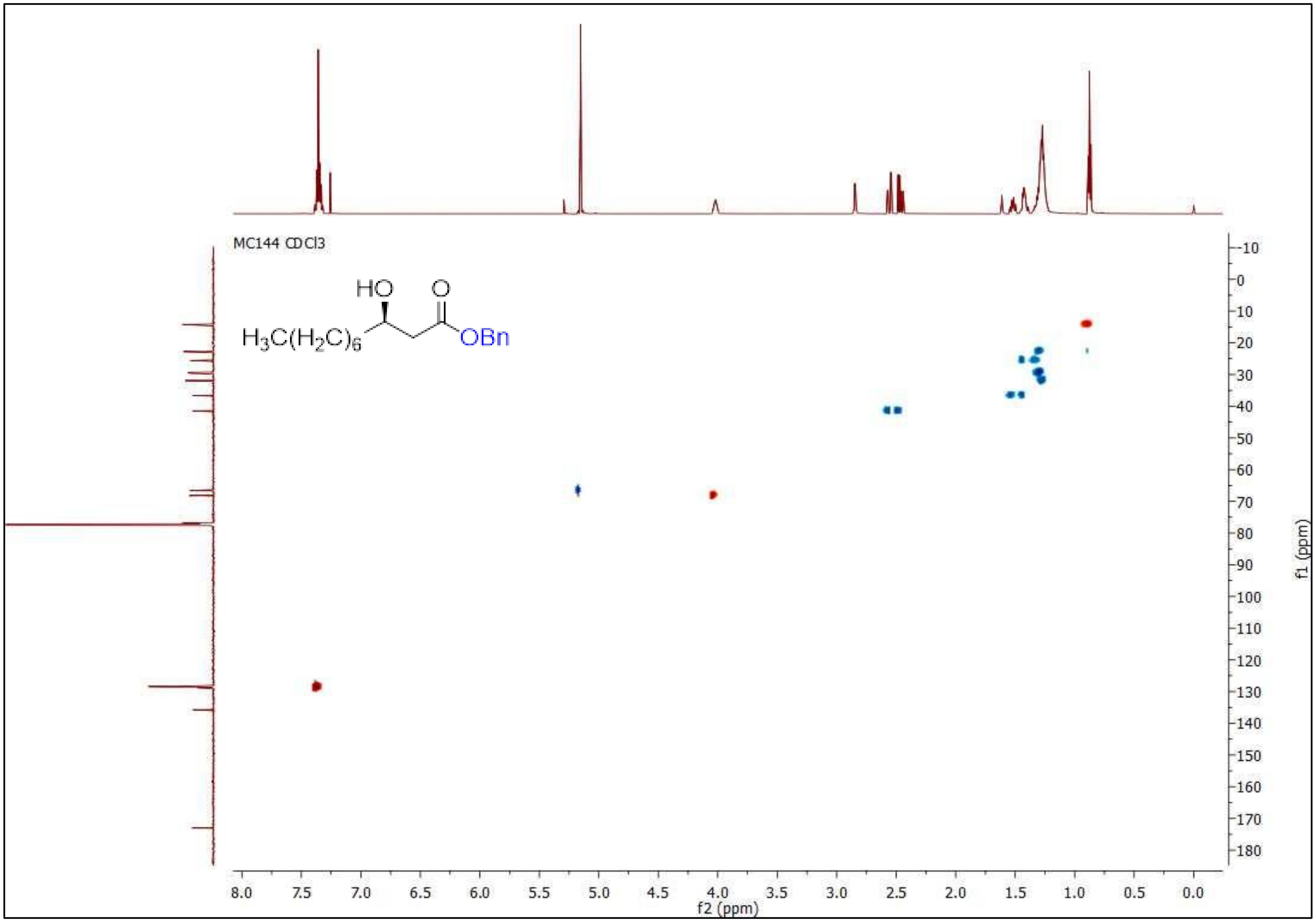

Figure S13 | ^{1}H NMR spectrum ($CDCl_3$, 600 MHz) of (*R*)-benzyl 3-(((*R*)-((*tert*-butyldimethylsilyl)oxy)decanoyl)oxy)decanoate (**16**).

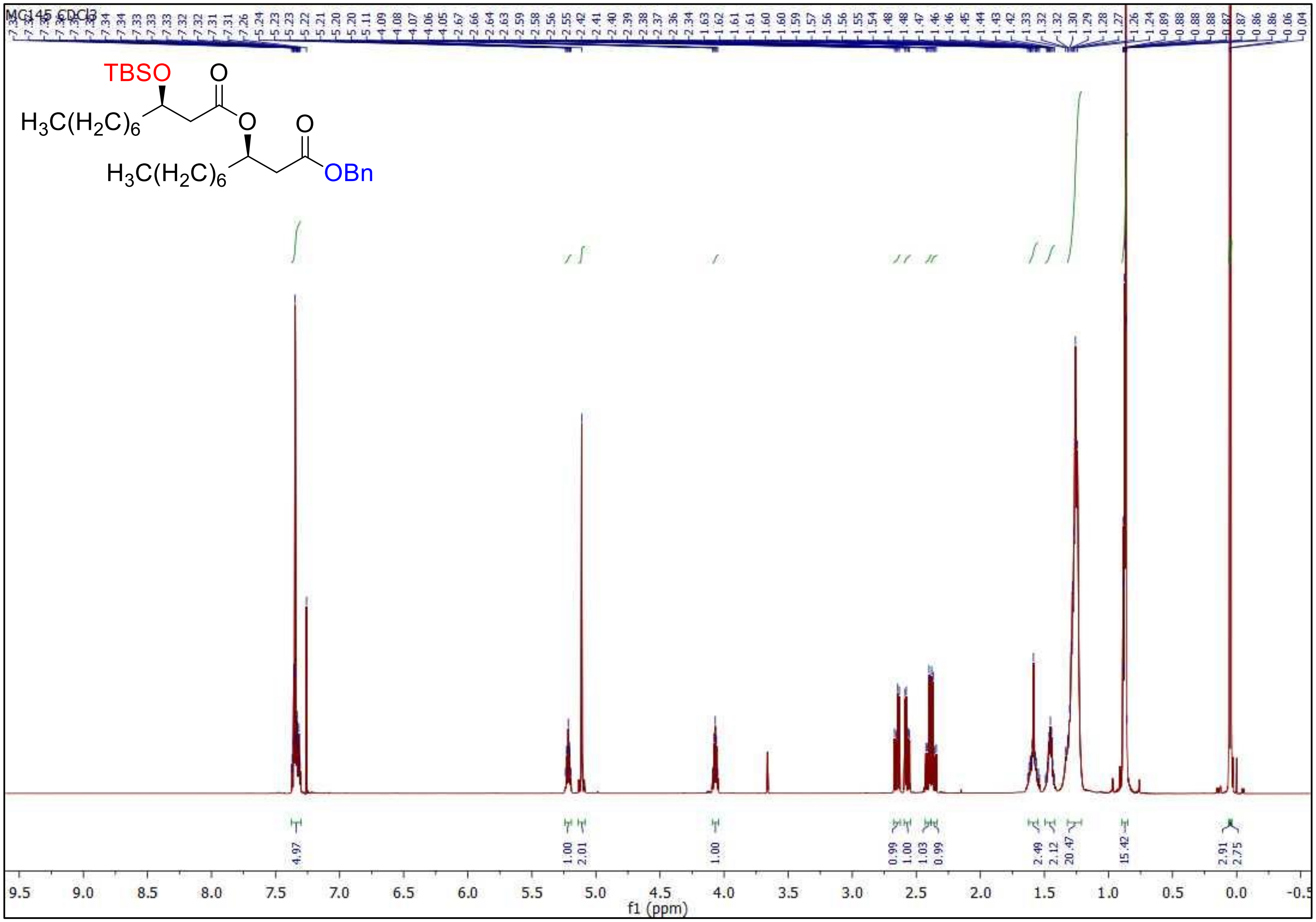

Figure S14 | COSY NMR spectrum ($CDCl_3$, 600 MHz) of (*R*)-benzyl 3-(((*R*)-((*tert*-butyldimethylsilyl)oxy)decanoyl)oxy)decanoate (**16**).

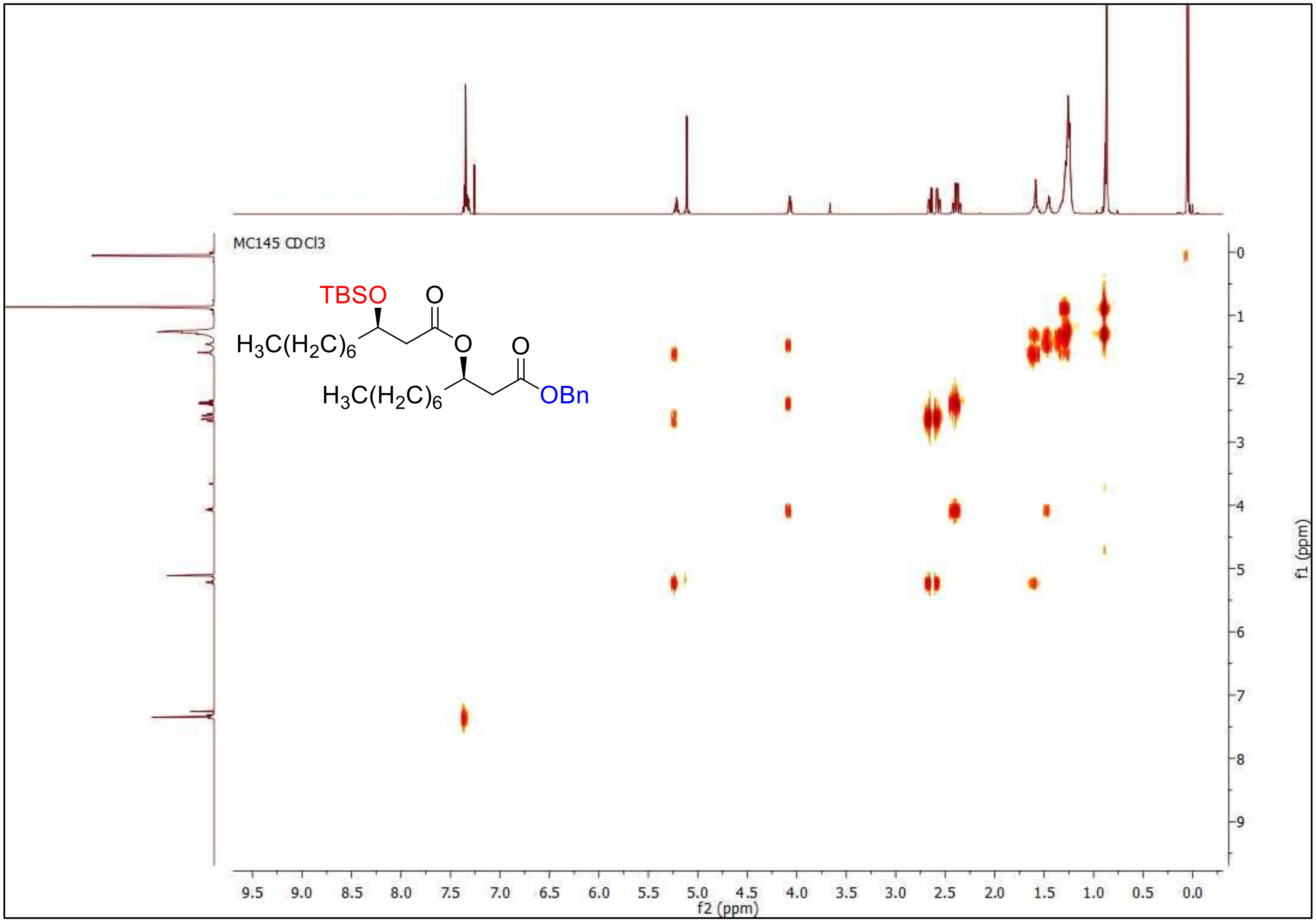

Figure S15 | ^{13}C NMR spectrum ($CDCl_3$, 150 MHz) of (*R*)-benzyl 3-(((*R*)-((*tert*-butyldimethylsilyl)oxy)decanoyl)oxy)decanoate (**16**).

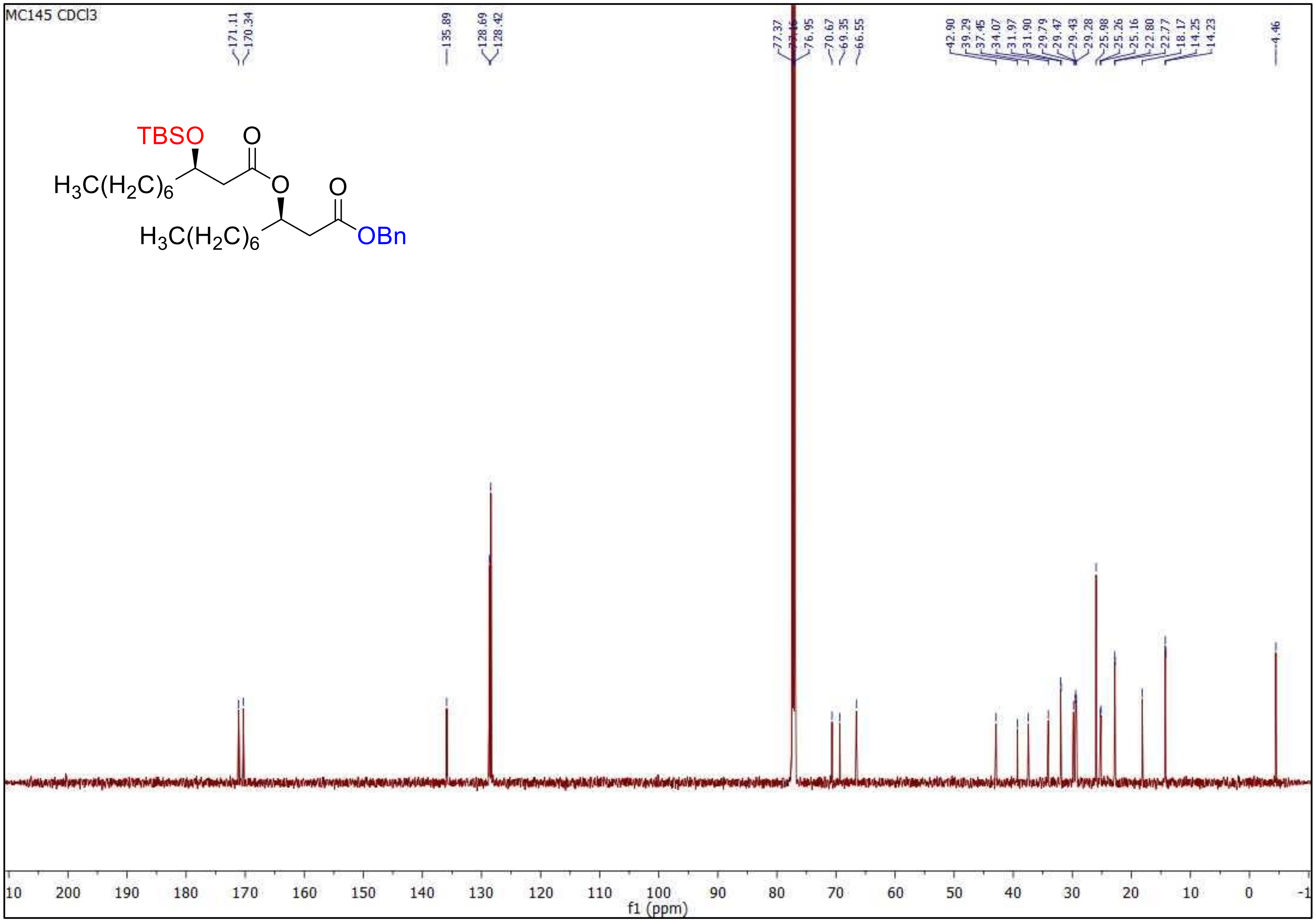

Figure S16 | HSQC NMR spectrum ($CDCl_3$, 600 MHz) of (*R*)-benzyl 3-(((*R*)-((*tert*-butyldimethylsilyl)oxy)decanoyl)oxy)decanoate (**16**).

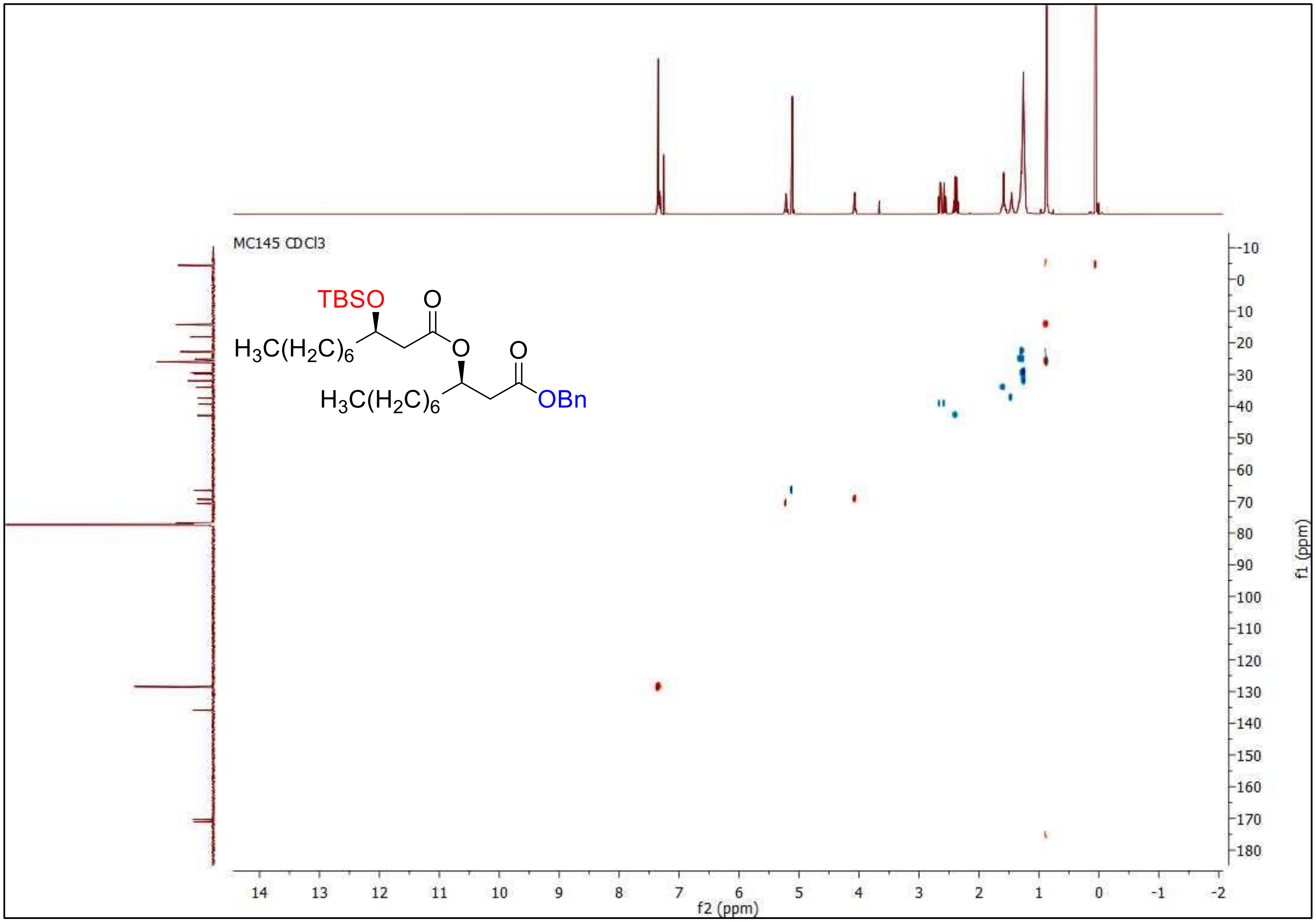

Figure S17 | ^{1}H NMR spectrum ($CDCl_3$, 600 MHz) of (*R*)-3-(((*R*)-3-((*tert*-butyldimethylsilyl)oxy)decanoyl)oxy)decanoic acid (**10**).

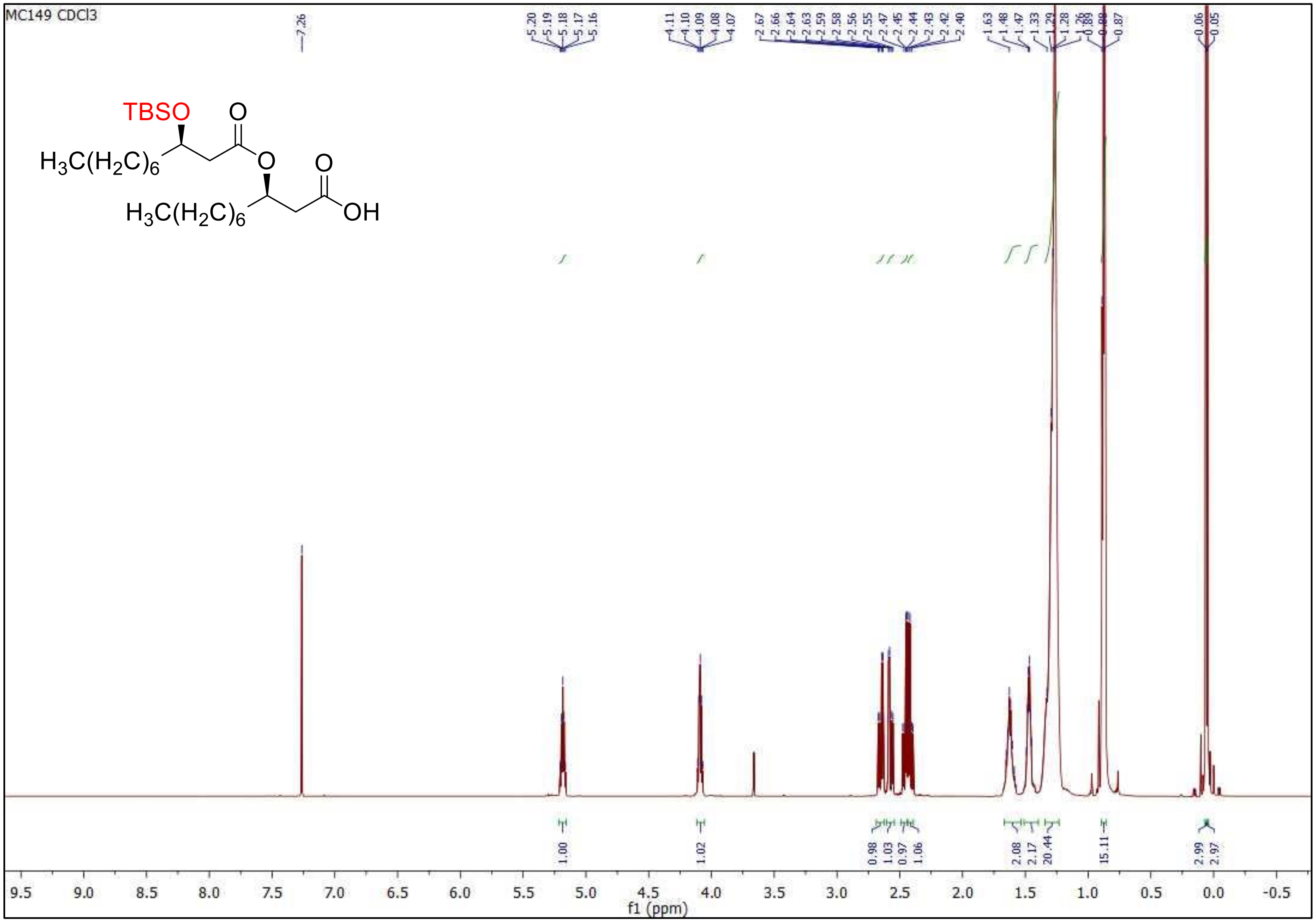

Figure S18 | COSY NMR spectrum ($CDCl_3$, 600 MHz) of (*R*)-3-(((*R*)-3-((*tert*-butyldimethylsilyl)oxy)decanoyl)oxy)decanoic acid (**10**).

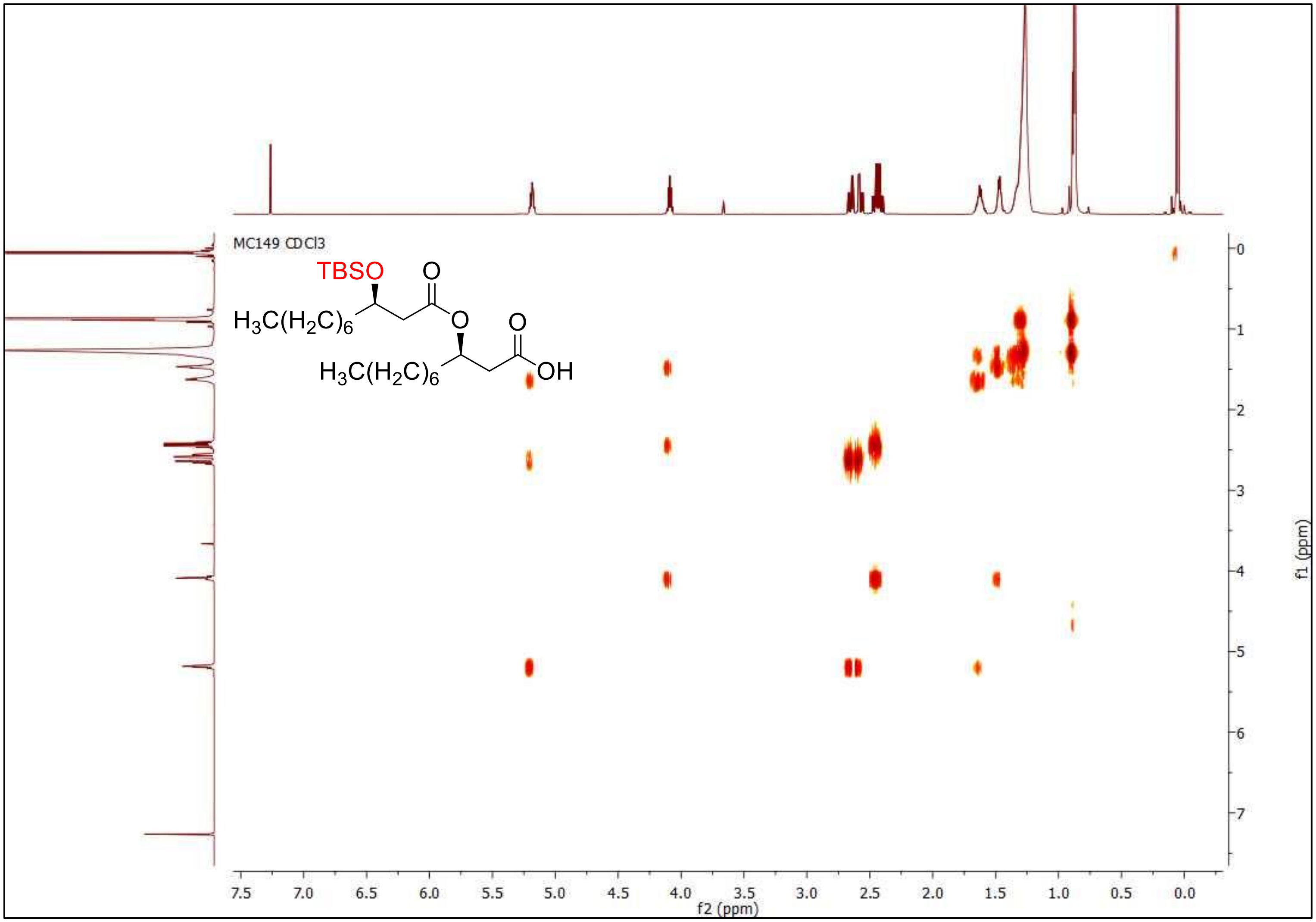

Figure S19 | ^{13}C NMR spectrum ($CDCl_3$, 600 MHz) of (*R*)-3-(((*R*)-3-((*tert*-butyldimethylsilyl)oxy)decanoyl)oxy)decanoic acid (**10**).

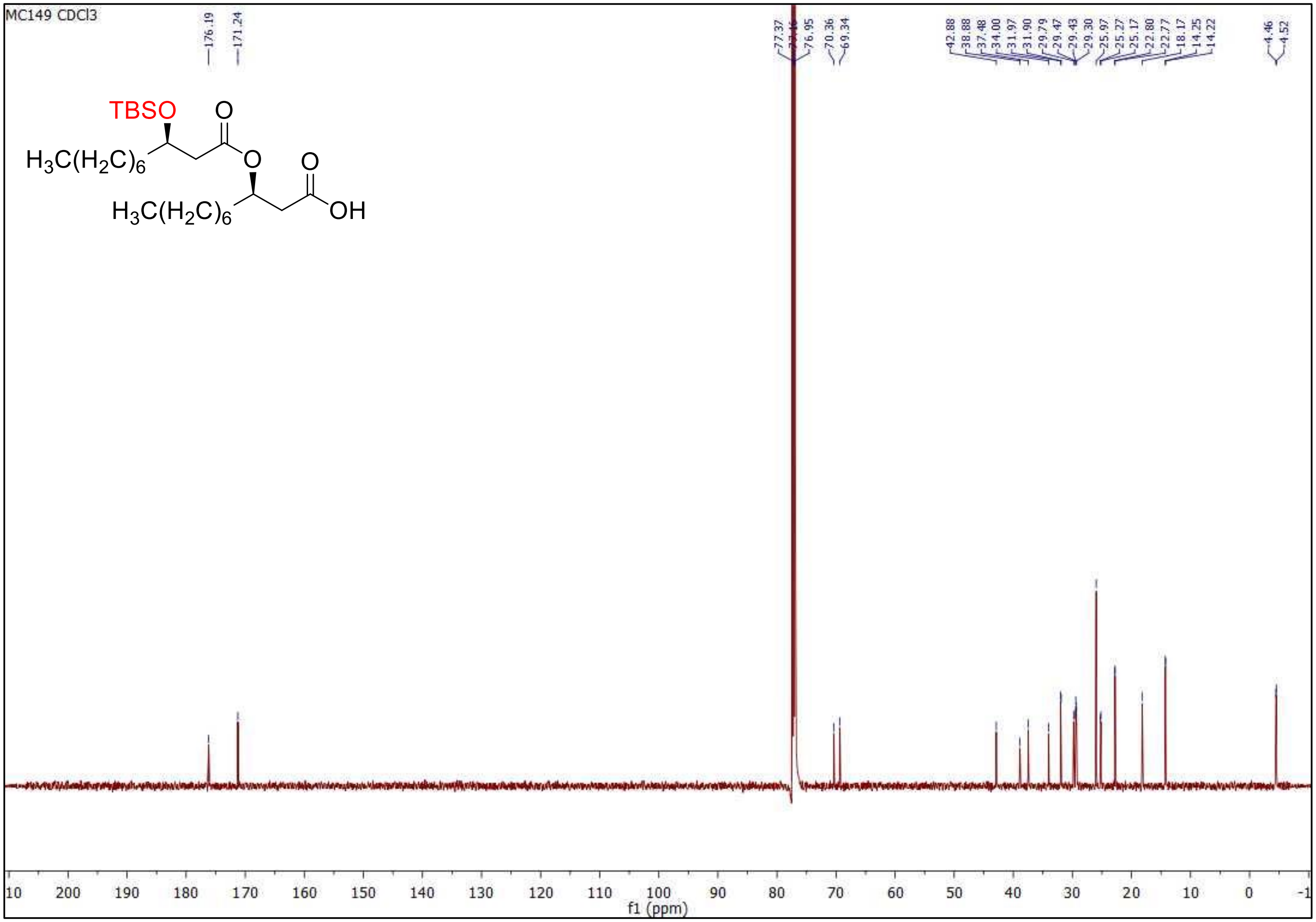

Figure S20 | HSQC NMR spectrum ($CDCl_3$, 600 MHz) of (*R*)-3-(((*R*)-3-((*tert*-butyldimethylsilyl)oxy)decanoyl)oxy)decanoic acid (**10**).

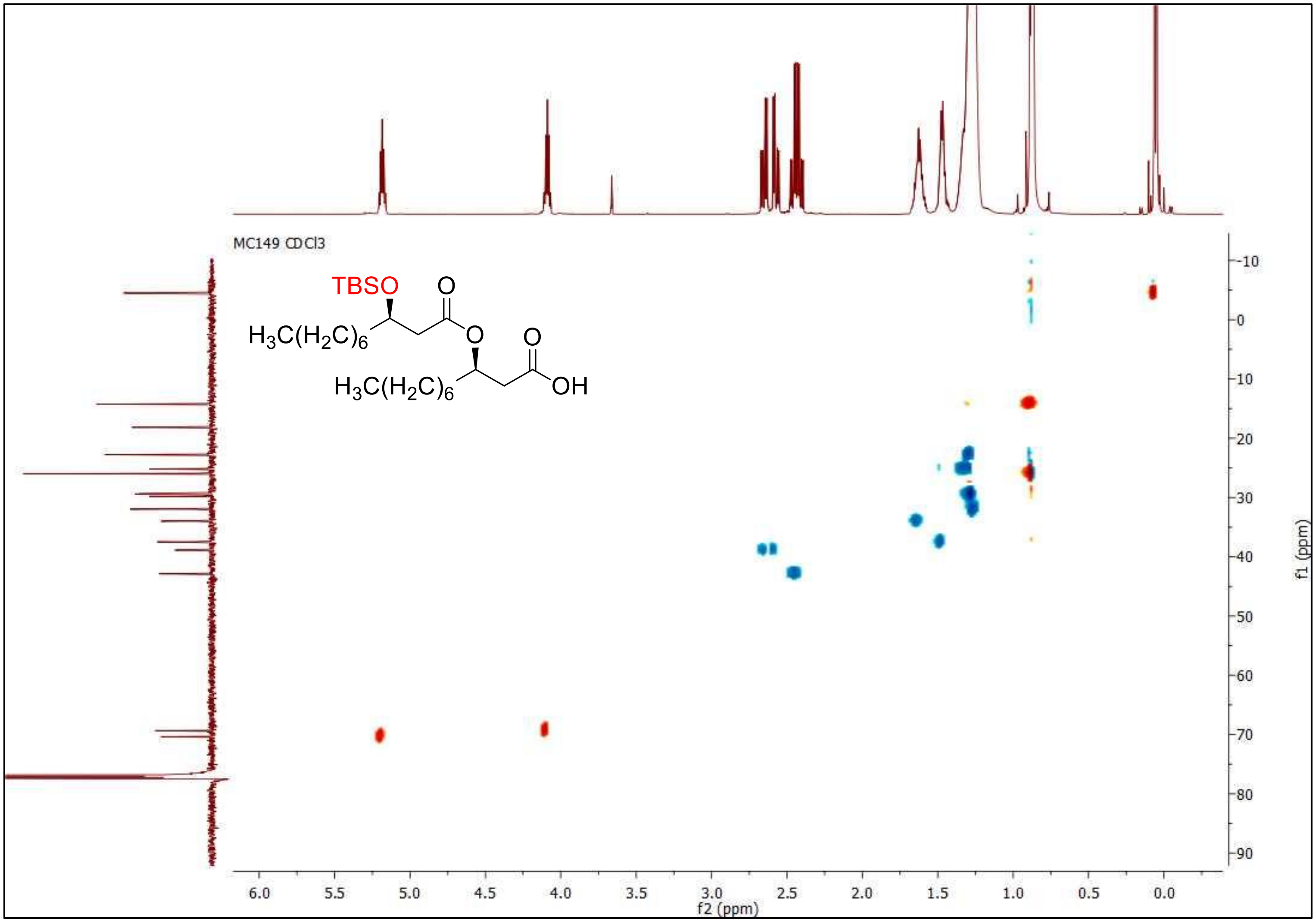

Figure S21 | ^{1}H NMR spectrum ($CDCl_3$, 600 MHz) of (*R*)-benzyl 3-(((*R*)-3-hydroxydecanoyl)oxy)decanoate (**12**).

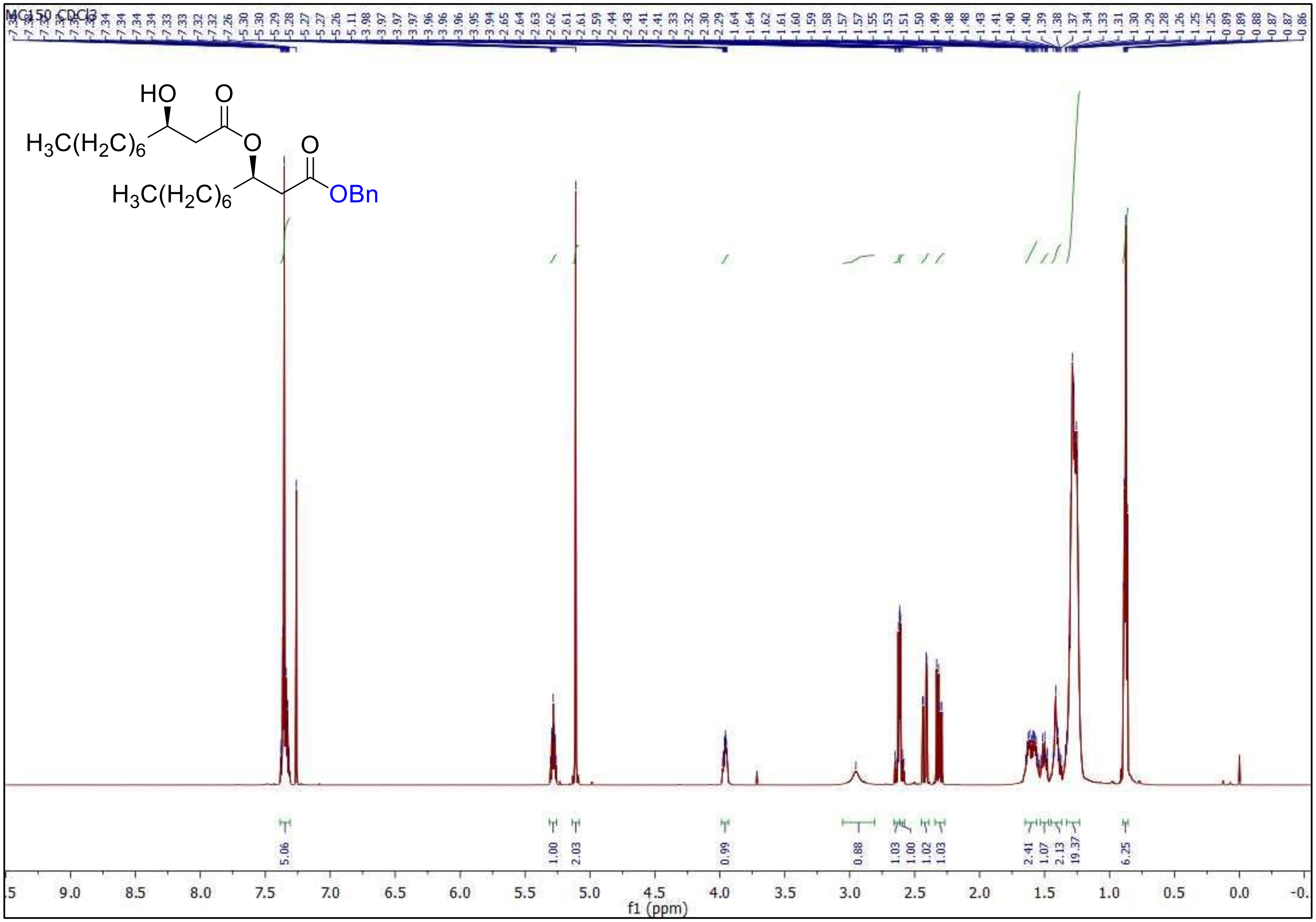

Figure S22 | COSY NMR spectrum ($CDCl_3$, 600 MHz) of (*R*)-benzyl 3-(((*R*)-3-hydroxydecanoyl)oxy)decanoate (**12**).

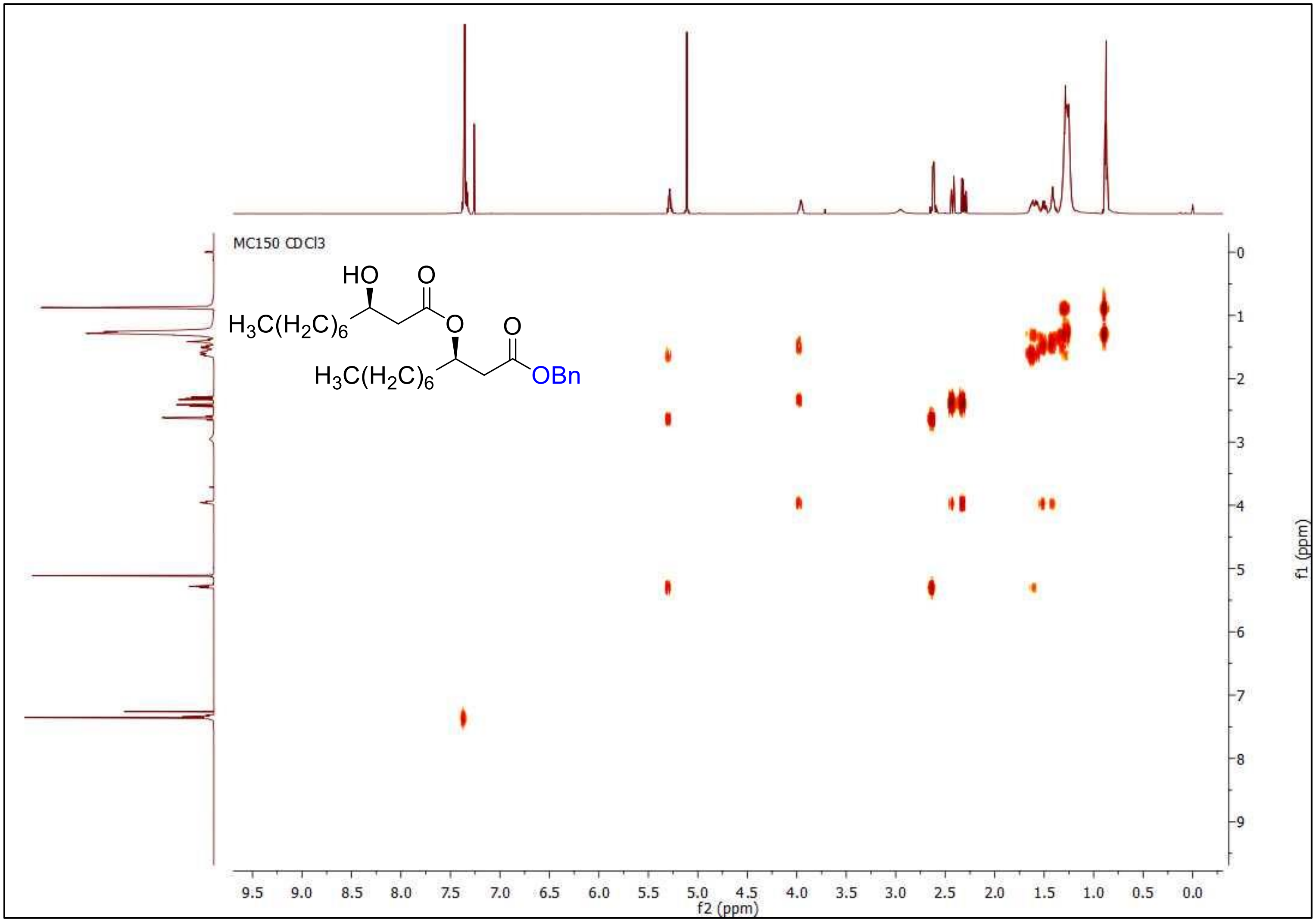

Figure S23 | ^{13}C NMR spectrum ($CDCl_3$, 150 MHz) of (*R*)-benzyl 3-(((*R*)-3-hydroxydecanoyl)oxy)decanoate (**12**).

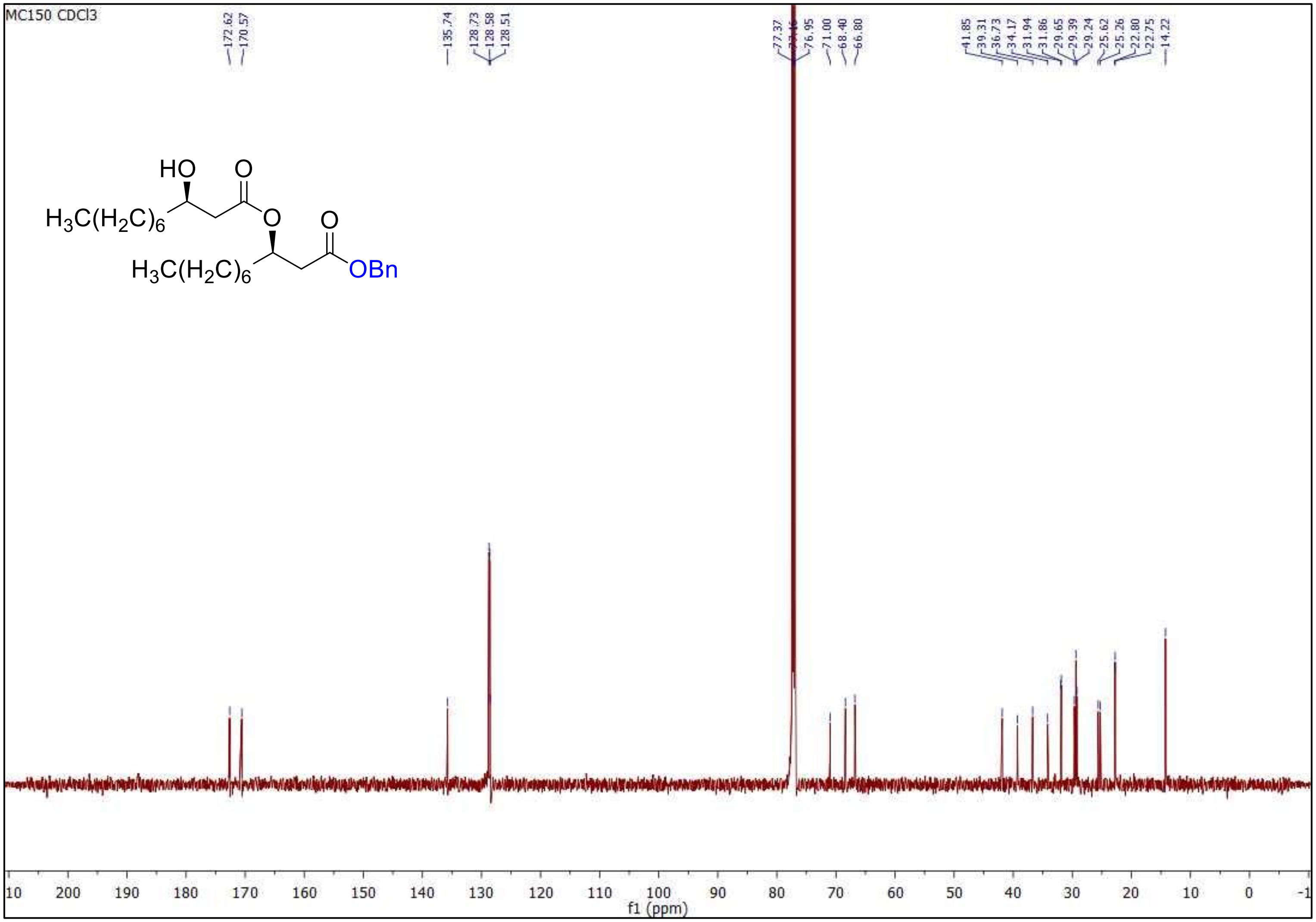

Figure S24 | HSQC NMR spectrum ($CDCl_3$, 600 MHz) of (*R*)-benzyl 3-(((*R*)-3-hydroxydecanoyl)oxy)decanoate (**12**).

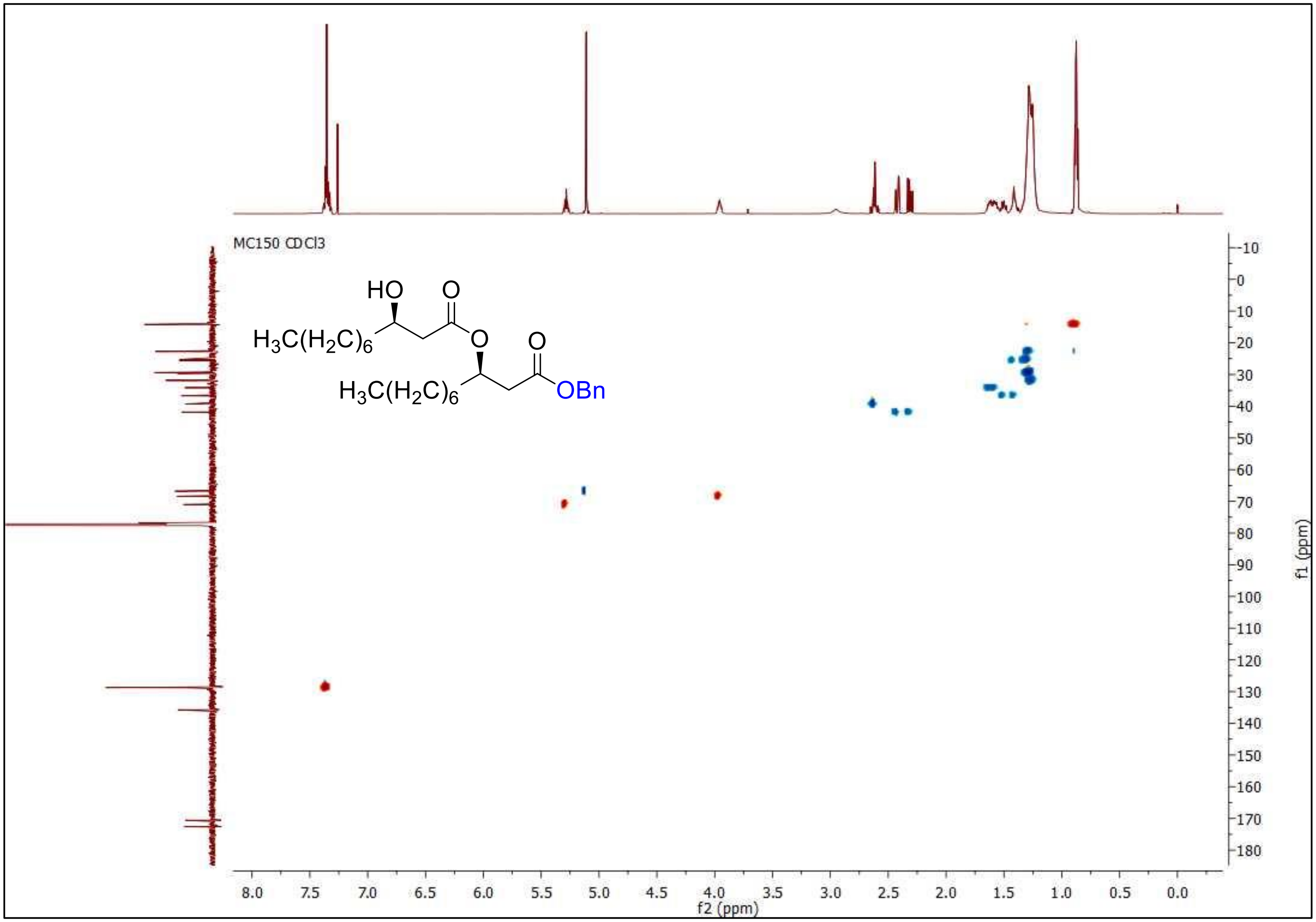

Figure S25 | ^{1}H NMR spectrum ($CDCl_3$, 600 MHz) of *para*-methylphenyl 3,4-di-*O*-benzyl-6-*O*-*tert*-butyldimethylsilyl-1-thio-*β*-D-glucopyranoside (**S10**).

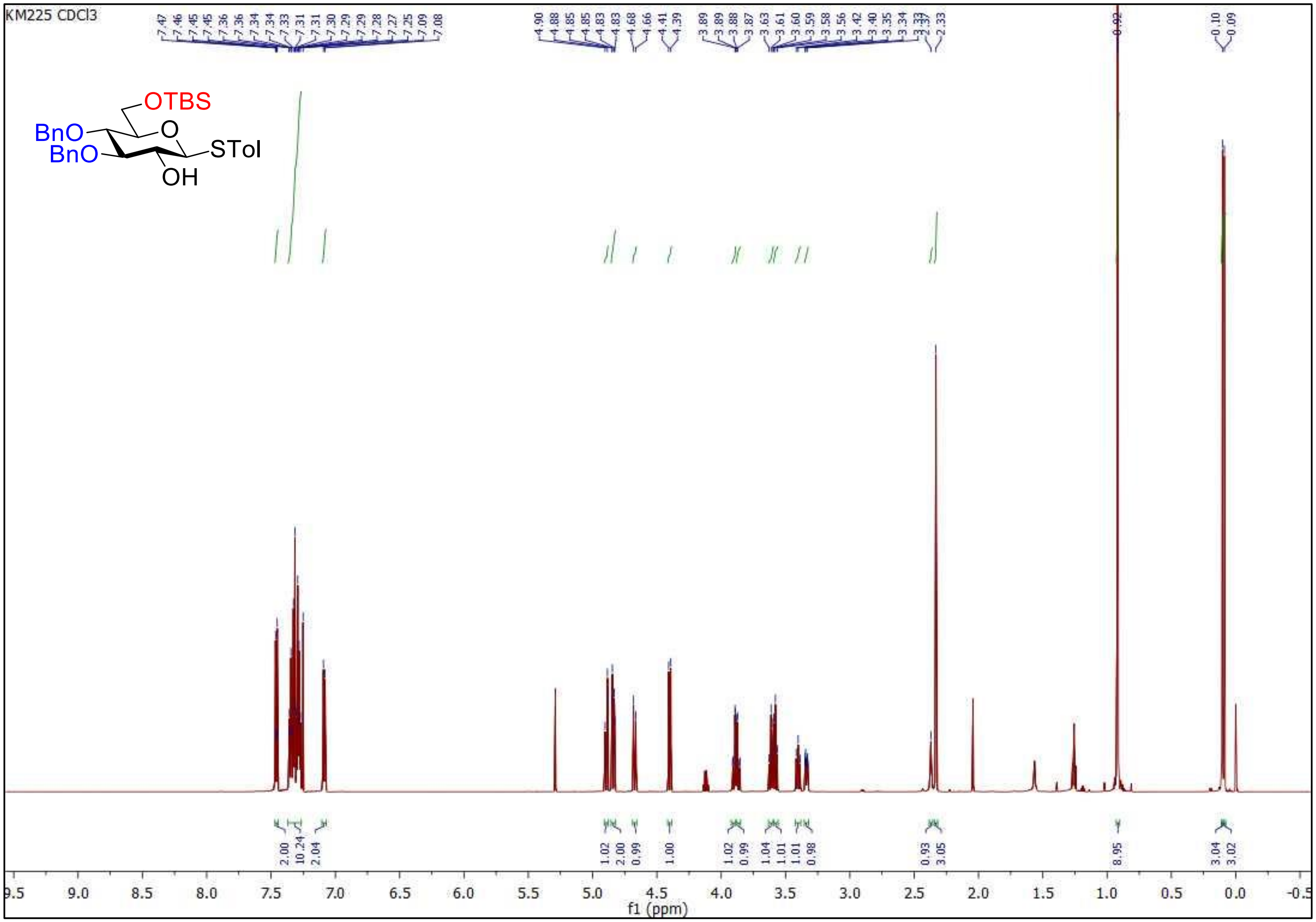

Figure S26 | COSY NMR spectrum ($CDCl_3$, 600 MHz) of *para*-methylphenyl 3,4-di-*O*-benzyl-6-*O*-*tert*-butyldimethylsilyl-1-thio-*β*-D-glucopyranoside (**S10**).

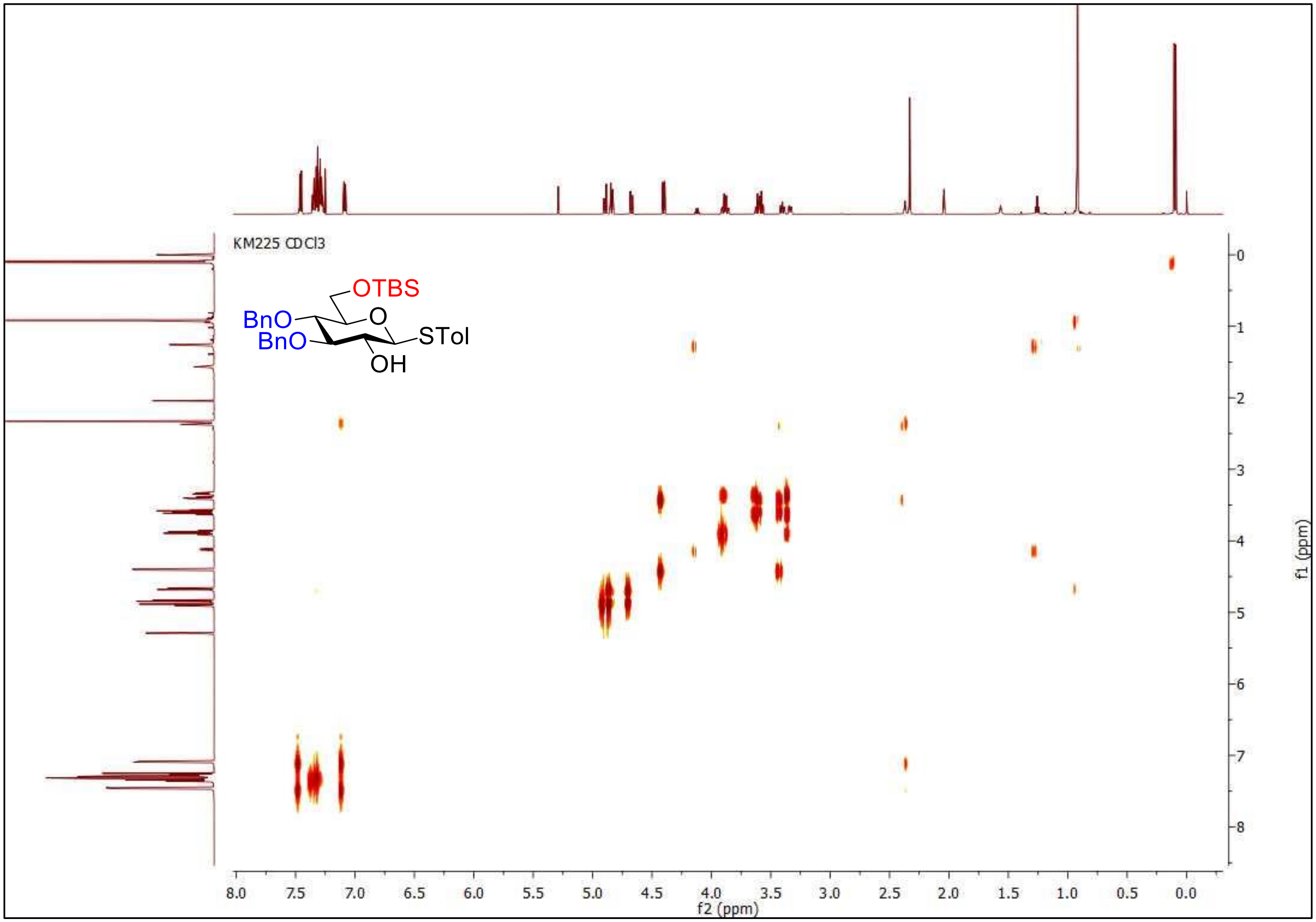

Figure S27 | ^{13}C NMR spectrum ($CDCl_3$, 600 MHz) of *para*-methylphenyl 3,4-di-*O*-benzyl-6-*O*-*tert*-butyldimethylsilyl-1-thio-*β*-D-glucopyranoside (**S10**).

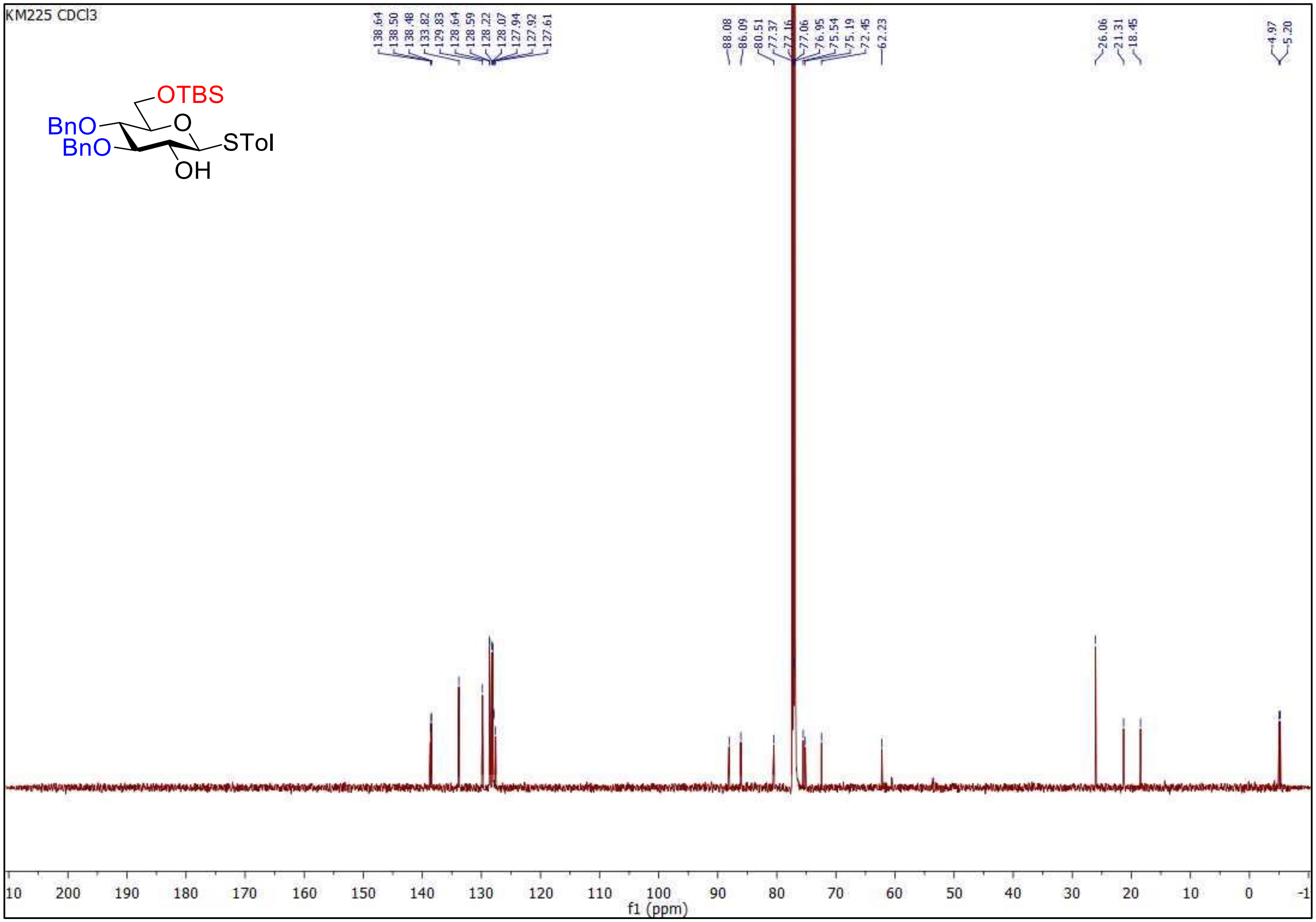

Figure S28 | HSQC NMR spectrum ($CDCl_3$, 600 MHz) of *para*-methylphenyl 3,4-di-*O*-benzyl-6-*O*-*tert*-butyldimethylsilyl-1-thio-*β*-D-glucopyranoside (**S10**).

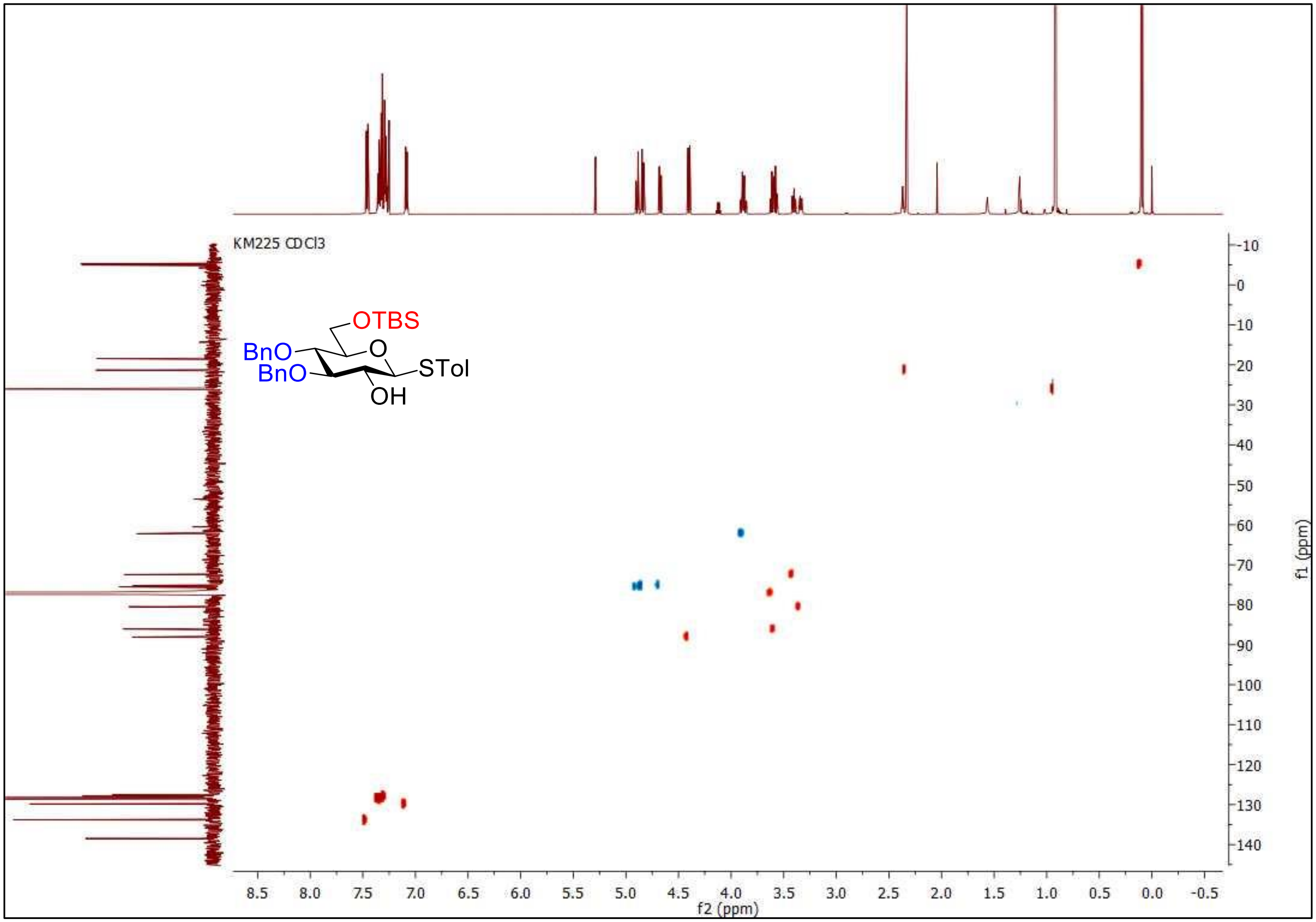

Figure S29 | ^{1}H NMR spectrum ($CDCl_3$, 600 MHz) of *para*-methylphenyl 3,4-di-*O*-benzyl-6-*O*-*tert*-butyldimethylsilyl-2-*O*-levulinoyl-1-thio-*β*-D-glucopyranoside (**11**).

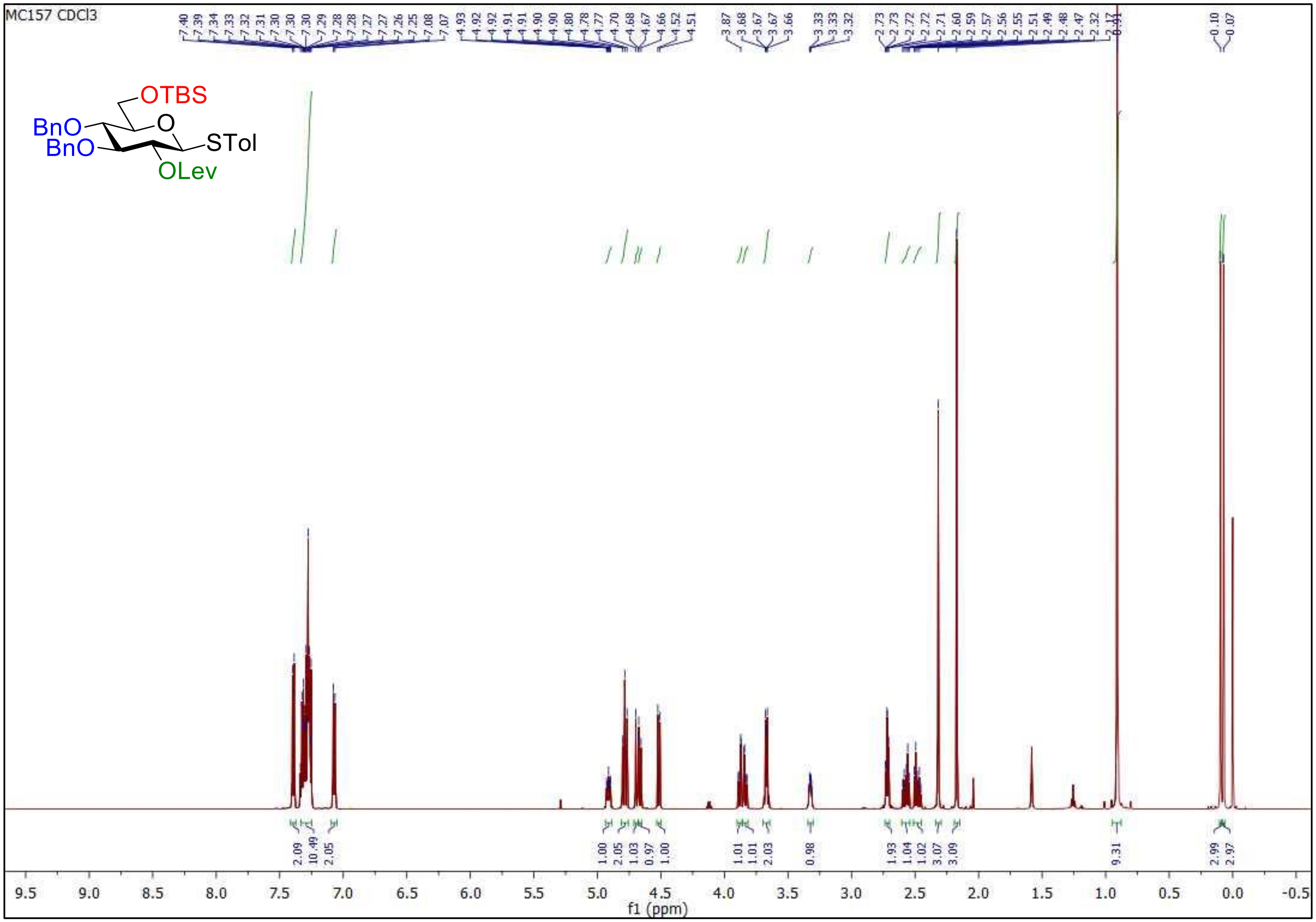

Figure S30 | COSY NMR spectrum ($CDCl_3$, 600 MHz) of *para*-methylphenyl 3,4-di-*O*-benzyl-6-*O*-*tert*-butyldimethylsilyl-2-*O*-levulinoyl-1-thio-*β*-D-glucopyranoside (**11**).

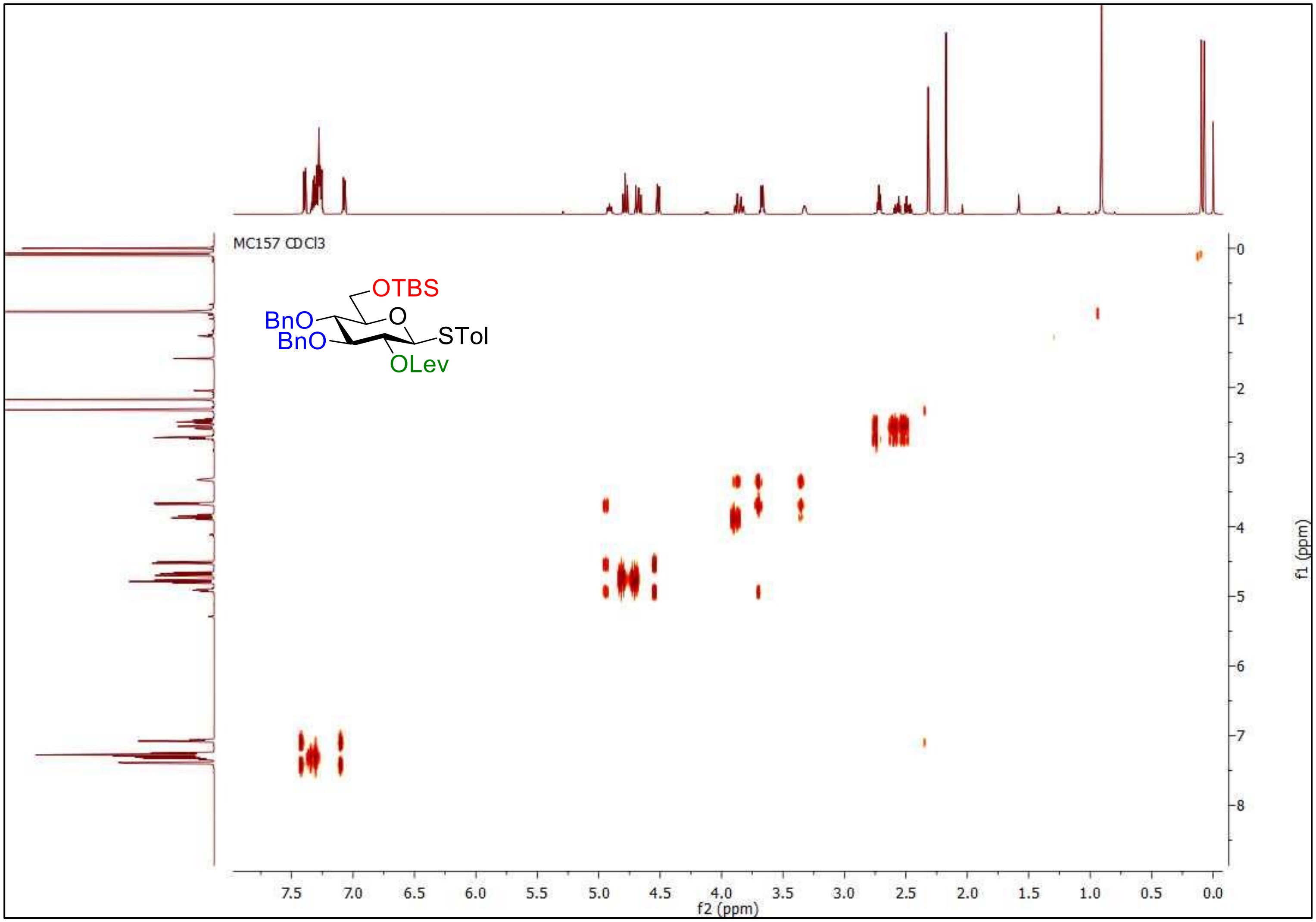

Figure S31 | ^{13}C NMR spectrum ($CDCl_3$, 600 MHz) of *para*-methylphenyl 3,4-di-*O*-benzyl-6-*O*-*tert*-butyldimethylsilyl-2-*O*-levulinoyl-1-thio-*β*-D-glucopyranoside (**11**).

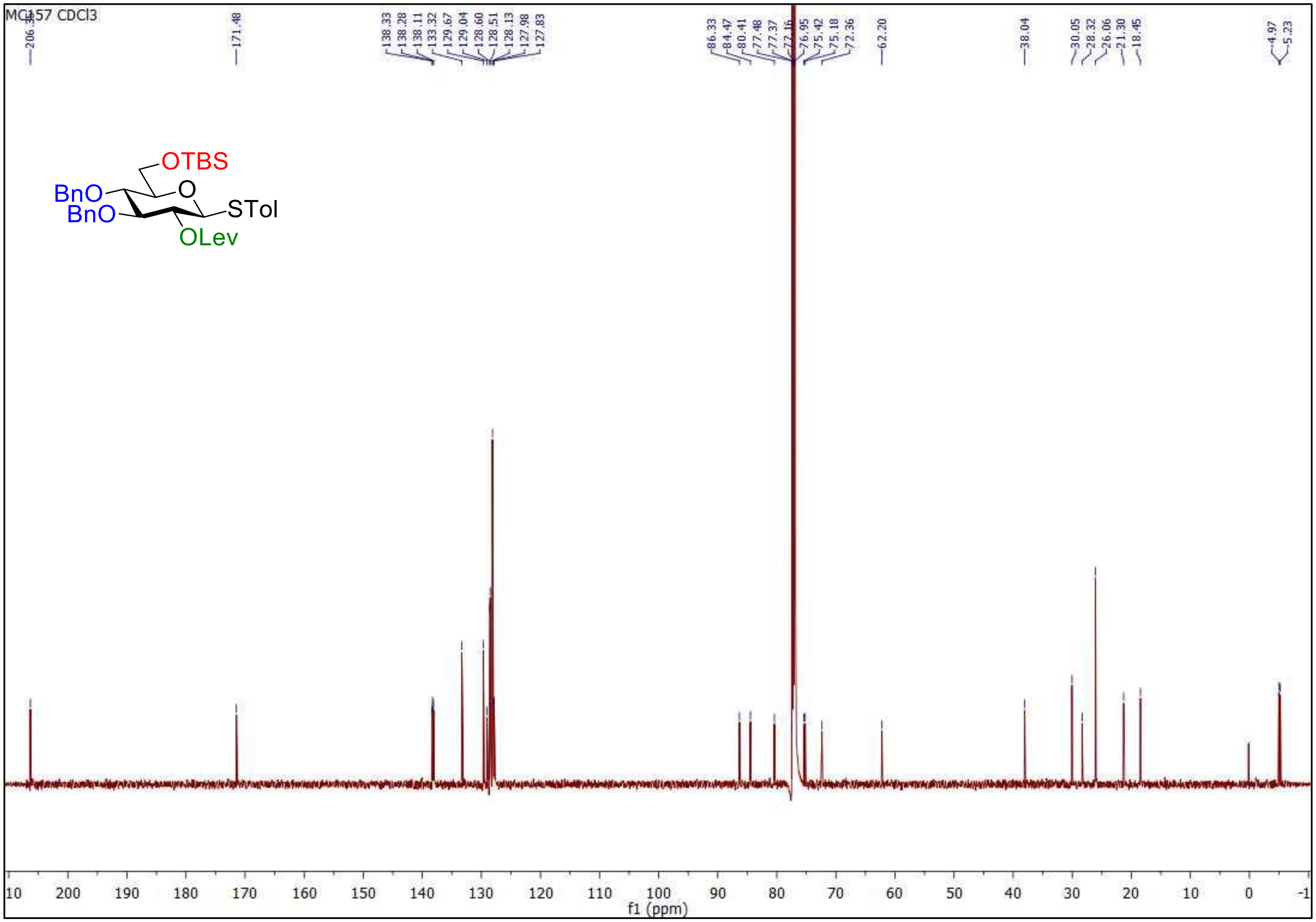

Figure S32 | HSQC NMR spectrum ($CDCl_3$, 600 MHz) of *para*-methylphenyl 3,4-di-*O*-benzyl-6-*O*-*tert*-butyldimethylsilyl-2-*O*-levulinoyl-1-thio-*β*-D-glucopyranoside (**11**).

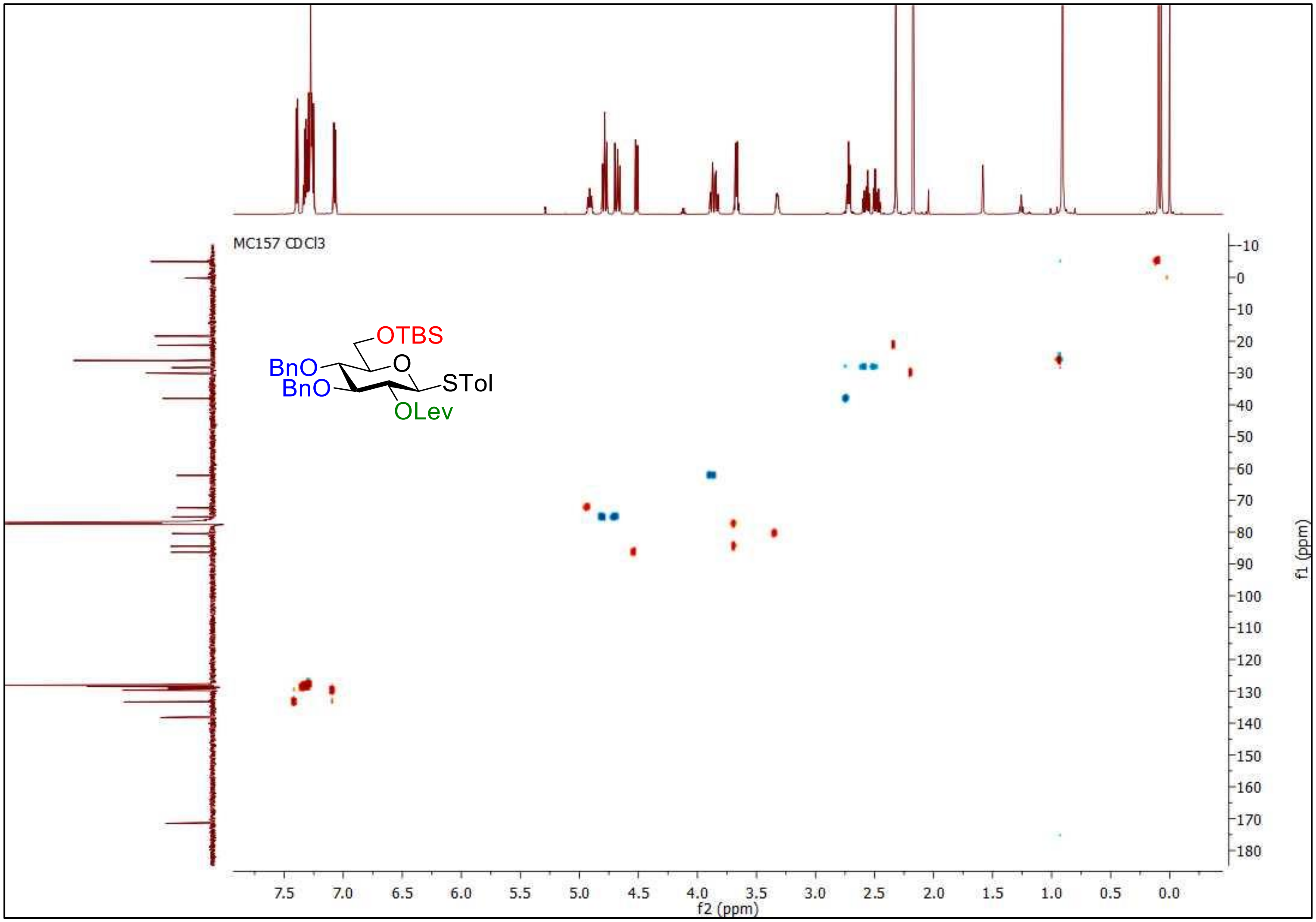

Figure S33 | ^{1}H NMR spectrum ($CDCl_3$, 600 MHz) of benzyl (*R*)-3-*O*-[(*R*)-(3'-*O*-decyl)-3,4-di-*O*-benzyl-6-*O*-*tert*-butyldimethylsilyl-2-*O*-levulinoyl-*β*-D-glucopyranosyl]decanoate (**8**).

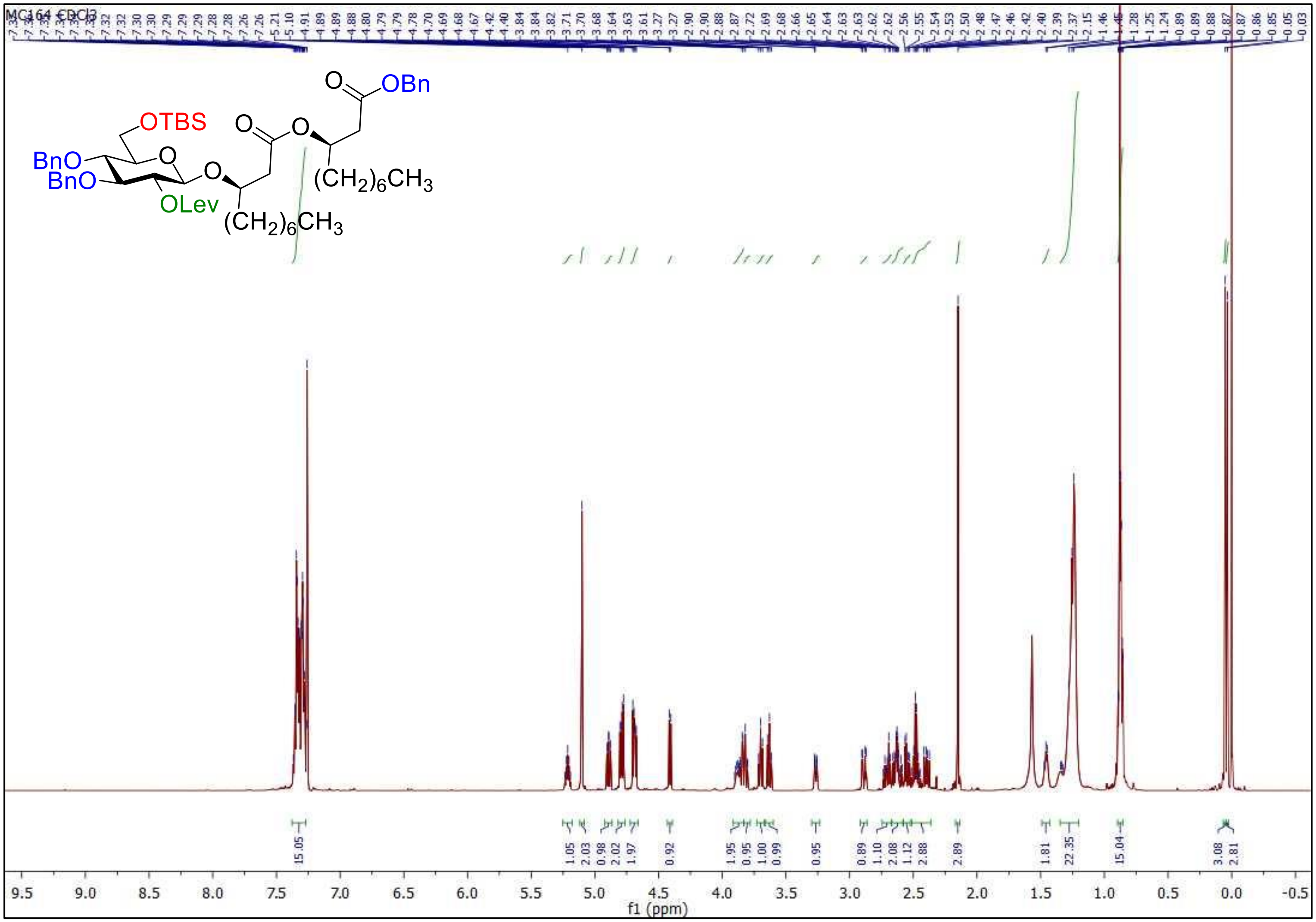

Figure S34 | COSY NMR spectrum ($CDCl_3$, 600 MHz) of benzyl (*R*)-3-*O*-[(*R*)-(3'-*O*-decyl)-3,4-di-*O*-benzyl-6-*O*-*tert*-butyldimethylsilyl-2-*O*-levulinoyl-*β*-D-glucopyranosyl]decanoate (**8**).

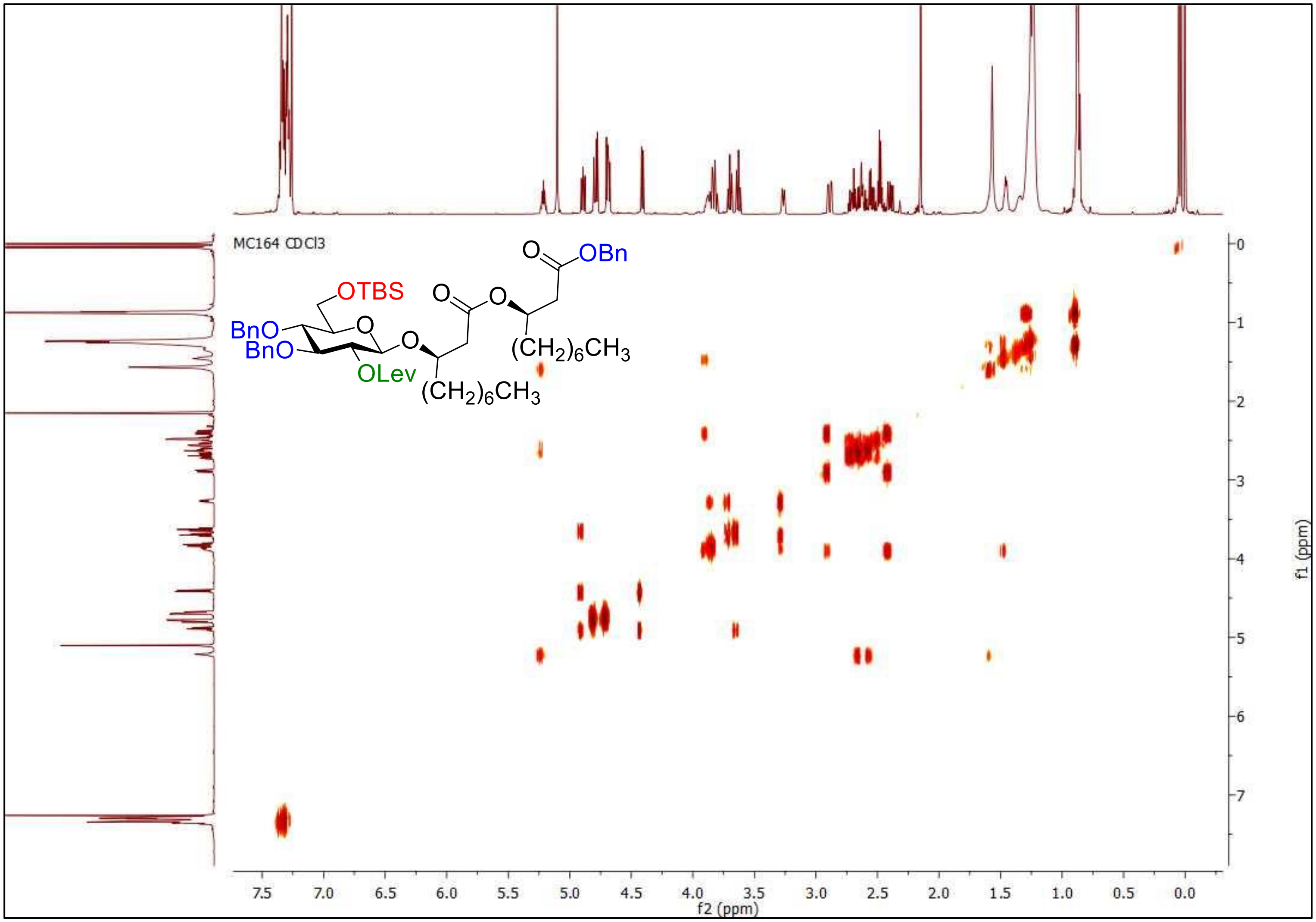

Figure S35 | ^{13}C NMR spectrum ($CDCl_3$, 600 MHz) of benzyl (*R*)-3-*O*-[(*R*)-(3'-*O*-decyl)-3,4-di-*O*-benzyl-6-*O*-*tert*-butyldimethylsilyl-2-*O*-levulinoyl-*β*-D-glucopyranosyl]decanoate (**8**).

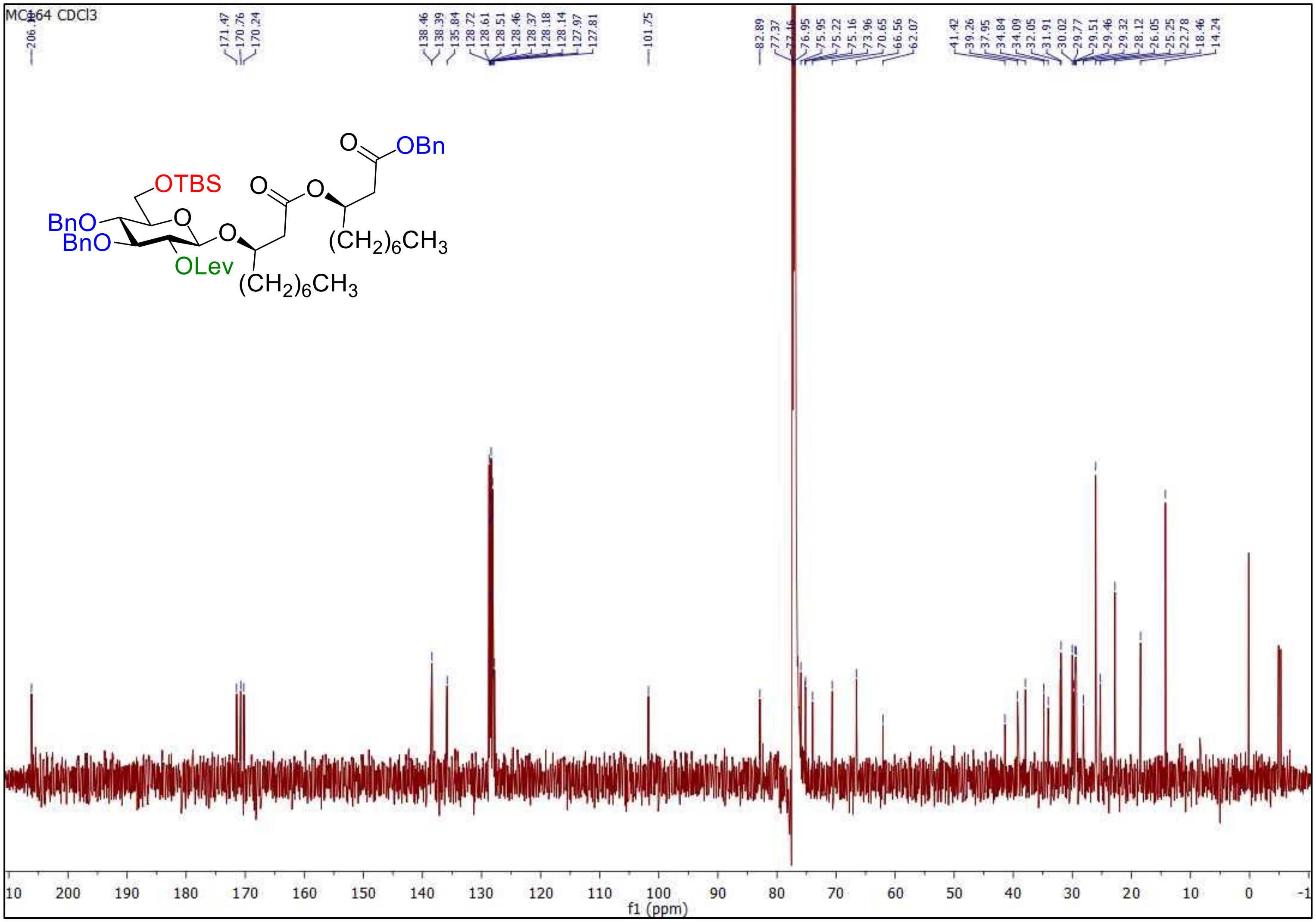

Figure S36 | HSQC NMR spectrum ($CDCl_3$, 600 MHz) of benzyl (*R*)-3-*O*-[(*R*)-(3'-*O*-decyl)-3,4-di-*O*-benzyl-6-*O*-*tert*-butyldimethylsilyl-2-*O*-levulinoyl-*β*-D-glucopyranosyl]decanoate (**8**).

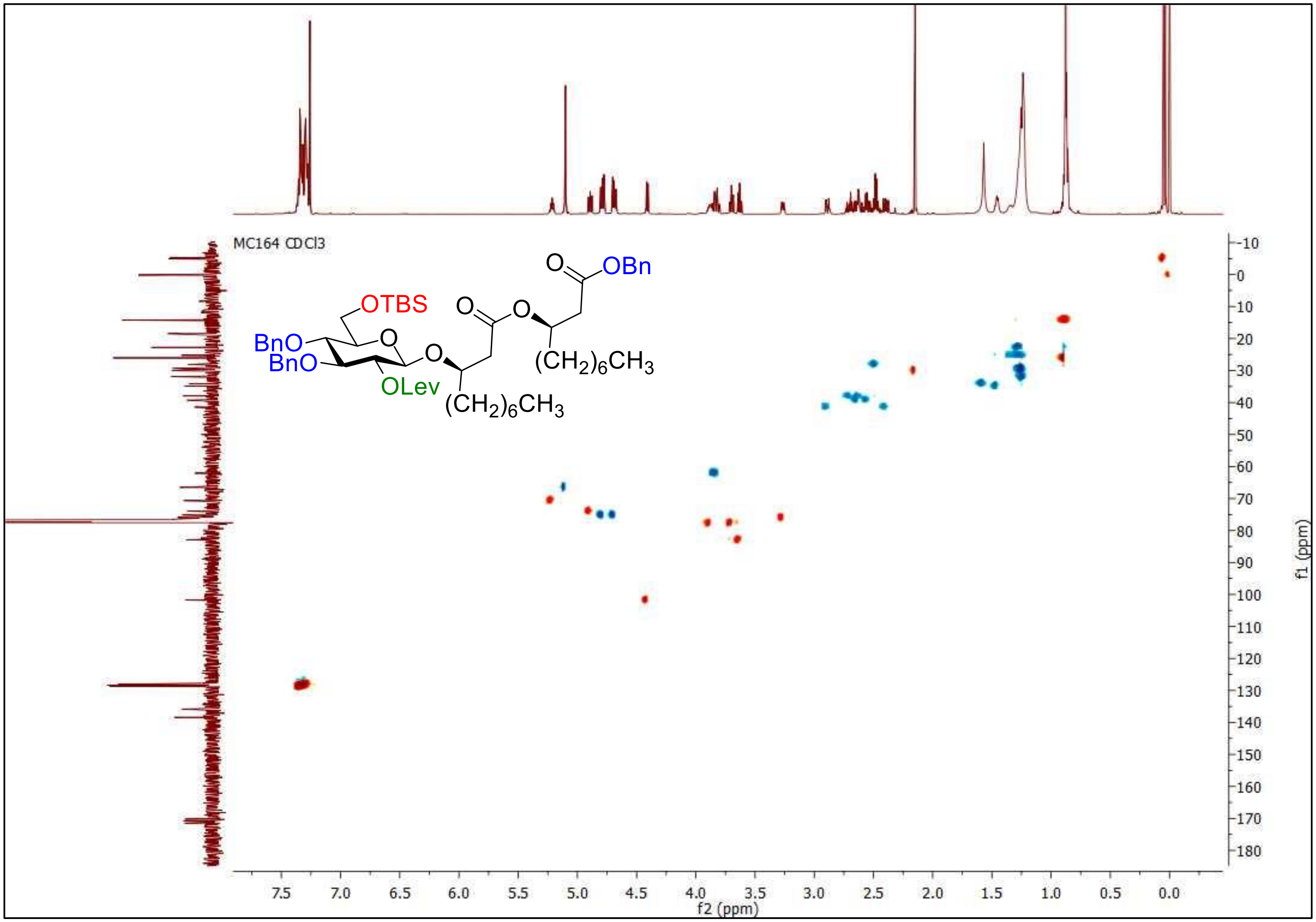

Figure S37 | ^{1}H NMR spectrum ($CDCl_3$, 600 MHz) of benzyl (*R*)-3-*O*-[(*R*)-(3'-*O*-decyl)-3,4-di-*O*-benzyl-2-*O*-levulinoyl-*β*-D-glucopyranosyl]decanoate (**18**).

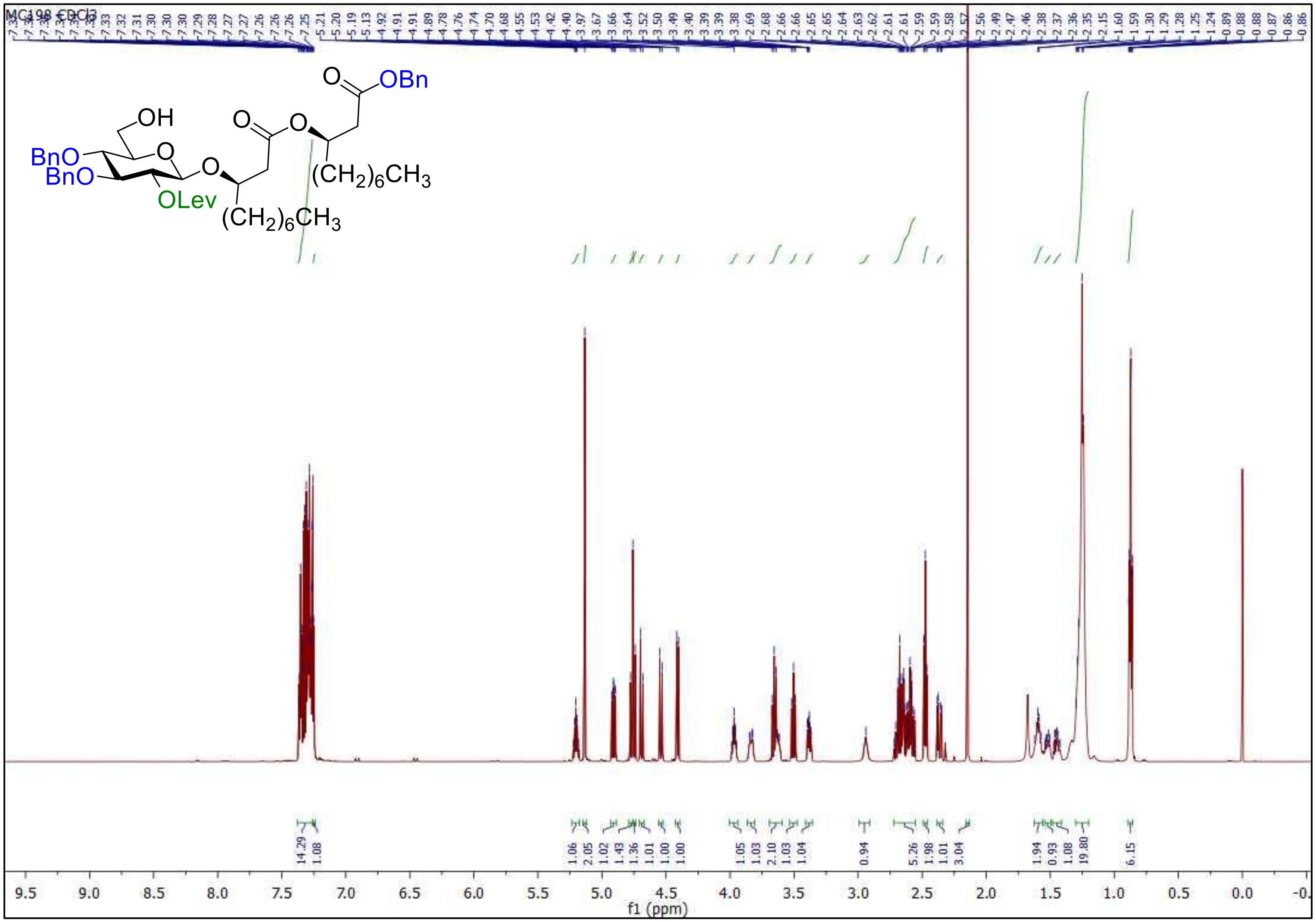

Figure S38 | COSY NMR spectrum ($CDCl_3$, 600 MHz) of benzyl (*R*)-3-*O*-[(*R*)-(3'-*O*-decyl)-3,4-di-*O*-benzyl-2-*O*-levulinoyl-*β*-D-glucopyranosyl]decanoate (**18**).

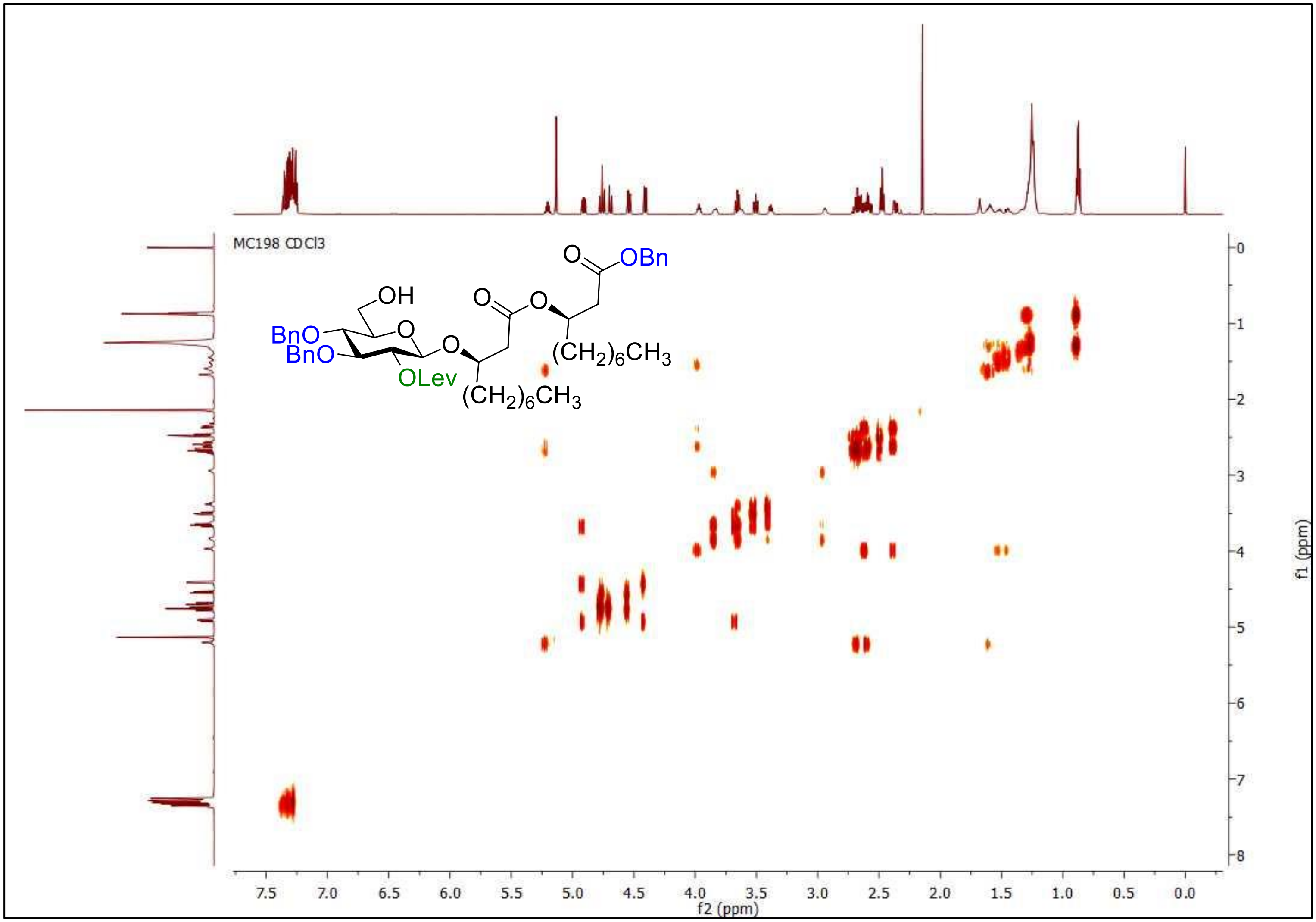

Figure S39 | ^{13}C NMR spectrum (CDCl$_3$, 600 MHz) of benzyl (*R*)-3-*O*-[(*R*)-(3'-*O*-decyl)-3,4-di-*O*-benzyl-2-*O*-levulinoyl-*β*-D-glucopyranosyl]decanoate (**18**).

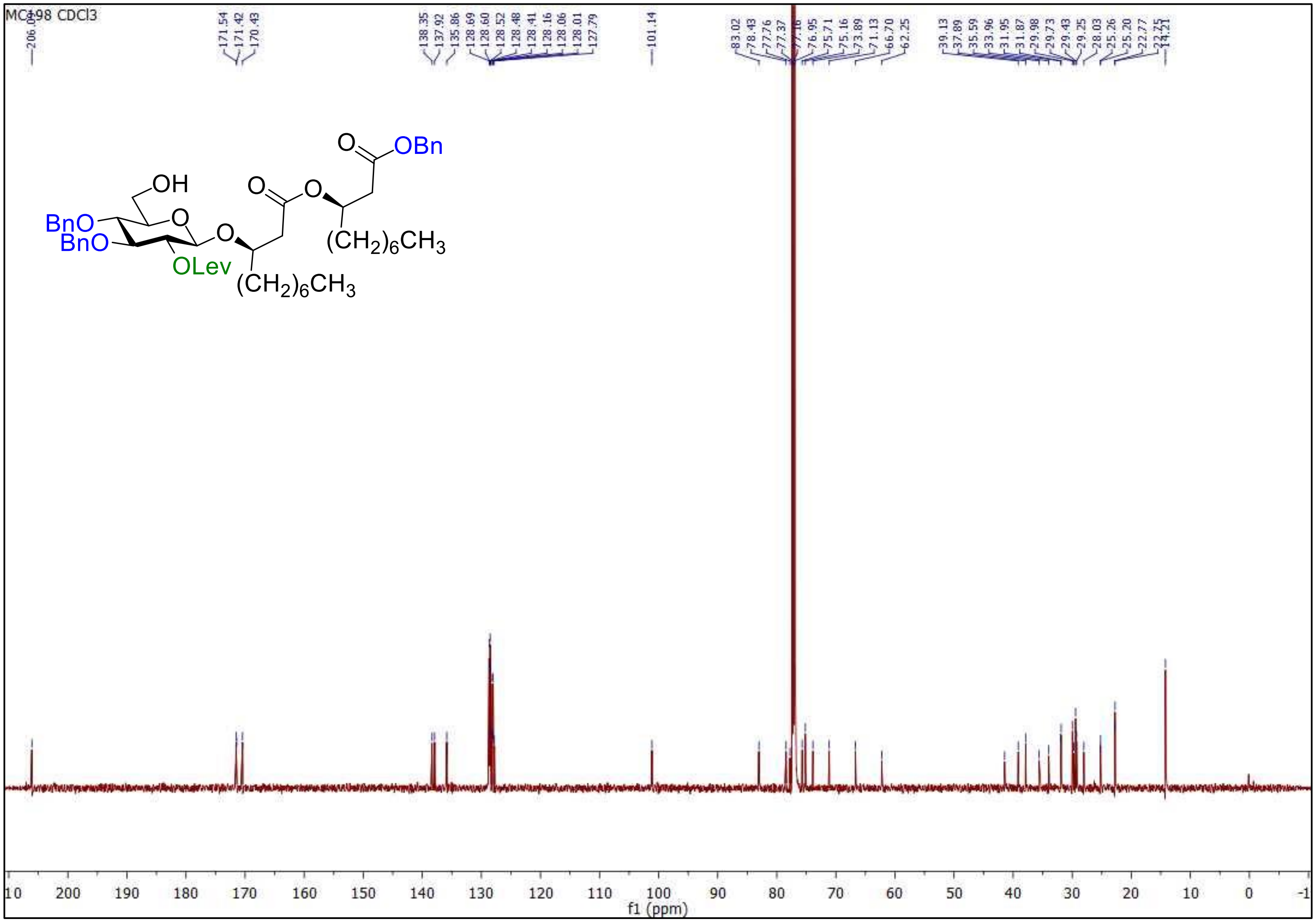

Figure S40 | HSQC NMR spectrum ($CDCl_3$, 600 MHz) of benzyl (*R*)-3-*O*-[(*R*)-(3'-*O*-decyl)-3,4-di-*O*-benzyl-2-*O*-levulinoyl-*β*-D-glucopyranosyl]decanoate (**18**).

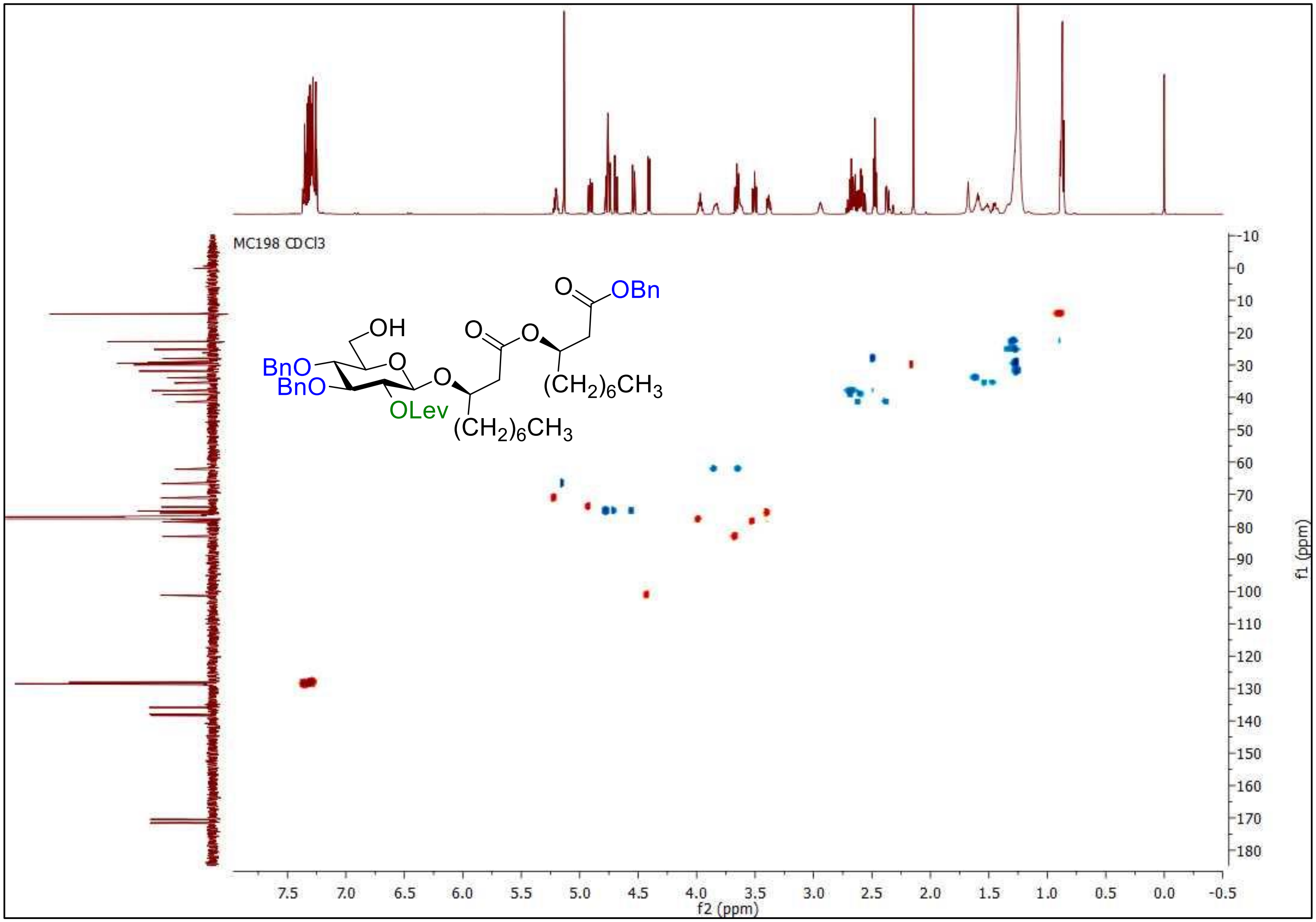

Figure S41 | ^{1}H NMR spectrum ($CDCl_3$, 600 MHz) of benzyl (*R*)-3-*O*-[(*R*)-(3'-*O*-decyl)-3,4-di-*O*-benzyl-*β*-D-glucopyranosyl]decanoate (**19**).

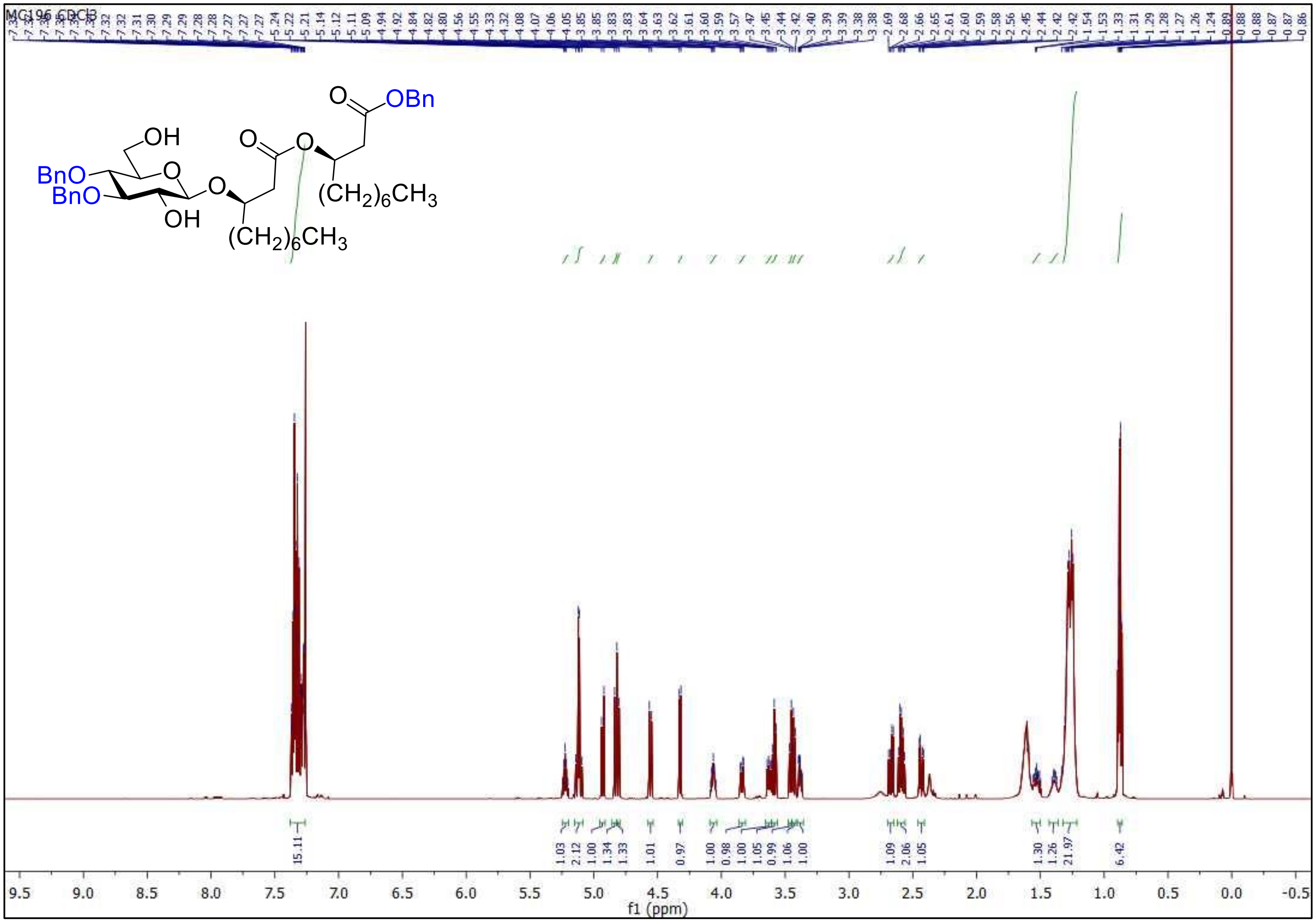

Figure S42 | COSY NMR spectrum ($CDCl_3$, 600 MHz) of benzyl (*R*)-3-*O*-[(*R*)-(3'-*O*-decyl)-3,4-di-*O*-benzyl-*β*-D-glucopyranosyl]decanoate (**19**).

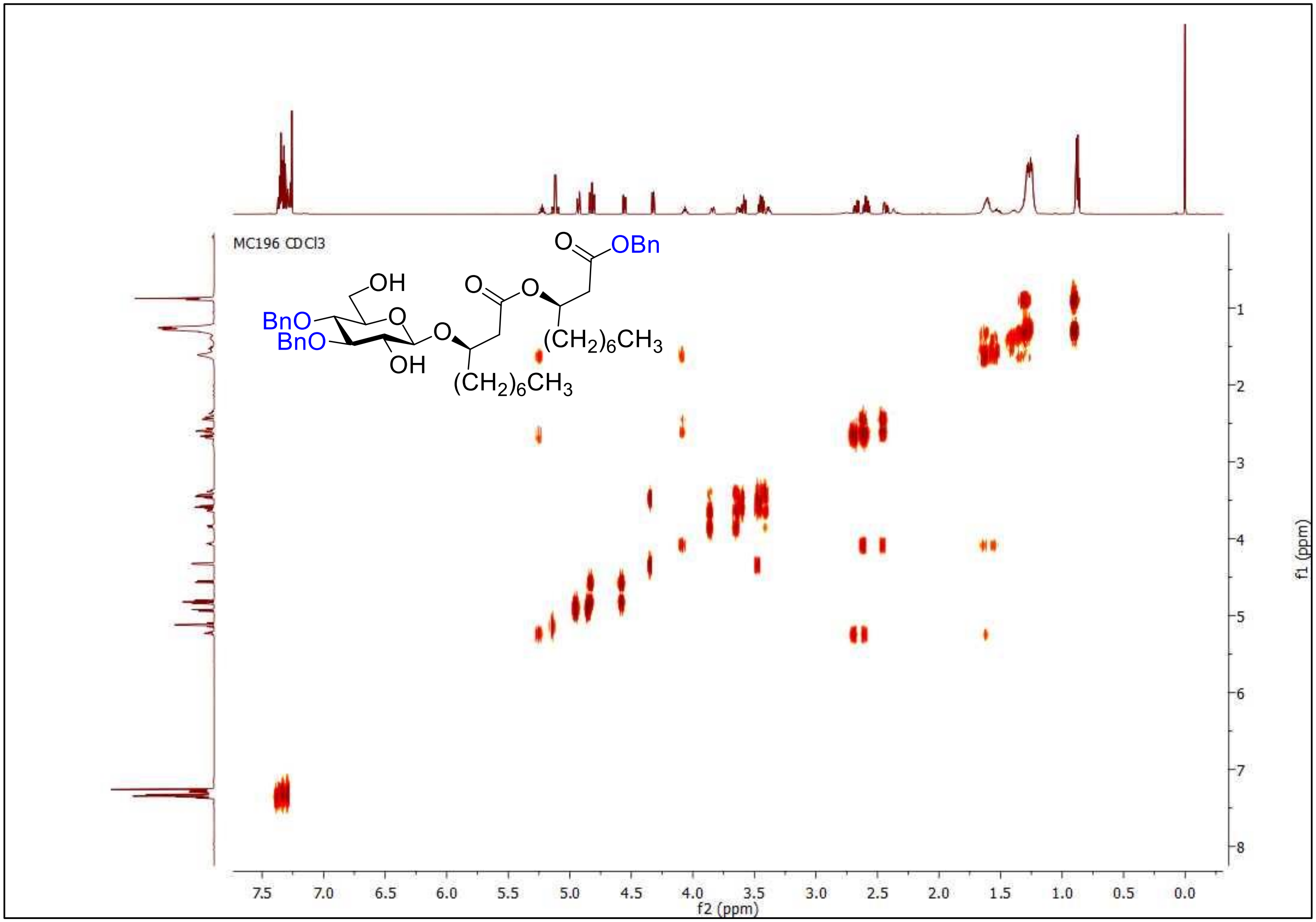

Figure S43 | ^{13}C NMR spectrum ($CDCl_3$, 600 MHz) of benzyl (*R*)-3-*O*-[(*R*)-(3'-*O*-decyl)-3,4-di-*O*-benzyl-*β*-D-glucopyranosyl]decanoate (**19**).

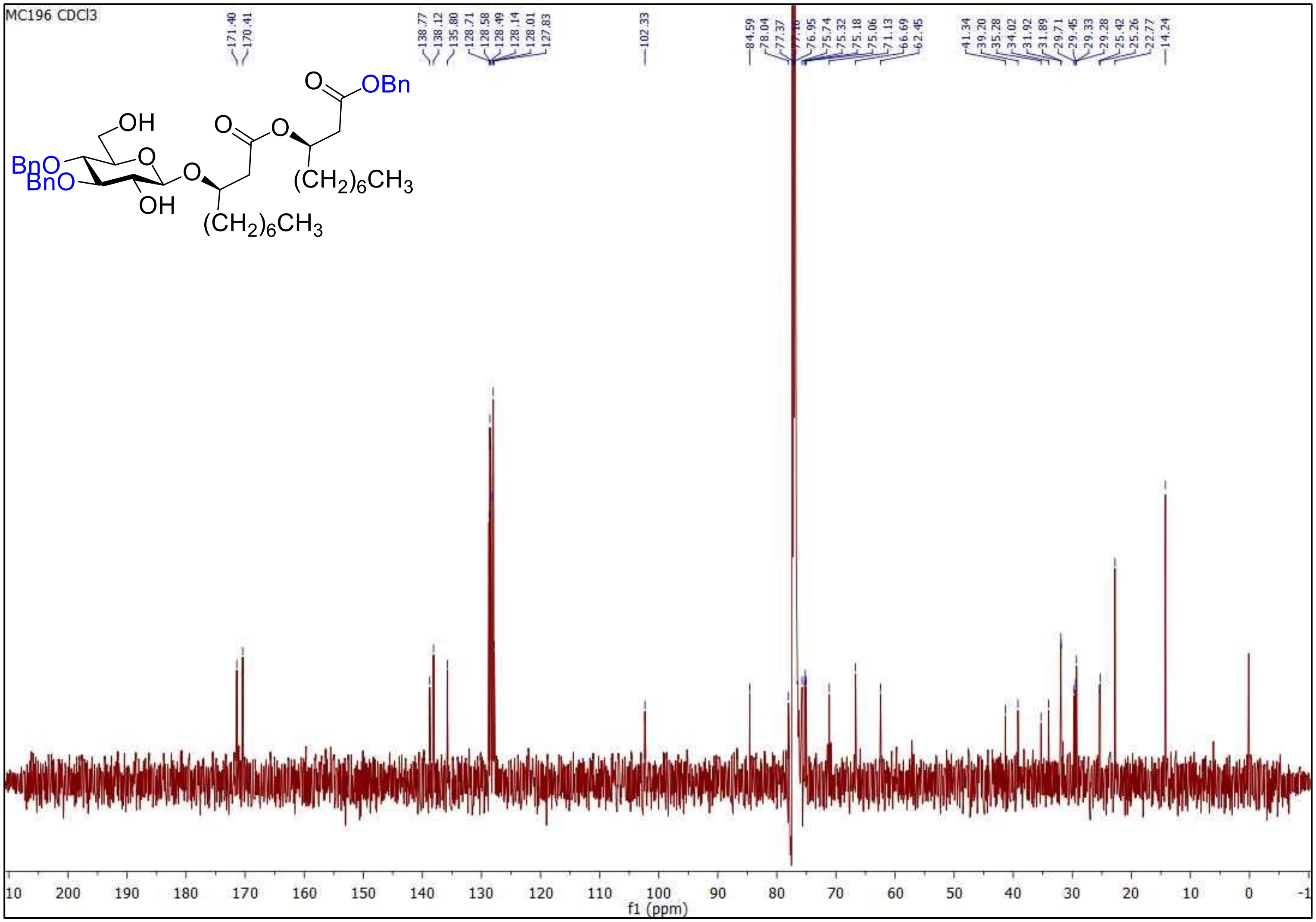

Figure S44 | HSQC NMR spectrum ($CDCl_3$, 600 MHz) of benzyl (*R*)-3-*O*-[(*R*)-(3'-*O*-decyl)-3,4-di-*O*-benzyl-*β*-D-glucopyranosyl]decanoate (**19**).

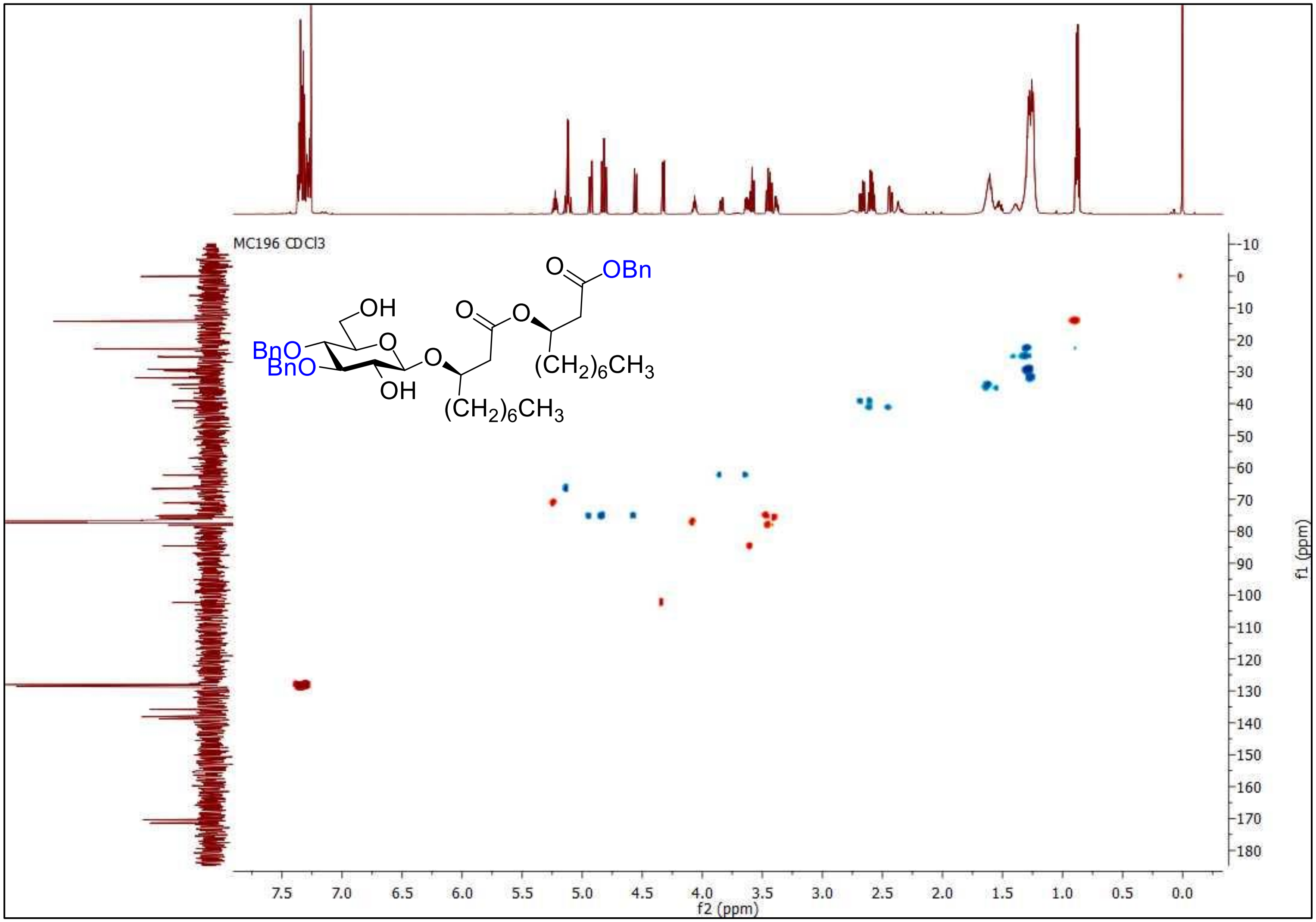

Figure S45 | ^{1}H NMR spectrum (pyr-d_5, 600 MHz) of synthetic ananatoside B (**2**).

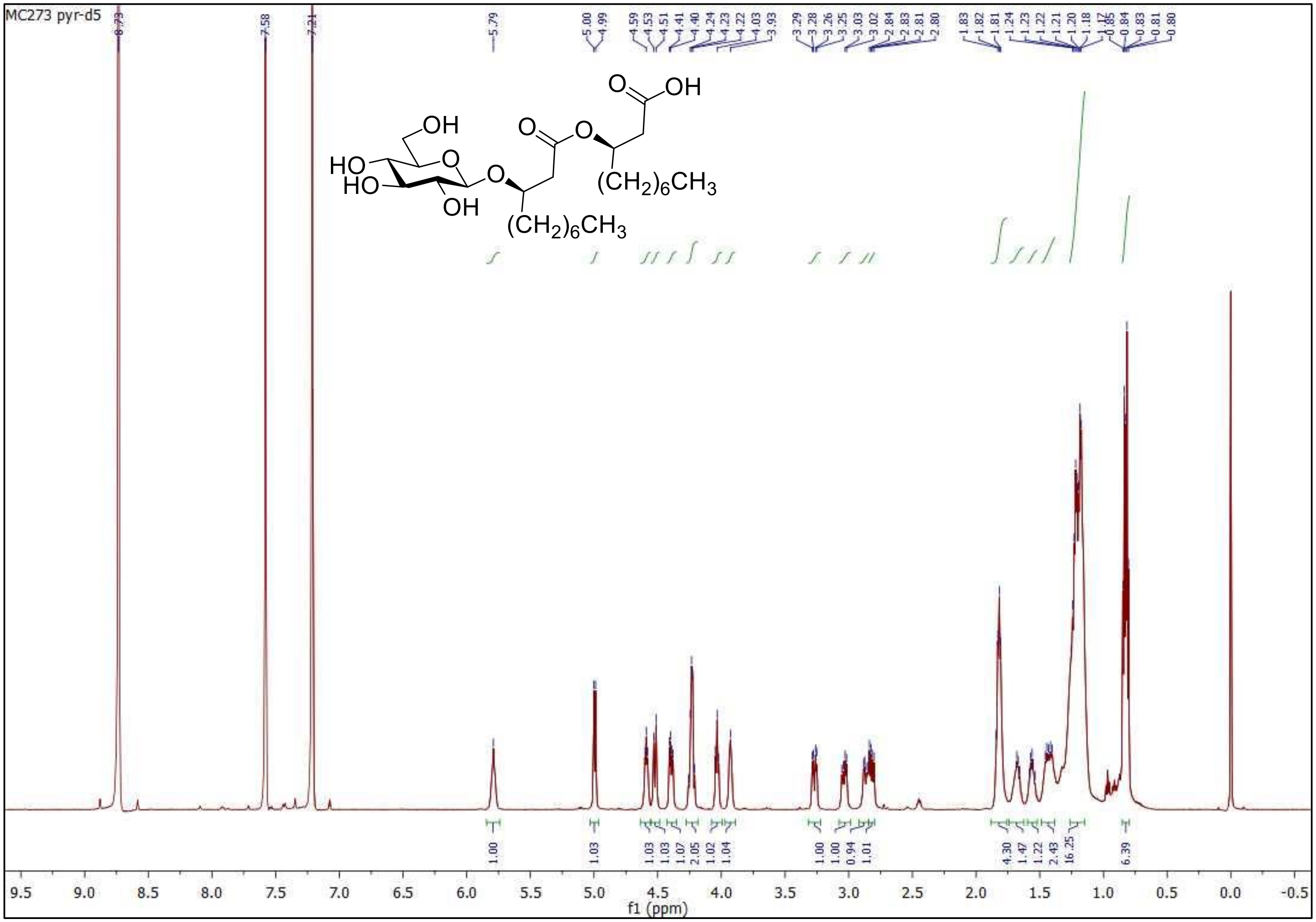

Figure S46 | COSY NMR spectrum (pyr-d_5, 600 MHz) of synthetic ananatoside B (**2**).

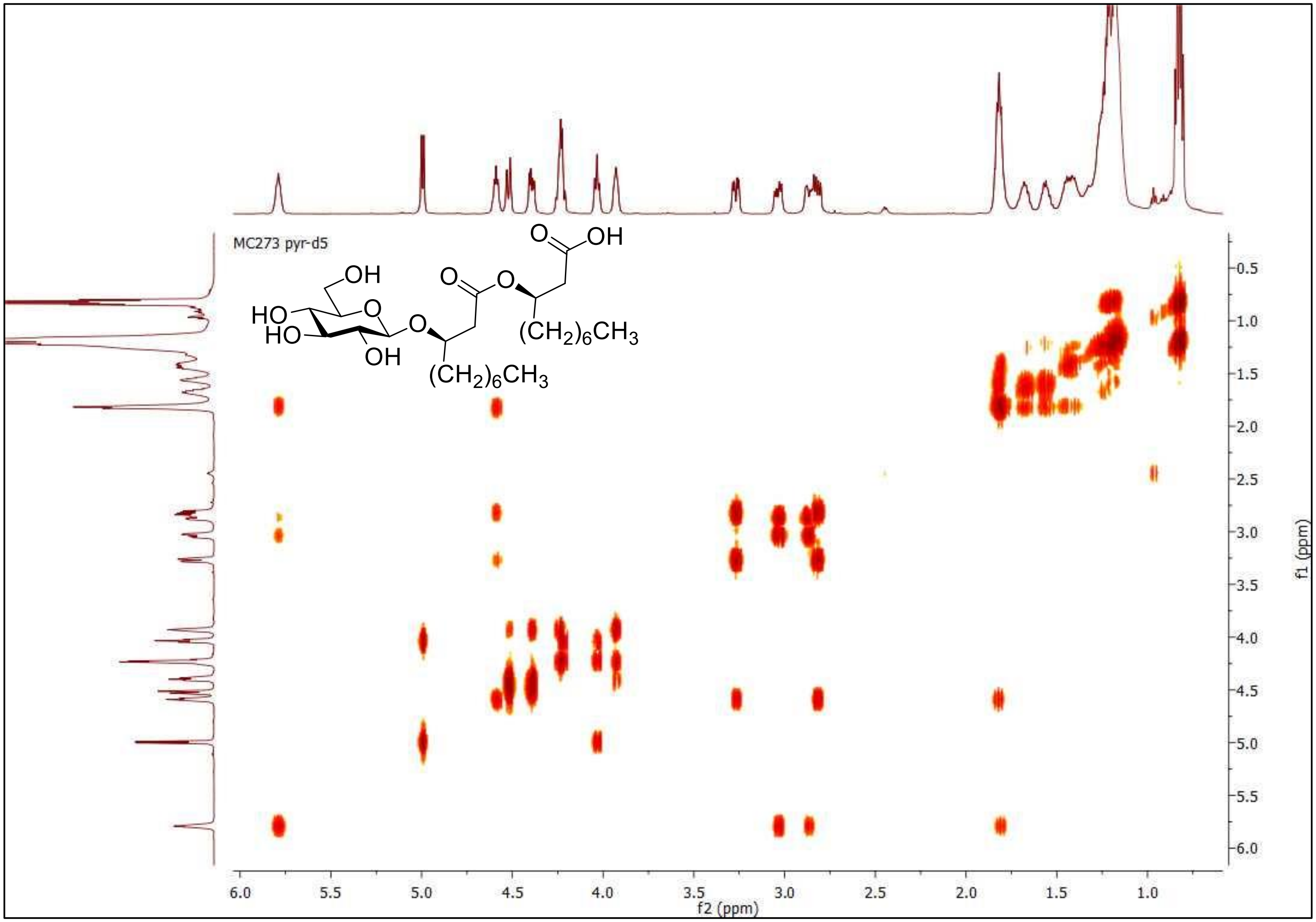

Figure S47 | ^{13}C NMR spectrum (pyr-d_5, 600 MHz) of synthetic ananatoside B (**2**).

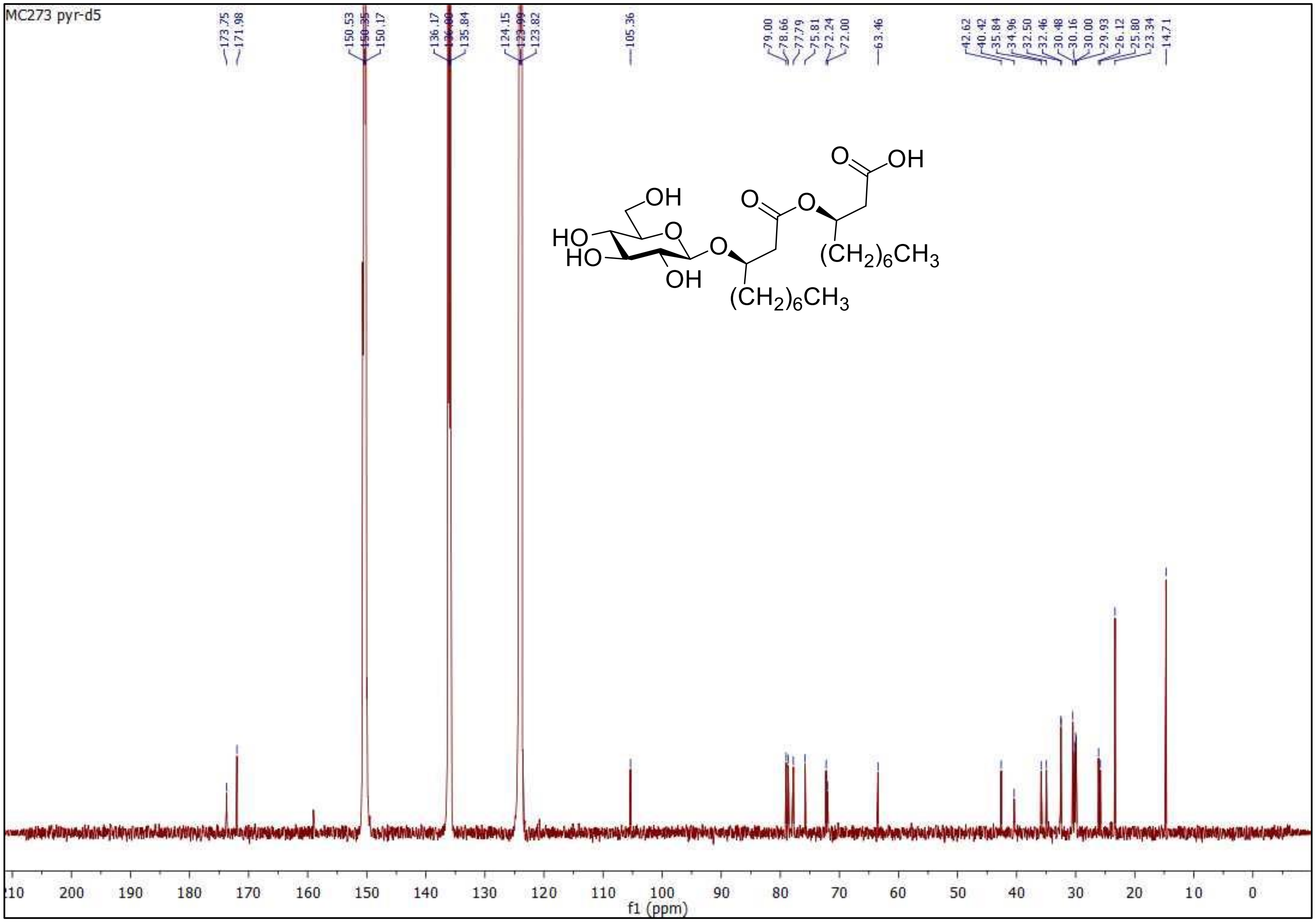

Figure S48 | HSQC NMR spectrum (pyr-d_5, 600 MHz) of synthetic ananatoside B (**2**).

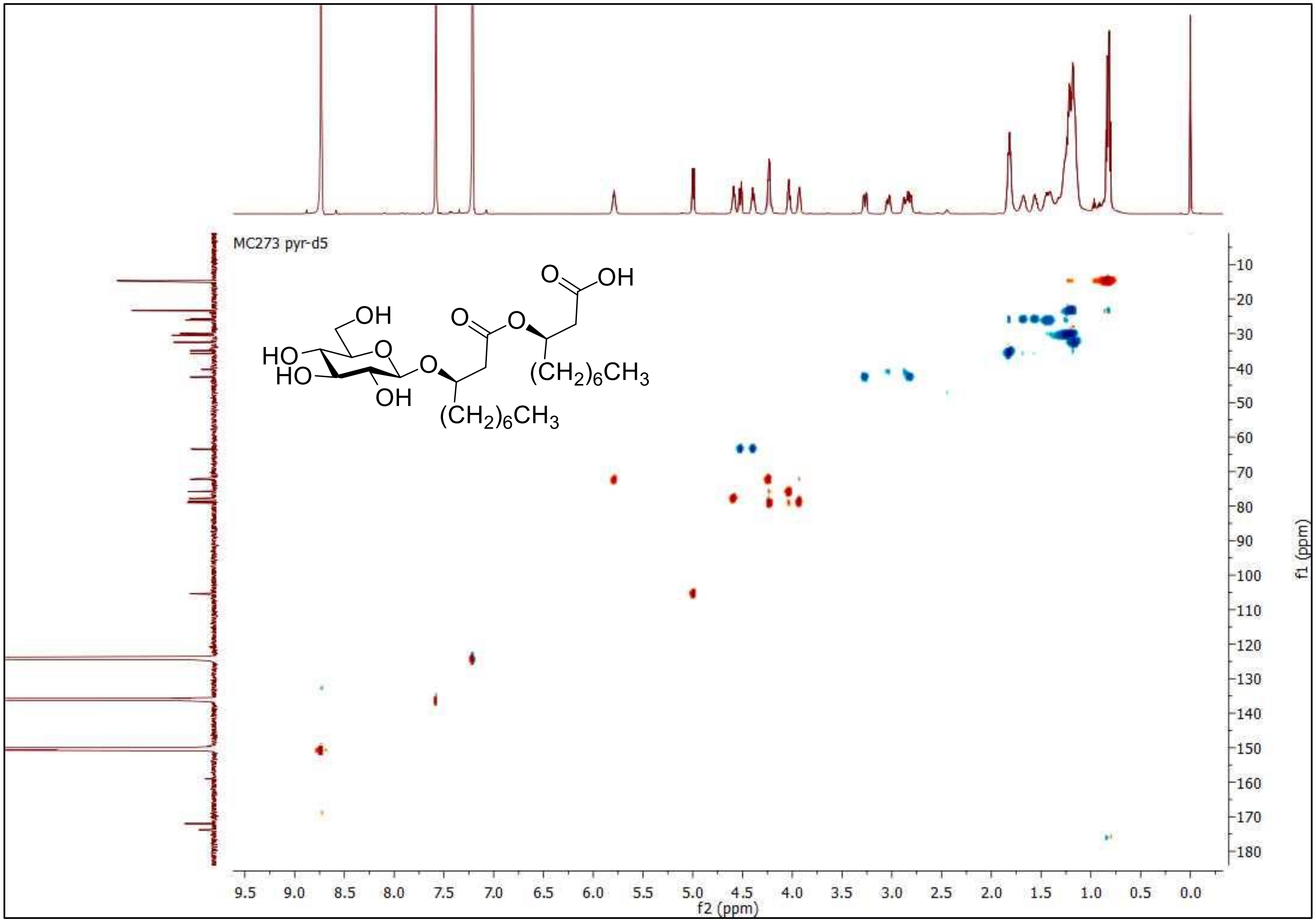

Figure S49 | HMBC NMR spectrum (pyr-d_5, 600 MHz) of synthetic ananatoside B (**2**).

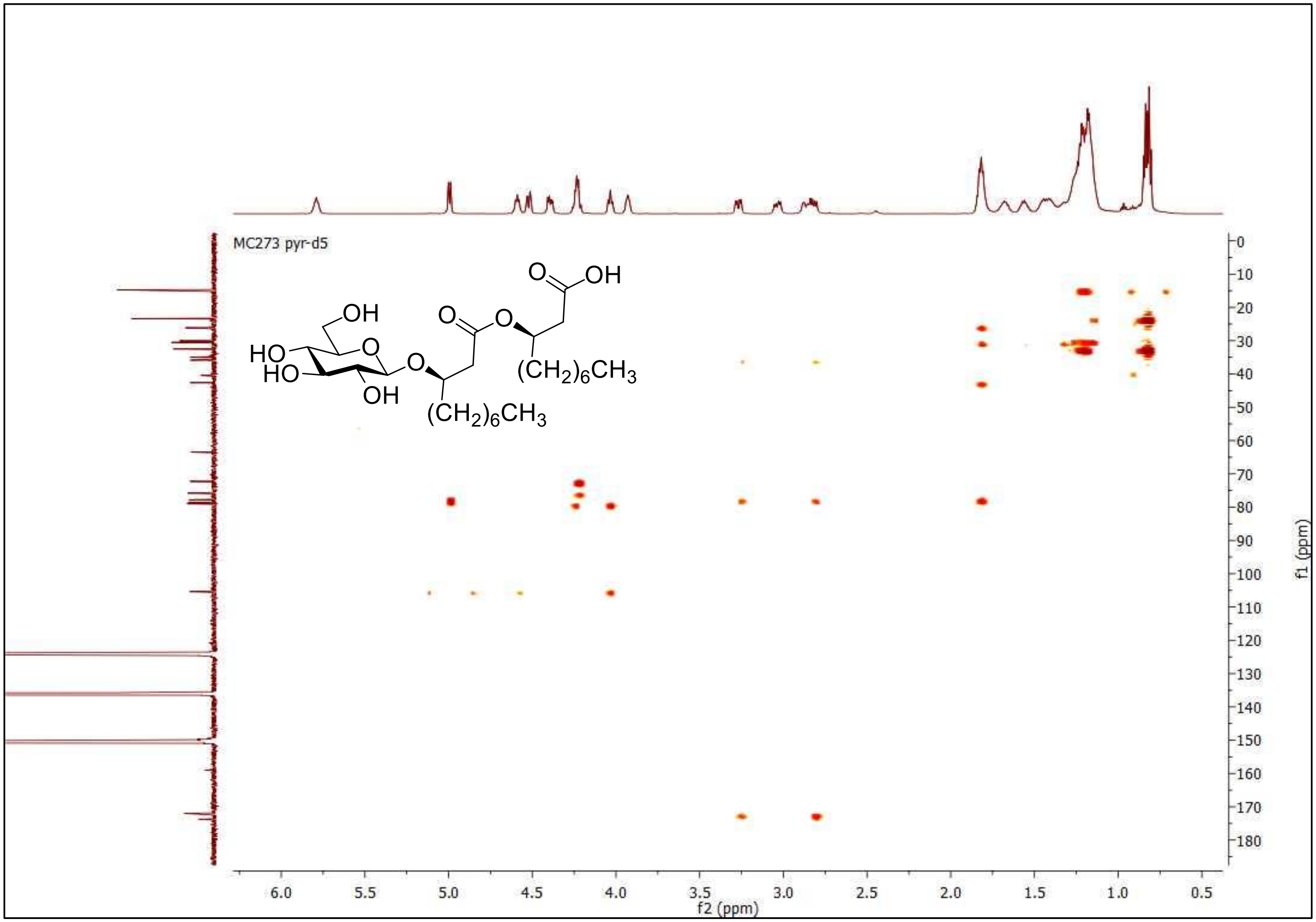

Figure S50 | ^{1}H NMR spectrum (pyr-d_5, 600 MHz) of natural ananatoside B (**2**).

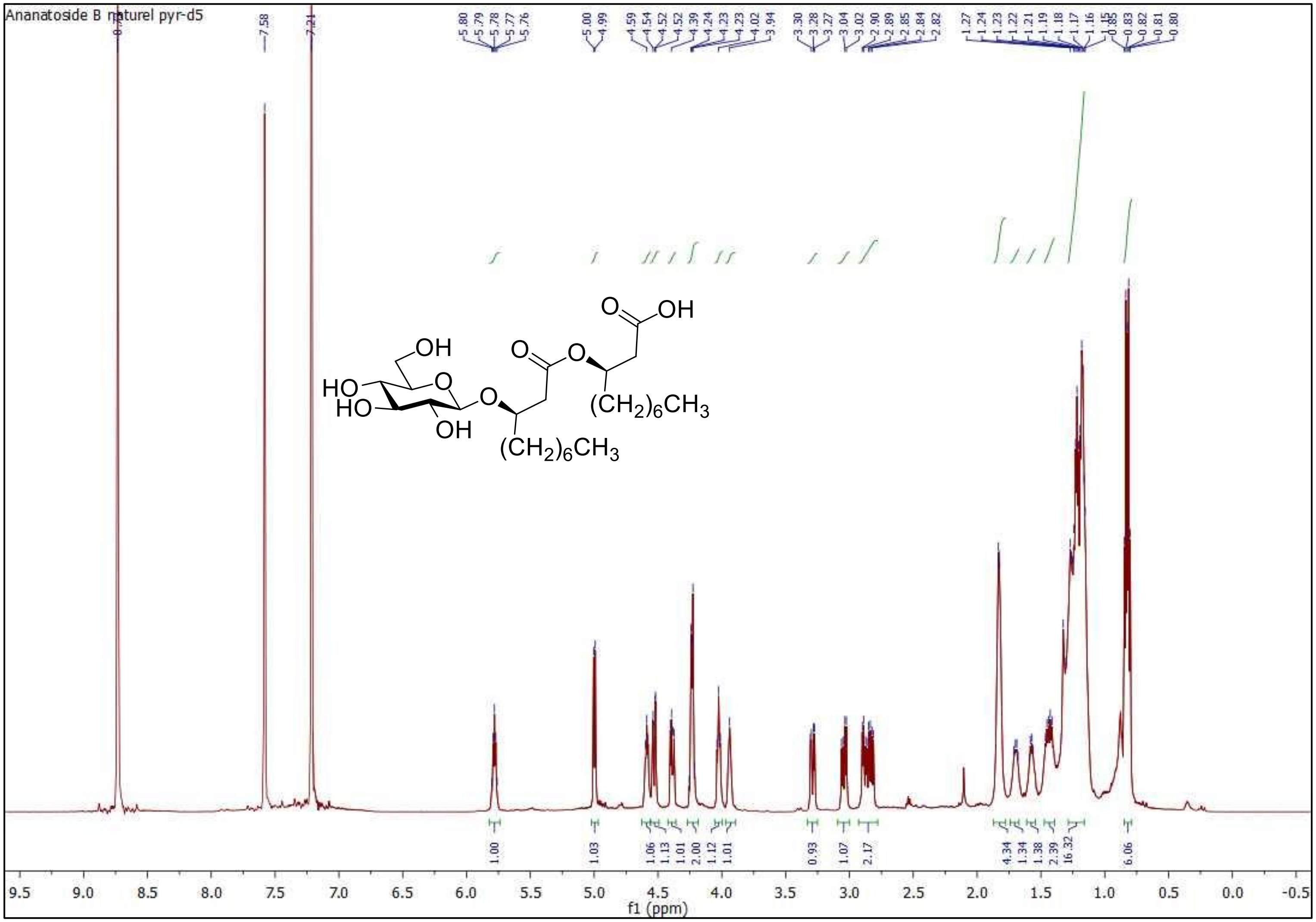

Figure S51 | ^{1}H NMR spectrum ($CDCl_3$, 600 MHz) of *para*-methylphenyl 2-*O*-*ortho*-(azidomethyl)benzoyl-3,4-di-*O*-benzyl-6-*O*-*tert*-butyldimethylsilyl-1-thio-*β*-D-glucopyranoside (**S11**).

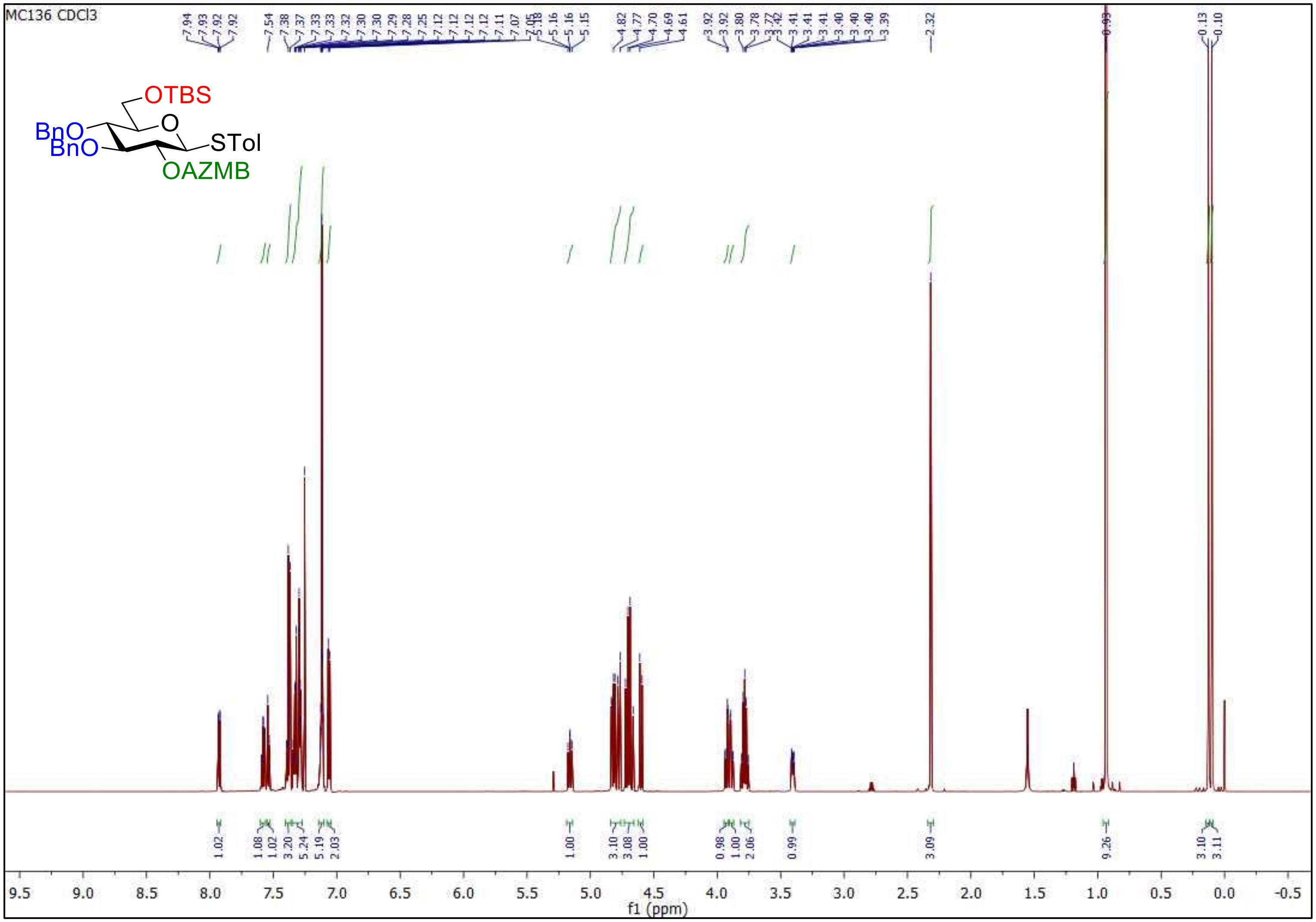

Figure S52 | COSY NMR spectrum ($CDCl_3$, 600 MHz) of *para*-methylphenyl 2-*O*-*ortho*-(azidomethyl)benzoyl-3,4-di-*O*-benzyl-6-*O*-*tert*-butyldimethylsilyl-1-thio-*β*-D-glucopyranoside (**S11**).

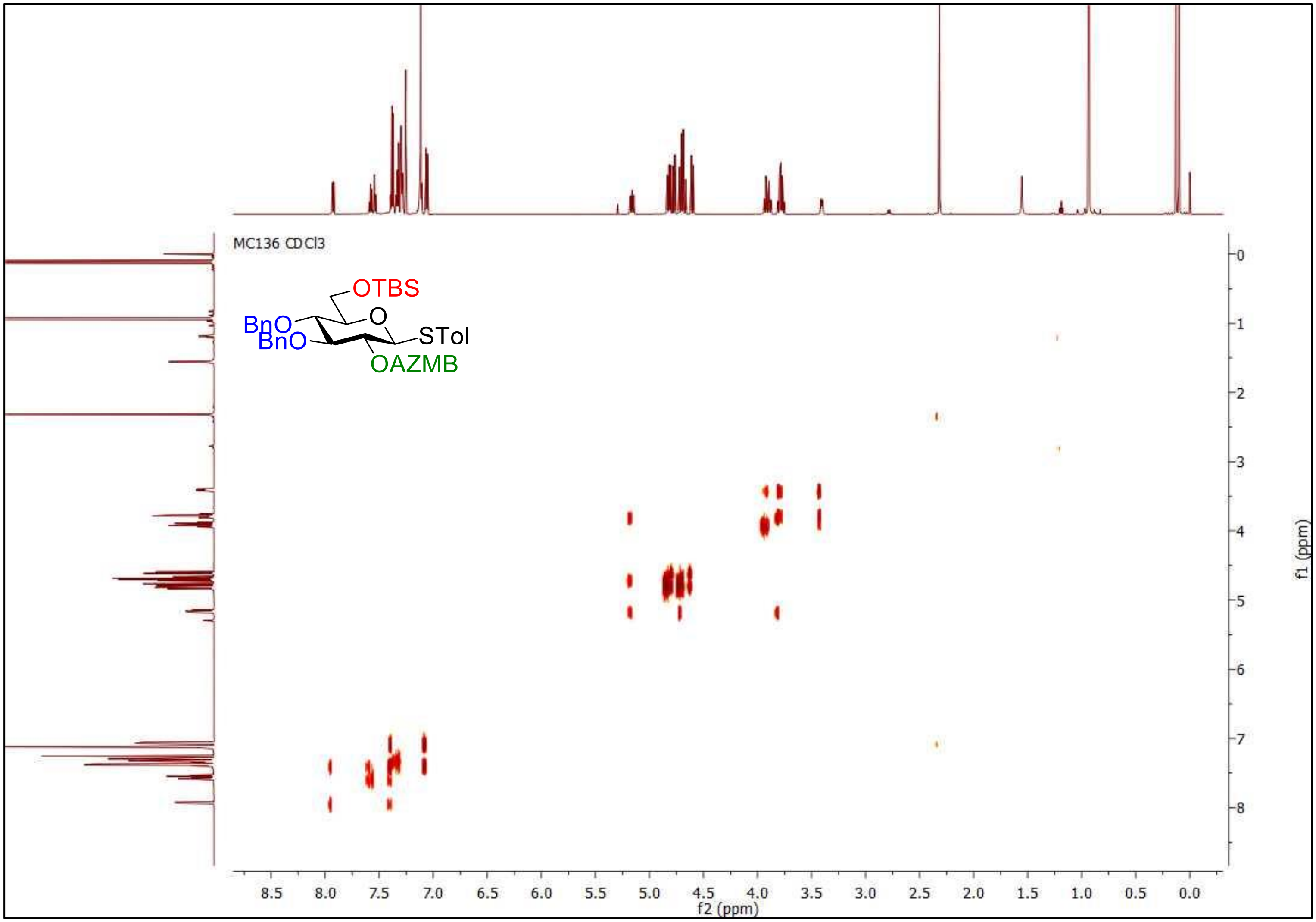

Figure S53 | ^{13}C NMR spectrum ($CDCl_3$, 600 MHz) of *para*-methylphenyl 2-*O*-*ortho*-(azidomethyl)benzoyl-3,4-di-*O*-benzyl-6-*O*-*tert*-butyldimethylsilyl-1-thio-*β*-D-glucopyranoside (**S11**).

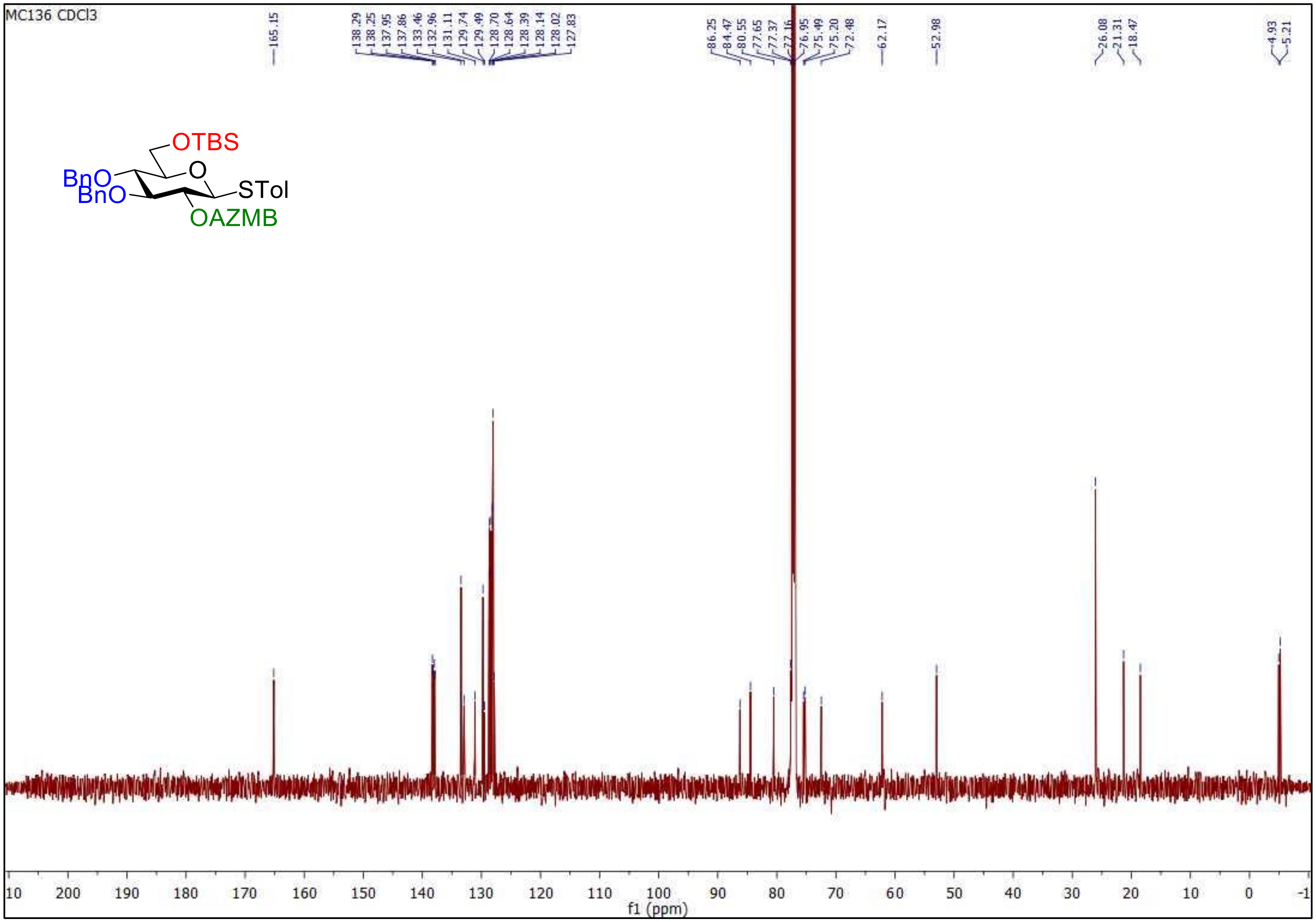

Figure S54 | HSQC NMR spectrum ($CDCl_3$, 600 MHz) of *para*-methylphenyl 2-*O*-*ortho*-(azidomethyl)benzoyl-3,4-di-*O*-benzyl-6-*O*-*tert*-butyldimethylsilyl-1-thio-*β*-D-glucopyranoside (**S11**).

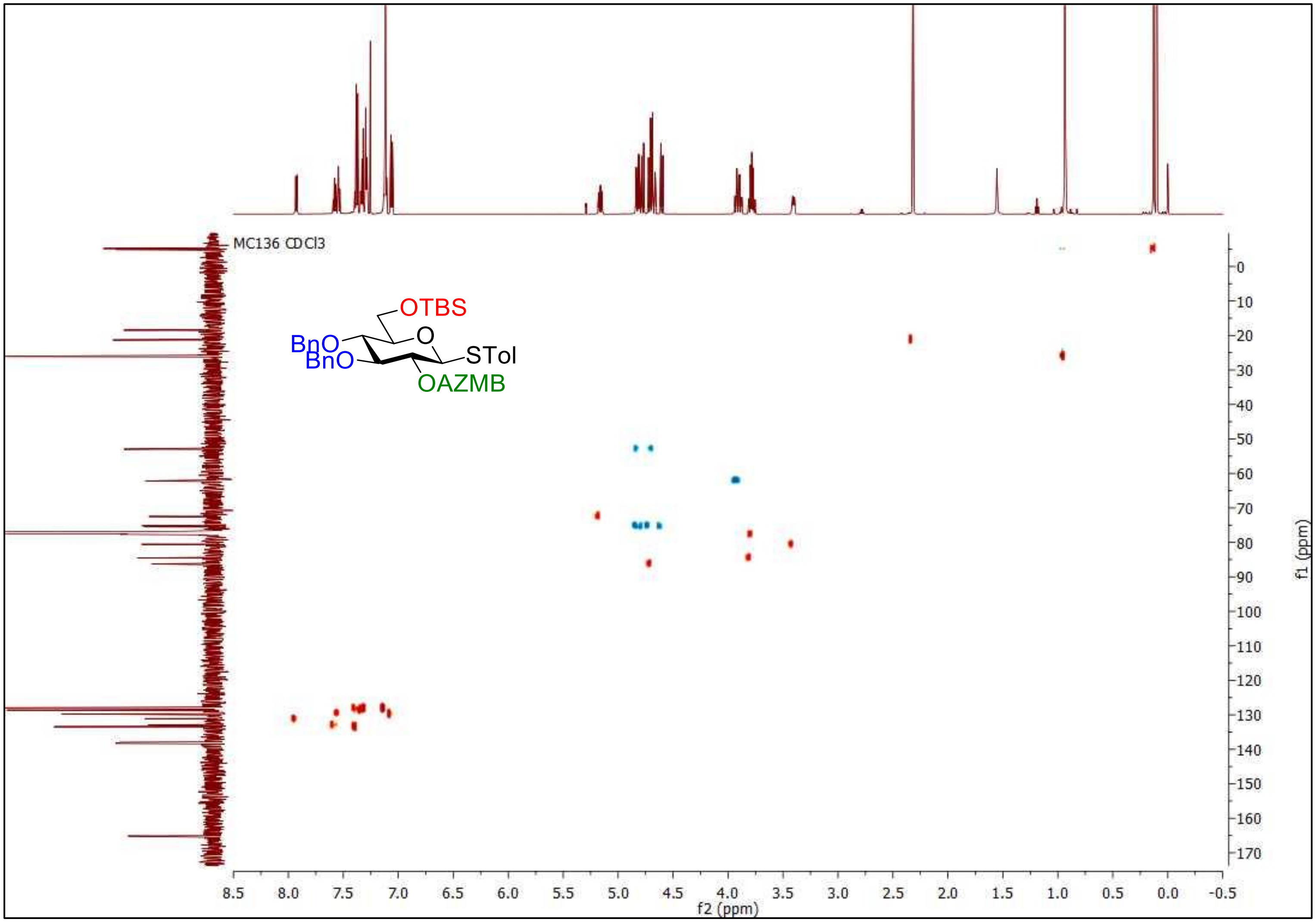

Figure S55 | ^{1}H NMR spectrum ($CDCl_3$, 600 MHz) of *para*-methylphenyl 2-*O*-*ortho*-(azidomethyl)benzoyl-3,4-di-*O*-benzyl-1-thio-*β*-D-glucopyranoside (**9**).

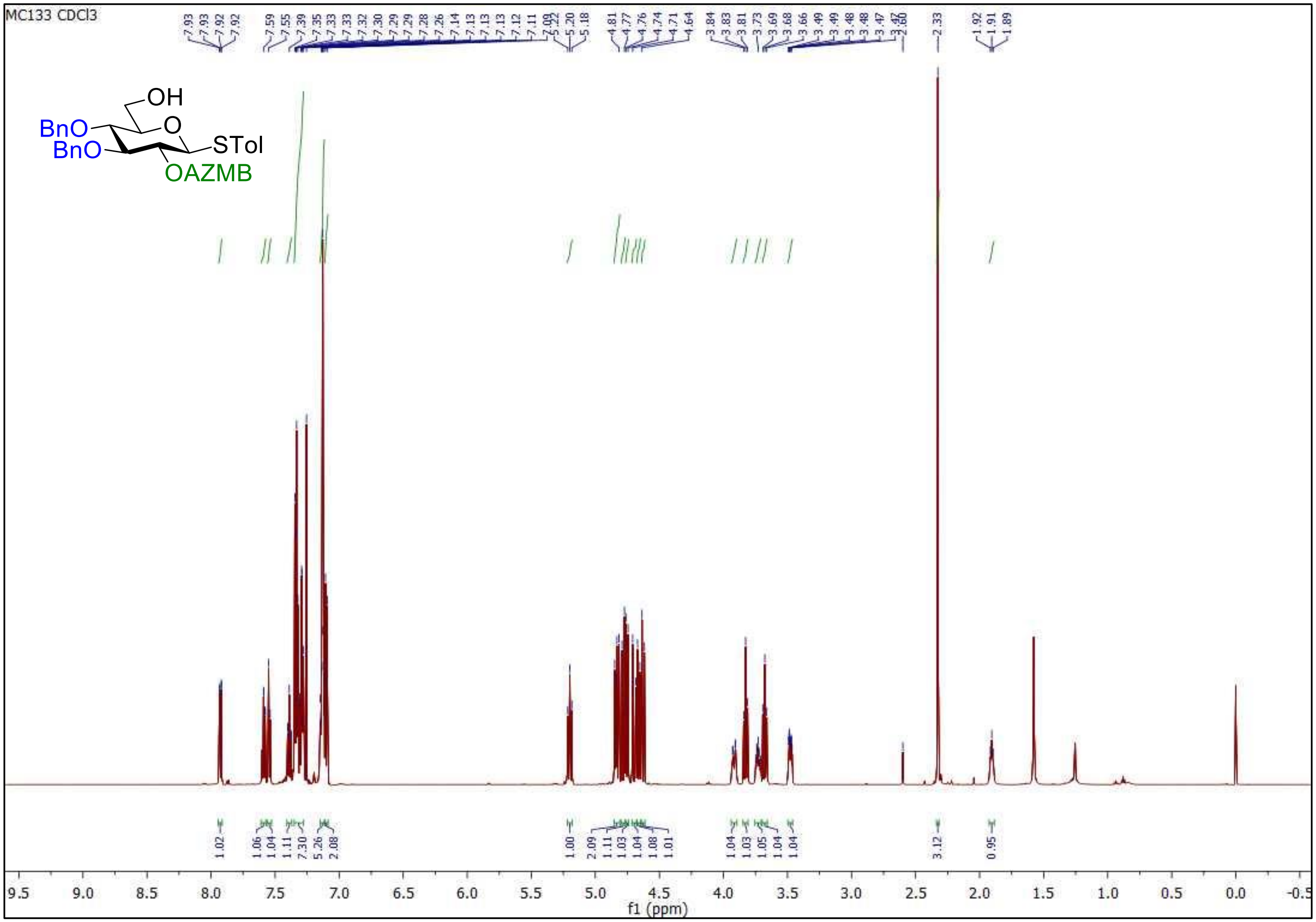

Figure S56 | COSY NMR spectrum ($CDCl_3$, 600 MHz) of *para*-methylphenyl 2-*O*-*ortho*-(azidomethyl)benzoyl-3,4-di-*O*-benzyl-1-thio-*β*-D-glucopyranoside (**9**).

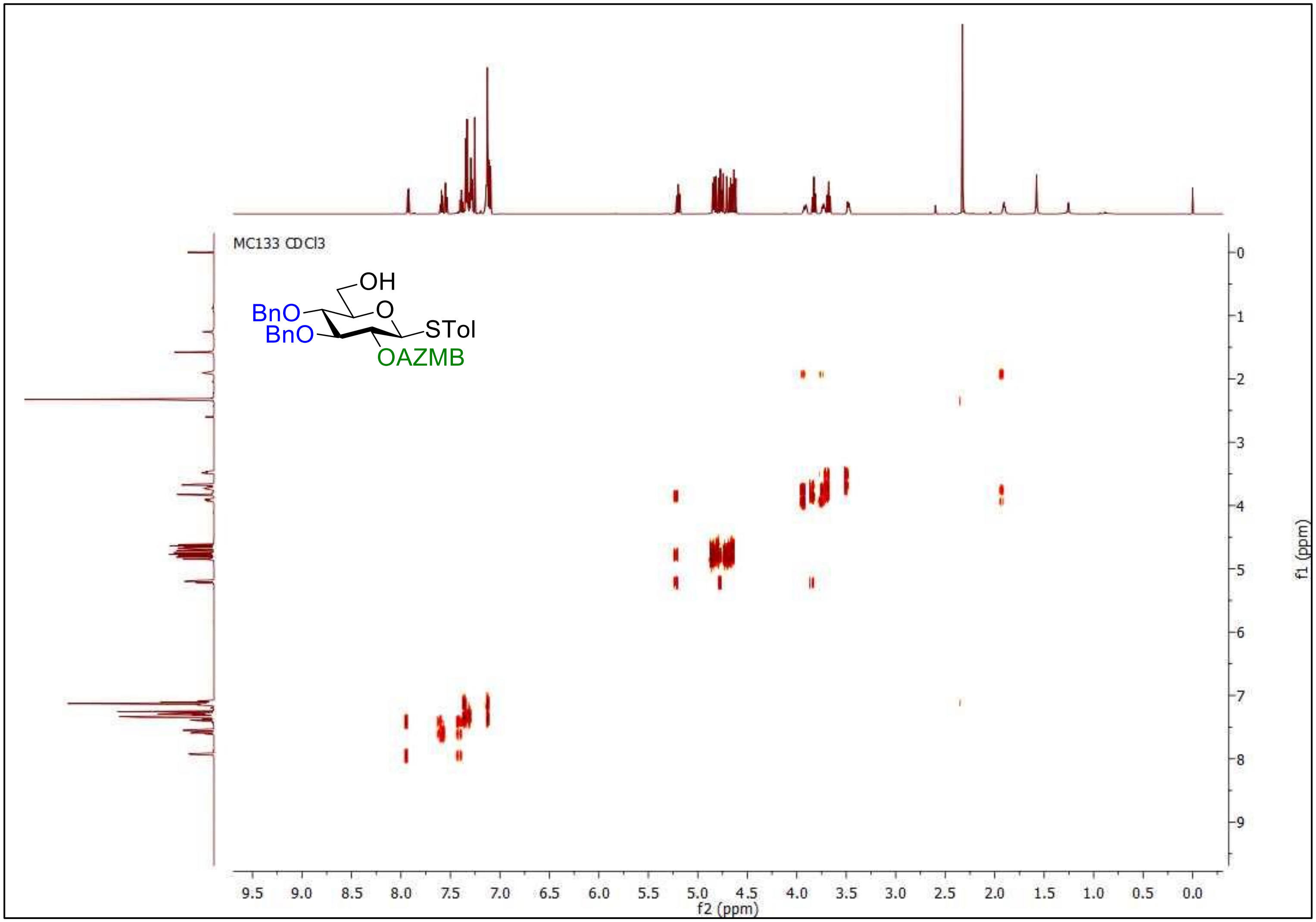

Figure S57 | ^{13}C NMR spectrum (CDCl$_3$, 600 MHz) of *para*-methylphenyl 2-*O*-*ortho*-(azidomethyl)benzoyl-3,4-di-*O*-benzyl-1-thio-*β*-D-glucopyranoside (**9**).

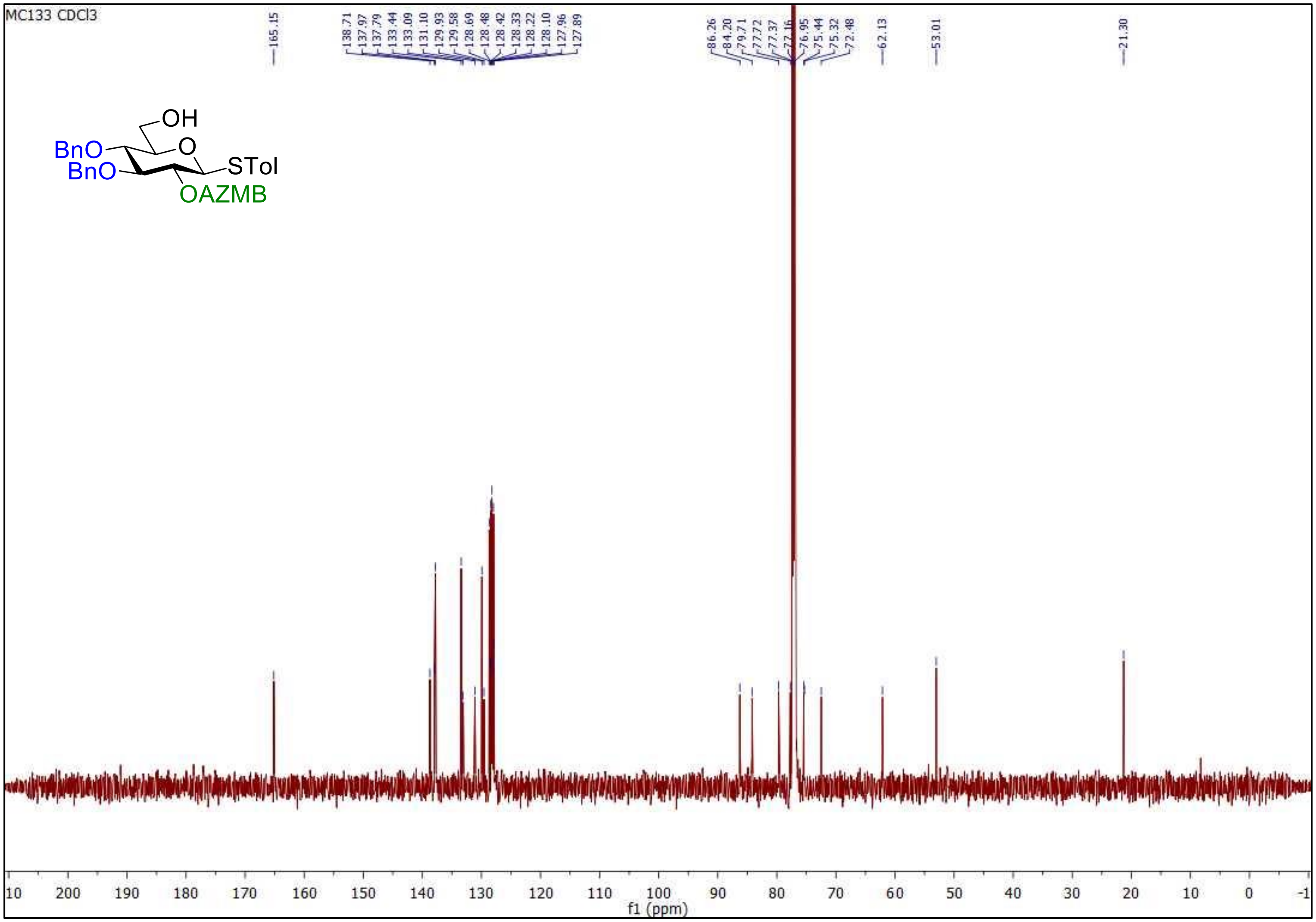

Figure S58 | HSQC NMR spectrum ($CDCl_3$, 600 MHz) of *para*-methylphenyl 2-*O*-*ortho*-(azidomethyl)benzoyl-3,4-di-*O*-benzyl-1-thio-*β*-D-glucopyranoside (**9**).

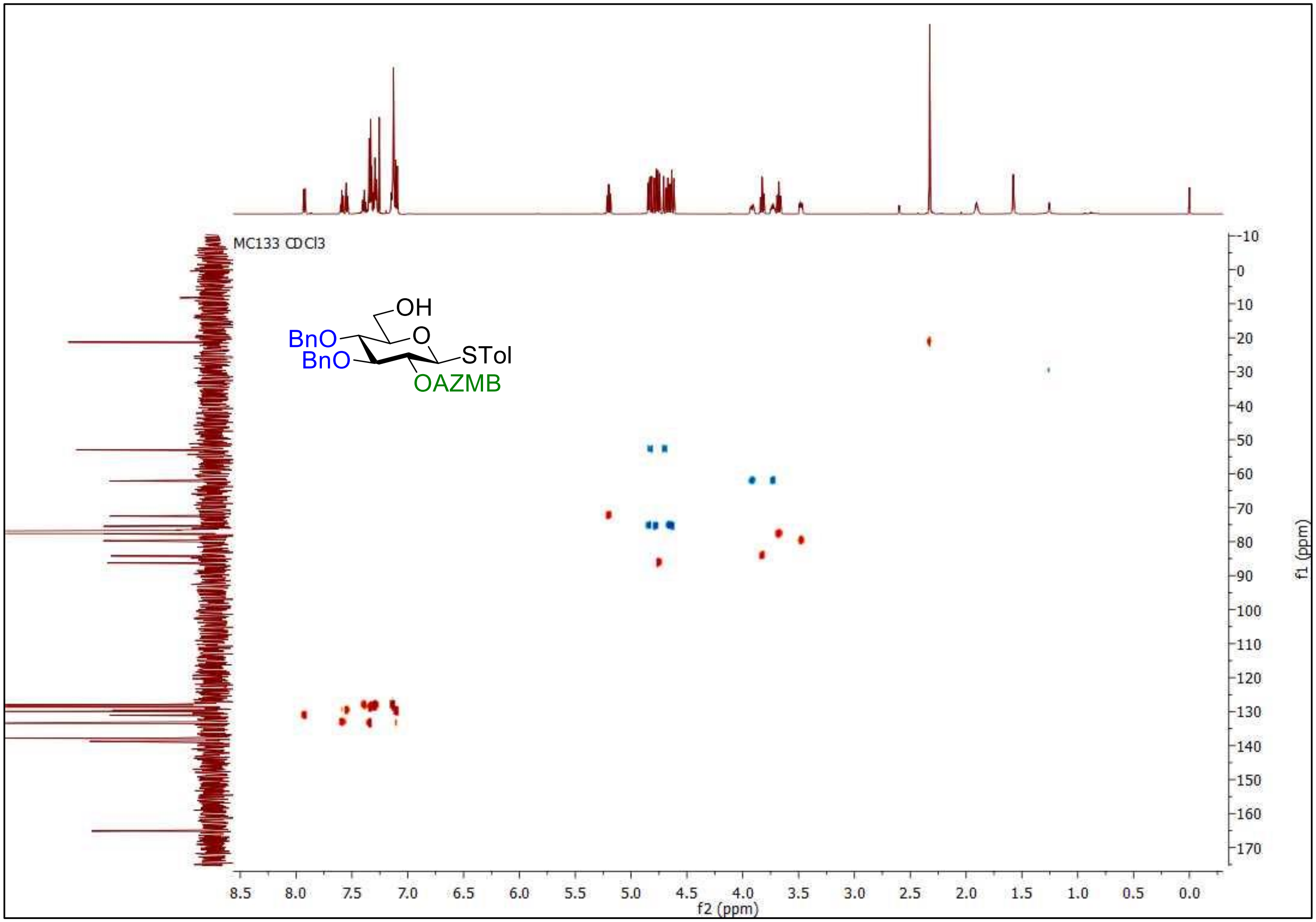

Figure S59 | ^{1}H NMR spectrum ($CDCl_3$, 600 MHz) of *para*-methylphenyl 2-*O*-*ortho*-(azidomethyl)benzoyl-3,4-di-*O*-benzyl-6-*O*-(*R*)-3-((*R*)-3-(((*tert*-butyldimethylsilyl)oxy)decanoyl)oxy)decanoyl-1-thio-*β*-D-glucopyranoside (**7**).

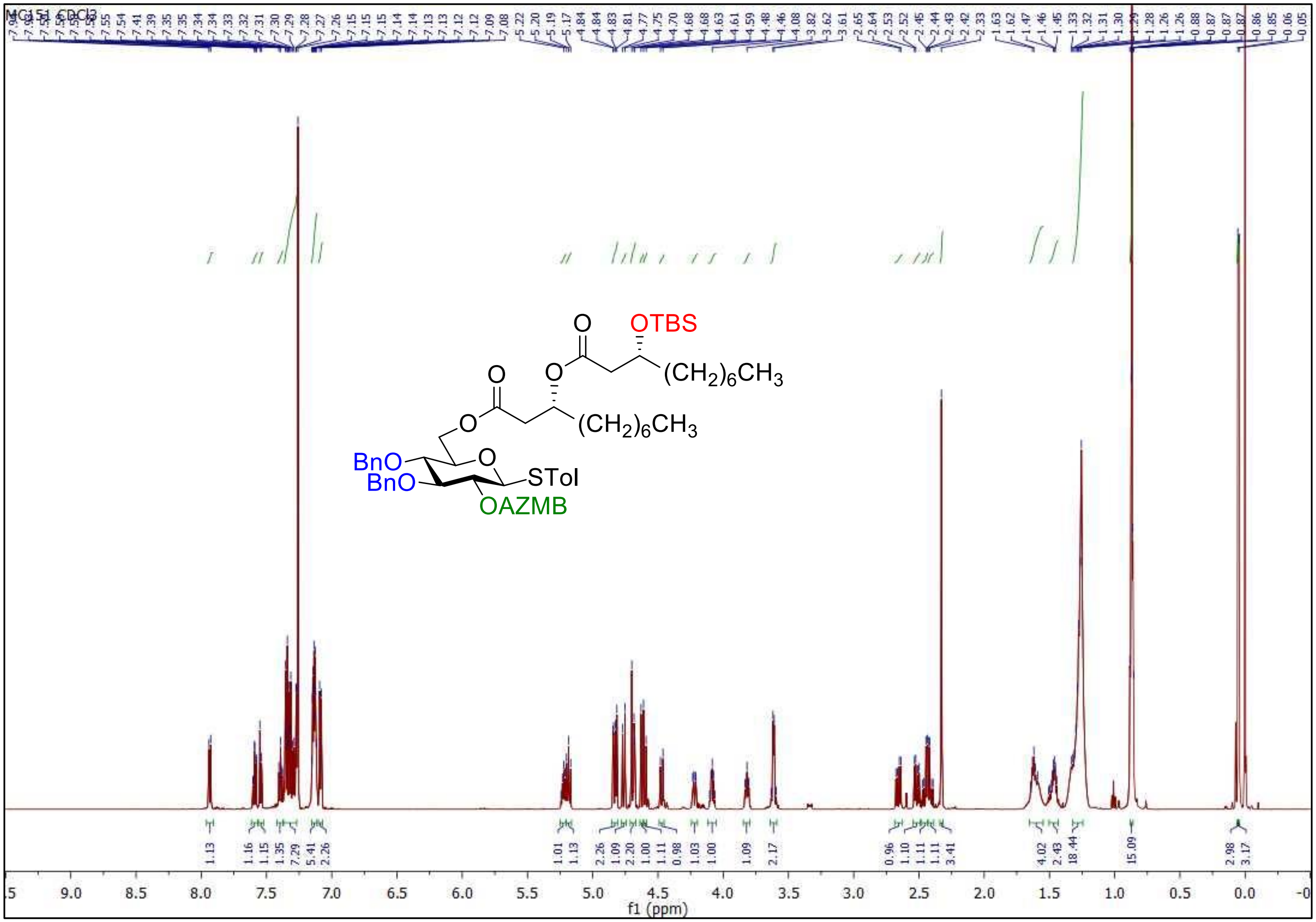

Figure S60 | COSY NMR spectrum (CDCl3, 600 MHz) of *para*-methylphenyl 2-*O*-*ortho*-(azidomethyl)benzoyl-3,4-di-*O*-benzyl-6-*O*-(*R*)-3-((*R*)-3-(((*tert*-butyldimethylsilyl)oxy)decanoyl)oxy)decanoyl-1-thio-*β*-D-glucopyranoside (**7**).

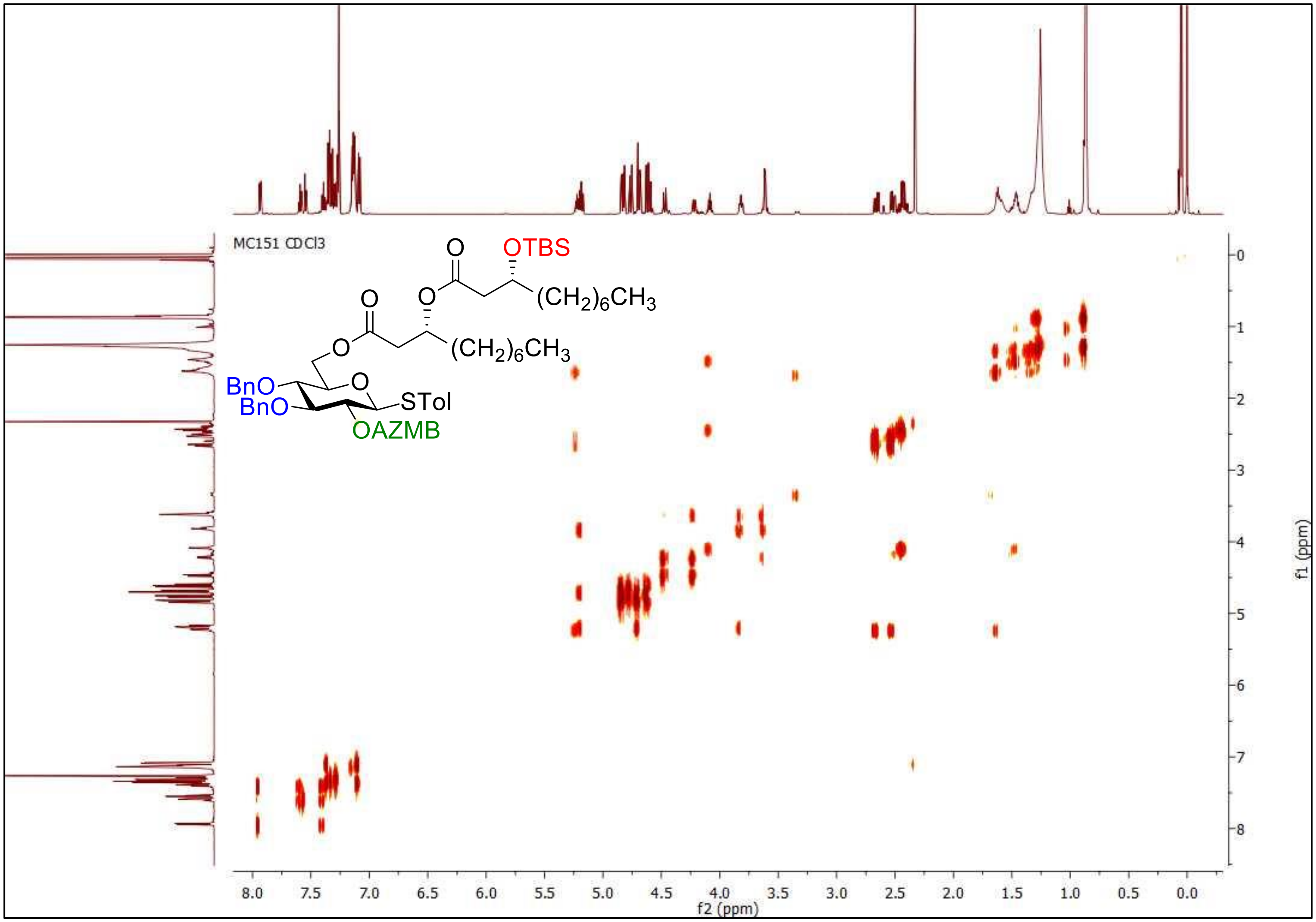

Figure S61 | ^{13}C NMR spectrum ($CDCl_3$, 600 MHz) of *para*-methylphenyl 2-*O*-*ortho*-(azidomethyl)benzoyl-3,4-di-*O*-benzyl-6-*O*-(*R*)-3-((*R*)-3-(((*tert*-butyldimethylsilyl)oxy)decanoyl)oxy)decanoyl-1-thio-*β*-D-glucopyranoside (**7**).

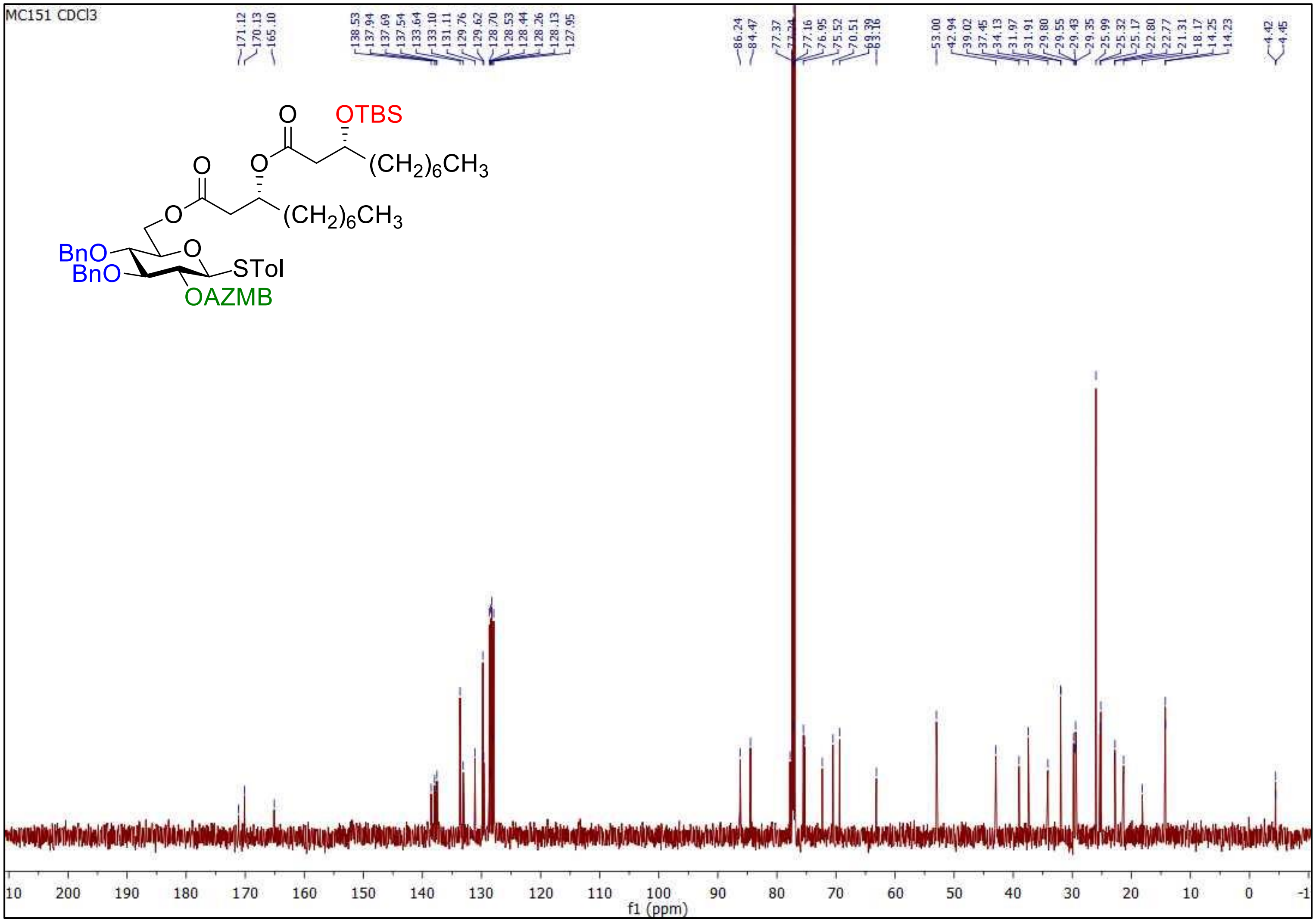

Figure S62 | HSQC NMR spectrum ($CDCl_3$, 600 MHz) of *para*-methylphenyl 2-*O*-*ortho*-(azidomethyl)benzoyl-3,4-di-*O*-benzyl-6-*O*-(*R*)-3-((*R*)-3-(((*tert*-butyldimethylsilyl)oxy)decanoyl)oxy)decanoyl-1-thio-*β*-D-glucopyranoside (**7**).

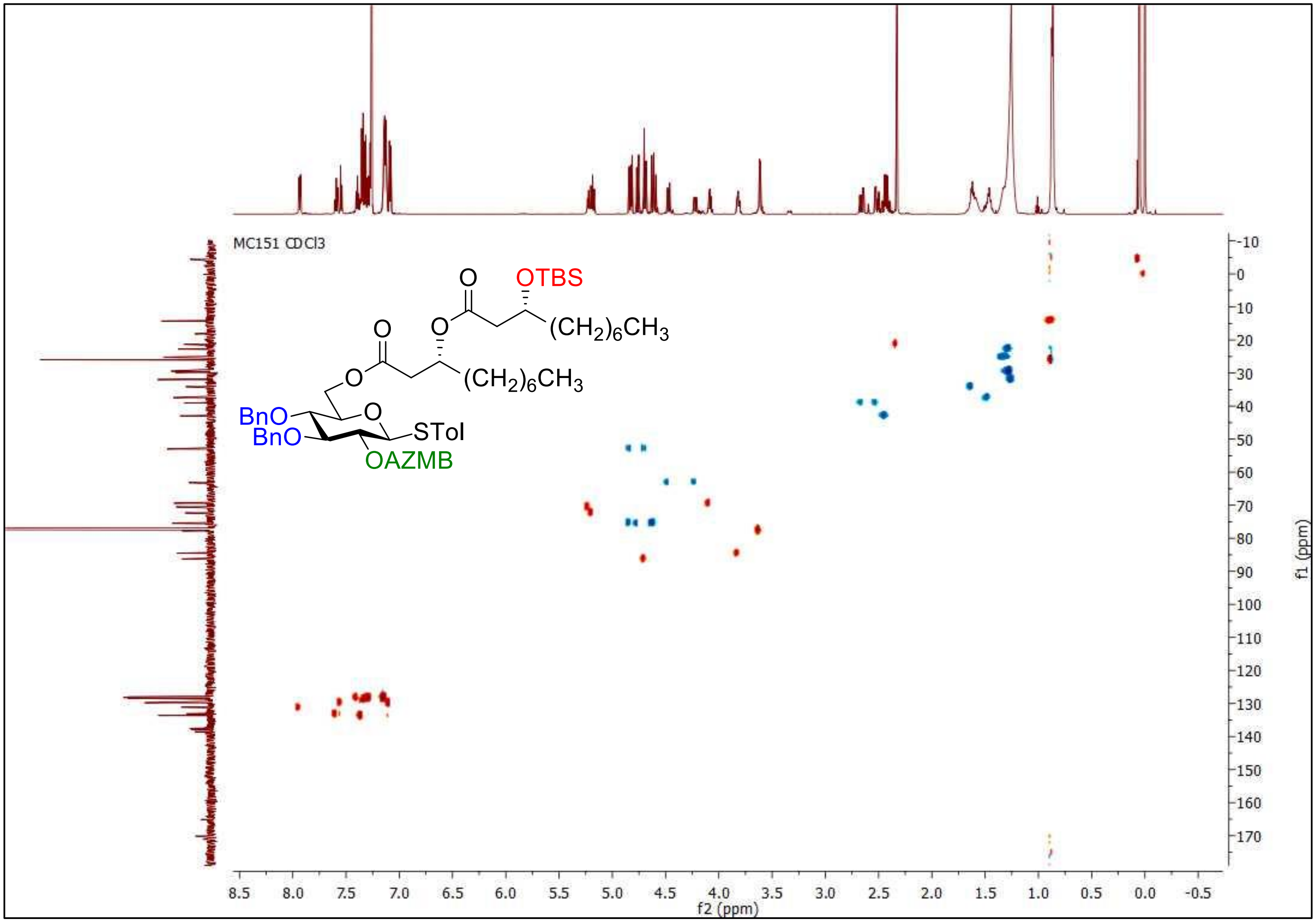

Figure S63 | ^{1}H NMR spectrum ($CDCl_3$, 600 MHz) of *para*-methylphenyl 2-*O*-*ortho*-(azidomethyl)benzoyl-3,4-di-*O*-benzyl-6-*O*-(*R*)-3-((*R*)-3-(hydroxydecanoyl)oxy)decanoyl-1-thio-*β*-D-glucopyranoside (**20**).

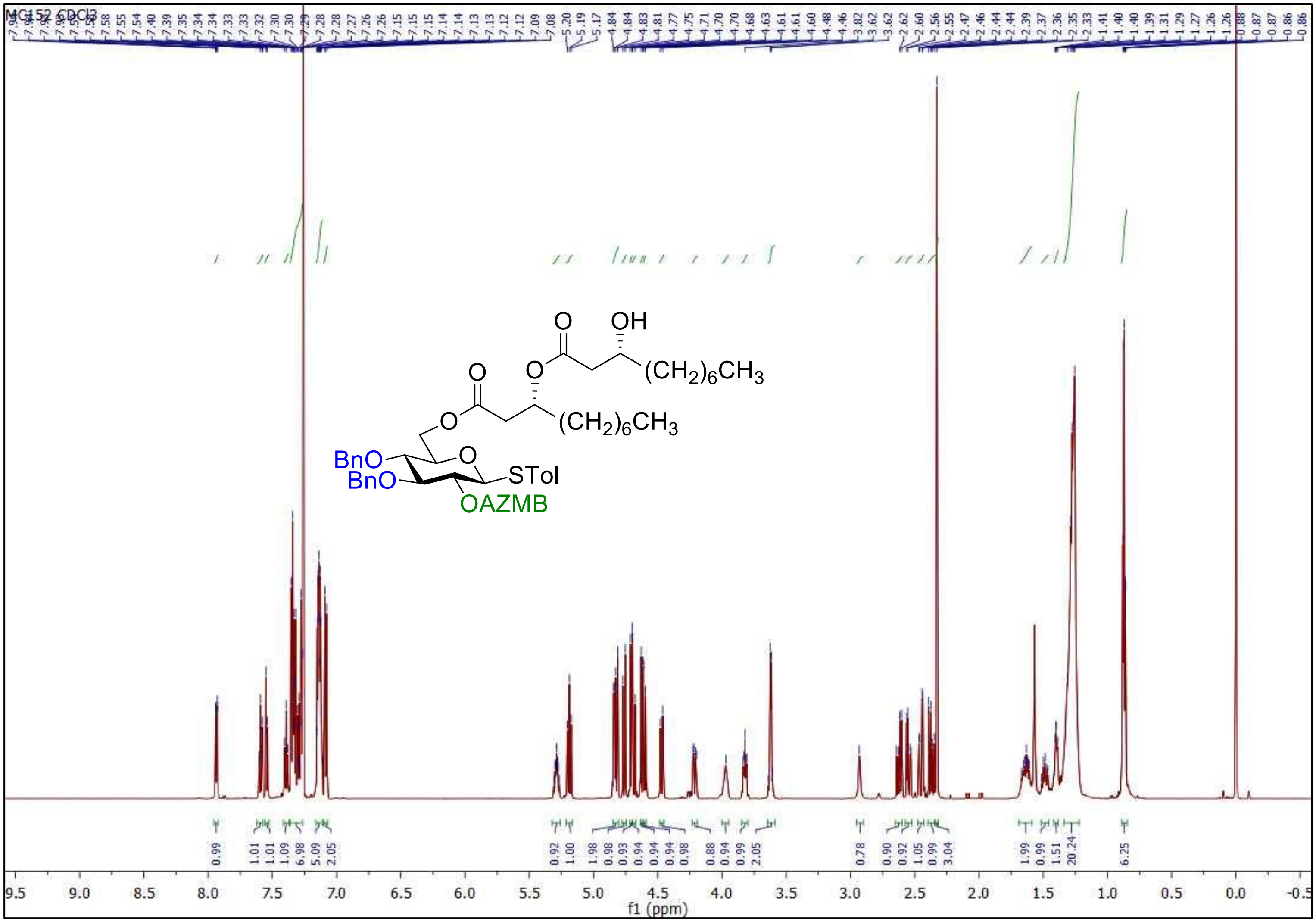

Figure S64 | COSY NMR spectrum (CDCl3, 600 MHz) of *para*-methylphenyl 2-*O*-*ortho*-(azidomethyl)benzoyl-3,4-di-*O*-benzyl-6-*O*-(*R*)-3-((*R*)-3-(hydroxydecanoyl)oxy)decanoyl-1-thio-*β*-D-glucopyranoside (**20**).

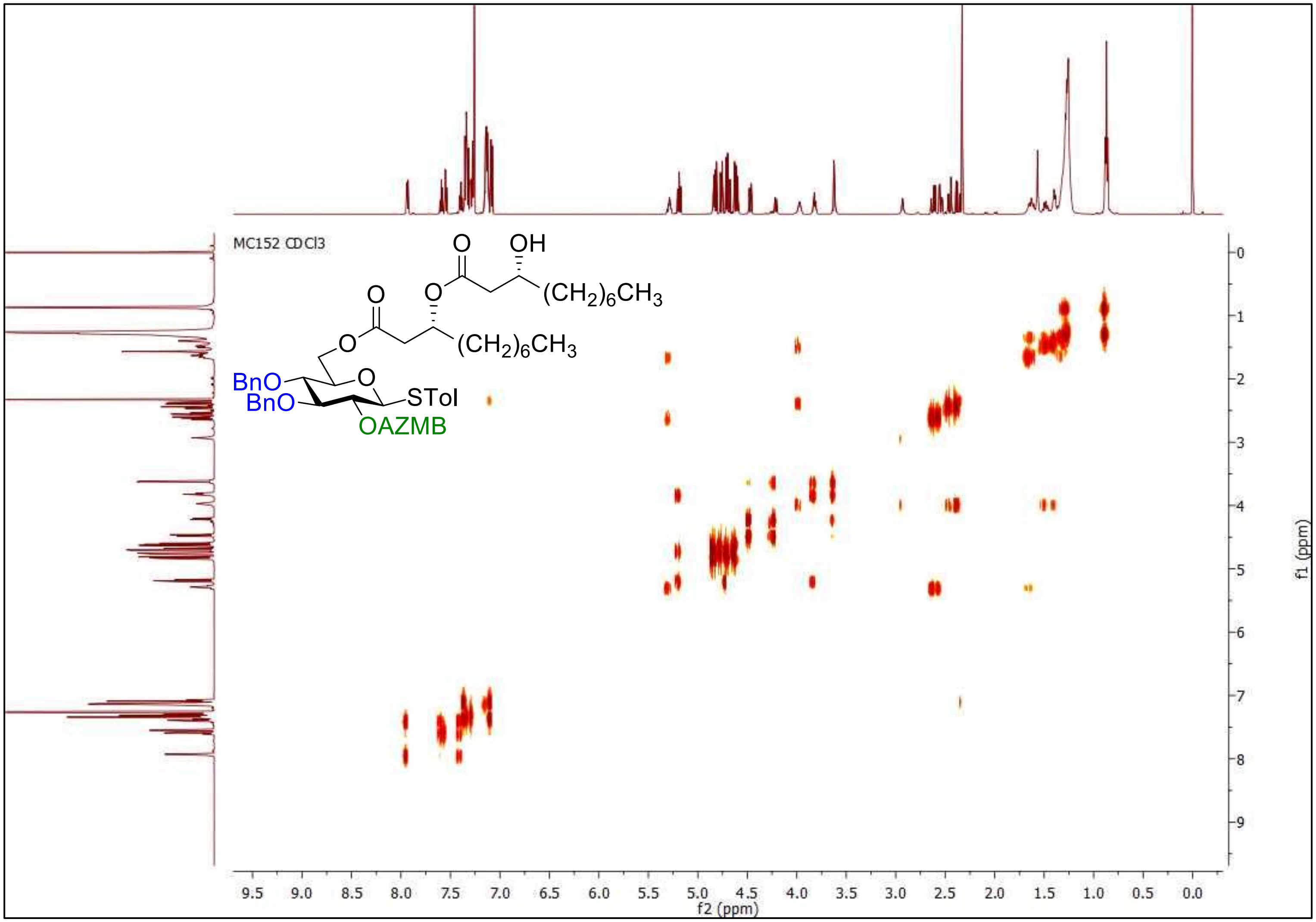

Figure S65 | ^{13}C NMR spectrum ($CDCl_3$, 600 MHz) of *para*-methylphenyl 2-*O*-*ortho*-(azidomethyl)benzoyl-3,4-di-*O*-benzyl-6-*O*-(*R*)-3-((*R*)-3-(hydroxydecanoyl)oxy)decanoyl-1-thio-*β*-D-glucopyranoside (**20**).

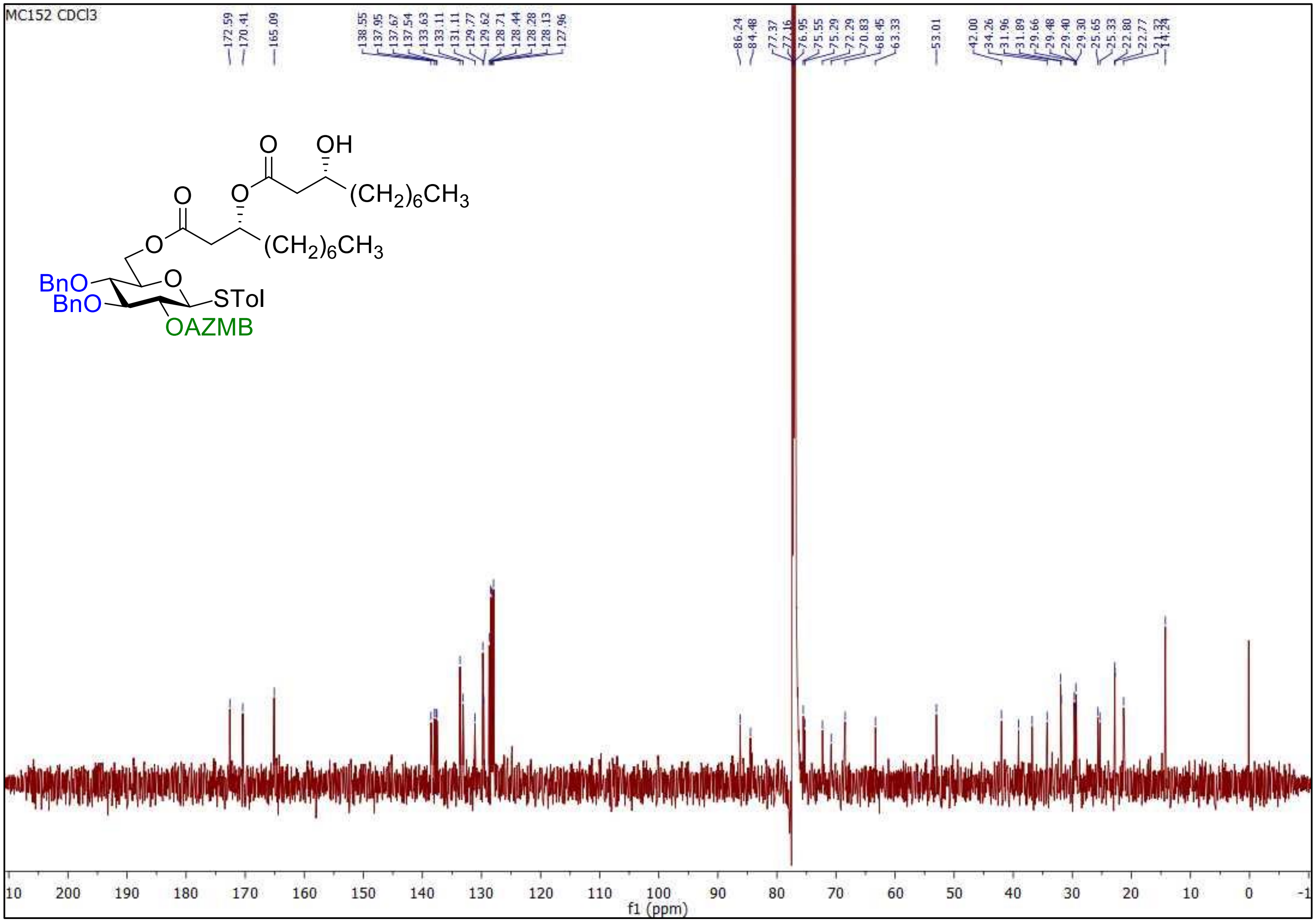

Figure S66 | HSQC NMR spectrum ($CDCl_3$, 600 MHz) of *para*-methylphenyl 2-*O*-*ortho*-(azidomethyl)benzoyl-3,4-di-*O*-benzyl-6-*O*-(*R*)-3-((*R*)-3-(hydroxydecanoyl)oxy)decanoyl-1-thio-*β*-D-glucopyranoside (**20**).

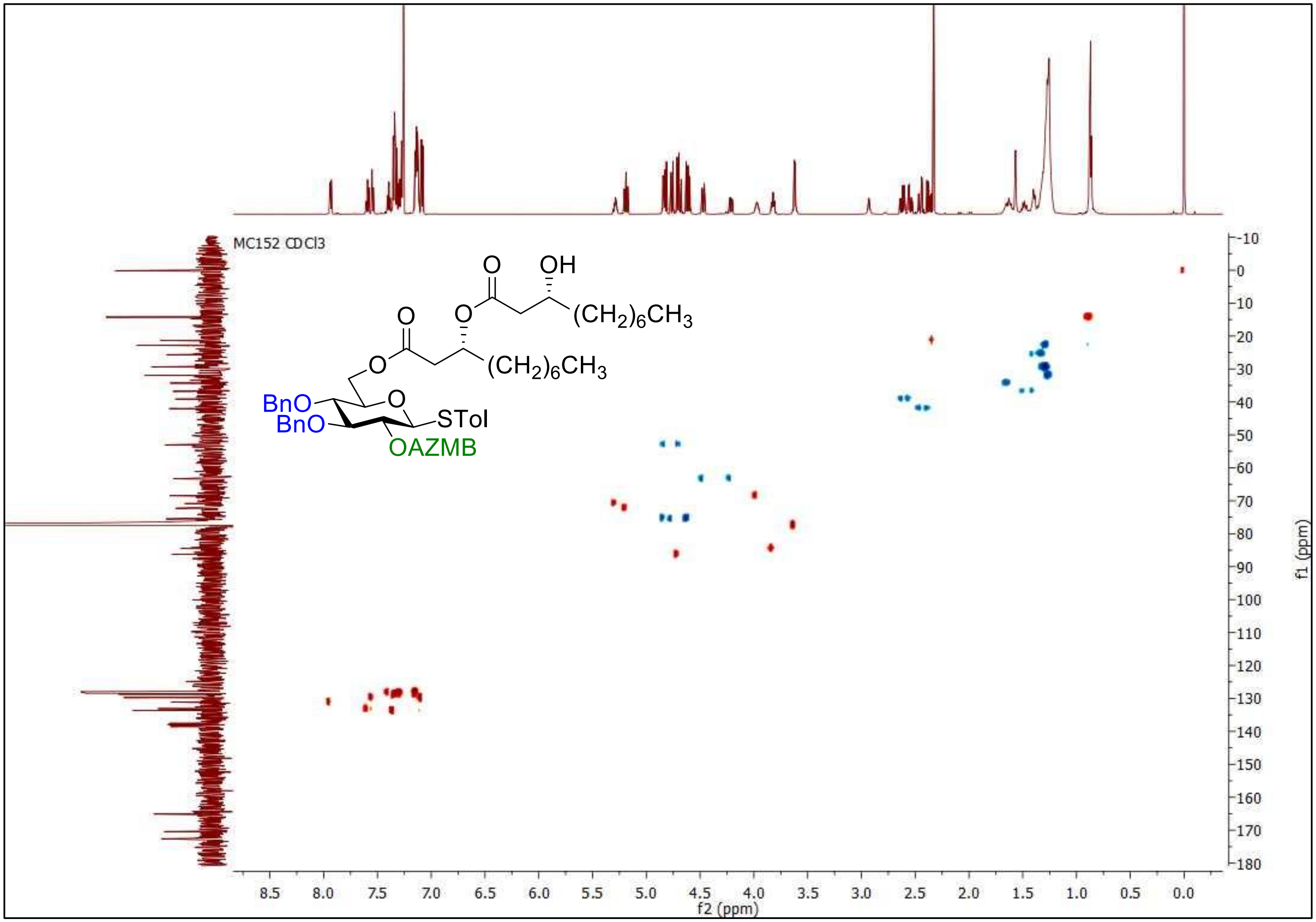

Figure S67 | ^{1}H NMR spectrum ($CDCl_3$, 600 MHz) of macrolide **21**.

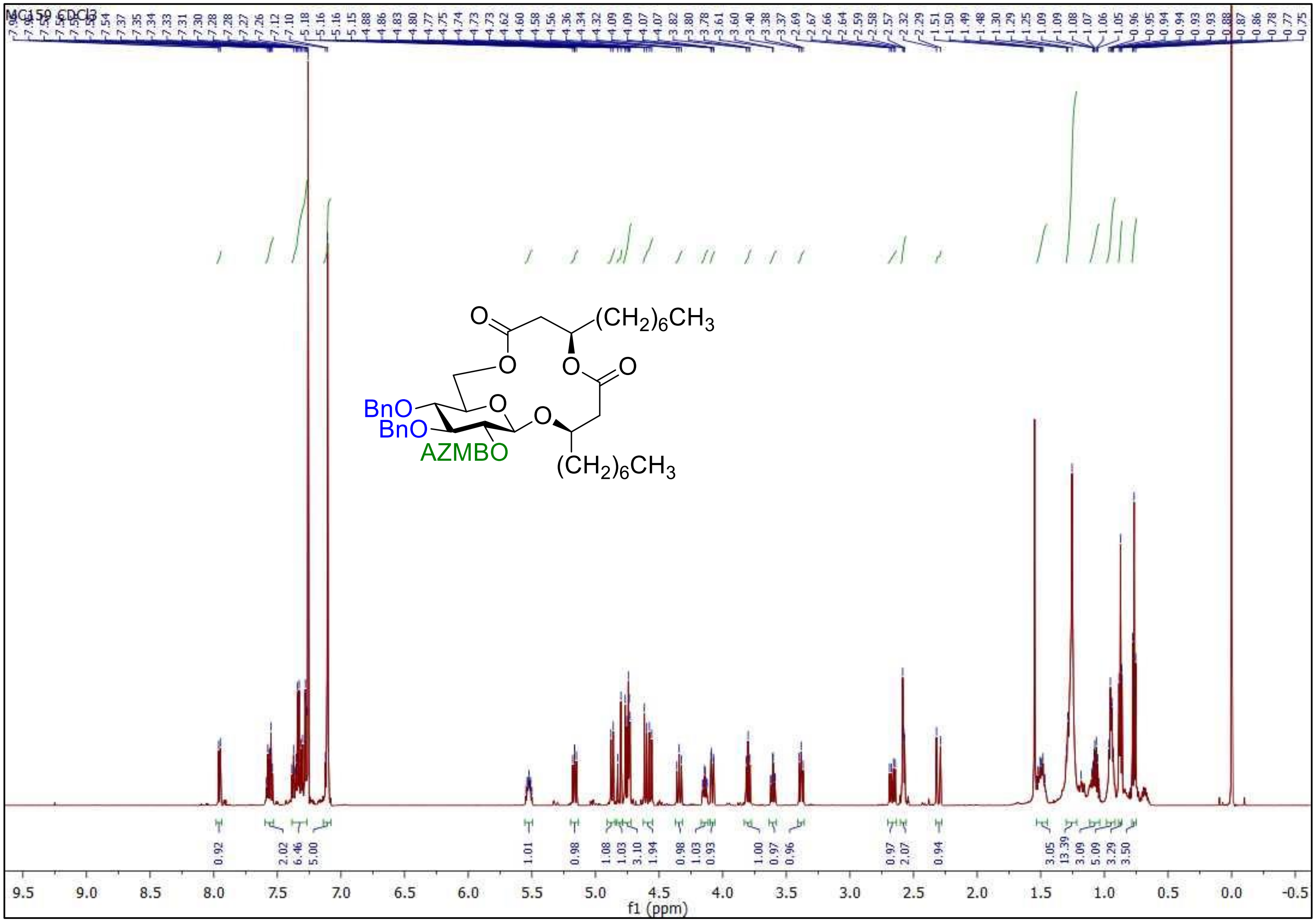

Figure S68 | COSY NMR spectrum (CDCl3, 600 MHz) of macrolide **21**.

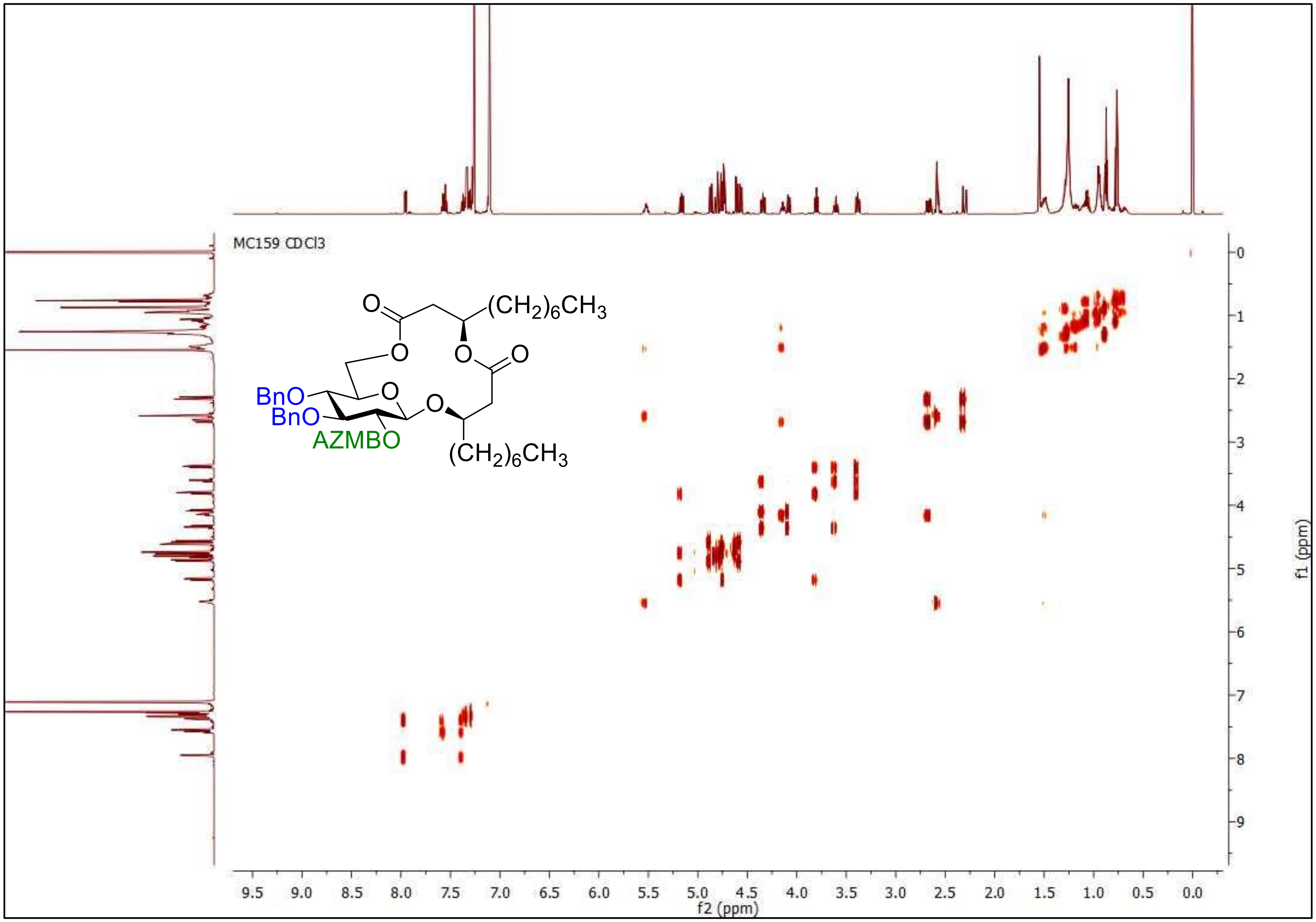

Figure S69 | ^{13}C NMR spectrum ($CDCl_3$, 600 MHz) of macrolide **21**.

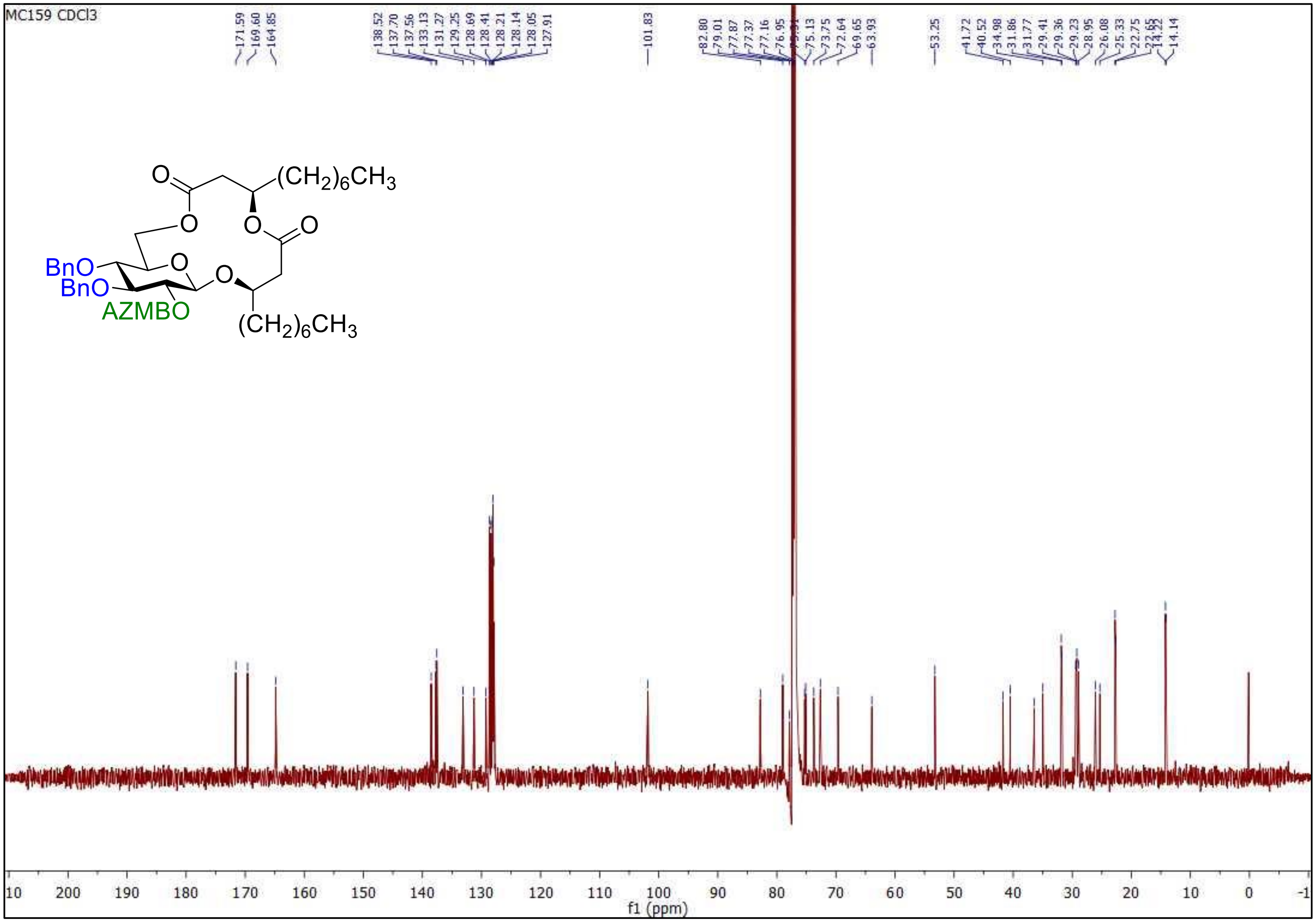

Figure S70 | HSQC NMR spectrum ($CDCl_3$, 600 MHz) of macrolide **21**.

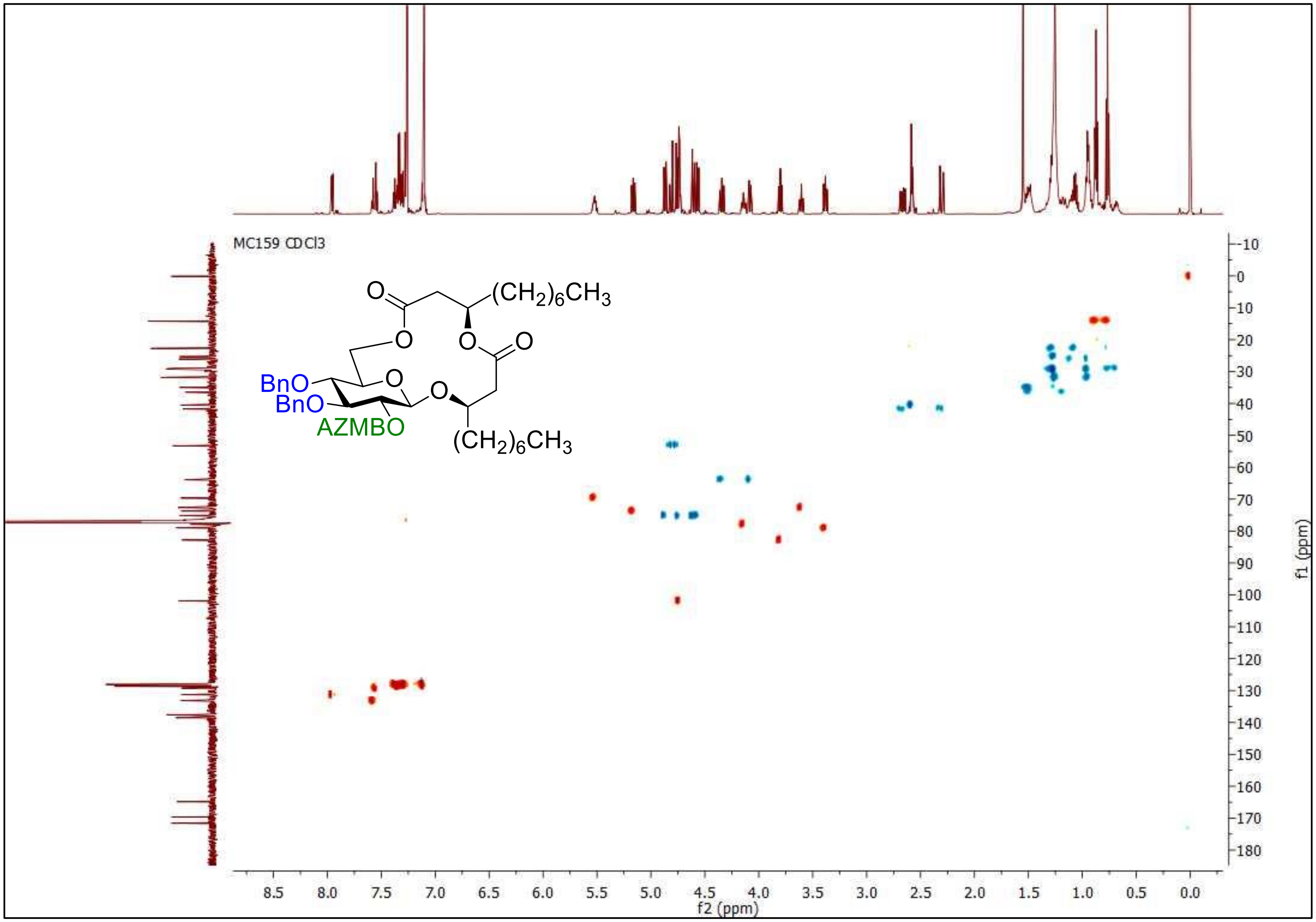

Figure S71 | ^{1}H NMR spectrum ($CDCl_3$, 600 MHz) of (*R*)-3-*O*-[(*R*)-(3'-*O*-decyl)-3,4-di-*O*-benzyl-2-*O*-levulinoyl-*β*-D-glucopyranosyl] decanoic acid (**22**).

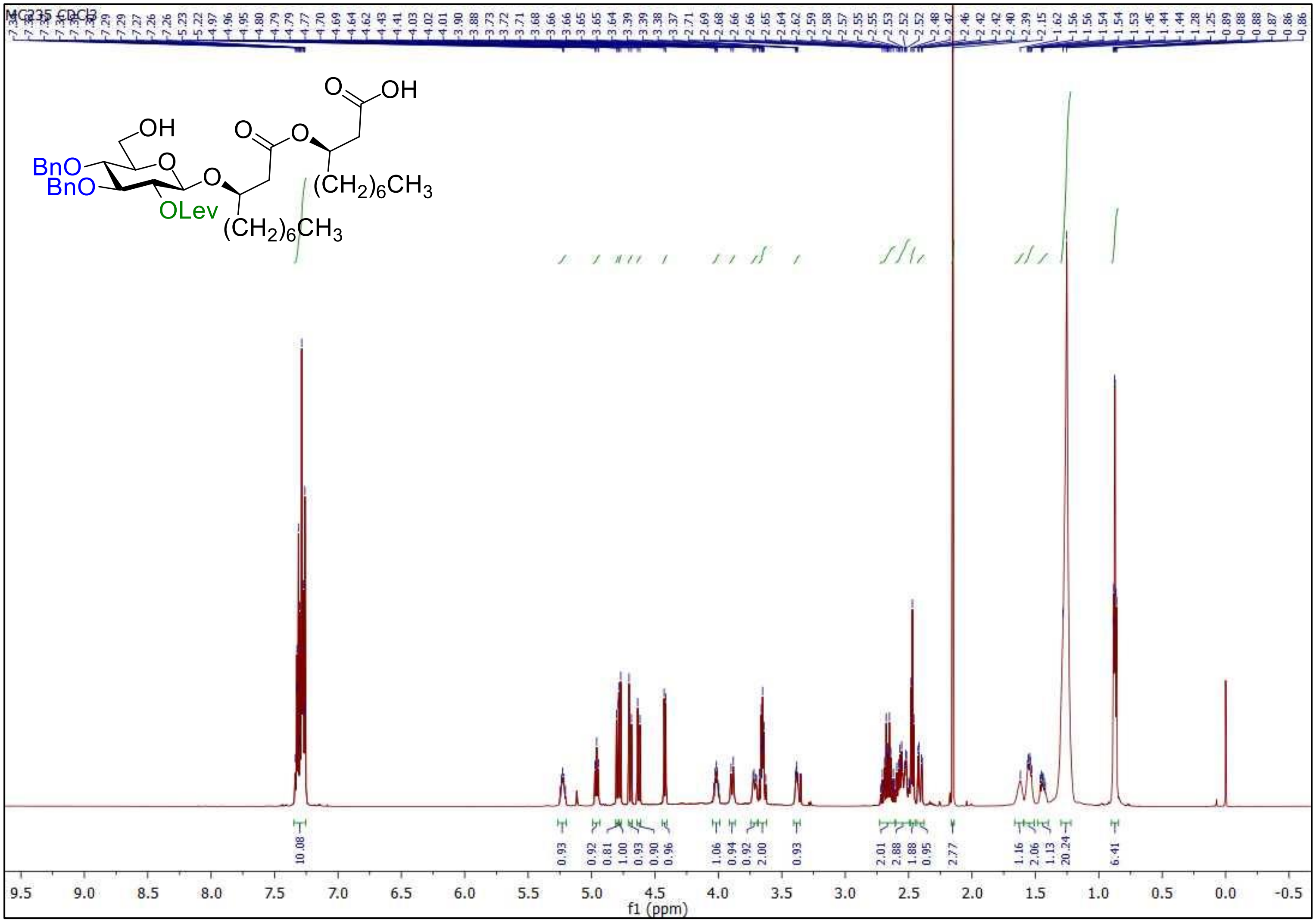

Figure S72 | COSY NMR spectrum ($CDCl_3$, 600 MHz) of (*R*)-3-*O*-[(*R*)-(3'-*O*-decyl)-3,4-di-*O*-benzyl-2-*O*-levulinoyl-*β*-D-glucopyranosyl] decanoic acid (**22**).

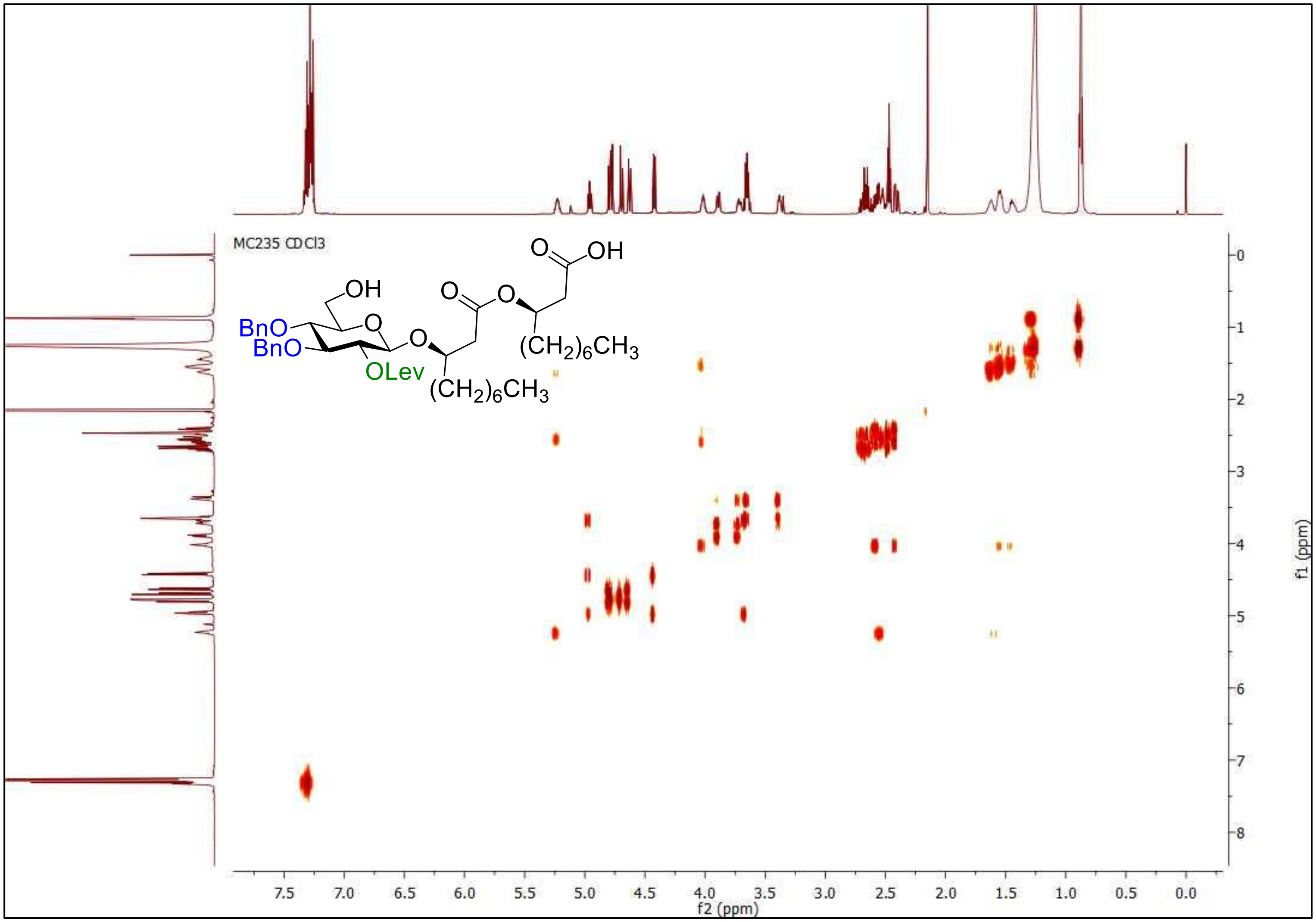

Figure S73 | ^{13}C NMR spectrum ($CDCl_3$, 600 MHz) of (*R*)-3-*O*-[(*R*)-(3'-*O*-decyl)-3,4-di-*O*-benzyl-2-*O*-levulinoyl-*β*-D-glucopyranosyl] decanoic acid (**22**).

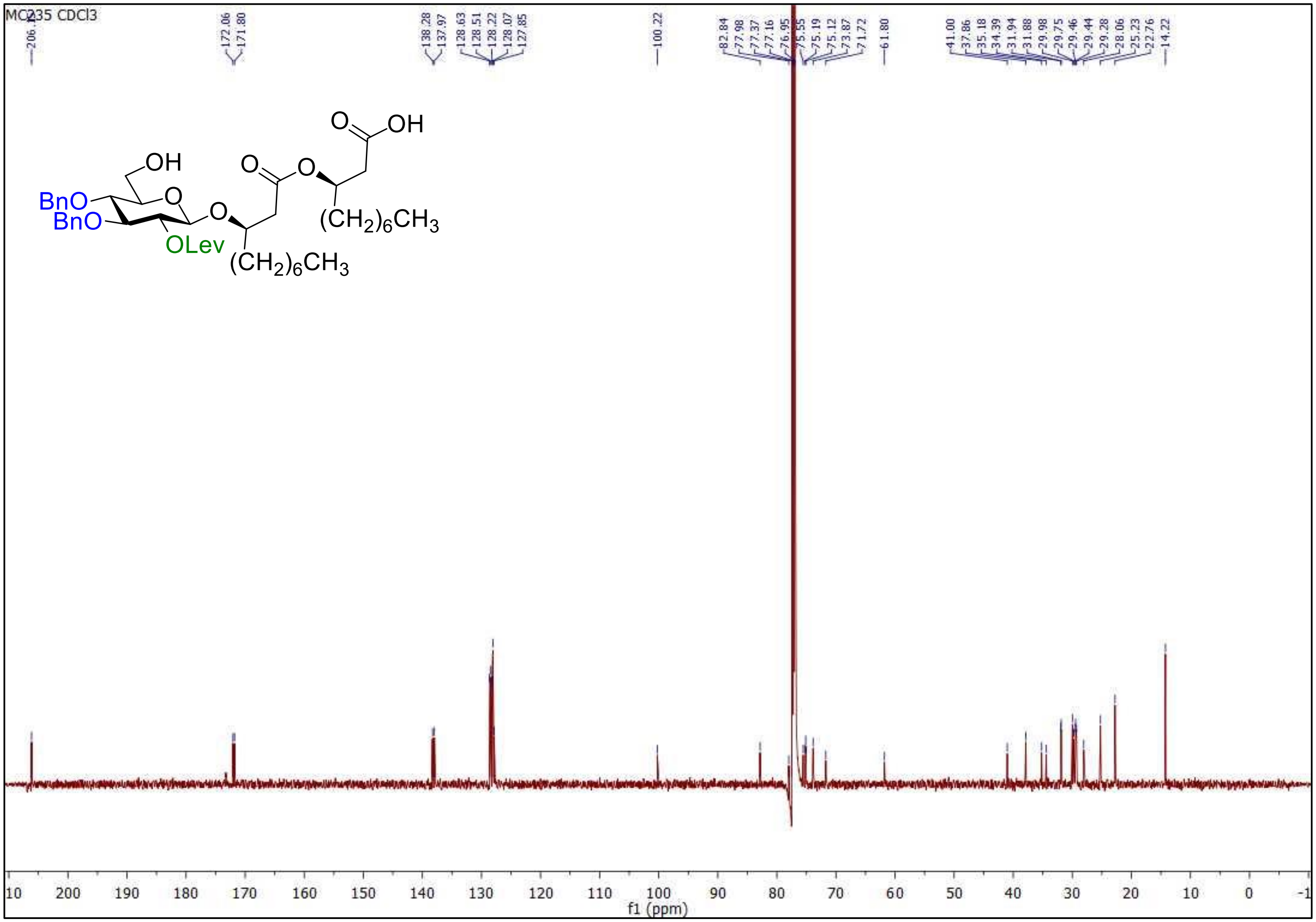

Figure S74 | HSQC NMR spectrum ($CDCl_3$, 600 MHz) of (*R*)-3-*O*-[(*R*)-(3'-*O*-decyl)-3,4-di-*O*-benzyl-2-*O*-levulinoyl-*β*-D-glucopyranosyl] decanoic acid (**22**).

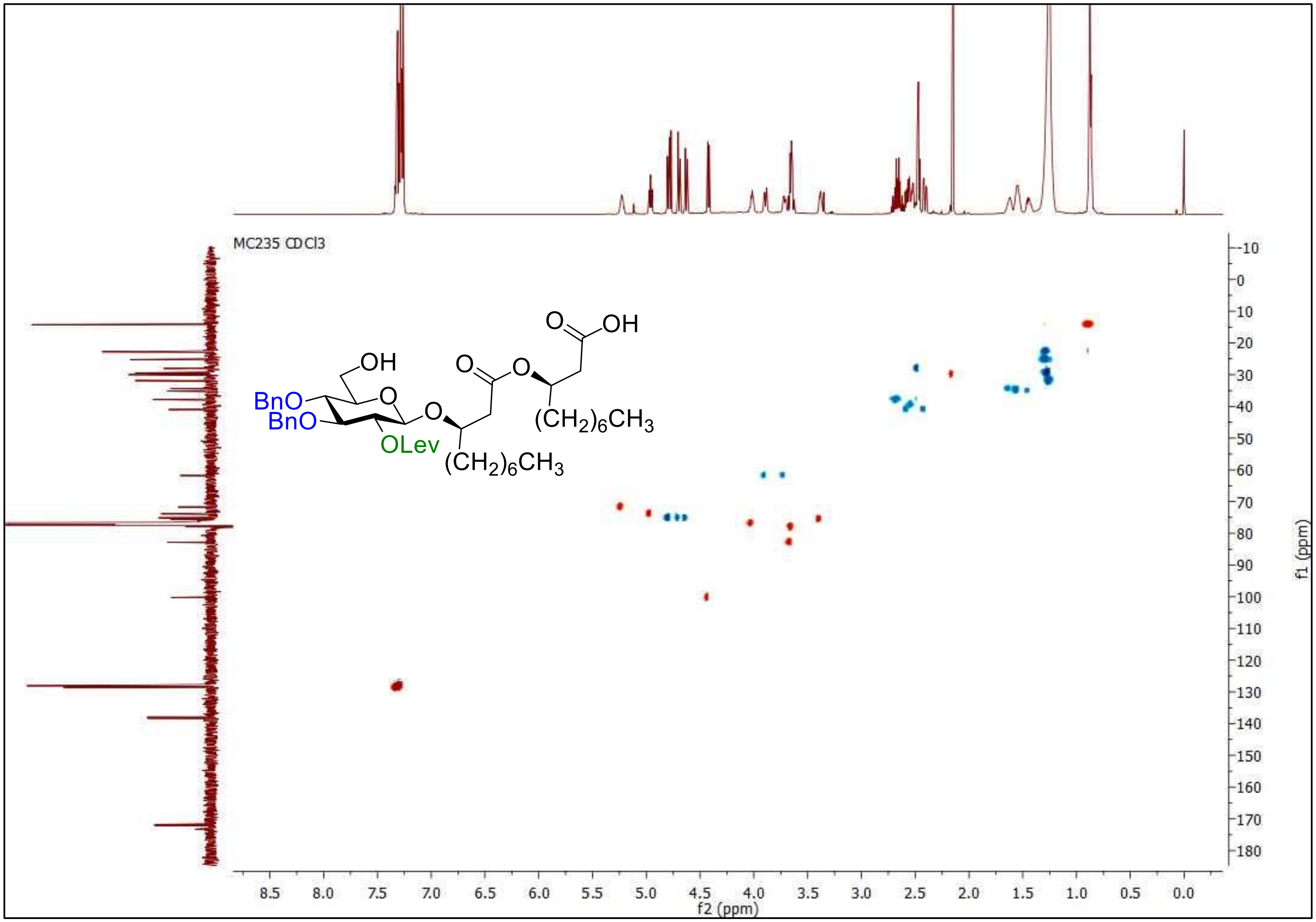

Figure S75 | ^{1}H NMR spectrum ($CDCl_3$, 600 MHz) of macrolide **23**.

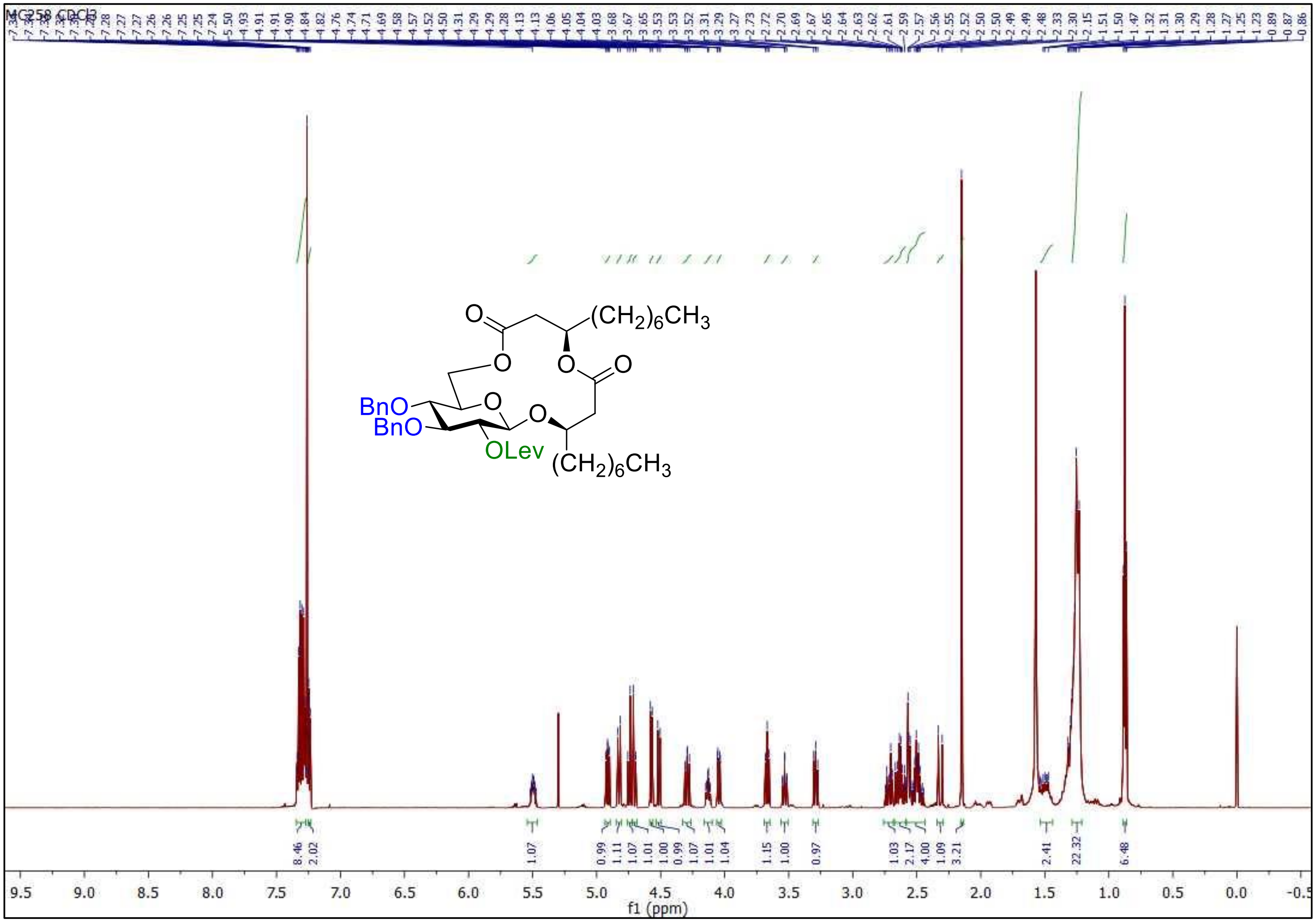

Figure S76 | COSY NMR spectrum ($CDCl_3$, 600 MHz) of macrolide **23**.

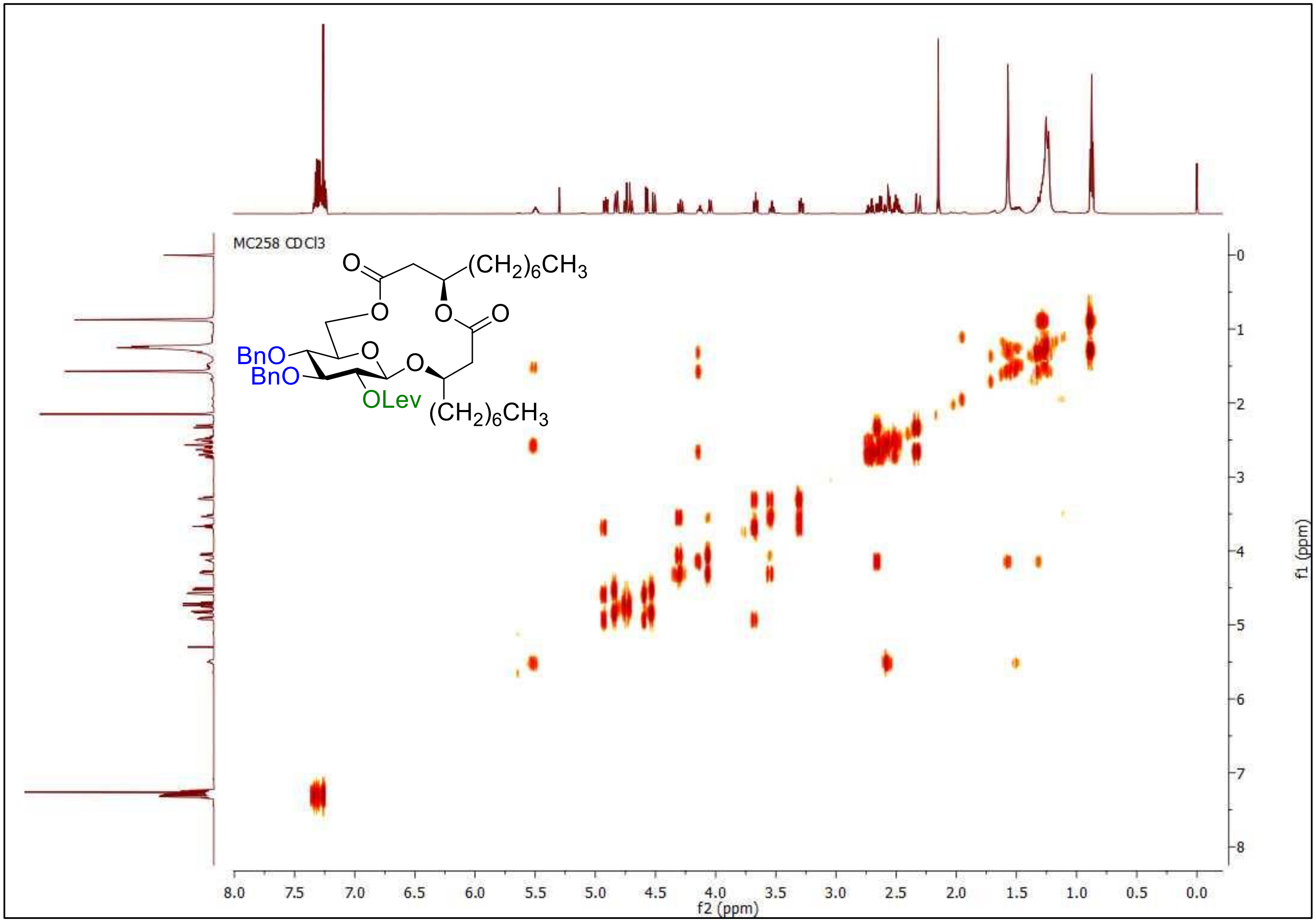

Figure S77 | ^{13}C NMR spectrum ($CDCl_3$, 600 MHz) of macrolide **23**.

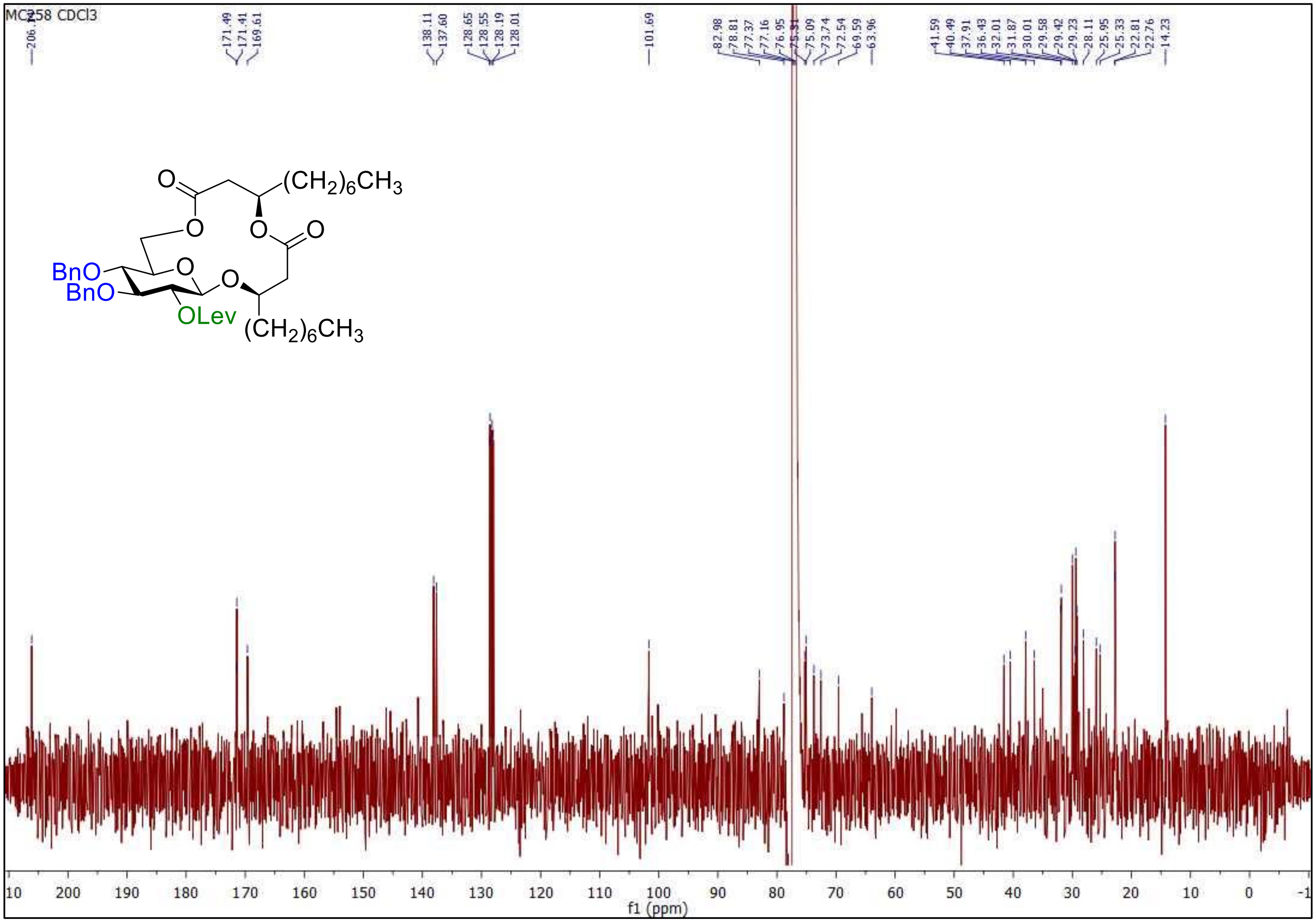

Figure S78 | HSQC NMR spectrum ($CDCl_3$, 600 MHz) of macrolide **23**.

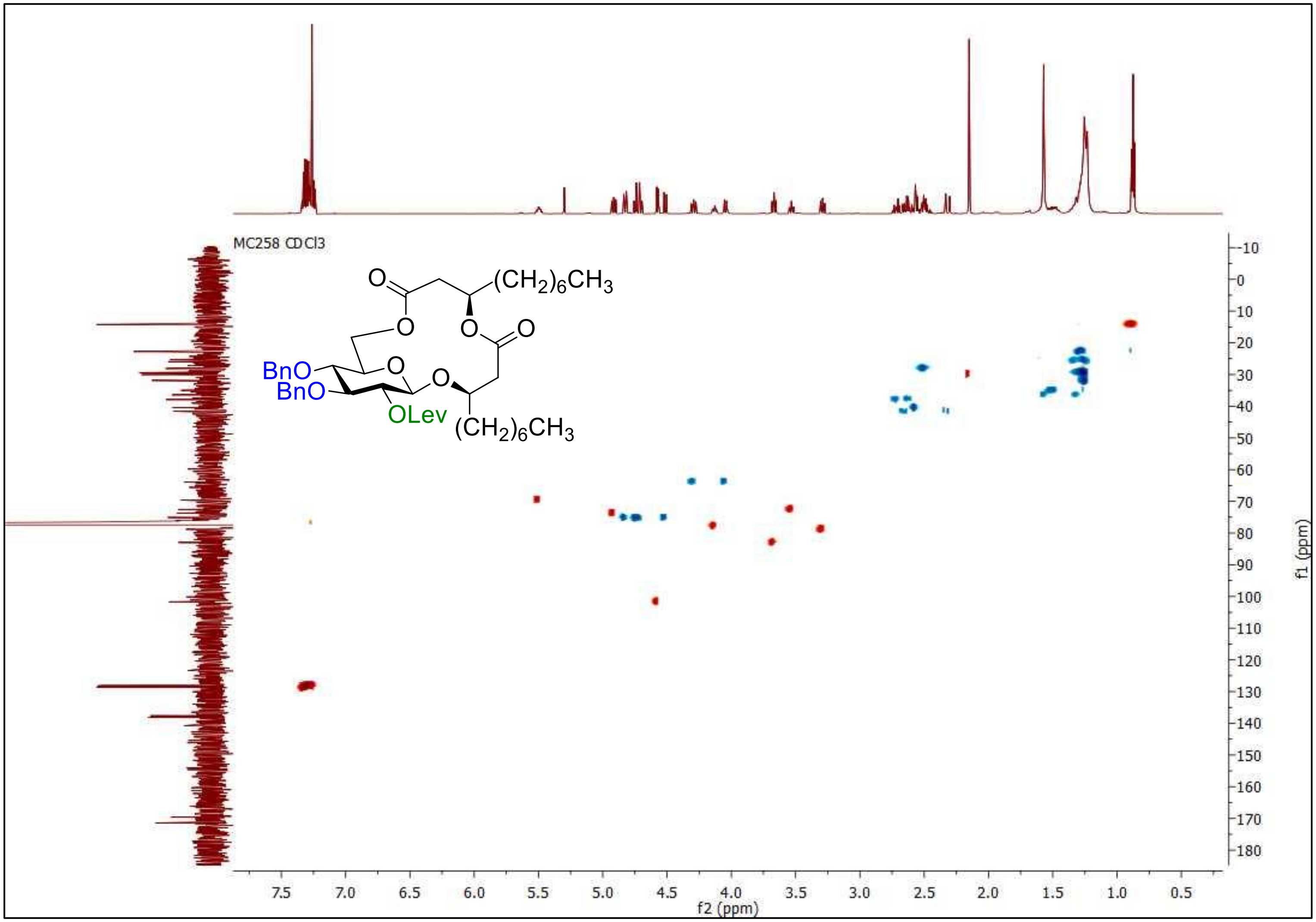

Figure S79 | ^{1}H NMR spectrum ($CDCl_3$, 600 MHz) of macrolide **S12.**

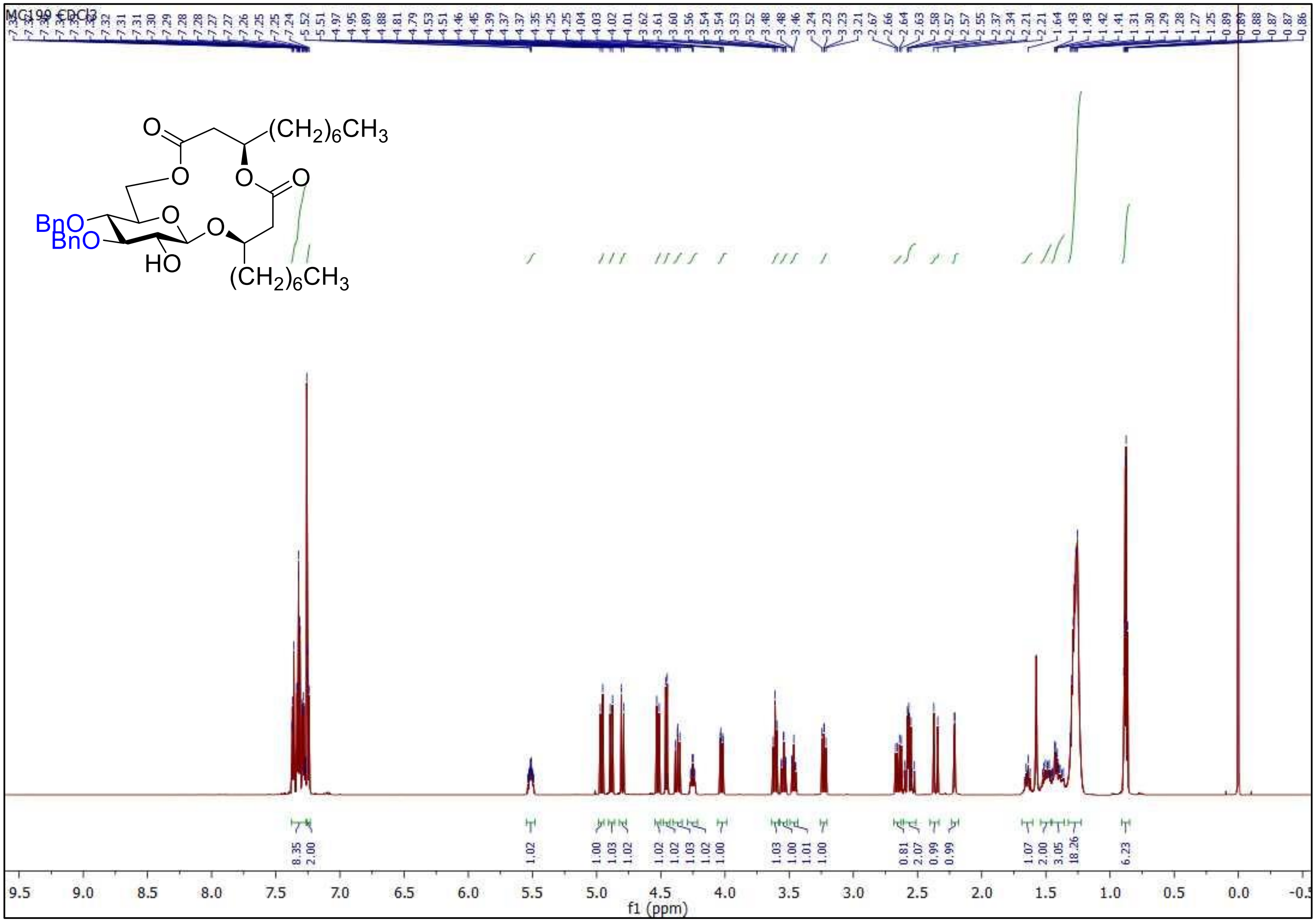

Figure S80 | COSY NMR spectrum ($CDCl_3$, 600 MHz) of macrolide **S12**.

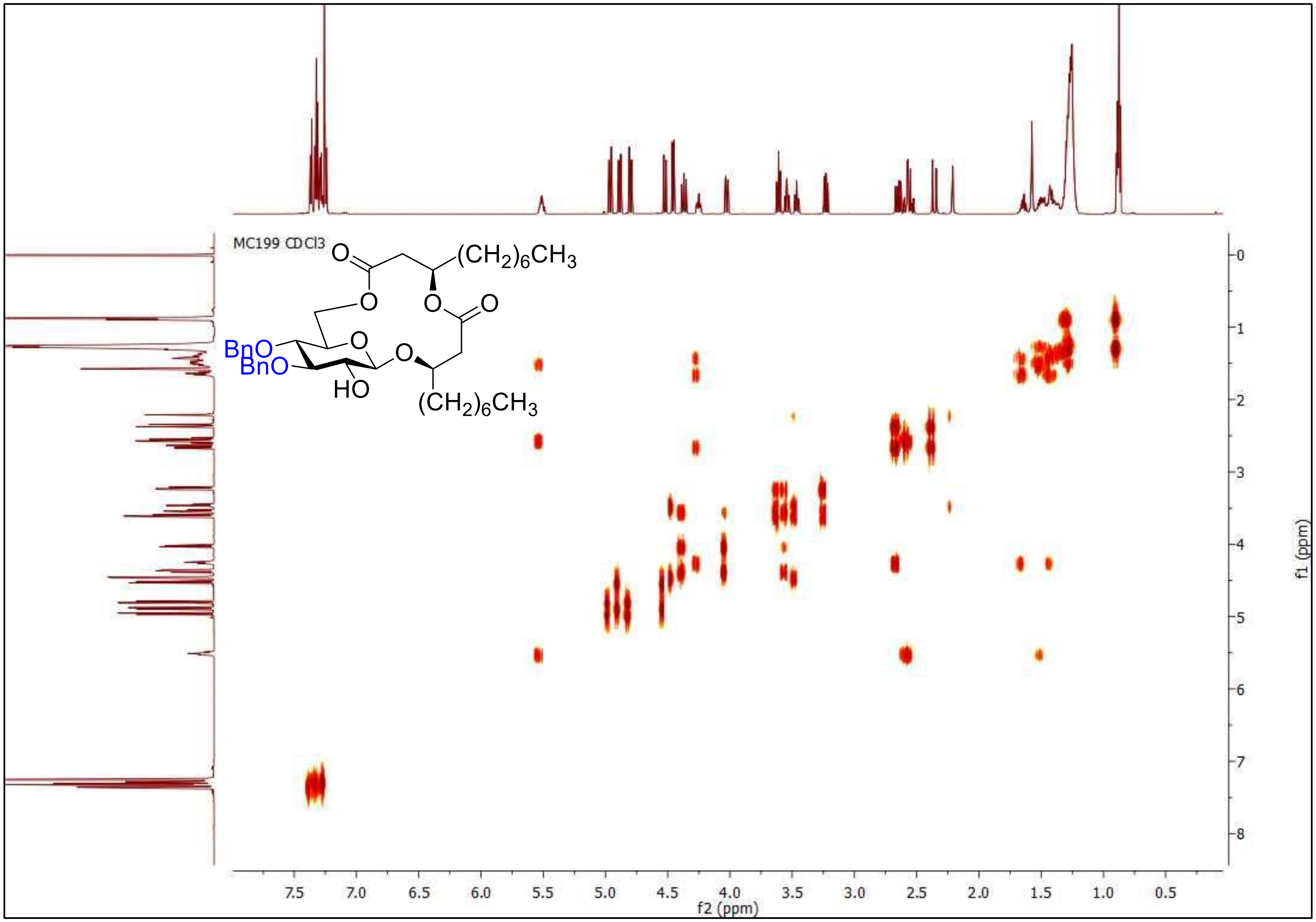

Figure S81 | ^{13}C NMR spectrum ($CDCl_3$, 600 MHz) of macrolide **S12**.

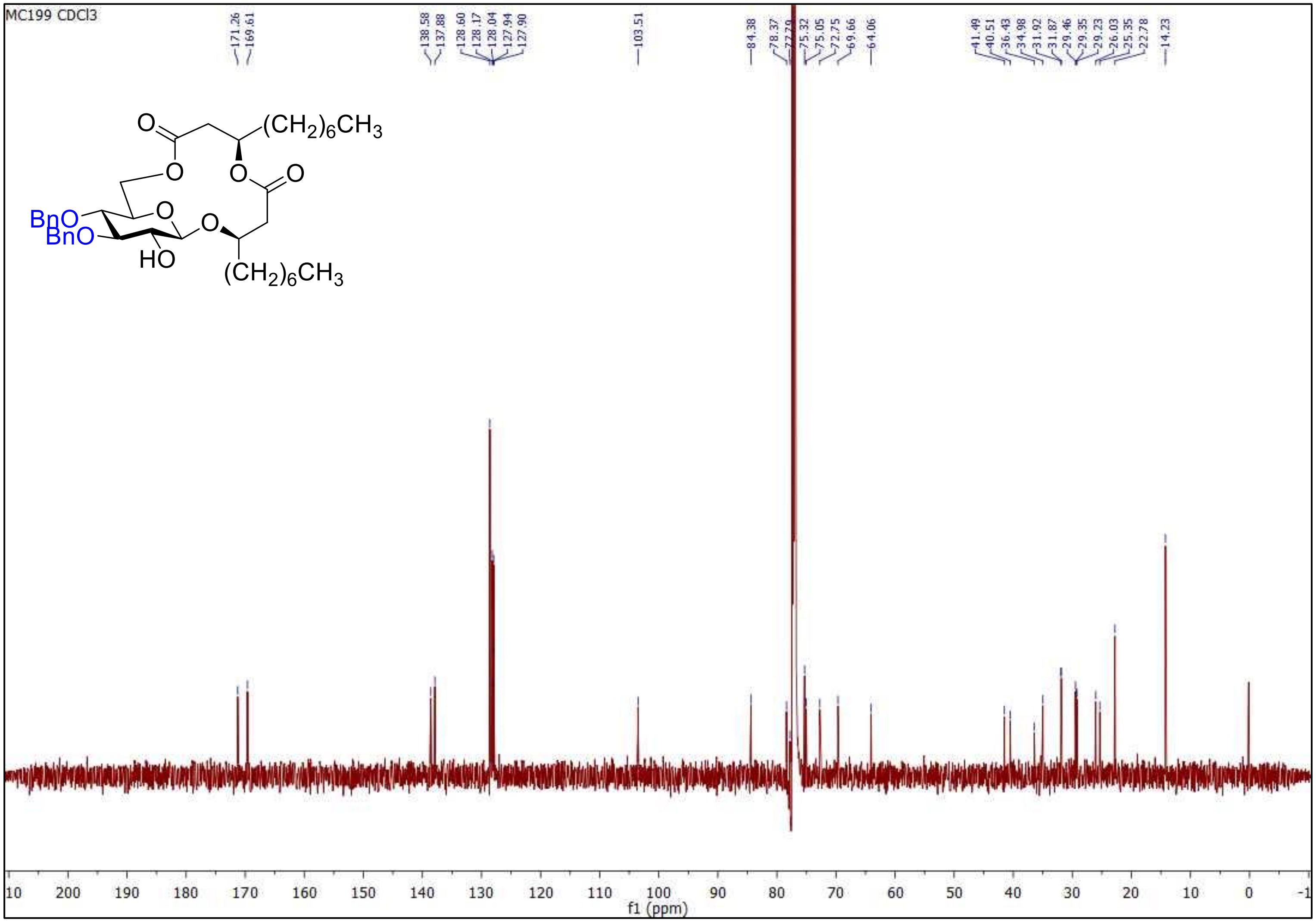

Figure S82 | HSQC NMR spectrum ($CDCl_3$, 600 MHz) of macrolide **S12**.

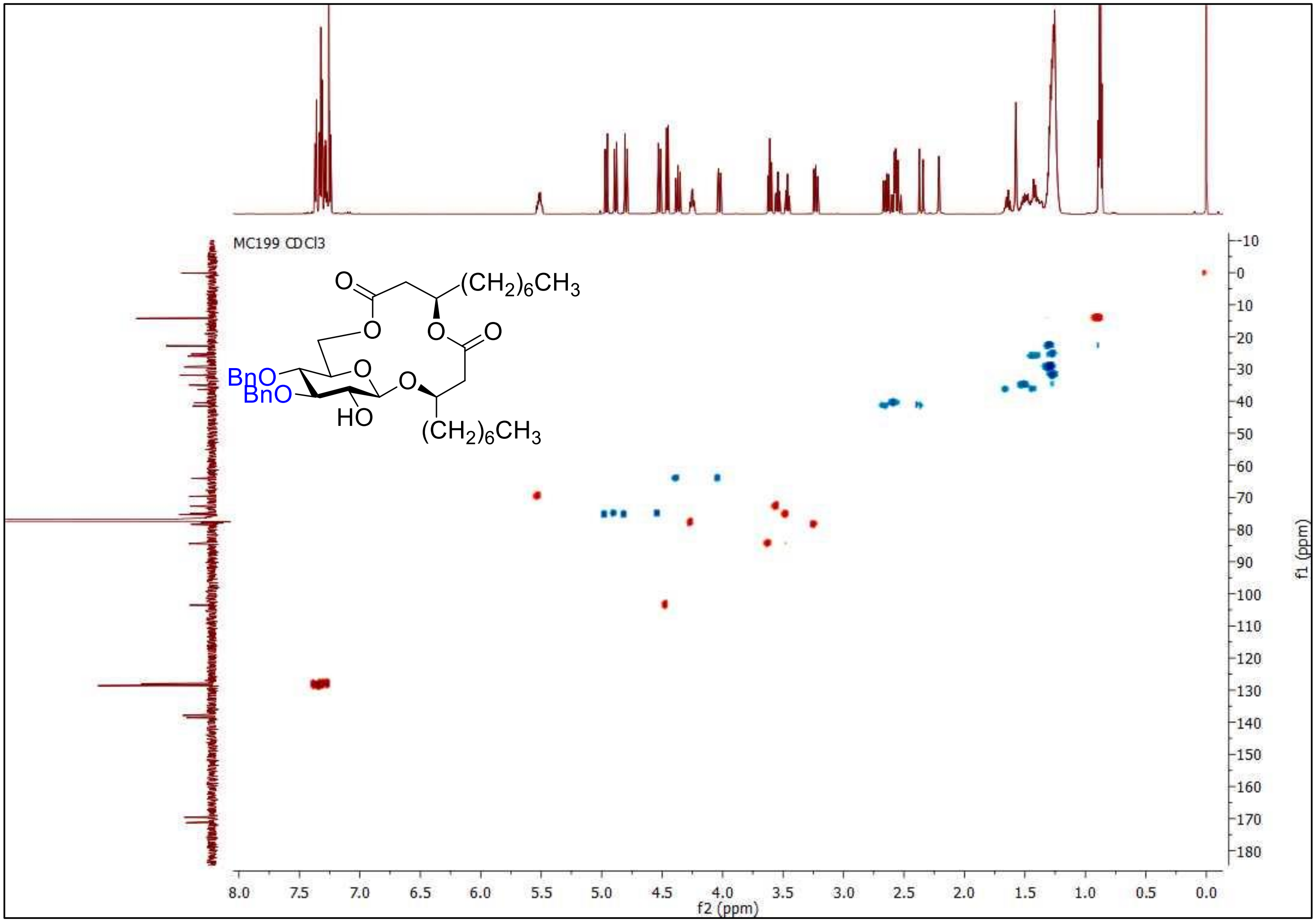

Figure S83 | ^{1}H NMR spectrum (pyr-d_5, 600 MHz) of ananatoside A (**1**).

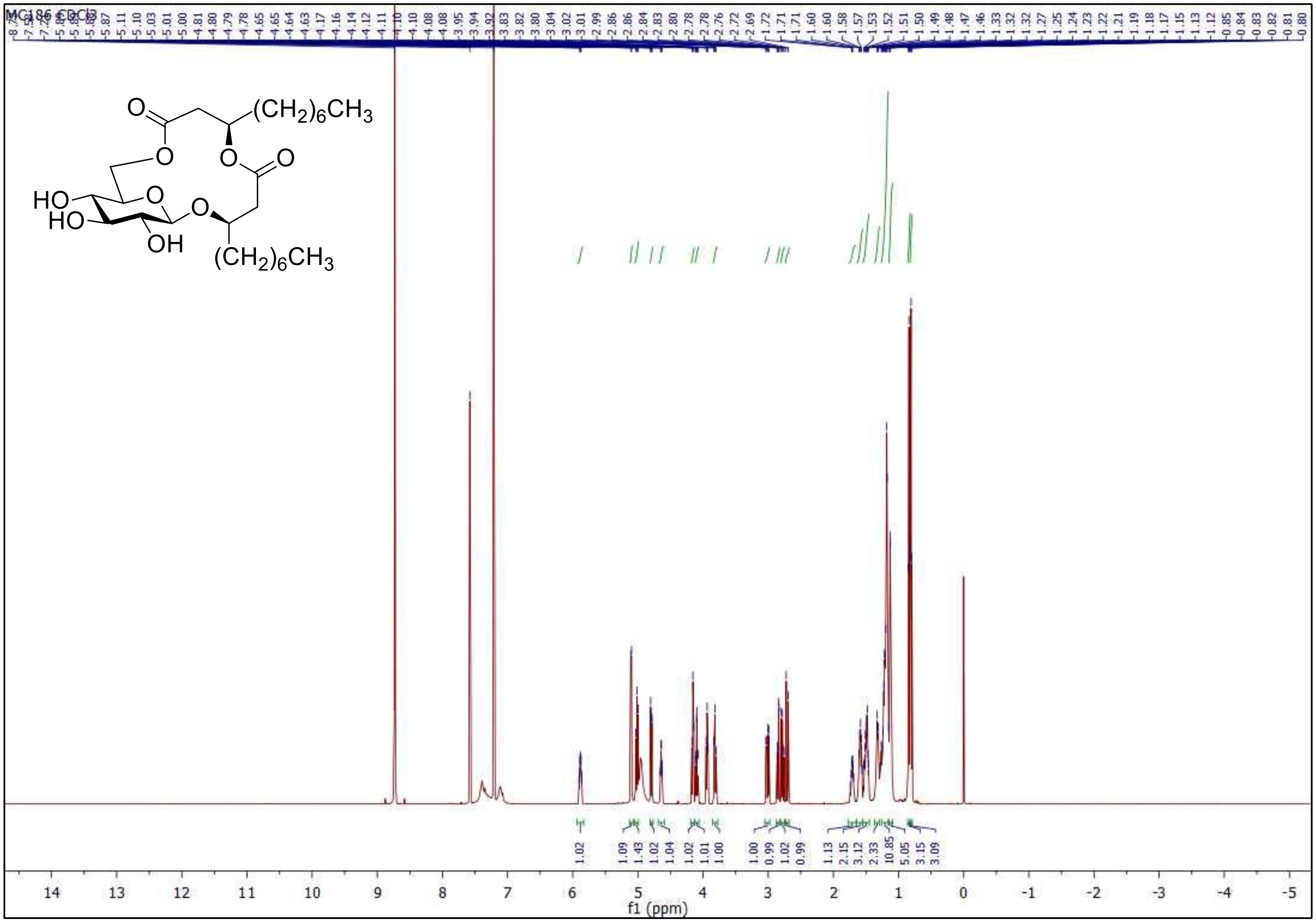

Figure S84 | ^{1}H NMR spectrum ($CDCl_3$, 600 MHz) of *para*-methylphenyl 4-*O*-levulinoyl-3-*O*-*para*-methoxybenzyl-1-thio-α-L-rhamnopyranoside (**S13**).

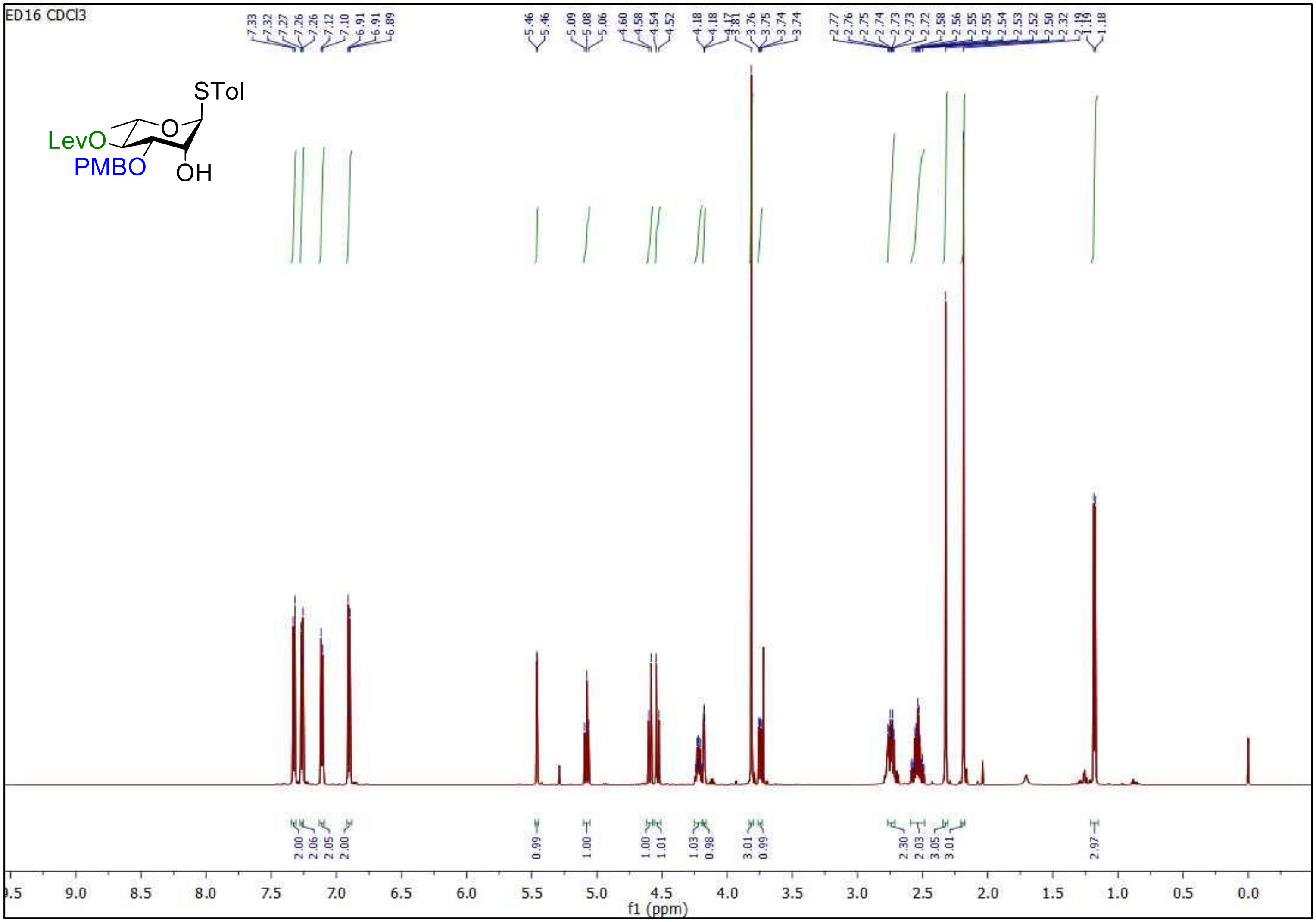

Figure S85 | COSY NMR spectrum ($CDCl_3$, 600 MHz) of *para*-methylphenyl 4-*O*-levulinoyl-3-*O*-*para*-methoxybenzyl-1-thio-*α*-L-rhamnopyranoside (**S13**).

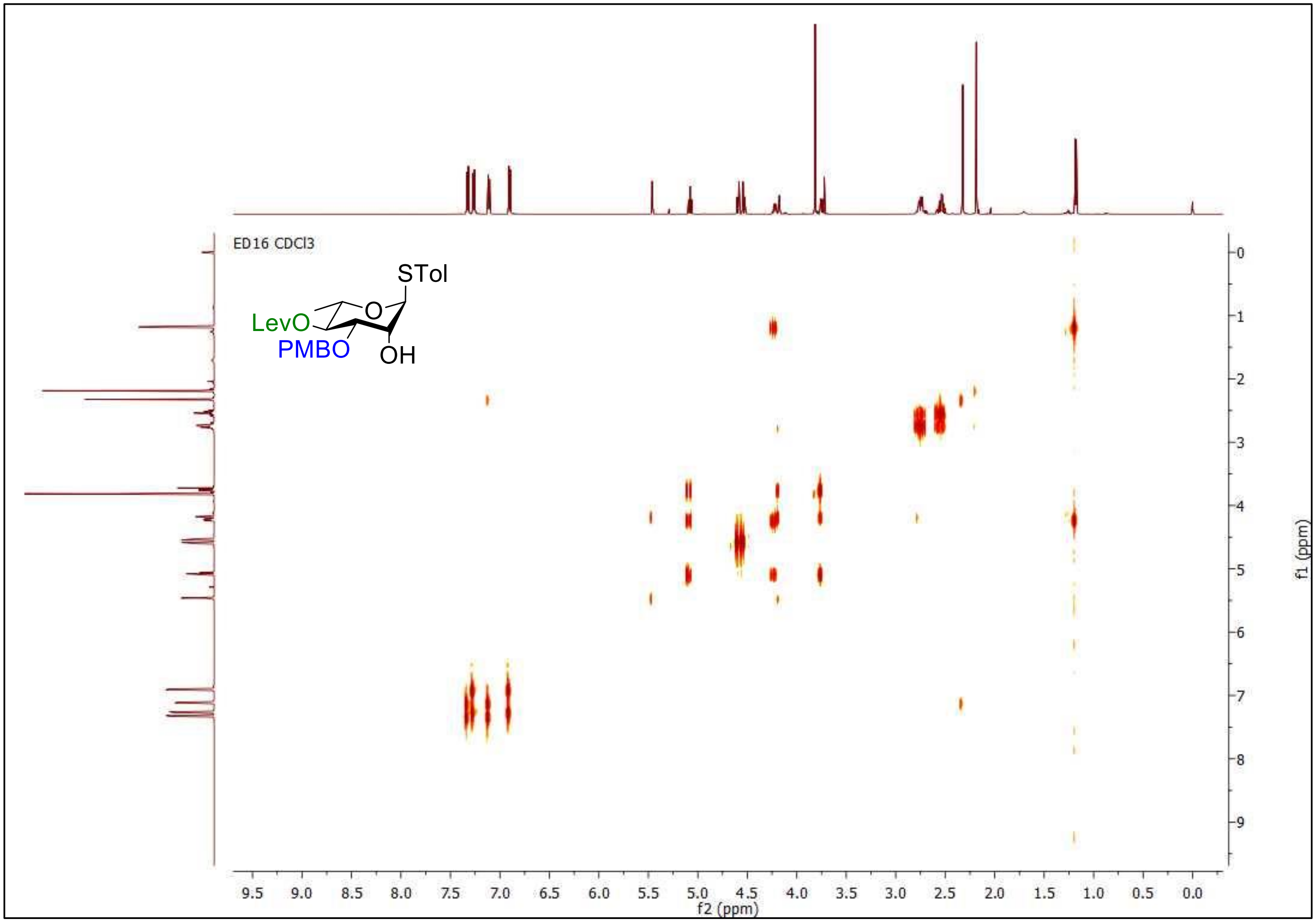

Figure S86 | ^{13}C NMR spectrum ($CDCl_3$, 600 MHz) of *para*-methylphenyl 4-*O*-levulinoyl-3-*O*-*para*-methoxybenzyl-1-thio-*α*-L-rhamnopyranoside (**S13**).

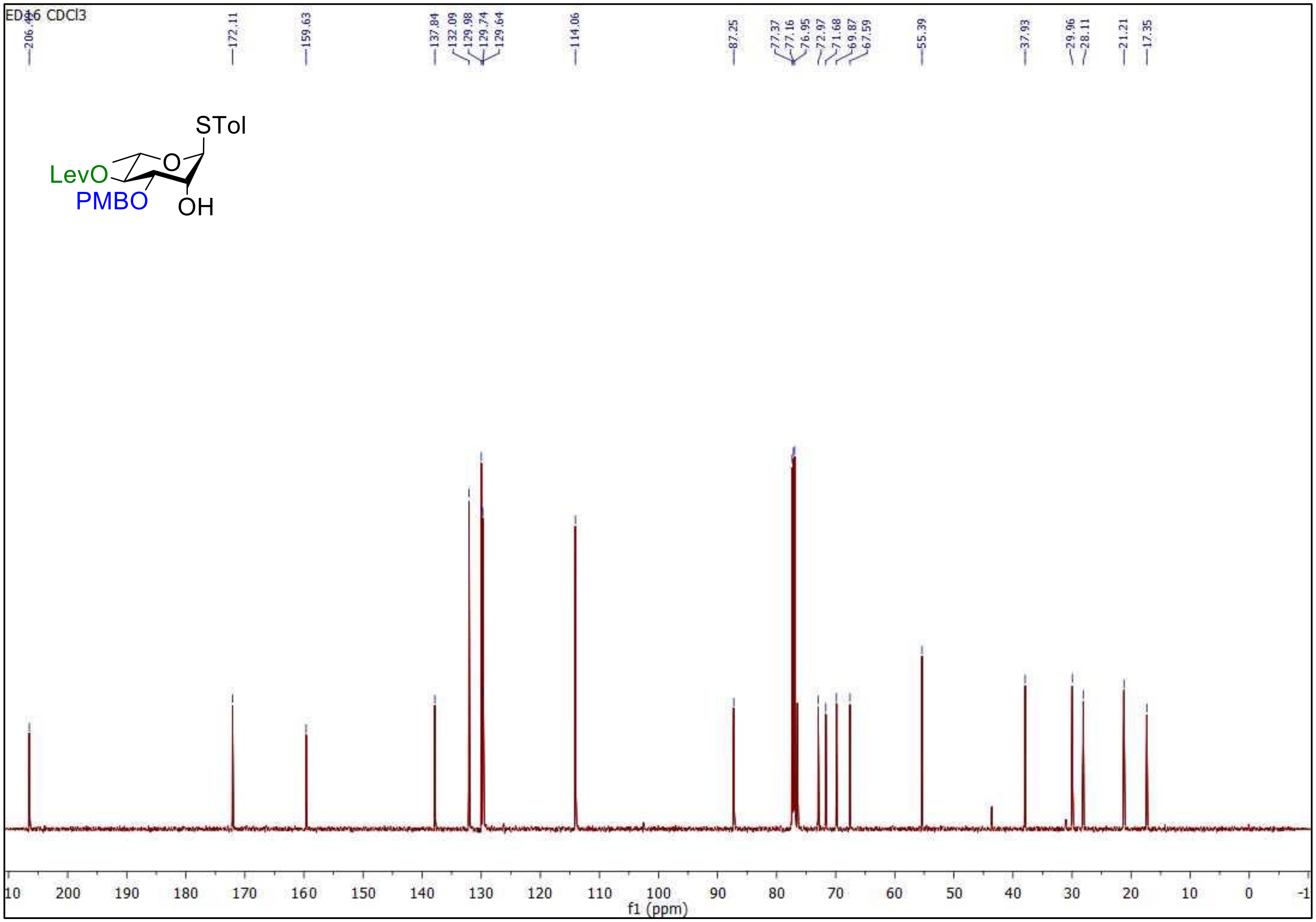

Figure S87 | HSQC NMR spectrum ($CDCl_3$, 600 MHz) of *para*-methylphenyl 4-*O*-levulinoyl-3-*O*-*para*-methoxybenzyl-1-thio-α-L-rhamnopyranoside (**S13**).

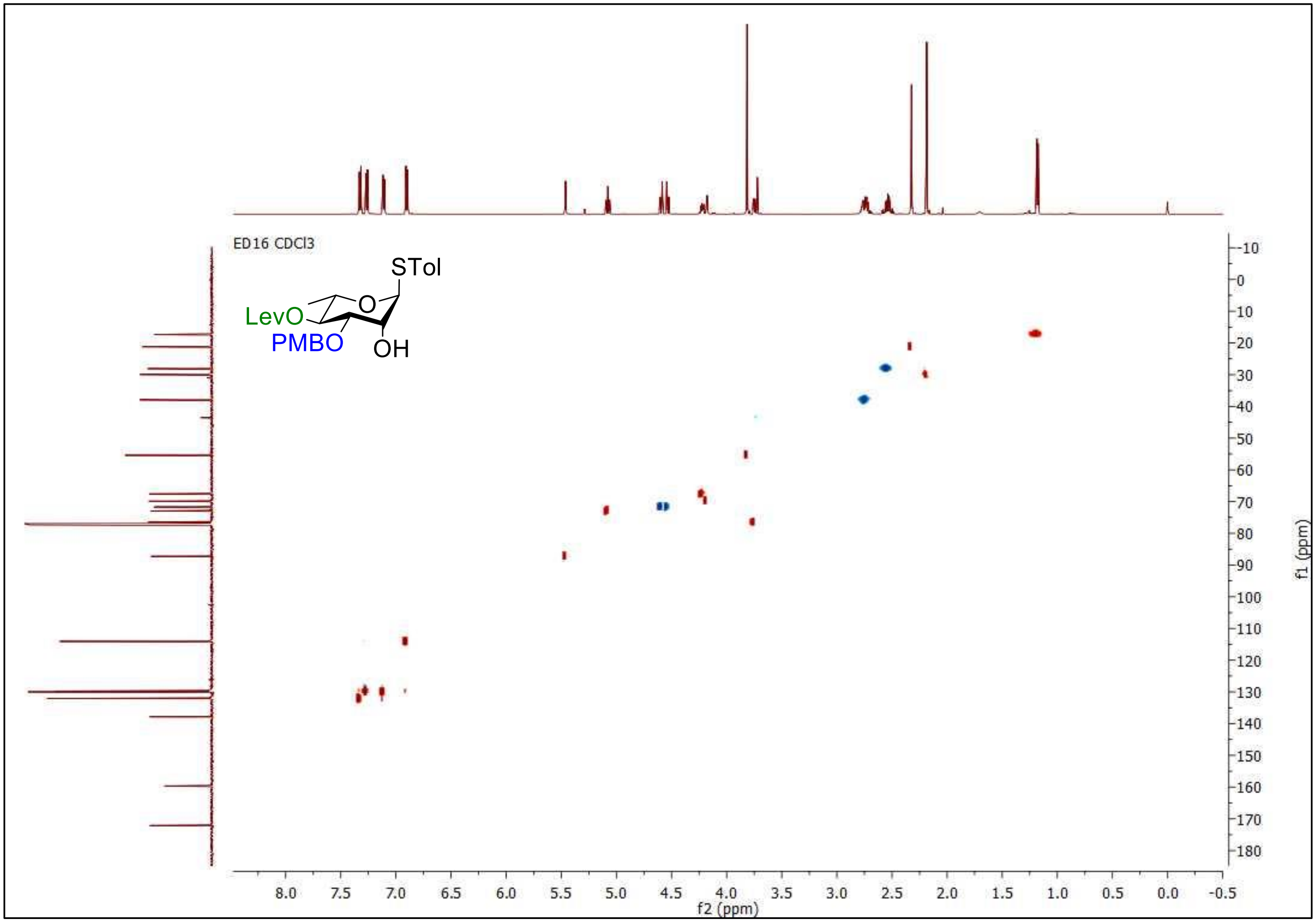

Figure S88 | ^{1}H NMR spectrum ($CDCl_3$, 600 MHz) of *para*-methylphenyl 2-*O*-*ortho*-(azidomethyl)benzoyl-4-*O*-levulinoyl-3-*O*-*para*-methoxybenzyl-1-thio-α-L-rhamnopyranoside (**25**).

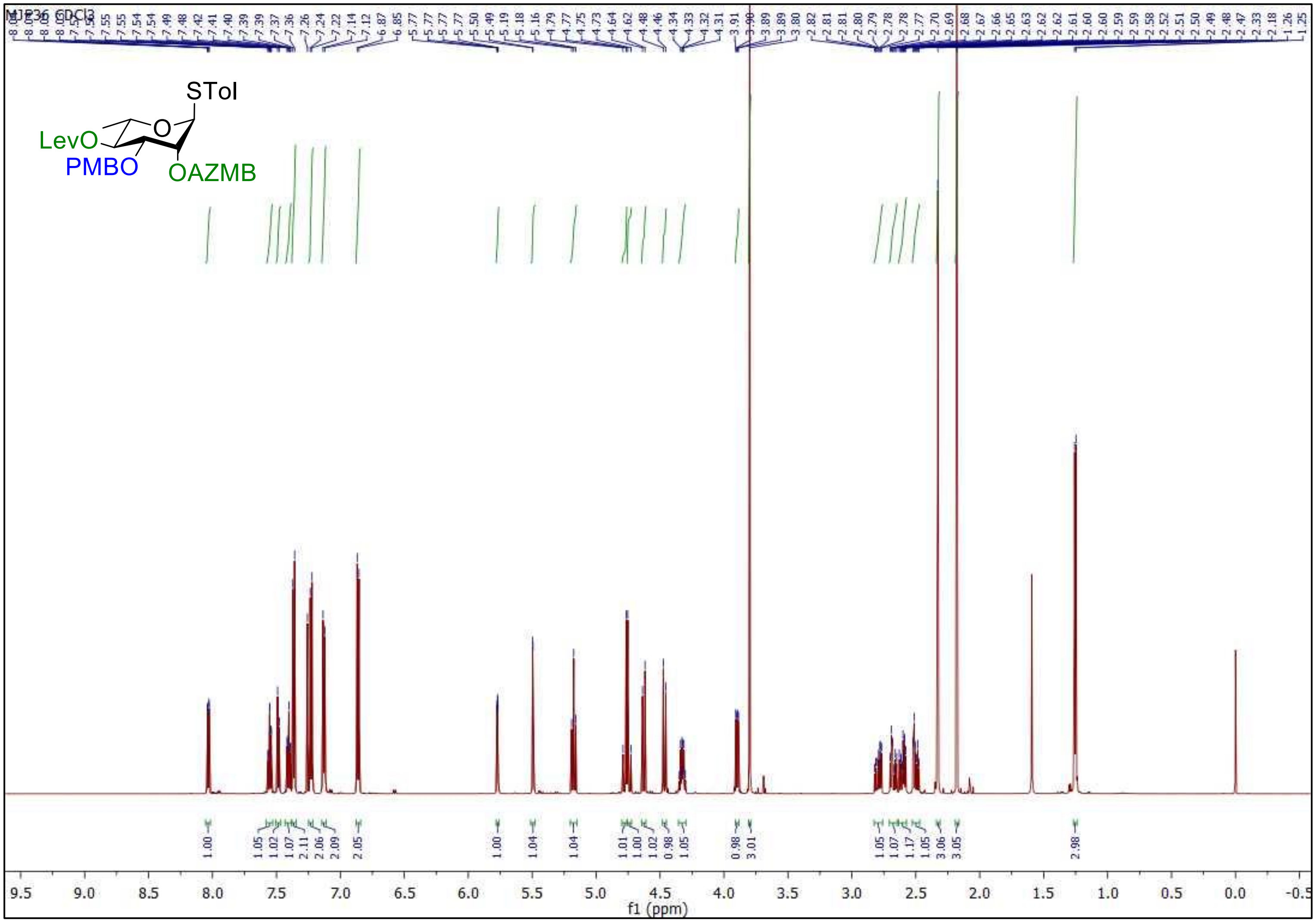

Figure S89 | COSY NMR spectrum ($CDCl_3$, 600 MHz) of *para*-methylphenyl 2-*O*-*ortho*-(azidomethyl)benzoyl-4-*O*-levulinoyl-3-*O*-*para*-methoxybenzyl-1-thio-*α*-L-rhamnopyranoside (**25**).

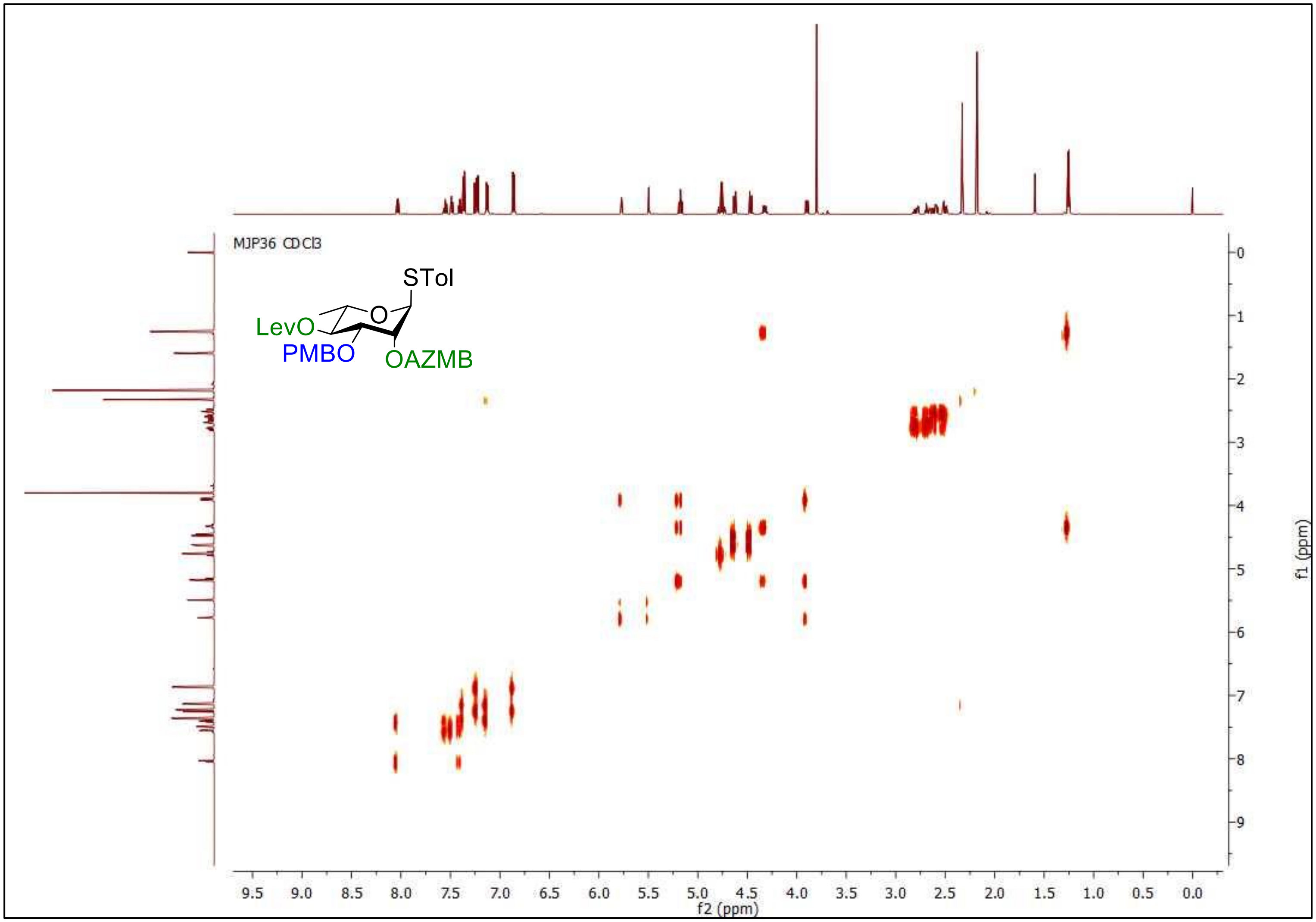

Figure S90 | ^{13}C NMR spectrum ($CDCl_3$, 600 MHz) of *para*-methylphenyl 2-*O*-*ortho*-(azidomethyl)benzoyl-4-*O*-levulinoyl-3-*O*-*para*-methoxybenzyl-1-thio-*α*-L-rhamnopyranoside (**25**).

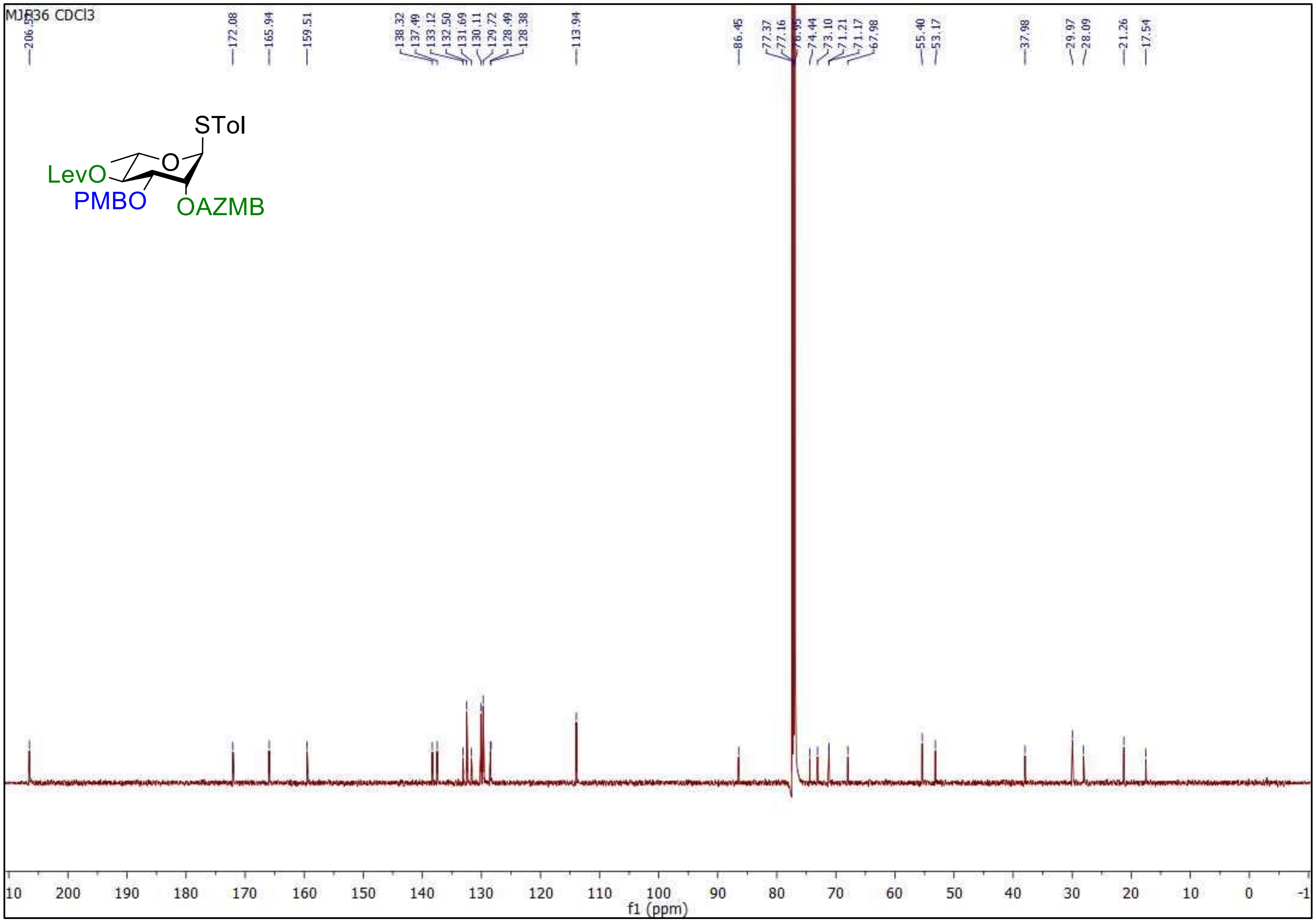

Figure S91 | HSQC NMR spectrum ($CDCl_3$, 600 MHz) of *para*-methylphenyl 2-*O*-*ortho*-(azidomethyl)benzoyl-4-*O*-levulinoyl-3-*O*-*para*-methoxybenzyl-1-thio-*α*-L-rhamnopyranoside (**25**).

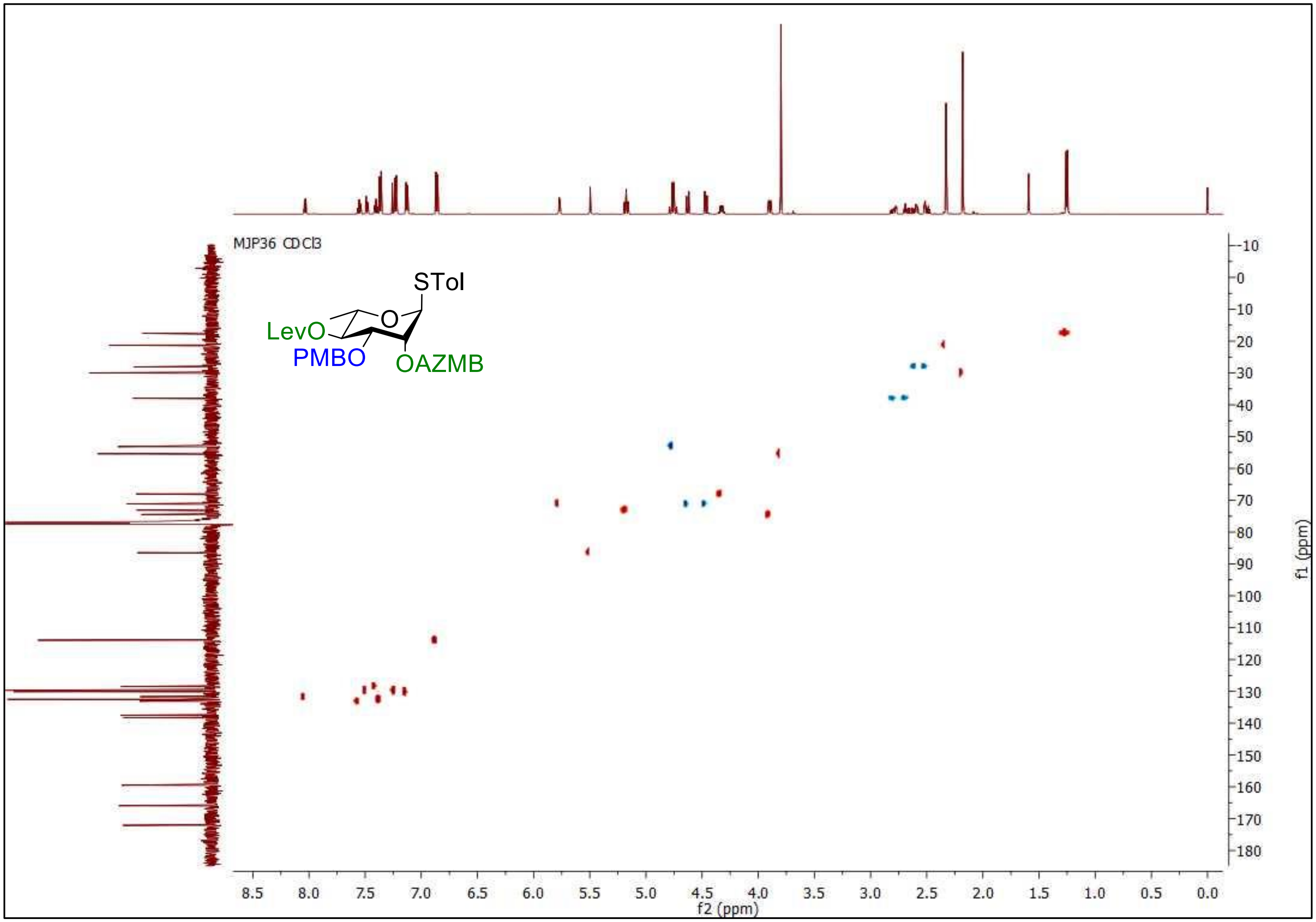

Figure S92 | ^{1}H NMR spectrum (CDCl$_3$, 600 MHz) of benzyl (*R*)-3-*O*-[(*R*)-(3'-*O*-decyl)-2-*O*-*ortho*-(azidomethyl)benzoyl-4-*O*-levulinoyl-3-*O*-*para*-methoxybenzyl-α-L-rhamnopyranosyl]decanoate (**24**).

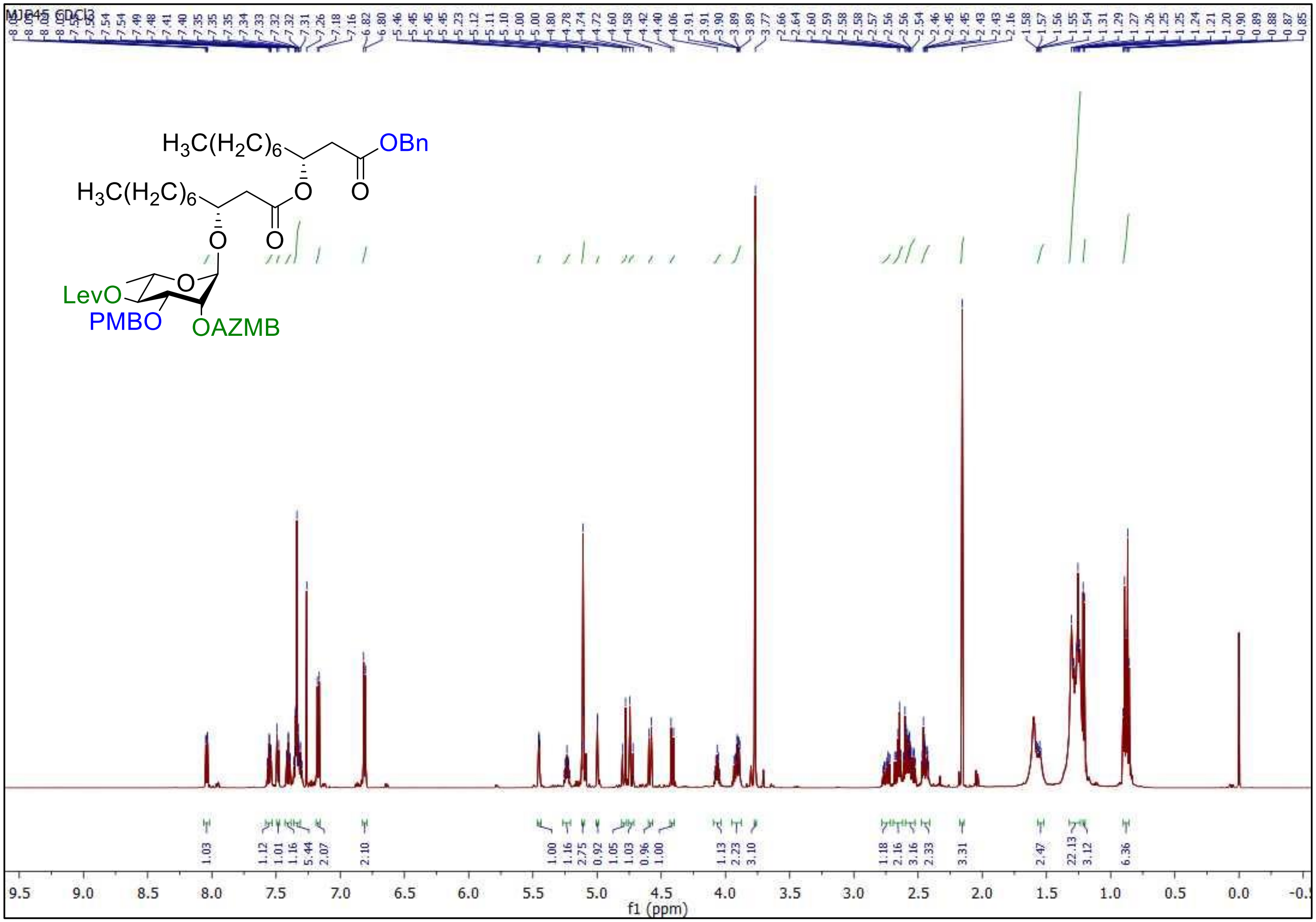

Figure S93 | COSY NMR spectrum ($CDCl_3$, 600 MHz) of benzyl (*R*)-3-*O*-[(*R*)-(3'-*O*-decyl)-2-*O*-*ortho*-(azidomethyl)benzoyl-4-*O*-levulinoyl-3-*O*-*para*-methoxybenzyl-α-L-rhamnopyranosyl]decanoate (**24**).

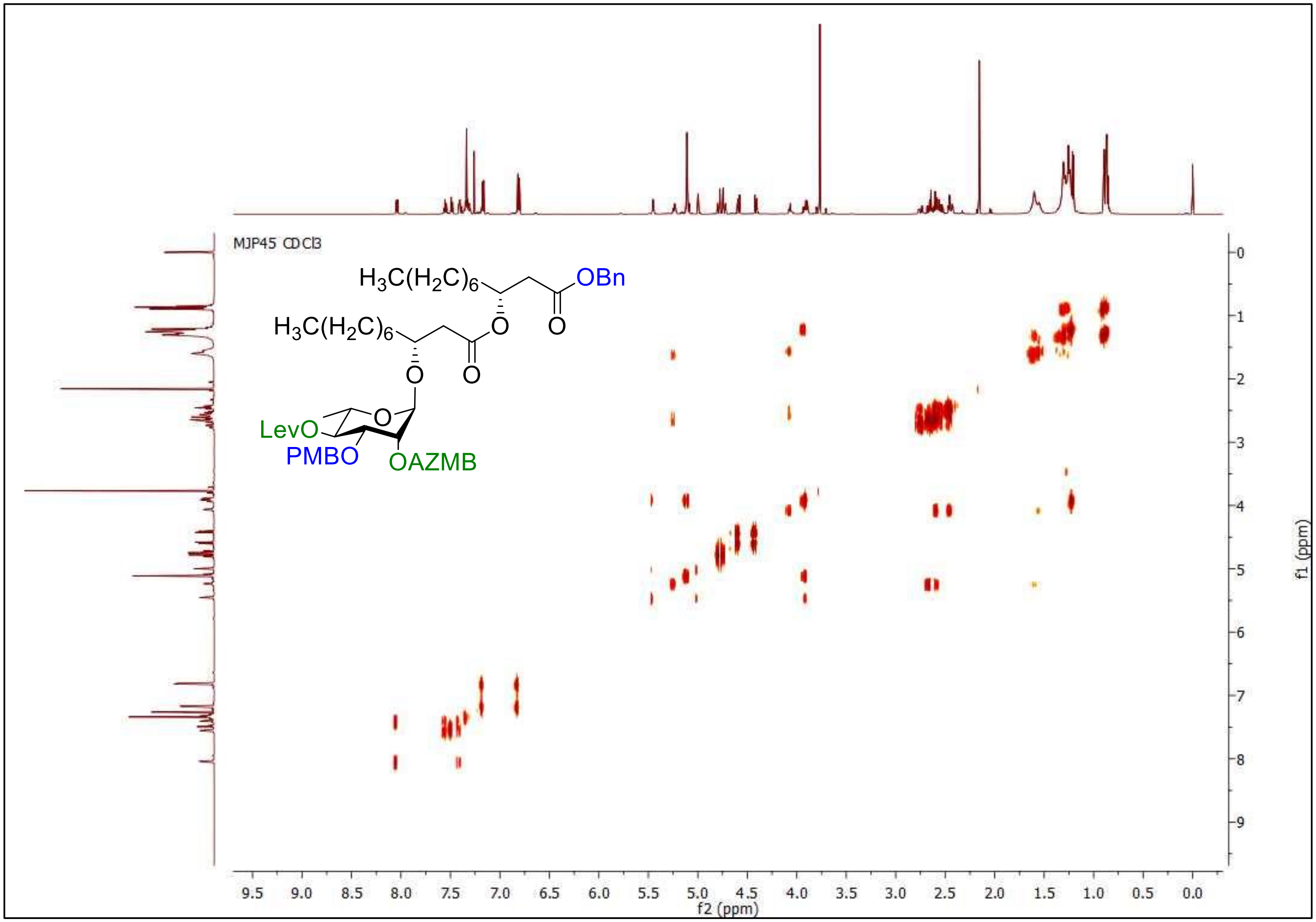

Figure S94 | ^{13}C NMR spectrum ($CDCl_3$, 600 MHz) of benzyl (*R*)-3-*O*-[(*R*)-(3'-*O*-decyl)-2-*O*-*ortho*-(azidomethyl)benzoyl-4-*O*-levulinoyl-3-*O*-*para*-methoxybenzyl-α-L-rhamnopyranosyl]decanoate (**24**).

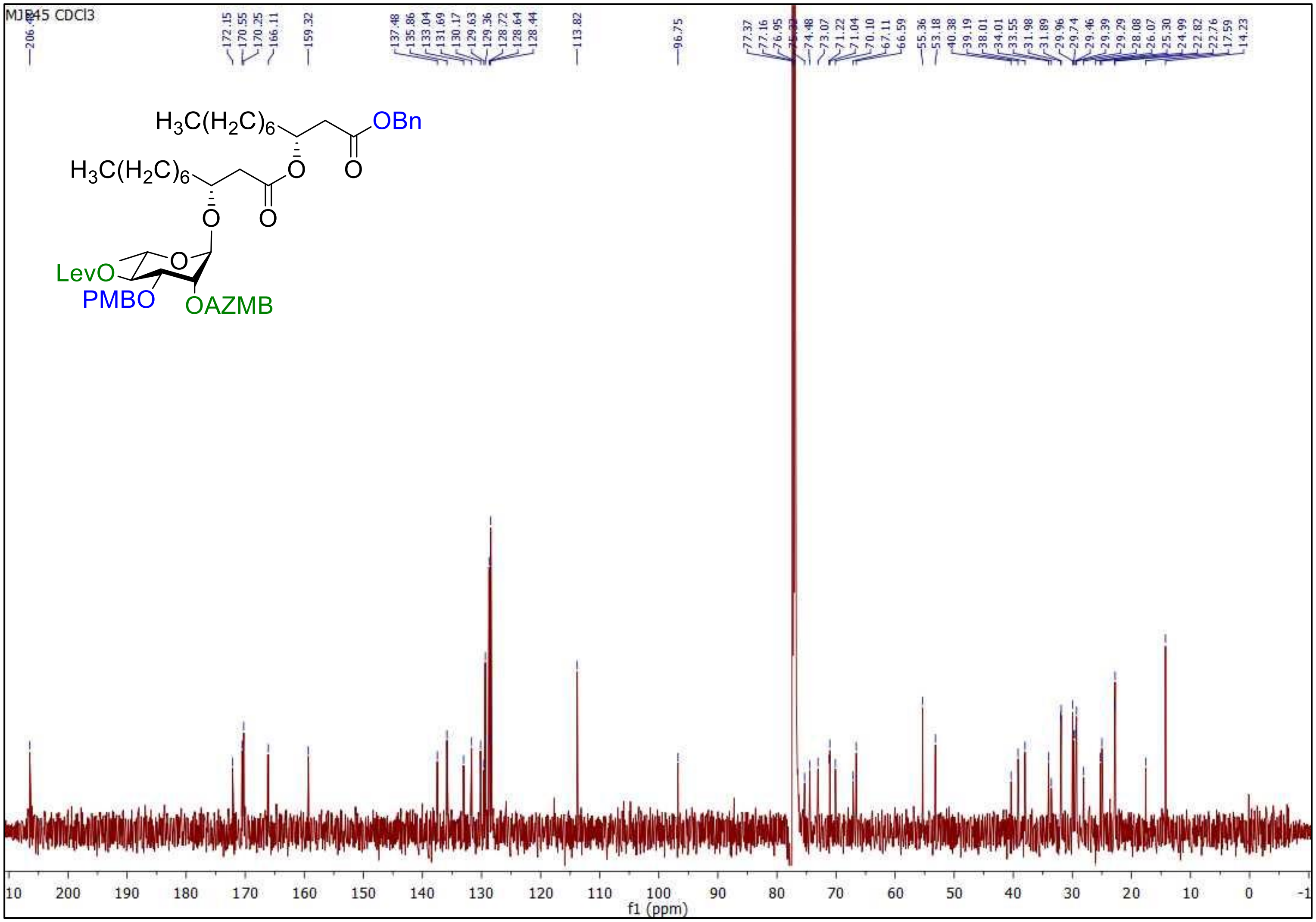

Figure S95 | HSQC NMR spectrum ($CDCl_3$, 600 MHz) of benzyl (*R*)-3-*O*-[(*R*)-(3'-*O*-decyl)-2-*O*-*ortho*-(azidomethyl)benzoyl-4-*O*-levulinoyl-3-*O*-*para*-methoxybenzyl-α-L-rhamnopyranosyl]decanoate (**24**).

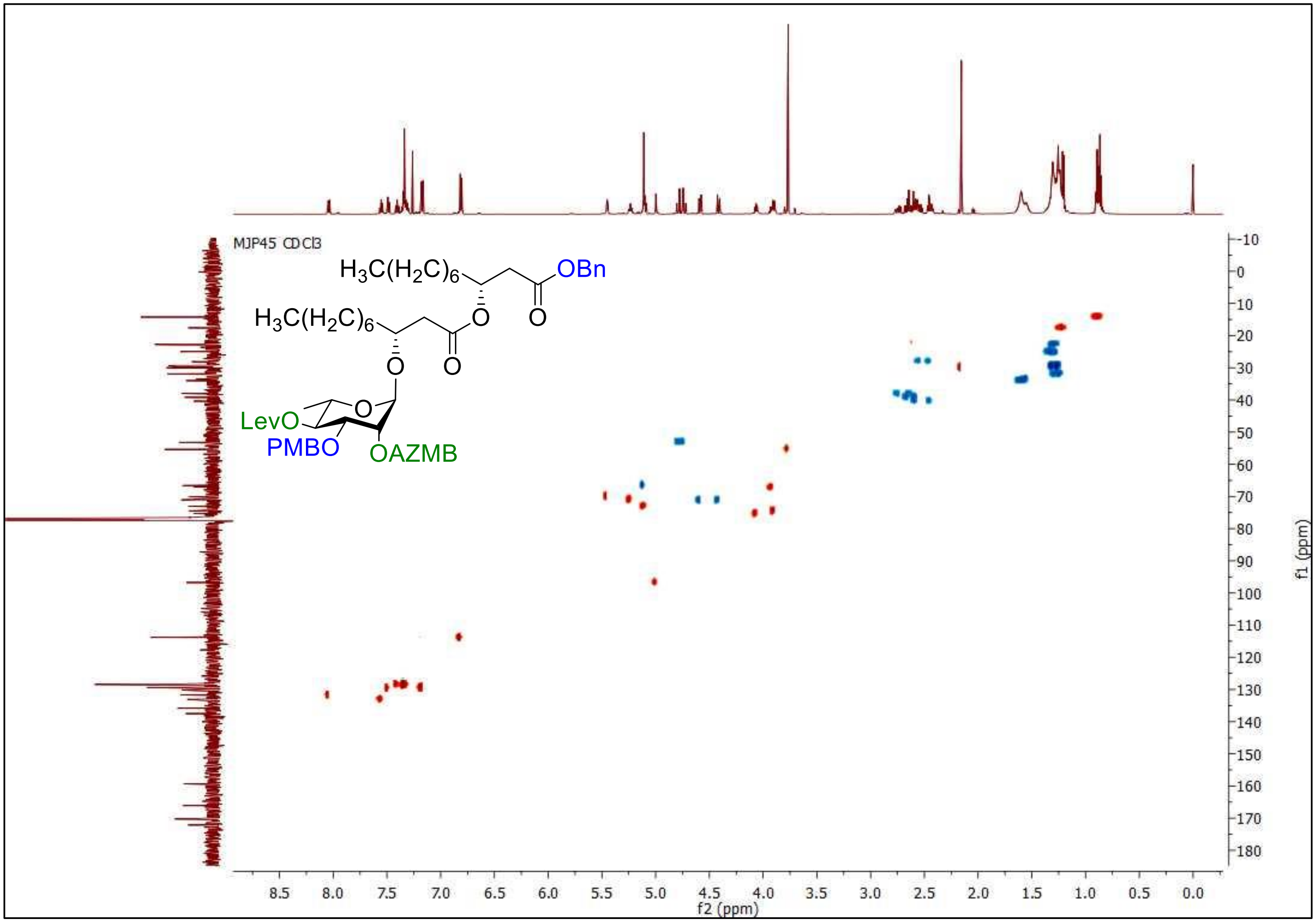

Figure S96 | ^{1}H NMR spectrum ($CDCl_3$, 600 MHz) of benzyl (*R*)-3-*O*-[(*R*)-(3'-*O*-decyl)-4-*O*-levulinoyl-3-*O*-*para*-methoxybenzyl-*α*-L-rhamnopyranosyl]decanoate (**S14**).

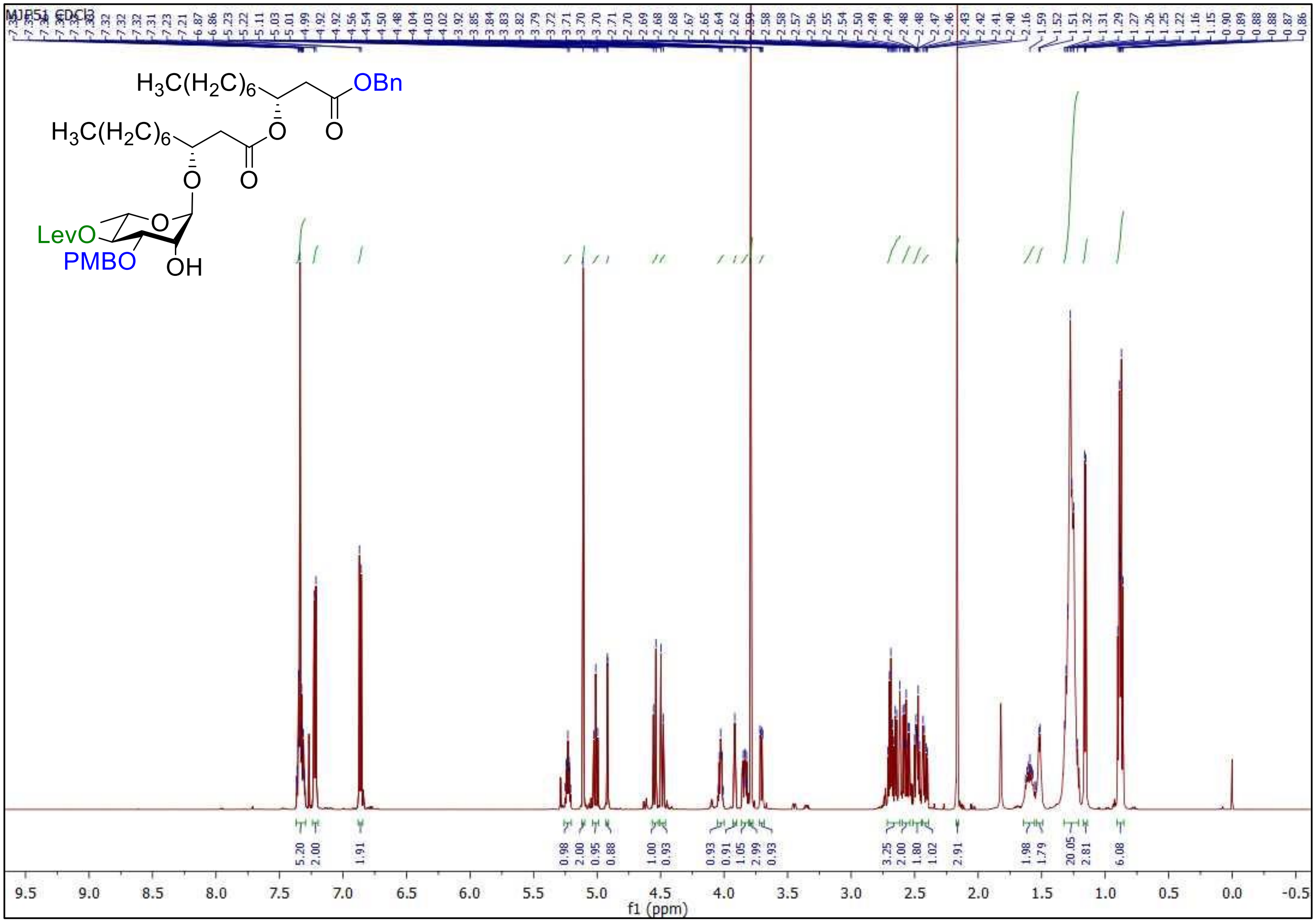

Figure S97 | COSY NMR spectrum ($CDCl_3$, 600 MHz) of benzyl (*R*)-3-*O*-[(*R*)-(3'-*O*-decyl)-4-*O*-levulinoyl-3-*O*-*para*-methoxybenzyl-α-L-rhamnopyranosyl]decanoate (**S14**).

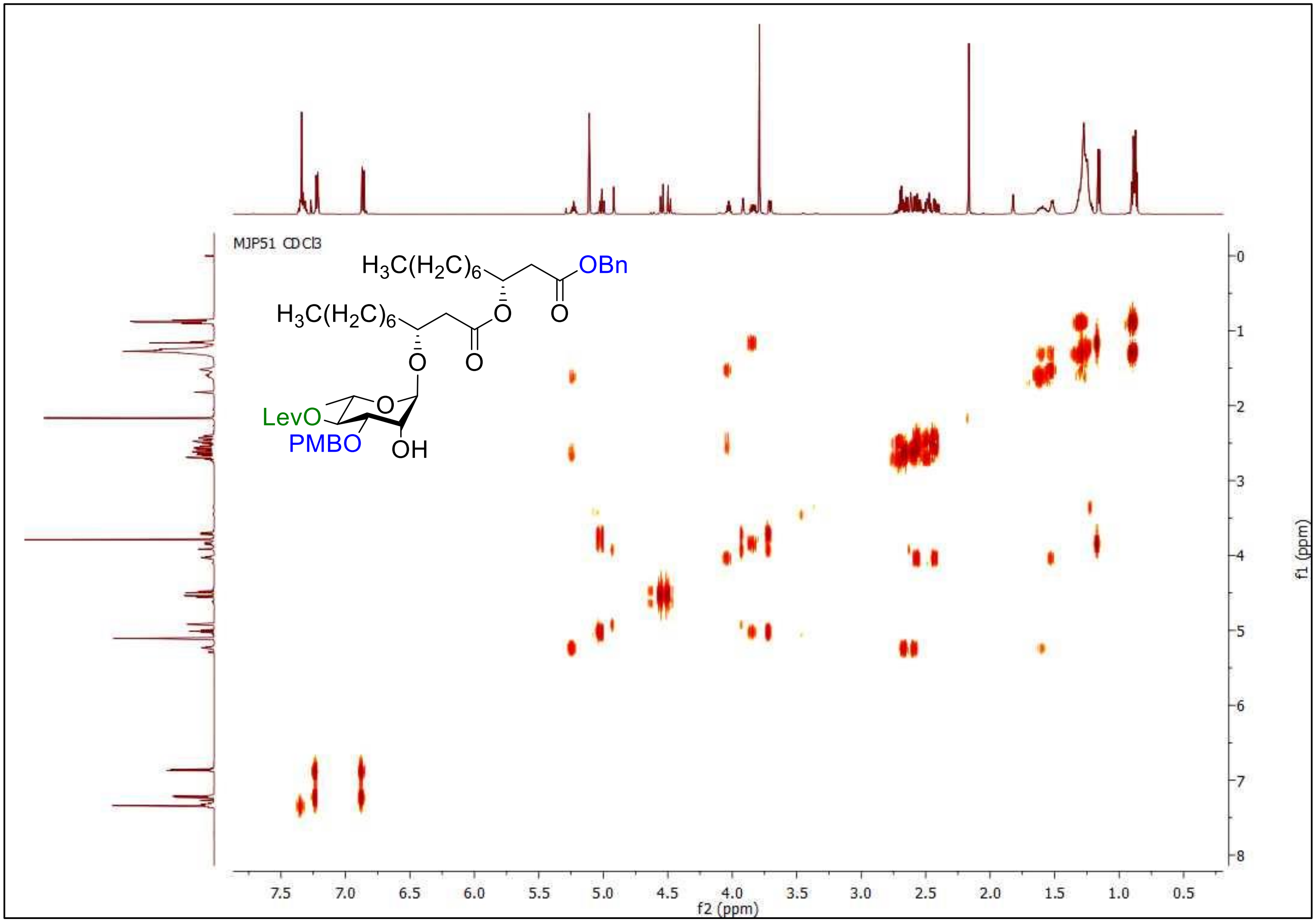

Figure S98 | ^{13}C NMR spectrum (CDCl$_3$, 600 MHz) of benzyl (*R*)-3-*O*-[(*R*)-(3'-*O*-decyl)-4-*O*-levulinoyl-3-*O*-*para*-methoxybenzyl-*α*-L-rhamnopyranosyl]decanoate (**S14**).

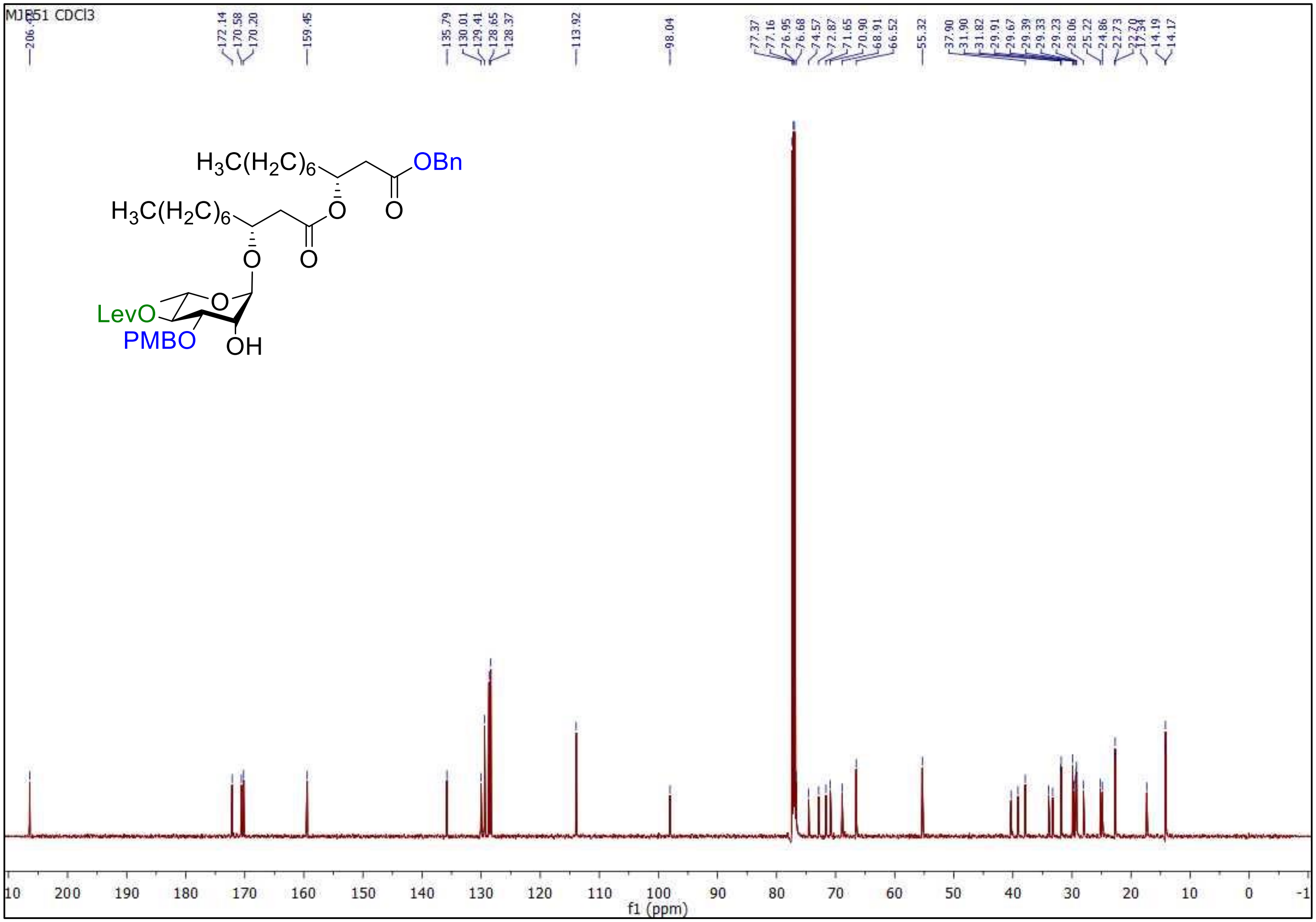

Figure S99| HSQC NMR spectrum ($CDCl_3$, 600 MHz) of benzyl (*R*)-3-*O*-[(*R*)-(3'-*O*-decyl)-4-*O*-levulinoyl-3-*O*-*para*-methoxybenzyl-*α*-L-rhamnopyranosyl]decanoate (**S14**).

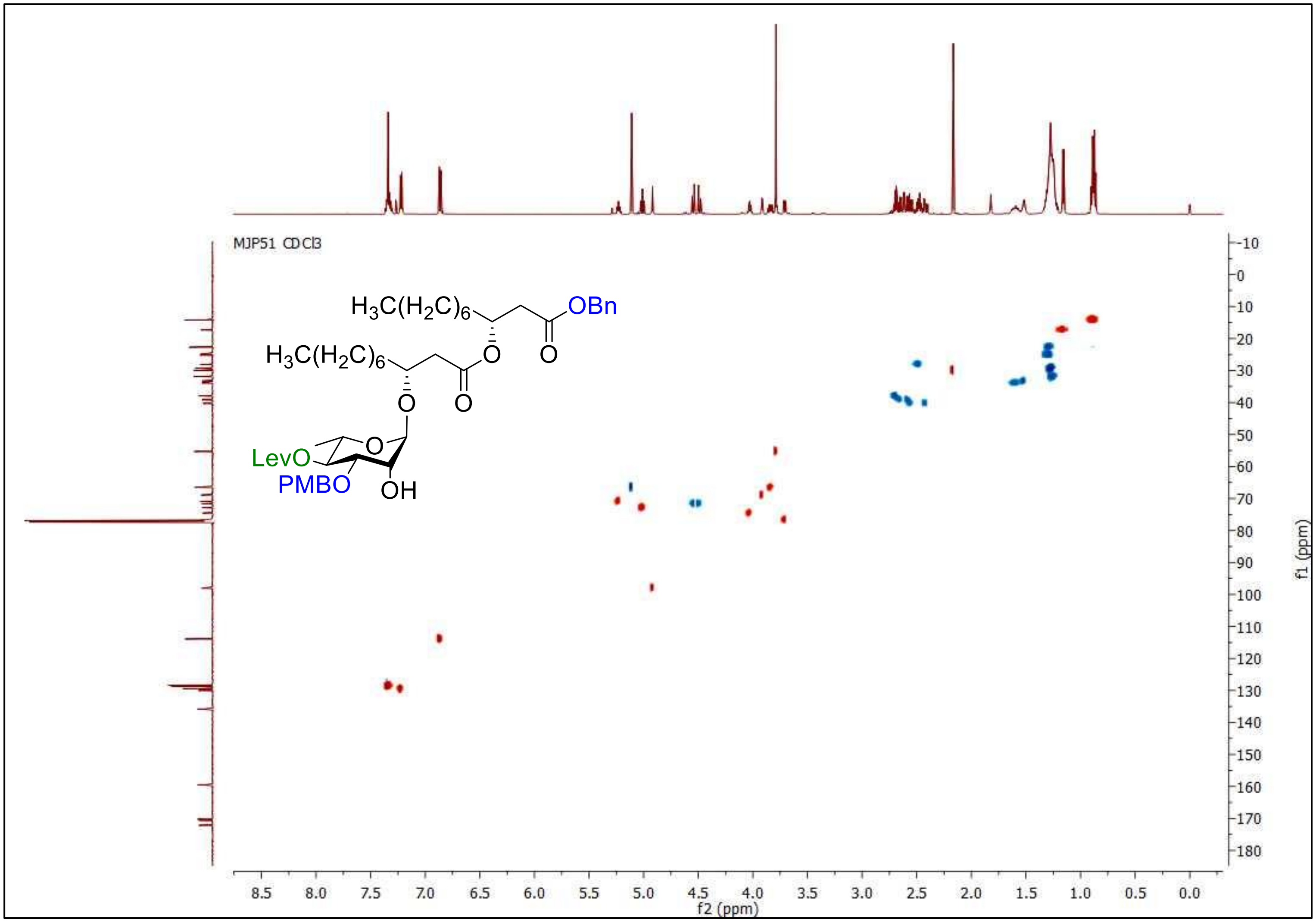

Figure S100 | ^{1}H NMR spectrum ($CDCl_3$, 600 MHz) of benzyl (*R*)-3-*O*-[(*R*)-(3'-*O*-decyl)-3-*O*-*para*-methoxybenzyl-*α*-L-rhamnopyranosyl]decanoate (**33**).

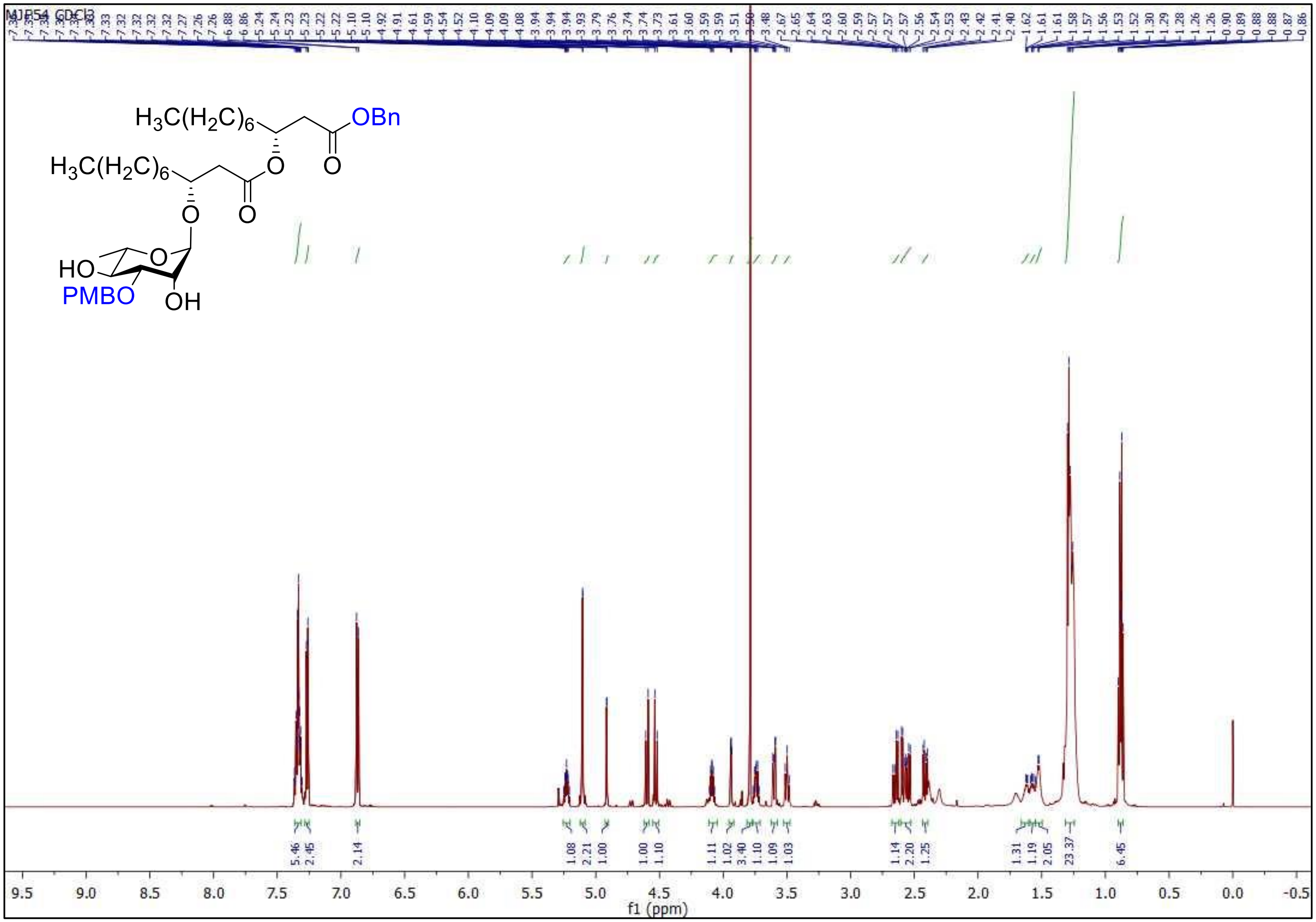

Figure S101 | COSY NMR spectrum ($CDCl_3$, 600 MHz) of benzyl (*R*)-3-*O*-[(*R*)-(3'-*O*-decyl)-3-*O*-*para*-methoxybenzyl-*α*-L-rhamnopyranosyl]decanoate (**33**).

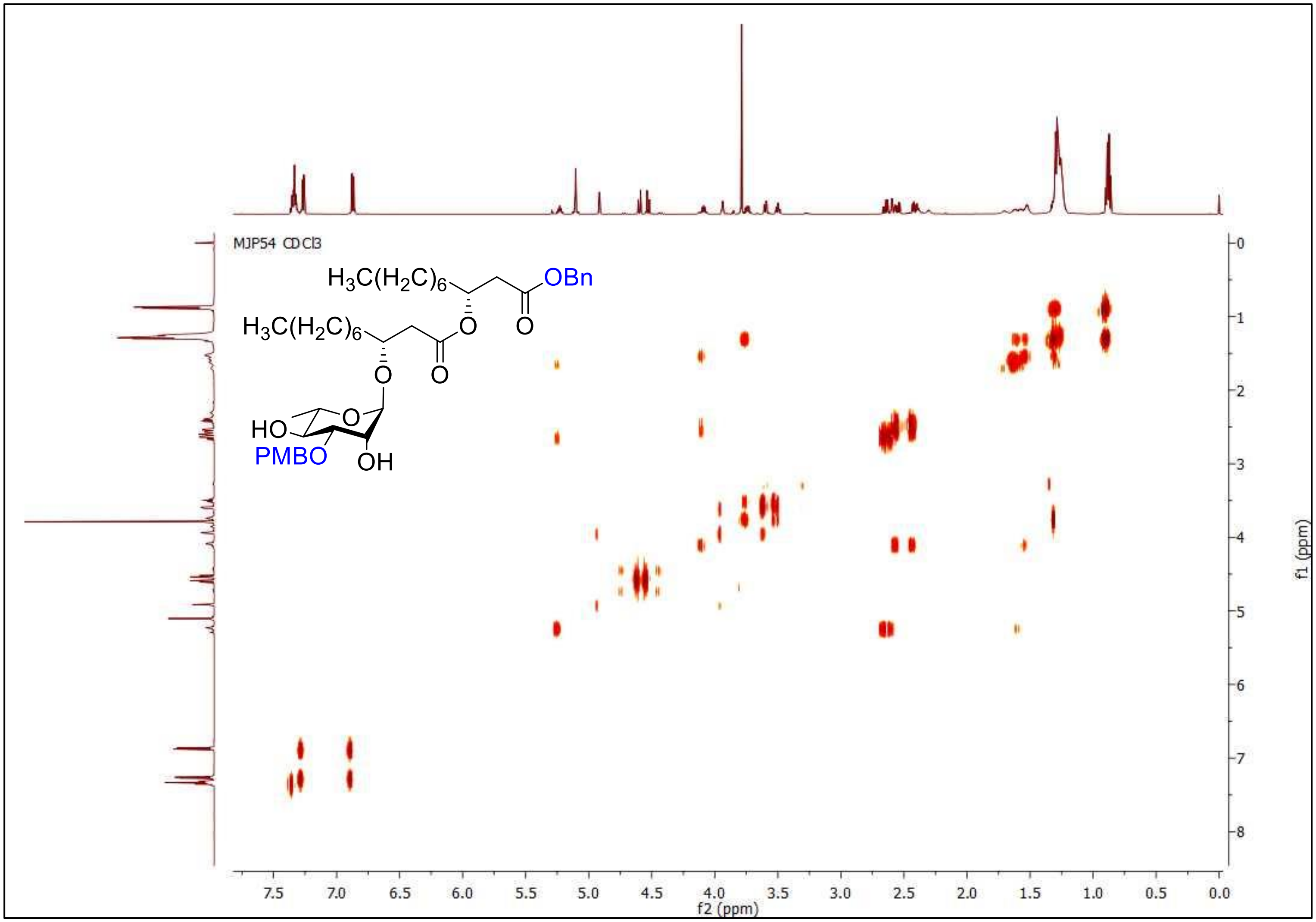

Figure S102 | ^{13}C NMR spectrum ($CDCl_3$, 600 MHz) of benzyl (*R*)-3-*O*-[(*R*)-(3'-*O*-decyl)-3-*O*-*para*-methoxybenzyl-*α*-L-rhamnopyranosyl]decanoate (**33**).

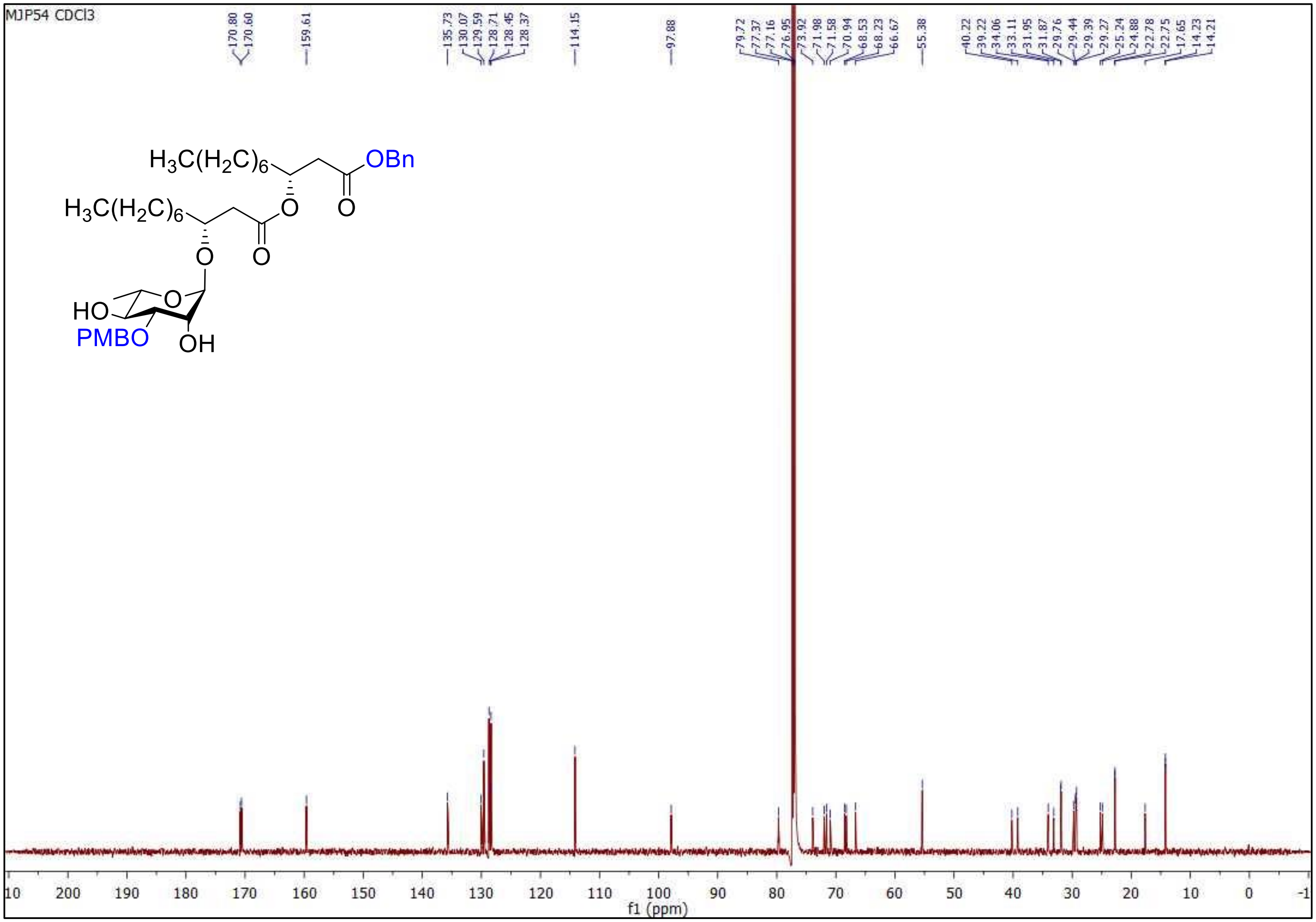

Figure S103 | HSQC NMR spectrum ($CDCl_3$, 600 MHz) of benzyl (*R*)-3-*O*-[(*R*)-(3'-*O*-decyl)-3-*O*-*para*-methoxybenzyl-*α*-L-rhamnopyranosyl]decanoate (**33**).

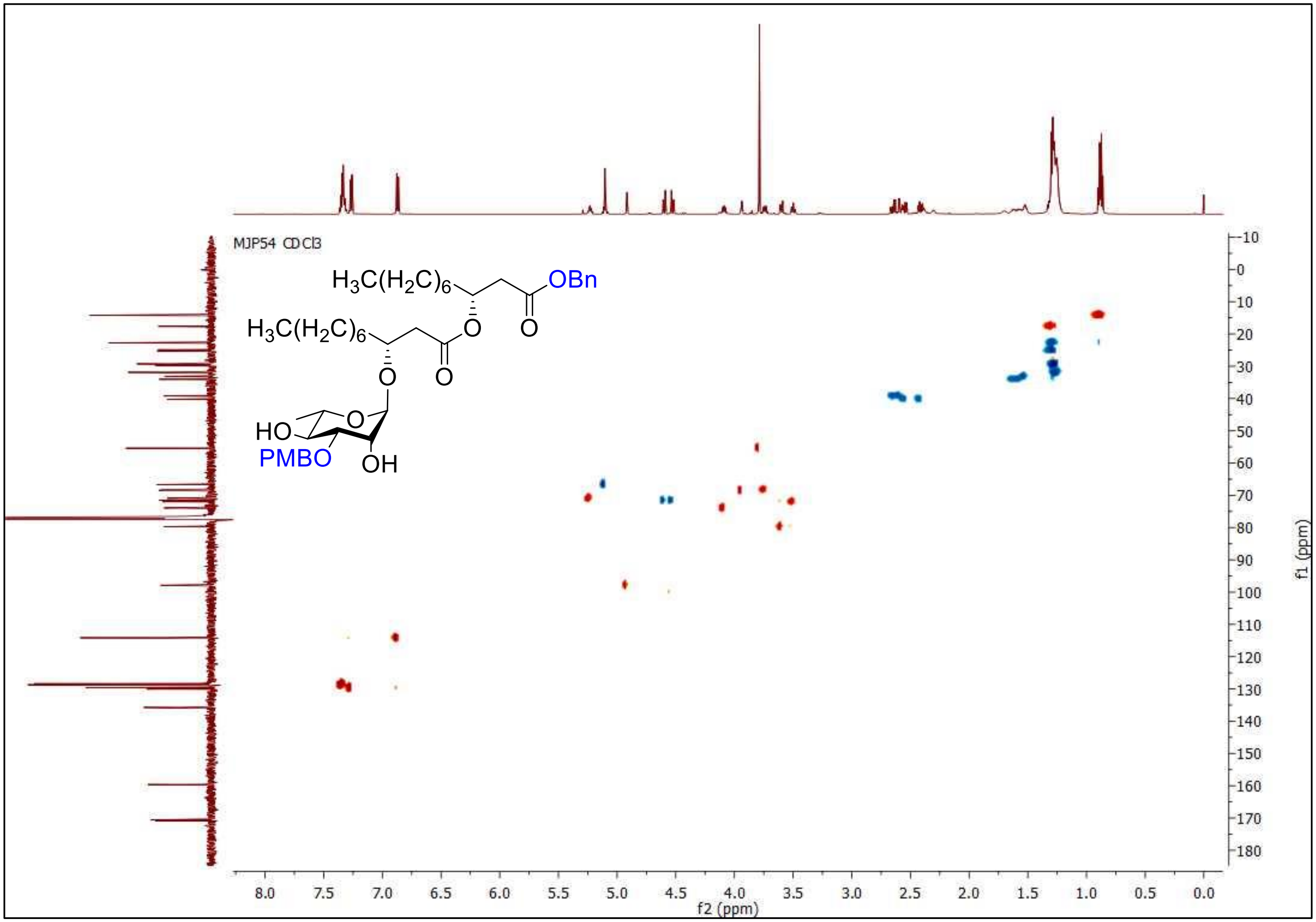

Figure S104 | ^{1}H NMR spectrum ($CDCl_3$/CD_3OD, 600 MHz) of (*R*)-3-*O*-[(*R*)-(3'-*O*-decyl)-*α*-L-rhamnopyranosyl]decanoic acid (**3**).

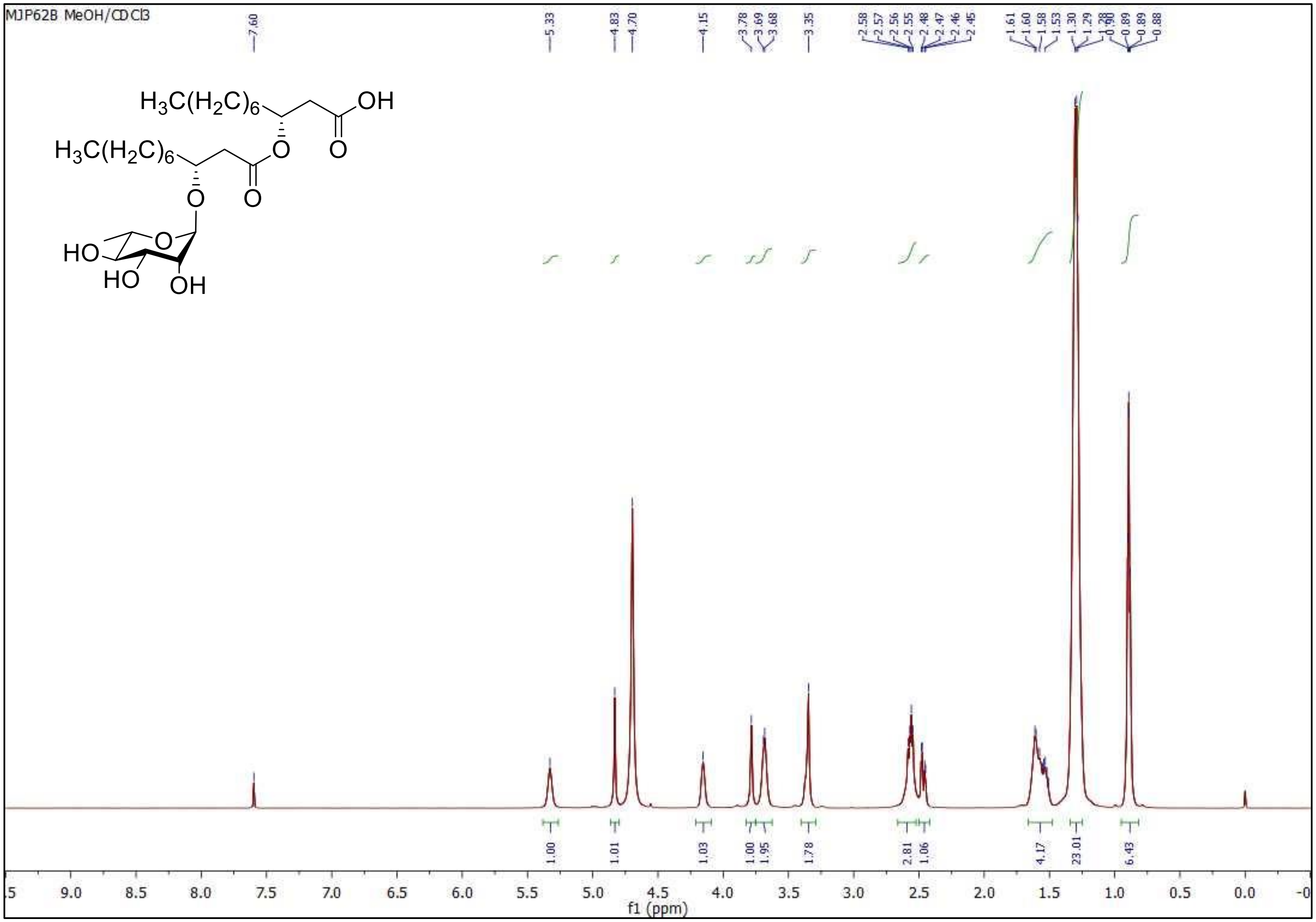

Figure S105 | ^{1}H NMR spectrum ($CDCl_3$, 600 MHz) of *para*-methylphenyl 2-*O*-*ortho*-(azidomethyl)benzoyl-3,4-*O*-(2,3-dimethoxybutan-2,3-diyl)-1-thio-α-L-rhamnopyranoside (**S7**).

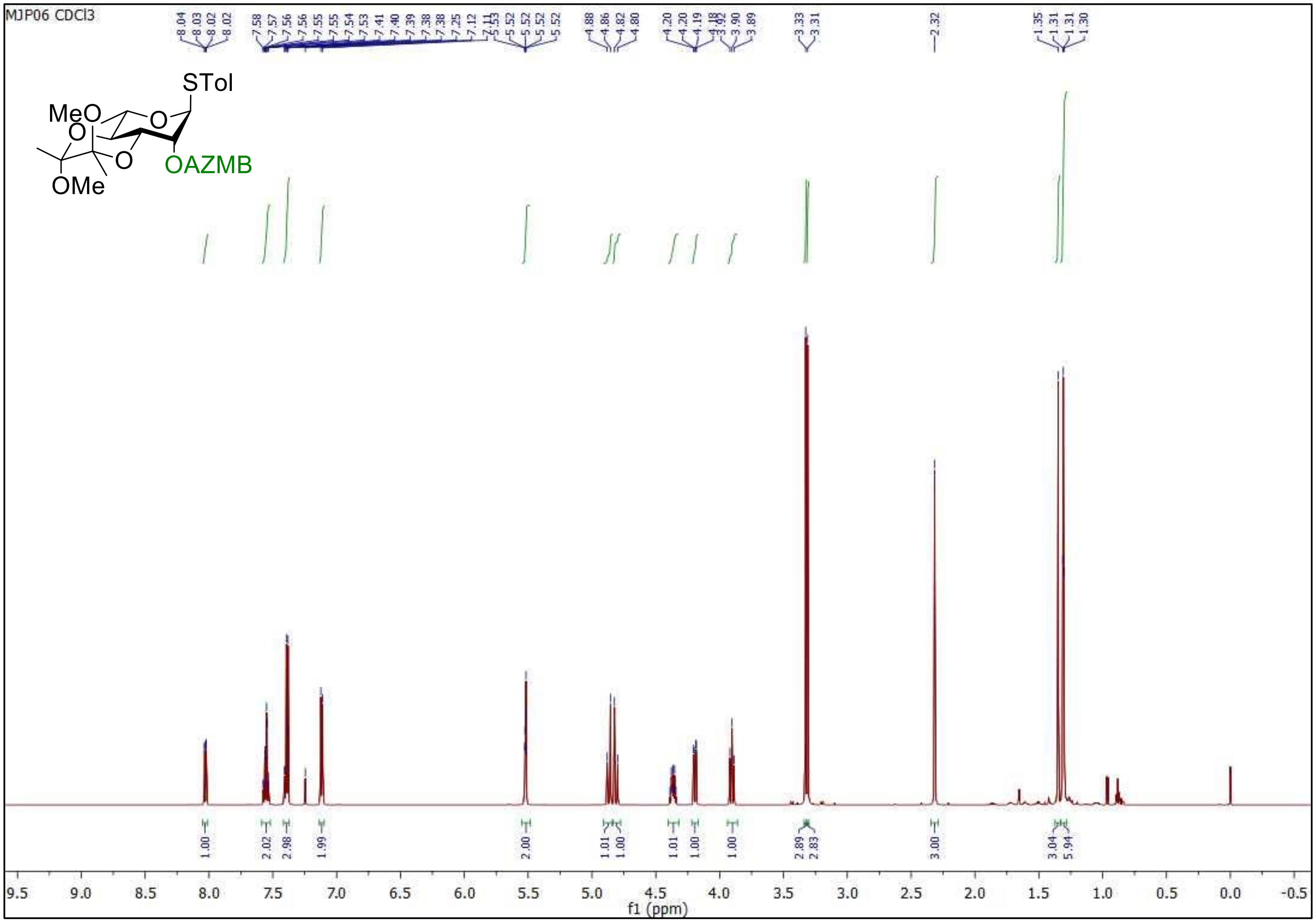

Figure S106 | COSY NMR spectrum ($CDCl_3$, 600 MHz) of *para*-methylphenyl 2-*O*-*ortho*-(azidomethyl)benzoyl-3,4-*O*-(2,3-dimethoxybutan-2,3-diyl)-1-thio-α-L-rhamnopyranoside (**S7**).

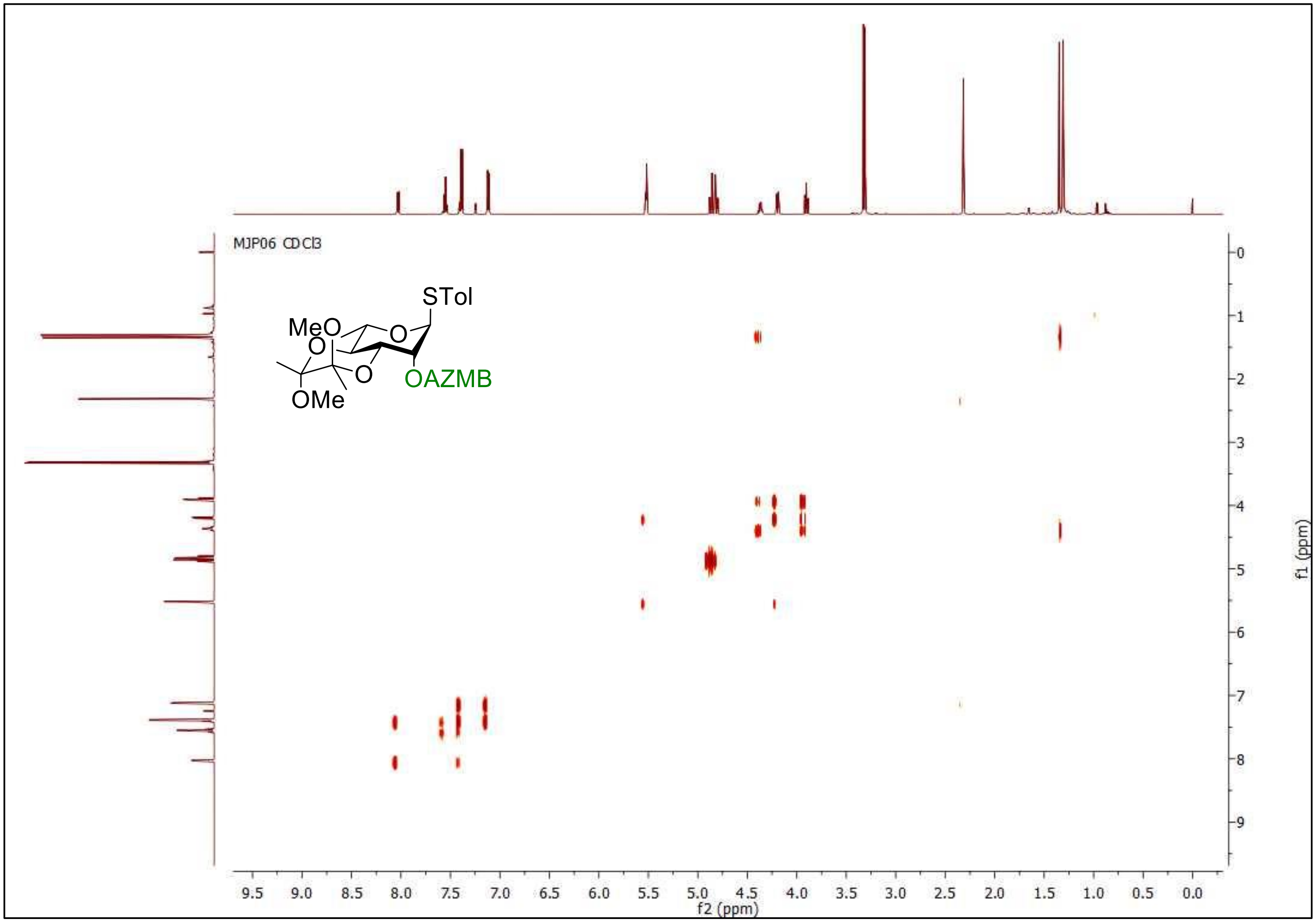

Figure S107 | ^{13}C NMR spectrum ($CDCl_3$, 600 MHz) of *para*-methylphenyl 2-*O*-*ortho*-(azidomethyl)benzoyl-3,4-*O*-(2,3-dimethoxybutan-2,3-diyl)-1-thio-α-L-rhamnopyranoside (**S7**).

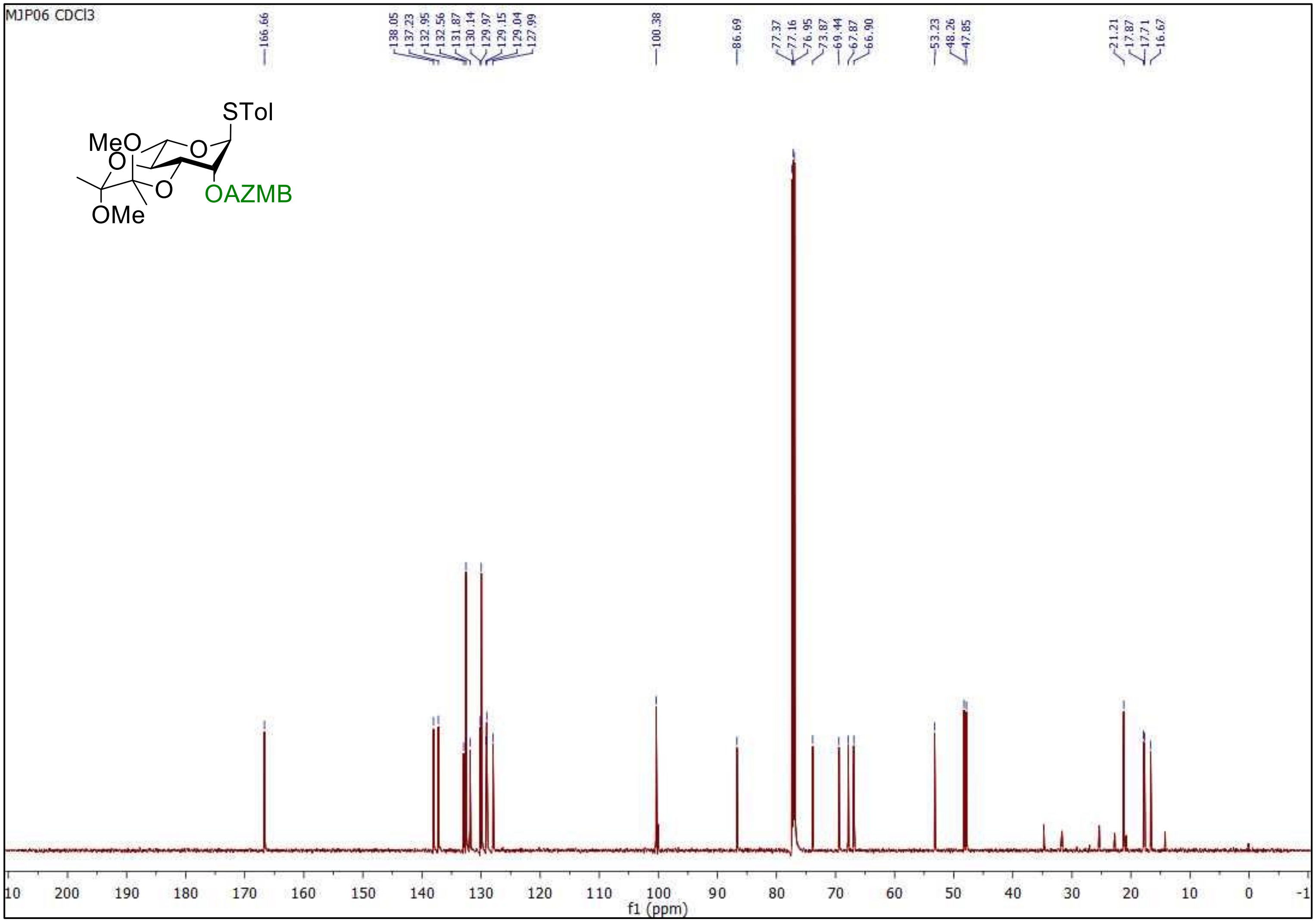

Figure S108 | HSQC NMR spectrum (CDCl$_3$, 600 MHz) of *para*-methylphenyl 2-*O*-*ortho*-(azidomethyl)benzoyl-3,4-*O*-(2,3-dimethoxybutan-2,3-diyl)-1-thio-α-L-rhamnopyranoside (**S7**).

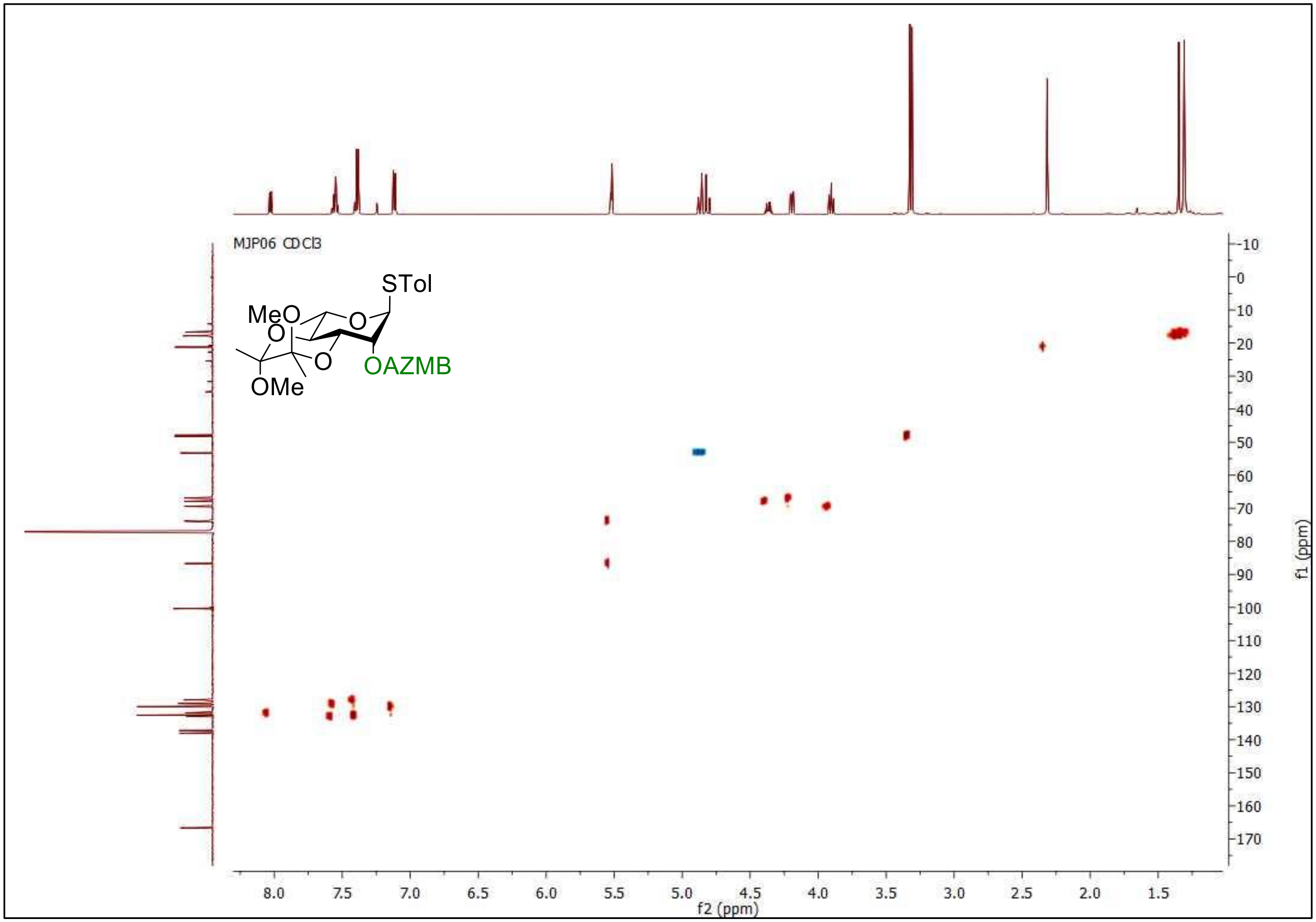

Figure S109 | ^{1}H NMR spectrum (CDCl$_3$, 600 MHz) of benzyl (*R*)-3-*O*-[(*R*)-(3'-*O*-decyl)-2-*O*-*ortho*-(azidomethyl)benzoyl-[3,4-*O*-(2,3-dimethoxybutan-2,3-diyl)]-*α*-L-rhamnopyranosyl]decanoate (**S8**).

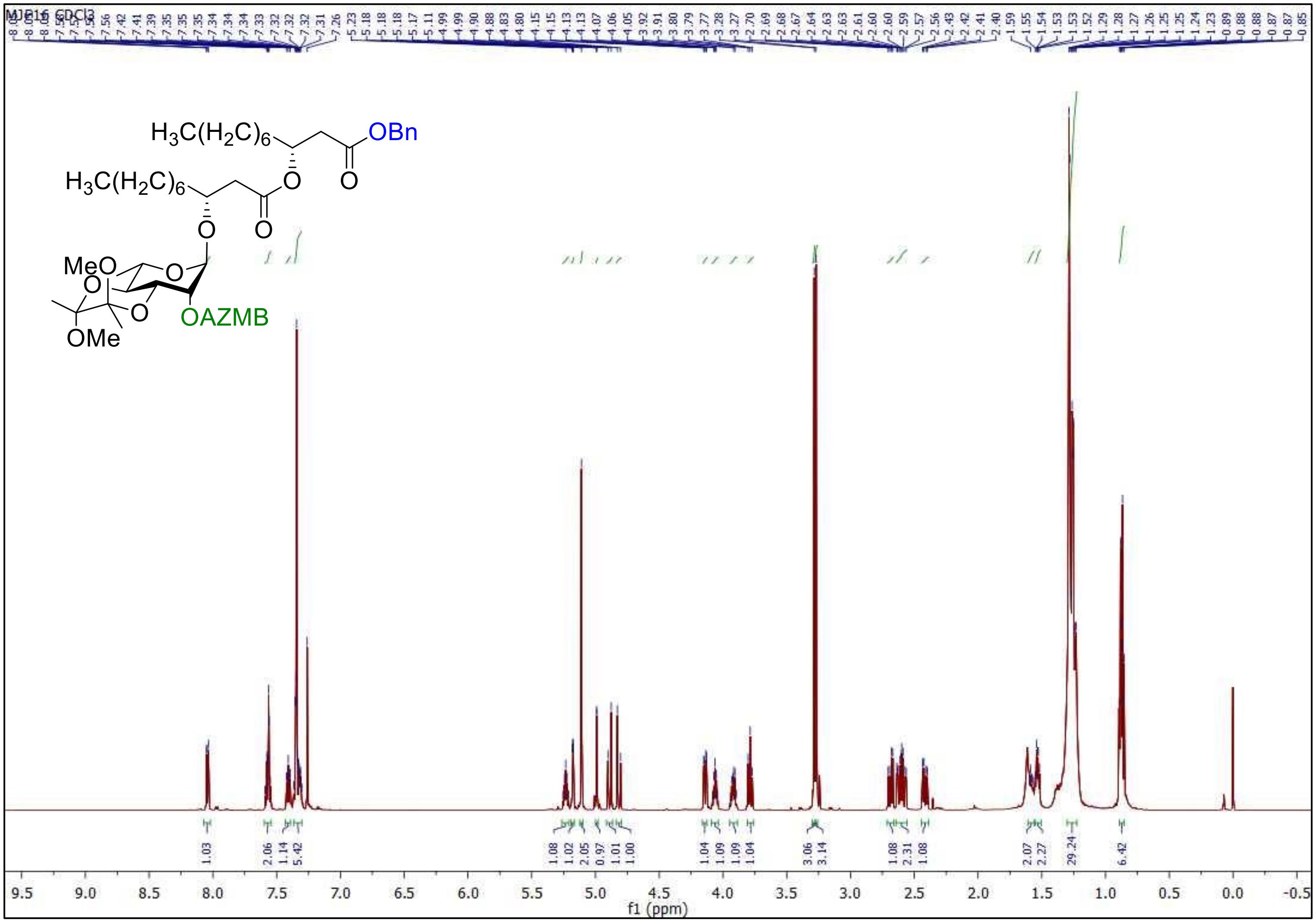

Figure S110 | COSY NMR spectrum ($CDCl_3$, 600 MHz) of benzyl (*R*)-3-*O*-[(*R*)-(3'-*O*-decyl)-2-*O*-*ortho*-(azidomethyl)benzoyl-[3,4-*O*-(2,3-dimethoxybutan-2,3-diyl)]-*α*-L-rhamnopyranosyl]decanoate (**S8**).

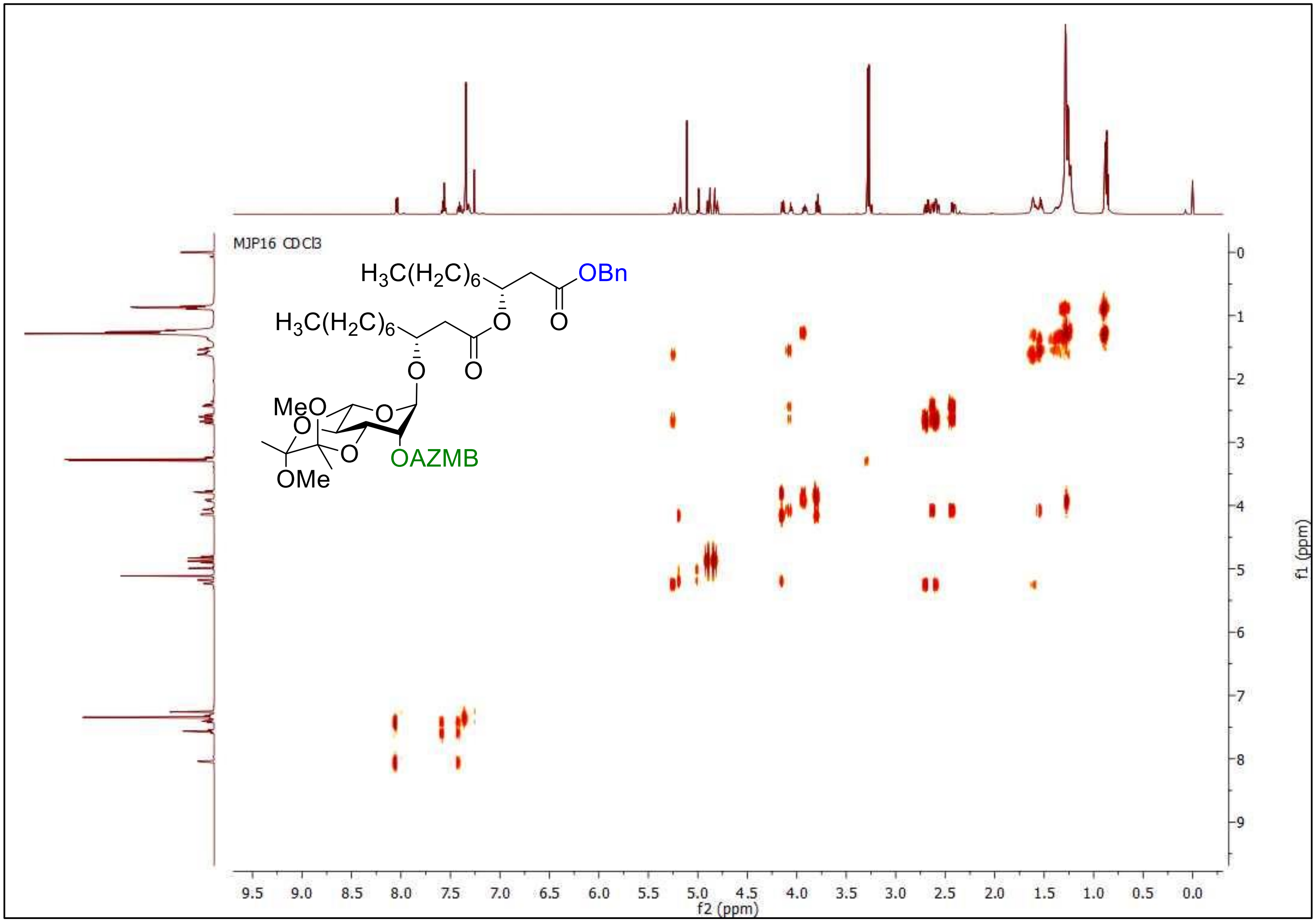

Figure S111 | ^{13}C NMR spectrum ($CDCl_3$, 600 MHz) of benzyl (*R*)-3-*O*-[(*R*)-(3'-*O*-decyl)-2-*O*-*ortho*-(azidomethyl)benzoyl-[3,4-*O*-(2,3-dimethoxybutan-2,3-diyl)]-*α*-L-rhamnopyranosyl]decanoate (**S8**).

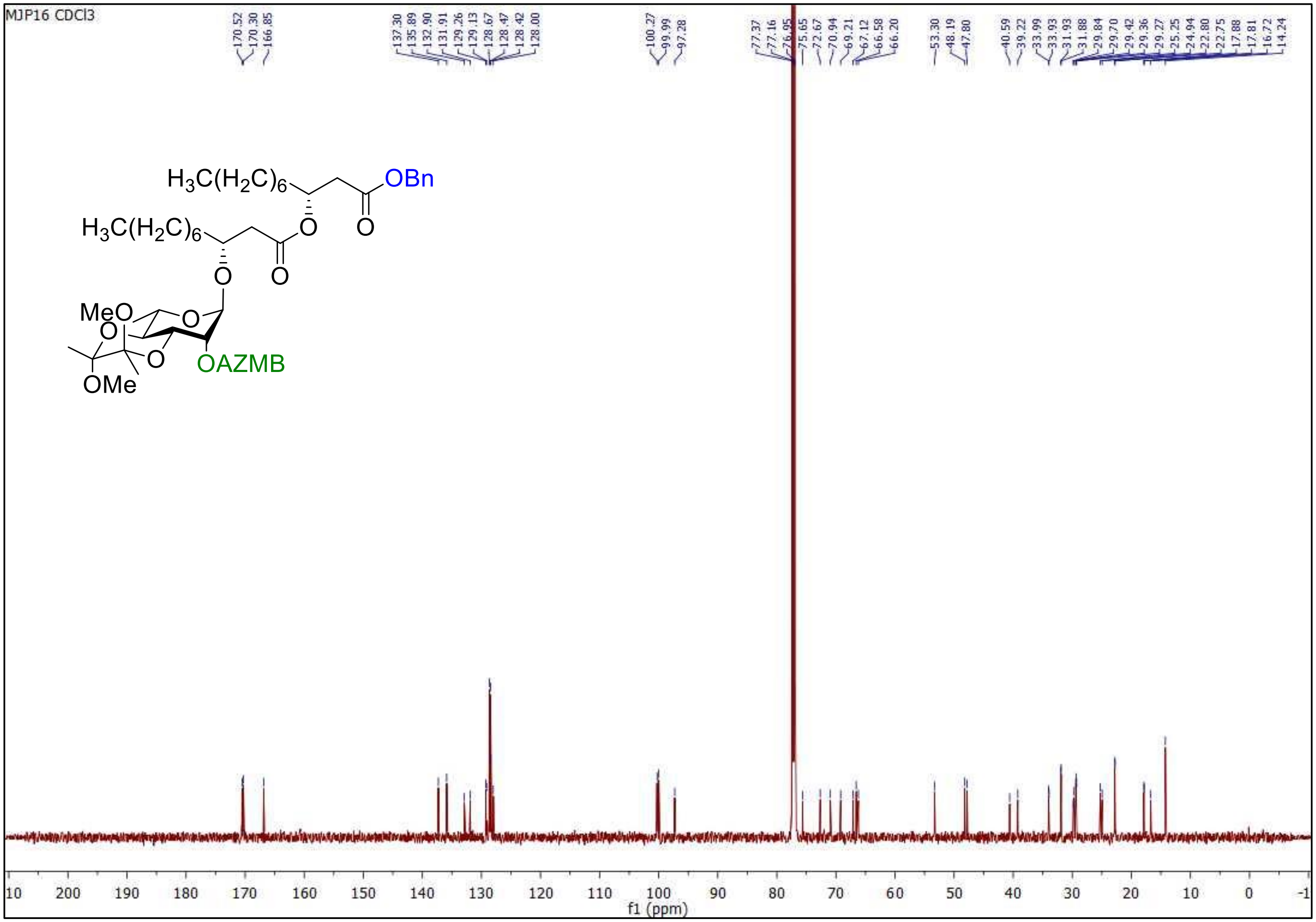

Figure S112 | HSQC NMR spectrum ($CDCl_3$, 600 MHz) of benzyl (*R*)-3-*O*-[(*R*)-(3'-*O*-decyl)-2-*O*-*ortho*-(azidomethyl)benzoyl-[3,4-*O*-(2,3-dimethoxybutan-2,3-diyl)]-*α*-L-rhamnopyranosyl]decanoate (**S8**).

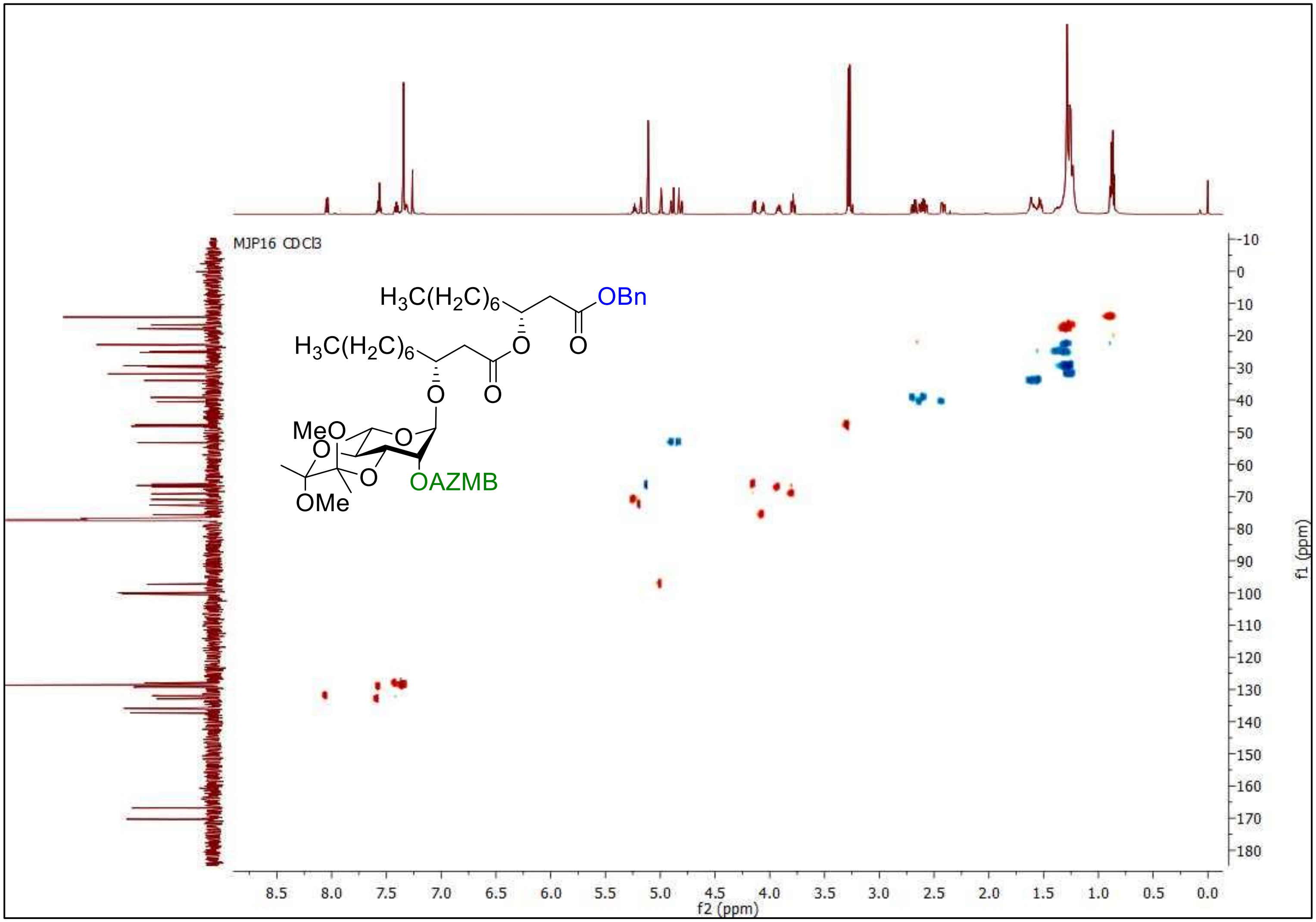

Figure S113 | ^{1}H NMR spectrum ($CDCl_3$, 600 MHz) of benzyl (*R*)-3-*O*-[(*R*)-(3'-*O*-decyl)-*α*-L-rhamnopyranosyl]decanoate (**S9**).

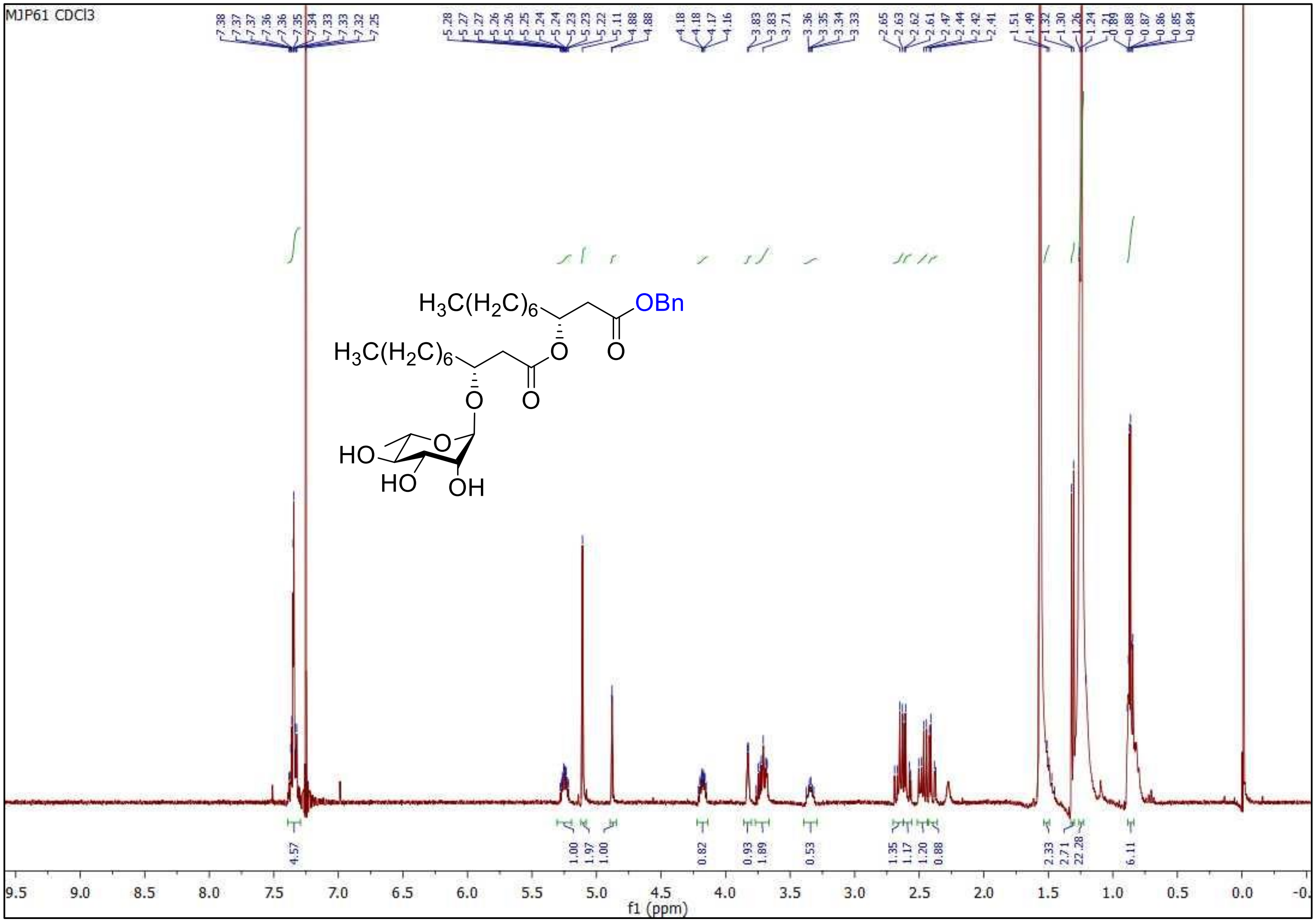

Figure S114 | COSY NMR spectrum ($CDCl_3$, 600 MHz) of benzyl (*R*)-3-*O*-[(*R*)-(3'-*O*-decyl)-*α*-L-rhamnopyranosyl]decanoate (**S9**).

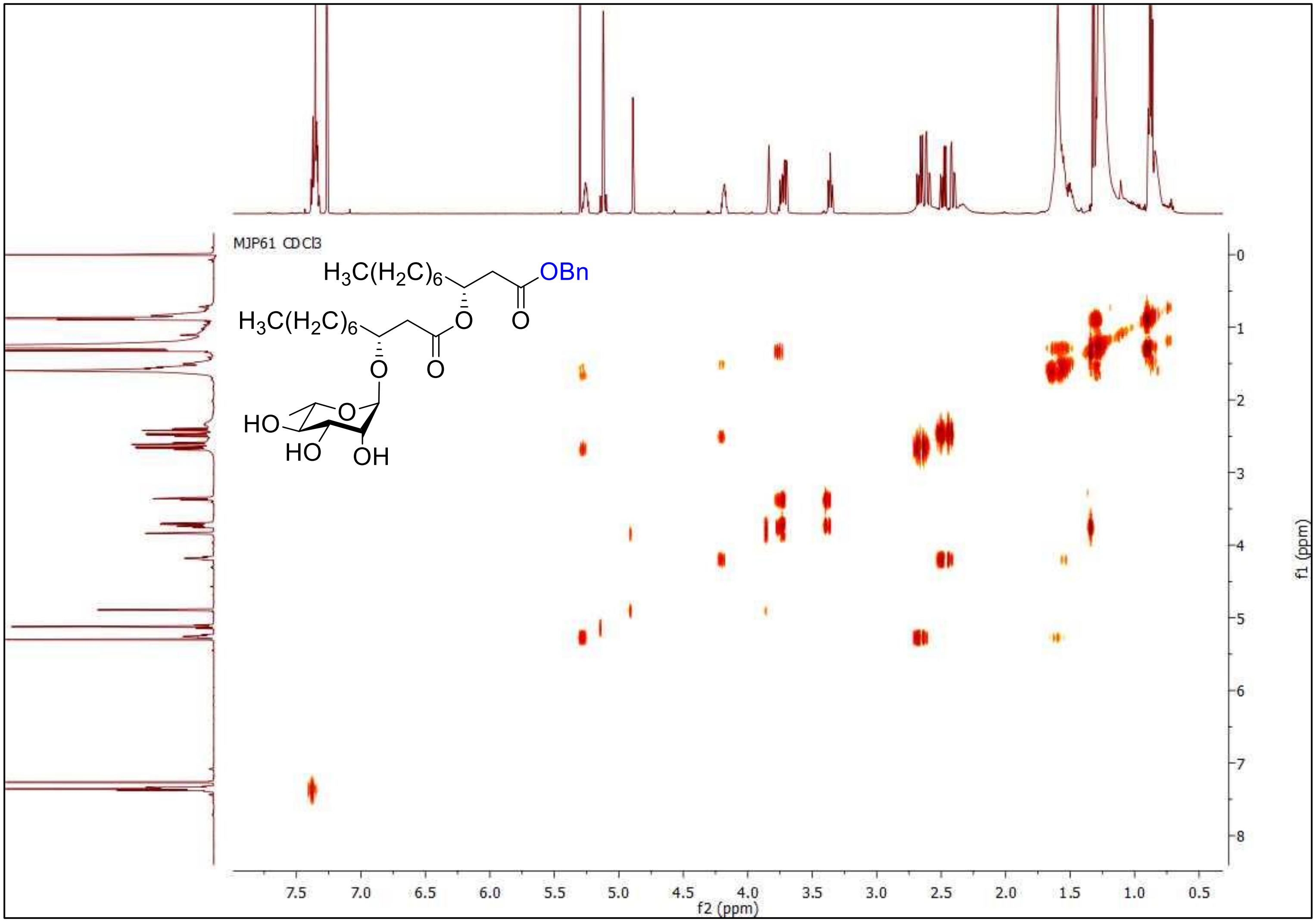

Figure S115 | ^{13}C NMR spectrum ($CDCl_3$, 600 MHz) of benzyl (*R*)-3-*O*-[(*R*)-(3'-*O*-decyl)-*α*-L-rhamnopyranosyl]decanoate (**S9**)

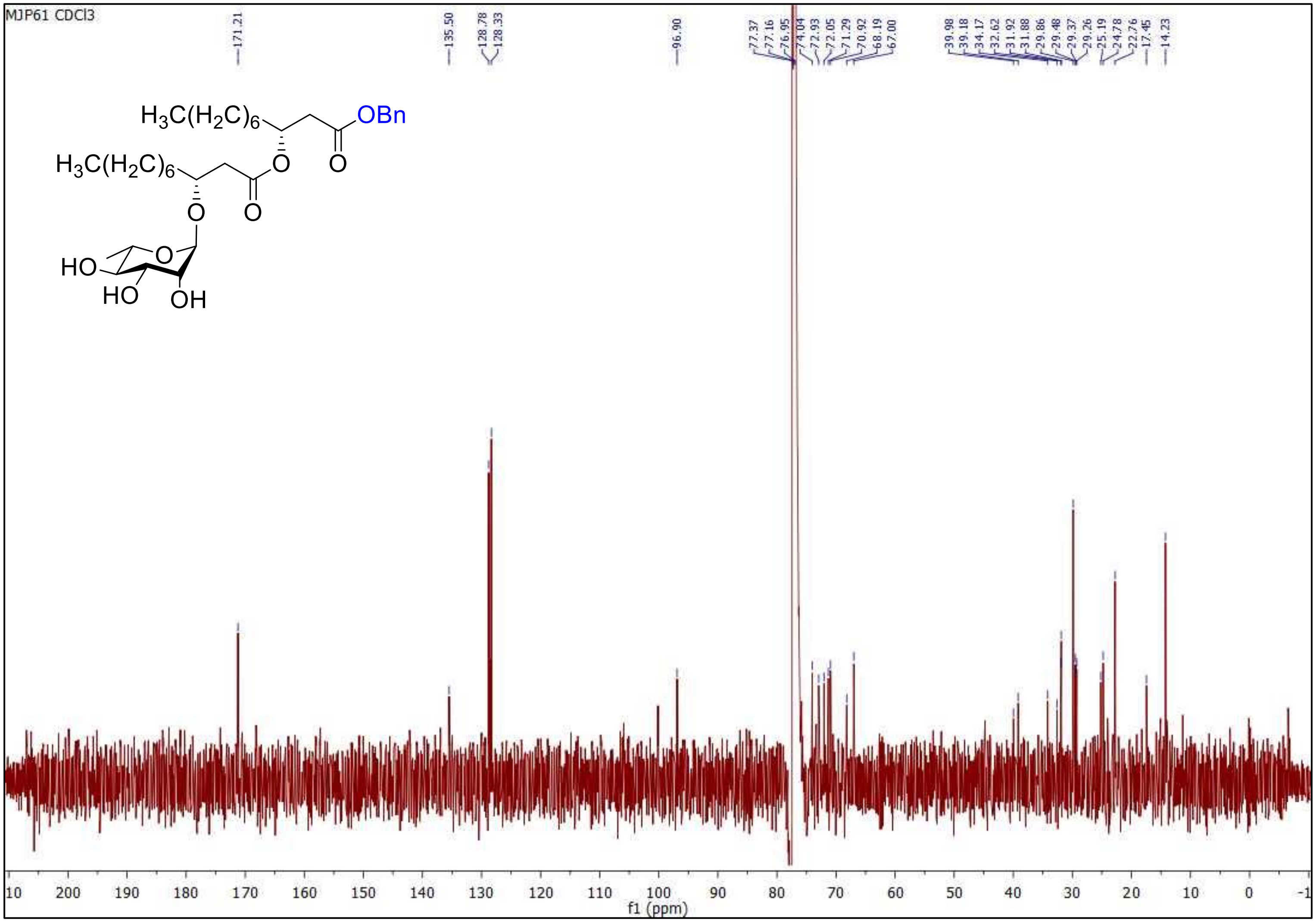

Figure S116 | HSQC NMR spectrum ($CDCl_3$, 600 MHz) of benzyl (*R*)-3-*O*-[(*R*)-(3'-*O*-decyl)-*α*-L-rhamnopyranosyl]decanoate (**S9**).

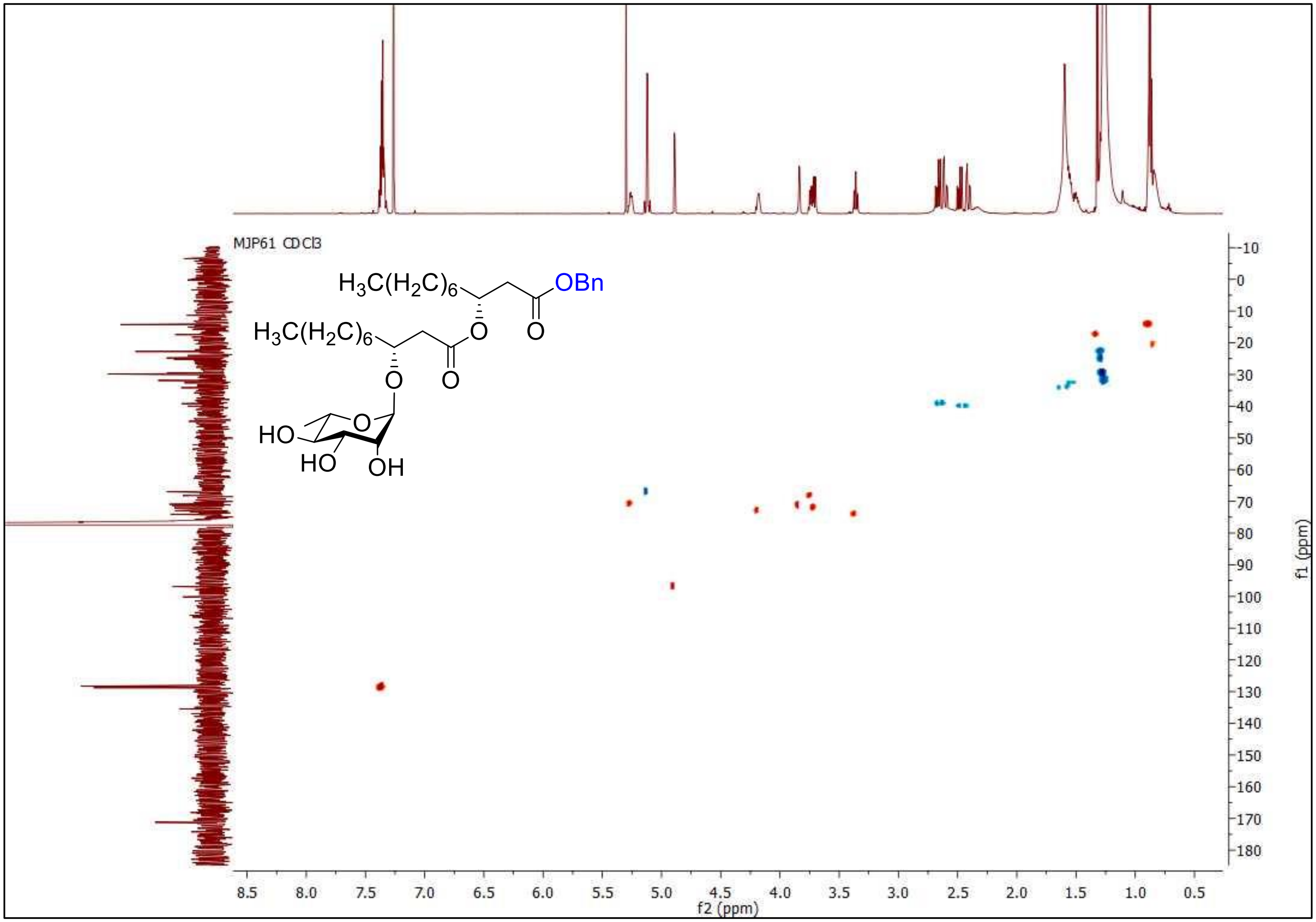

Figure S117 | ^{1}H NMR spectrum ($CDCl_3$, 600 MHz) of *para*-methylphenyl 3-*O*-benzyl-4-*O*-levulinoyl-1-thio-*α*-L-rhamnopyranoside (**30**).

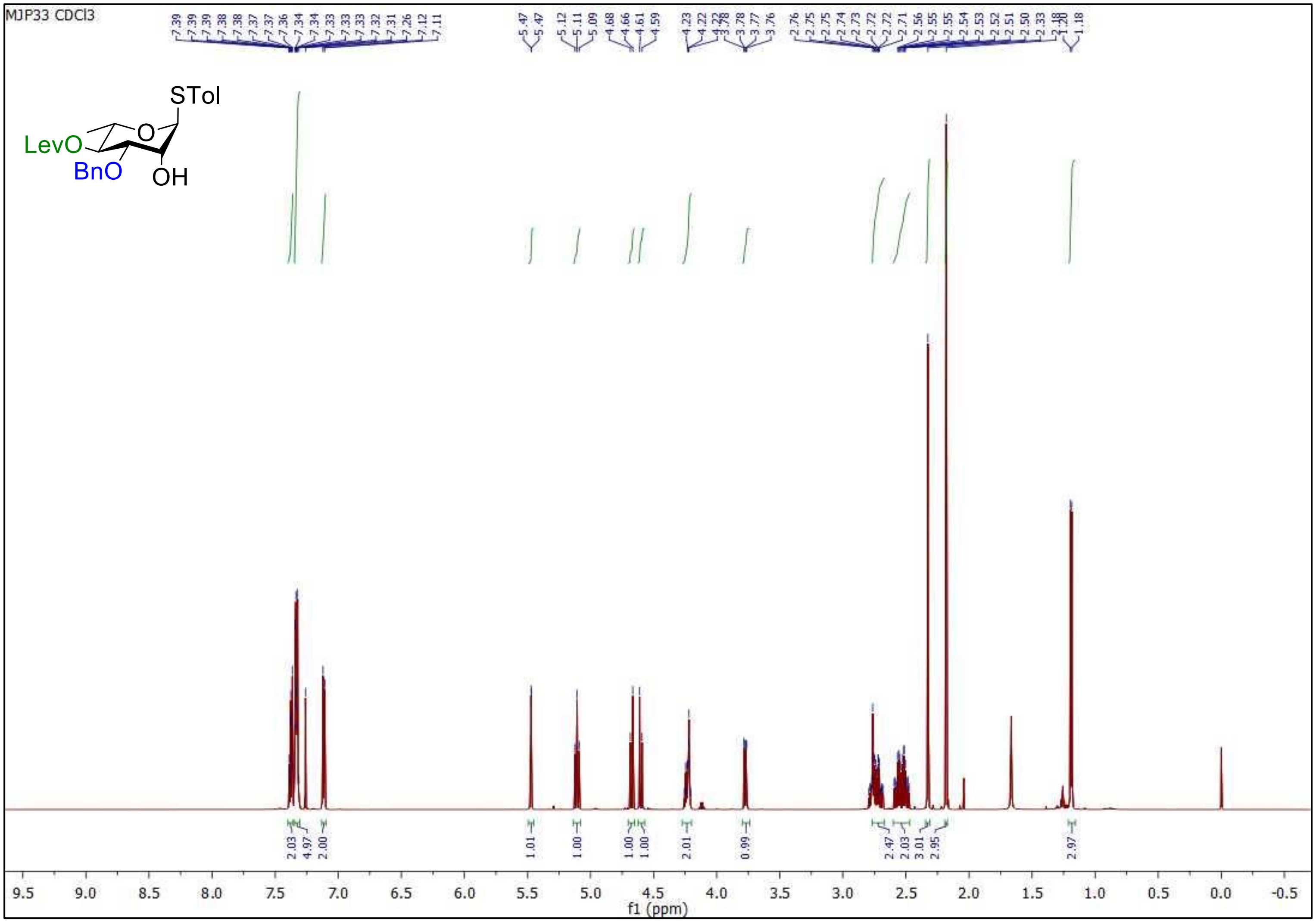

Figure S118 | COSY NMR spectrum ($CDCl_3$, 600 MHz) of *para*-methylphenyl 3-*O*-benzyl-4-*O*-levulinoyl-1-thio-*α*-L-rhamnopyranoside (**30**).

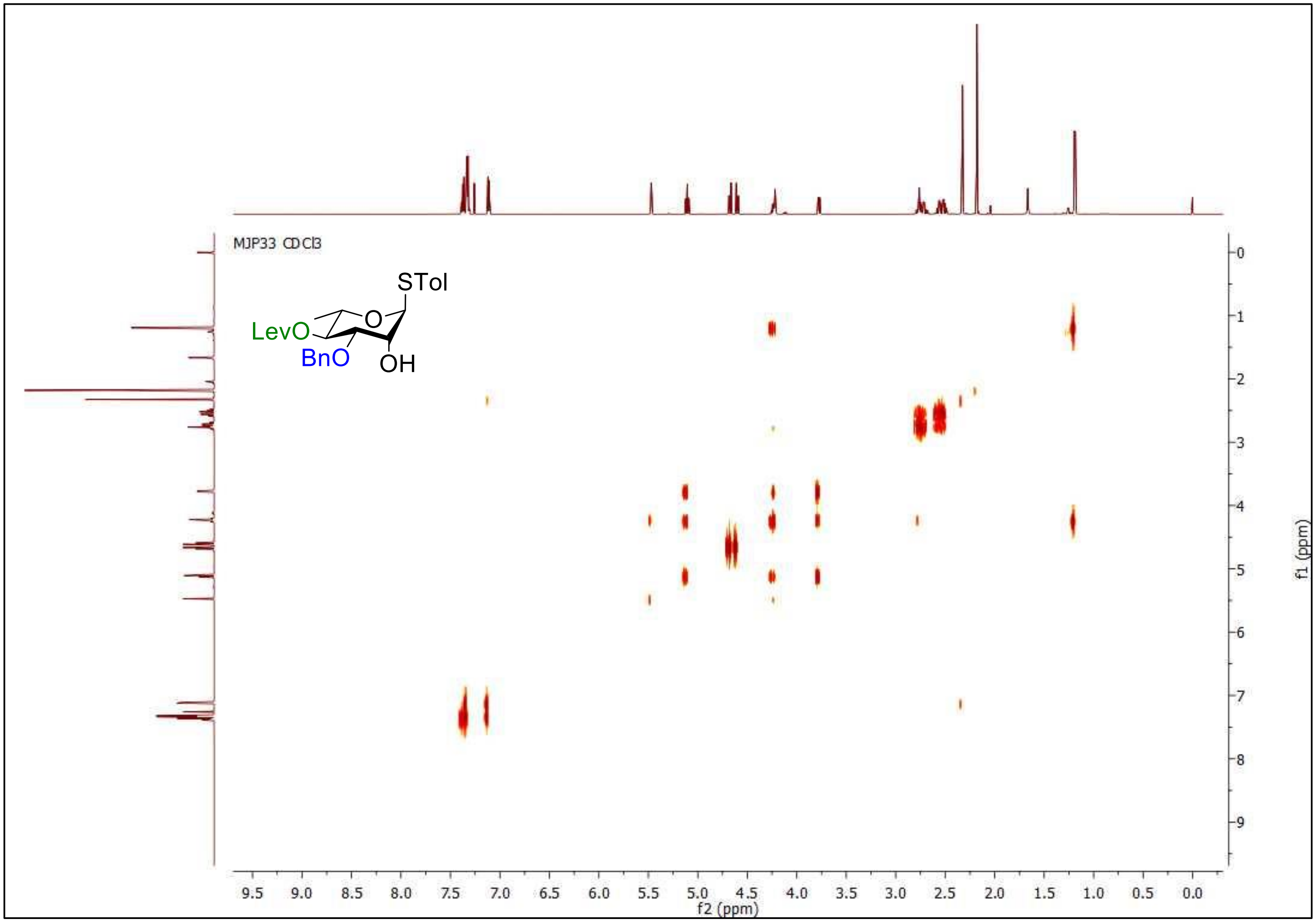

Figure S119 | ^{13}C NMR spectrum ($CDCl_3$, 600 MHz) of *para*-methylphenyl 3-*O*-benzyl-4-*O*-levulinoyl-1-thio-*α*-L-rhamnopyranoside (**30**).

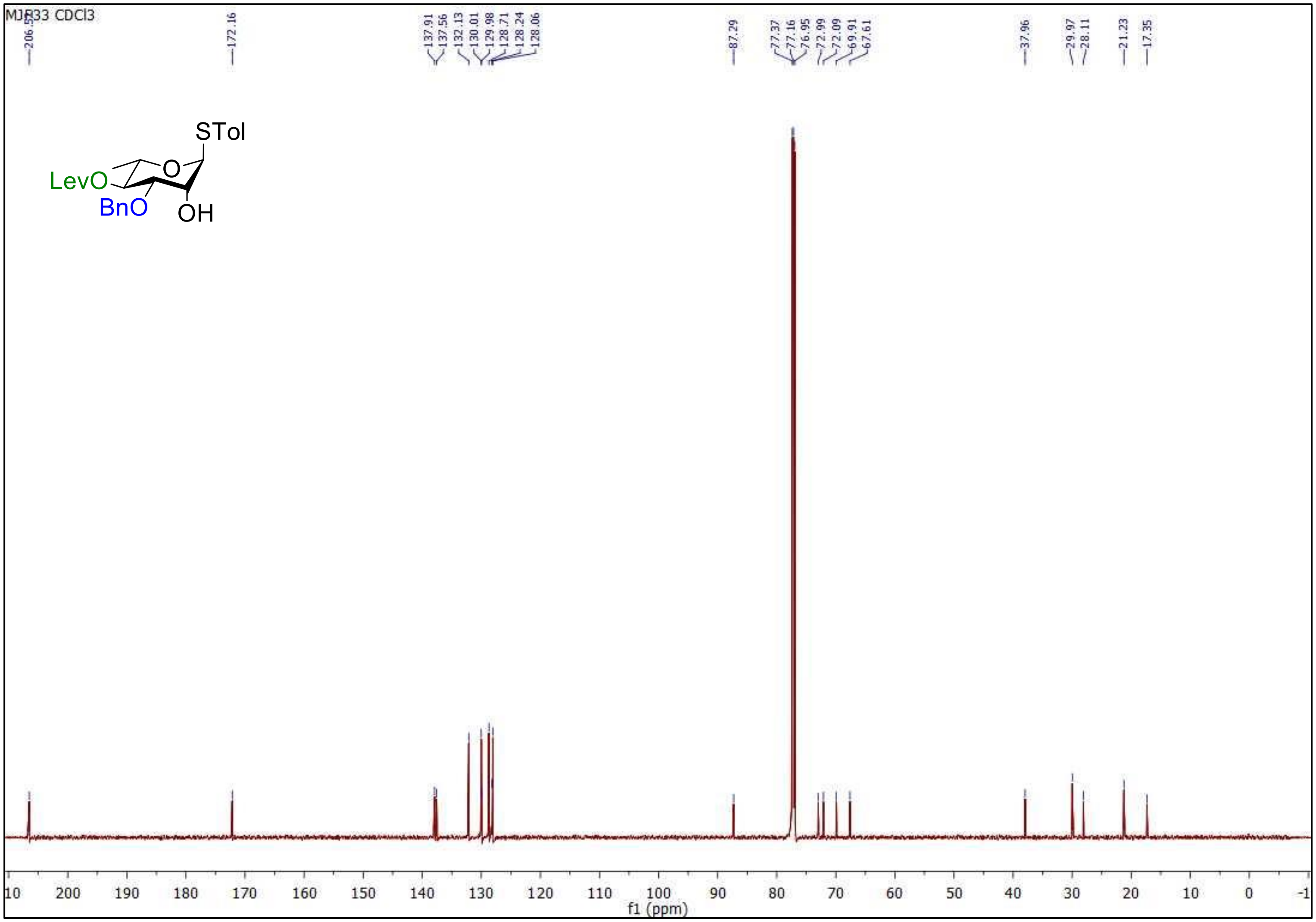

Figure S120 | HSQC NMR spectrum ($CDCl_3$, 600 MHz) of *para*-methylphenyl 3-*O*-benzyl-4-*O*-levulinoyl-1-thio-*α*-L-rhamnopyranoside (**30**).

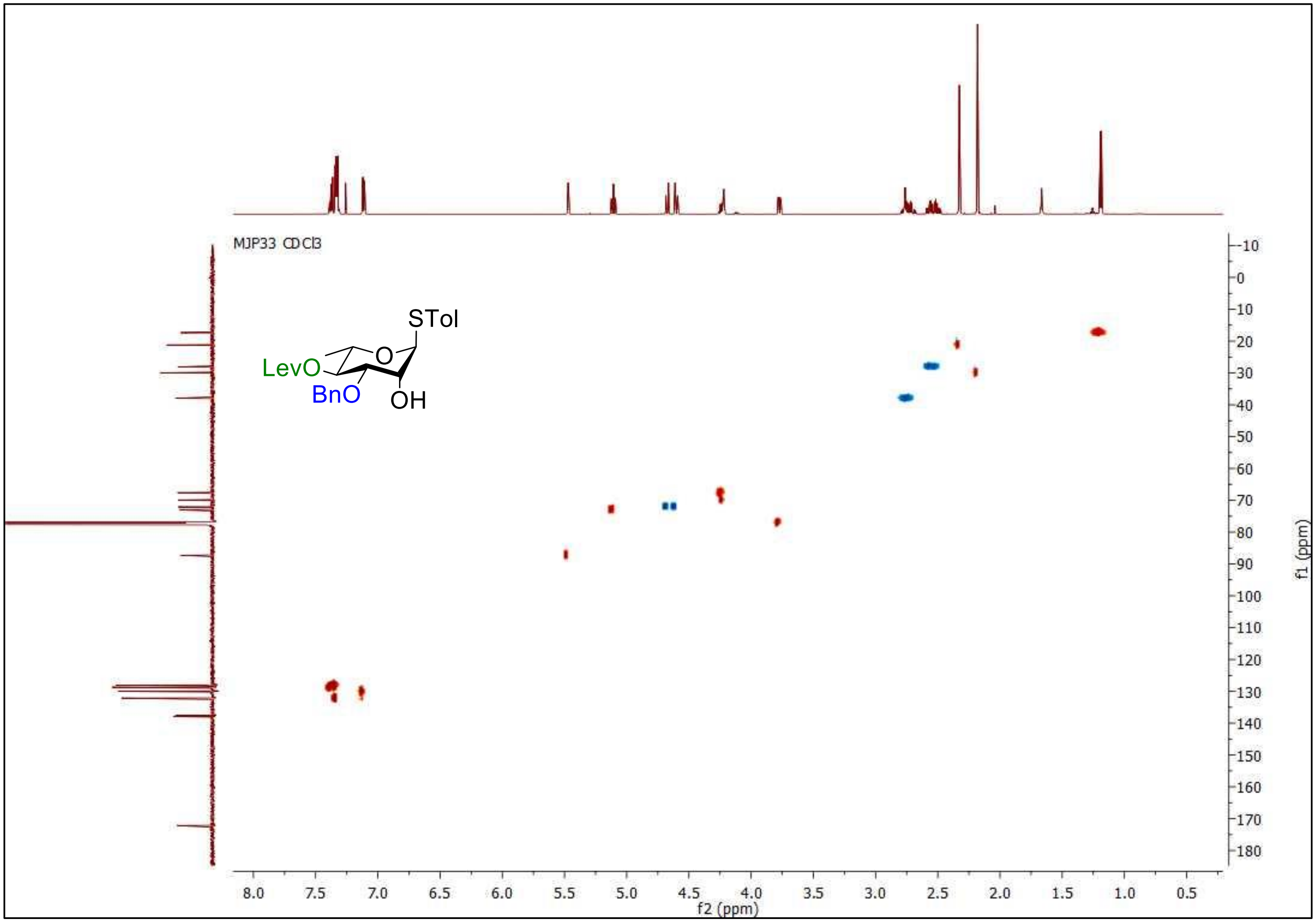

Figure S121 | ^{1}H NMR spectrum ($CDCl_3$, 600 MHz) of *para*-methylphenyl 2-*O*-*ortho*-(azidomethyl)benzoyl-3-*O*-benzyl-4-*O*-levulinoyl-1-thio-*α*-L-rhamnopyranoside (**S15**).

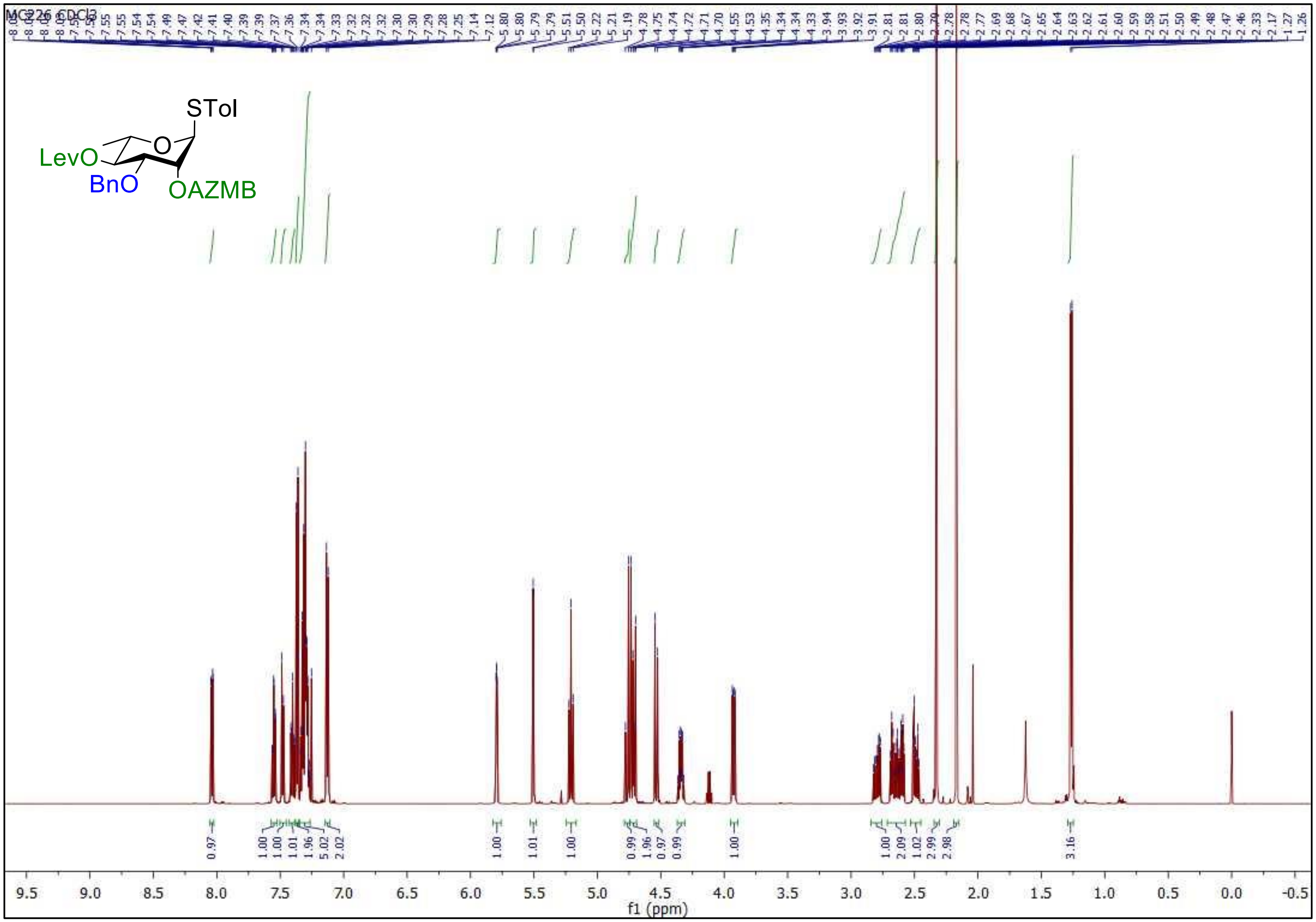

Figure S122 | COSY NMR spectrum ($CDCl_3$, 600 MHz) of *para*-methylphenyl 2-*O*-*ortho*-(azidomethyl)benzoyl-3-*O*-benzyl-4-*O*-levulinoyl-1-thio-*α*-L-rhamnopyranoside (**S15**).

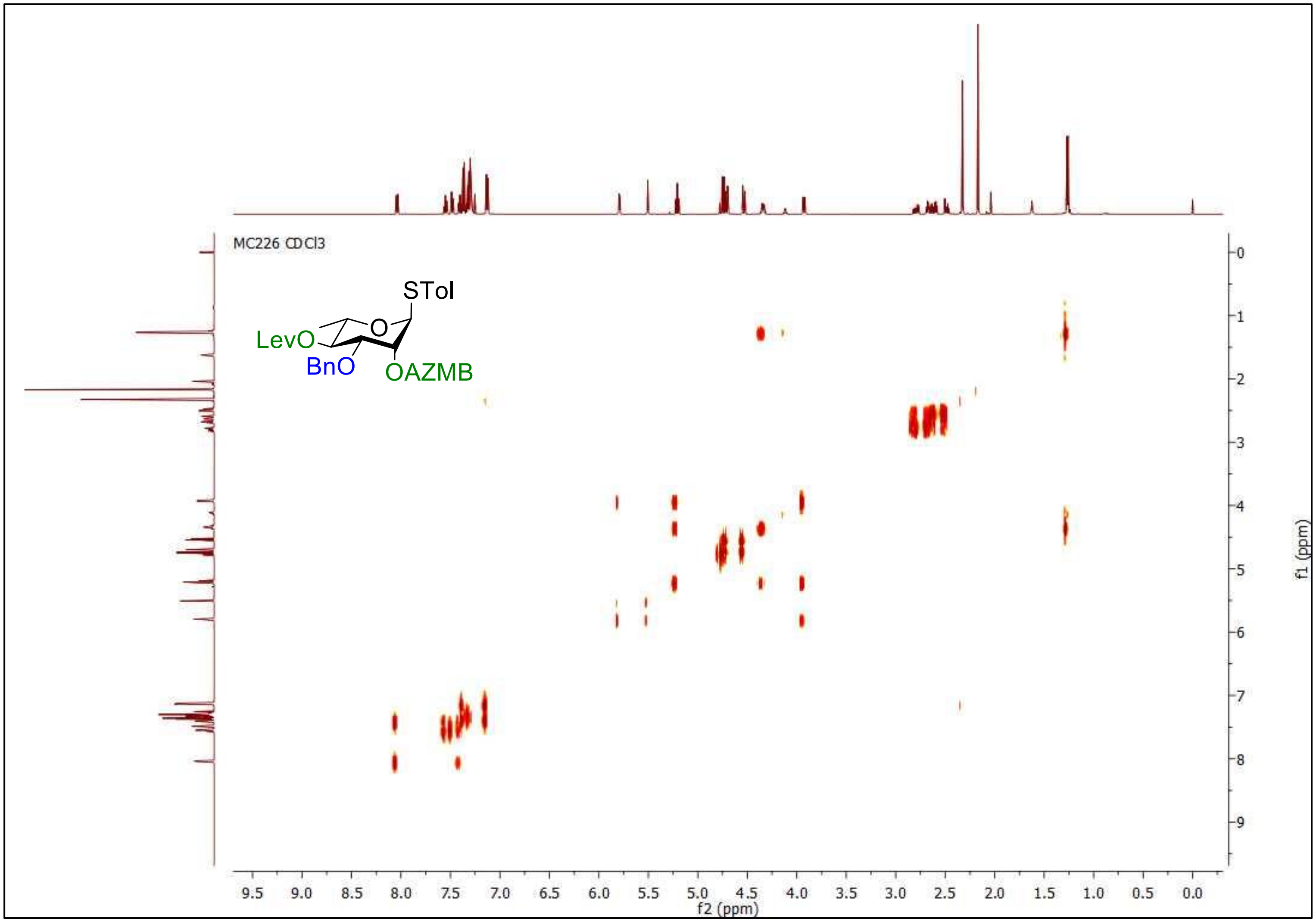

Figure S123 | ^{13}C NMR spectrum ($CDCl_3$, 600 MHz) of *para*-methylphenyl 2-*O*-*ortho*-(azidomethyl)benzoyl-3-*O*-benzyl-4-*O*-levulinoyl-1-thio-*α*-L-rhamnopyranoside (**S15**).

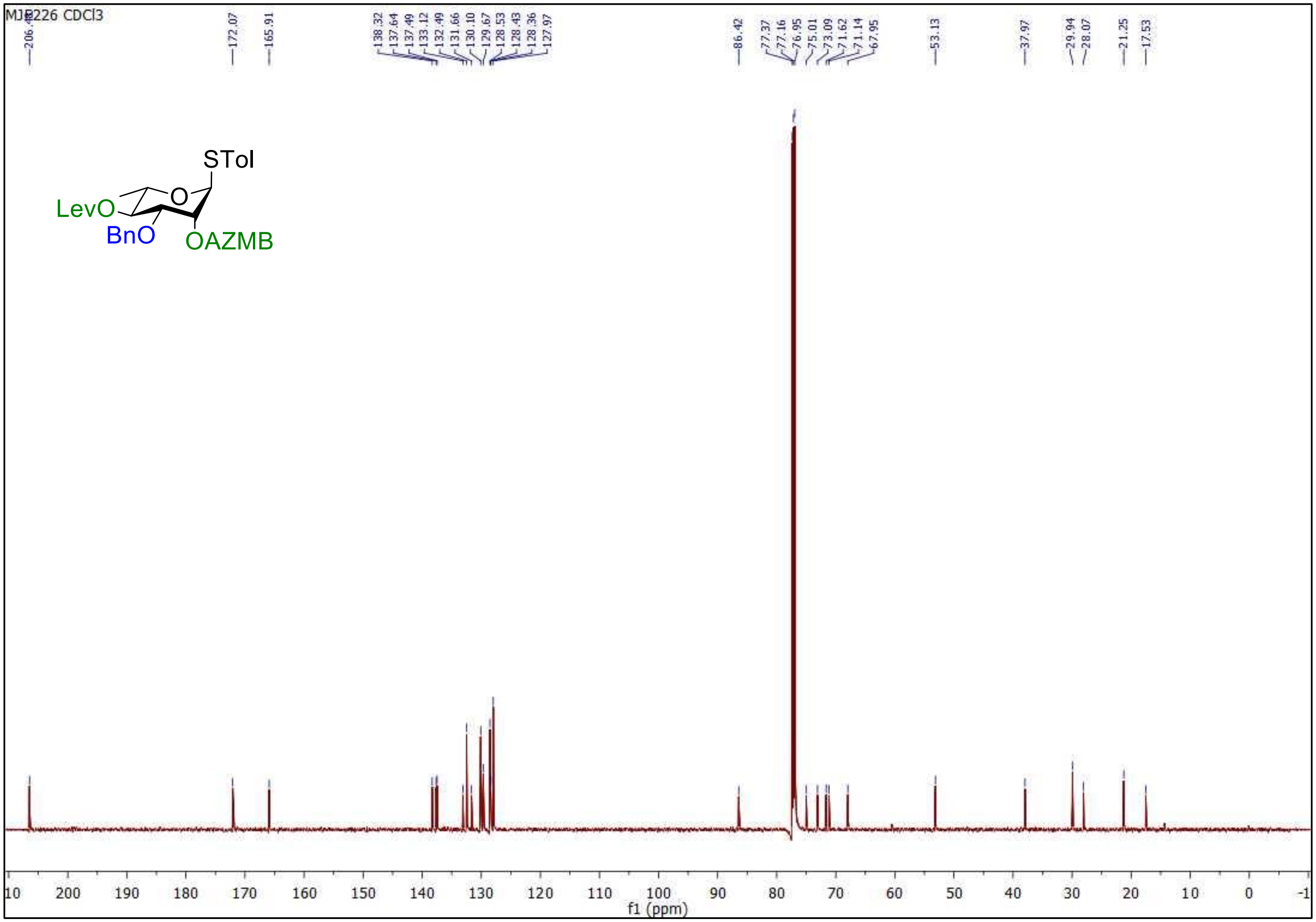

Figure S124 | HSQC NMR spectrum (CDCl$_3$, 600 MHz) of *para*-methylphenyl 2-*O*-*ortho*-(azidomethyl)benzoyl-3-*O*-benzyl-4-*O*-levulinoyl-1-thio-*α*-L-rhamnopyranoside (**S15**).

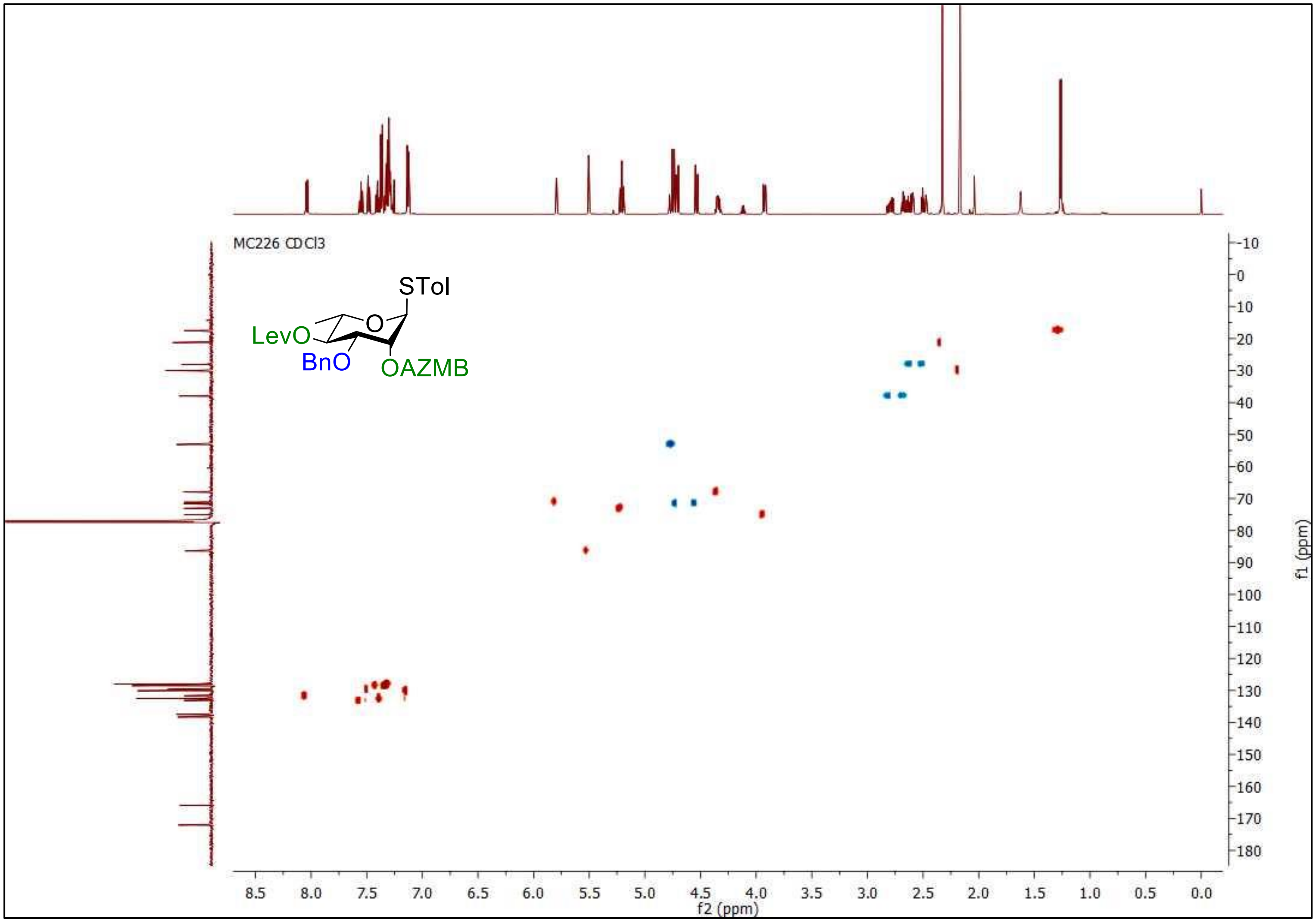

Figure S125 | ^{1}H NMR spectrum ($CDCl_3$, 600 MHz) of *para*-methylphenyl 2-*O*-*ortho*-(azidomethyl)benzoyl-3-*O*-benzyl-1-thio-*α*-L-rhamnopyranoside (**29**).

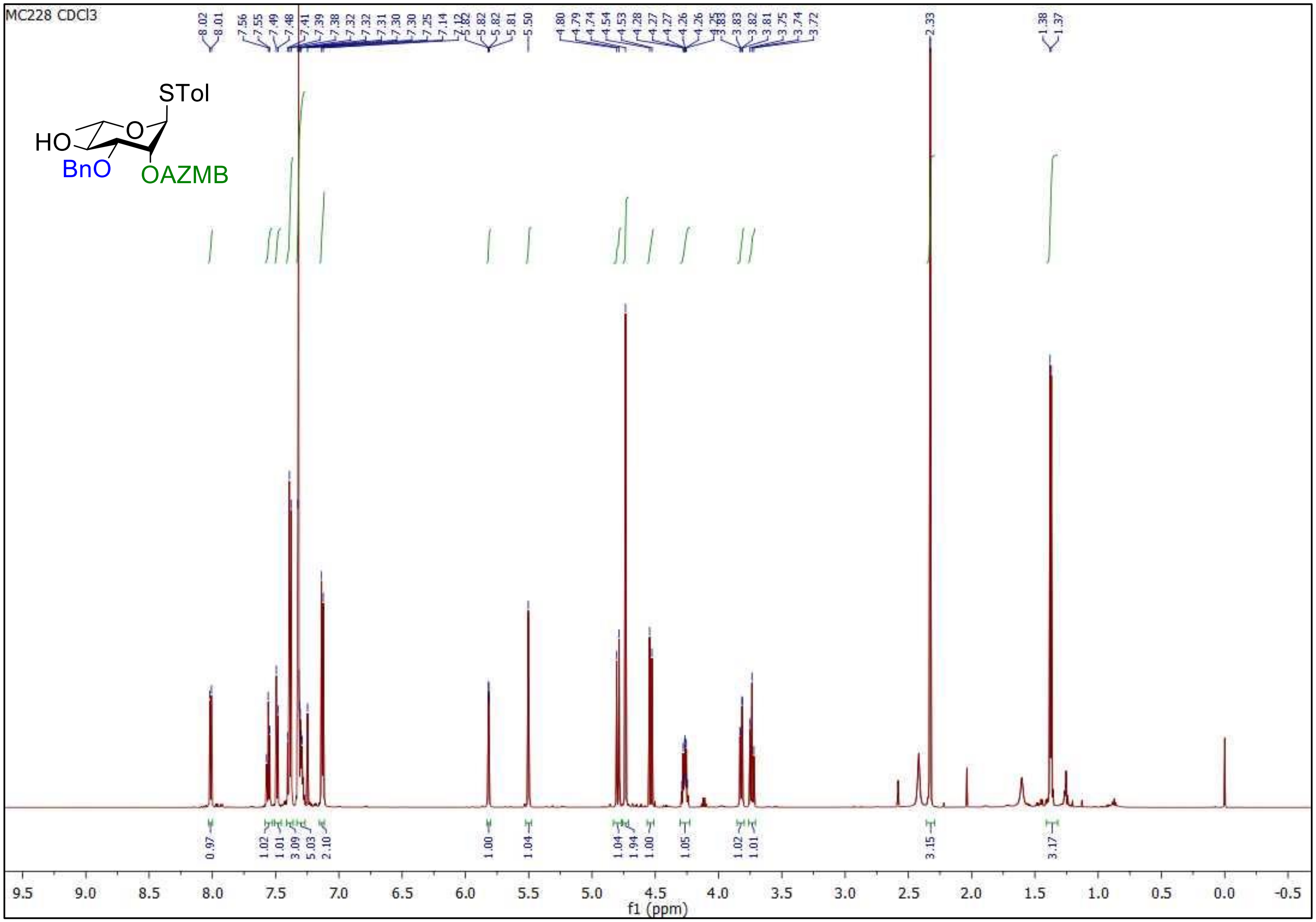

Figure S126 | COSY NMR spectrum ($CDCl_3$, 600 MHz) of *para*-methylphenyl 2-*O*-*ortho*-(azidomethyl)benzoyl-3-*O*-benzyl-1-thio-*α*-L-rhamnopyranoside (**29**).

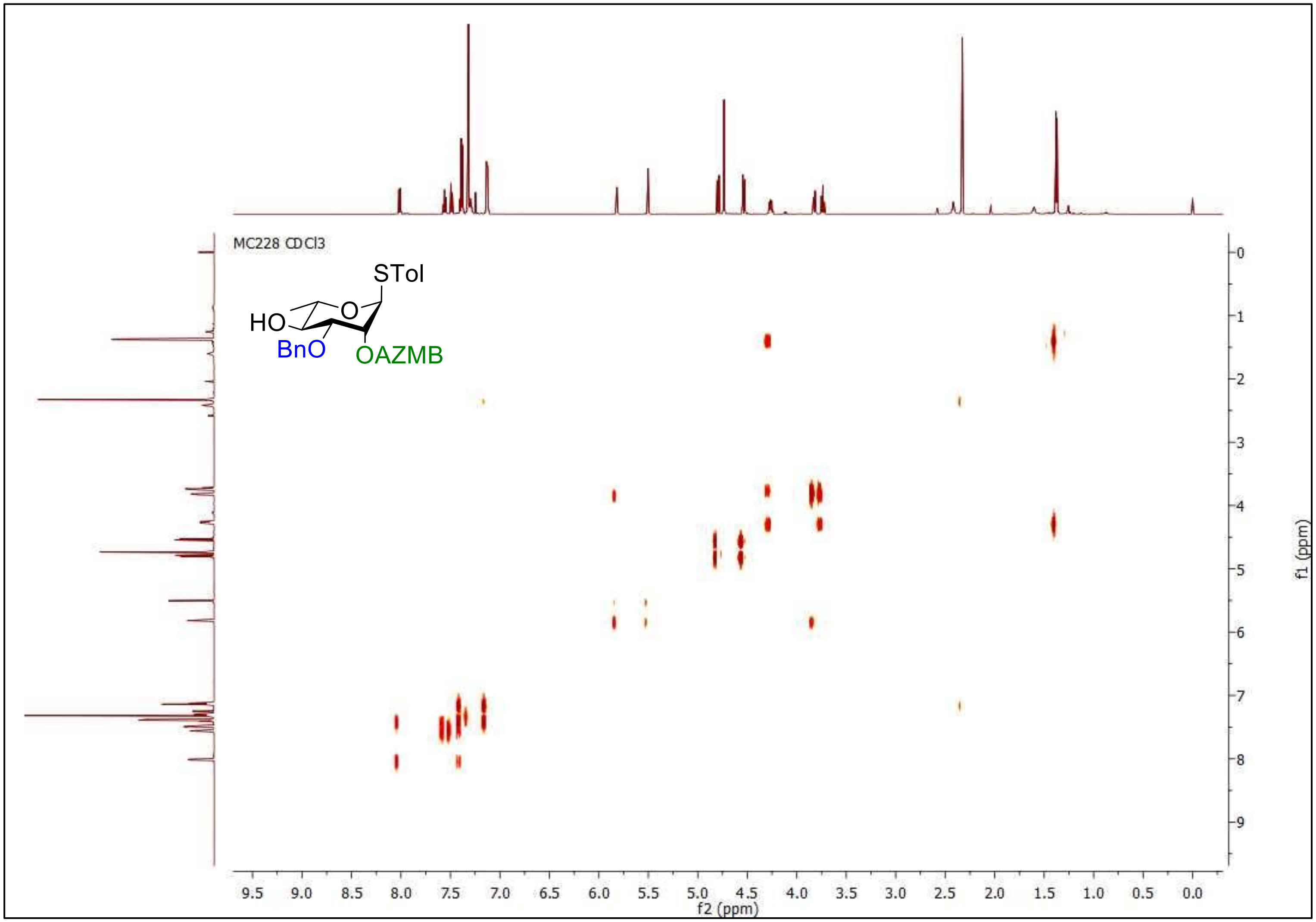

Figure S127 | ^{13}C NMR spectrum ($CDCl_3$, 600 MHz) of *para*-methylphenyl 2-*O*-*ortho*-(azidomethyl)benzoyl-3-*O*-benzyl-1-thio-*α*-L-rhamnopyranoside (**29**).

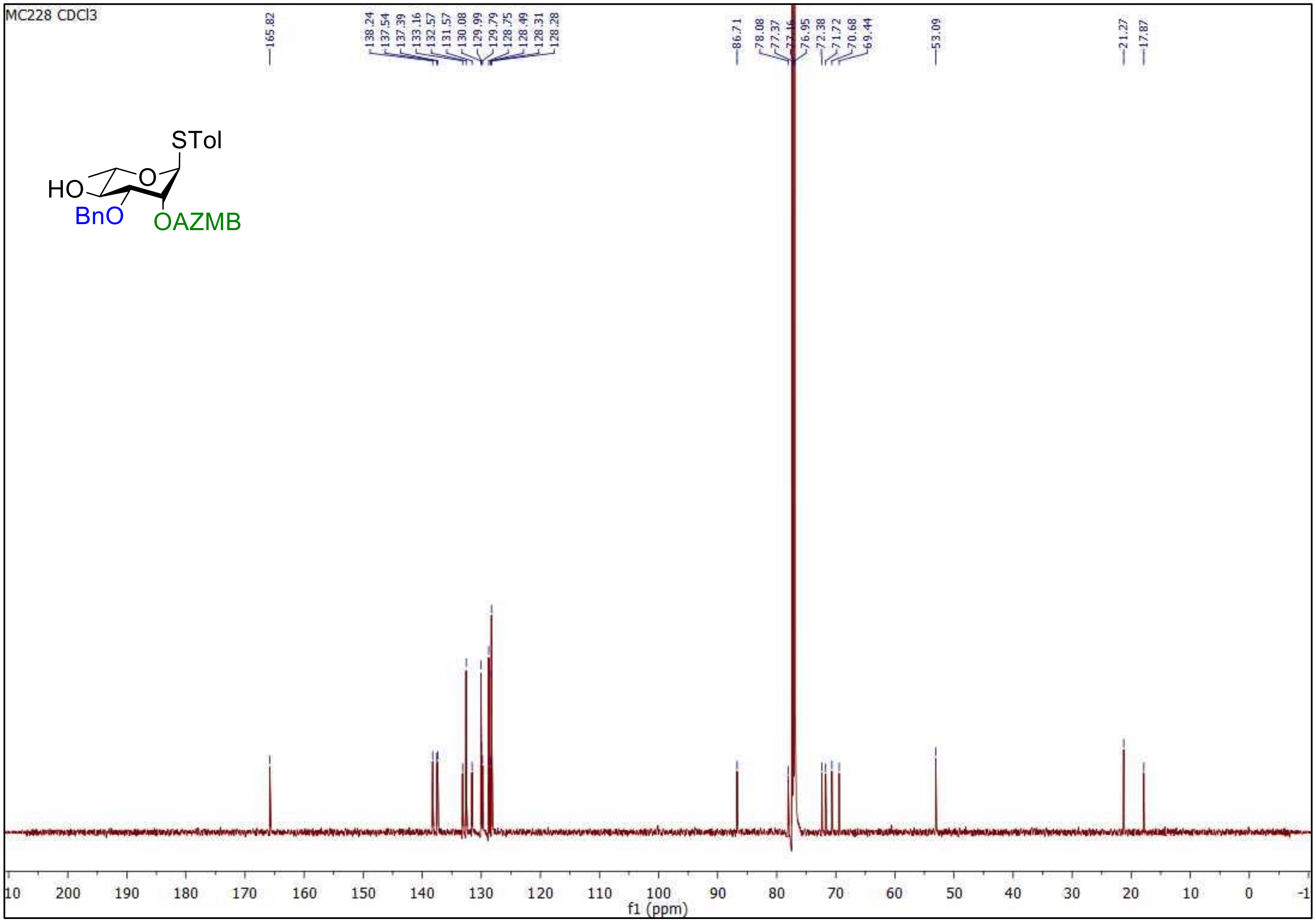

Figure S128 | HSQC NMR spectrum ($CDCl_3$, 600 MHz) of *para*-methylphenyl 2-*O*-*ortho*-(azidomethyl)benzoyl-3-*O*-benzyl-1-thio-*α*-L-rhamnopyranoside (**29**).

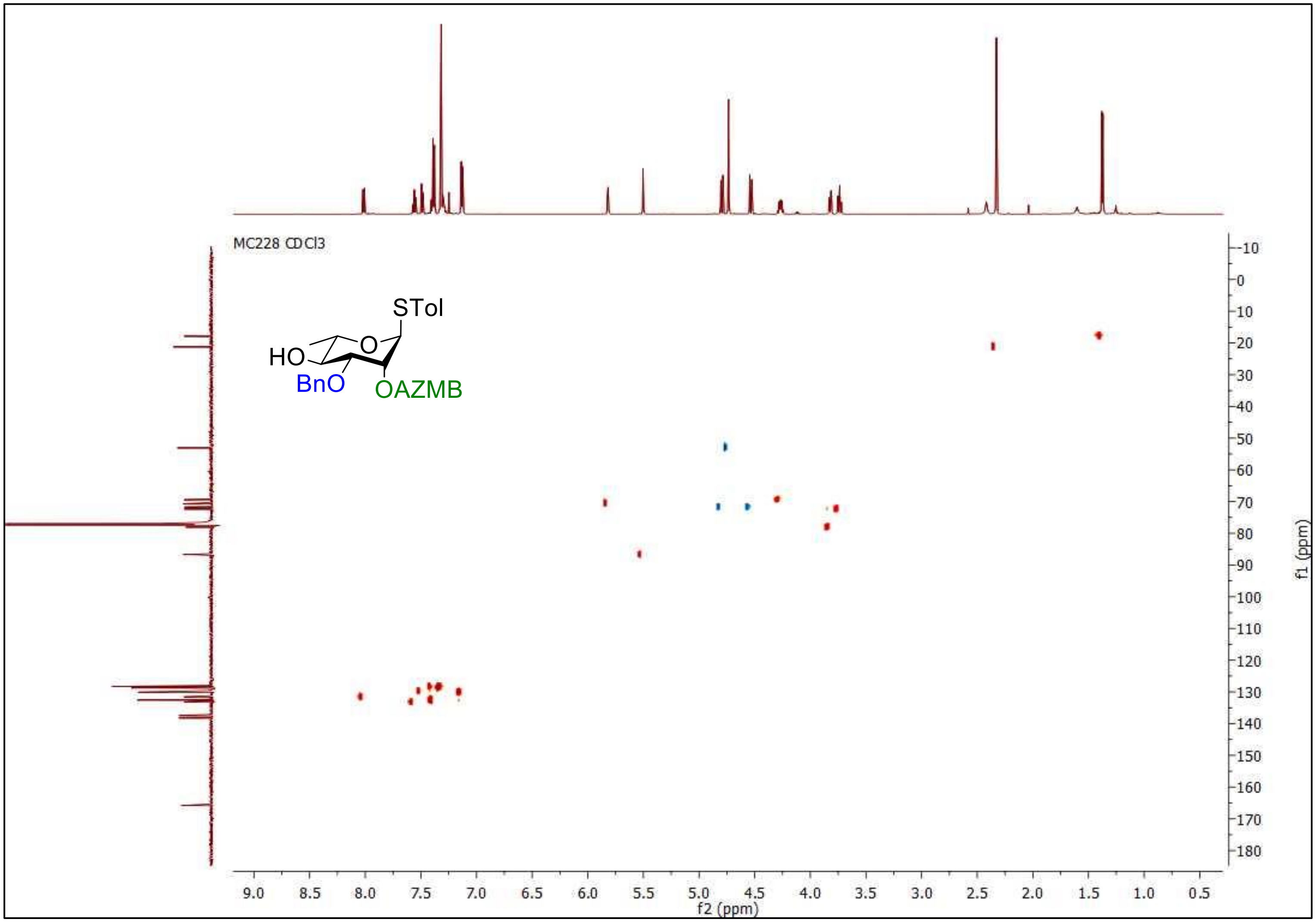

Figure S129 | ^{1}H NMR spectrum ($CDCl_3$, 600 MHz) of *para*-methylphenyl 2-*O*-*ortho*-(azidomethyl)benzoyl-3-*O*-benzyl-4-*O*-(*R*)-3-(((*R*)-3-hydroxydecanoyl)oxy)decanoyl-1-thio-*α*-L-rhamnopyranoside (**34**).

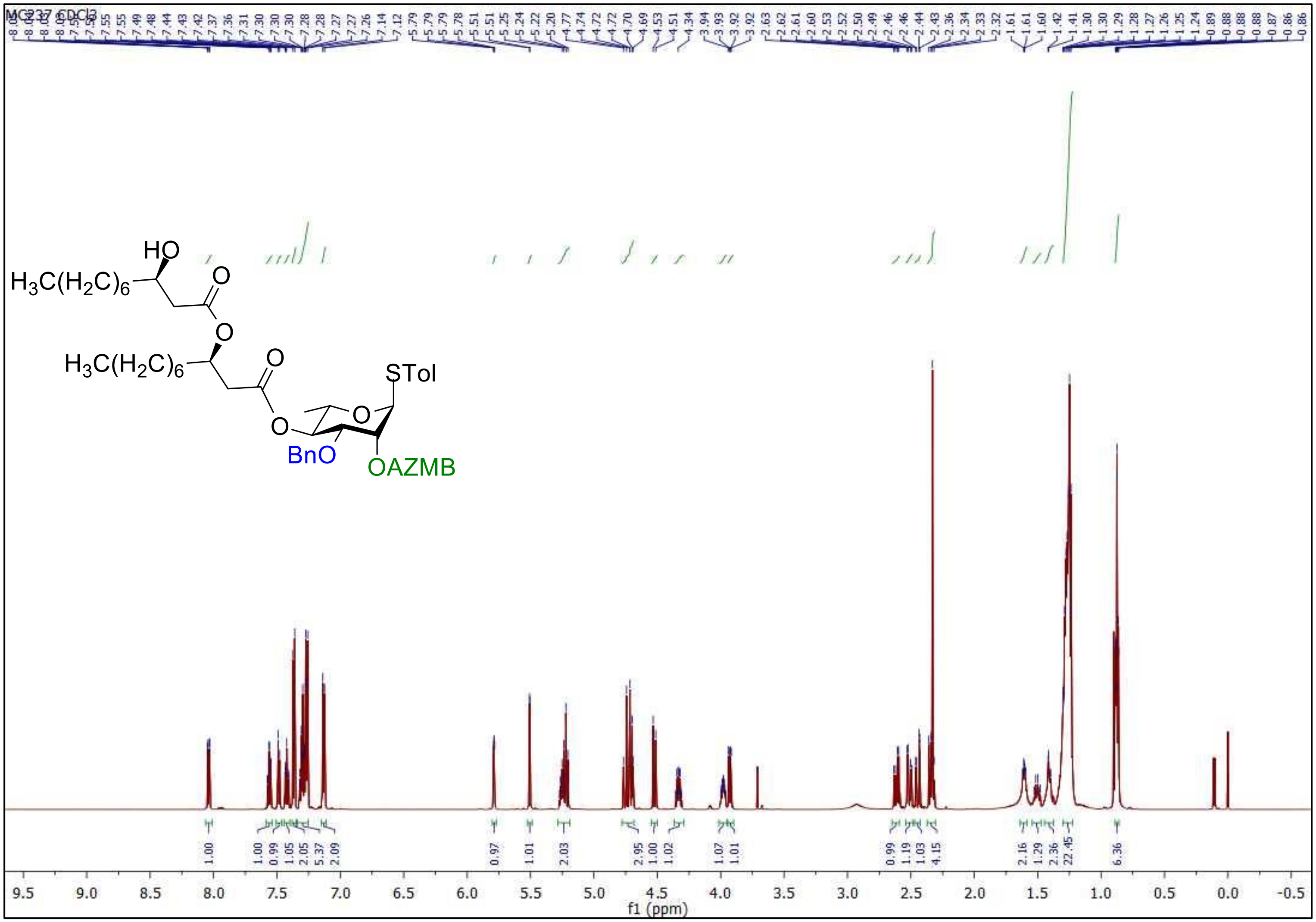

Figure S130 | COSY NMR spectrum (CDCl3, 600 MHz) of *para*-methylphenyl 2-*O*-*ortho*-(azidomethyl)benzoyl-3-*O*-benzyl-4-*O*-(*R*)-3-(((*R*)-3-hydroxydecanoyl)oxy)decanoyl-1-thio-*α*-L-rhamnopyranoside (**34**).

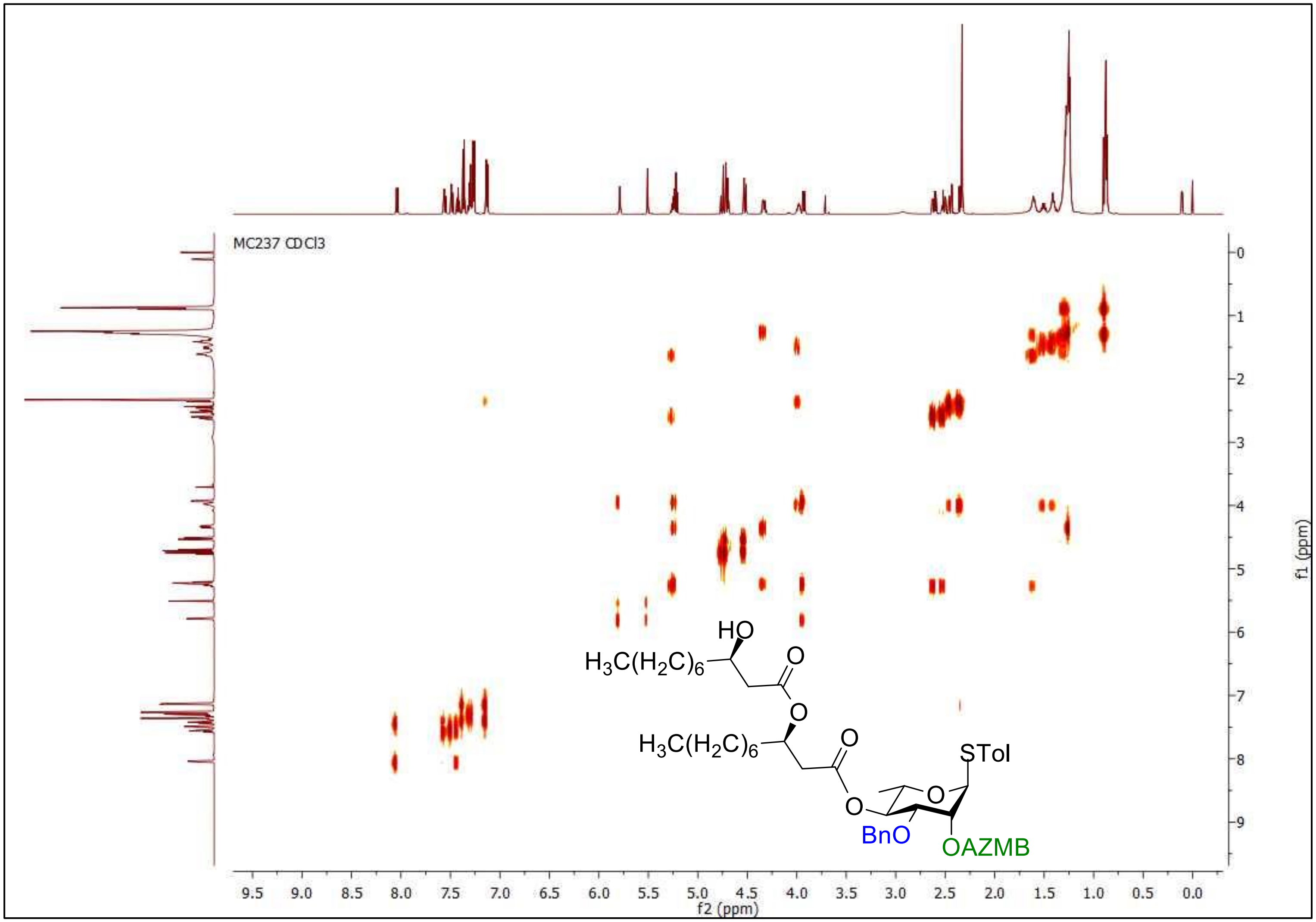

Figure S131 | ^{13}C NMR spectrum ($CDCl_3$, 600 MHz) of *para*-methylphenyl 2-*O*-*ortho*-(azidomethyl)benzoyl-3-*O*-benzyl-4-*O*-(*R*)-3-(((*R*)-3-hydroxydecanoyl)oxy)decanoyl-1-thio-*α*-L-rhamnopyranoside (**34**).

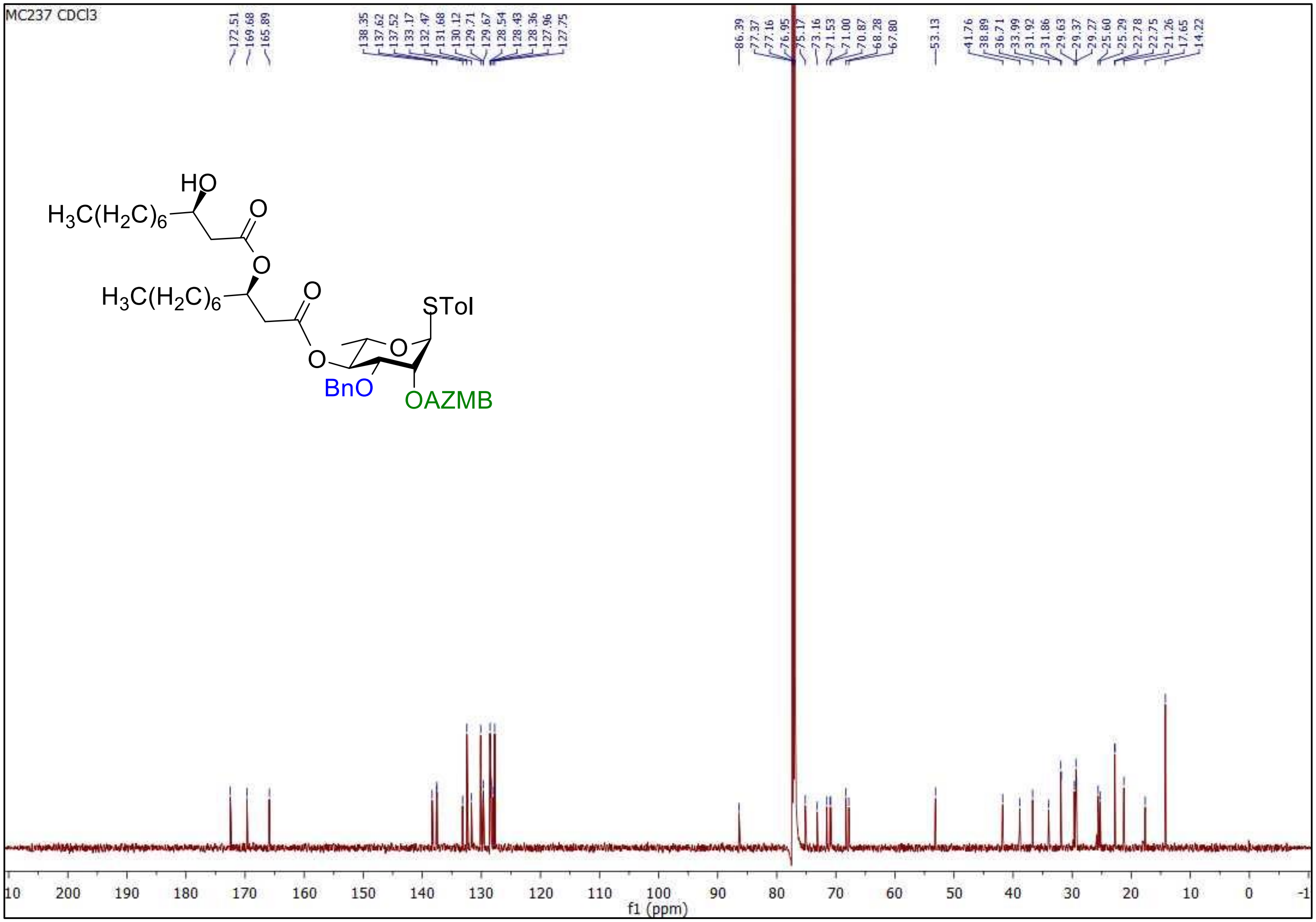

Figure S132 | HSQC NMR spectrum (CDCl3, 600 MHz) of *para*-methylphenyl 2-*O*-*ortho*-(azidomethyl)benzoyl-3-*O*-benzyl-4-*O*-(*R*)-3-(((*R*)-3-hydroxydecanoyl)oxy)decanoyl-1-thio-*α*-L-rhamnopyranoside (**34**).

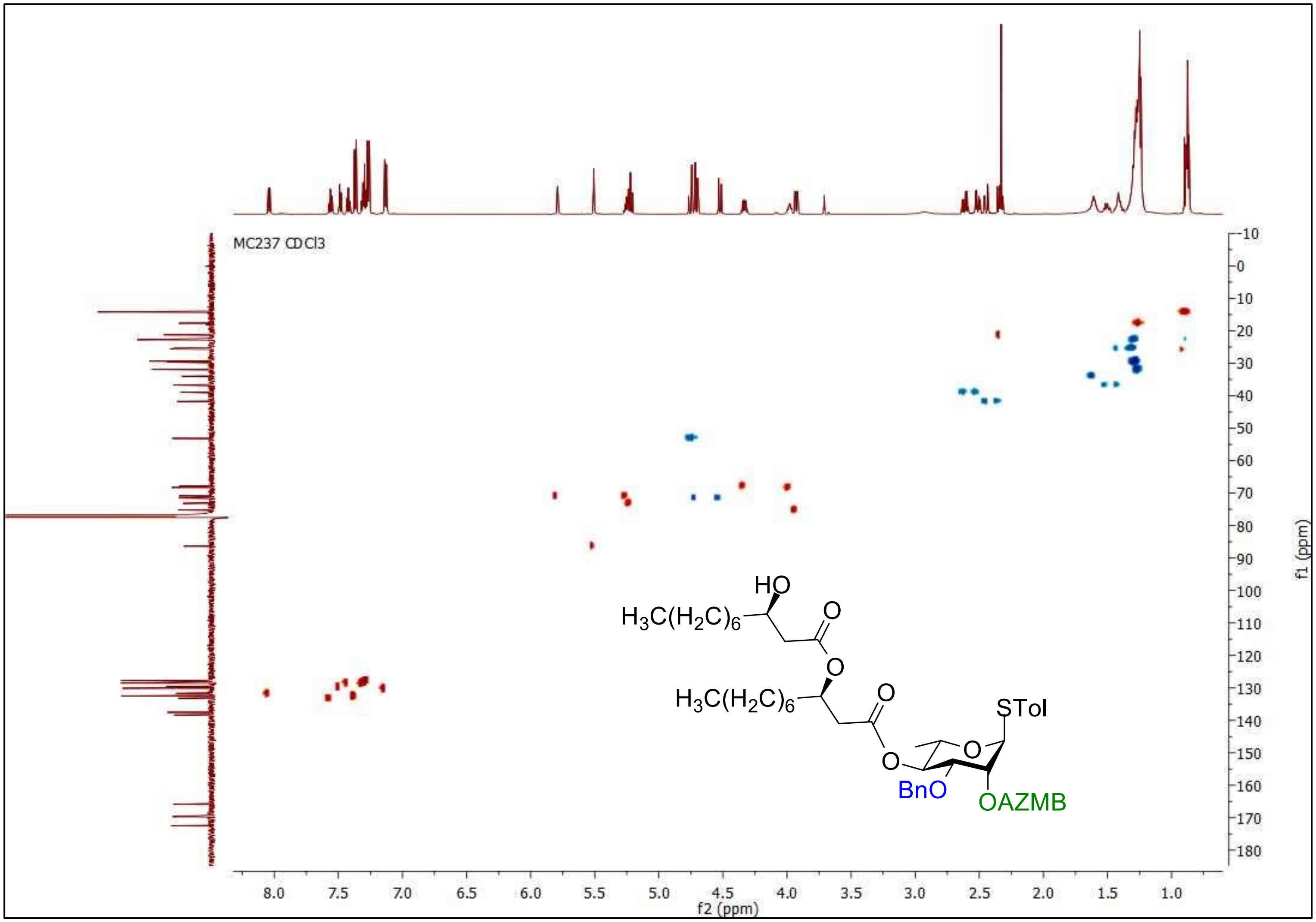

Figure S133 | ^{1}H NMR spectrum ($CDCl_3$, 600 MHz) of macrolide **35**.

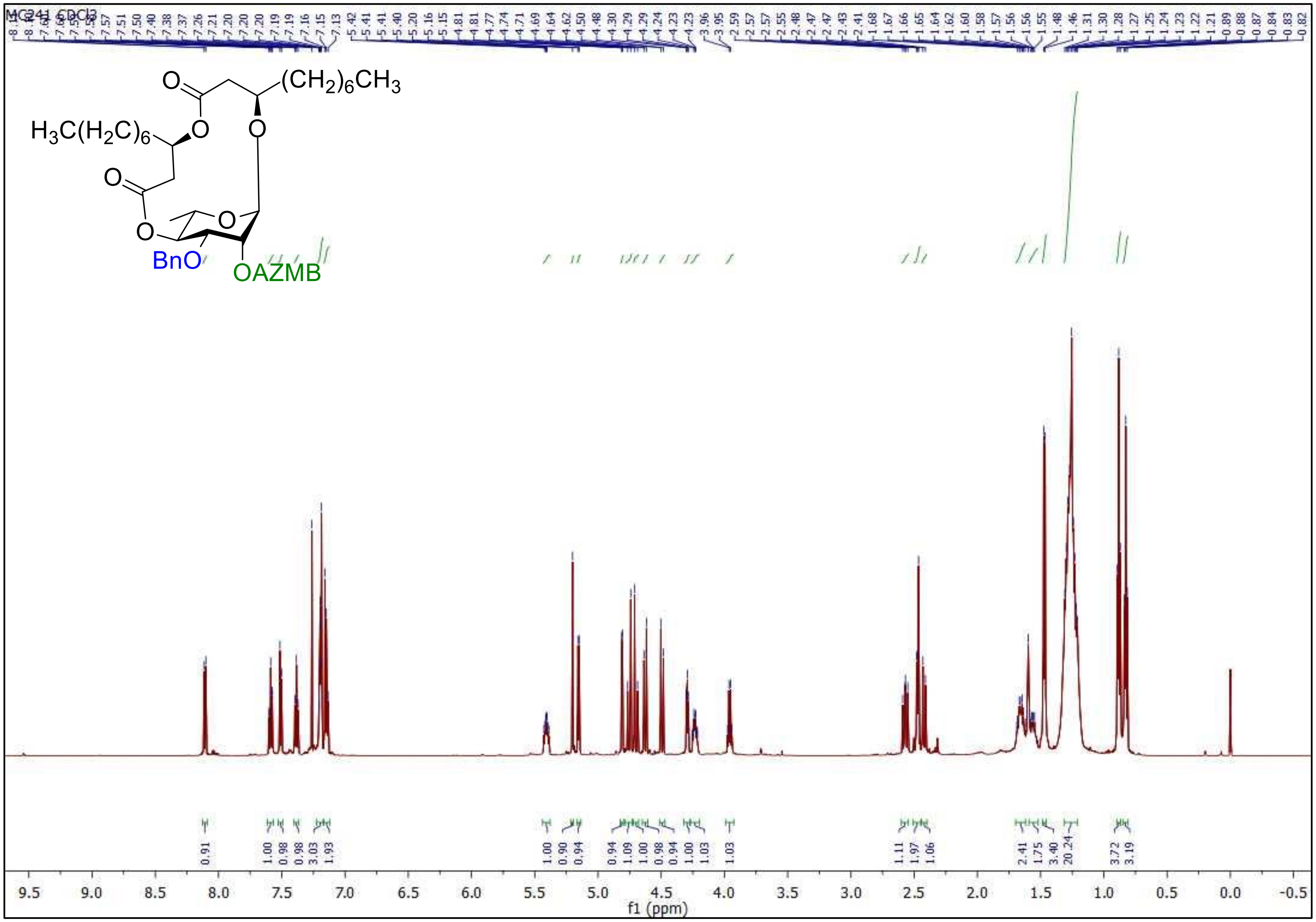

Figure S134 | COSY NMR spectrum ($CDCl_3$, 600 MHz) of macrolide **35**.

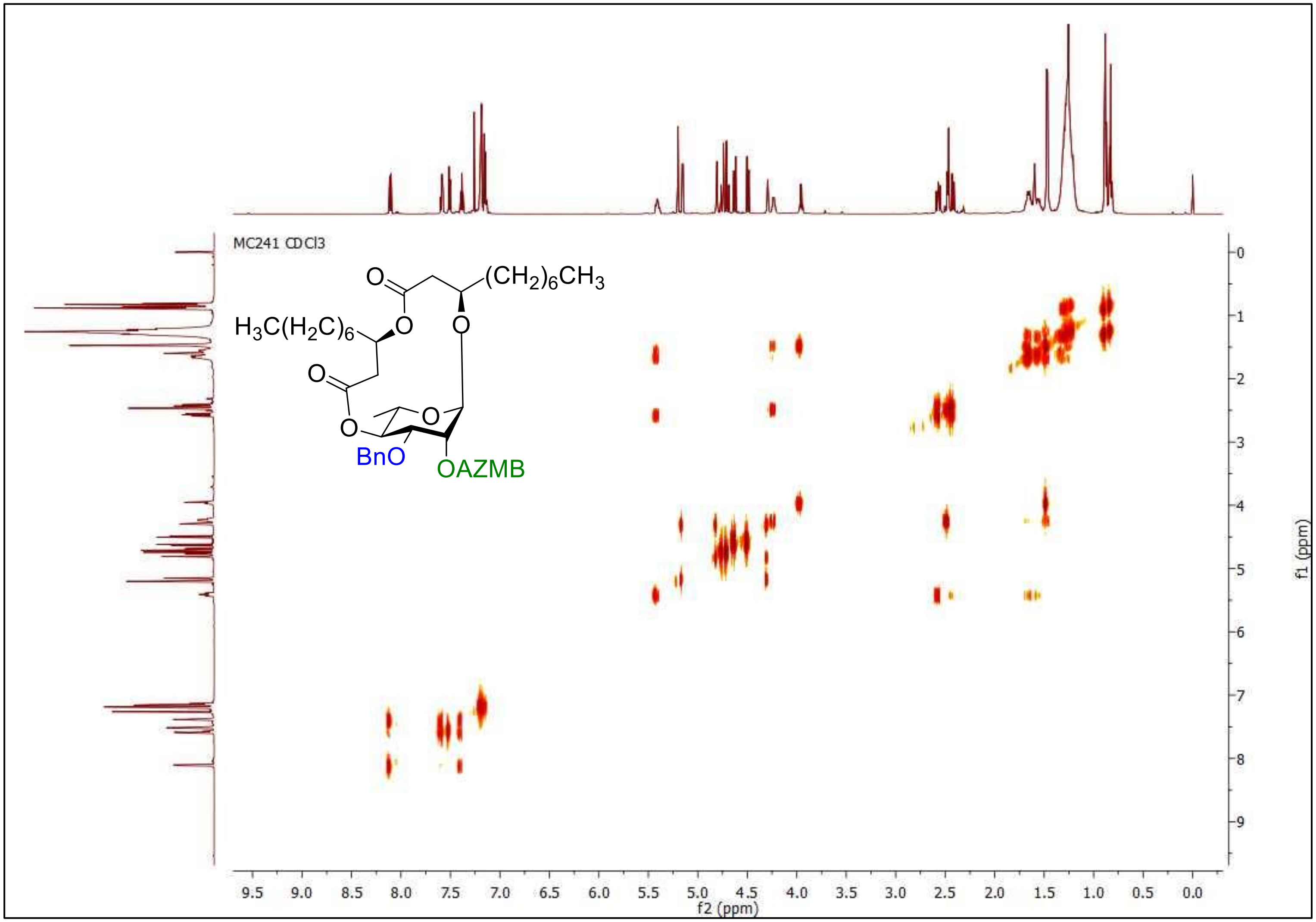

Figure S135 | ^{13}C NMR spectrum ($CDCl_3$, 600 MHz) of macrolide **35**.

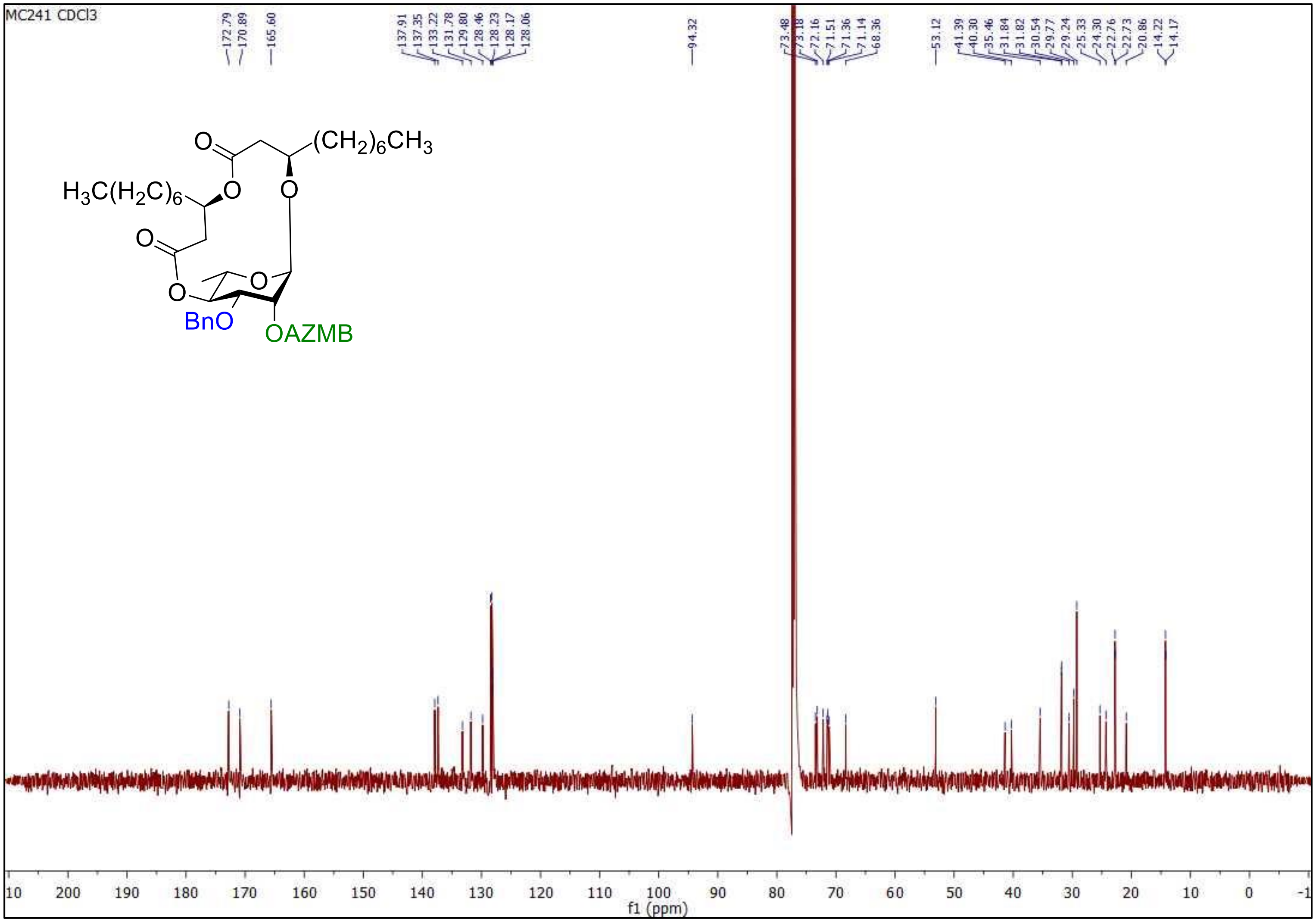

Figure S136 | HSQC NMR spectrum ($CDCl_3$, 600 MHz) of macrolide **35**.

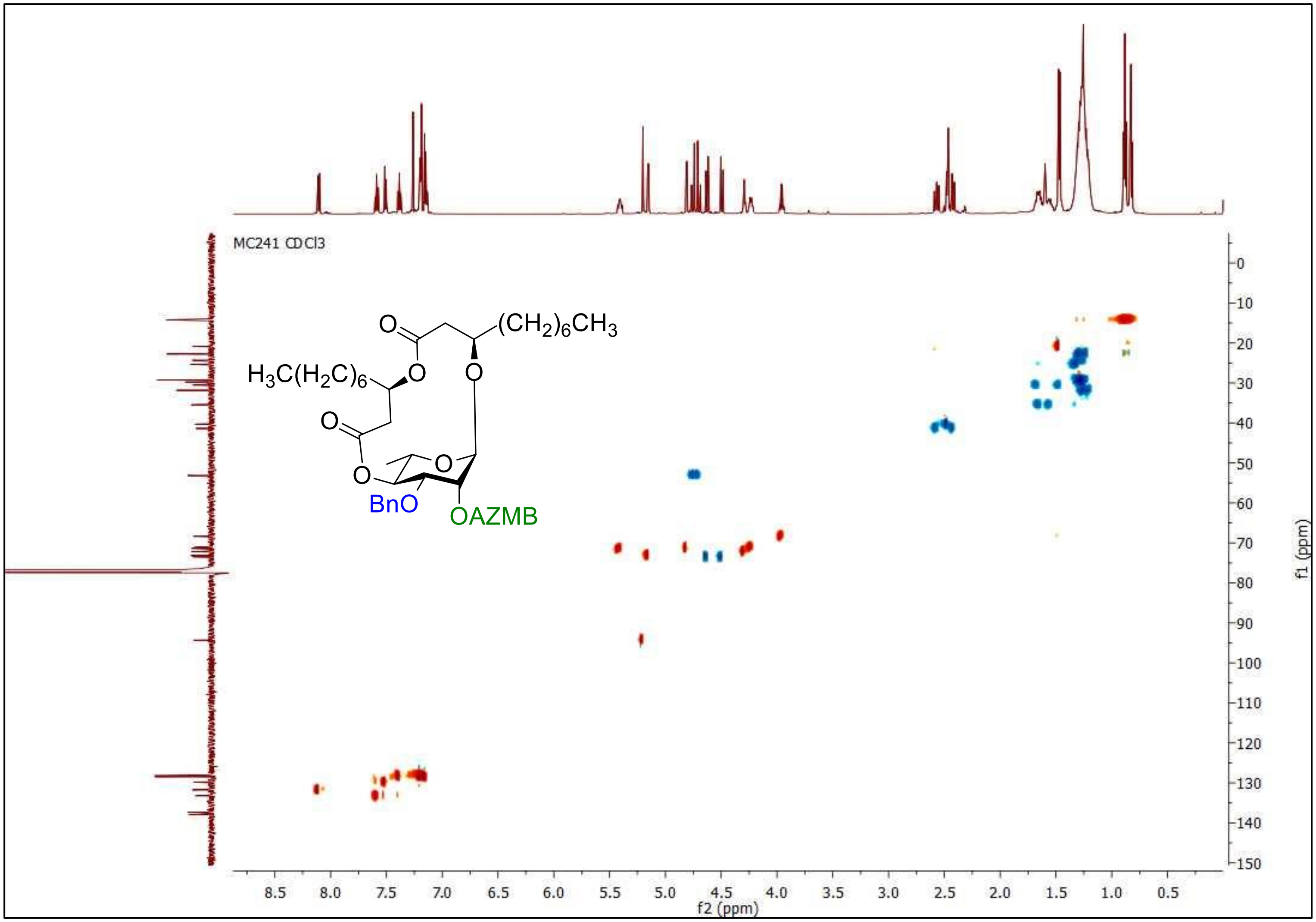

Figure S137 | undecoupled HSQC NMR spectrum ($CDCl_3$, 600 MHz) of macrolide **35**.

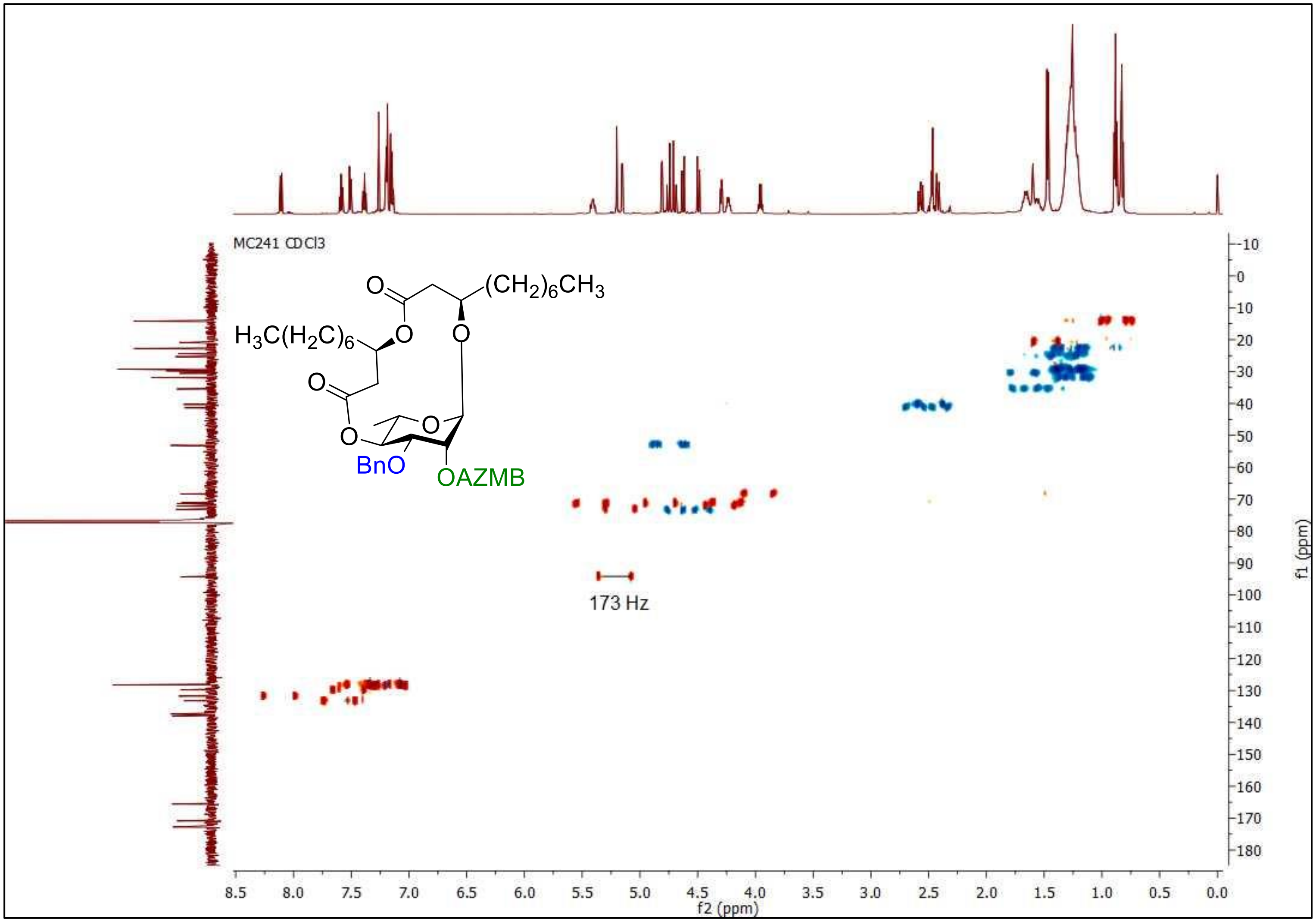

Figure S138 | ^{1}H NMR spectrum ($CDCl_3$, 600 MHz) of macrolide **S16**.

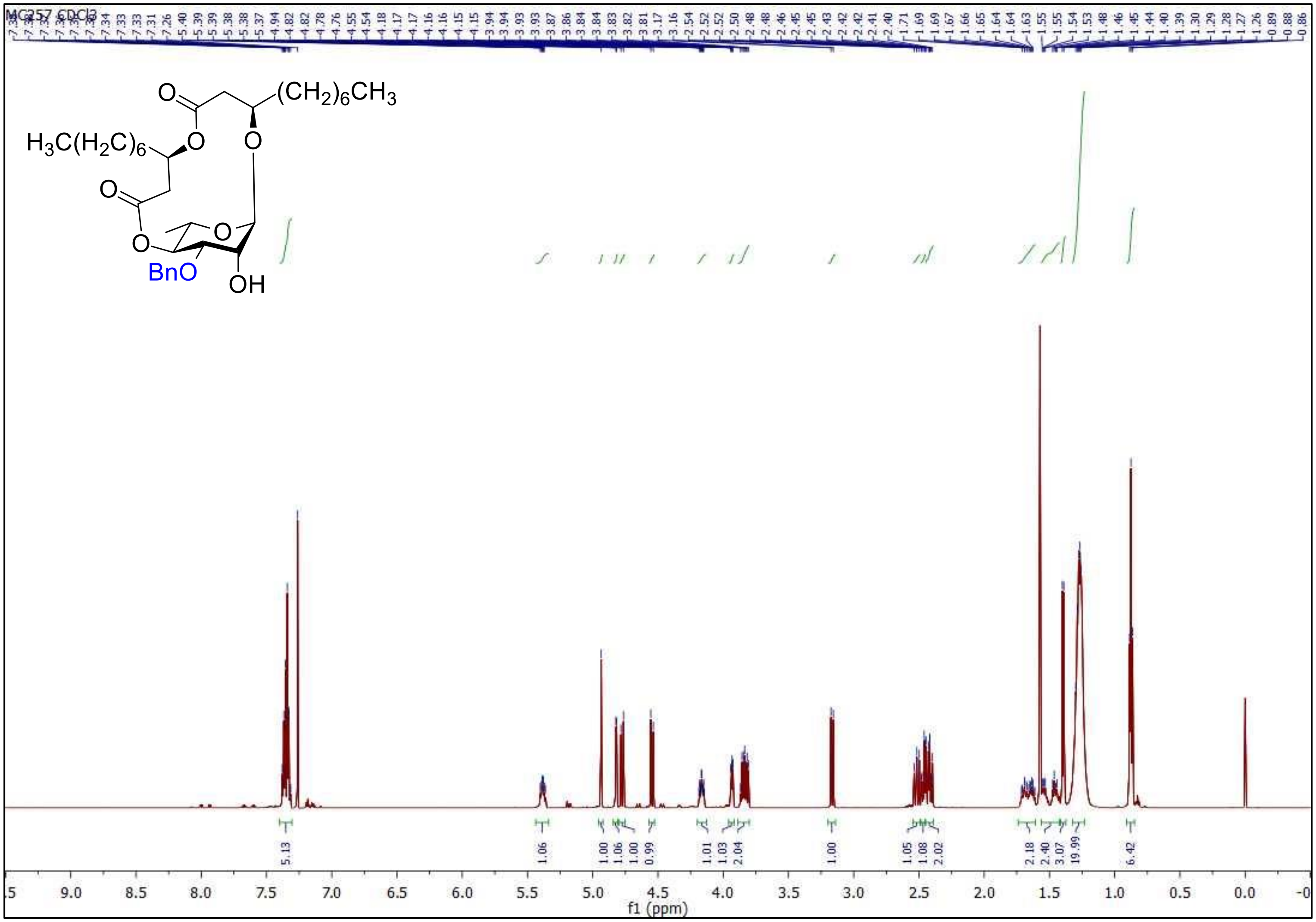

Figure S139 | COSY NMR spectrum ($CDCl_3$, 600 MHz) of macrolide **S16**.

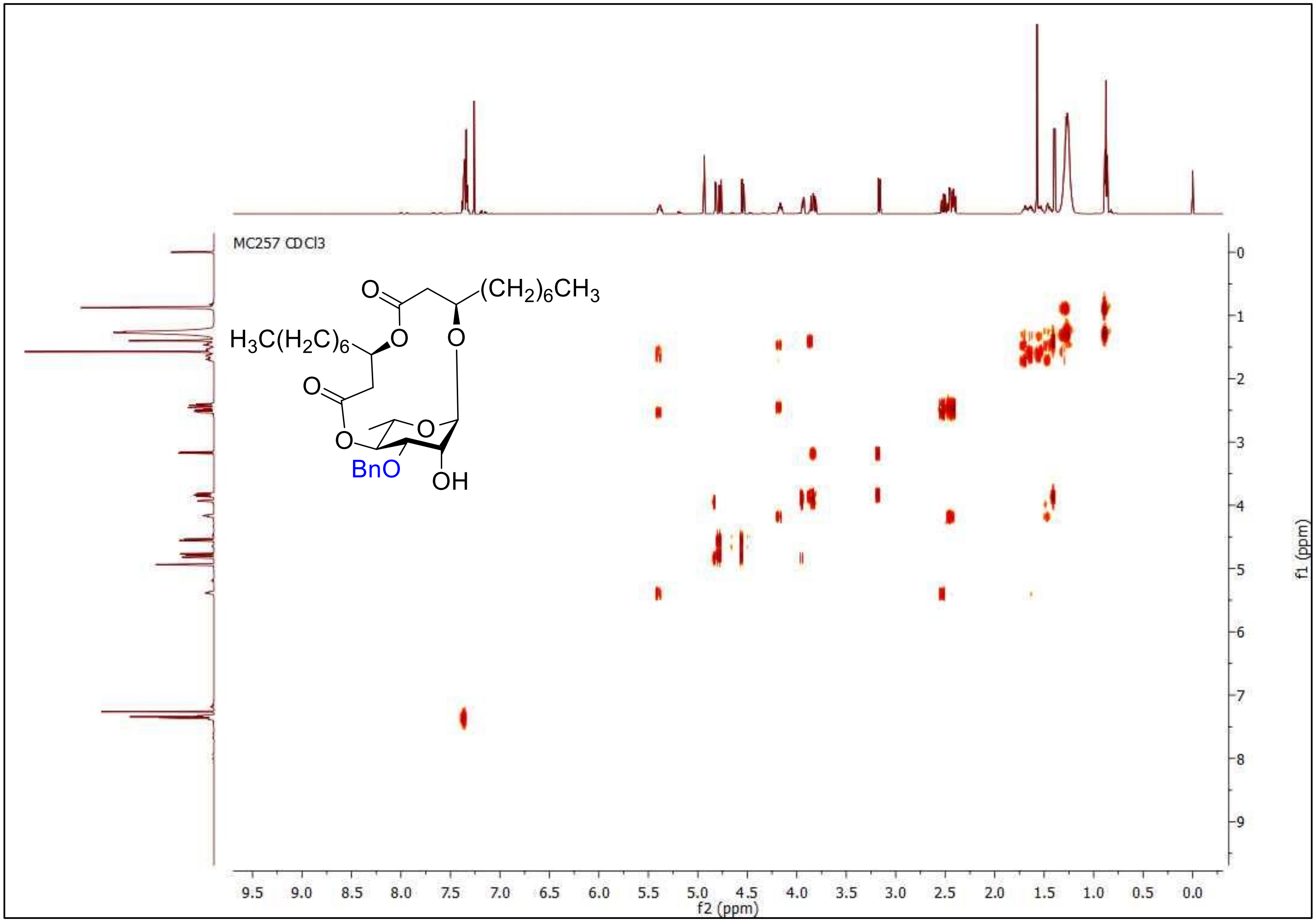

Figure S140 | ^{13}C NMR spectrum ($CDCl_3$, 600 MHz) of macrolide **S16**.

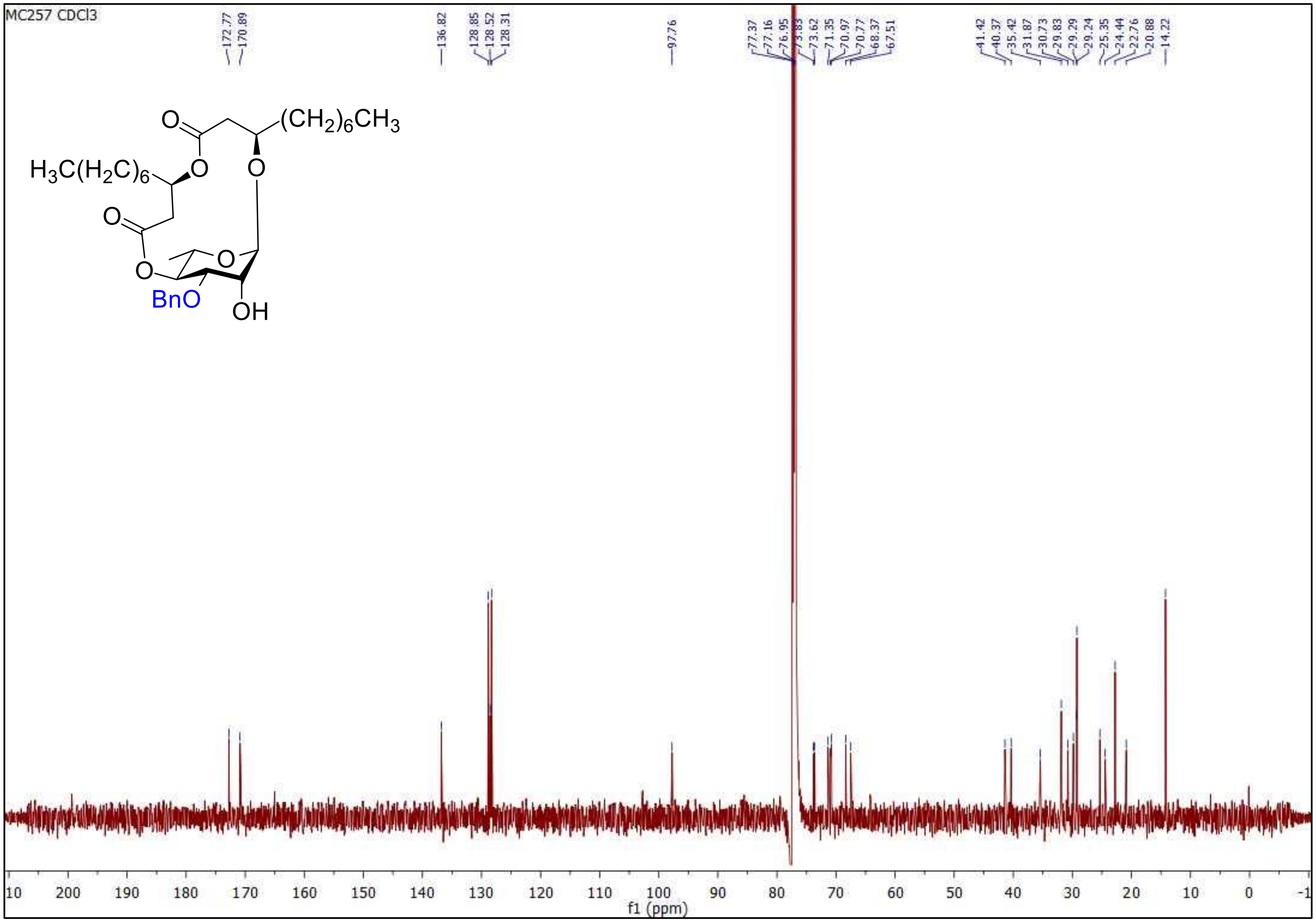

Figure S141 | HSQC NMR spectrum ($CDCl_3$, 600 MHz) of macrolide **S16**.

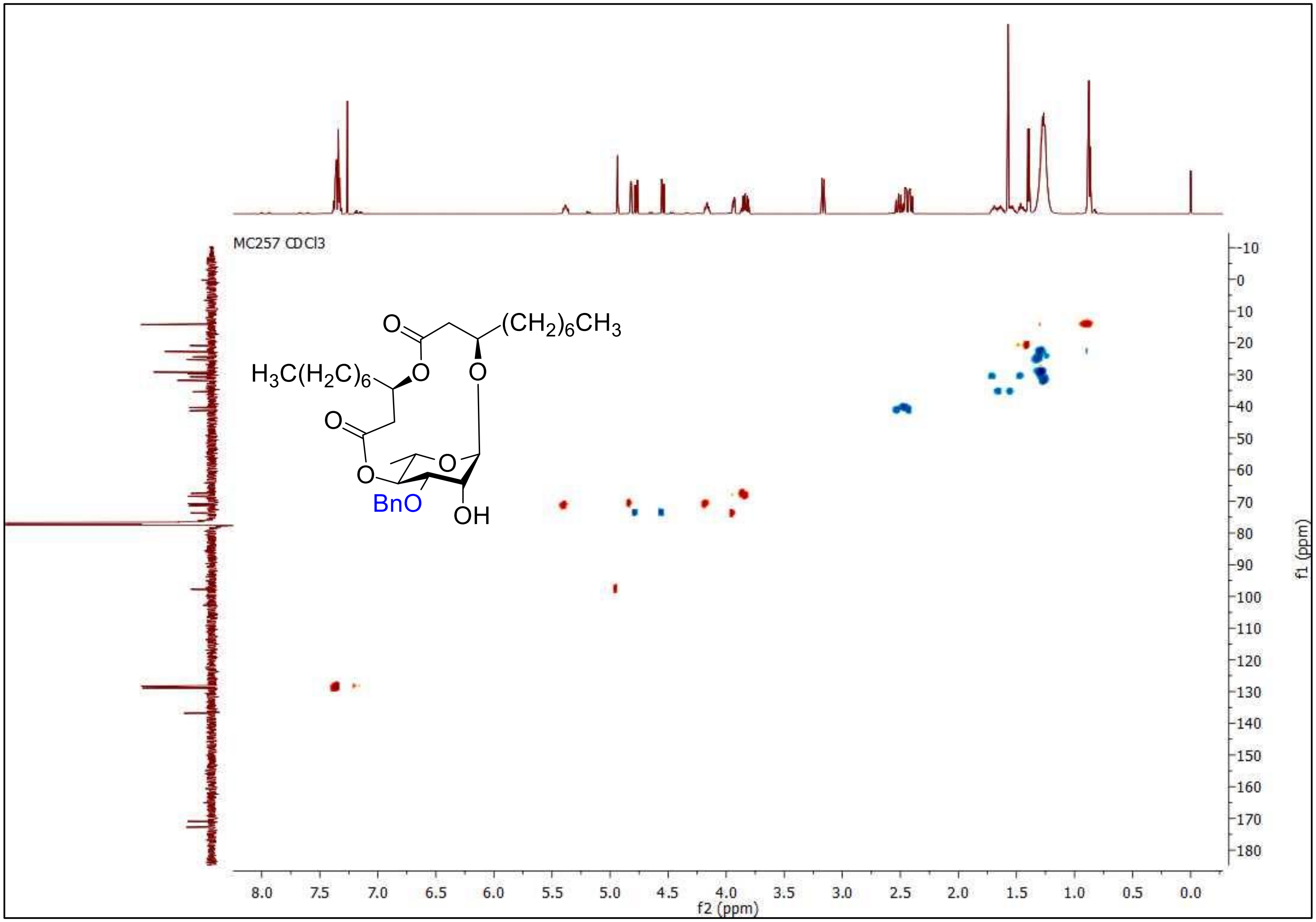

Figure S142 | ^{1}H NMR spectrum ($CDCl_3$, 600 MHz) of (1→4)-macrolactonized rhamnolipid **4**.

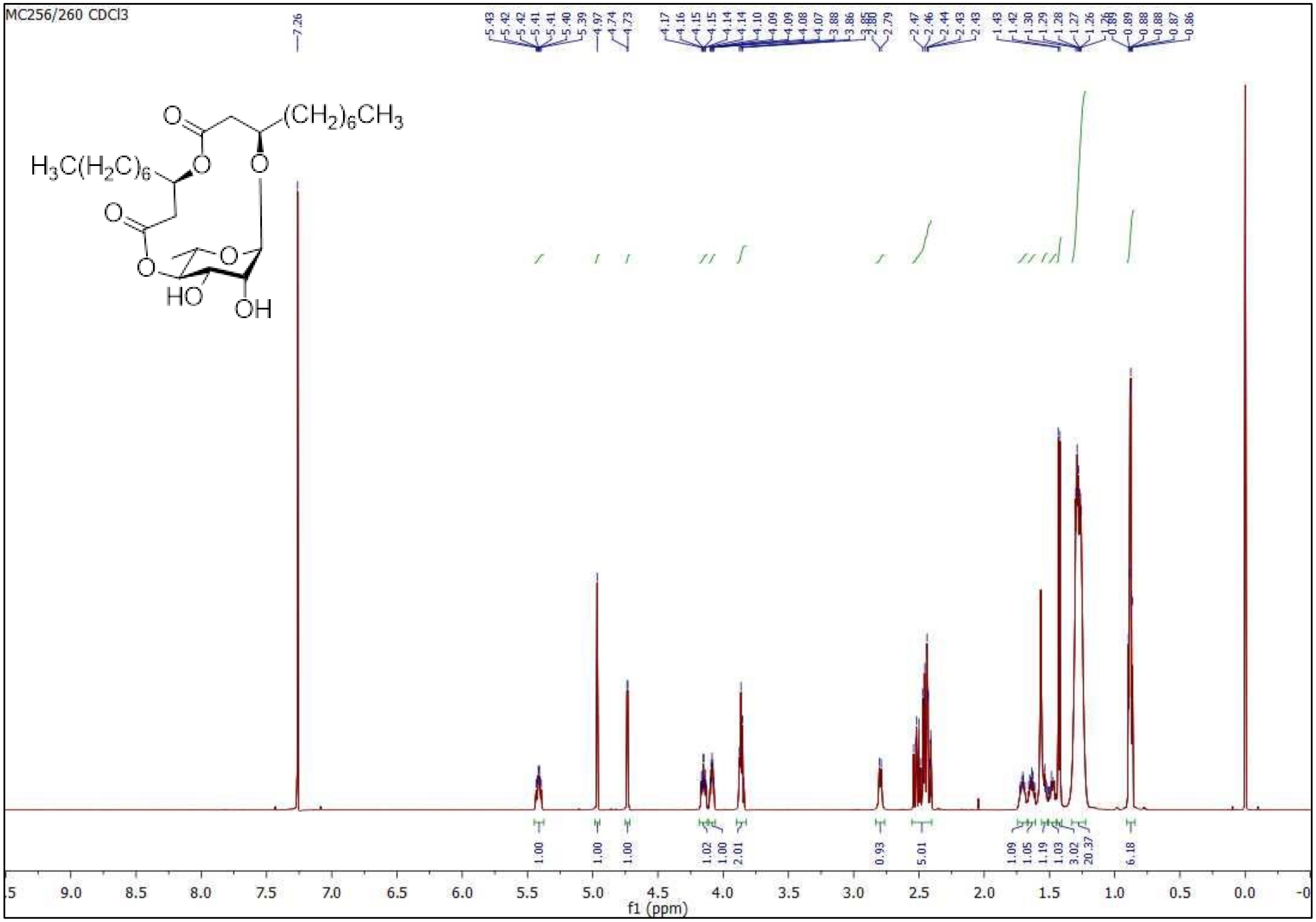

Figure S143 | COSY NMR spectrum ($CDCl_3$, 600 MHz) of (1→4)-macrolactonized rhamnolipid **4**.

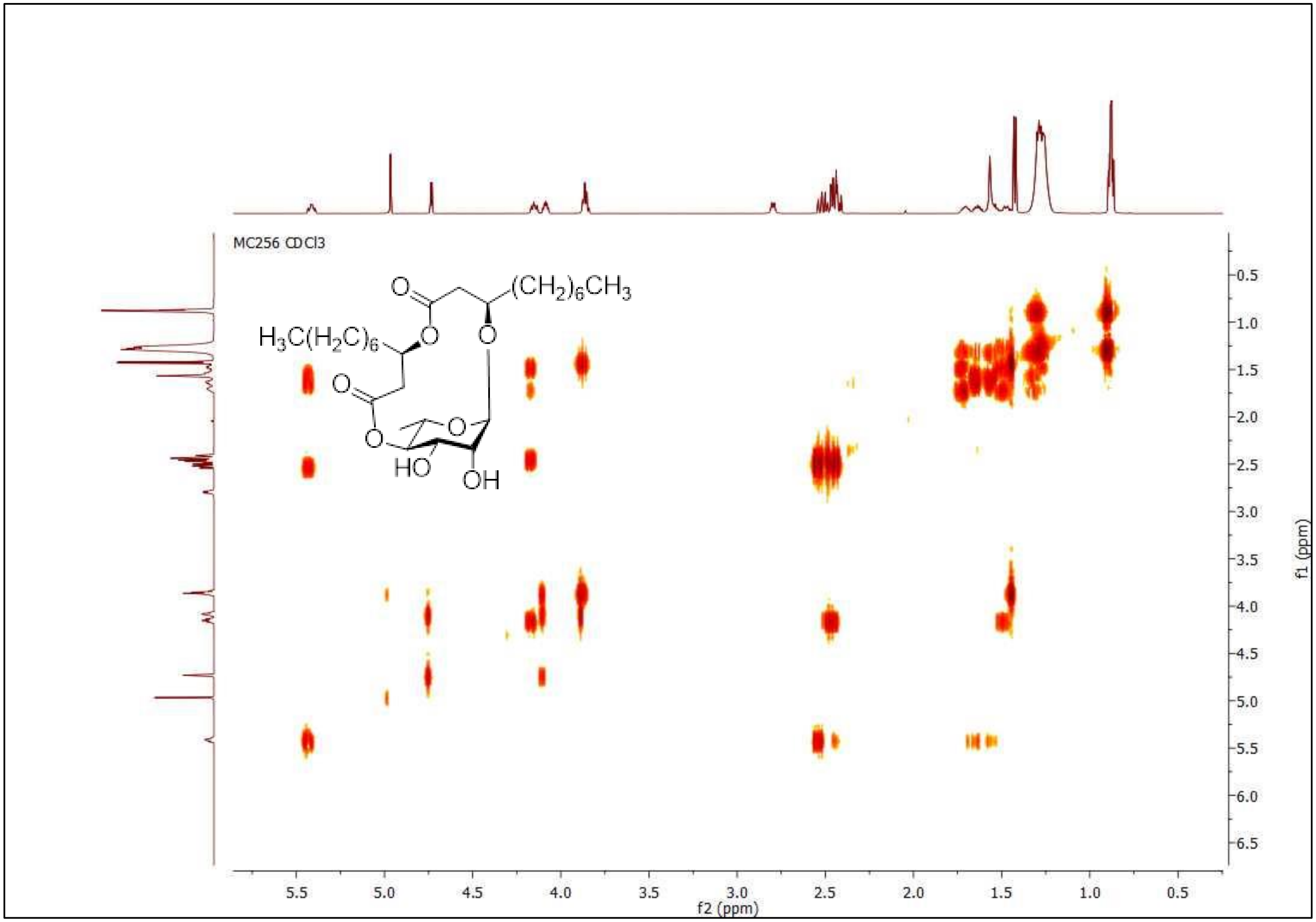

Figure S144 | ^{13}C NMR spectrum ($CDCl_3$, 600 MHz) of (1→4)-macrolactonized rhamnolipid **4**.

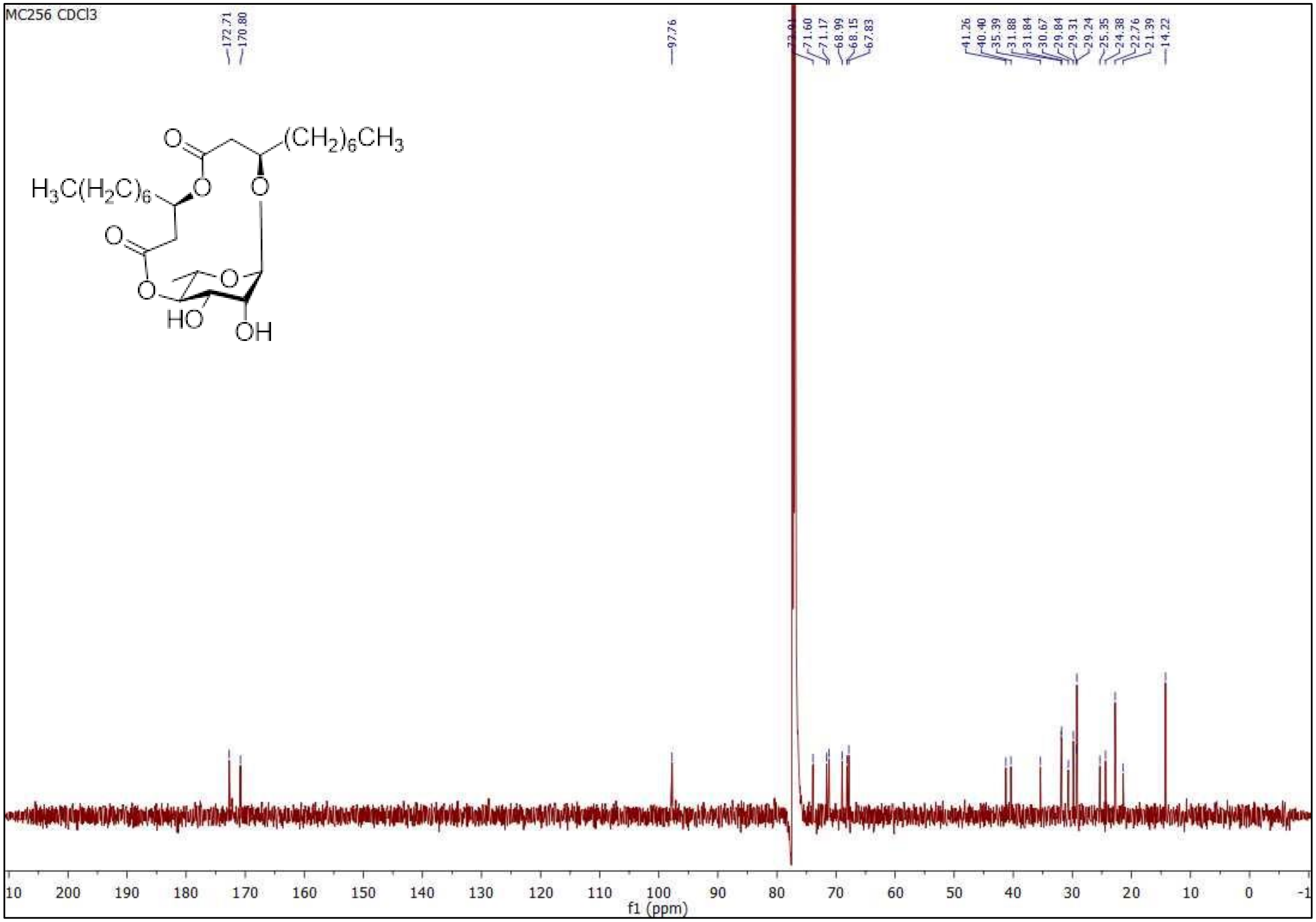

Figure S145 | HSQC NMR spectrum ($CDCl_3$, 600 MHz) of (1→4)-macrolactonized rhamnolipid **4**.

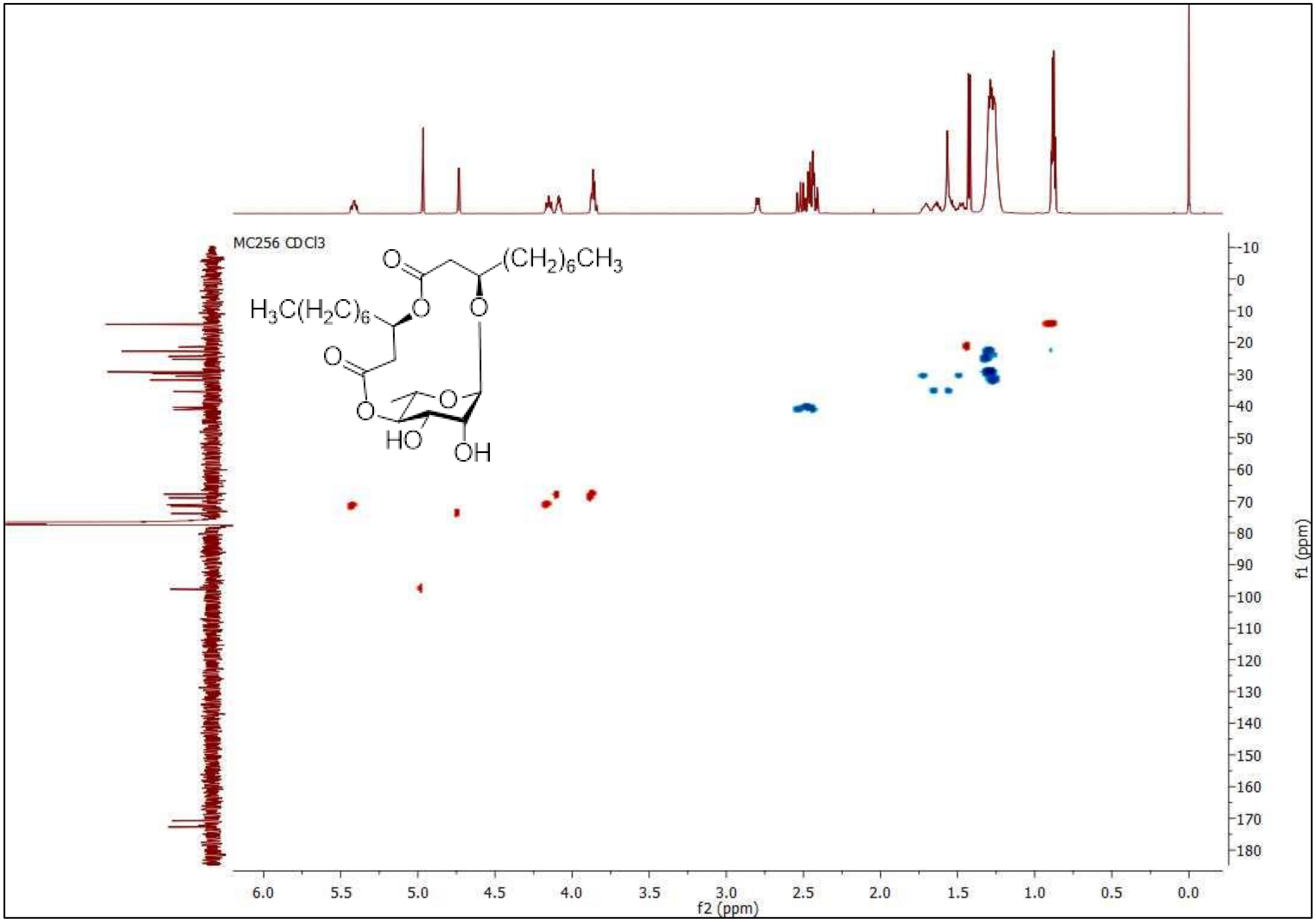

Figure S146 | ^{1}H NMR spectrum (CDCl3, 600 MHz) of *para*-methylphenyl 3-*O*-benzyl-2-*O*-(*R*)-3-(((*R*)-3-(*tert*-butyldimethylsilyl)oxy)decanoyl)oxy)decanoyl-4-*O*-levulinoyl-1-thio-*α*-L-rhamnopyranoside (**27**).

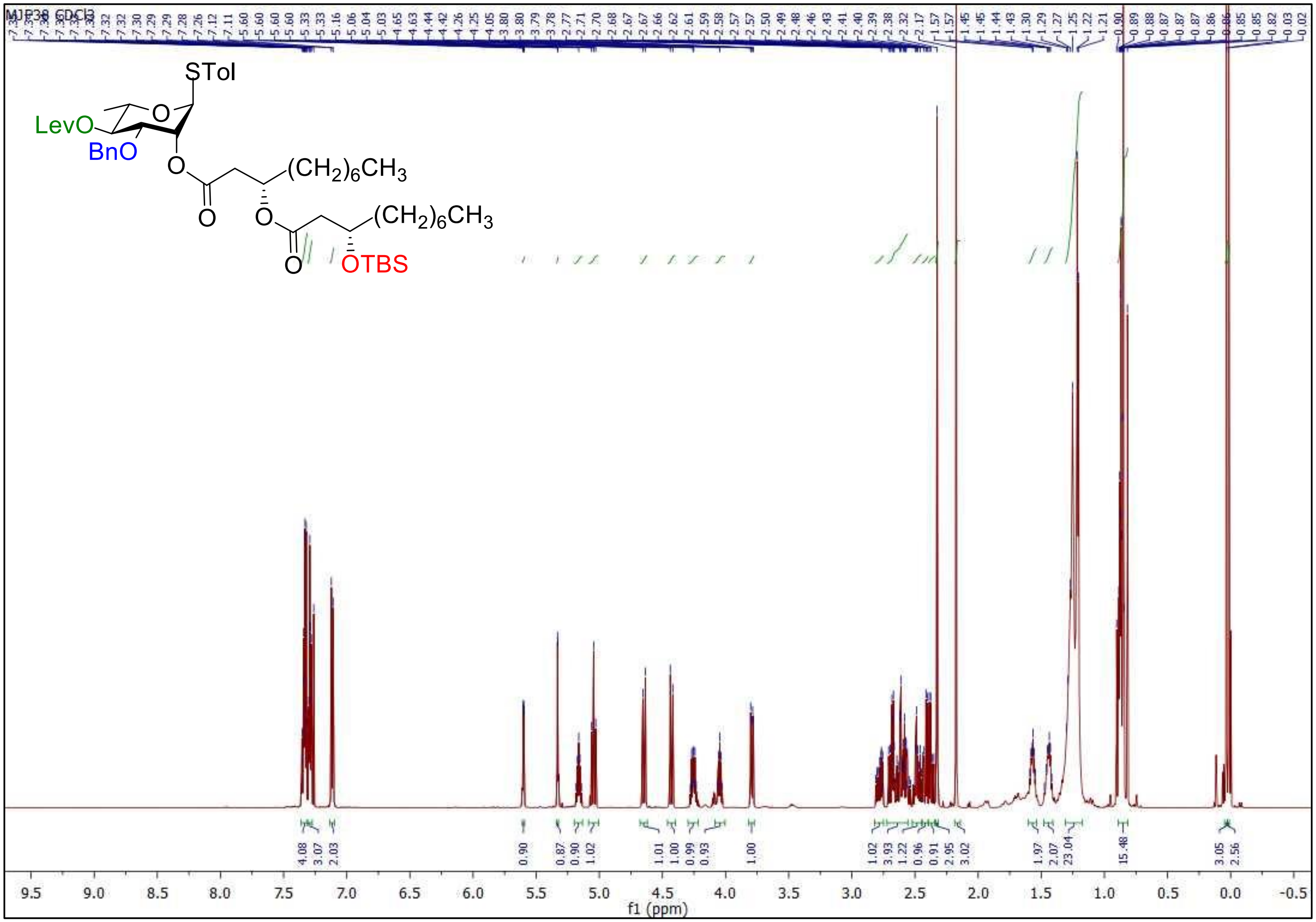

Figure S147 | COSY NMR spectrum ($CDCl_3$, 600 MHz) of *para*-methylphenyl 3-*O*-benzyl-2-*O*-(*R*)-3-(((*R*)-3-(*tert*-butyldimethylsilyl)oxy)decanoyl)oxy)decanoyl-4-*O*-levulinoyl-1-thio-*α*-L-rhamnopyranoside (**27**).

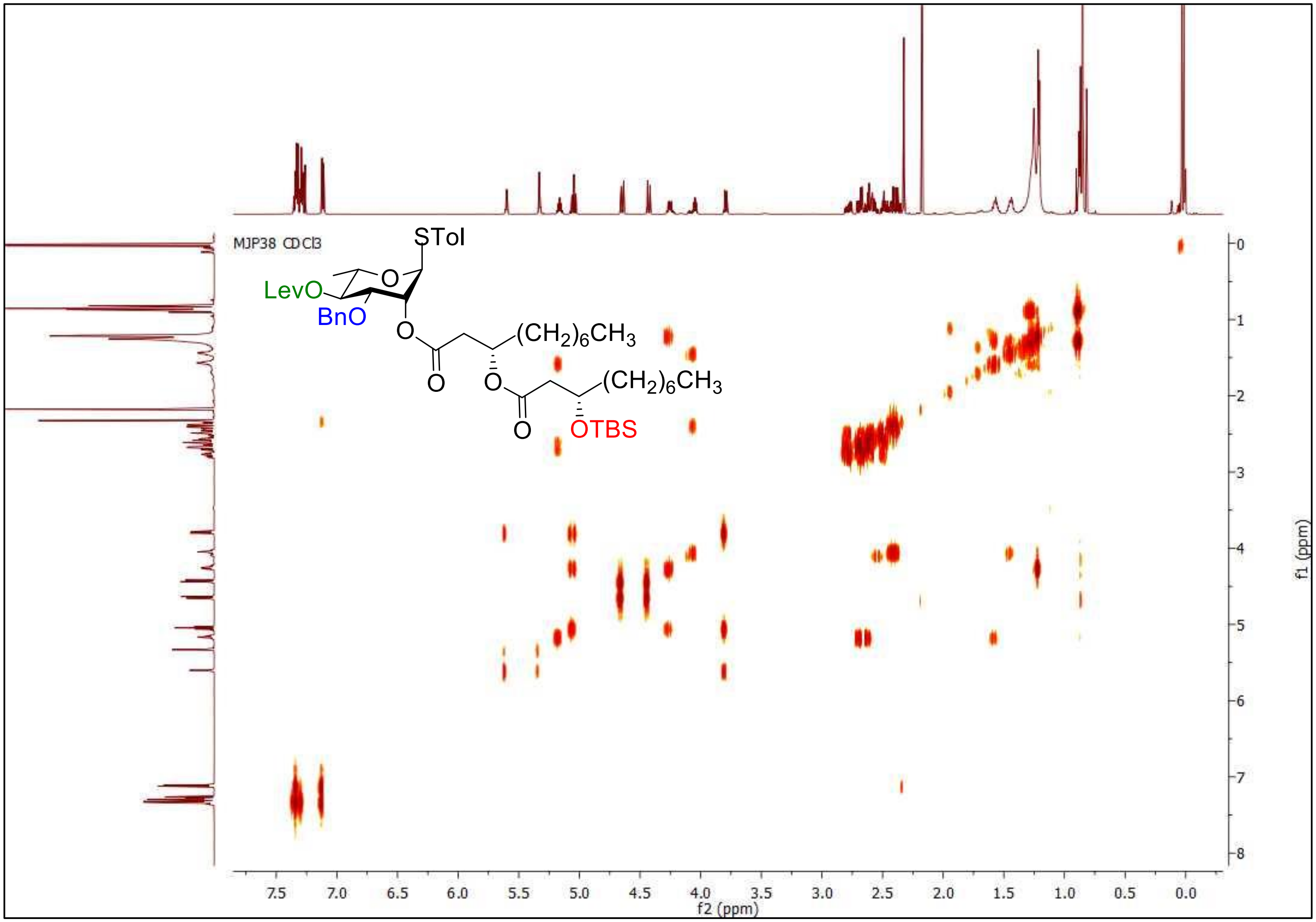

Figure S148 | ^{13}C NMR spectrum ($CDCl_3$, 600 MHz) of *para*-methylphenyl 3-*O*-benzyl-2-*O*-(*R*)-3-(((*R*)-3-(*tert*-butyldimethylsilyl)oxy)decanoyl)oxy)decanoyl-4-*O*-levulinoyl-1-thio-*α*-L-rhamnopyranoside (**27**).

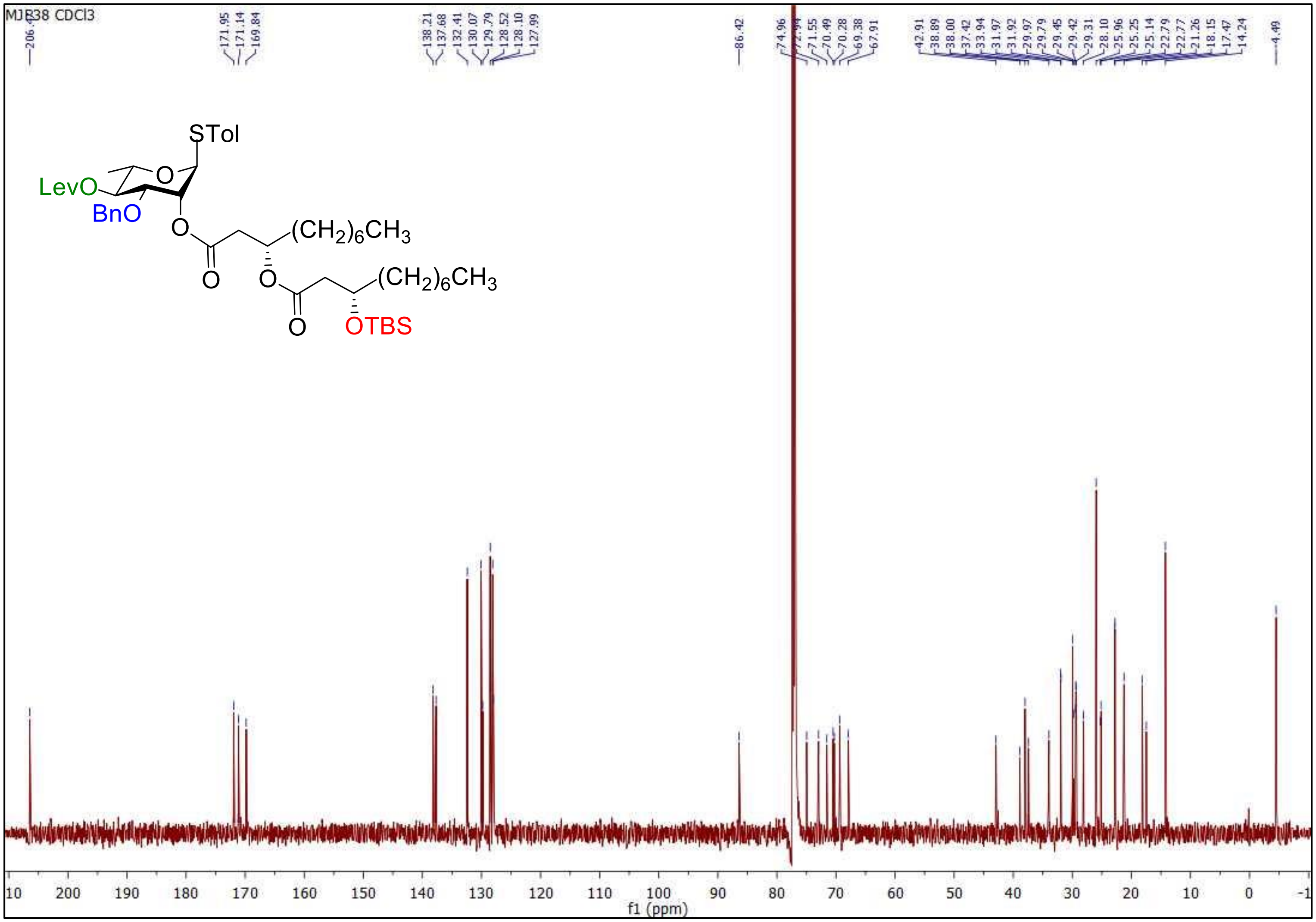

Figure S149 | HSQC NMR spectrum ($CDCl_3$, 600 MHz) of *para*-methylphenyl 3-*O*-benzyl-2-*O*-(*R*)-3-(((*R*)-3-(*tert*-butyldimethylsilyl)oxy)decanoyl)oxy)decanoyl-4-*O*-levulinoyl-1-thio-*α*-L-rhamnopyranoside (**27**).

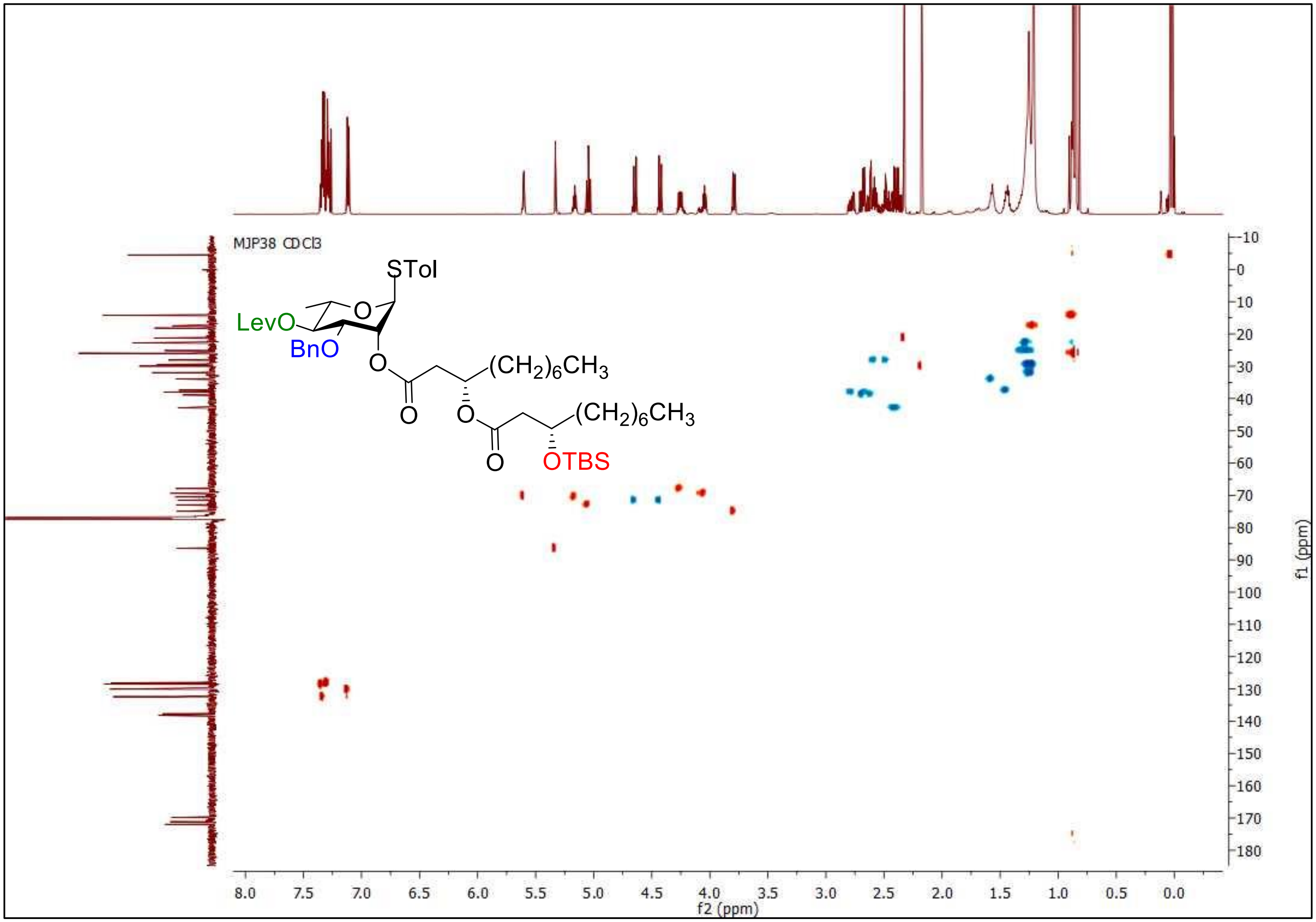

Figure S150 | ^{1}H NMR spectrum ($CDCl_3$, 600 MHz) of *para*-methylphenyl 3-*O*-benzyl-2-*O*-(*R*)-3-(((*R*)-3-(hydroxydecanoyl)oxy)decanoyl-4-*O*-levulinoyl-1-thio-*α*-L-rhamnopyranoside (**36**).

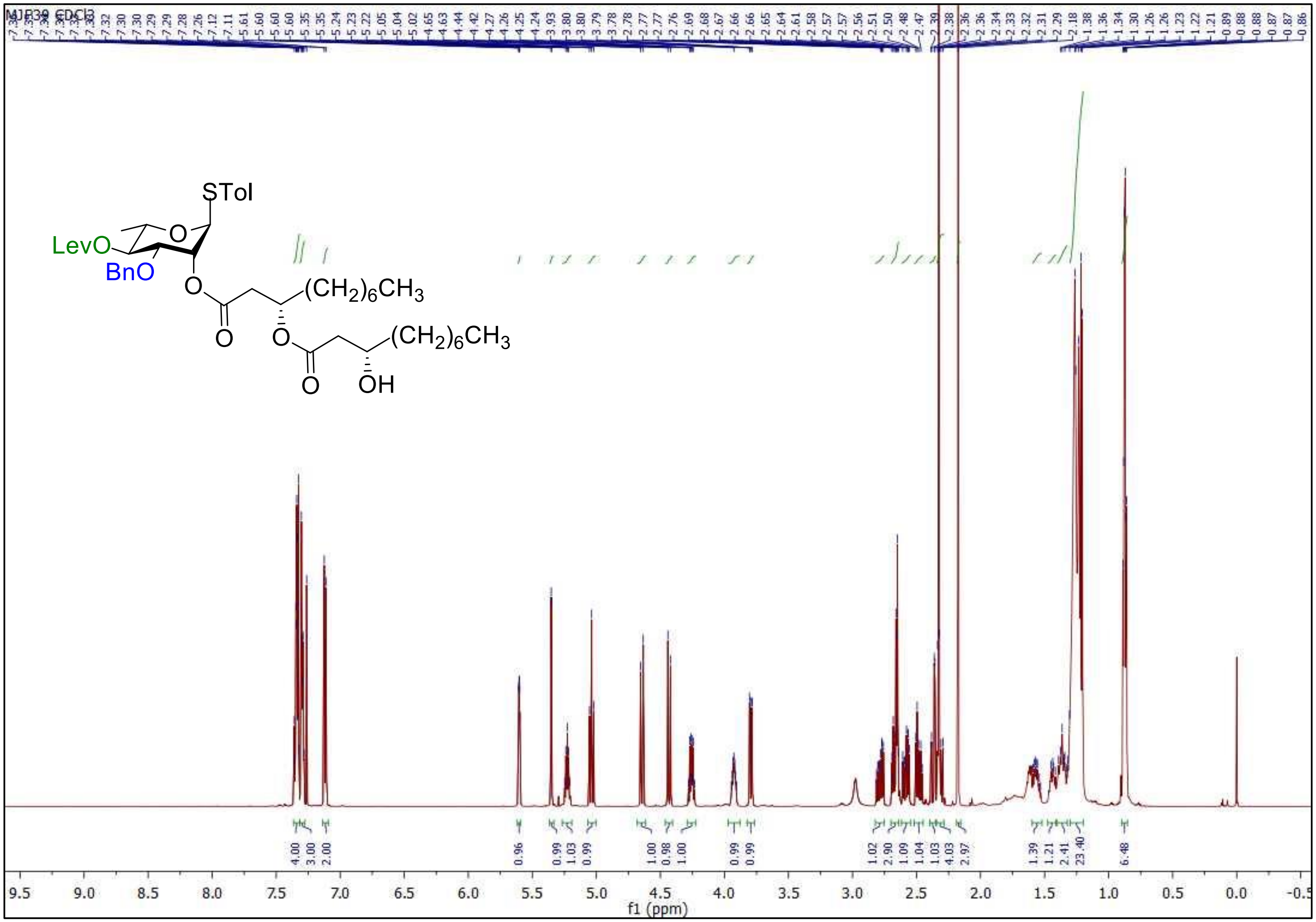

Figure S151 | COSY NMR spectrum ($CDCl_3$, 600 MHz) of *para*-methylphenyl 3-*O*-benzyl-2-*O*-(*R*)-3-(((*R*)-3-(hydroxydecanoyl)oxy)decanoyl-4-*O*-levulinoyl-1-thio-*α*-L-rhamnopyranoside (**36**).

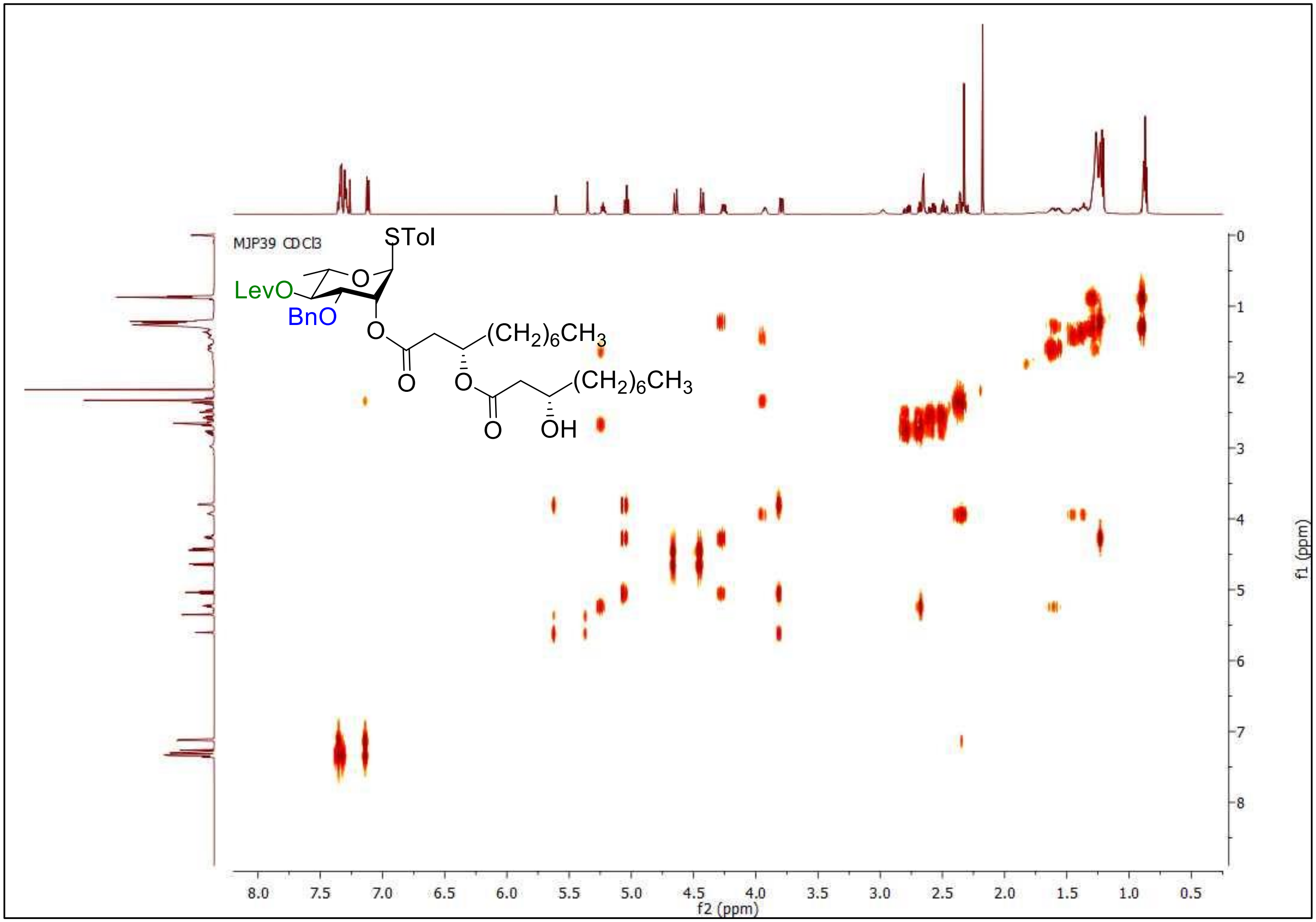

Figure S152 | ^{13}C NMR spectrum ($CDCl_3$, 600 MHz) of *para*-methylphenyl 3-*O*-benzyl-2-*O*-(*R*)-3-(((*R*)-3-(hydroxydecanoyl)oxy)decanoyl-4-*O*-levulinoyl-1-thio-*α*-L-rhamnopyranoside (**36**).

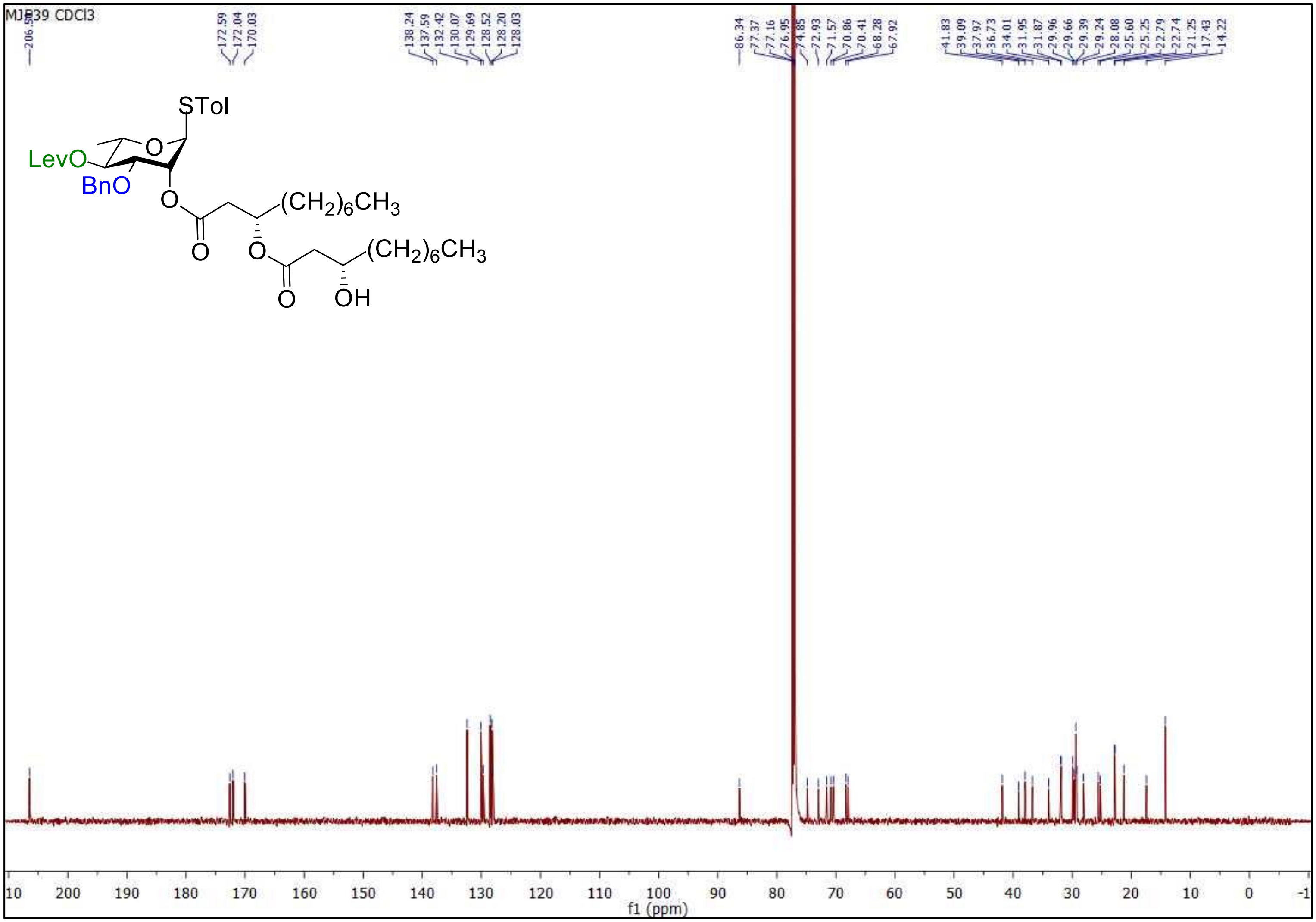

Figure S153 | HSQC NMR spectrum (CDCl₃, 600 MHz) of *para*-methylphenyl 3-*O*-benzyl-2-*O*-(*R*)-3-(((*R*)-3-(hydroxydecanoyl)oxy)decanoyl-4-*O*-levulinoyl-1-thio-*α*-L-rhamnopyranoside (**36**).

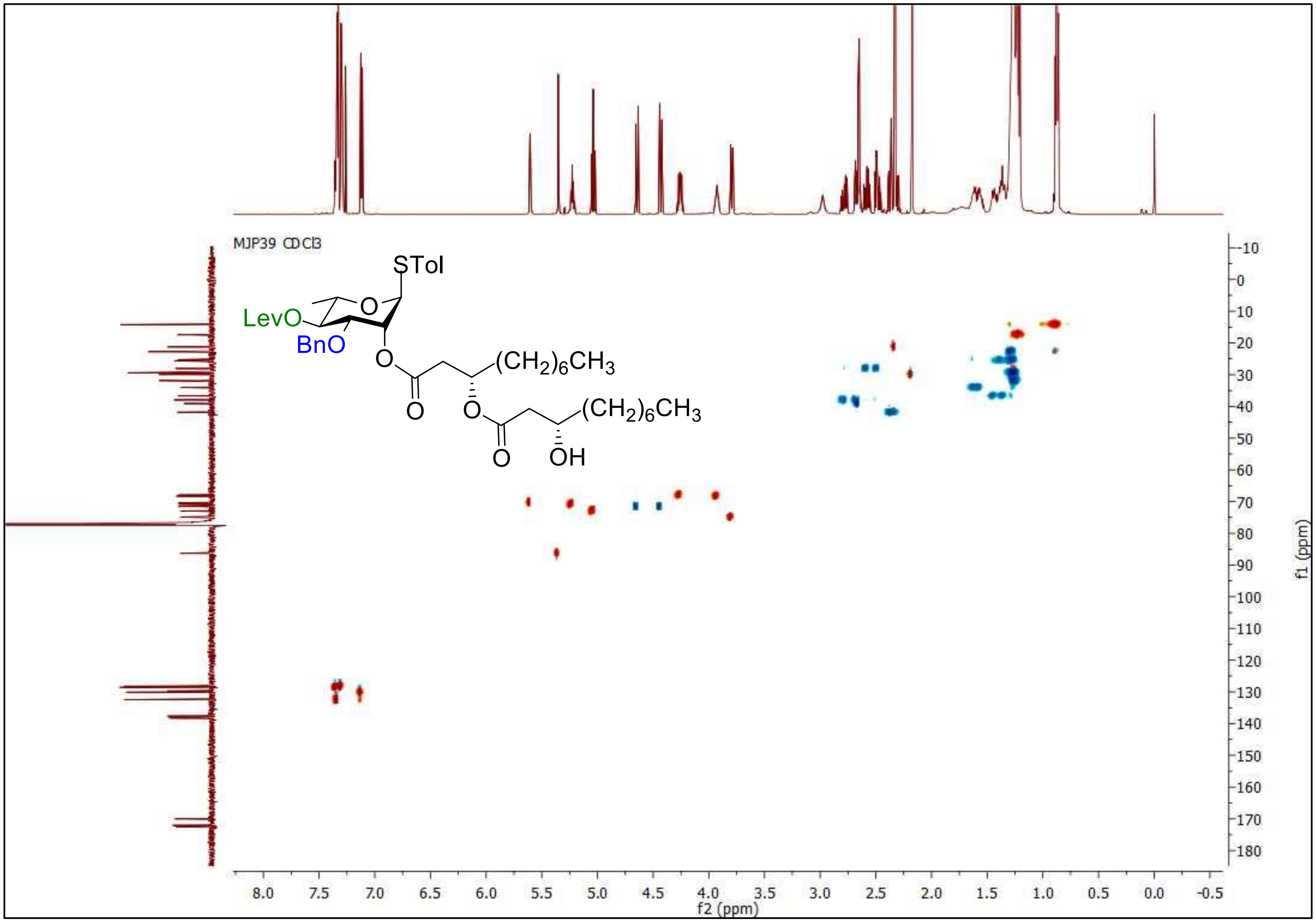

Figure S154 | ^{1}H NMR spectrum ($CDCl_3$, 600 MHz) of macrolide **37**.

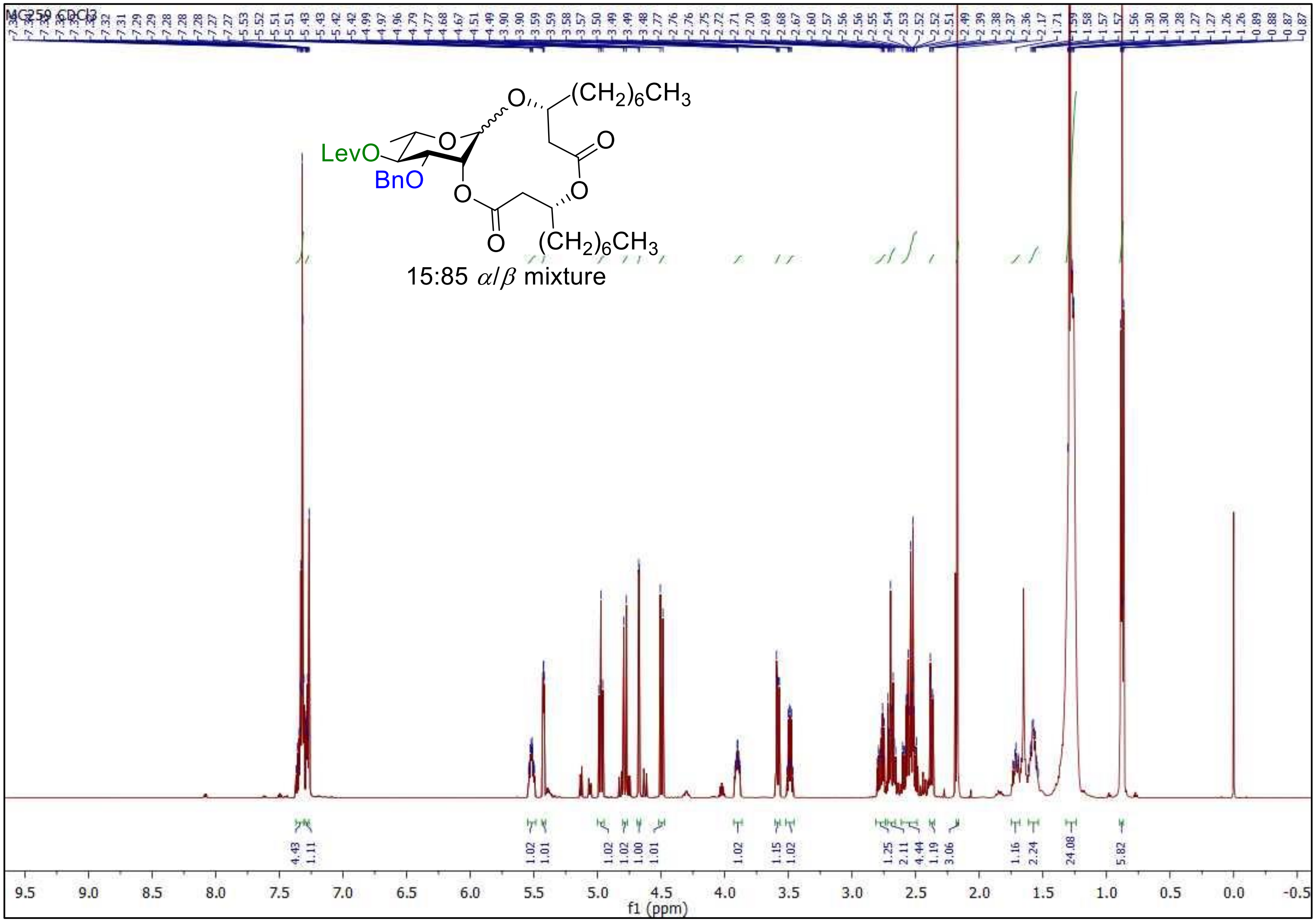

Figure S155 | COSY NMR spectrum ($CDCl_3$, 600 MHz) of macrolide **37**.

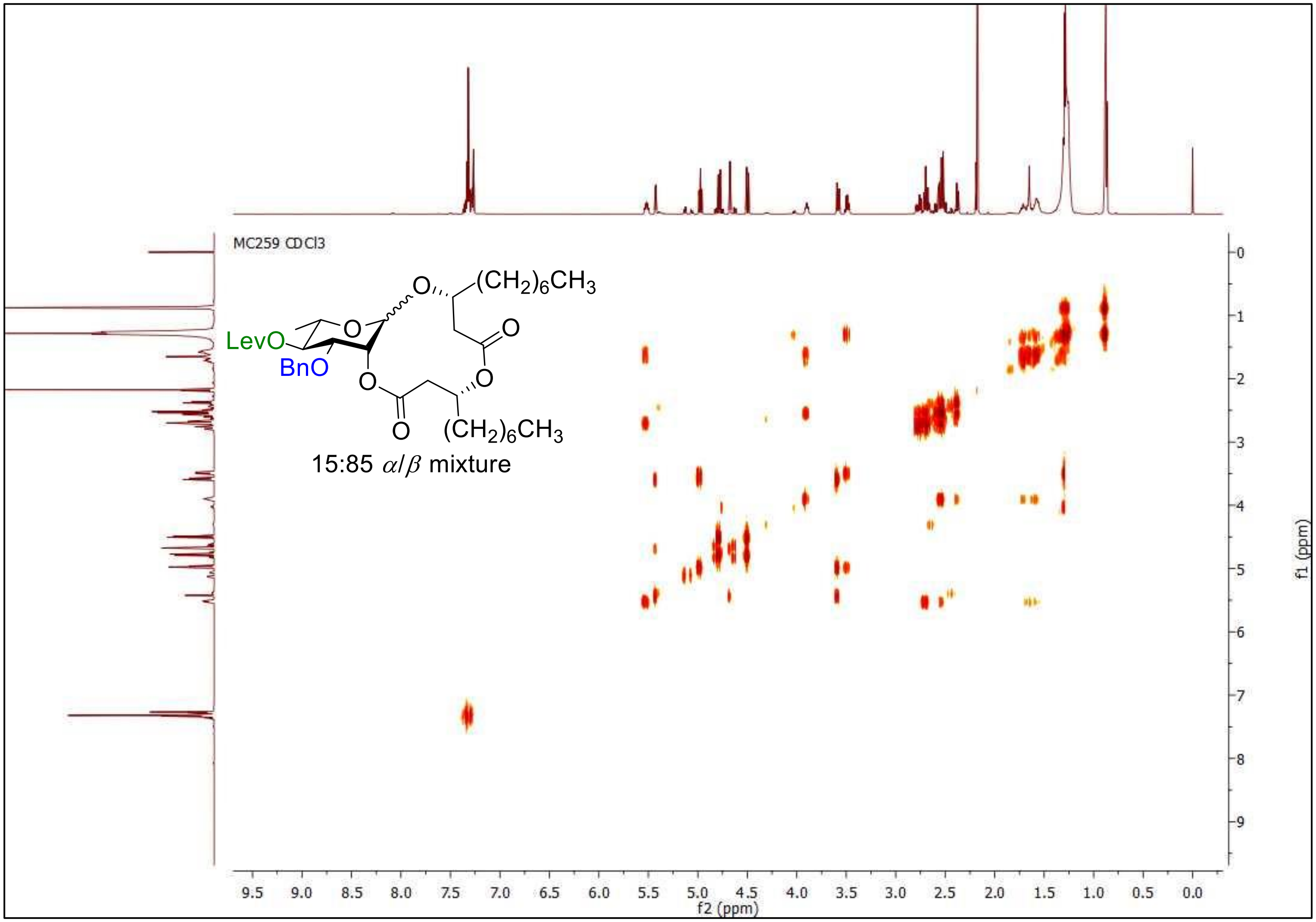

Figure S156 | ^{13}C NMR spectrum (CDCl$_3$, 600 MHz) of macrolide **37**.

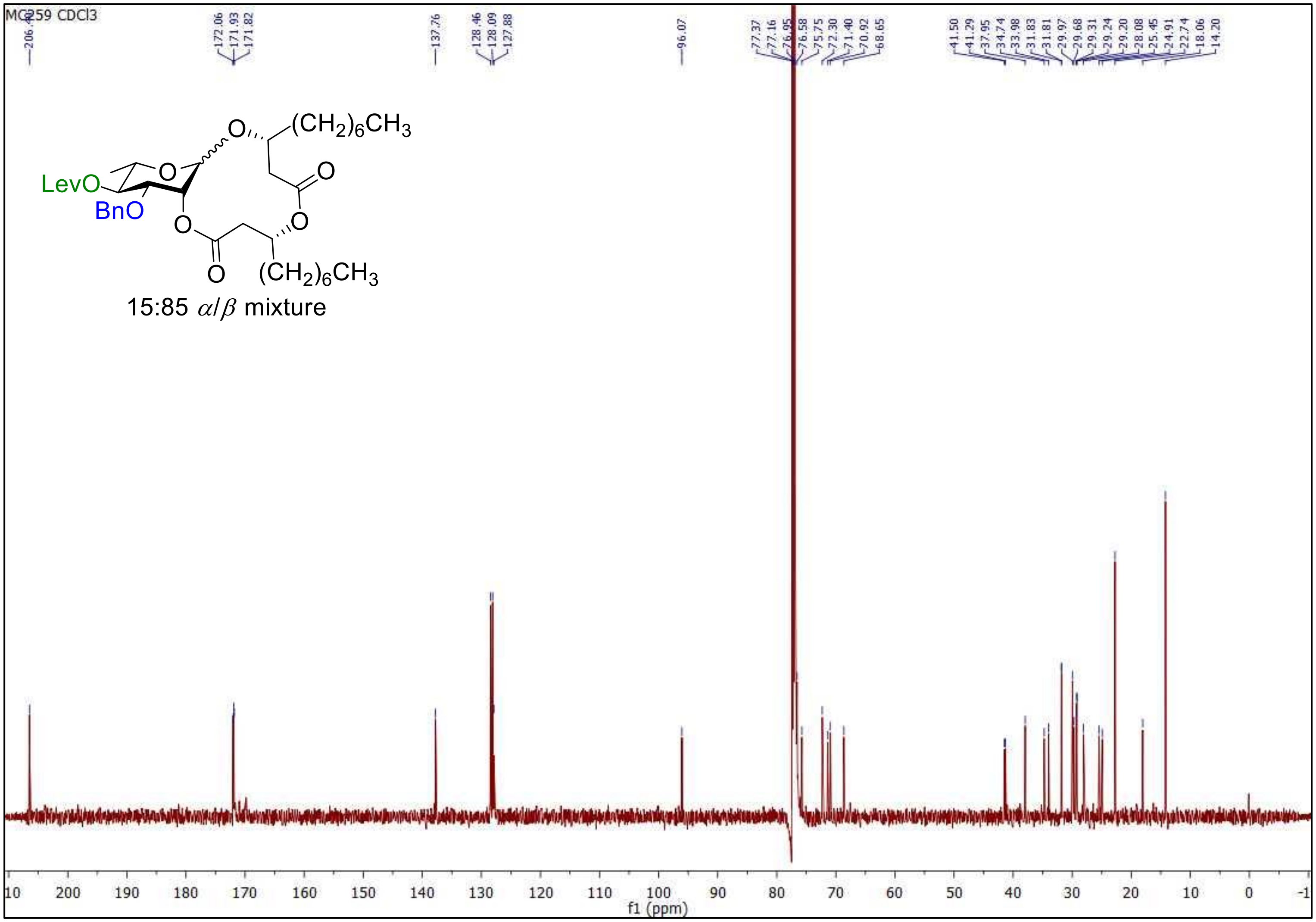

Figure S157 | HSQC NMR spectrum ($CDCl_3$, 600 MHz) of macrolide **37**.

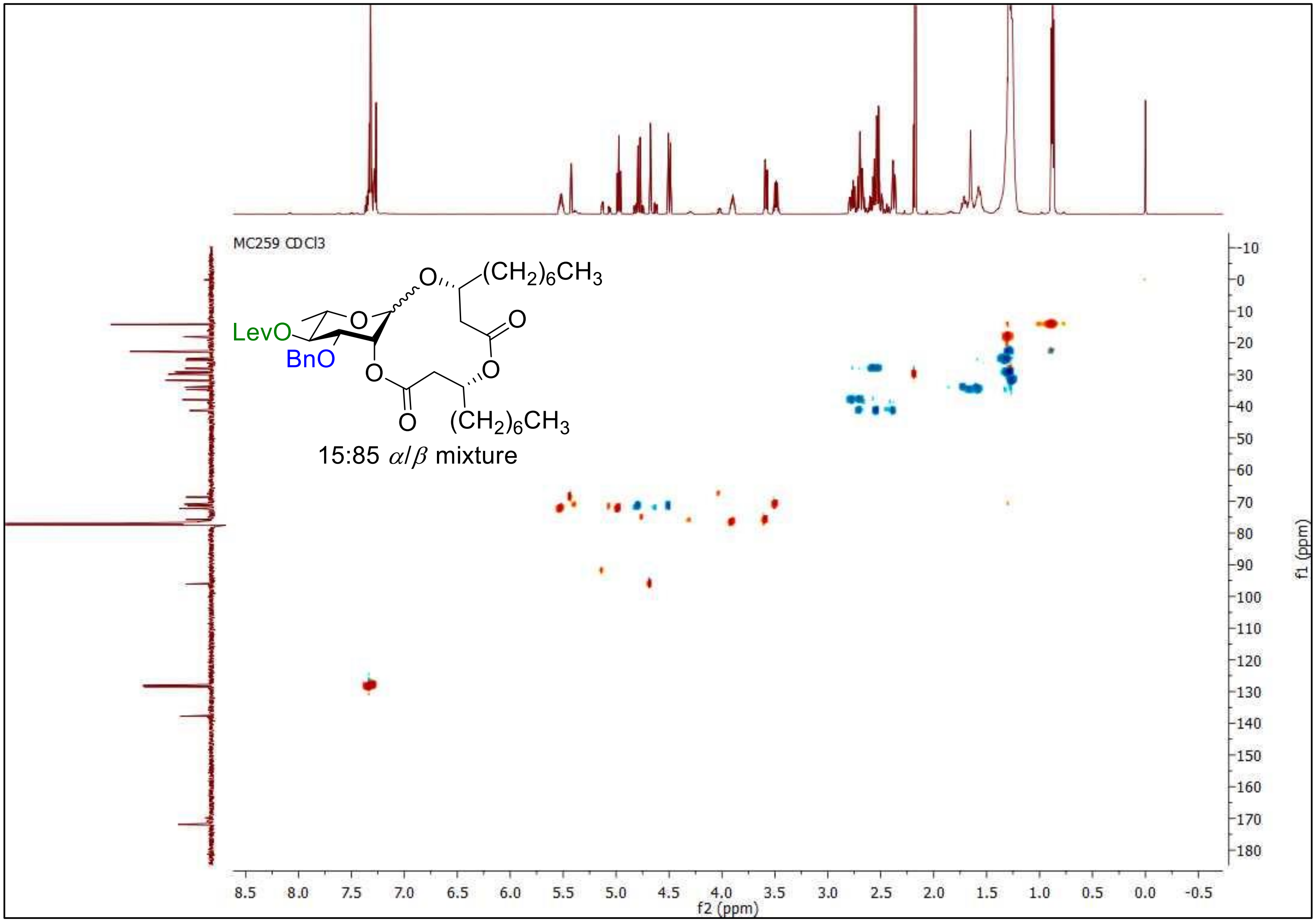

Figure S158 | undecoupled HSQC NMR spectrum ($CDCl_3$, 600 MHz) of macrolide **37**.

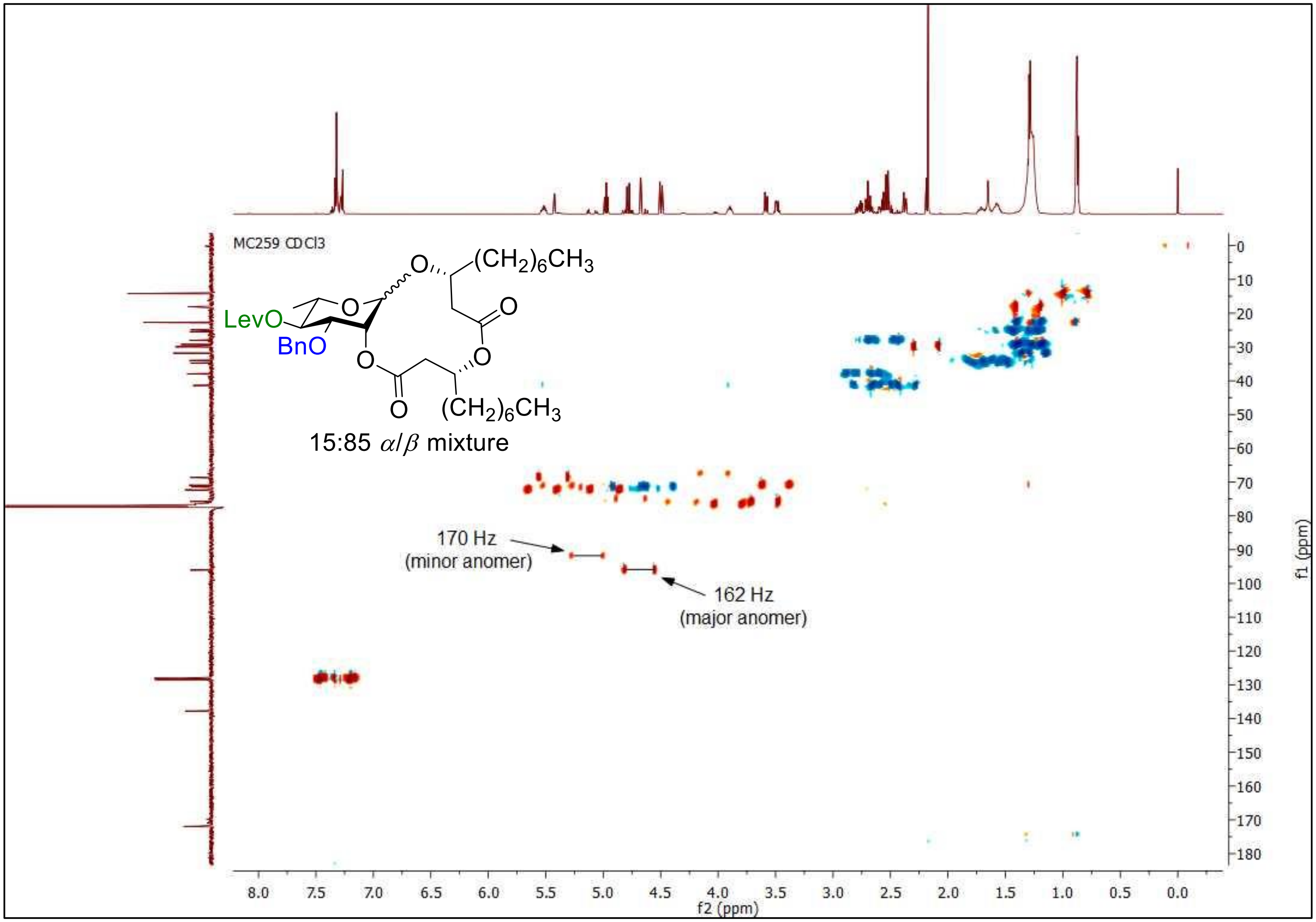

Figure S159 | ^{1}H NMR spectrum ($CDCl_3$, 600 MHz) of macrolide **S17*β***.

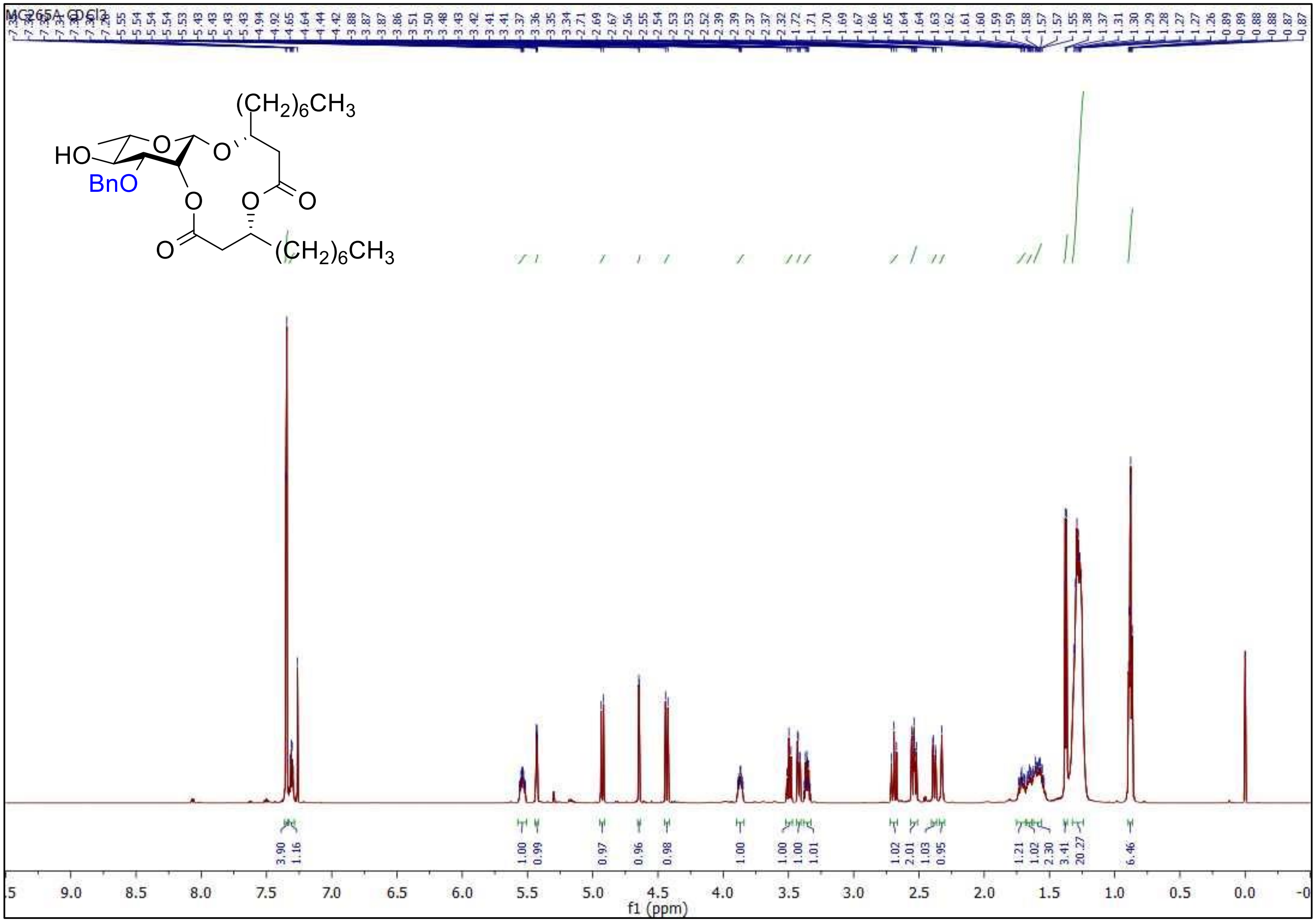

Figure S160 | COSY NMR spectrum ($CDCl_3$, 600 MHz) of macrolide **S17*β***.

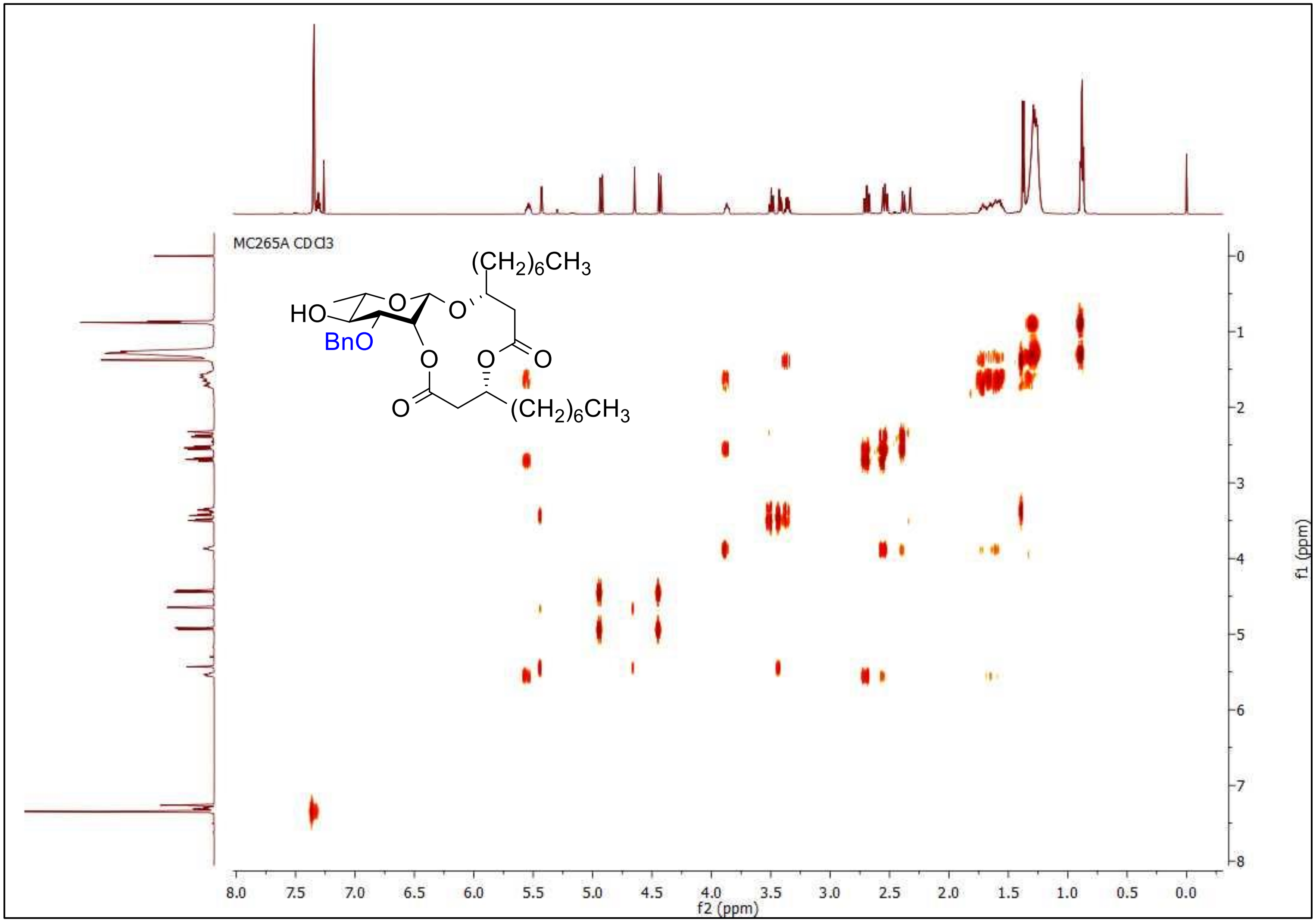

Figure S161 | ^{13}C NMR spectrum ($CDCl_3$, 600 MHz) of macrolide **S17*β***.

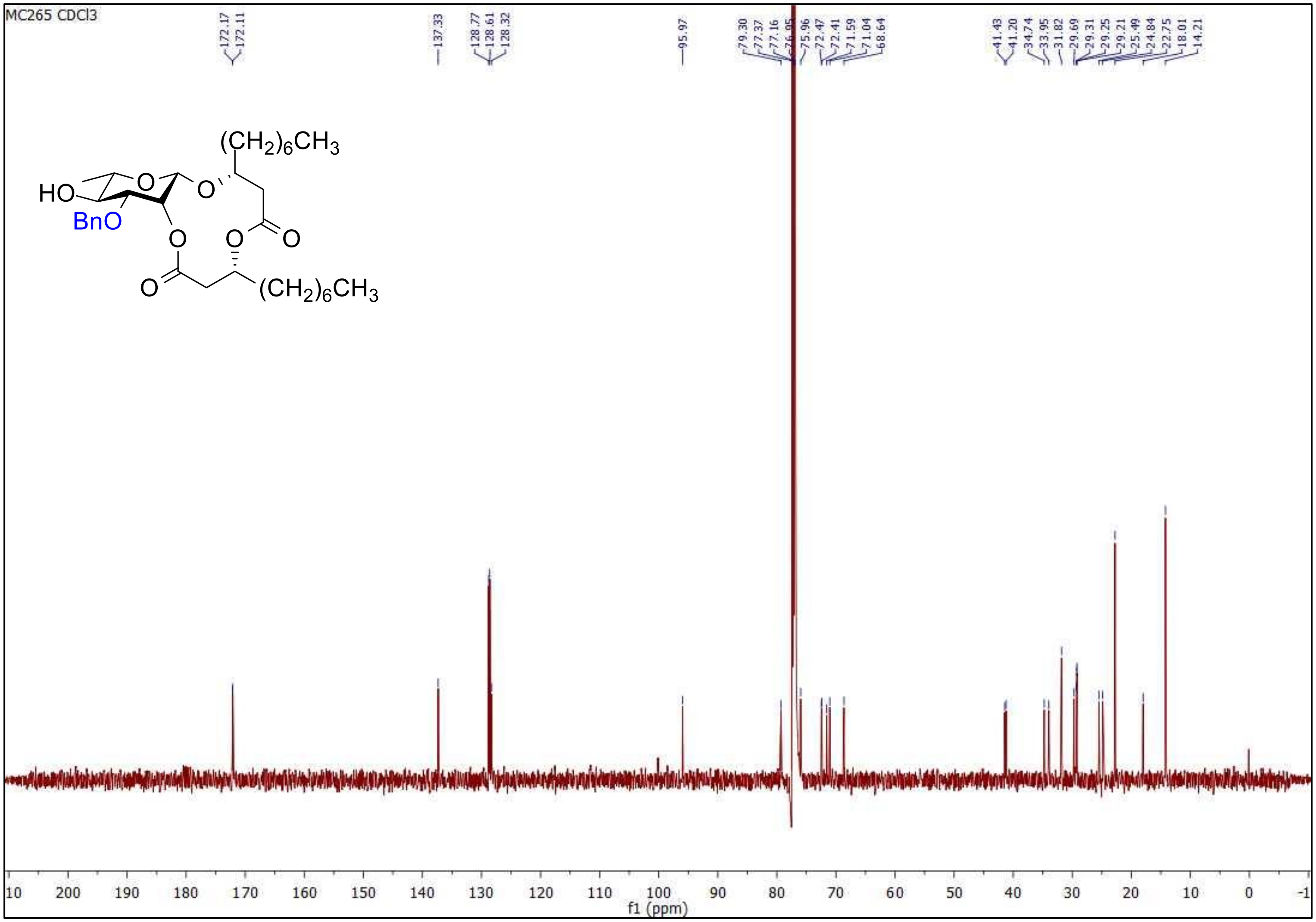

Figure S162 | HSQC NMR spectrum ($CDCl_3$, 600 MHz) of macrolide **S17*β***.

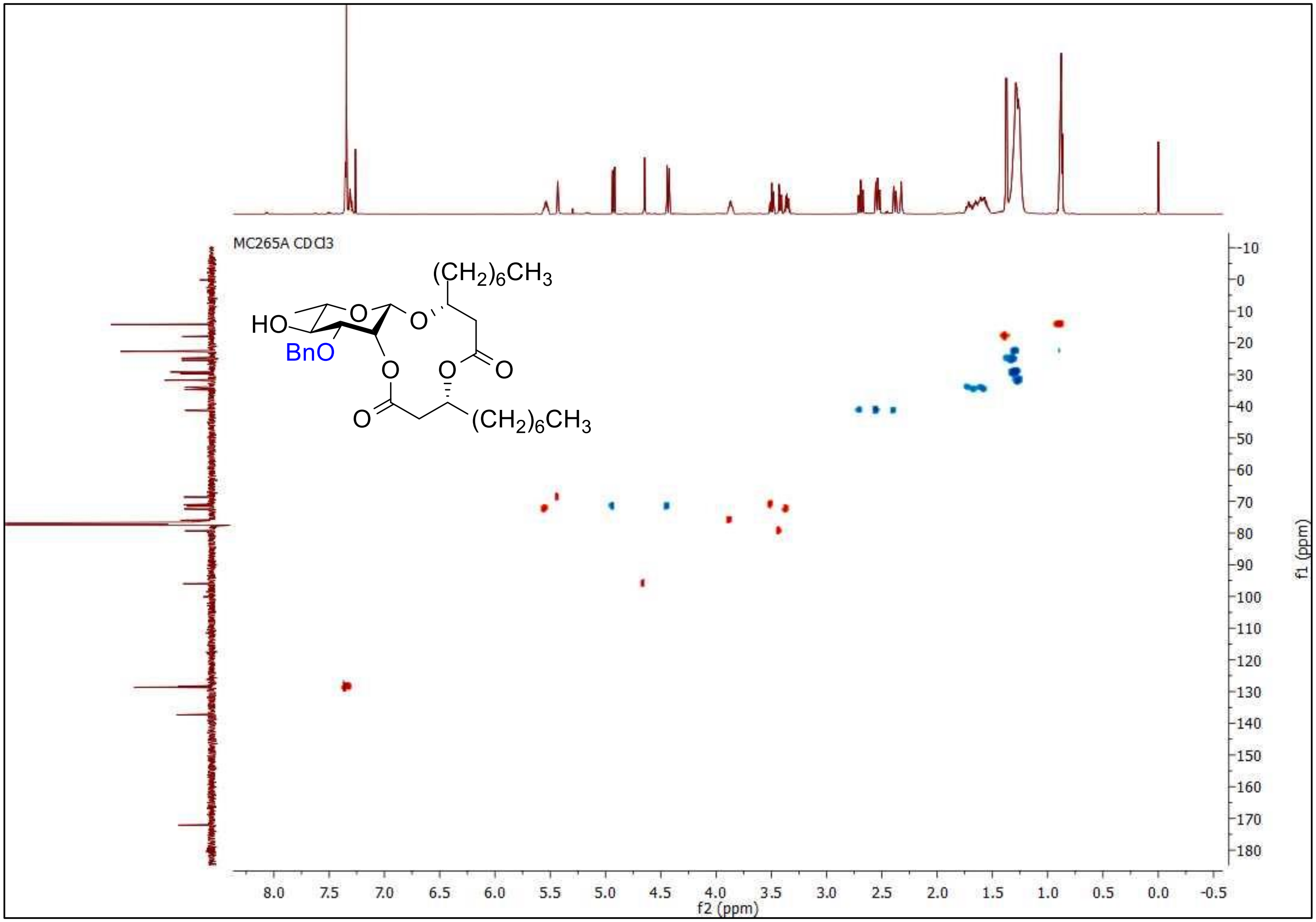

Figure S163 | undecoupled HSQC NMR spectrum ($CDCl_3$, 600 MHz) of macrolide **S17*β***.

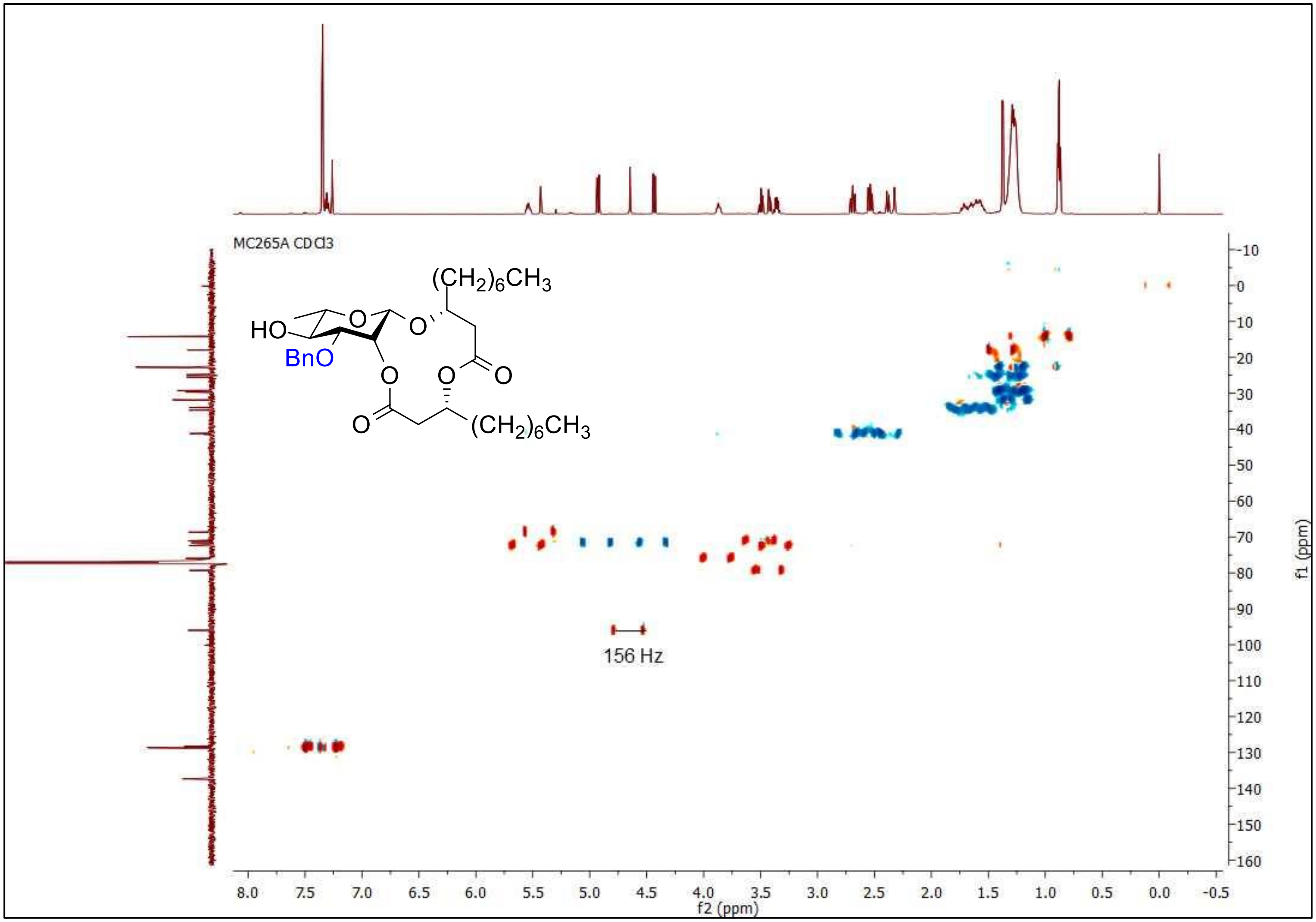

Figure S164 | ^{1}H NMR spectrum ($CDCl_3$, 600 MHz) of (1→2)-macrolactonized rhamnolipid **5*β***.

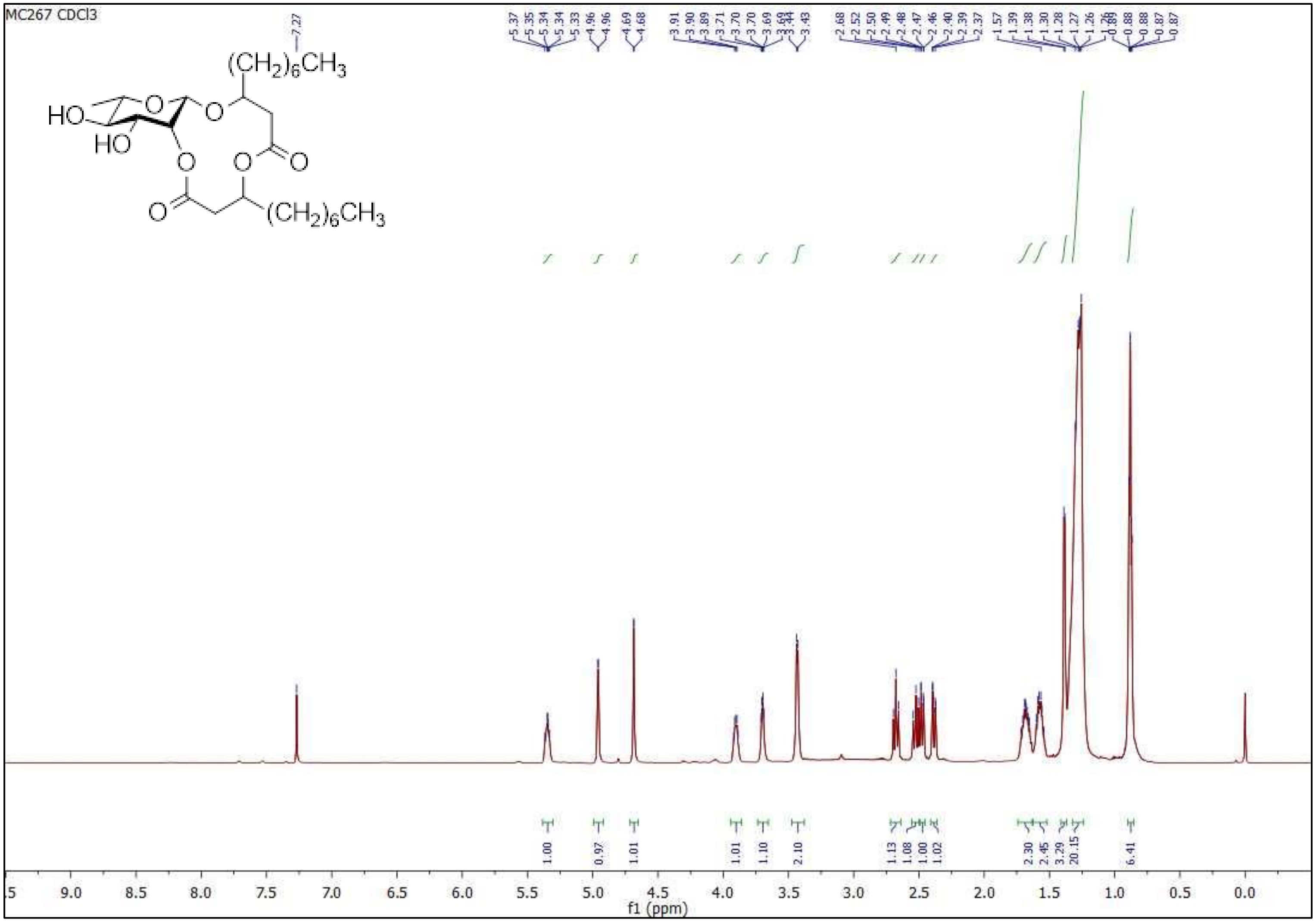

Figure S165 | COSY NMR spectrum ($CDCl_3$, 600 MHz) of (1→2)-macrolactonized rhamnolipid ***5β***.

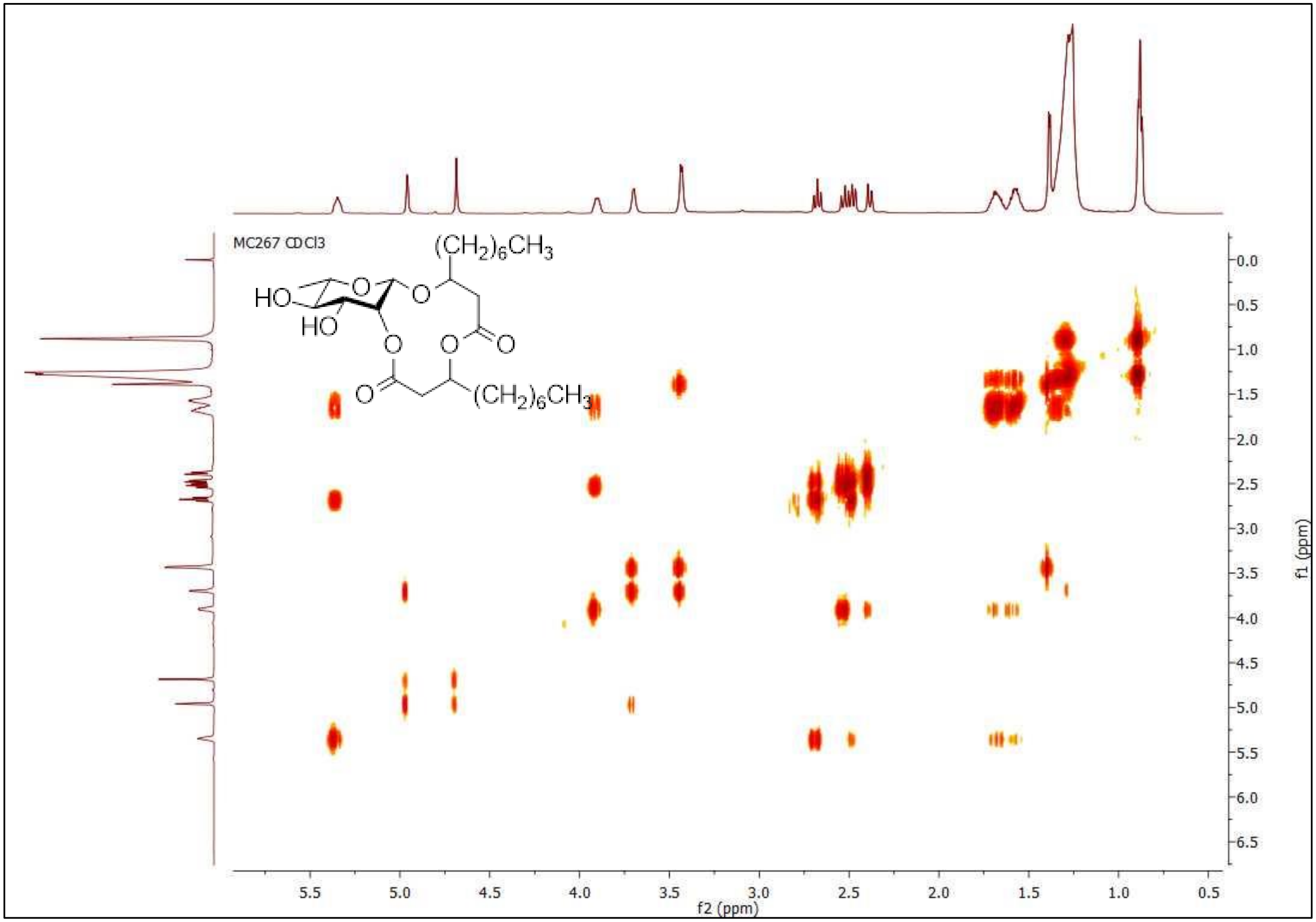

Figure S166 | ^{13}C NMR spectrum ($CDCl_3$, 600 MHz) of (1→2)-macrolactonized rhamnolipid **5*β***.

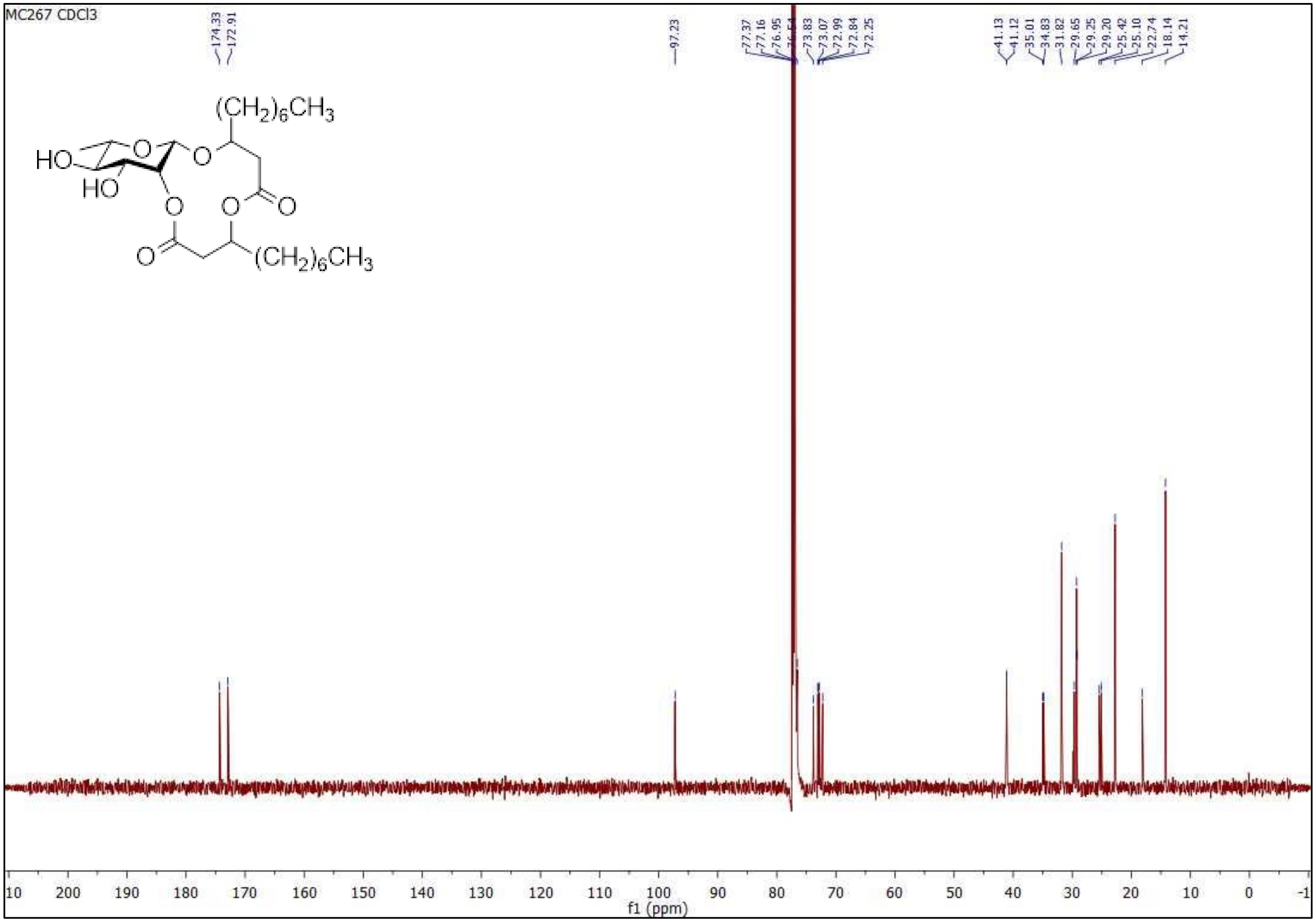

Figure S167 | HSQC NMR spectrum ($CDCl_3$, 600 MHz) of (1→2)-macrolactonized rhamnolipid **5*β***.

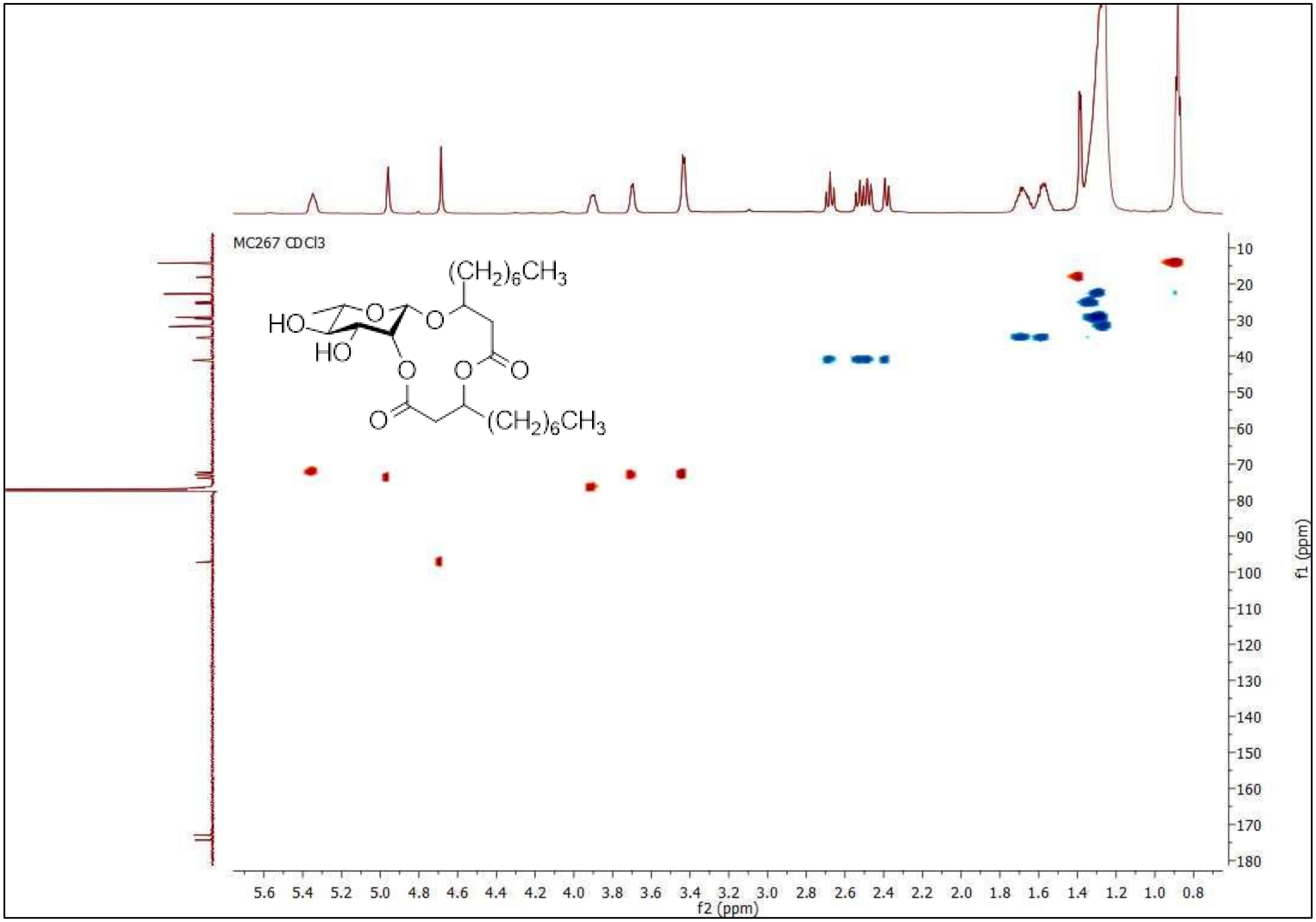

Figure S168 | ^{1}H NMR spectrum ($CDCl_3$, 600 MHz) of (1→2)-macrolactonized rhamnolipid **5*α***.

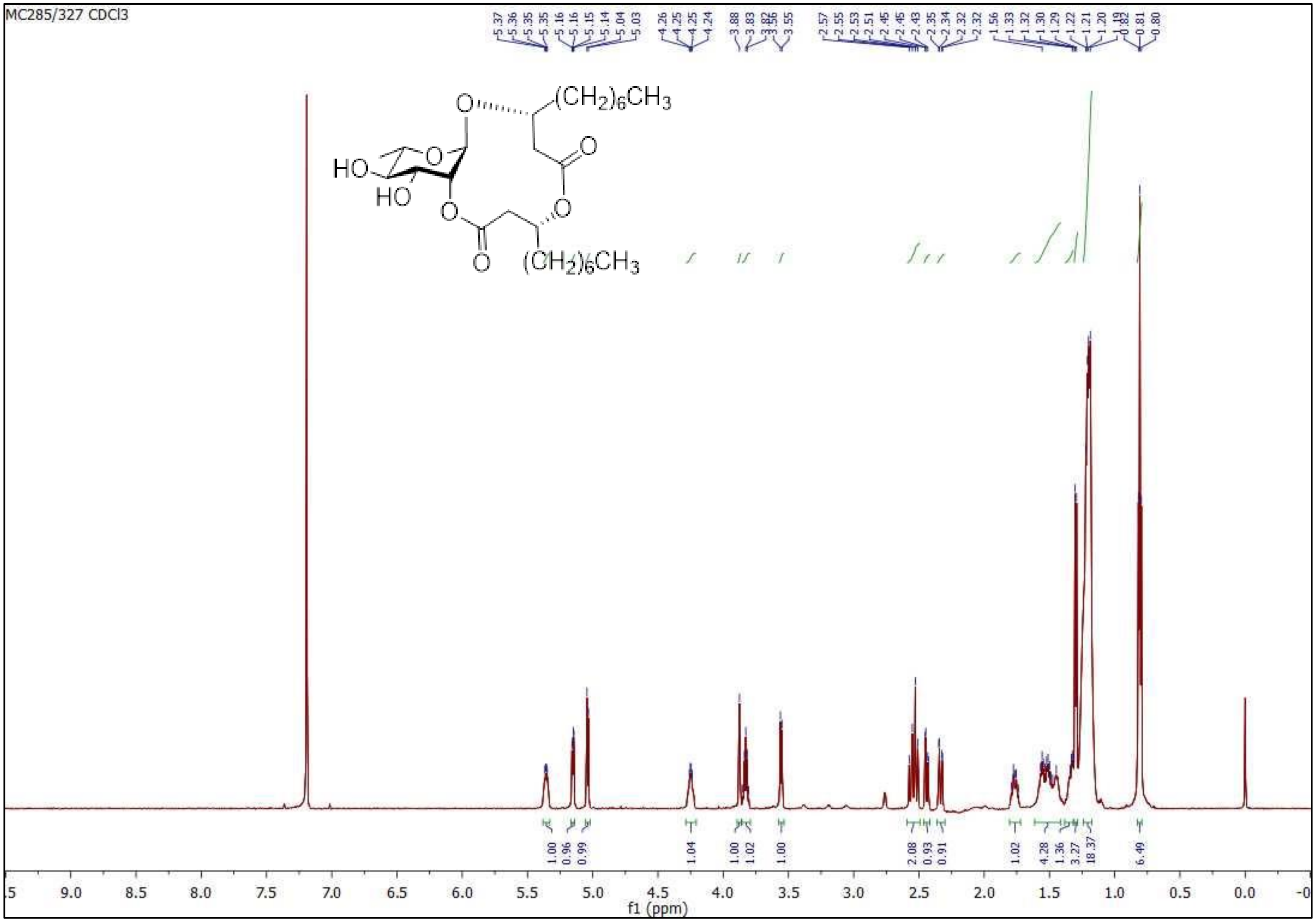

Figure S169 | COSY NMR spectrum ($CDCl_3$, 600 MHz) of (1→2)-macrolactonized rhamnolipid **5*α***.

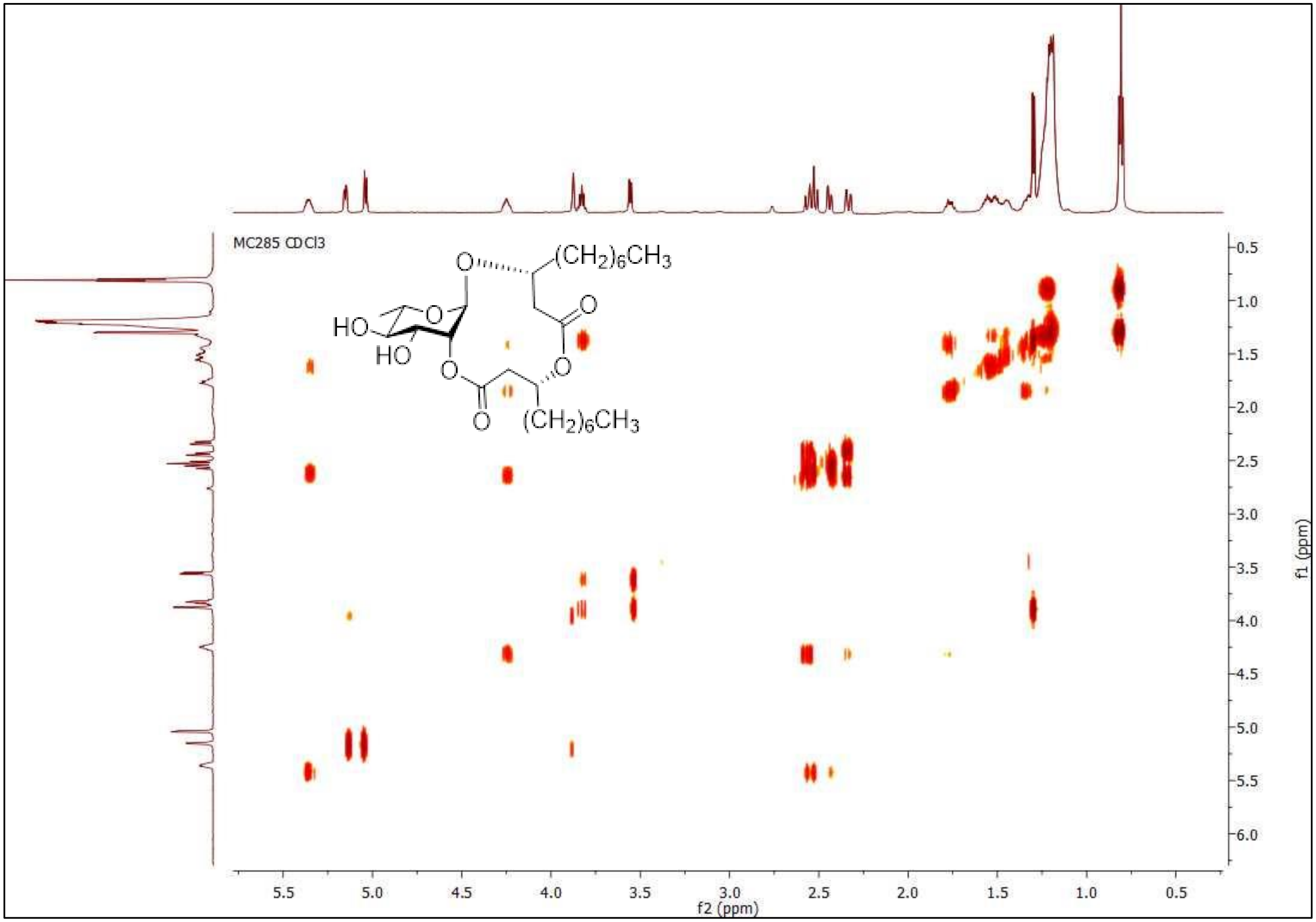

Figure S170 | ^{13}C NMR spectrum ($CDCl_3$, 600 MHz) of (1→2)-macrolactonized rhamnolipid **5*α***.

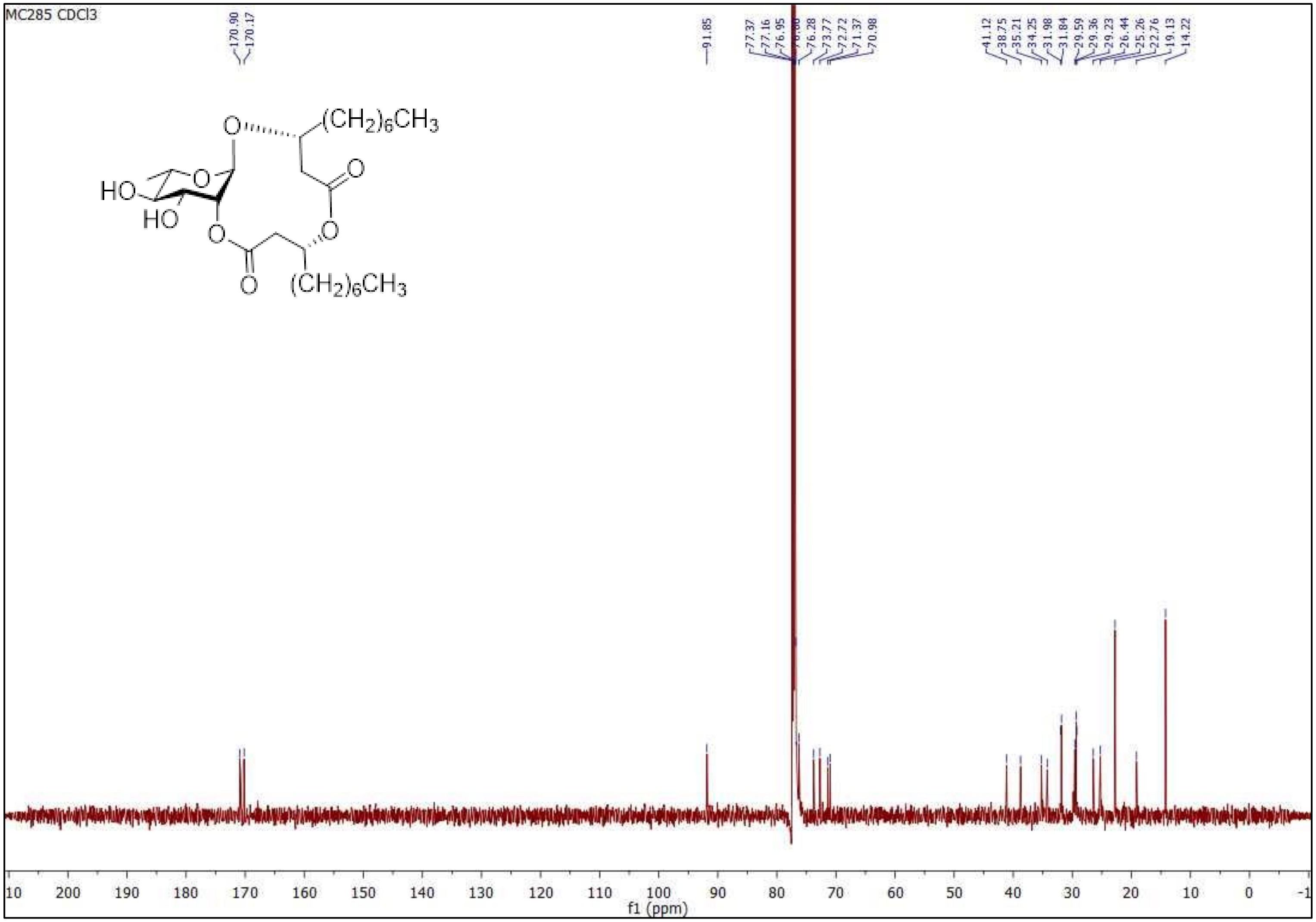

Figure S171 | HSQC NMR spectrum ($CDCl_3$, 600 MHz) of (1→2)-macrolactonized rhamnolipid **5*α***.

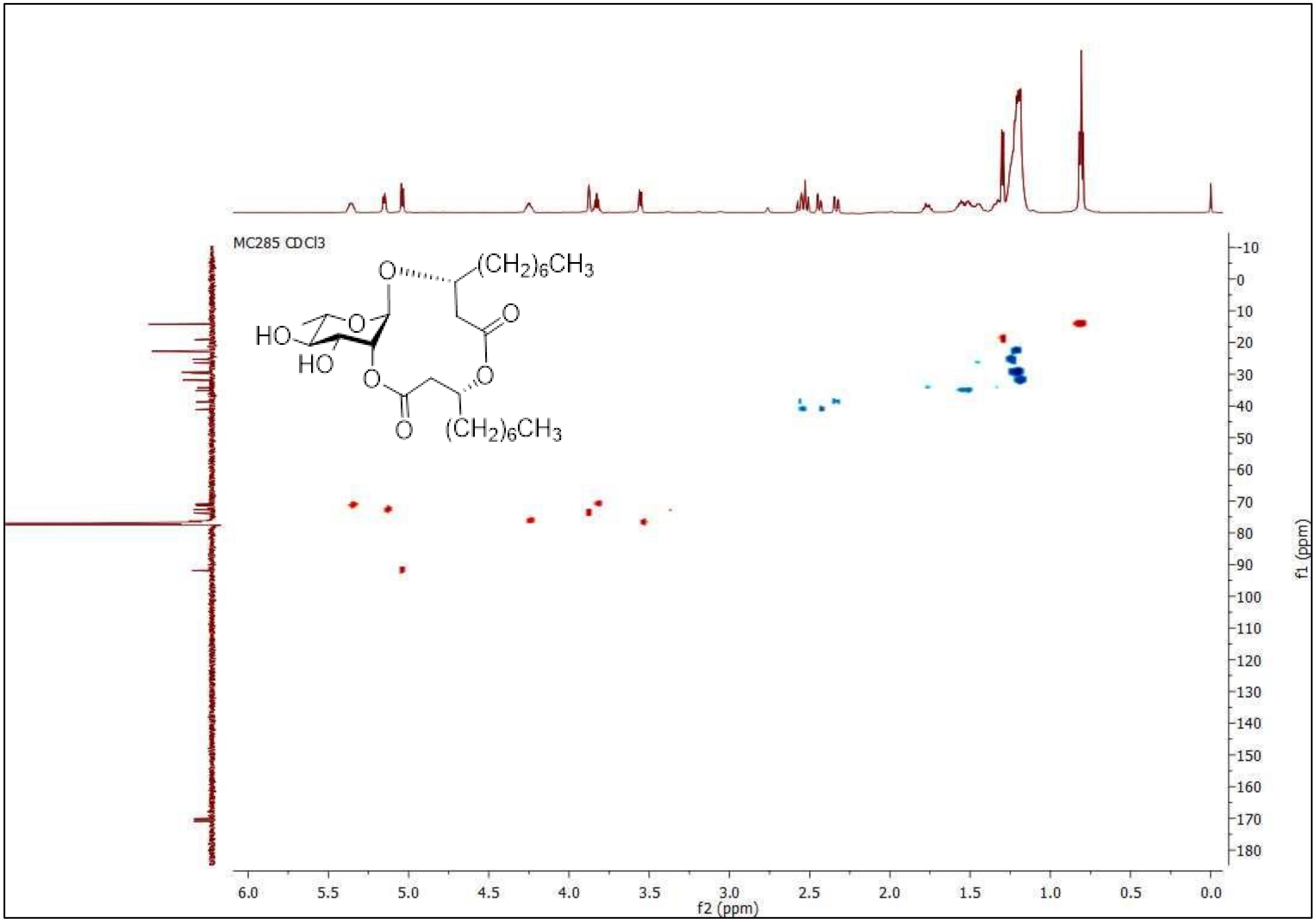

Figure S172 | ^{1}H NMR spectrum ($CDCl_3$, 600 MHz) of *para*-methylphenyl 2-*O*-*ortho*-(azidomethyl)benzoyl-4-*O*-levulinoyl-1-thio-*α*-L-rhamnopyranoside (**31**).

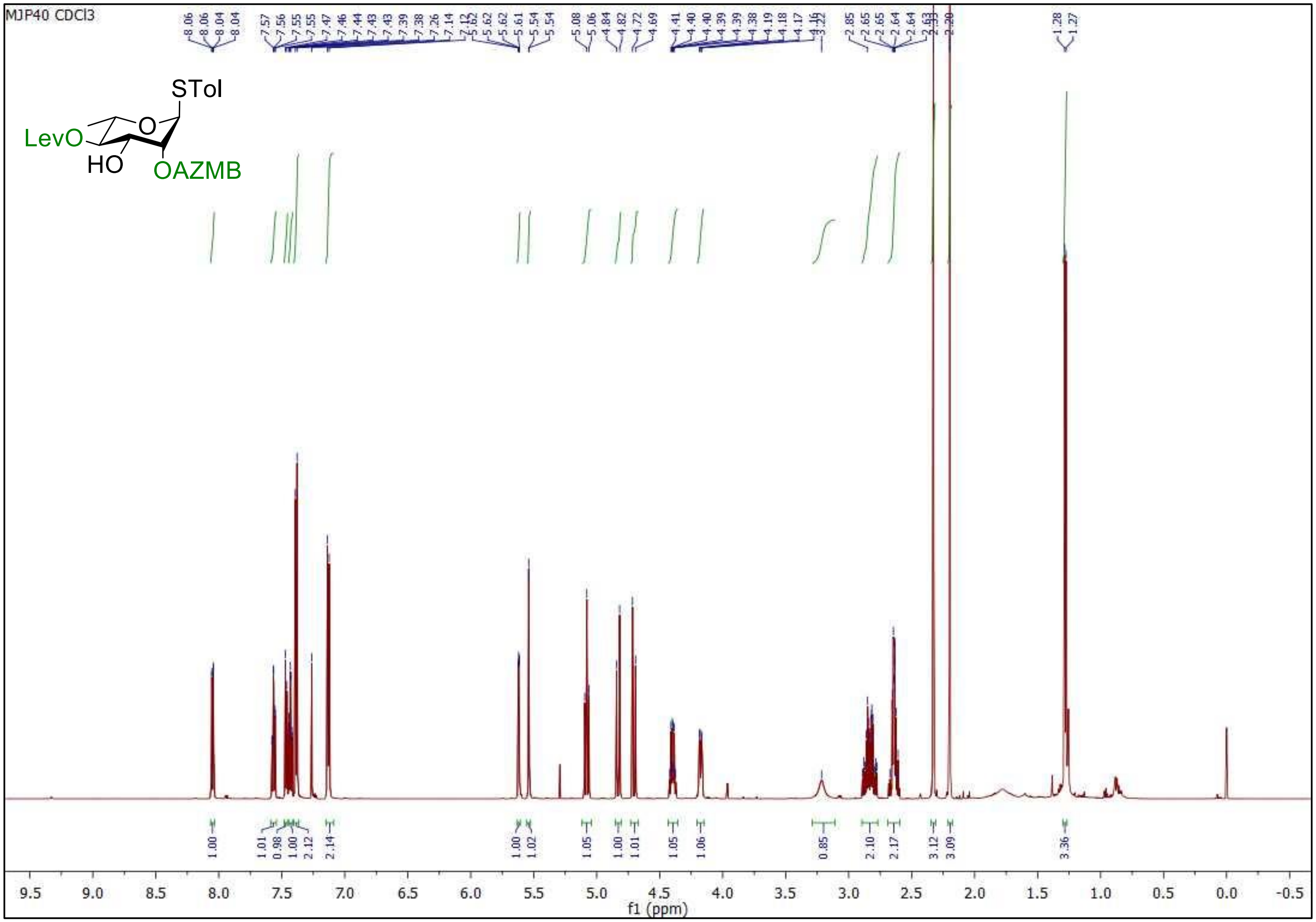

Figure S173 | COSY NMR spectrum ($CDCl_3$, 600 MHz) of *para*-methylphenyl 2-*O*-*ortho*-(azidomethyl)benzoyl-4-*O*-levulinoyl-1-thio-*α*-L-rhamnopyranoside (**31**).

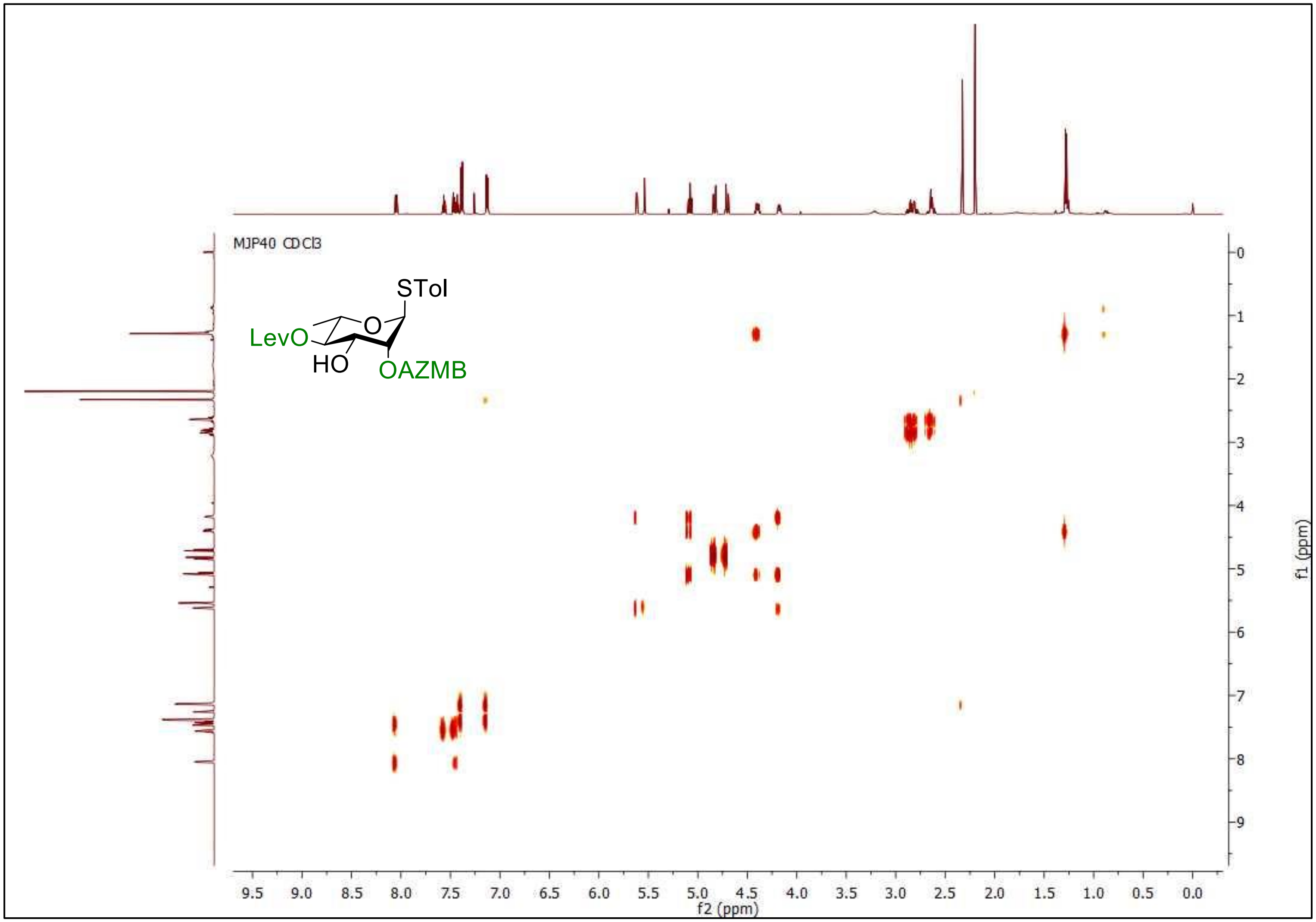

Figure S174 | ^{13}C NMR spectrum ($CDCl_3$, 600 MHz) of *para*-methylphenyl 2-*O*-*ortho*-(azidomethyl)benzoyl-4-*O*-levulinoyl-1-thio-*α*-L-rhamnopyranoside (**31**).

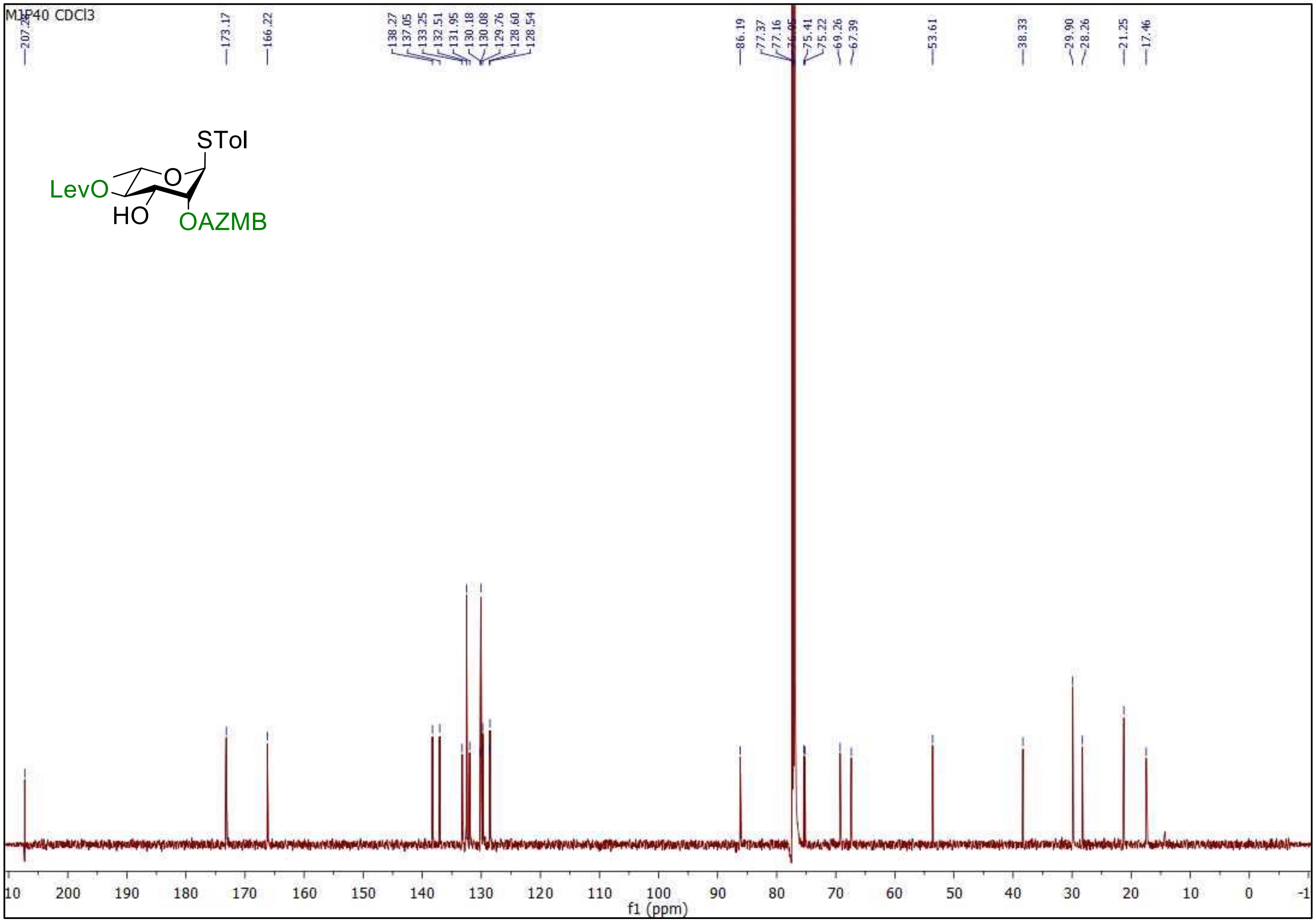

Figure S175 | HSQC NMR spectrum ($CDCl_3$, 600 MHz) of *para*-methylphenyl 2-*O*-*ortho*-(azidomethyl)benzoyl-4-*O*-levulinoyl-1-thio-*α*-L-rhamnopyranoside (**31**).

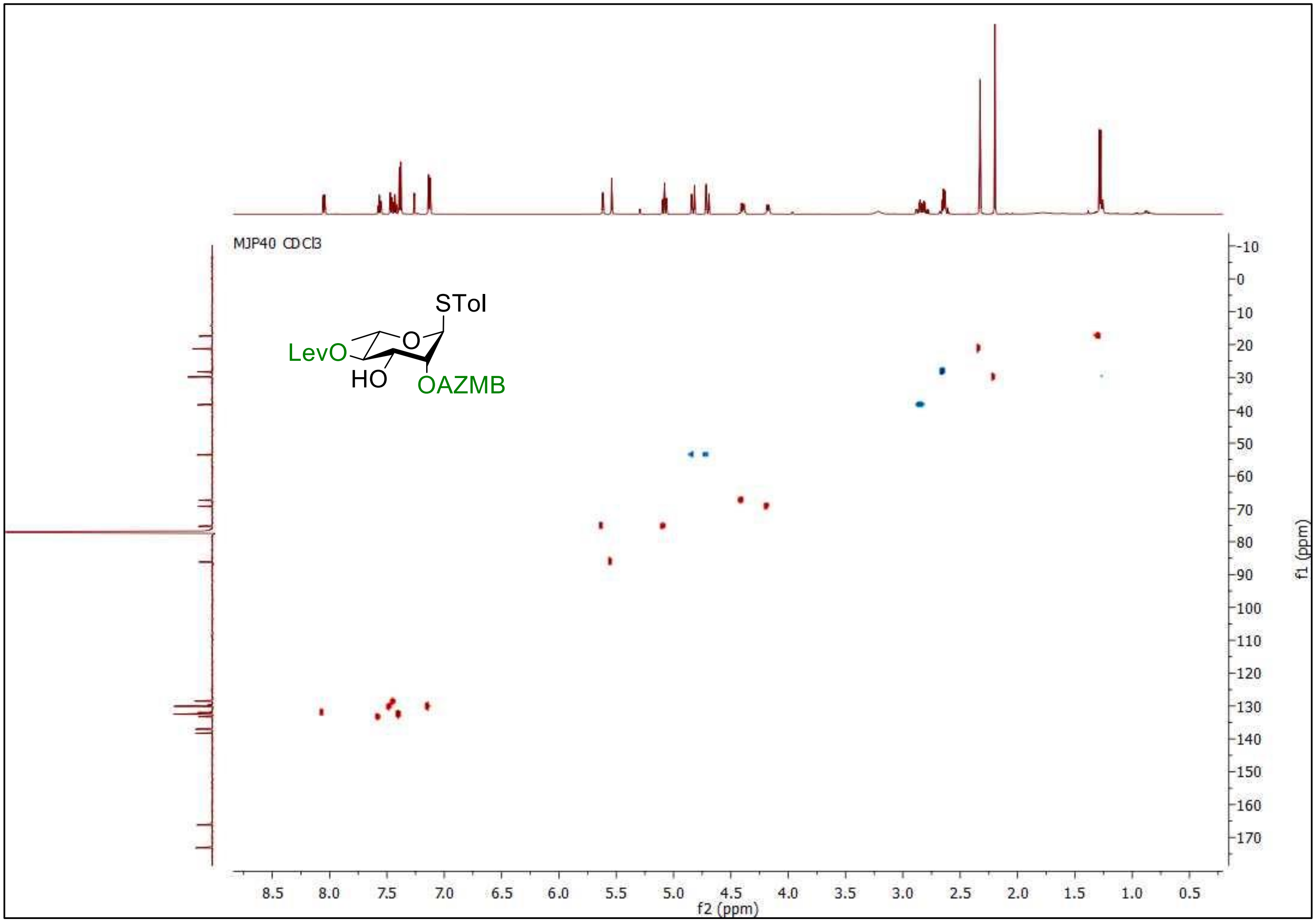

Figure S176 | ^{1}H NMR spectrum ($CDCl_3$, 600 MHz) of *para*-methylphenyl 2-*O*-*ortho*-(azidomethyl)benzoyl-3-*O*-(*R*)-3-(((*R*)-3-((*tert*-butyldimethylsilyl)oxy)decanoyl)oxy)decanoyl-4-*O*-levulinoyl-1-thio-α-L-rhamnopyranoside (**28**).

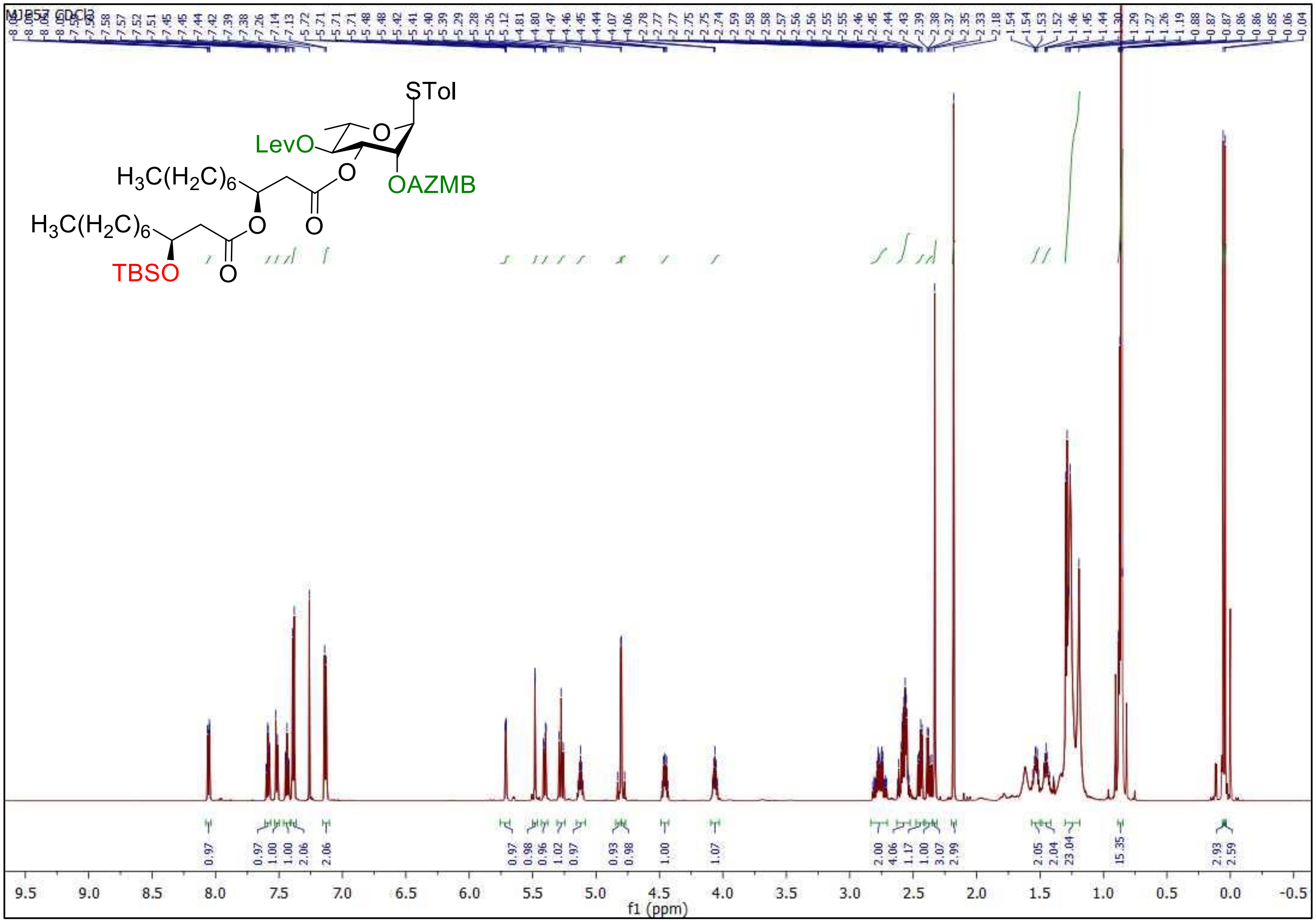

Figure S177 | COSY NMR spectrum ($CDCl_3$, 600 MHz) of *para*-methylphenyl 2-*O*-*ortho*-(azidomethyl)benzoyl-3-*O*-(*R*)-3-(((*R*)-3-((*tert*-butyldimethylsilyl)oxy)decanoyl)oxy)decanoyl-4-*O*-levulinoyl-1-thio-*α*-L-rhamnopyranoside (**28**).

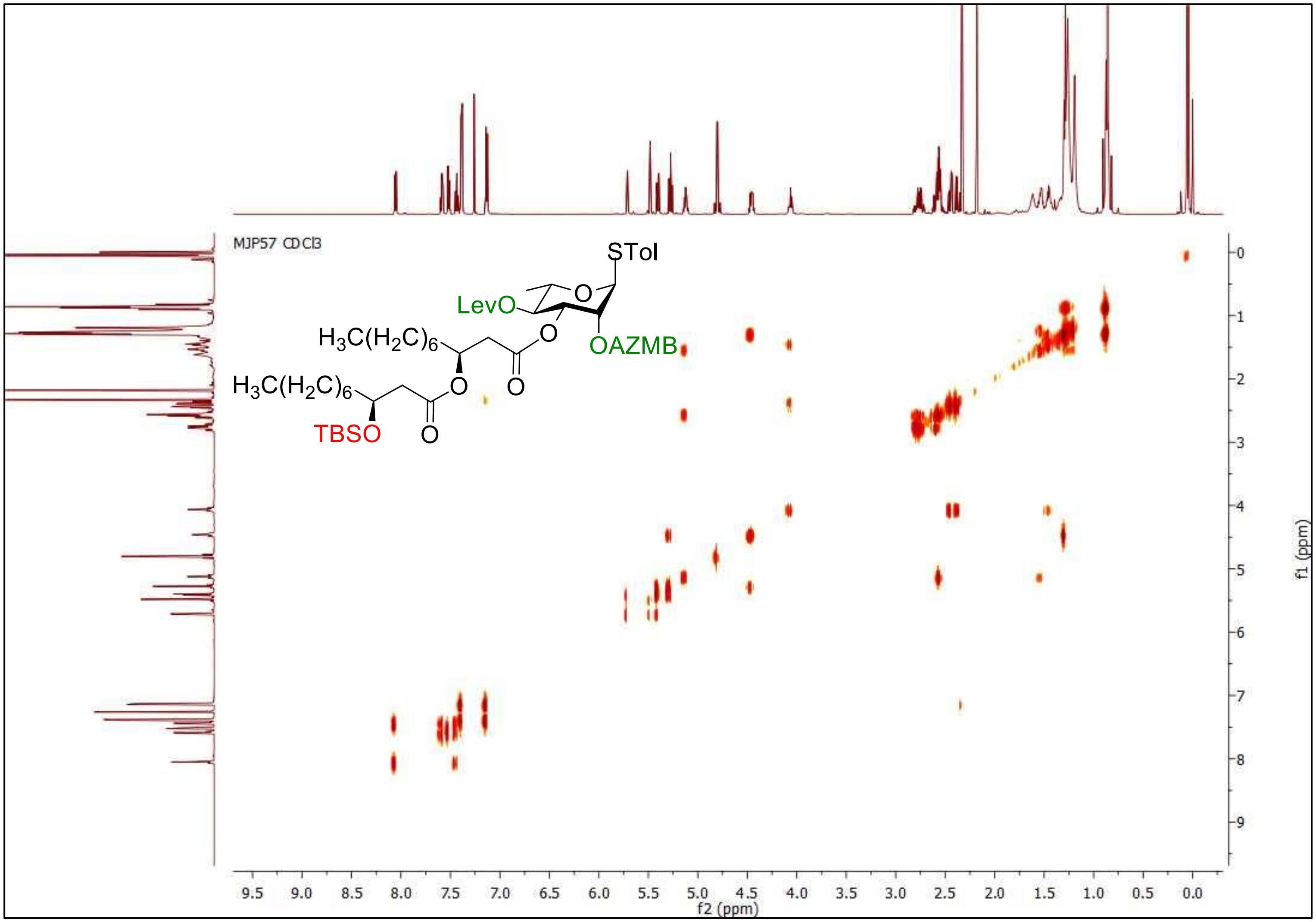

Figure S178 | ^{13}C NMR spectrum (CDCl$_3$, 600 MHz) of *para*-methylphenyl 2-*O*-*ortho*-(azidomethyl)benzoyl-3-*O*-(*R*)-3-(((*R*)-3-((*tert*-butyldimethylsilyl)oxy)decanoyl)oxy)decanoyl-4-*O*-levulinoyl-1-thio-*α*-L-rhamnopyranoside (**28**).

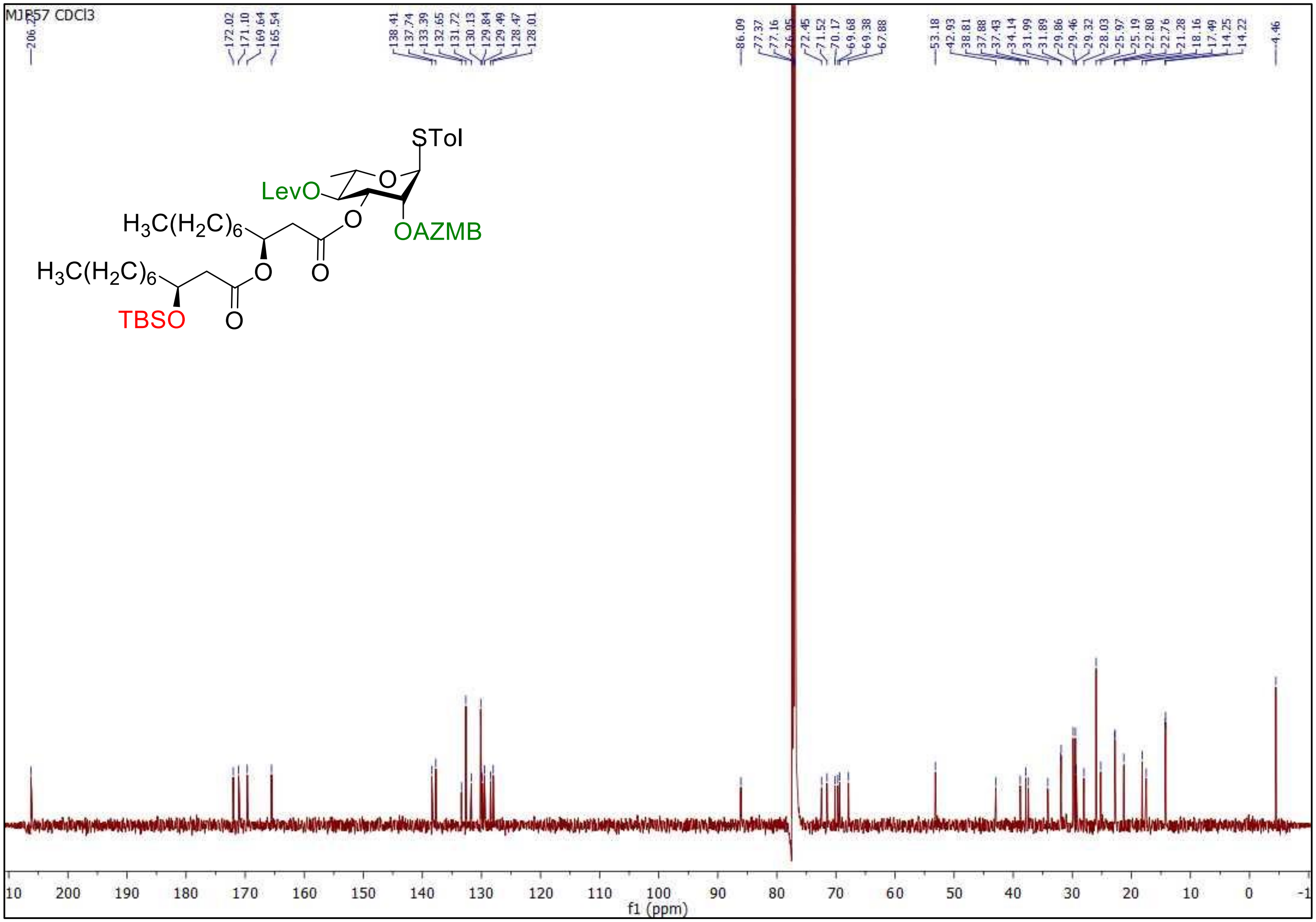

Figure S179 | HSQC NMR spectrum ($CDCl_3$, 600 MHz) of *para*-methylphenyl 2-*O*-*ortho*-(azidomethyl)benzoyl-3-*O*-(*R*)-3-(((*R*)-3-((*tert*-butyldimethylsilyl)oxy)decanoyl)oxy)decanoyl-4-*O*-levulinoyl-1-thio-α-L-rhamnopyranoside (**28**).

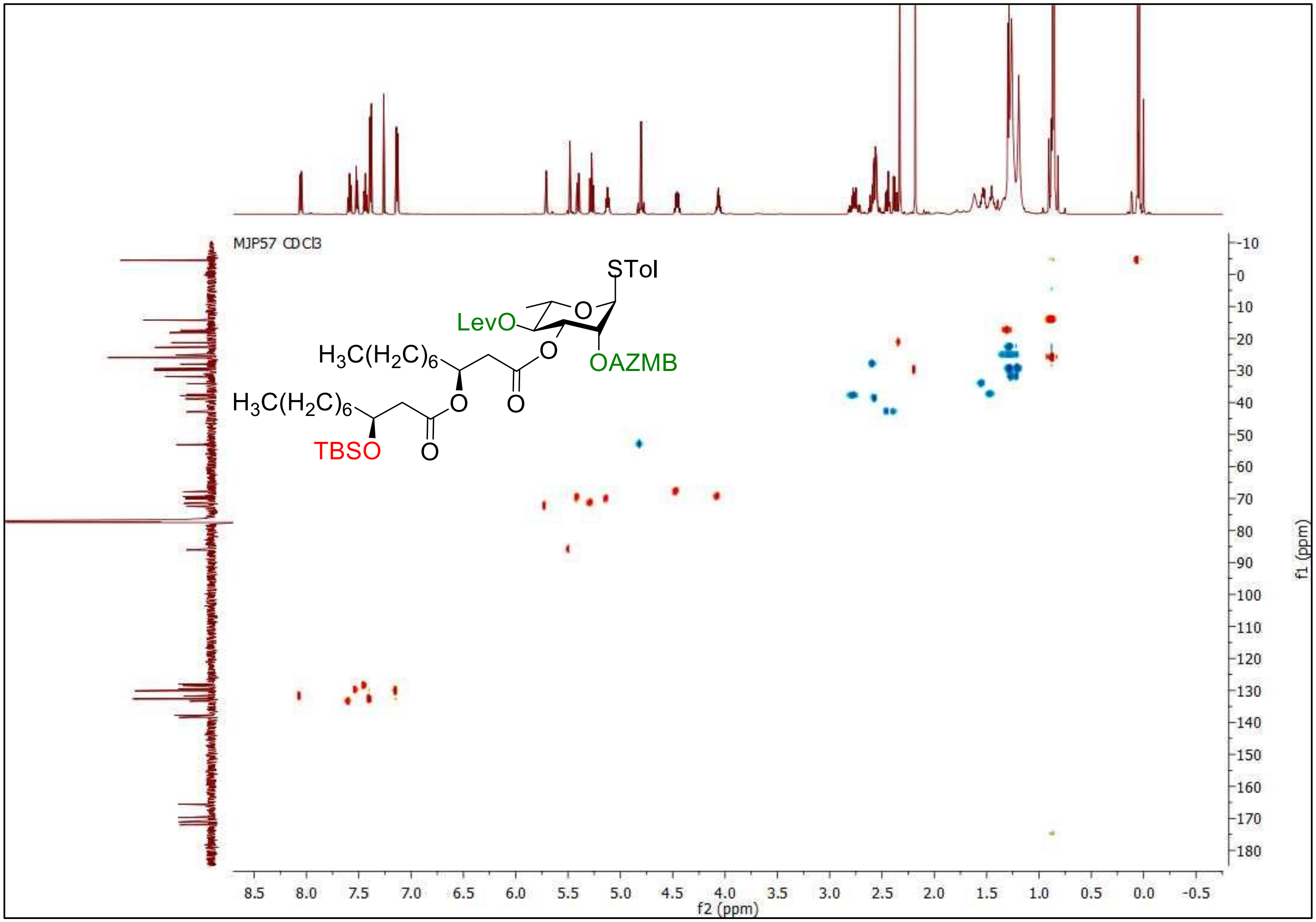

Figure S180 | ^{1}H NMR spectrum ($CDCl_3$, 600 MHz) of *para*-methylphenyl 2-*O*-*ortho*-(azidomethyl)benzoyl-3-*O*-(*R*)-3-(((*R*)-3-(hydroxydecanoyl)oxy)decanoyl-4-*O*-levulinoyl-1-thio-*α*-L-rhamnopyranoside (**38**).

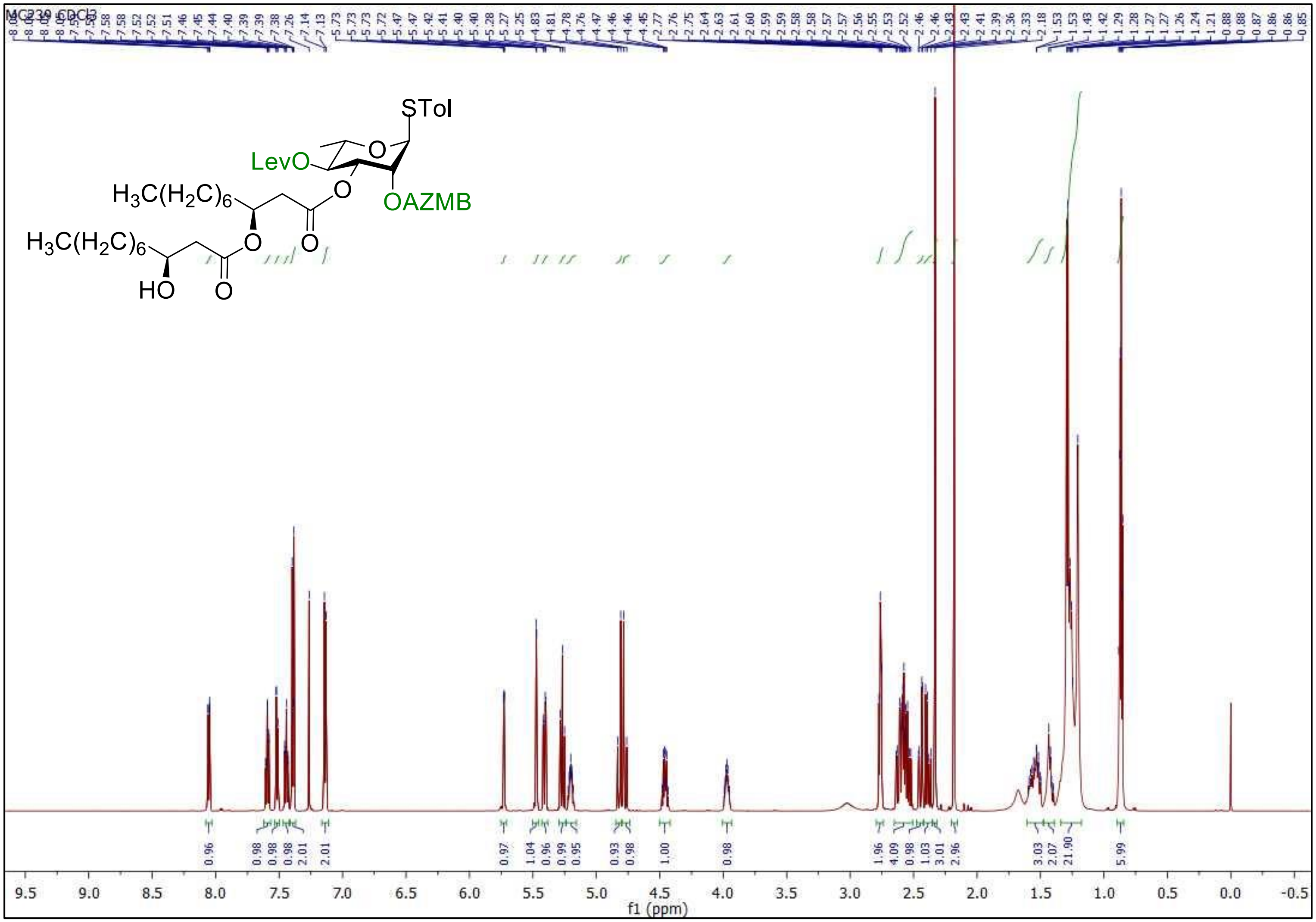

Figure S181 | COSY NMR spectrum ($CDCl_3$, 600 MHz) of *para*-methylphenyl 2-*O*-*ortho*-(azidomethyl)benzoyl-3-*O*-(*R*)-3-(((*R*)-3-(hydroxydecanoyl)oxy)decanoyl-4-*O*-levulinoyl-1-thio-*α*-L-rhamnopyranoside (**38**).

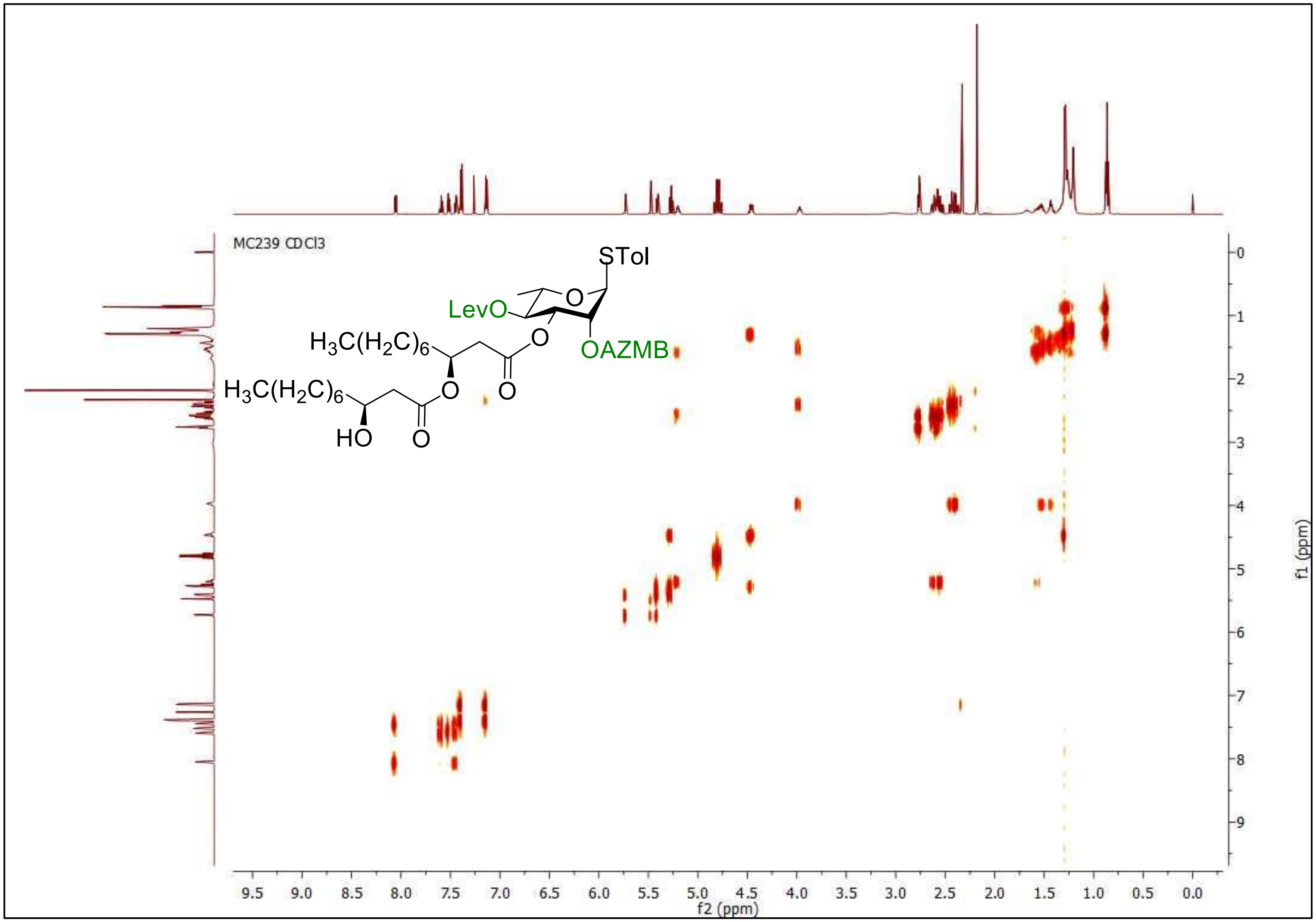

Figure S182 | ^{13}C NMR spectrum ($CDCl_3$, 600 MHz) of *para*-methylphenyl 2-*O*-*ortho*-(azidomethyl)benzoyl-3-*O*-(*R*)-3-(((*R*)-3-(hydroxydecanoyl)oxy)decanoyl-4-*O*-levulinoyl-1-thio-*α*-L-rhamnopyranoside (**38**).

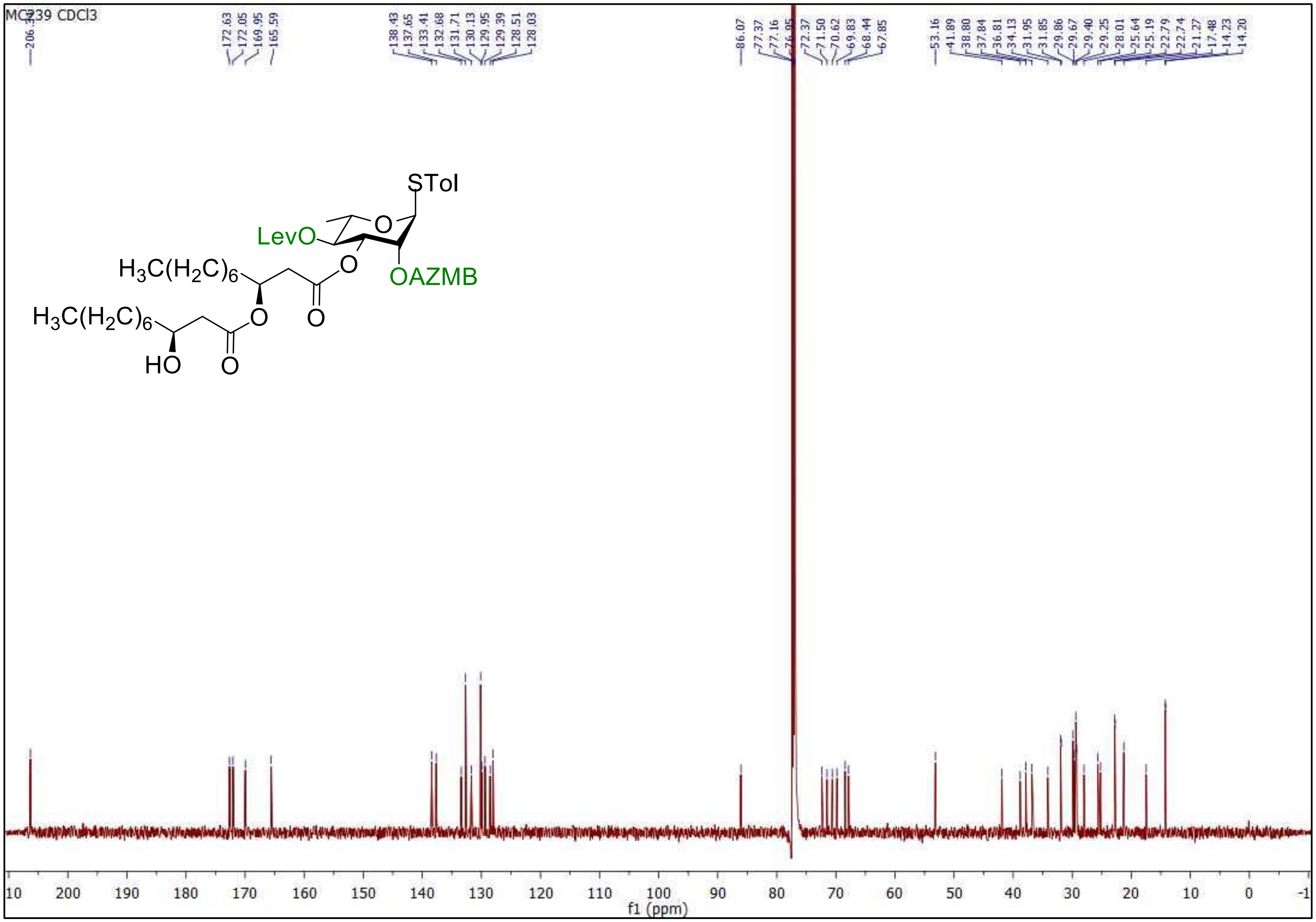

Figure S183 | HSQC NMR spectrum ($CDCl_3$, 600 MHz) of *para*-methylphenyl 2-*O*-*ortho*-(azidomethyl)benzoyl-3-*O*-(*R*)-3-(((*R*)-3-(hydroxydecanoyl)oxy)decanoyl-4-*O*-levulinoyl-1-thio-*α*-L-rhamnopyranoside (**38**).

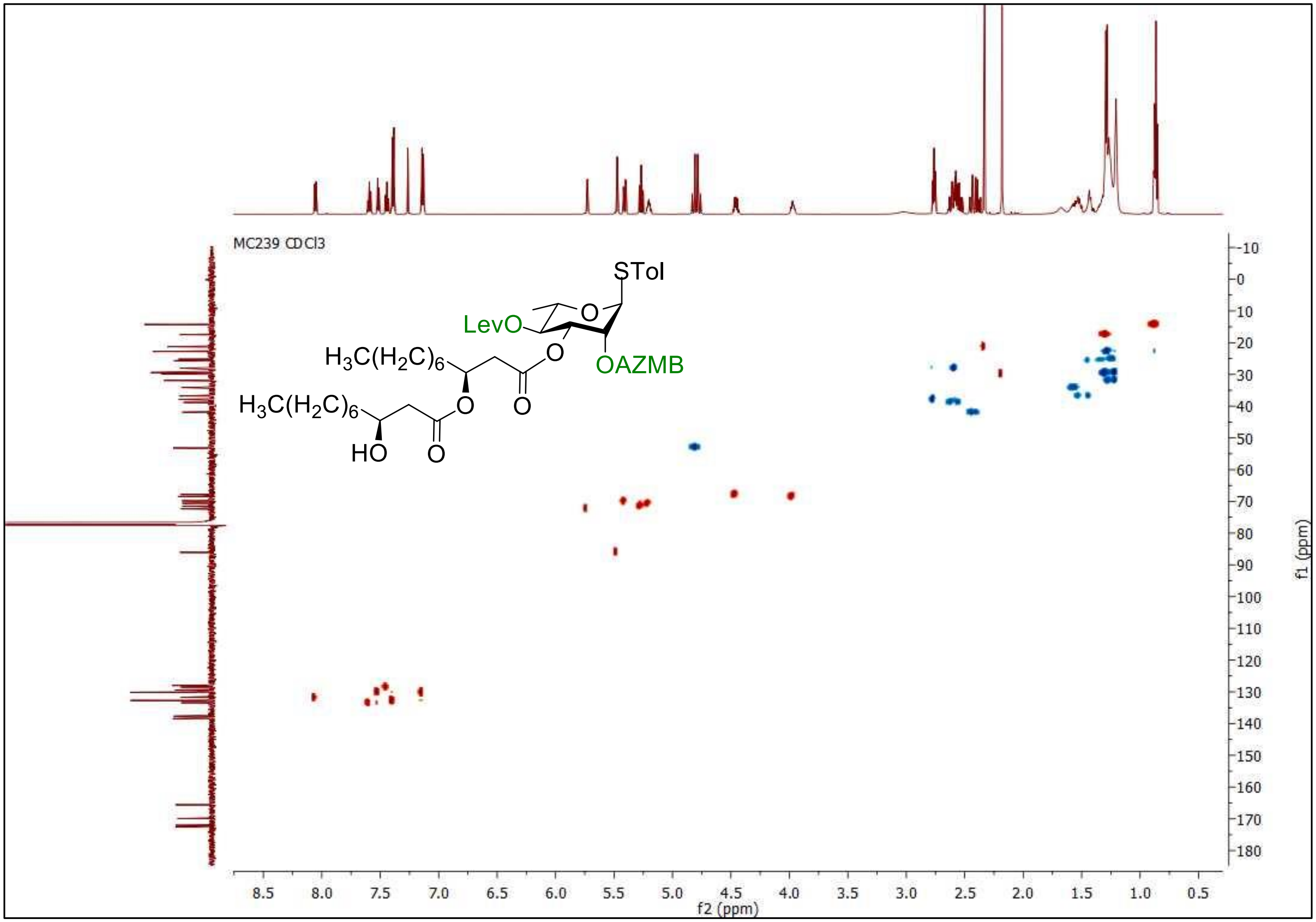

Figure S184 | ^{1}H NMR spectrum ($CDCl_3$, 600 MHz) of macrolide **39*β***.

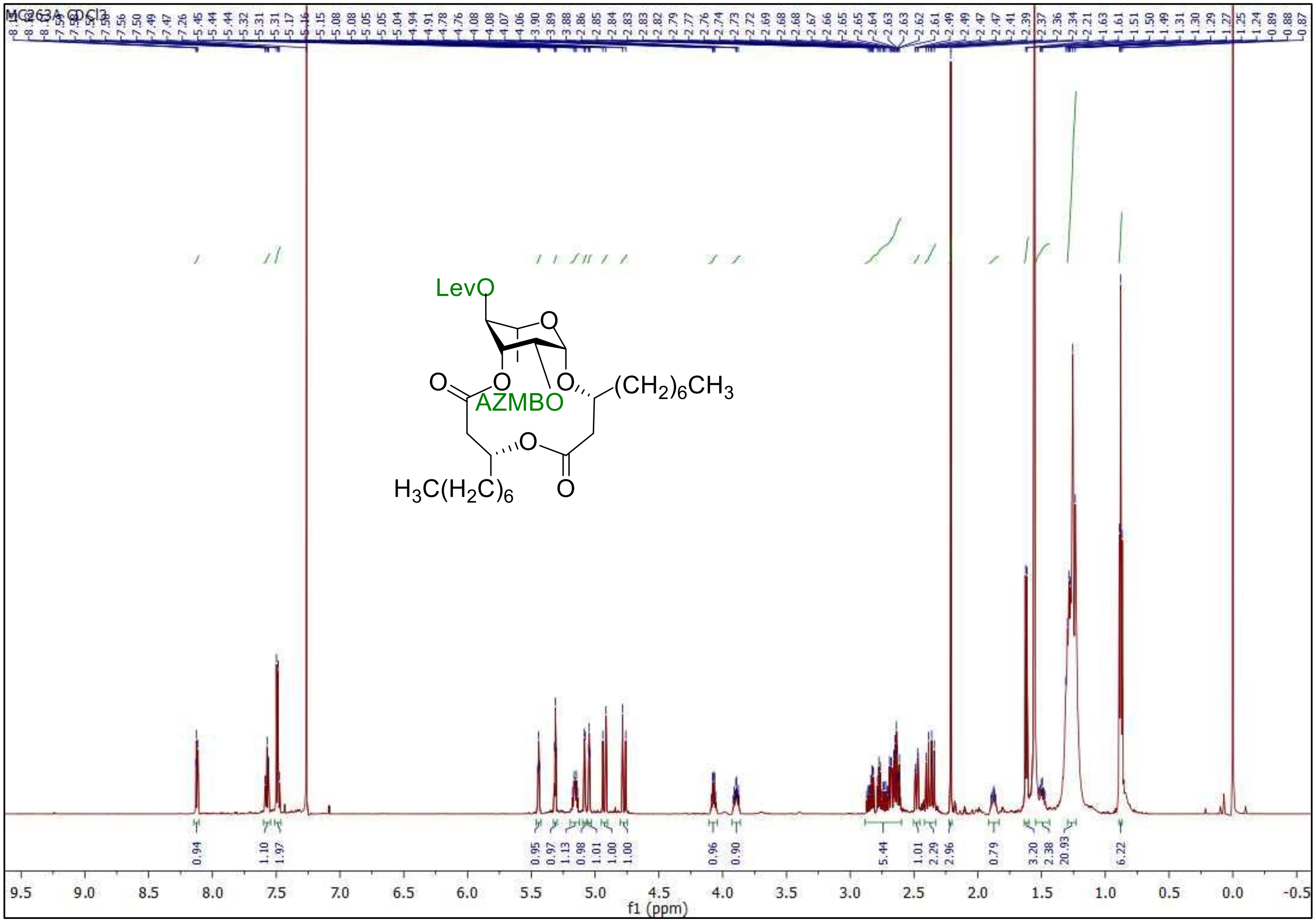

Figure S185| COSY NMR spectrum ($CDCl_3$, 600 MHz) of macrolide **39*β***.

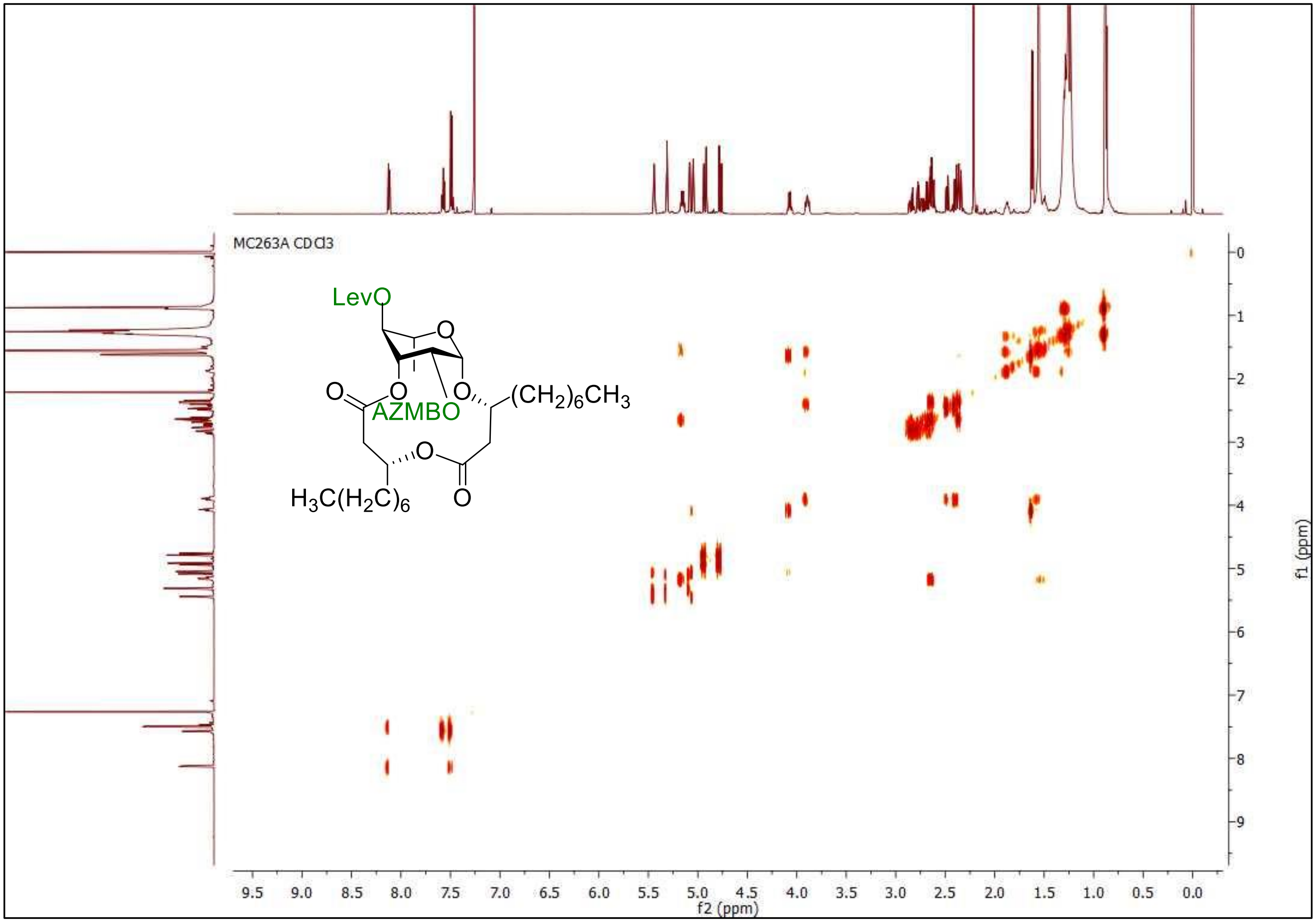

Figure S186 | ^{13}C NMR spectrum ($CDCl_3$, 600 MHz) of macrolide **39*β***.

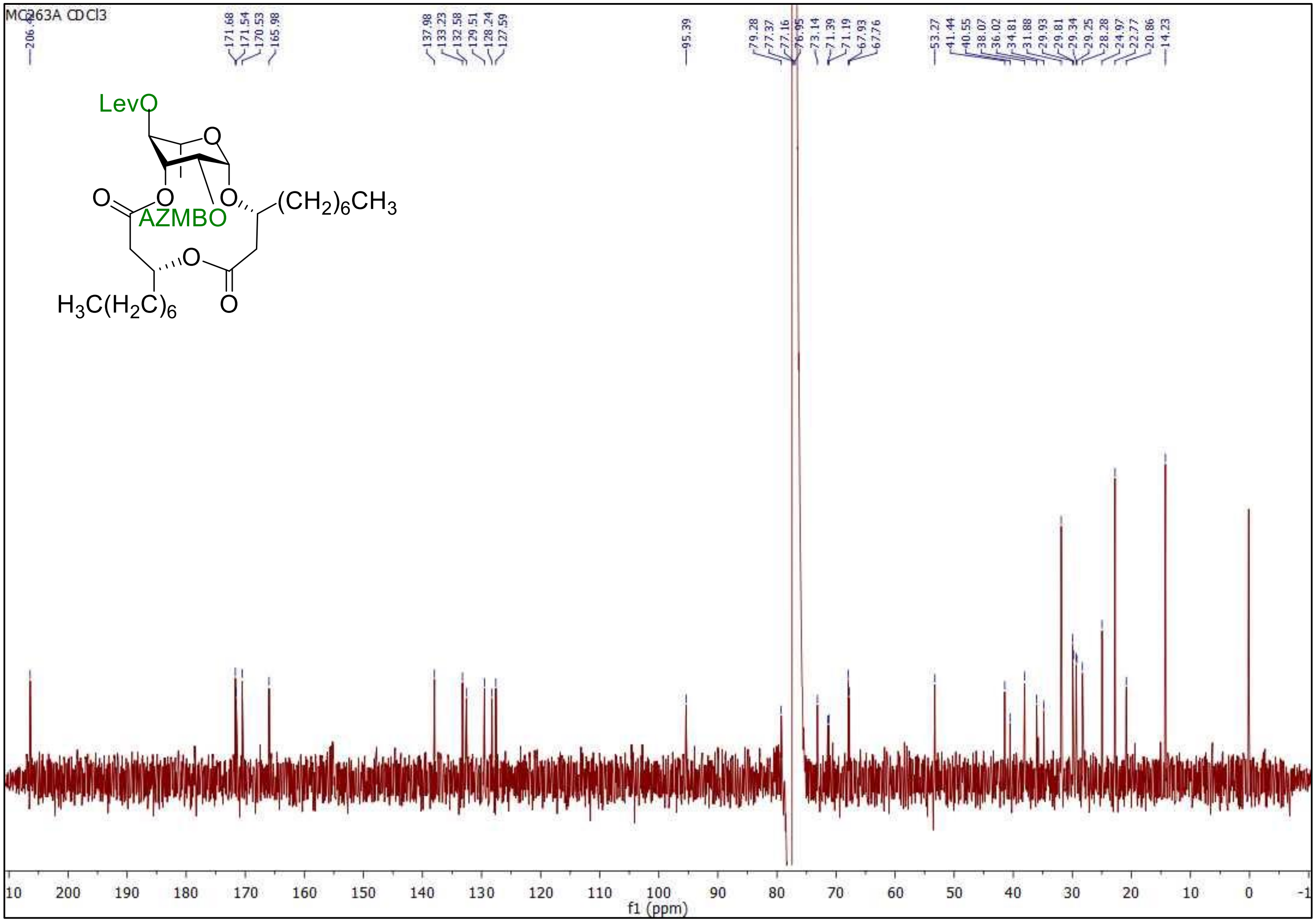

Figure S187 | HSQC NMR spectrum ($CDCl_3$, 600 MHz) of macrolide **39*β***.

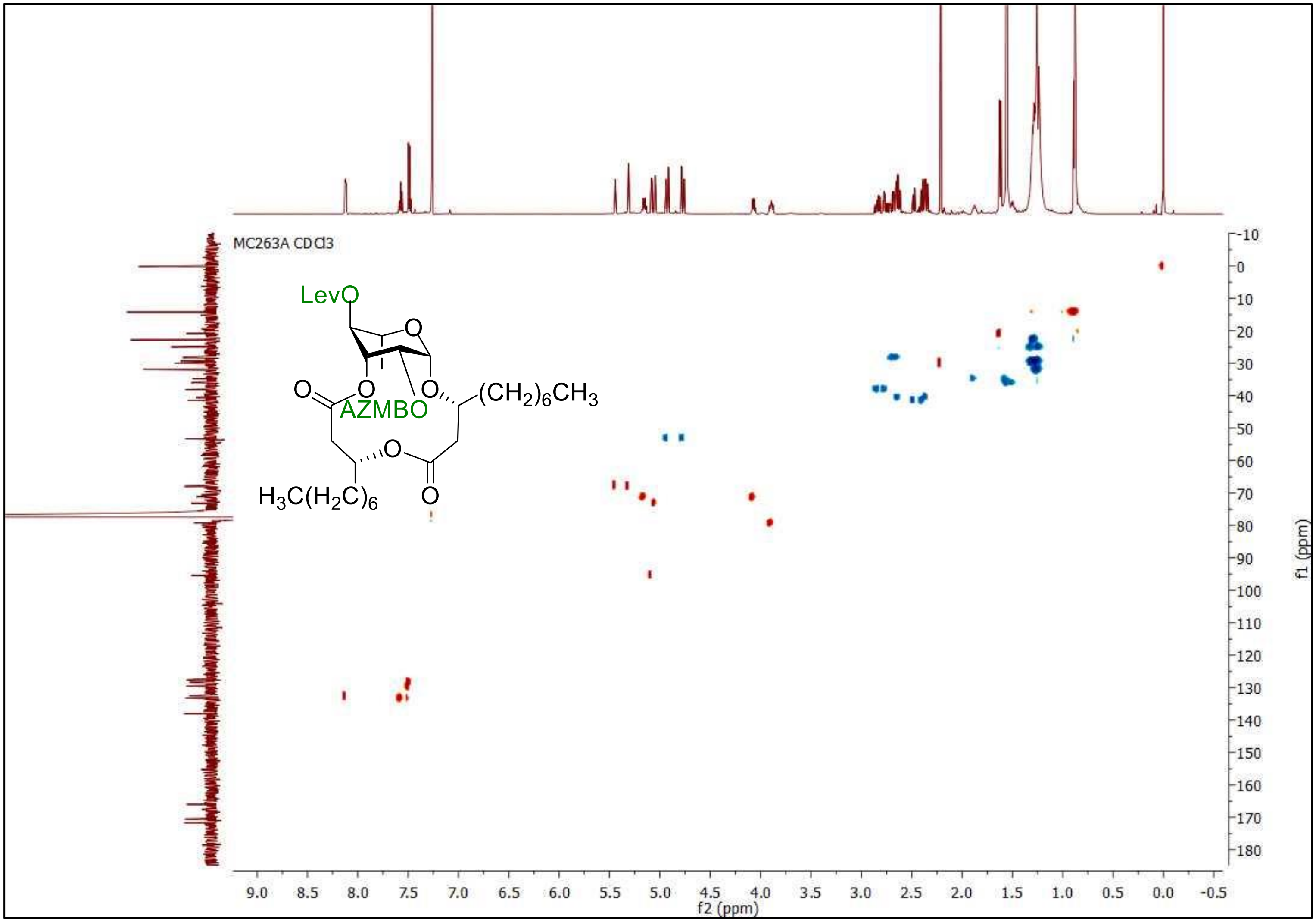

Figure S188 | undecoupled HSQC NMR spectrum ($CDCl_3$, 600 MHz) of macrolide **39*β***.

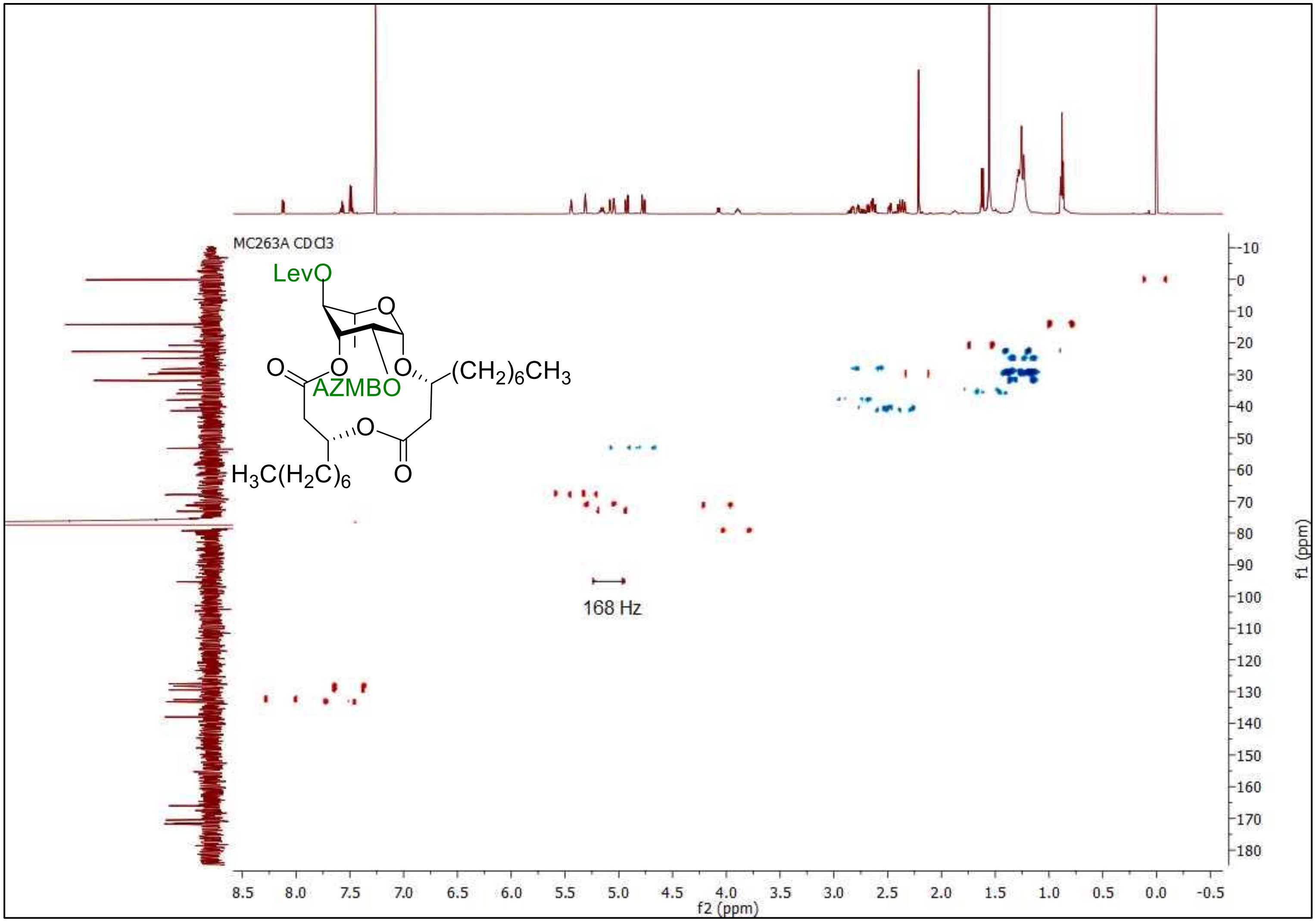

Figure S189 | ^{1}H NMR spectrum ($CDCl_3$, 600 MHz) of macrolide **39*α***.

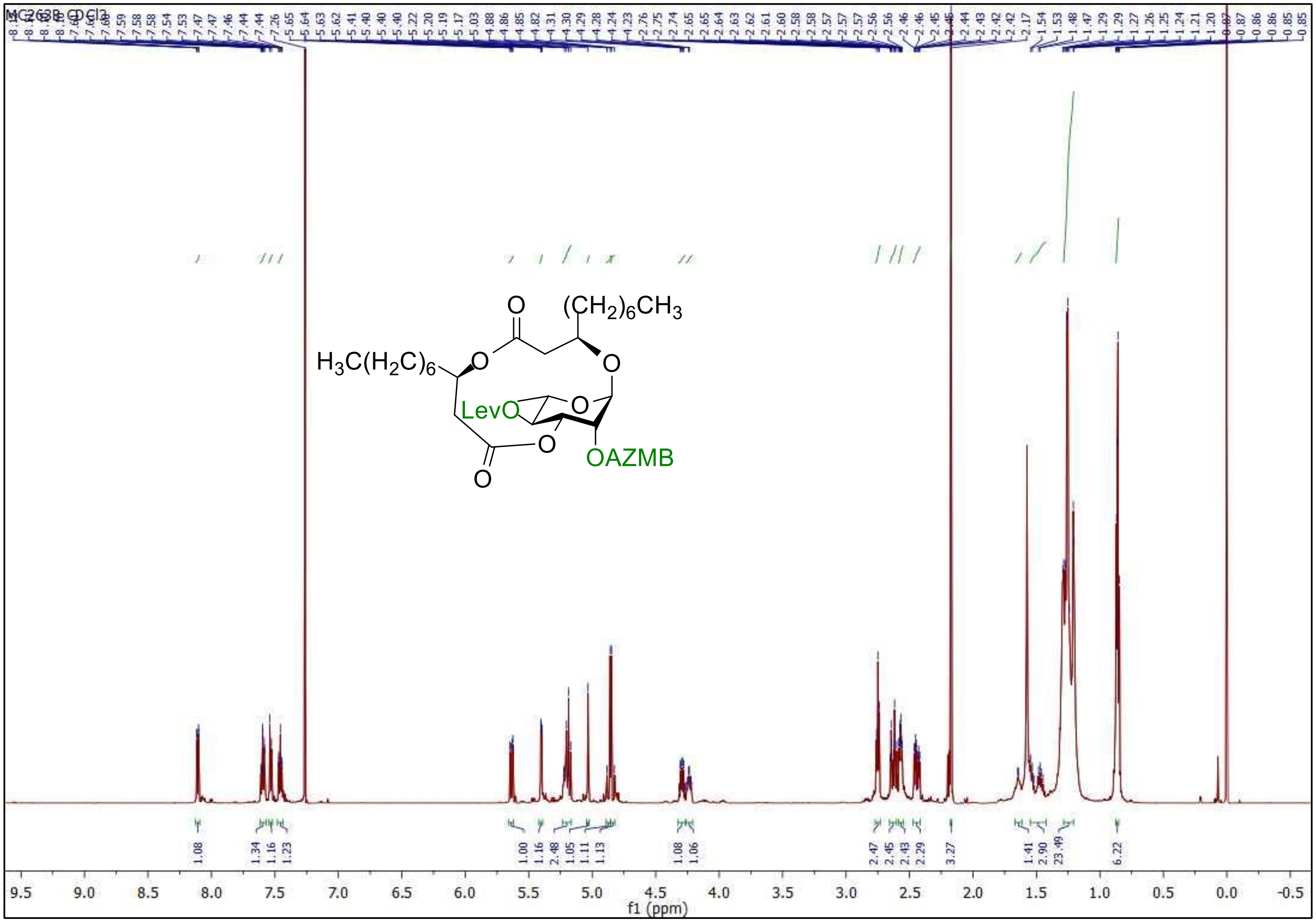

Figure S190 | COSY NMR spectrum ($CDCl_3$, 600 MHz) of macrolide **39*α***.

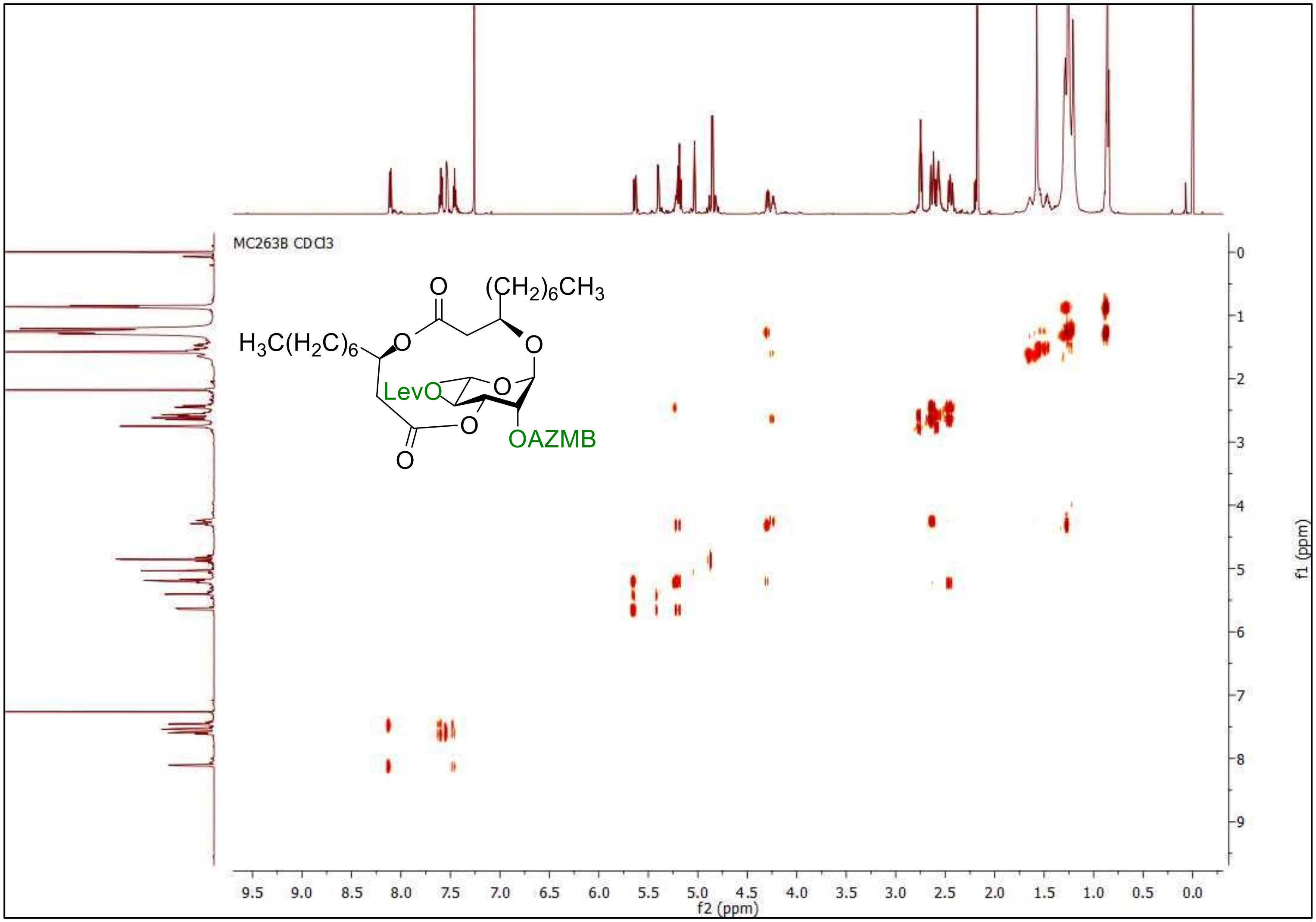

Figure S191 | ^{13}C NMR spectrum ($CDCl_3$, 600 MHz) of macrolide **39*α***.

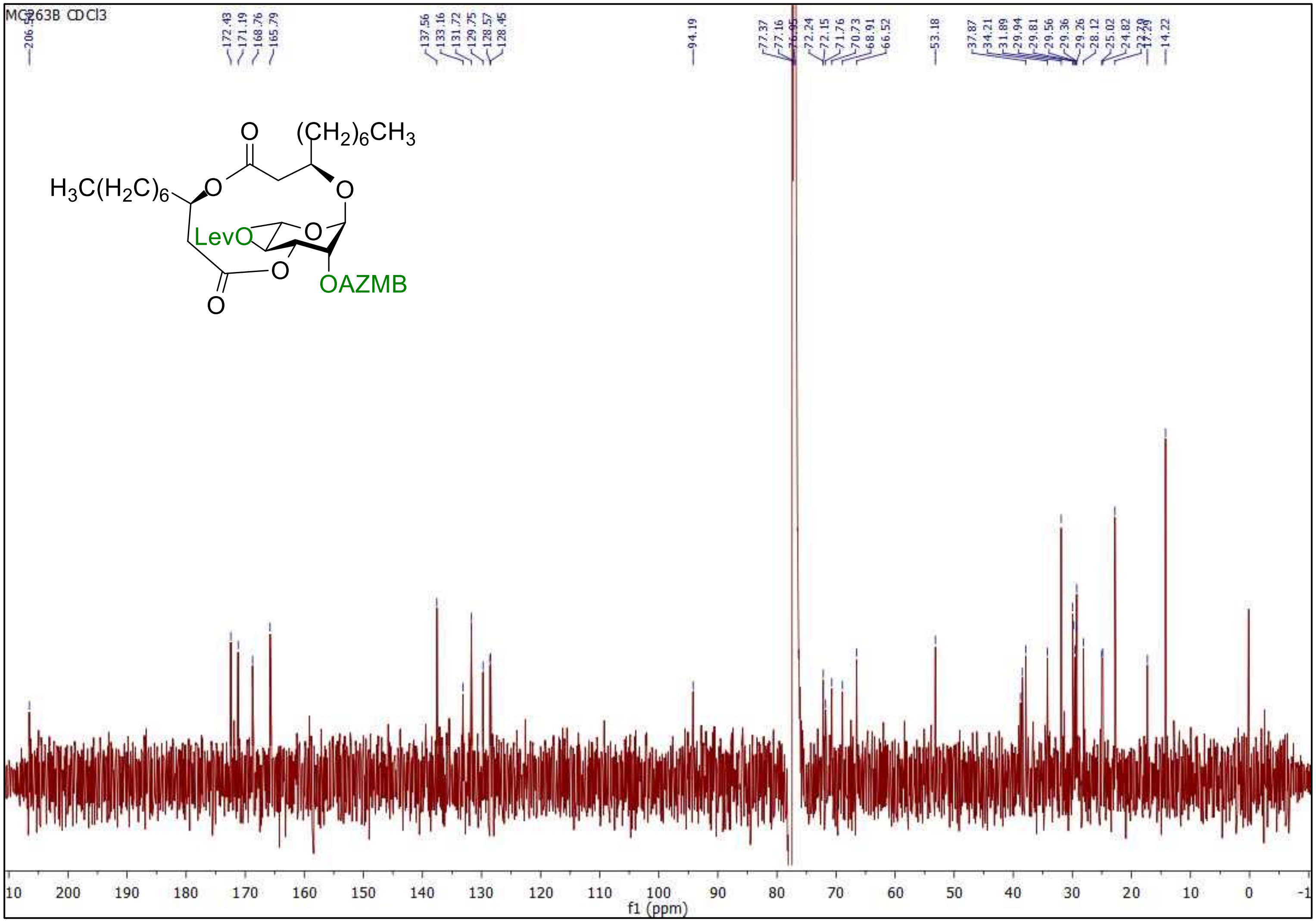

Figure S192 | HSQC NMR spectrum ($CDCl_3$, 600 MHz) of macrolide **39*α***.

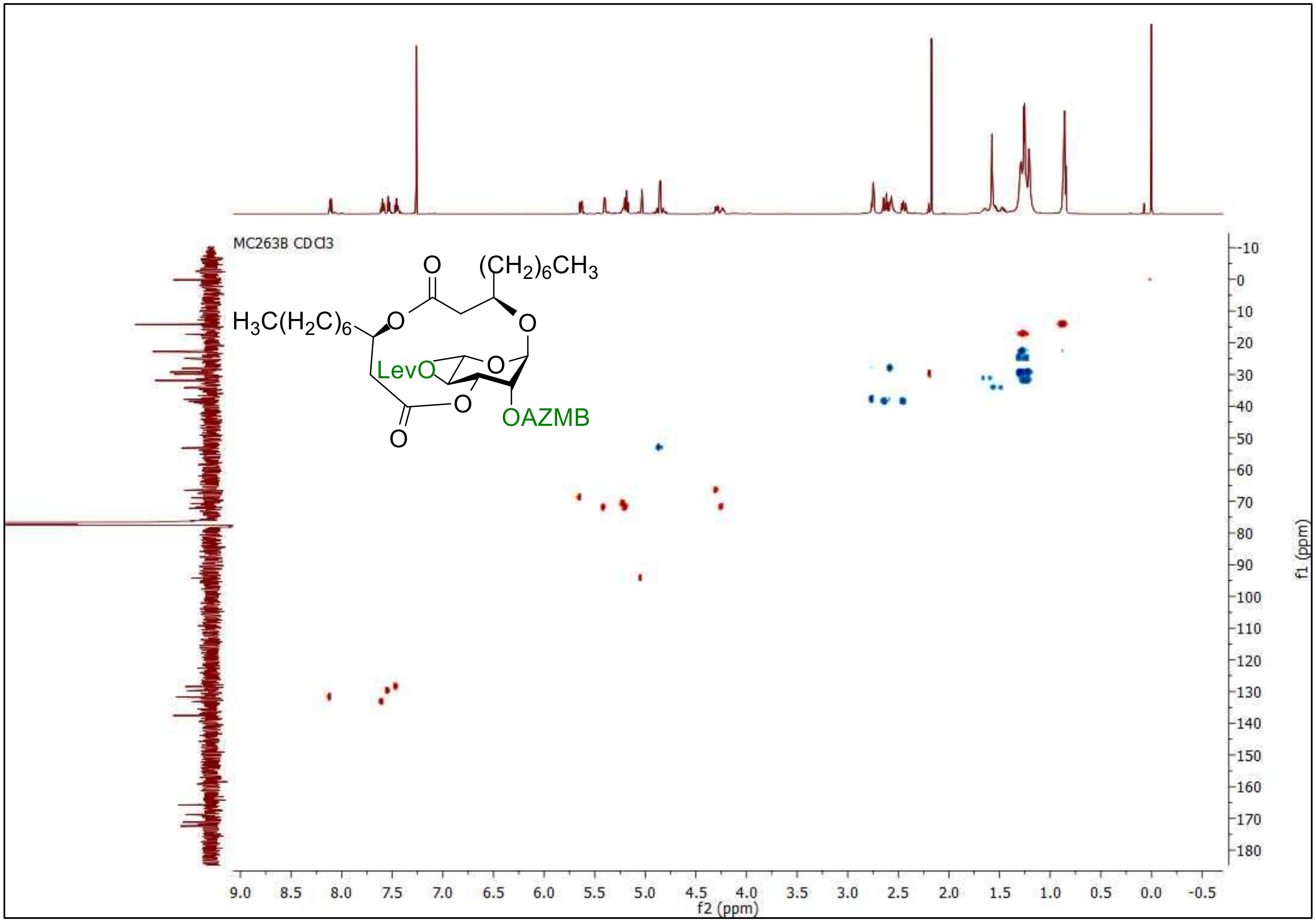

Figure S193 | undecoupled HSQC NMR spectrum ($CDCl_3$, 600 MHz) of macrolide **39*α***.

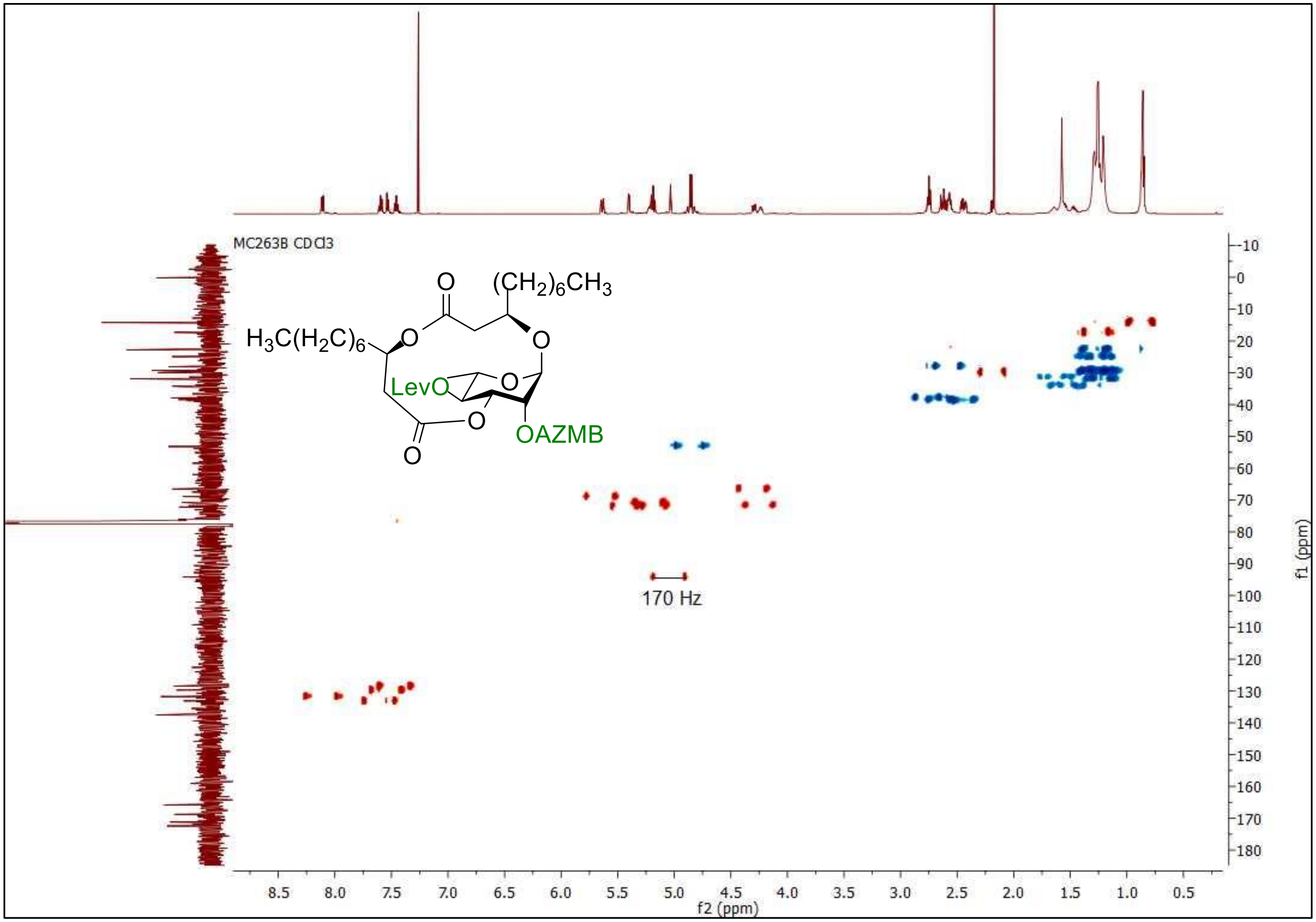

Figure S194 | ^{1}H NMR spectrum ($CDCl_3$, 600 MHz) of macrolide **S18*β***.

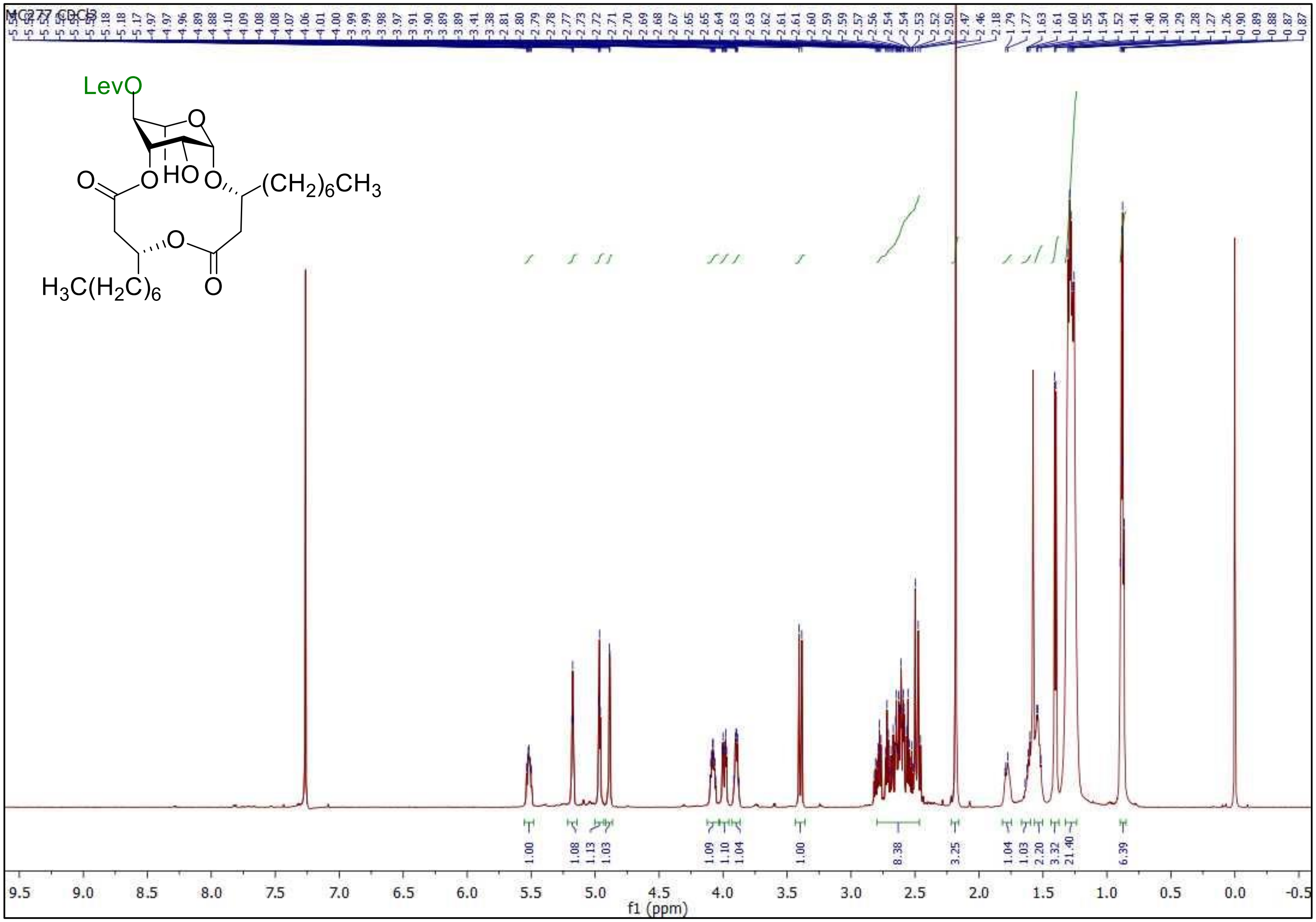

Figure S195 | COSY NMR spectrum ($CDCl_3$, 600 MHz) of macrolide **S18*β***.

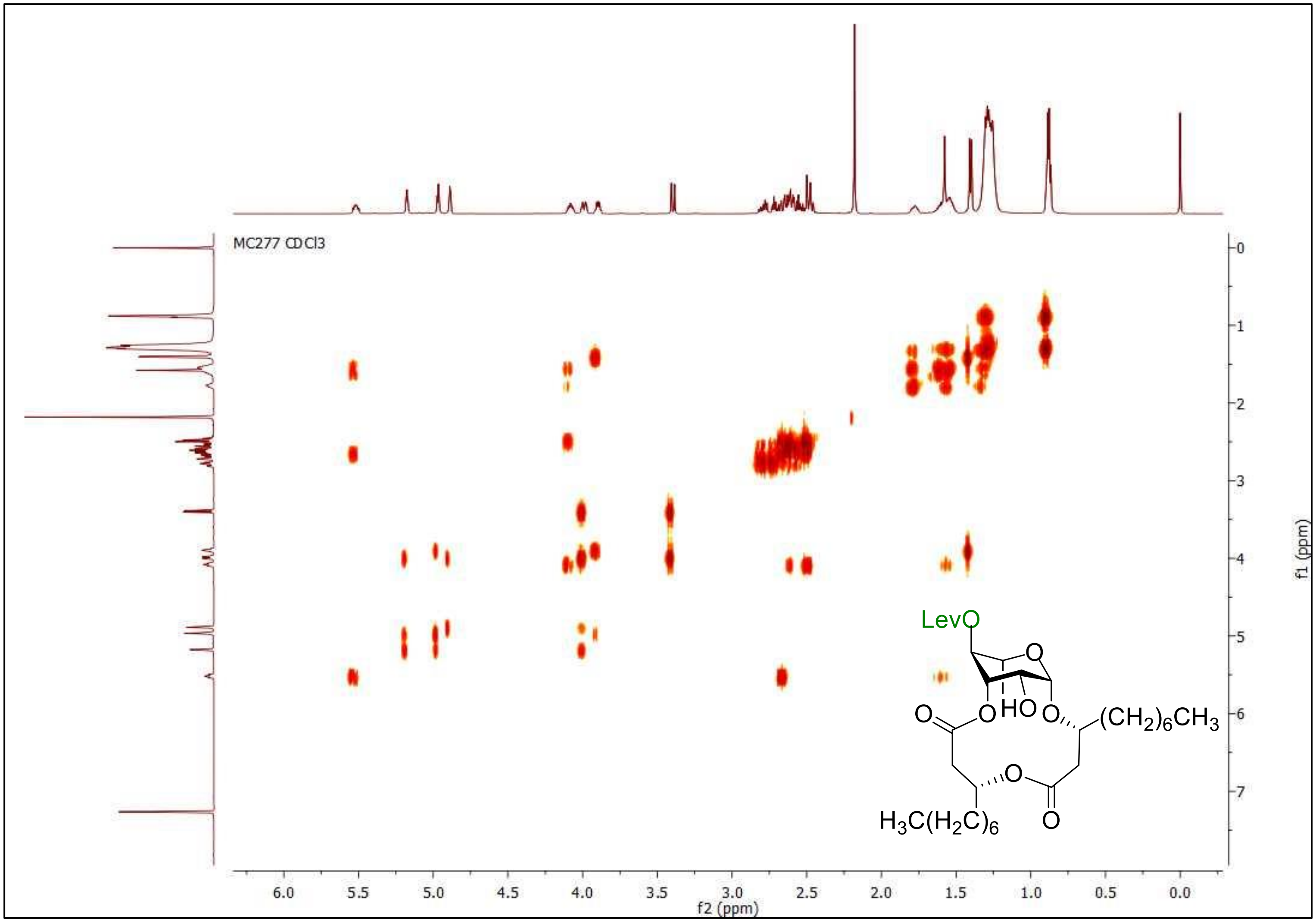

Figure S196 | ^{13}C NMR spectrum ($CDCl_3$, 600 MHz) of macrolide **S18*β***.

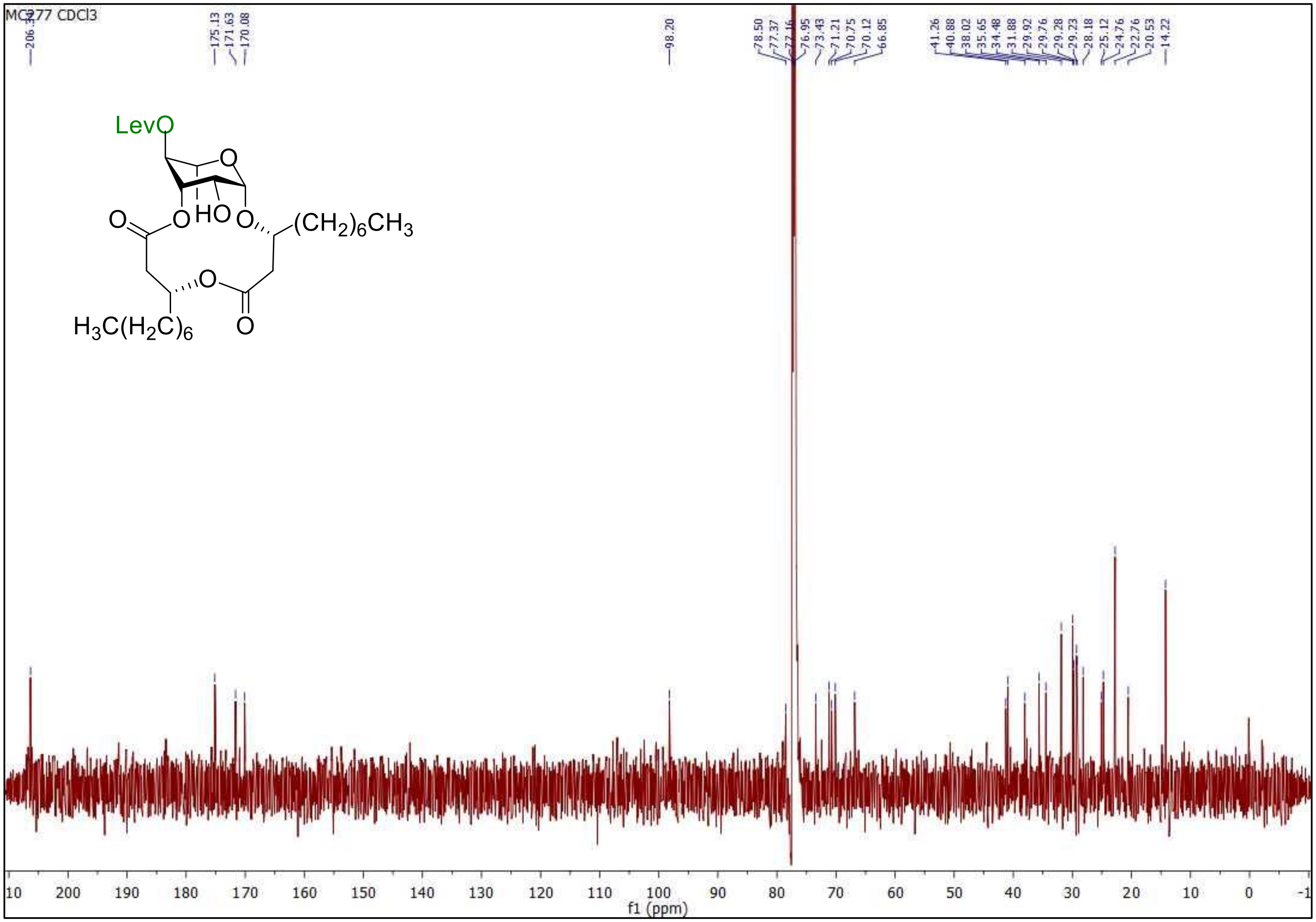

Figure S197 | HSQC NMR spectrum ($CDCl_3$, 600 MHz) of macrolide **S18*β***.

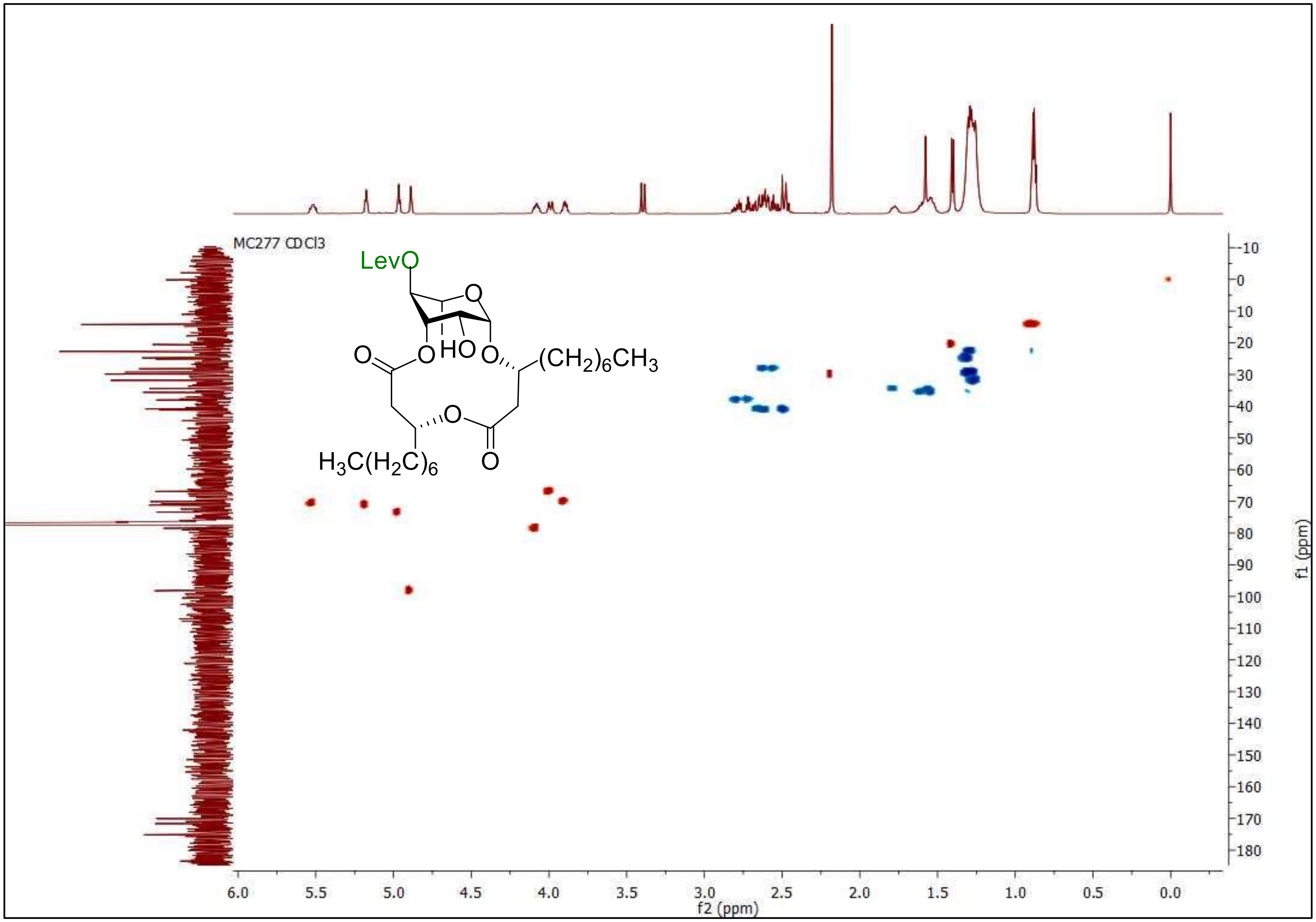

Figure S198 | ^{1}H NMR spectrum ($CDCl_3$, 600 MHz) of (1→3)-macrolactonized rhamnolipid **6*β***.

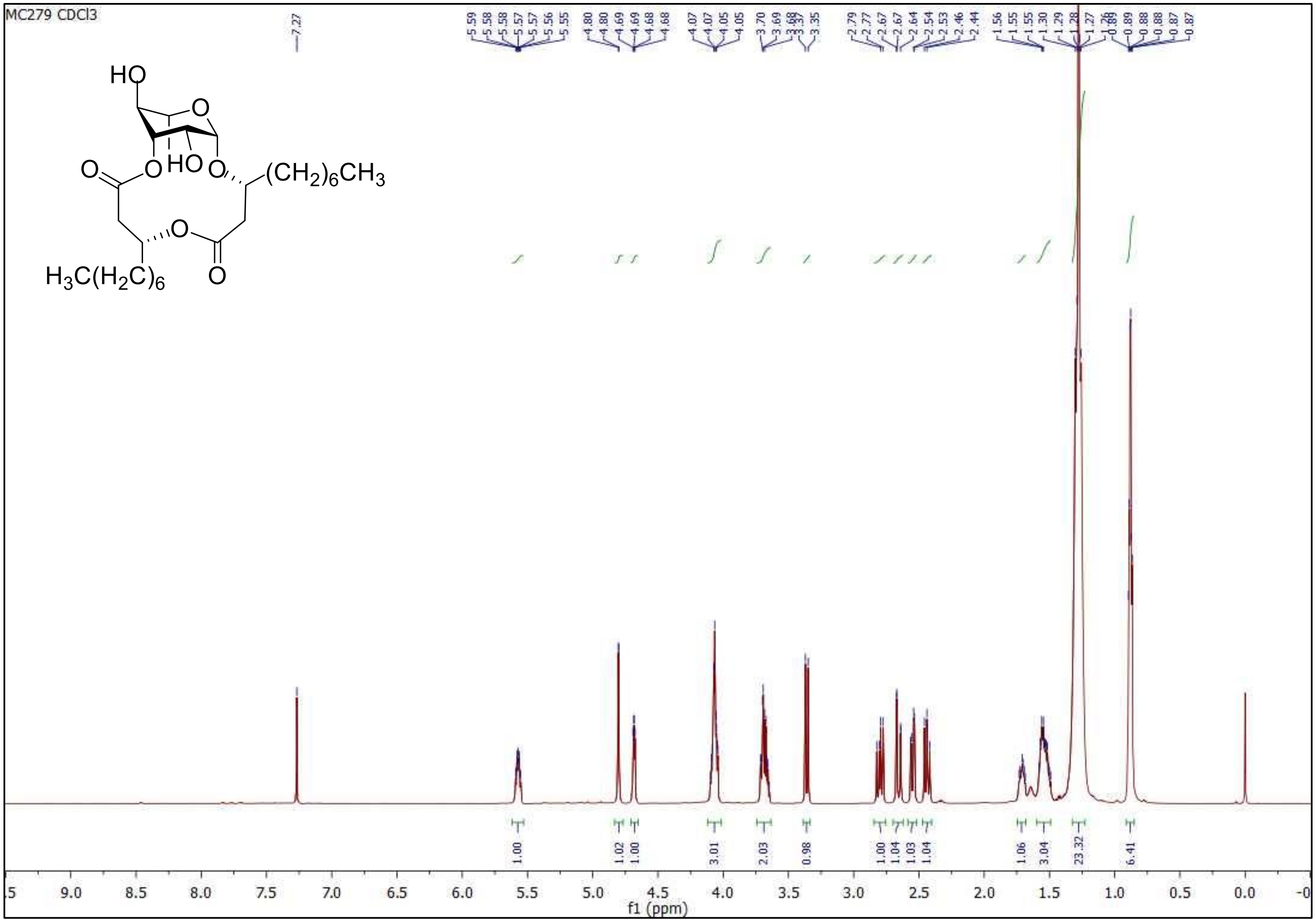

Figure S199 | COSY NMR spectrum ($CDCl_3$, 600 MHz) of (1→3)-macrolactonized rhamnolipid ***6β***.

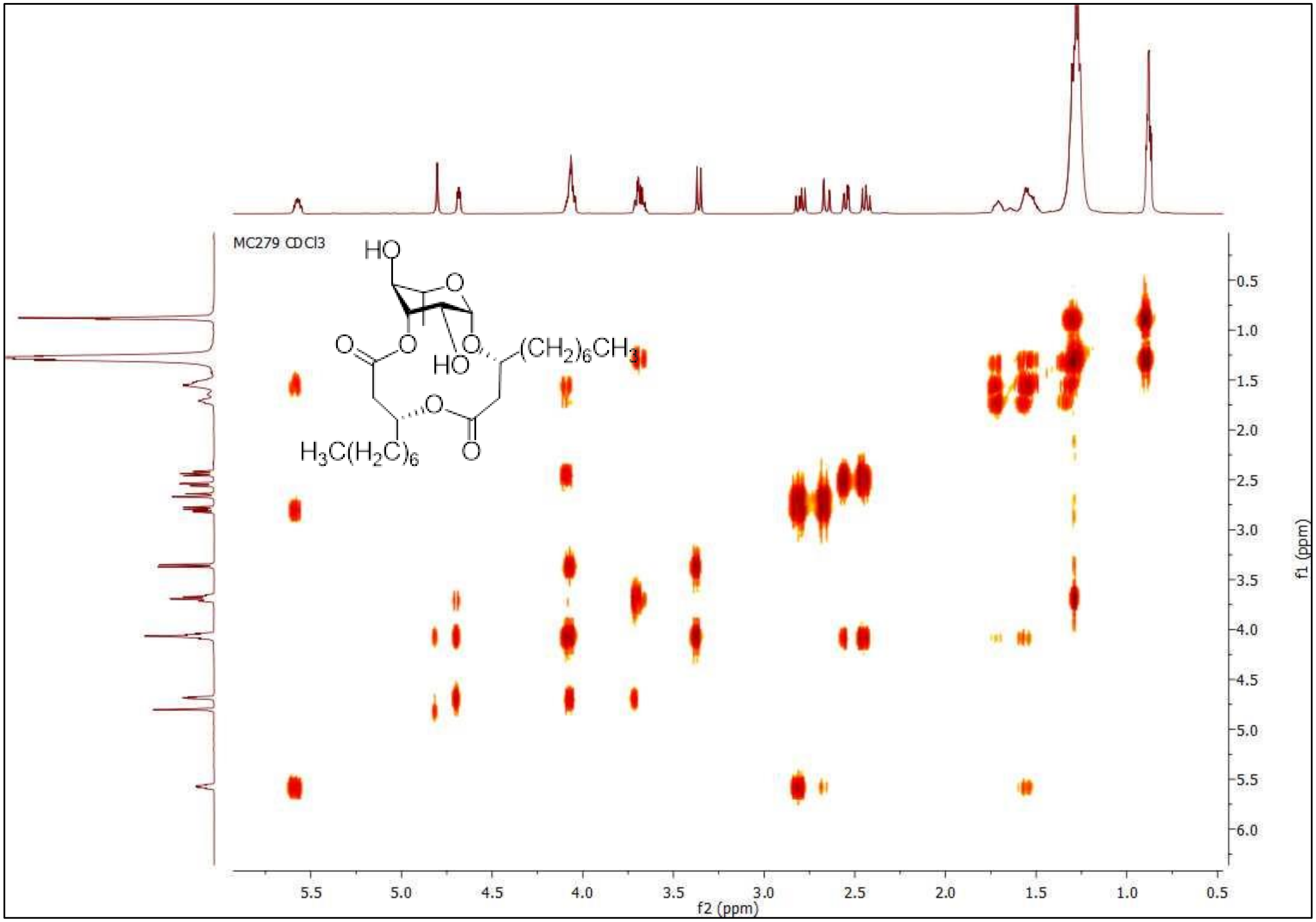

Figure S200 | ^{13}C NMR spectrum ($CDCl_3$, 600 MHz) of (1→3)-macrolactonized rhamnolipid **6*β***.

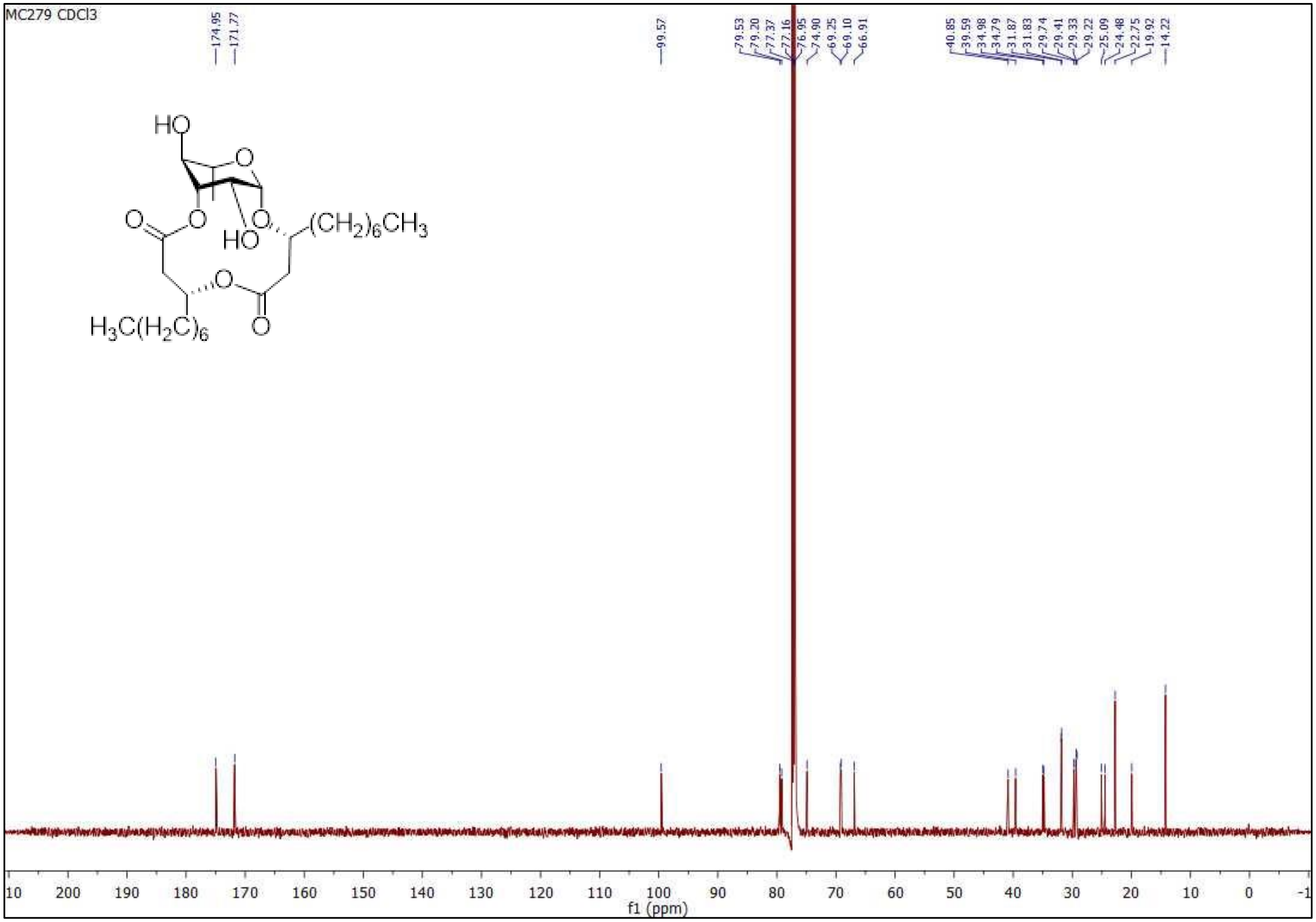

Figure S201 | HSQC NMR spectrum ($CDCl_3$, 600 MHz) of (1→3)-macrolactonized rhamnolipid **6*β***.

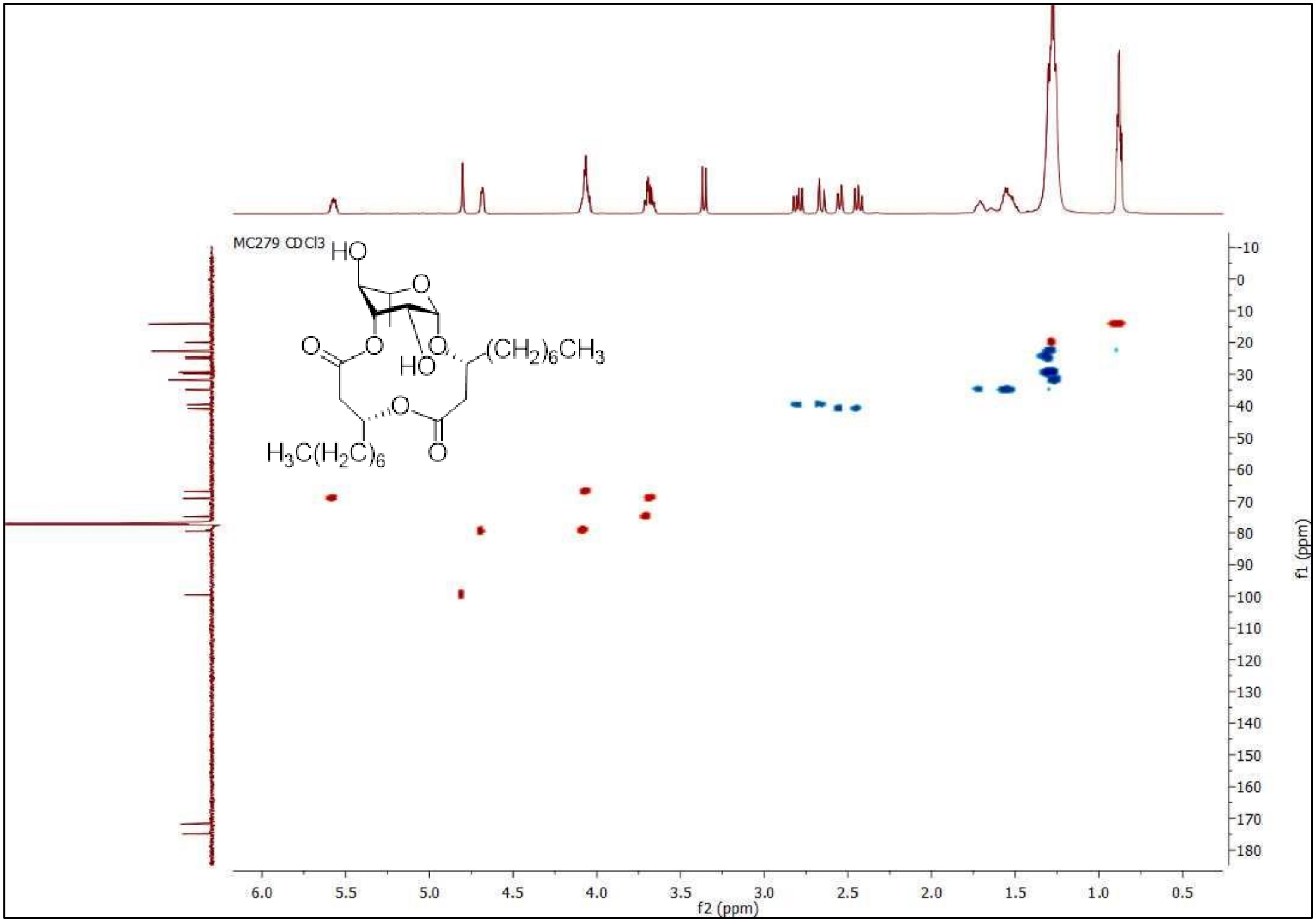

Figure S202 | ^{1}H NMR spectrum ($CDCl_3$, 600 MHz) of macrolide **S18*α***.

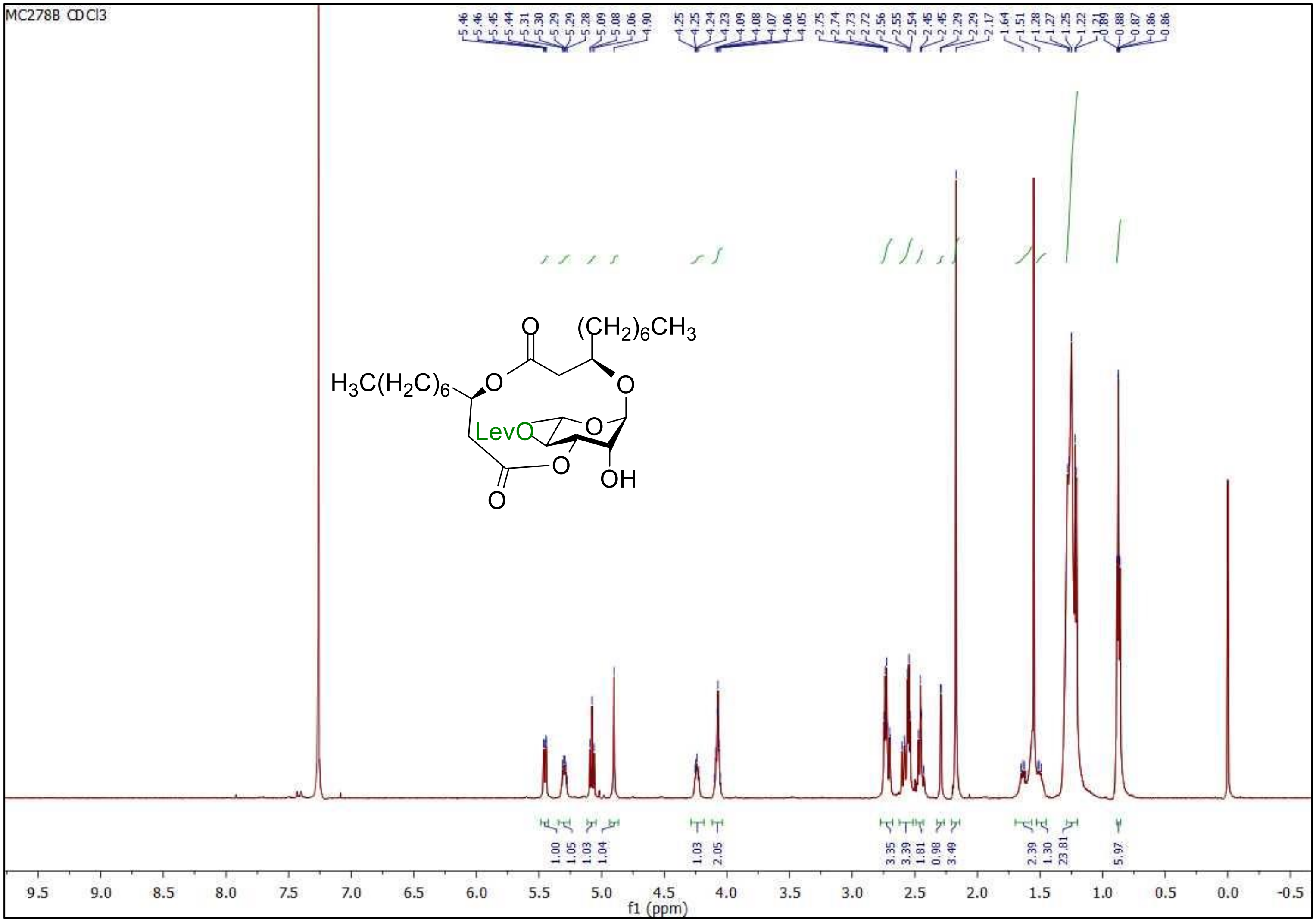

Figure S203 | COSY NMR spectrum ($CDCl_3$, 600 MHz) of macrolide **S18*α***.

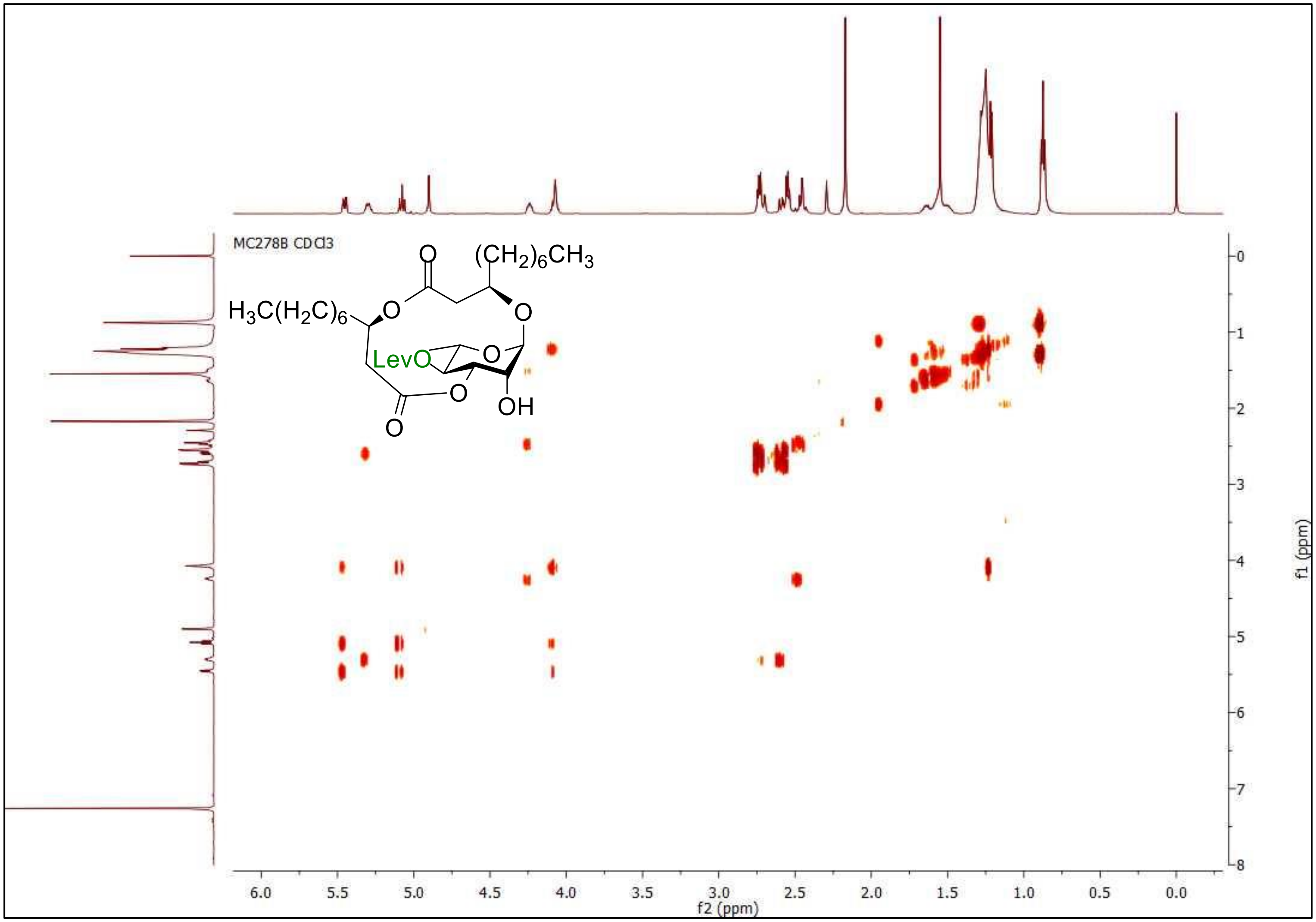

Figure S204 | ^{13}C NMR spectrum ($CDCl_3$, 600 MHz) of macrolide **S18*α***.

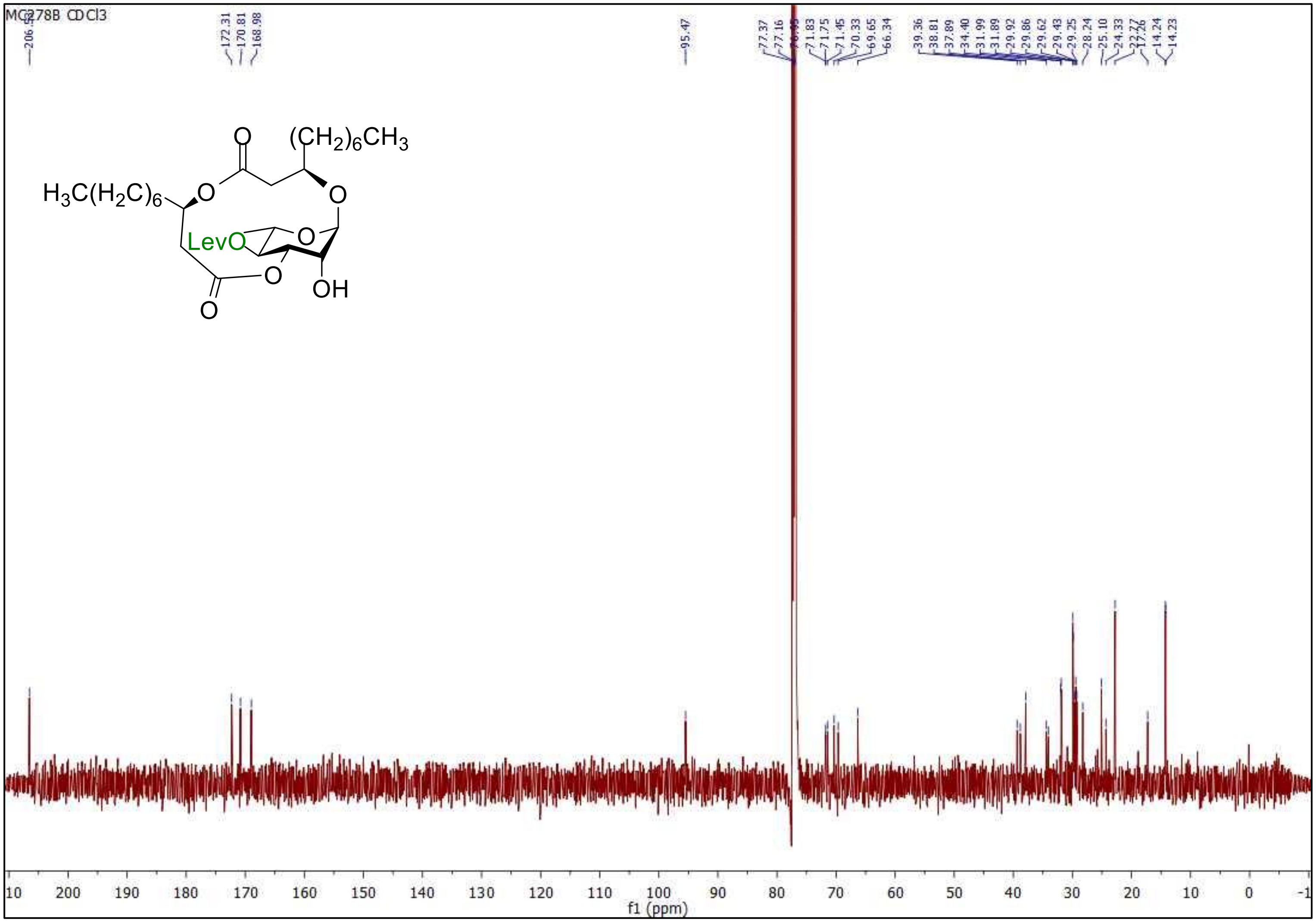

Figure S205 | HSQC NMR spectrum ($CDCl_3$, 600 MHz) of macrolide **S18*α***.

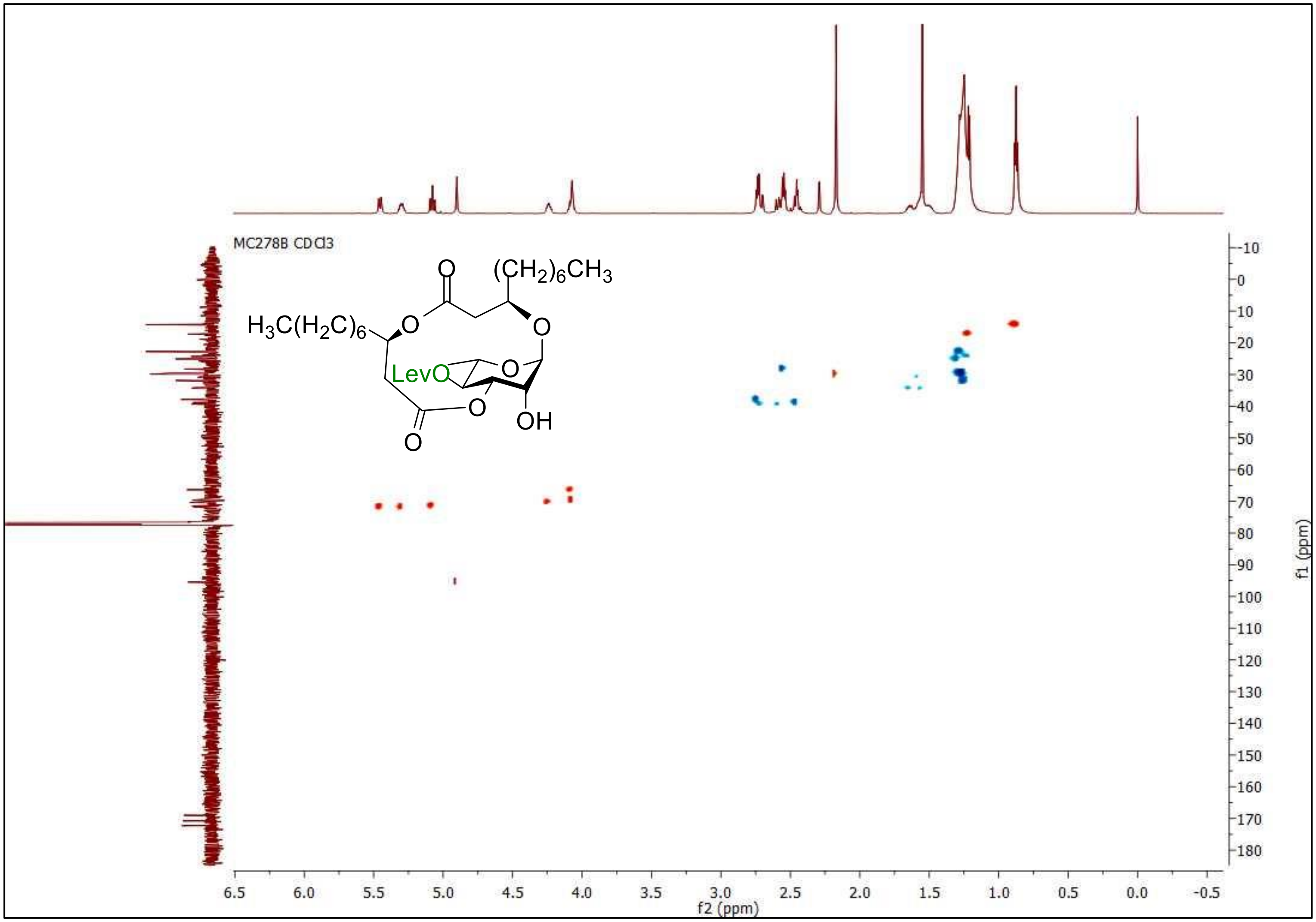

Figure S206 | ^{1}H NMR spectrum ($CDCl_3$, 600 MHz) of (1→3)-macrolactonized rhamnolipid **6*α***.

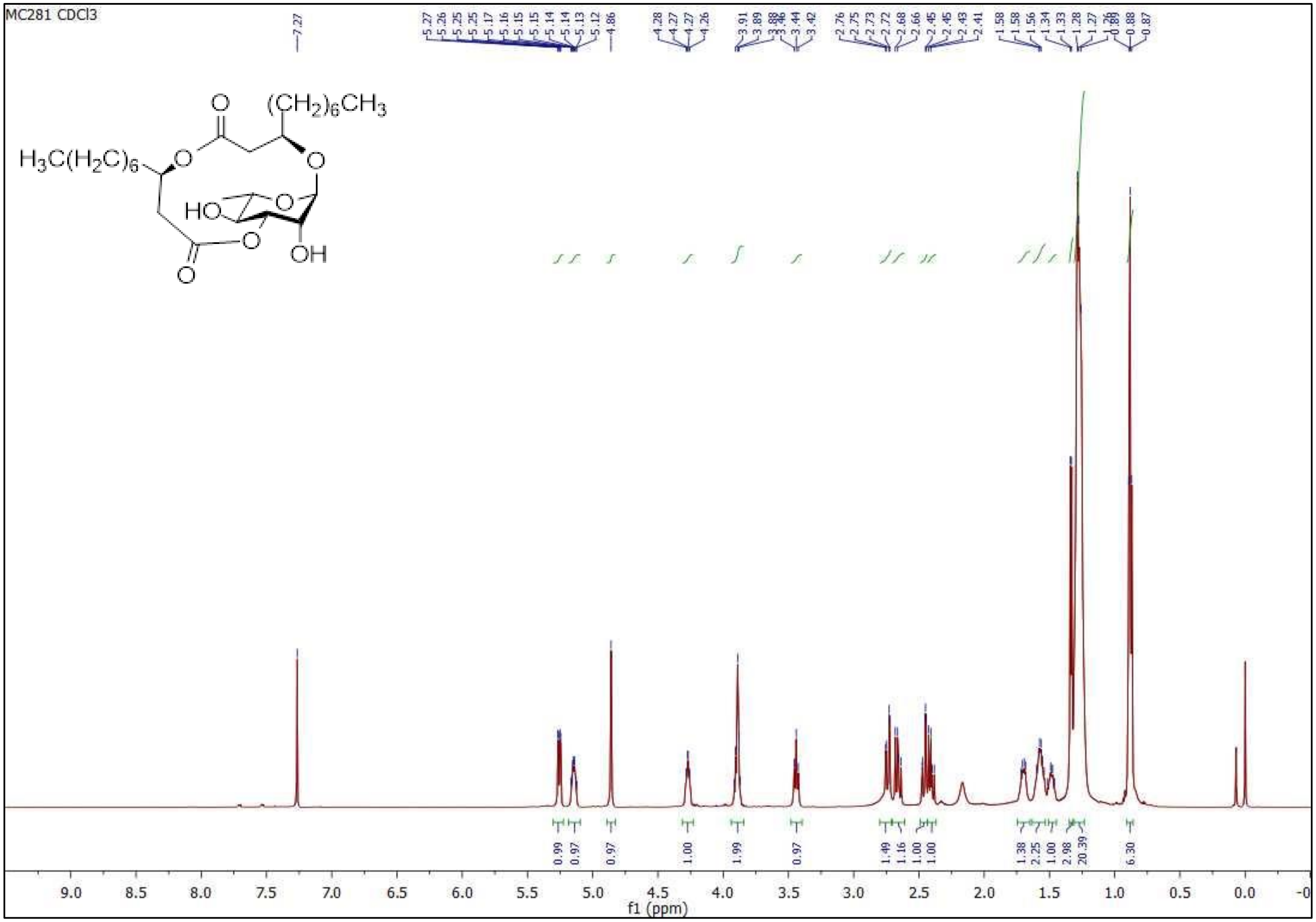

Figure S207 | COSY NMR spectrum ($CDCl_3$, 600 MHz) of (1→3)-macrolactonized rhamnolipid **6*α***.

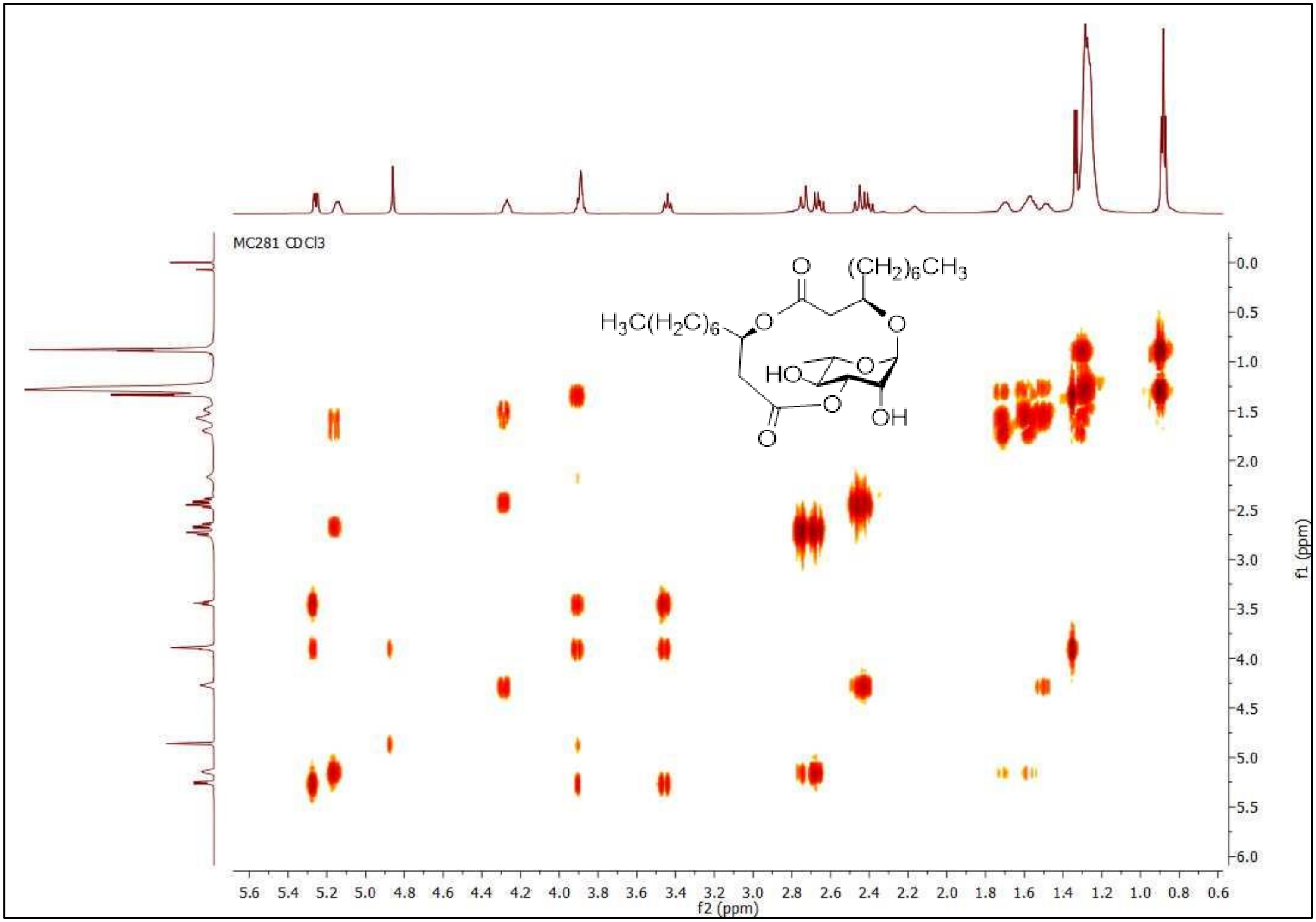

Figure S208 | ^{13}C NMR spectrum ($CDCl_3$, 600 MHz) of (1→3)-macrolactonized rhamnolipid **6*α***.

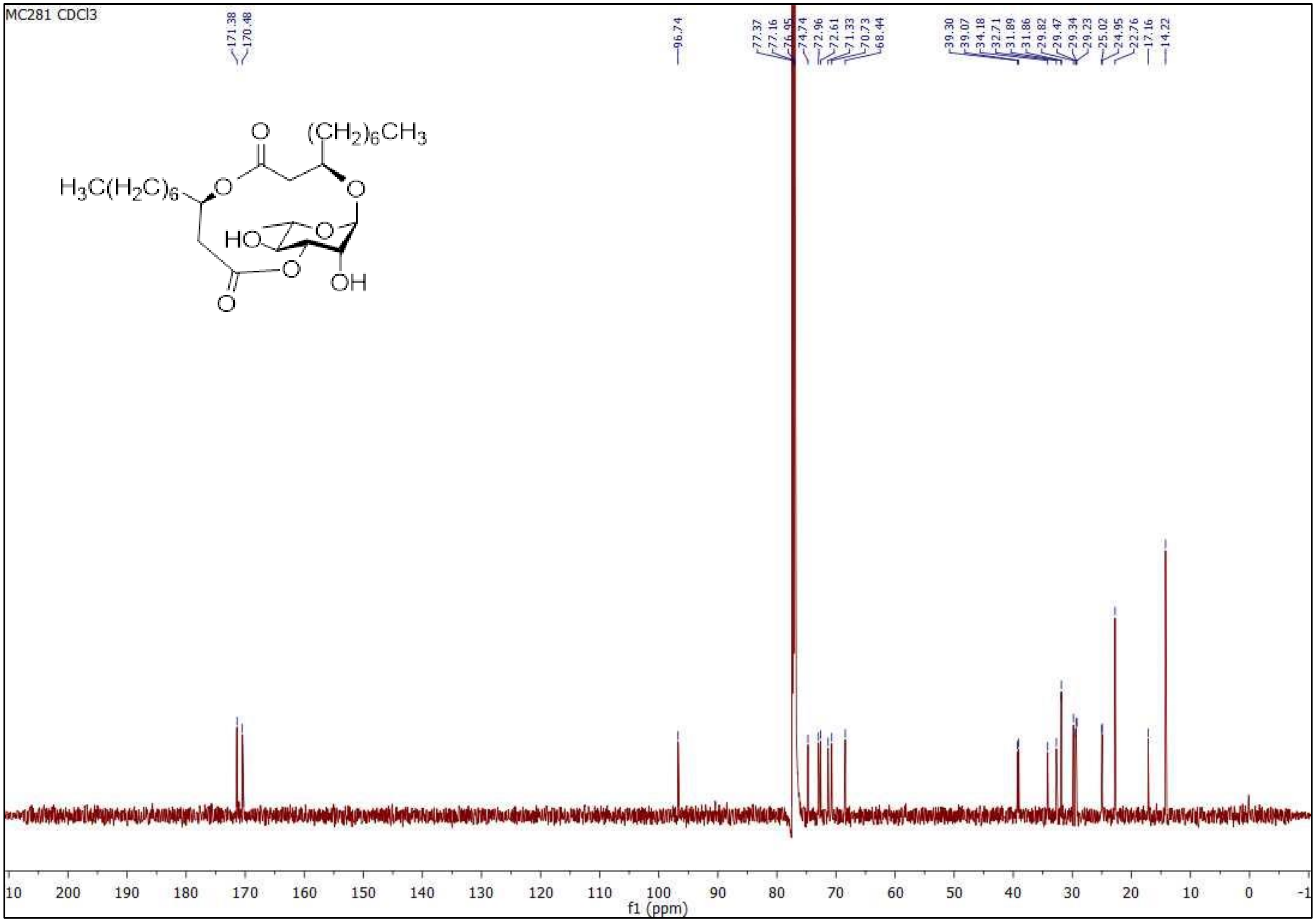

Figure S209 | HSQC NMR spectrum ($CDCl_3$, 600 MHz) of (1→3)-macrolactonized rhamnolipid **6*α***.

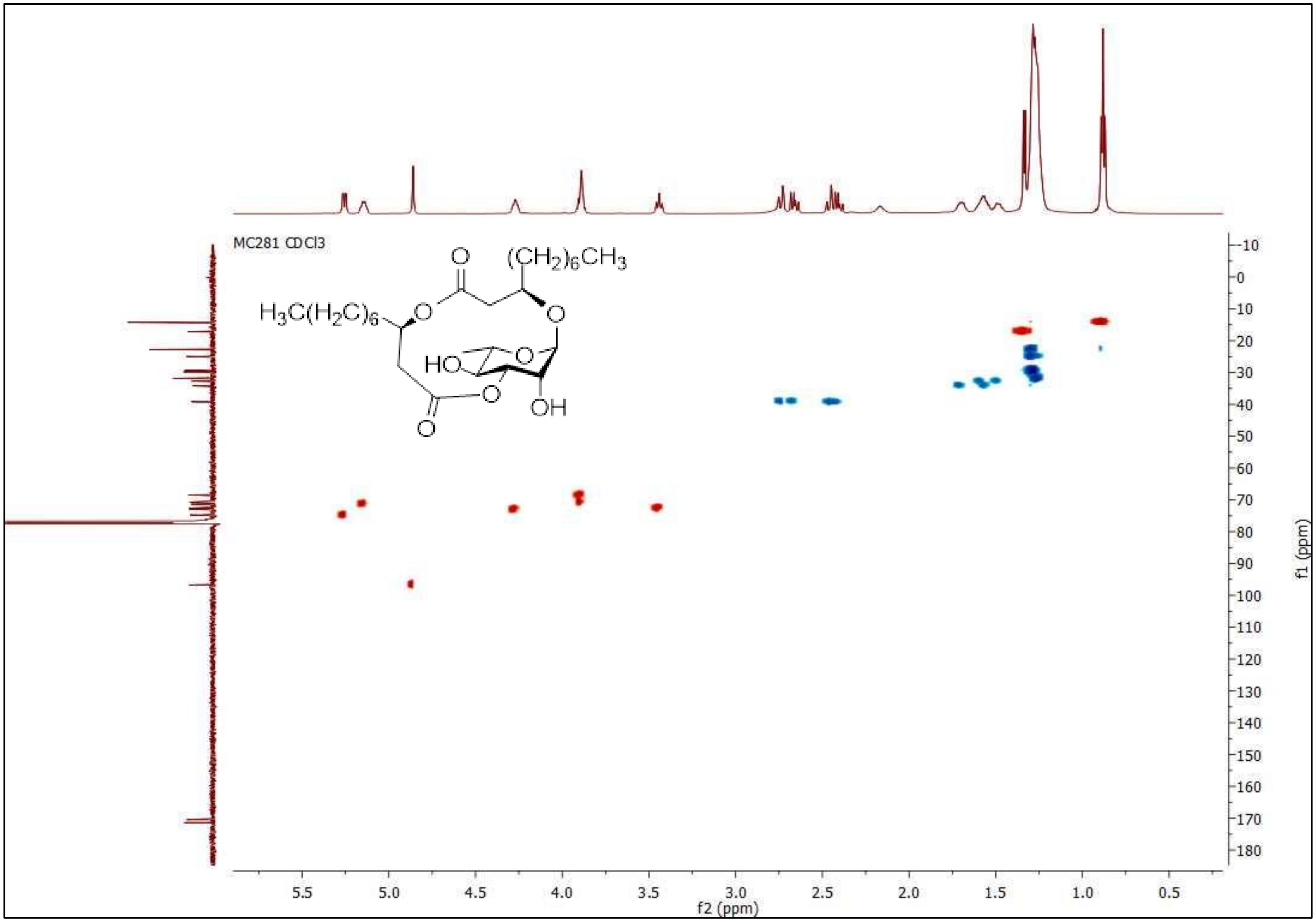

7. HPLC Chromatograms of Synthetic and Natural Surfactants

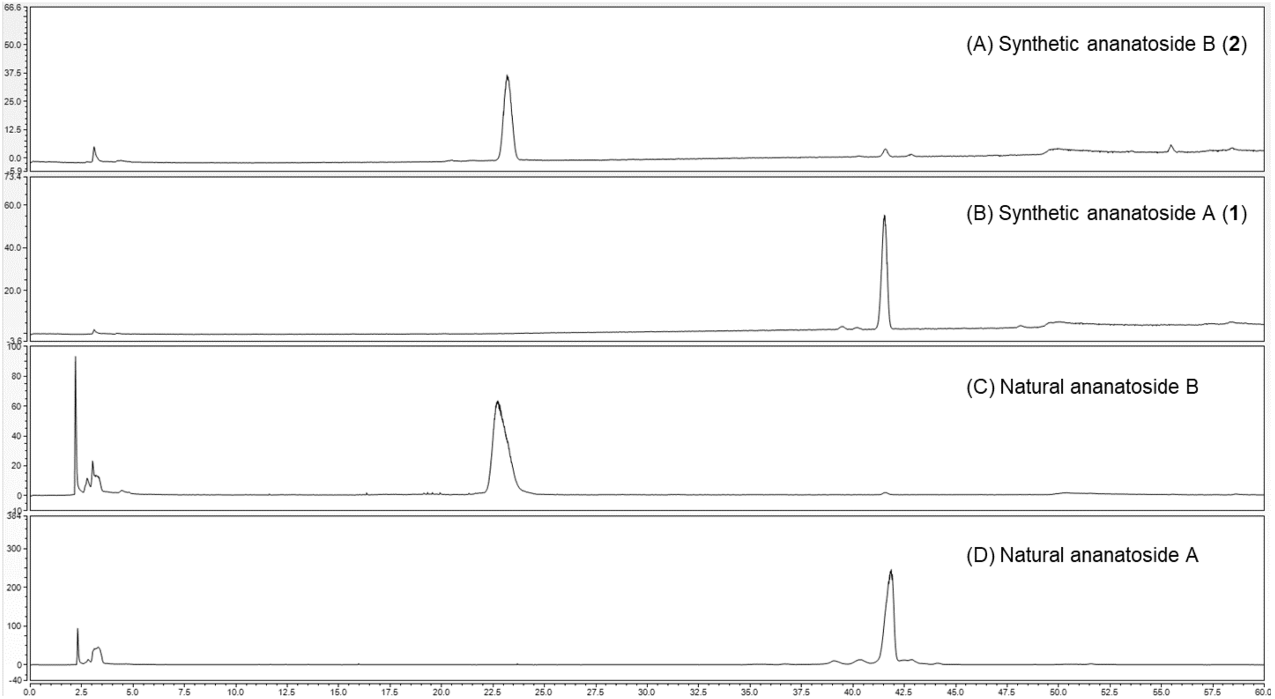

Figure S210. HPLC-CAD chromatograms of (A) synthetic ananatoside B (**2**); (B) synthetic ananatoside A (**1**); (C) natural ananatoside B (**2**); and (D) natural ananatoside A (**1**).

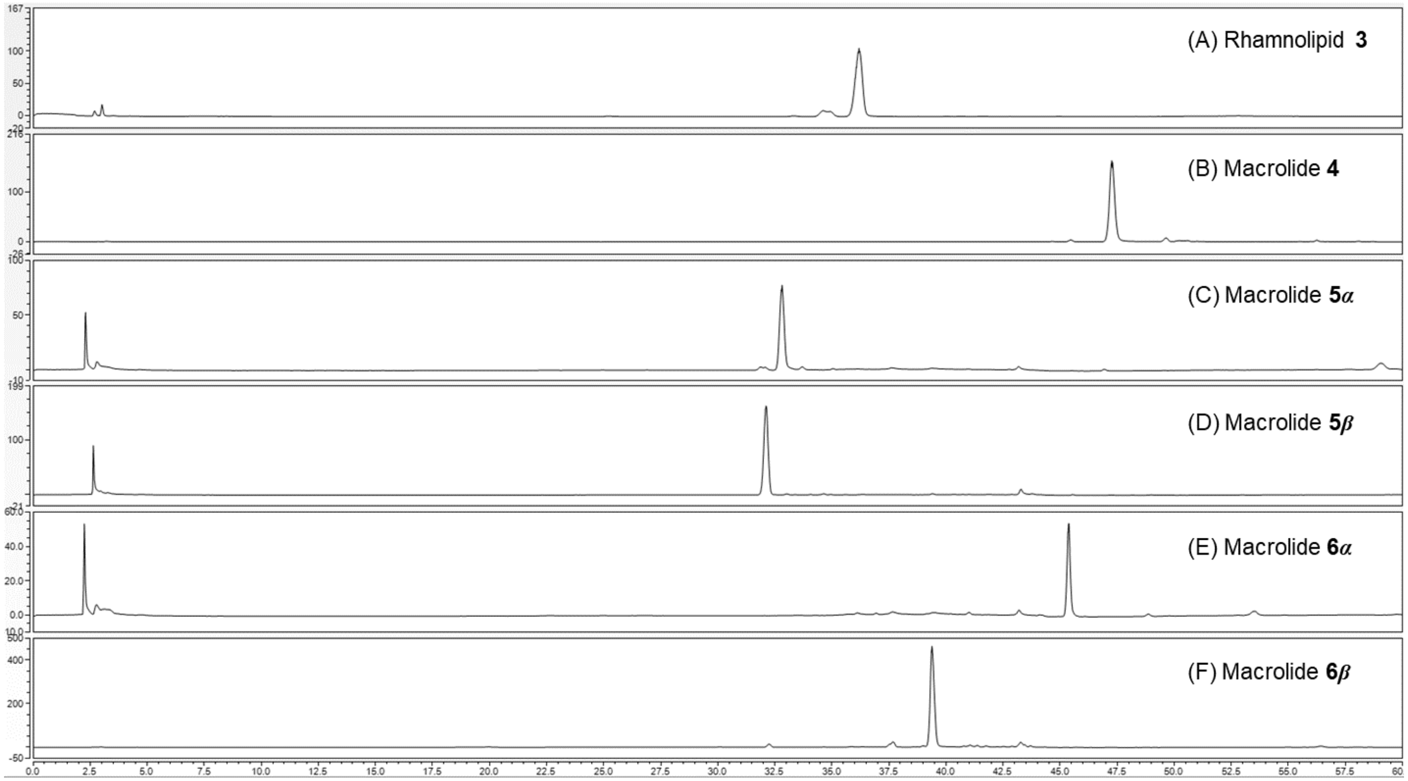

Figure S211. HPLC-CAD chromatograms of synthetic compounds (A) **3**; (B) **4**; (C) **5*α***; (D) **5*β***; (E) **6*α*** and (F) **6*β***.

8. References

1. Gauthier, C.; Lavoie, S.; Piochon, M.; Martinez, S.; Milot, S.; Déziel, E., Structural determination of ananatoside A: an unprecedented 15-membered macrodilactone-containing glycolipid from *Pantoea ananatis*. *Carbohydr. Res.* **2019**, *471*, 13-18.
2. De Vleeschouwer, M.; Sinnaeve, D.; Van den Begin, J.; Coenye, T.; Martins, J. C.; Madder, A., Rapid total synthesis of cyclic lipodepsipeptides as a premise to investigate their self-assembly and biological activity. *Chem. – Eur. J.* **2014**, *20*, 7766-7775.
3. Bauer, J.; Brandenburg, K.; Zähringer, U.; Rademann, J., Chemical synthesis of a glycolipid library by a solid-phase strategy allows elucidation of the structural specificity of immunostimulation by rhamnolipids. *Chem. – Eur. J.* **2006**, *12*, 7116-7124.
4. Hu, Y.-P.; Lin, S.-Y.; Huang, C.-Y.; Zulueta, M.; Liu, J.-Y.; Chang, W.; Hung, S.-C., Synthesis of 3-O-sulfonated heparan sulfate octasaccharides that inhibit the herpes simplex virus type 1 host-cell interaction. *Nat. Chem.* **2011**, *3*, 557-563.
5. Cloutier, M.; Delar, E.; Muru, K.; Ndong, S.; Hoyeck, R. R.; Kaewarpai, T.; Chantratita, N.; Burtnick, M. N.; Brett, P. J.; Gauthier, C., Melioidosis patient serum-reactive synthetic tetrasaccharides bearing the predominant epitopes of *Burkholderia pseudomallei* and *Burkholderia mallei* O-antigens. *Org. Biomol. Chem.* **2019**, *17*, 8878-8901.
6. Pacheco, R. P.; Eismin, R. J.; Coss, C. S.; Wang, H.; Maier, R. M.; Polt, R.; Pemberton, J. E., Synthesis and characterization of four diastereomers of monorhamnolipids. *J. Am. Chem. Soc.* **2017**, *139*, 5125-5132.
7. Cumpstey, I.; Fairbanks, A. J.; Redgrave, A. J., Allyl protecting group mediated intramolecular aglycon delivery (IAD): synthesis of α-glucofuranosides and β-rhamnopyranosides. *Tetrahedron* **2004**, *60*, 9061-9074.

8. Orhan, G.; Bayram, A.; Zer, Y.; Balci, I., Synergy tests by E test and checkerboard methods of antimicrobial combinations against *Brucella melitensis*. *J. Clin. Microbiol.* **2005**, *43*, 140-143.

9. O'Brien, J.; Wilson, I.; Orton, T.; Pognan, F., Investigation of the Alamar Blue (resazurin) fluorescent dye for the assessment of mammalian cell cytotoxicity. *Eur. J. Biochem.* **2000**, *267*, 5421-5426.

10. Gauthier, C.; Legault, J.; Girard-Lalancette, K.; Mshvildadze, V.; Pichette, A., Haemolytic activity, cytotoxicity and membrane cell permeabilization of semi-synthetic and natural lupane- and oleanane-type saponins. *Bioorg. Med. Chem.* **2009**, *17*, 2002-2008.

11. Luzuriaga-Loaiza, W. P.; Schellenberger, R.; De Gaetano, Y.; Akong, F. O.; Villaume, S. A.; Crouzet, J.; Haudrechy, A.; Baillieul, F.; Clément, C.; Lins, L.; Allais, F.; Ongena, M.; Bouquillon, S.; Deleu, M.; Dorey, S., Synthetic rhamnolipid bolaforms trigger an innate immune response in *Arabidopsis thaliana*. *Sci. Rep.* **2018**, *8*, 8534.

12. Smith, D. D. N.; Nickzad, A.; Déziel, E.; Stavrinides, J., A novel glycolipid biosurfactant confers grazing resistance upon *Pantoea ananatis* BRT175 against the social amoeba *Dictyostelium discoideum*. *mSphere* **2016**, *1*, e00075-15.

13. Díaz De Rienzo, M. A.; Kamalanathan, I. D.; Martin, P. J., Comparative study of the production of rhamnolipid biosurfactants by *B. thailandensis* E264 and *P. aeruginosa* ATCC 9027 using foam fractionation. *Process Biochem.* **2016**, *5*, 820-827.

14. Lu, P. J.; Fu, W. E.; Huang, S. C.; Lin, C. Y.; Ho, M. L.; Chem, Y. P.; Cheng, H. F., Methodology for sample preparation and size measurement of commercial ZnO nanoparticles. *J. Food Drug Anal.* **2017**, *26*, 628-636.

15. Hanwell, M. D.; Curtis, D. E.; Lonie, D. C.; Vandermeerschd, T.; Zurek, E.; Hutchison, G. R., Avogadro: An advanced semantic chemical editor, visualization, and analysis platform. *J. Cheminformatics* **2012**, *4* (1), 17.

16. Dalby, A.; Nourse, J. G.; Hounshell, W. D.; Gushurst, A. K. I.; Grier, D. L.; Leland, B. A.; Laufer, J., Description of several chemical structure file formats used by computer programs developed at molecular design limited. *J. Chem. Inf. Comp. Sci.* **1992**, *32,* 244-255.

17. Riniker, S.; Landrum, G. A., Better informed distance geometry: using what we know to improve conformation generation. *J. Chem. Inf. Model.* **2015**, *55*, 2562-2574.

18. Wang, S.; Witek, J.; Landrum, G. A.; Riniker, S., Improving conformer generation for small rings and macrocycles based on distance geometry and experimental torsional-angle preferences. *J. Chem. Inf. Model.* **2020**, *60*, 2044-2058.

19. Adamo, C.; Barone, V., Exchange functionals with improved long-range behavior and adiabatic connection methods without adjustable parameters: The mPW and mPW1PW models. *J. Chem. Phys.* **1998**, *108*, 664-675.

20. Frisch, M. J.; Trucks, G. W.; Schlegel, H. B.; Scuseria, G. E.; Robb, M. A.; Cheeseman, J. R.; Scalmani, G.; Barone, V.; Petersson, G. A.; Nakatsuji, H.; Li, X.; Caricato, M.; Marenich, A. V.; Bloino, J.; Janesko, B. G.; Gomperts, R.; Mennucci, B.; Hratchian, H. P.; Ortiz, J. V.; Izmaylov, A. F.; Sonnenberg, J. L.; Williams-Young, D.; Ding, F.; Lipparini, F.; Egidi, F.; Goings, J.; Peng, B.; Petrone, A.; Henderson, T.; Ranasinghe, D.; Zakrzewski, V. G.; Gao, J.; Rega, N.; Zheng, G.; Liang, W.; Hada, M.; Ehara, M.; Toyota, K.; Fukuda, R.; Hasegawa, J.; Ishida, M.; Nakajima, T.; Honda, Y.; Kitao, O.; Nakai, H.; Vreven, T.; Throssell, K.; Jr., J. A. M.; Peralta, J. E.; Ogliaro, F.; Bearpark, M. J.; Heyd, J. J.; Brothers, E. N.; Kudin, K. N.; Staroverov, V. N.; Keith, T. A.; Kobayashi, R.; Normand, J.; Raghavachari, K.; Rendell, A. P.; Burant, J. C.; Iyengar, S. S.; Tomasi, J.; Cossi, M.; Millam, J. M.; Klene, M.; Adamo, C.;

Cammi, R.; Ochterski, J. W.; Martin, R. L.; Morokuma, K.; Farkas, O.; Foresman, J. B.; Fox, D. J. *Gaussian 16, Revision C.01*: Wallingford, CT, 2019.

21. Wilson, P. J.; Bradley, T. J.; Tozer, D. J., Hybrid exchange-correlation functional determined from thermochemical data and ab initio potentials. *J. Chem. Phys.* **2001**, *115*, 9233-9242.
22. Kendall, R. A.; Dunning Jr, T. H.; Harrison, R. J., Electron affinities of the first-row atoms revisited. Systematic basis sets and wave functions. *J. Chem. Phys.* **1992**, *96*, 6796-6806.
23. Wolinski, K.; Hinton, J. F.; Pulay, P., Efficient implementation of the gauge-independent atomic orbital method for NMR chemical shift calculations. *J. Am. Chem. Soc.* **1990**, *112*, 8251-8260.
24. Willoughby, P. H.; Jansma, M. J.; Hoye, T. R., A guide to small-molecule structure assignment through computation of (1H and 13C) NMR chemical shifts. *Nat. Protoc.* **2014**, *9*, 643-660.
25. Sarotti, A. M.; Pellegrinet, S. C., A multi-standard approach for GIAO ^{13}C NMR calculations. *J. Org. Chem.* **2009**, *74*, 7254-7260.
26. Sarotti, A. M.; Pellegrinet, S. C., Application of the multi-standard methodology for calculating ^{1}H NMR chemical shifts. *J. Org. Chem.* **2012**, *77*, 6059-6065.
27. Lauro, G.; Das, P.; Riccio, R.; Reddy, D. S.; Bifulco, G., DFT/NMR approach for the configuration assignment of groups of stereoisomers by the combination and comparison of experimental and predicted sets of data. *J. Org. Chem.* **2020**, *85* (5), 3297-3306.
28. Bérces, A.; Whitfield, D. M.; Nukada, T., Quantitative description of six-membered ring conformations following the IUPAC conformational nomenclature. *Tetrahedron* **2001**, *57*, 477-491.
